# A method to “gamify” exposure to vegetable flavor and its potential influence on liking

**DOI:** 10.1002/fsn3.4272

**Published:** 2024-06-21

**Authors:** Lissa A. Davis, Elizabeth I. Kielb, Kameron J. Moding, Cordelia A. Running

**Affiliations:** ^1^ Department of Nutrition Science Purdue University West Lafayette Indiana USA; ^2^ Department of Human Development and Family Science Purdue University West Lafayette Indiana USA

**Keywords:** gamification, repeated exposure, sensory acceptance, taste, vegetables

## Abstract

Unpleasant flavor is a commonly stated reason for avoiding vegetables. However, repeated exposure to flavors, even unpleasant vegetable flavors, improves acceptability. Yet, increasing exposure to vegetables is difficult, as vegetables can be less convenient, available, and affordable than other foods. This study describes a method to circumvent these barriers to repeated flavor exposure. We designed a game with vegetable‐ or control‐flavored gummies, where players (*N* = 34) were challenged to identify the flavors over 2 weeks. One round was played per day, and the questions and gummies were designed to make it harder to identify the flavors as the game progressed. At screening, qualified subjects needed to consume <2.5 servings of nonstarchy vegetables per day as well as dislike at least one “target” and one “non‐target” vegetable. The “target” vegetables (kale and broccoli) were included in the game and the “non‐target” vegetables (asparagus and spinach) were included in sensory tests but not the game. Vegetable liking ratings were measured at baseline (before gameplay), after 1 week of gameplay, and after 2 weeks of gameplay. Pilot data indicate one target vegetable (kale) increased in liking after 1 and 2 weeks of gameplay among the vegetable group, but not the control group. Liking for broccoli (other target vegetable), as well as asparagus and spinach (nontarget vegetables), did not significantly change for either group. Thus, our “gamified” approach to vegetable flavor exposure may be useful in increasing acceptance of some vegetables, but additional work to identify why the game improved liking for kale but not broccoli is needed.

## Graphical abstract

Participants were recruited to play a flavor identification game in either the vegetable group (game used vegetable‐flavored gummies) or control group (game used unusual but non‐vegetable‐flavored gummies). After 1–2 weeks of daily gameplay, liking ratings increased for kale but not broccoli for the vegetable but not control group. The game shows potential to improve liking for vegetables, but work should explore why the improvement in liking was unique to kale.

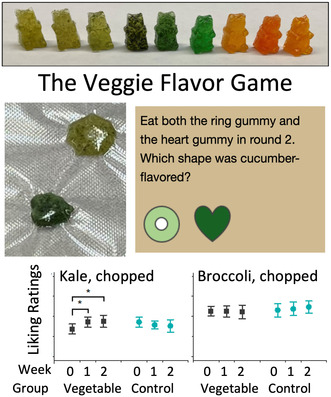

## 1 INTRODUCTION

Vegetables are strongly recommended as part of a healthy diet, as vegetable consumption is associated with decreased risk of many cancers, cardiovascular disease, stroke, and other chronic conditions (Van Duyn & Pivonka, 2000). However, nearly 90% of Americans do not meet recommended intake of vegetables as laid out by the Dietary Guidelines for Americans (U.S. Department of Agriculture and U.S. Department of Health and Human Services, 2020), indicating significant room for improvement. Increasing vegetable intake is challenging because there are often considerable barriers preventing adequate consumption of these foods. Noteworthy barriers include vegetable cost, lack of knowledge of how to choose or cook vegetables, or lack of convenience to procure or prepare vegetables. However, a major barrier to consumption cited across age groups relates to poor flavor of vegetables (Graham et al., 2013; Nicklas et al., 2013).

When it comes to food intake, the relationship between disliking and nonconsumption of foods is actually more consistent than liking and consumption (Hayes, 2020). For example, if a person dislikes kale, they will likely avoid consuming that vegetable. However, if a person likes apples, it does not mean they will seek out apples as a component to every meal, or even to their daily diet. Thus, it may be valuable to focus on decreasing disliking of healthy foods rather than reducing liking of unhealthy foods to influence overall diet quality. Vegetables—dark‐green vegetables specifically—are promising targets for this focus. Dark‐green vegetables such as those in the *Brassica* family (e.g., broccoli, kale, and cabbage) and other leafy greens contain high concentrations of health‐promoting phytochemicals, fiber, and micronutrients, and yet also are often less liked due to aversive sensory properties including bitterness, astringency, and sulphureous smells (Dinehart et al., 2006; Wieczorek et al., 2018).

Multiple studies have demonstrated that repeated exposure to vegetables improves acceptance (for systematic reviews and meta‐analyses, see Appleton et al., 2018; Bell et al., 2021; Spill et al., 2019). While many of these studies are conducted in children, data from adults also suggest that repeated exposure increases liking for vegetables and other initially disliked foods (Bingham et al., 2005; De Leon et al., 2020; Go et al., 2017; Song et al., 2019). Intriguingly, despite the approaches using repeated exposure appearing to work similarly in adults and children, there are remarkably fewer studies in adults attempting to increase vegetable liking through repeated exposure. Regardless of this, from a practical perspective, implementing repeated exposure strategies can be difficult for consumers. Repeated exposure inherently requires multiple tastings of disliked or rejected food, which can lead to food waste (Holley et al., 2018). That waste is impractical and unaffordable in households with limited time and/or financial resources.

Another concept used to try to improve vegetable acceptance is “gamification,” or using game elements within nongame contexts (Deterding et al., 2011). Gamification has been applied to dietary interventions with children to promote healthy eating in many different ways, including goals related to increased fruit and vegetable intake (Chow et al., 2020). Often employed in schools or classrooms, a majority of studies have demonstrated positive impacts of gamification strategies on fruit and vegetable intakes or other measures of behavior (Chow et al., 2020). While evaluation of these strategies in adults is more limited, several studies have indicated that gamification may be a promising strategy to increase fruit and vegetable consumption in adolescents and young adults (Nour et al., 2018; Yoshida‐Montezuma et al., 2020).

Thus, we endeavored to combine gamification with repeated exposure to vegetable flavors. Here, we present a method where we repeatedly expose subjects to vegetable flavor as gummies. Those gummies are used in a “game” that challenges players to identify the flavor of the gummy. To test whether repeatedly tasting the vegetable flavor as a gummy would result in increased liking for actual vegetables, we included two target vegetable flavors in the game: kale and broccoli. Additionally, we created a second (control) game that only included nonvegetable atypical gummy flavors (e.g. chicken and oats). Players in both games were recruited from a screening visit where we confirmed they initially disliked at least one of the target vegetables (kale or broccoli, flavors included in the game), disliked at least one of the nontarget vegetables (asparagus or spinach, flavors not included in the game), and consumed less than 2.5 servings of nonstarchy vegetables per day. Using sensory evaluations before, after 1 week, and after 2 weeks of gameplay, we were able to compare pilot data on whether the game using gummies influenced liking for chopped vegetables.

The primary goal of the game is to increase liking ratings for chopped vegetable samples. Thus, for our pilot test of the game, our hypothesis was that target vegetable liking ratings would increase for the vegetable flavor game group (“Vegetable Group”) but not for the control group (“Control Group”). We also hypothesized that vegetables that were not included in the game as flavors would not change in liking ratings.

## 2 METHODS

The study was approved by the Purdue Institutional Review Board (IRB‐2021‐1742), and all participants provided written informed consent. The study, analyses, and data are registered in the Open Science Framework as part of Project nb9d3 (https://osf.io/nb9d3).

### 2.1 Subject screening and eligibility

**FIGURE 1:**
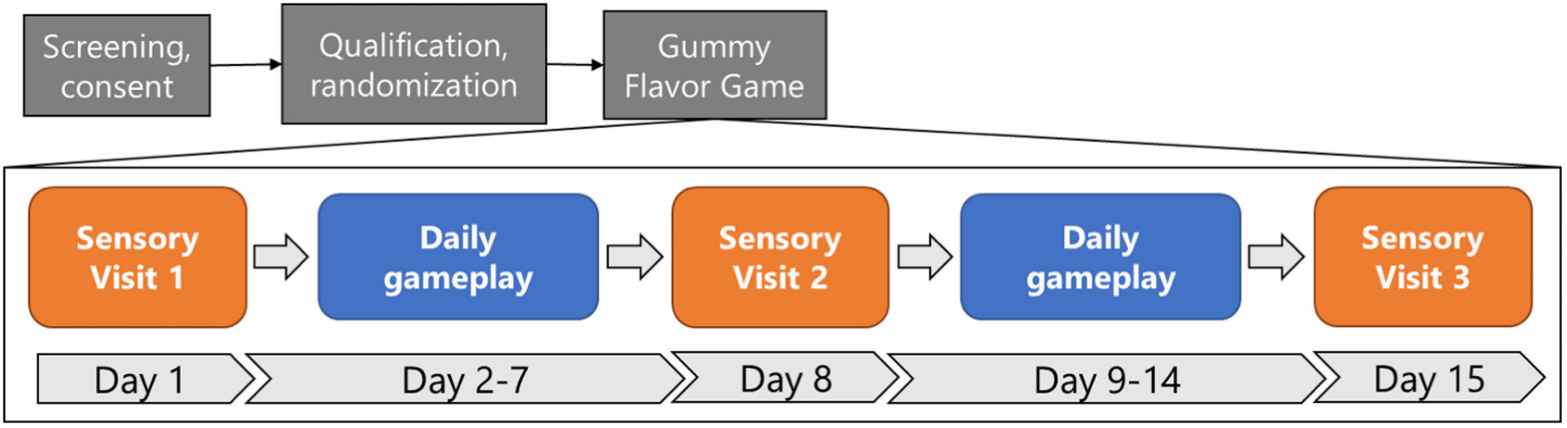
Study overview.

Subjects were recruited for this study via the Saliva, Perception, Ingestion, and Tongues (SPIT) Lab participant database and through local advertisements, flyers, and social media. An overview of the complete study is provided in Figure 1. First, individuals were pre‐screened online (Qualtrics XM, Provo, and UT). Individuals were eligible if they: (1) were between 18 and 25 years of age; (2) were willing and able to consume all study foods and ingredients; (3) agreed to comply with study protocols; and (4) had access to a computer or smartphone device to complete the study questionnaires. Individuals were excluded from the study if they: (1) had self‐reported Type I or Type II diabetes; (2) had food allergies or dietary restrictions to any of the study ingredients, or severe food allergies of any kind; (3) were under the care of a medical professional or taking medications for dry mouth/xerostomia; (4) had a history of choking, trouble swallowing, or dysphagia; or (5) currently smoked, vaped, or used other tobacco products. We selected ages 18–25 as college‐age individuals were the target population for this trial of the “game.”

### 2.2 Game qualification

To qualify for the game phase of the study, participants at the qualification visit needed to:

- Have no signs of smell dysfunction, as assessed by a Sensonics International Pocket Smell Test™ (Haddon Heights, NJ)
- Have no signs of taste dysfunction, as assessed by recognizing 5.0% w/w sucrose as sweet, 1.2% w/w sodium chloride as salty, 0.27% w/w citric acid as sour, and 0.016% w/w sucrose octaacetate as bitter.
- Consume fewer than 2.5 servings of nonstarchy vegetables per day based on survey responses to the Food Attitudes and Behaviors Questionnaire (Erinosho et al., 2015)
- Rate at least one target (broccoli or kale) and one nontarget (asparagus or spinach) chopped vegetable in the sensory evaluation task at or below “Neutral” on our hedonic visual analog scale (more details below on samples and scale design)

Full details for these qualification metrics are available in the supplemental files and at https://osf.io/nb9d3. Of 77 participants who completed the qualification visit, 39 qualified to participate in the gummy flavor game.

### 2.3 Gummy flavor game

#### 2.3.1 Game levels

**FIGURE 2:**
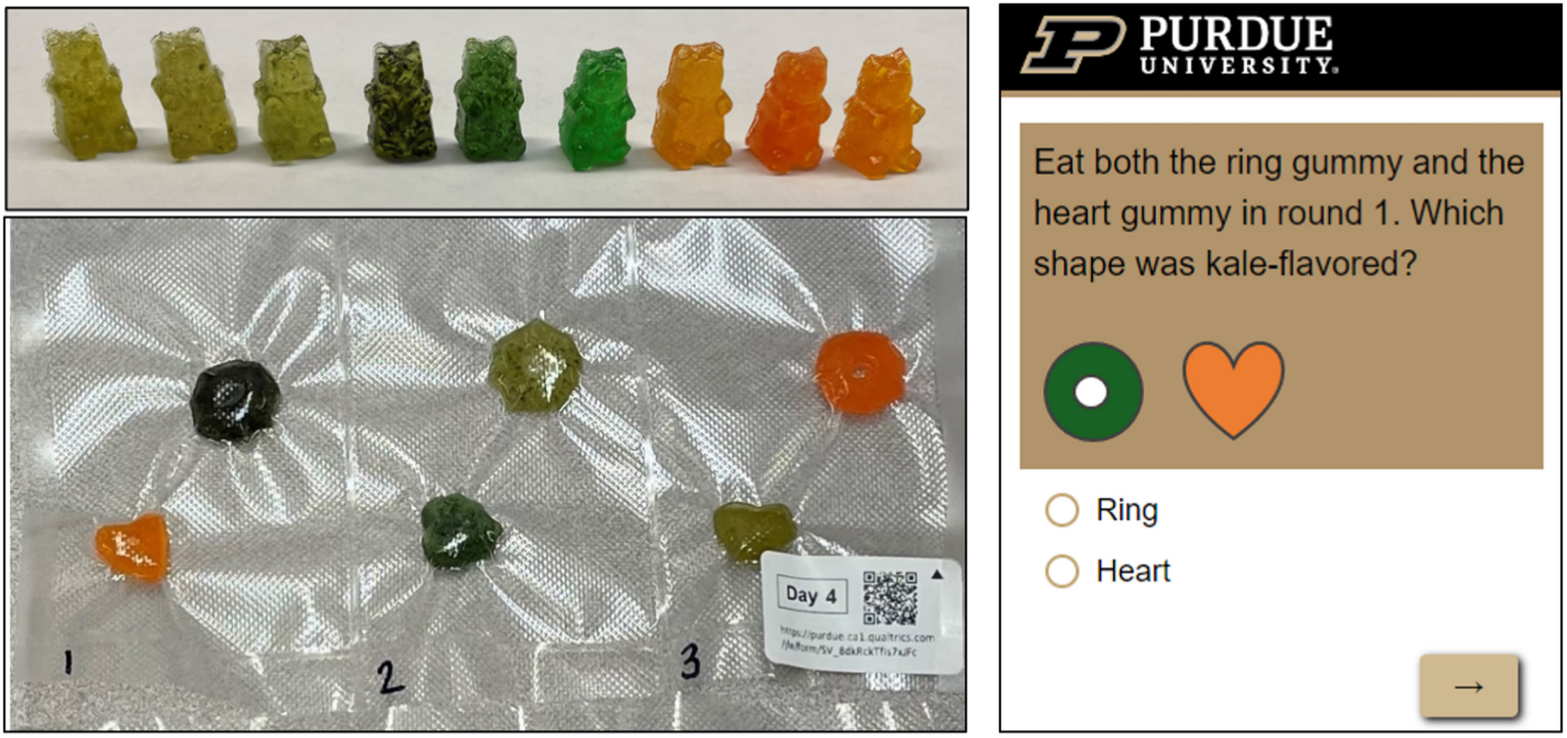
Top left: Example of bear‐shaped vegetable‐flavored gummies. Bottom left: Example of game‐level package. Right: Example of survey question for the vegetable group.

Participants were provided with six “levels” (in separate packages) of the gummy flavor game (see Figure 2 below for an example) to play per week. Full details for all the levels are available in the study registration at https://osf.io/nb9d3. Participants were asked to play the game once daily at least five times per week for 2 weeks, for a total of 10–12 “levels” of play. Each package contained a different combination of flavored and colored gummies, which provided a range of difficulty levels. A label on each package denoted the level number and displayed a QR code/link to a Qualtrics (Provo, UT, USA) survey for gameplay. The gameplay survey was also used to track participant progress and compliance.

The gameplay survey asked flavor identification questions using a variety of formats. Each level package contained six gummies, but the gummies were differently flavored, shaped, and colored to alter the difficulty level. Thus, the overall added sugar and calories from gummy consumption varied from 6 to 15 g of added sugar and 30 to 75 kcals per day. Gameplay during days 2–7 (week 1) of the game used “easy,” color‐congruent levels, where gummy colors matched their respective flavor (e.g., a light‐green broccoli gummy). Gameplay during days 9–14 (week 2) of the game used “difficult,” color‐incongruent levels, where gummy colors did not match their respective flavor (e.g., a blue broccoli gummy). Each week of gameplay contained questions with either two or four answer options, which varied the question difficulty and the probability of getting a question right by chance (50% chance for two answer options and 25% chance for four answer options). Full gameplay questions and gummies included at each level are included in the OSF registration and in the Supplemental Files.

Gameplay compliance was monitored online. If, by the second sensory visit, the participants had not completed the surveys on at least 5 days that week, they were removed from the study for noncompliance. The same compliance check was completed at the third sensory visit. Two participants were removed due to noncompliance with gameplay.

#### 2.3.2 Gummy flavors

We formulated two sets of flavored gummies: one for the control group and one for the vegetable group. The “Vegetable” set of gummies contained primarily vegetable flavors, while the “Control” set of gummies contained nonvegetable flavors that were unfamiliar or unpleasant in gummy form, such as meats, herbs, grains, or legumes. The gummies had a gelatin, sucrose, and corn syrup base, with small variations in formulation for each flavor. Most gummies were made from foods, but some gummies in the control group included natural and artificial flavors. The general formulation (all w/w) for gummies was approximately:6% gelatin, 33% sucrose, 34% corn syrup, 21% juice or water, 0.8% citric acid solution, 0.1–8% puree or flavor, and < 0.1% food coloring. The overall preparation method follows, but specifics for each gummy flavor are in the supplemental files and the OSF registration.

Generally, beef gelatin (250 bloom; Modernist Pantry, Eliot ME) was mixed with granulated sucrose (local grocery retailer). Separately, water or juice (depending on gummy flavor, see Supplemental Table S2 and S3) was mixed with light corn syrup (Karo® Syrup, Freshmeadow, NY). Next, the corn syrup mixture was combined with the gelatin/sucrose mixture and rested for 5 minutes. The mixture was then heated to approximately 105.0°C, stirring frequently. Next, the mixture was cooled to 95°C. Then, citric acid solution (50% Milliard™ citric acid, [Lakewood, NJ] in deionized water), food coloring (Chefmaster®, Fullerton, CA), puree, and/or natural/artificial flavor (details in supplement) were added. Once all ingredients were incorporated, the syrup was poured into assorted silicone molds (1–3 mL capacities), cooled for 30 minutes, and refrigerated at 4°C overnight. Finally, gummies were removed from silicone molds and coated with mineral oil (Kroger, Cincinnati, OH).

The gummies were formulated to be safe to store at room temperature, with a water activity below 0.85 and pH below 4.5. To limit loss of fresh flavor, we froze gummies in vacuum‐sealed packages until gameplay.

### 2.4 Sensory evaluation of vegetables

To evaluate if our game influenced liking for chopped vegetables, we conducted sensory tests before (baseline), after 1 week of gameplay (week 1), and after 2 weeks of gameplay (week 2). From our qualification visit, 39 subjects qualified for the research study on whether the game influenced liking for vegetables. Two participants dropped out before starting the game, one dropped out during the game due to illness unrelated to the game, and two were dropped from the game due to noncompliance (not playing the game at least five times per week). All three dropouts during the game occurred in the control group. Thus, 34 participants completed the game phase (24 women, 10 men, average age 22, age range 19–25); full demographic information can be found in the Supplemental Files.

Participants were randomly assigned to either the vegetable group (gameplay with vegetable‐flavored gummies, *N* = 18) or the control group (gameplay with nonvegetable‐flavored gummies, *N* = 19 but 3 dropped (1 due to unrelated illness and 2 due to noncompliance) out; for final, *N* = 16).

In the qualification visit, baseline visit, week 1 visit, and week 2 visit, participants evaluated four chopped vegetable samples and eight pureed food samples, all served cold. The chopped vegetables included four green vegetables that are often disliked: kale, spinach, asparagus, and broccoli. Two of these would function as target vegetables (kale and broccoli, included in the vegetable group's flavor game), and two of these as nontarget vegetables (spinach and asparagus, not included in any game). We included the nontarget vegetables in order to assess whether general liking for vegetables would change in response to the game, or if any changes were specific to flavors included in the game. Pilot data indicated that people could accurately pair the kale and broccoli gummies to their identity, indicating that these gummies did taste like the actual vegetables. Pilot data for spinach and asparagus indicated similar levels of liking as kale and broccoli, but poorer performance on matching the gummies to the flavors. Thus, these were used as the nontarget (not included in gummy game) vegetables, as their gummies seemed to taste less like the actual vegetables. Details for these pilot data can be found in L. Davis' published doctoral thesis (Davis, 2023).

All four of the vegetables were also evaluated as purees. The remaining four pureed samples (oats, black beans, beef, and chicken) were included as nonvegetable distractor samples. These nonvegetable samples also served to increase difficulty during a sample identification task. All vegetable samples were made from locally purchased frozen vegetables. Distractor samples were made from ingredients purchased from local and online retailers. Full details on how the purees and chopped samples were prepared are found in the Supplemental Files, but a summary is included below.

Pureed vegetables were cooked using a 1200 W microwave from frozen, slightly longer than package directions to ensure uniform sample texture. Each vegetable type was pureed with enough de‐ionized water to produce a thick puree. Purees were cooled to room temperature, packaged into opaque barrier pouches (ULINE, Pleasant Prairie, WI) to minimize visual cues, and frozen at −20°C until use. Chopped vegetables were cooked using a 1200 W microwave from frozen according to package directions, cooled to room temperature, packaged in 30‐mL clear plastic souffle cups (Dart Container Corporation, Mason, MI), and frozen at −20°C until further use. Test samples were thawed overnight in a 4°C refrigerator before packaging to give to participants. All samples were evaluated cold.

Participants rated bitterness, sweetness, and liking/disliking of each sample, followed by a four‐option multiple‐choice question that asked participants to identify the sample. Bitterness and sweetness intensity were rated on 110‐pt visual analog scales, with labels corresponding to the following points: “None” = 0, “Barely detectable” = 5, “Weak” = 25, “Moderate” = 45, “Strong” = 65, “Very strong” = 85, and “Strongest ever” = 105 (adapted from Kershaw & Running, 2019). Liking/disliking was rated on a hedonic visual analog scale ranging from −110 to 110, with labels corresponding to the following points: “Worst ever” = −100, “Dislike” = −50, “Neutral” = 0, “Like” = 50, and “Best ever” = 100. Participants evaluated all eight pureed samples first and then evaluated all four chopped samples, but sample order was randomized within the purees and chopped samples. Sample re‐tasting during rating was allowed.

### 2.5 Data analysis

Our pilot data indicated that to find a mean difference of at least 20 pts on our sensory scales, at least 17 subjects per group were required (assumes SD = 35, alpha 0.05, within‐subject correlation of ratings of 0.7, two‐sided test). 20 pts is equal to the difference between most labels on our sweetness and bitterness intensity scales and is under half the difference between markings on our hedonic scale (markings are 50 pts apart). Thus, the study concluded slightly under our recruitment goal for the control group due to the number of subjects who dropped out, but we had adequate sample size in our vegetable group to find a difference of at least 20 pts (note: however, per results below, the control group ended up with no differences near 20 pts, so an additional subject would be unlikely to alter any effects).

Sensory data from this study were analyzed with linear mixed models using the MIXED procedure in SAS OnDemand (Cary, NC, USA), implemented using notebooks running Python 3 in Jupyter Lab. All data were evaluated for outliers, defined as points 1.5 × (interquartile range) above quartile 3 or below quartile 1. No outliers were detected.

#### 2.5.1 Descriptive analysis of game performance

We assessed how well people performed in the game, mainly to assess whether the game was too difficult (very few participants achieving correct answers) or not difficult enough (almost all participants achieving correct answers). Game performance was not compared to other outcomes, as the study was not powered to evaluate how “correctness” in the game could influence outcomes.

We counted the number of correct responses across vegetables/controls, specifically focusing on our target vegetables versus other vegetables versus the control group questions. Additionally, we counted the correct responses by whether the question was from week 1 when colors were congruent with expected vegetable color, and week 2 when the colors were incongruent. Counts were also separated based on whether the question had a 0.5 or 0.25 chance of getting the question correct by guessing. These counts were designed as a quick look to see generally how people were performing in the game. Binomial tests were run on the counts to determine if participants were performing better than chance within each of these categories.

#### 2.5.2 Primary analysis: Effect of game on vegetable ratings

The primary outcomes of interest were liking/disliking ratings of the vegetable samples across the game phase, with bitterness and sweetness intensity ratings as secondary outcomes of interest. Analyses were run separately for each sample. Model statements for analysis of sensory data were as follows:

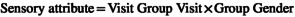




Sensory attribute refers to liking, sweetness intensity, or bitterness intensity. Visit refers to the sensory evaluation visit during the game phase (baseline [before game], week 1 [after 1 week of play], or week 2 [after 2 weeks of play]). Group refers to whether the participant was in the vegetable or control game group. Visit × Group tests the interaction of the two, or whether the groups differed in ratings over the course of the game. Gender was tested as a covariate, and slightly improved the fit of the model per the BIC score. Thus, gender was kept in the model as a covariate.

Distributions of residuals during the initial model‐fitting process showed heteroskedasticity for sweetness intensity, and thus a square root transformation was applied only to that sensory attribute.

For each model, participant was included as a repeated factor, Kenward–Roger approximation was used for degrees of freedom (Kenward & Roger, 1997; *SAS Help Center: Kenward‐Roger Degrees of Freedom Approximation*, n.d.), and compound symmetry was used as the covariance structure.

Our primary outcomes (effects of game on liking within each group, and comparisons of liking ratings between each group by visit) appear in the interaction term Visit × Group, which would include many nonsensical comparisons (such as baseline liking in the control group compared to week 2 liking in the vegetable group). Thus, to assess relevant effects within the Visit × Group interaction term, contrast statements were applied based on a priori comparisons of interest. These included within‐group comparisons between each sensory visit (six total comparisons and three within each group) to assess the effect of the games over time. Additionally, we included between‐group comparisons within each visit (three total comparisons) to observe whether differences appeared between groups over time. Alpha was set at 0.05, with no adjustments as these contrasts were designated a priori.

Detailed code and output files for all models can be found in the supplemental materials. Data are available at the OSF registration at https://osf.io/nb9d3.

#### 2.5.3 Secondary analyses

After we finished the primary analysis, we wanted to explore potential explanations for why we observed effects for one vegetable but not others. Thus, to augment our understanding of the results observed in the models described above, we added two additional analyses. First, we conducted simple paired t‐tests for all vegetable liking ratings comparing baseline to week 1 and baseline to week 2. This was to confirm in a simpler statistical model whether the patterns were consistent. Additionally, to assess whether differences in average baseline liking between the vegetables might contribute to outcomes, we ran linear mixed models for each visit (baseline, week 1, and week 2) with the samples as a variable:

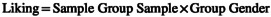




This model allowed us to evaluate if the samples differed in liking at the different visits, which helps interpret the results if one sample changes but another does not after the intervention.

As before, the Kenward–Roger approximation for degrees of freedom and compound symmetry for covariance structure was used. Code is included in the supplemental files.

## 3 RESULTS

Details for each analysis follow. We show here the data for vegetable samples (chopped and pureed), but data for the distractor samples (pureed oats, beef, chicken, and black beans) are in the supplemental files.

### 3.1 Descriptive analysis of game performance

Participant performance across the game phase is displayed in Table 1. All question types demonstrated average performance of greater than 50% regardless of flavor or difficulty. Considering questions grouped by flavor, congruency, and chance correct, all sets of questions perform better than chance (the highest binomial test p‐value was 0.0032), indicating that at least some participants in both vegetable and control groups were able to correctly identify the gummy flavors while playing the game.

**TABLE 1:** Game performance across days and groups.

Day	Congruency	Details	Chance	Correct/total, (%correct)	
				Vegetable group	Control group
2	Congruent	Select identity from a list of two options; color congruent with food item	0.5	91/102, 89%	74/108, 69%
3	Congruent	Select identity from a list of two options; color congruent with food item	0.5	81/108, 75%	81/114, 71%
4	Congruent	Eat two different shapes in different colors; identify which was specific flavor; color congruent with food item	0.5	48/48, 100%	54/54, 100%
5	Congruent	Eat two different shapes in same color; identify which was specific flavor; color congruent with food item	0.5	39/51, 76%	35/51, 69%
6	Congruent	Select identity from a list of four options; color congruent with food item	0.25	76/108, 70%	73/108, 68%
7	Congruent	Select identity from a list of four options; color congruent with food item	0.25	80/108, 74%	69/102, 68%
9	Incongruent	Eat two different shapes in different colors; identify which was specific flavor; color masked but different within pair	0.5	48/51, 94%	45/51, 88%
10	Incongruent	Select identity from a list of four options; color masked	0.25	75/108, 69%	72/102, 71%
11	Incongruent	Eat two different shapes in different colors; identify which was specific flavor; color masked but different within pair	0.5	39/51, 76%	42/48, 88%
12	Incongruent	Select identity from a list of four options; color masked	0.25	79/108, 73%	72/90, 80%
13	Incongruent	Eat two different shapes in same colors; identify which was specific flavor; color masked and the same within pairs	0.5	46/51, 90%	37/48, 77%
14	Incongruent	Select identity from a list of four options; color masked and the same (black) for all gummies	0.25	56/102, 55%	65/90, 72%

*Note*: The number of correct/incorrect responses varies across days due to differences in the number of questions/rounds played that day. Participants tasted the same total number of gummies each day to keep tasting exposure consistent. With the different types of questions, this means the number of questions varied to keep the number of tastings constant.

### 3.2 Analysis of game's effect on vegetable liking

From the model: Sensory attribute = Visit Group Visit × Group Gender (evaluated for each sample type).

**FIGURE 3:**
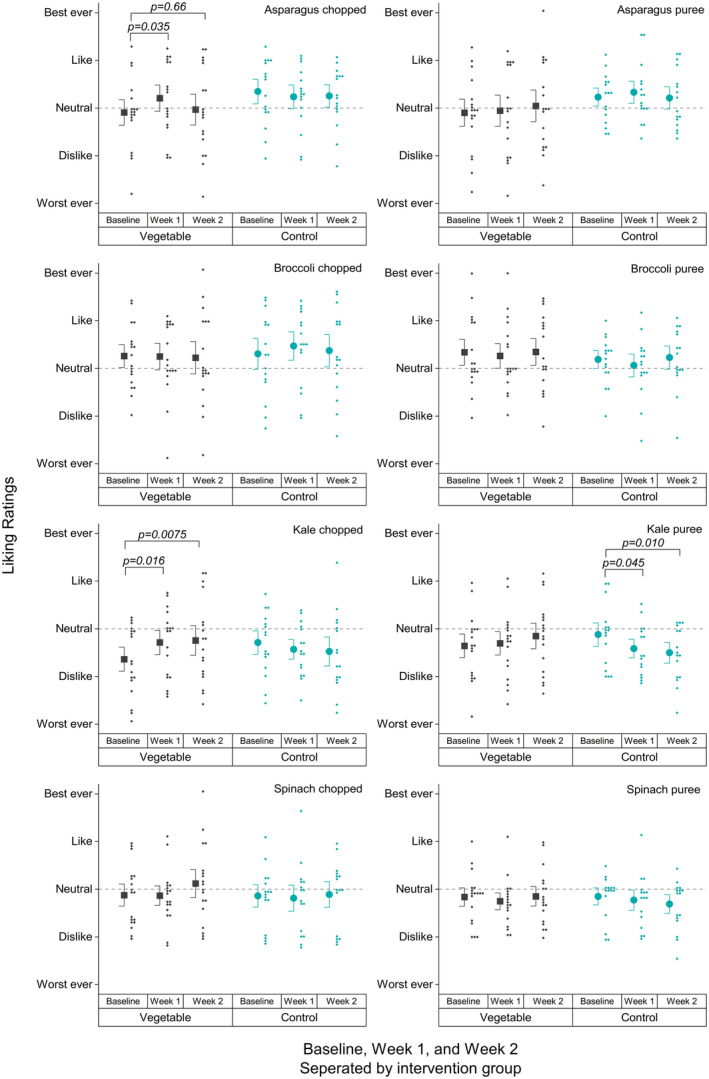
Mean and SE liking ratings of study vegetables across study visits. Large symbols indicate means and bars are SE. Small symbols are individual data points.

Liking ratings for all vegetable samples evaluated at the sensory visits are shown in Table 2 and Figure 3. Nonvegetables sample ratings are in the Supplemental Files.

**TABLE 2:** Least squared mean liking ratings and standard error for samples at each visit. Model (run by sample) Liking = Visit Group (Visit × Group) Gender (gender included as covariate only).

Sample	Group	Week		
		Baseline	Week 1	Week 2
Asparagus chopped	Control	17 ± 10	11 ± 10	12 ± 10
	Vegetable	−6 ± 10	9 ± 10*	−3 ± 10
Asparagus puree	Control	11 ± 10	16 ± 10	10 ± 10
	Vegetable	−5 ± 10	−3 ± 10	2 ± 10
Broccoli chopped	Control	15 ± 11	21 ± 11^†^	16 ± 11
	Vegetable	8 ± 11	8 ± 11	6 ± 11
Broccoli puree	Control	9 ± 9	2 ± 9	11 ± 9
	Vegetable	16 ± 9	12 ± 9	16 ± 9
Kale chopped	Control	−13 ± 9	−20 ± 9^†^	−22 ± 9^†^
	Vegetable	−28 ± 9^†^	−11 ± 9*	−8 ± 9*
Kale puree	Control	−6 ± 8	−21 ± 8*, ^†^	−25 ± 8*, ^†^
	Vegetable	−17 ± 8^†^	−15 ± 8	−7 ± 8
Spinach chopped	Control	−7 ± 9	−10 ± 9	−6 ± 9
	Vegetable	−7 ± 9	−7 ± 9	5 ± 9^^^
Spinach puree	Control	−8 ± 7	−12 ± 7	−16 ± 7^†^
	Vegetable	−8 ± 7	−13 ± 7	−8 ± 7

*Note*: Between groups, the only pair of values that approached being significantly different were the ratings for asparagus, chopped, at baseline (*p* = .092). Tables with detailed p‐values and nonvegetable samples can be obtained in the Supplemental File. Ratings are from a −110 to 110 visual analog scale, with labels corresponding to: “Worst ever” = −100, “Dislike” = −50, “Neutral” = 0, “Like” = 50, and “Best ever” = 100.

* Indicates significantly different from baseline (*p* < .05), within group.

^^^ Indicates trending toward significant difference from baseline (.05 < *p* < .1), within group.

^†^ Indicates value is significantly different from 0, from least‐squared means analysis.

There were no main effects of visit or group on the vegetable liking ratings. This means that averaging across all visits, there is no difference between the control and vegetable groups; additionally, averaging across both groups, there is no difference between visits.

The only effects appear in the contrasts from the interaction term, which includes comparisons within a group across visits, or between groups within visits.

#### 3.2.1 Liking ratings within game groups

Chopped vegetables: The vegetable group of participants displayed improvements in liking ratings for chopped kale over the course of the game (*p* = .016 at week 1 and *p* = .0075 at week 2). The change reflects moving from about −28 (about halfway between “dislike” and “neutral”) on the scale to −8 (just under “neutral”). A possible trend was also observed for the vegetable group's liking ratings of chopped spinach, with ratings trending toward higher at week 2 compared to baseline (*p* = .095), potentially shifting from just below “neutral” to just above “neutral.” However, we were not powered to find a difference this small (a change in rating of about 12 pts), thus this potential effect needs testing in a larger sample. Chopped asparagus increased in liking for the vegetable group at week 1 compared to baseline (*p* = .035), but the difference was lost by week 2 (*p* = .66). The control group's liking ratings did not change for any chopped vegetables.

Pureed vegetables: The vegetable group's liking ratings for pureed vegetables did not change. The control group's liking for pureed kale decreased at week 1 (*p* = .045) and remained decreased at week 2 (*p* = .010). The change reflects moving from barely under the “neutral” mark on our scale to about halfway between “dislike” and “neutral” on our scale.

#### 3.2.2 Liking ratings between game groups

Comparing ratings between the vegetable and control groups, the only difference that approached significance was for ratings of chopped asparagus at baseline (*p* = .092). No other differences are significant between the groups at each visit for any of the vegetables, regardless of whether chopped or pureed.

### 3.3 Secondary outcomes: Sweetness and bitterness

Chopped vegetables, sweetness: For sweetness, the only effect was in the vegetable group, where sweetness of chopped spinach increased comparing baseline to week 2 ratings (*p* = .020).

Chopped vegetables, bitterness: For bitterness, some changes were observed at week 1 but lost at week 2. These included the vegetable group's ratings for broccoli (more bitter at week 1, *p* = .031), and the control group's ratings for chopped asparagus (more bitter at week 1, *p* = .013). The only significant change in bitterness at week 2 was the vegetable group's decreased bitter ratings for chopped spinach (*p* = .013). Between groups, at baseline, the vegetable group found chopped asparagus more bitter than the control group (*p* = .034), but no differences were observed by week 1 or week 2.

Pureed vegetables, sweetness: As with chopped spinach, pureed spinach sweetness increased for the vegetable group by week 2 (*p* = .022).

Pureed vegetables, bitterness: No differences were observed for pureed vegetables sweetness or bitterness.

Full details for all sweetness and bitterness comparisons are in the Supplemental Tables.

### 3.4 Secondary analysis: Paired t‐tests on liking

The outcomes of these tests mirror what we observed in the linear mixed models, comparing liking ratings as baseline to week 1 and baseline to week 2. The same effects appear for increased liking for chopped kale in the vegetable group (*p* = .023 at week 1 and *p* = .043 at week 2), but not the control group. A trend is observed here as well for a very small potential increase in liking for chopped spinach in vegetable group at week 2 (*p* = .069). Decreases in liking are again seen for kale puree in the control group, who disliked samples more at week 1 and week 2 compared to baseline (*p* = .024 and .022). All of these patterns in liking were also observed in the least linear mixed models (Table 2). Thus, these results are in the Supplemental Files.

### 3.5 Secondary analysis: Comparison of liking ratings between vegetables and differences from “neutral”

**FIGURE 4:**
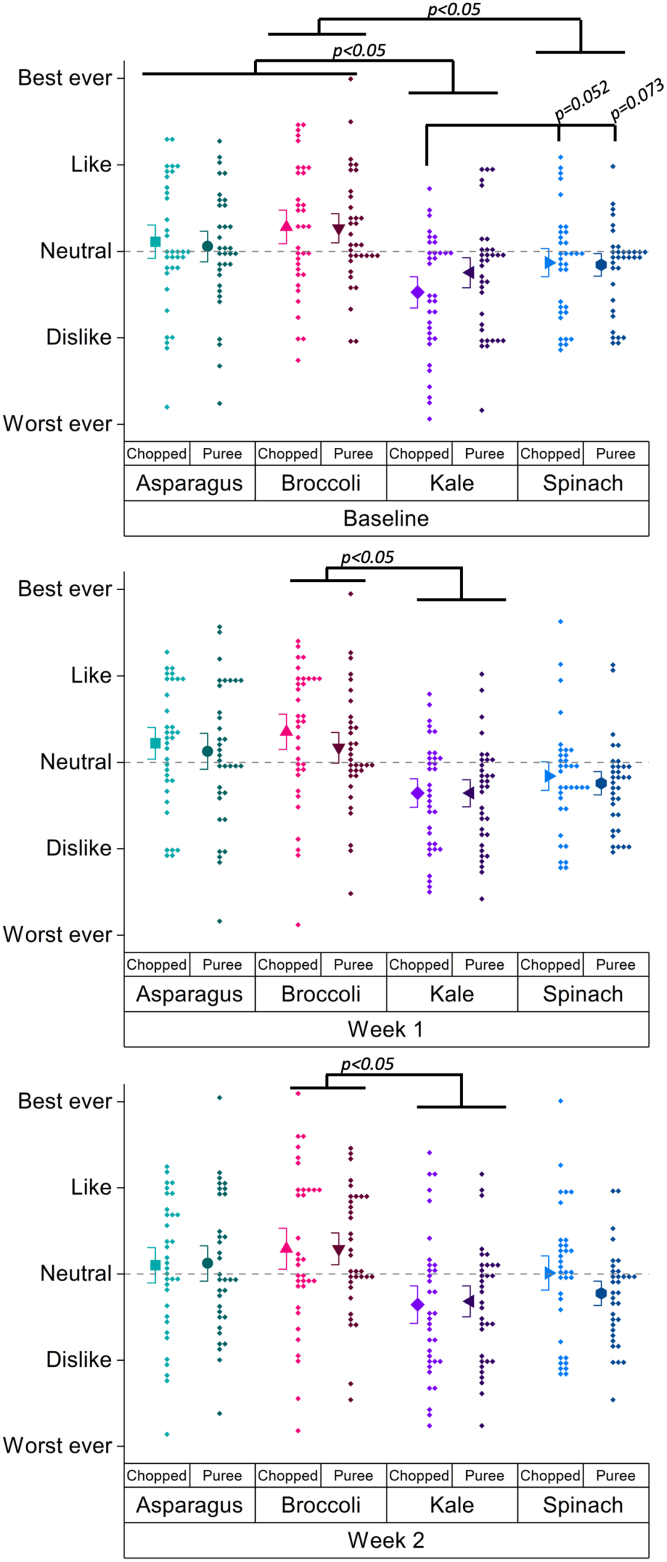
Liking ratings for vegetables at each visit, from secondary analysis model, with significant differences marked with p‐values. Large symbols indicate means and bars are SE. Small symbols are individual data points.

There were no significant between‐group differences for liking vegetables at any visit. However, at baseline, chopped kale was liked less (*p* < .05) than all other samples except pureed kale (*p* = .19), and differences were borderline significantly lower from chopped (*p* = .052) and maybe pureed spinach (*p* = .073). Also at baseline, pureed kale was liked less than chopped asparagus and both pureed and chopped broccoli. Both broccolis were liked more than spinach, but neither was different from the asparagus. No other differences among the asparaguses or the spinaches were observed. After week 1, both kales were still liked less than both asparaguses and both broccolis (*p* < .05). Kales and spinaches were no longer different after week 1. Figure 4 summarizes differences among vegetables at the different visits.

Table 2 also shows which least square means differ from 0, denoted by crosses. These analyses are included in the outputs from the linear mixed models procedure. As 0 on our scale is “neutral,” observing which values are statistically significantly greater or less than 0 can be informative. We note the patterns for kale, in particular, given the significant increases in liking over time for chopped kale in the vegetable group and decreases in liking over time for pureed kale in the control group. Looking at differences from 0, patterns mirror the time effects of the contrast terms in the linear mixed models. At baseline, both kales were rated significantly below neutral by the vegetable group, but the ratings were not significantly different from neutral by weeks 1 and 2. Also at baseline, both kales were not rated significantly different from neutral by the control group, but at weeks 1 and 2, this group rated both kales significantly lower than neutral.

## 4 DISCUSSION

The method described here, using a game to repeatedly expose people to the flavor of vegetables, may have potential to increase liking ratings of some vegetables, as seen in the effects on kale liking. However, the other vegetable tested in our game, broccoli, did not increase in liking. Thus, the effects are mixed. Indeed, the only other vegetable with any pattern showing a possible increase in liking was spinach, whose flavor was not included in the game. This indicates that while our game may increase liking for kale, the effect may not be as direct or predictable as we had intended.

The effect of exposure to kale flavor increasing kale liking is consistent with other reports demonstrating increased liking of vegetables after repeated exposure. The fact that our kale flavor was also paired with sugar could also have enhanced the effect, as shown in studies using more intentionally targeted conditioning approaches. In children, vegetables conditioned with glucose over six trials were more liked than those not paired with glucose when evaluating the unsweetened vegetables after conditioning (Havermans & Jansen, 2007). Another study in younger children tested acceptance of a novel vegetable puree exposed alone, paired with sugar, or paired with oil (Caton et al., 2013). All strategies significantly increased vegetable intake postintervention and during a 5‐month follow‐up period compared to preexposure. These patterns have also been observed in adult interventions. One test in college students involved repeated consumption of broccoli and cauliflower either unsweetened or dipped in a 20% sucrose solution, with evaluation of vegetable pleasantness, bitterness, and sweetness of the unsweetened vegetables pre‐ and postintervention (Capaldi & Privitera, 2008). After five exposures, pleasantness increased for vegetables paired with sucrose during exposure, but not for plain vegetables. In our work, we observed a similar effect for one of our target vegetables: liking increased for chopped kale for participants in the vegetable group who repeatedly consumed a sugar‐sweetened kale gummy during the game phase, but not for participants in the nonvegetable control group who were not exposed to the sweet kale gummy. Yet, this effect did not occur for broccoli, which was sweetened the same as kale. This inconsistency needs further investigation.

One potential reason for the different outcomes for chopped kale compared to chopped broccoli could be the difference in initial liking ratings. In the vegetable group, chopped kale ratings started below neutral and increased to neutral, while chopped broccoli's ratings were never different from neutral. Potentially, the repeated exposure method of increasing liking may work less when a vegetable is already neutral or liked. This has been documented before. For example, adult women were tested for the effect of weekly exposure to different types of spinach on liking ratings (Bingham et al., 2005). Overall, increases in liking for spinach were driven by spinach dislikers (ratings less than 3 on a 10‐pt hedonic scale). Subjects who initially rated spinach as neutral did not increase liking across that intervention either. In another example highlighting the importance of initial liking, elementary school‐aged children were provided alternating weekly exposures to fruits and vegetables over 8 weeks, and liking was analyzed over time. Liking ratings increased for all fruits and vegetables by the end of the intervention in children who were initial dislikers, whereas liking ratings were maintained in the children who initially liked the fruit and vegetables (Lakkakula et al., 2010). Another research group in Denmark tested repeated exposure to initially liked and disliked snack bars in 9‐ to 11‐year‐old children (Hausner et al., 2012). Exposure to an initially disliked snack bar increased to the level of the initially liked bar, while ratings for the initially liked bar did not change. The same research group later conducted a similar study using novel juices and demonstrated that initial dislikers of the juices were the only group to significantly increase their liking ratings after the repeated exposure protocol, although the liking ratings did not increase to the level of the initial likers (Hartvig et al., 2015). Thus, the initial magnitude of liking influences the success of repeated exposure interventions, with initially disliked foods having the greatest likelihood for increased acceptance postintervention. This could potentially explain why, in our study, we see increases for liking chopped kale but not chopped broccoli.

Overall, our method showed mixed effects for our primary outcome of vegetable liking, with kale showing improvement in liking after our vegetable flavor game but broccoli not showing improvement. Our secondary analyses give some insight into potential reasons why this discrepancy occurred, but in general, the method should be tested in a larger sample size with a broader array of vegetables both in the game and in the sensory trials.

## 5 LIMITATIONS

As this work was designed as a pilot study to test the gamification method, there are several limitations. First, our sample size was limited, although we were adequately powered to find the effect size, we observed for kale. Nevertheless, future experiments should validate these findings in a larger study.

While our study was powered to find effects, the small sample limits the generalizability of our outcomes. Our study results may only apply to younger adults (ages 18–25), mostly from a university environment and generally White or Asian in ethnic identity.

Additionally, several resource constraints may have introduced some variability. For example, vegetables are naturally occurring products that vary between seasons and lots. We mitigated this by using frozen vegetables for our sensory evaluation samples, buying in bulk, and making purees in large batches, but minor differences in flavor may have occurred across samples.

Another potential limitation is our method of selecting participants who qualified for the game. We selected low vegetable consumers who disliked at least one target and one nontarget vegetable sample, but it is possible that some individuals who qualified liked vegetables generally, but not our specific samples (served cold without any seasoning, which is not a particularly common way to consume vegetables). Additionally, we selected our “low vegetable consumers” using a food frequency questionnaire (Erinosho et al., 2015), which while validated, is still subject to high levels of bias and inaccurate reporting.

In general, the cognitive influence of gameplay (and ability to identify vegetables, or “win” at the game) on vegetable liking also warrants further investigation. Participants were trained through gameplay to identify the gummy flavors by name, which introduced awareness and information into the protocol that may influence flavor conditioning (Baeyens et al., 2001; Brunstrom, 2004). In the more identifiable chopped samples, participants could recognize the sample before tasting it, which adds cognitive factors to the assessment. Moreover, our study was not powered to assess the effects of game performance on outcomes. Potentially, if identification is a key part of improved liking, then individuals who improve more during the game might acquire more liking than individuals whose flavor identification abilities do not improve. These cognitive factors should be further explored as if this game were to be used in free‐living environments, people would be aware of the vegetables they are eating, and whether they overlap with vegetable flavors in the game.

## 6 CONCLUSIONS

In this work, we tested a “gamified” approach to improve vegetable liking. We repeatedly exposed subjects to vegetable flavor by challenging subjects to identify flavors in vegetable‐flavored gummies. We demonstrated that this repeated vegetable flavor exposure paired with sweetness may increase liking of chopped kale, but not chopped broccoli. Secondary analysis shows both kales were more disliked at the beginning of the game than broccoli by the group who played the vegetable game, suggesting that future tests of this vegetable flavor game should more tightly control for initial liking of the vegetables. This would aid in determining if this method might have broader use to increase liking of disliked vegetables, or if our effect was somehow specific to kale. Additional testing in larger, more diverse subject populations with a broader array of vegetable flavors would also aid in determining the broader utility of this method for simplifying vegetable flavor exposure, as a means to increase liking for actual vegetables.

## AUTHOR CONTRIBUTIONS

**Lissa A. Davis:** Conceptualization (equal); data curation (equal); formal analysis (equal); funding acquisition (supporting); investigation (equal); methodology (equal); project administration (equal); resources (equal); software (equal); validation (equal); visualization (equal); writing – original draft (equal); writing – review and editing (equal). **Elizabeth I. Kielb:** Conceptualization (supporting); formal analysis (supporting); investigation (supporting); methodology (supporting); visualization (supporting); writing – review and editing (supporting). **Kameron J. Moding:** Conceptualization (equal); data curation (equal); formal analysis (equal); funding acquisition (equal); investigation (equal); methodology (equal); project administration (equal); resources (equal); software (equal); supervision (equal); validation (equal); visualization (supporting); writing – original draft (supporting); writing – review and editing (equal). **Cordelia A. Running:** Conceptualization (lead); data curation (equal); formal analysis (equal); funding acquisition (equal); investigation (equal); methodology (lead); project administration (equal); resources (equal); software (equal); supervision (lead); validation (equal); visualization (equal); writing – original draft (supporting); writing – review and editing (equal).

## CONFLICT OF INTEREST STATEMENT

Authors Davis, Moding, and Running have a provisional patent application on the game design. Author Running occasionally consults for the food or biomedical industries, but no company or group had any participation or influence on this work. Author Davis currently works in the food industry, but all of this research and analysis was conducted prior to her work in industry. Her company has no influence or affiliation with this work.

## ETHICS STATEMENT

Ethical Review: The study was approved by the Purdue University Human Subjects Review Board (IRB‐2021‐1742).

Informed consent: All subjects provided written, informed consent.

## Supporting information


Data S1.

Data S2.

## Data Availability

Data are available at the study registration, https://osf.io/nb9d3.
