# Supplementary material for: A method to “gamify” exposure to vegetable flavor and its potential influence on liking
Source: Food Sci Nutr. 2024 Jun 21;12(9):6873–85. doi: 10.1002/fsn3.4272 (PMC11561783; doi:10.1002/fsn3.4272)
Supplement: Supplementary file 1 — Data S1. [file FSN3-12-6873-s001.pdf]

This code analyses data from our Veggie Flavor Game intervention

In [ ]:

```
#These packages are used in the analysis
import pandas as pd
import numpy as np
import saspy
sas = saspy.SASsession()
```

In [2]:

```
#This is our raw data in long form (each *rating* is it's own row, so participants have many rows each
gamevisits = pd.read_csv('./GameVisitsLong.csv')
#gamevisits.info()
```

In [3]:

```
# reading the data into the SAS package
vegsas = sas.df2sd(gamevisits, 'gamevisits')
```

In [4]:

```
%%SAS sas

proc sort data=gamevisits;
by samplename;
run;

title 'OSF planned analysis: Liking';
ods output diffs=diff1 tests3=tests1
SolutionF=sol1 lsmeans=means1 FitStatistics=fit1 lsmESTIMATEs =LikeEstim1;
proc mixed data= gamevisits;
*where participantid ne 1010 ;
*excluding this person does not change kale chopped outcome
(we thought they might have been an outlier, with almost 100 pts increase, but
 turns out they are not per the rule of more than 1.5 IQR above Q3);
class participantID gamevisit group (ref = 'Control') gender samplename;
by samplename;
model liking = gamevisit group gamevisit*group gender/ ddfm=kr solution; *fit is slightly better with
repeated/ subject = participantID type = cs;
lsmeans group/pdiff ADJDFE=ROW;
lsmeans gamevisit/pdiff ADJDFE=ROW;
lsmeans gamevisit*group/pdiff adjdfe=row;
lsmestimate gamevisit*group 'Control, GV3-GV1' 0 -1 0 0 0 1 ;
lsmestimate gamevisit*group 'Control, GV2-GV1' 0 -1 0 1 0 0 ;
lsmestimate gamevisit*group 'Veg, GV3-GV1' -1 0 0 0 1 0 ;
lsmestimate gamevisit*group 'Veg, GV2-GV1' -1 0 1 0 0 0 ;
lsmestimate gamevisit*group 'Veg - Control, GV1' 1 -1 0 0 0 0 ;
lsmestimate gamevisit*group 'Veg - Control, GV2' 0 0 1 -1 0 0 ;
lsmestimate gamevisit*group 'Veg - Control, GV3' 0 0 0 0 1 -1 ;
run;
```

Out[4]:

OSF planned analysis: Liking

The Mixed Procedure

SampleName=Asparagus chopped

Model Information	
**Data Set**	WORK.GAMEVISITS
**Dependent Variable**	Liking
**Covariance Structure**	Compound Symmetry
**Subject Effect**	ParticipantID
**Estimation Method**	REML
**Residual Variance Method**	Profile
**Fixed Effects SE Method**	Kenward-Roger

Model Information	
Degrees of Freedom Method	Kenward-Roger

Class Level Information		
Class	Levels	Values
ParticipantID	34	1001 1002 1005 1006 1009 1010 1014 1016 1019 1020 1024 1025 1027 1029 1030 1034 1035 1041 1045 1046 1048 1049 1050 1051 1056 1061 1063 1064 1065 1066 1068 1069 1074 1078
GameVisit	3	1 2 3
Group	2	Vegetable Control
Gender	2	Man Woman
SampleName	1	Asparagus chopped

Dimensions	
Covariance Parameters	2
Columns in X	14
Columns in Z	0
Subjects	34
Max Obs per Subject	3

Number of Observations	
Number of Observations Read	102
Number of Observations Used	102
Number of Observations Not Used	0

Iteration History			
Iteration	Evaluations	-2 Res Log Like	Criterion
0	1	978.57450049	
1	1	931.36776407	0.00000000

Convergence criteria met.

Covariance Parameter Estimates		
Cov Parm	Subject	Estimate
CS	ParticipantID	992.35
Residual		439.81

Fit Statistics	
-2 Res Log Likelihood	931.4
AIC (Smaller is Better)	935.4
AICC (Smaller is Better)	935.5
BIC (Smaller is Better)	938.4

Null Model Likelihood Ratio Test		
DF	Chi-Square	Pr > ChiSq
1	47.21	<.0001

Solution for Fixed Effects								
Effect	Group	Gender	GameVisit	Estimate	Standard Error	DF	t Value	Pr > |t|
Intercept				14.4833	10.6233	43.4	1.36	0.1798
GameVisit			1	4.5625	7.4146	64	0.62	0.5405
GameVisit			2	-0.9375	7.4146	64	-0.13	0.8998
GameVisit			3	0	.	.	.	.
Group	Vegetable			-15.0302	13.1510	47	-1.14	0.2589
Group	Control			0	.	.	.	.
GameVisit*Group	Vegetable		1	-7.6181	10.1903	64	-0.75	0.4575
GameVisit*Group	Control		1	0	.	.	.	.
GameVisit*Group	Vegetable		2	12.9375	10.1903	64	1.27	0.2088
GameVisit*Group	Control		2	0	.	.	.	.
GameVisit*Group	Vegetable		3	0	.	.	.	.
GameVisit*Group	Control		3	0	.	.	.	.
Gender		Man		-4.7888	12.8841	31	-0.37	0.7127
Gender		Woman		0	.	.	.	.

Type 3 Tests of Fixed Effects				
Effect	Num DF	Den DF	F Value	Pr > F
GameVisit	2	64	0.69	0.5037
Group	1	31	1.27	0.2683
GameVisit*Group	2	64	2.08	0.1333
Gender	1	31	0.14	0.7127

Least Squares Means Estimate						
Effect	Label	Estimate	Standard Error	DF	t Value	Pr > |t|
GameVisit*Group	Control, GV3-GV1	-4.5625	7.4146	64	-0.62	0.5405

Least Squares Means Estimate						
Effect	Label	Estimate	Standard Error	DF	t Value	Pr > |t|
GameVisit*Group	Control, GV2-GV1	-5.5000	7.4146	64	-0.74	0.4609

Least Squares Means Estimate						
Effect	Label	Estimate	Standard Error	DF	t Value	Pr > |t|
GameVisit*Group	Veg, GV3-GV1	3.0556	6.9905	64	0.44	0.6635

Least Squares Means Estimate						
Effect	Label	Estimate	Standard Error	DF	t Value	Pr > |t|
GameVisit*Group	Veg, GV2-GV1	15.0556	6.9905	64	2.15	0.0350

Least Squares Means Estimate						
Effect	Label	Estimate	Standard Error	DF	t Value	Pr > |t|
GameVisit*Group	Veg - Control, GV1	-22.6483	13.1510	47.03	-1.72	0.0916

Least Squares Means Estimate						
Effect	Label	Estimate	Standard Error	DF	t Value	Pr > |t|
GameVisit*Group	Veg - Control, GV2	-2.0927	13.1510	47.03	-0.16	0.8742

Least Squares Means Estimate						
Effect	Label	Estimate	Standard Error	DF	t Value	Pr > |t|
GameVisit*Group	Veg - Control, GV3	-15.0302	13.1510	47.03	-1.14	0.2589

Least Squares Means							
Effect	Group	GameVisit	Estimate	Standard Error	DF	t Value	Pr > |t|
Group	Vegetable		0.04015	8.7226	31	0.00	0.9964
Group	Control		13.2972	8.5894	31	1.55	0.1317
GameVisit		1	5.3273	7.0001	44.7	0.76	0.4506
GameVisit		2	10.1050	7.0001	44.7	1.44	0.1558
GameVisit		3	4.5738	7.0001	44.7	0.65	0.5168
GameVisit*Group	Vegetable	1	-5.9969	9.6111	44.7	-0.62	0.5358
GameVisit*Group	Control	1	16.6514	9.5971	46.9	1.74	0.0893
GameVisit*Group	Vegetable	2	9.0587	9.6111	44.7	0.94	0.3510
GameVisit*Group	Control	2	11.1514	9.5971	46.9	1.16	0.2511
GameVisit*Group	Vegetable	3	-2.9413	9.6111	44.7	-0.31	0.7610
GameVisit*Group	Control	3	12.0889	9.5971	46.9	1.26	0.2140

Differences of Least Squares Means									
Effect	Group	GameVisit	_Group	_GameVisit	Estimate	Standard Error	DF	t Value	Pr > |t|
Group	Vegetable		Control		-13.2571	11.7615	31	-1.13	0.2683
GameVisit		1		2	-4.7778	5.0952	64	-0.94	0.3519
GameVisit		1		3	0.7535	5.0952	64	0.15	0.8829
GameVisit		2		3	5.5312	5.0952	64	1.09	0.2817
GameVisit*Group	Vegetable	1	Control	1	-22.6483	13.1510	47	-1.72	0.0916
GameVisit*Group	Vegetable	1	Vegetable	2	-15.0556	6.9905	64	-2.15	0.0350
GameVisit*Group	Vegetable	1	Control	2	-17.1483	13.1510	47	-1.30	0.1986
GameVisit*Group	Vegetable	1	Vegetable	3	-3.0556	6.9905	64	-0.44	0.6635
GameVisit*Group	Vegetable	1	Control	3	-18.0858	13.1510	47	-1.38	0.1756
GameVisit*Group	Control	1	Vegetable	2	7.5927	13.1510	47	0.58	0.5665
GameVisit*Group	Control	1	Control	2	5.5000	7.4146	64	0.74	0.4609
GameVisit*Group	Control	1	Vegetable	3	19.5927	13.1510	47	1.49	0.1429
GameVisit*Group	Control	1	Control	3	4.5625	7.4146	64	0.62	0.5405
GameVisit*Group	Vegetable	2	Control	2	-2.0927	13.1510	47	-0.16	0.8742
GameVisit*Group	Vegetable	2	Vegetable	3	12.0000	6.9905	64	1.72	0.0909
GameVisit*Group	Vegetable	2	Control	3	-3.0302	13.1510	47	-0.23	0.8188
GameVisit*Group	Control	2	Vegetable	3	14.0927	13.1510	47	1.07	0.2894
GameVisit*Group	Control	2	Control	3	-0.9375	7.4146	64	-0.13	0.8998
GameVisit*Group	Vegetable	3	Control	3	-15.0302	13.1510	47	-1.14	0.2589

OSF planned analysis: Liking

The Mixed Procedure

SampleName=Asparagus puree

Model Information	
Data Set	WORK.GAMEVISITS
Dependent Variable	Liking
Covariance Structure	Compound Symmetry
Subject Effect	ParticipantID
Estimation Method	REML
Residual Variance Method	Profile
Fixed Effects SE Method	Kenward-Roger
Degrees of Freedom Method	Kenward-Roger

Class Level Information		
Class	Levels	Values
ParticipantID	34	1001 1002 1005 1006 1009 1010 1014 1016 1019 1020 1024 1025 1027 1029 1030 1034 1035 1041 1045 1046 1048 1049 1050 1051 1056 1061 1063 1064 1065 1066 1068 1069 1074 1078
GameVisit	3	1 2 3
Group	2	Vegetable Control
Gender	2	Man Woman
SampleName	1	Asparagus puree

Dimensions	
Covariance Parameters	2
Columns in X	14
Columns in Z	0
Subjects	34
Max Obs per Subject	3

Number of Observations	
Number of Observations Read	102
Number of Observations Used	102
Number of Observations Not Used	0

Iteration History			
Iteration	Evaluations	-2 Res Log Like	Criterion
0	1	982.39308372	
1	1	941.06225848	0.00000000

Convergence criteria met.

Covariance Parameter Estimates		
Cov Parm	Subject	Estimate
CS	ParticipantID	980.49

Covariance Parameter Estimates		
Cov Parm	Subject	Estimate
Residual		509.29

Fit Statistics	
-2 Res Log Likelihood	941.1
AIC (Smaller is Better)	945.1
AICC (Smaller is Better)	945.2
BIC (Smaller is Better)	948.1

Null Model Likelihood Ratio Test		
DF	Chi-Square	Pr > ChiSq
1	41.33	<.0001

Solution for Fixed Effects								
Effect	Group	Gender	GameVisit	Estimate	Standard Error	DF	t Value	Pr > |t|
Intercept				11.0903	10.8022	45.2	1.03	0.3100
GameVisit			1	0.9375	7.9788	64	0.12	0.9068
GameVisit			2	6.0000	7.9788	64	0.75	0.4548
GameVisit			3	0	.	.	.	.
Group	Vegetable			-8.4998	13.4086	49.4	-0.63	0.5291
Group	Control			0	.	.	.	.
GameVisit*Group	Vegetable		1	-8.1597	10.9659	64	-0.74	0.4595
GameVisit*Group	Control		1	0	.	.	.	.
GameVisit*Group	Vegetable		2	-11.0556	10.9659	64	-1.01	0.3172
GameVisit*Group	Control		2	0	.	.	.	.
GameVisit*Group	Vegetable		3	0	.	.	.	.
GameVisit*Group	Control		3	0	.	.	.	.
Gender		Man		-1.4076	12.9479	31	-0.11	0.9141
Gender		Woman		0	.	.	.	.

Type 3 Tests of Fixed Effects				
Effect	Num DF	Den DF	F Value	Pr > F
GameVisit	2	64	0.26	0.7743
Group	1	31	1.59	0.2167
GameVisit*Group	2	64	0.55	0.5816
Gender	1	31	0.01	0.9141

Least Squares Means Estimate						
Effect	Label	Estimate	Standard Error	DF	t Value	Pr > |t|
GameVisit*Group	Control, GV3-GV1	-0.9375	7.9788	64	-0.12	0.9068

Least Squares Means Estimate						
Effect	Label	Estimate	Standard Error	DF	t Value	Pr > |t|

Least Squares Means Estimate						
Effect	Label	Estimate	Standard Error	DF	t Value	Pr > |t|
GameVisit*Group	Control, GV2-GV1	5.0625	7.9788	64	0.63	0.5280

Least Squares Means Estimate						
Effect	Label	Estimate	Standard Error	DF	t Value	Pr > |t|
GameVisit*Group	Veg, GV3-GV1	7.2222	7.5225	64	0.96	0.3406

Least Squares Means Estimate						
Effect	Label	Estimate	Standard Error	DF	t Value	Pr > |t|
GameVisit*Group	Veg, GV2-GV1	2.1667	7.5225	64	0.29	0.7743

Least Squares Means Estimate						
Effect	Label	Estimate	Standard Error	DF	t Value	Pr > |t|
GameVisit*Group	Veg - Control, GV1	-16.6595	13.4086	49.37	-1.24	0.2199

Least Squares Means Estimate						
Effect	Label	Estimate	Standard Error	DF	t Value	Pr > |t|
GameVisit*Group	Veg - Control, GV2	-19.5553	13.4086	49.37	-1.46	0.1511

Least Squares Means Estimate						
Effect	Label	Estimate	Standard Error	DF	t Value	Pr > |t|
GameVisit*Group	Veg - Control, GV3	-8.4998	13.4086	49.37	-0.63	0.5291

Least Squares Means							
Effect	Group	GameVisit	Estimate	Standard Error	DF	t Value	Pr > |t|
Group	Vegetable		-2.2058	8.7658	31	-0.25	0.8030
Group	Control		12.6991	8.6320	31	1.47	0.1513
GameVisit		1	2.9943	7.1252	46.8	0.42	0.6762
GameVisit		2	6.6089	7.1252	46.8	0.93	0.3584
GameVisit		3	6.1367	7.1252	46.8	0.86	0.3935
GameVisit*Group	Vegetable	1	-5.3354	9.7827	46.7	-0.55	0.5881
GameVisit*Group	Control	1	11.3241	9.7842	49.2	1.16	0.2527
GameVisit*Group	Vegetable	2	-3.1688	9.7827	46.7	-0.32	0.7474
GameVisit*Group	Control	2	16.3866	9.7842	49.2	1.67	0.1003
GameVisit*Group	Vegetable	3	1.8868	9.7827	46.7	0.19	0.8479
GameVisit*Group	Control	3	10.3866	9.7842	49.2	1.06	0.2936

Differences of Least Squares Means									
Effect	Group	GameVisit	_Group	_GameVisit	Estimate	Standard Error	DF	t Value	Pr > |t|
Group	Vegetable		Control		-14.9049	11.8198	31	-1.26	0.2167
GameVisit		1		2	-3.6146	5.4829	64	-0.66	0.5121
GameVisit		1		3	-3.1424	5.4829	64	-0.57	0.5686
GameVisit		2		3	0.4722	5.4829	64	0.09	0.9316
GameVisit*Group	Vegetable	1	Control	1	-16.6595	13.4086	49.4	-1.24	0.2199

Differences of Least Squares Means									
Effect	Group	GameVisit	_Group	_GameVisit	Estimate	Standard Error	DF	t Value	Pr > |t|
GameVisit*Group	Vegetable	1	Vegetable	2	-2.1667	7.5225	64	-0.29	0.7743
GameVisit*Group	Vegetable	1	Control	2	-21.7220	13.4086	49.4	-1.62	0.1116
GameVisit*Group	Vegetable	1	Vegetable	3	-7.2222	7.5225	64	-0.96	0.3406
GameVisit*Group	Vegetable	1	Control	3	-15.7220	13.4086	49.4	-1.17	0.2466
GameVisit*Group	Control	1	Vegetable	2	14.4928	13.4086	49.4	1.08	0.2850
GameVisit*Group	Control	1	Control	2	-5.0625	7.9788	64	-0.63	0.5280
GameVisit*Group	Control	1	Vegetable	3	9.4373	13.4086	49.4	0.70	0.4849
GameVisit*Group	Control	1	Control	3	0.9375	7.9788	64	0.12	0.9068
GameVisit*Group	Vegetable	2	Control	2	-19.5553	13.4086	49.4	-1.46	0.1511
GameVisit*Group	Vegetable	2	Vegetable	3	-5.0556	7.5225	64	-0.67	0.5040
GameVisit*Group	Vegetable	2	Control	3	-13.5553	13.4086	49.4	-1.01	0.3170
GameVisit*Group	Control	2	Vegetable	3	14.4998	13.4086	49.4	1.08	0.2848
GameVisit*Group	Control	2	Control	3	6.0000	7.9788	64	0.75	0.4548
GameVisit*Group	Vegetable	3	Control	3	-8.4998	13.4086	49.4	-0.63	0.5291

OSF planned analysis: Liking

The Mixed Procedure

SampleName=Beef puree

Model Information	
Data Set	WORK.GAMEVISITS
Dependent Variable	Liking
Covariance Structure	Compound Symmetry
Subject Effect	ParticipantID
Estimation Method	REML
Residual Variance Method	Profile
Fixed Effects SE Method	Kenward-Roger
Degrees of Freedom Method	Kenward-Roger

Class Level Information		
Class	Levels	Values
ParticipantID	34	1001 1002 1005 1006 1009 1010 1014 1016 1019 1020 1024 1025 1027 1029 1030 1034 1035 1041 1045 1046 1048 1049 1050 1051 1056 1061 1063 1064 1065 1066 1068 1069 1074 1078
GameVisit	3	1 2 3
Group	2	Vegetable Control
Gender	2	Man Woman
SampleName	1	Beef puree

Dimensions	
Covariance Parameters	2
Columns in X	14
Columns in Z	0

Dimensions	
Subjects	34
Max Obs per Subject	3

Number of Observations	
Number of Observations Read	102
Number of Observations Used	102
Number of Observations Not Used	0

Iteration History			
Iteration	Evaluations	-2 Res Log Like	Criterion
0	1	1053.15833976	
1	1	944.82864518	0.00000000

Convergence criteria met.

Covariance Parameter Estimates		
Cov Parm	Subject	Estimate
CS	ParticipantID	2809.75
Residual		343.75

Fit Statistics	
-2 Res Log Likelihood	944.8
AIC (Smaller is Better)	948.8
AICC (Smaller is Better)	949.0
BIC (Smaller is Better)	951.9

Null Model Likelihood Ratio Test		
DF	Chi-Square	Pr > ChiSq
1	108.33	<.0001

Solution for Fixed Effects								
Effect	Group	Gender	GameVisit	Estimate	Standard Error	DF	t Value	Pr > |t|
Intercept				-10.2143	16.0322	34.7	-0.64	0.5282
GameVisit			1	-7.2500	6.5551	64	-1.11	0.2729
GameVisit			2	-4.5625	6.5551	64	-0.70	0.4889
GameVisit			3	0	.	.	.	.
Group	Vegetable			-11.7275	19.5509	35.8	-0.60	0.5524
Group	Control			0	.	.	.	.
GameVisit*Group	Vegetable		1	-0.3056	9.0091	64	-0.03	0.9730
GameVisit*Group	Control		1	0	.	.	.	.
GameVisit*Group	Vegetable		2	-7.6597	9.0091	64	-0.85	0.3984
GameVisit*Group	Control		2	0	.	.	.	.
GameVisit*Group	Vegetable		3	0	.	.	.	.
GameVisit*Group	Control		3	0	.	.	.	.

Solution for Fixed Effects								
Effect	Group	Gender	GameVisit	Estimate	Standard Error	DF	t Value	Pr > |t|
Gender		Man		25.7382	20.6451	31	1.25	0.2218
Gender		Woman		0	.	.	.	.

Type 3 Tests of Fixed Effects				
Effect	Num DF	Den DF	F Value	Pr > F
GameVisit	2	64	2.07	0.1341
Group	1	31	0.58	0.4511
GameVisit*Group	2	64	0.46	0.6312
Gender	1	31	1.55	0.2218

Least Squares Means Estimate						
Effect	Label	Estimate	Standard Error	DF	t Value	Pr > |t|
GameVisit*Group	Control, GV3-GV1	7.2500	6.5551	64	1.11	0.2729

Least Squares Means Estimate						
Effect	Label	Estimate	Standard Error	DF	t Value	Pr > |t|
GameVisit*Group	Control, GV2-GV1	2.6875	6.5551	64	0.41	0.6832

Least Squares Means Estimate						
Effect	Label	Estimate	Standard Error	DF	t Value	Pr > |t|
GameVisit*Group	Veg, GV3-GV1	7.5556	6.1802	64	1.22	0.2260

Least Squares Means Estimate						
Effect	Label	Estimate	Standard Error	DF	t Value	Pr > |t|
GameVisit*Group	Veg, GV2-GV1	-4.6667	6.1802	64	-0.76	0.4530

Least Squares Means Estimate						
Effect	Label	Estimate	Standard Error	DF	t Value	Pr > |t|
GameVisit*Group	Veg - Control, GV1	-12.0331	19.5509	35.8	-0.62	0.5421

Least Squares Means Estimate						
Effect	Label	Estimate	Standard Error	DF	t Value	Pr > |t|
GameVisit*Group	Veg - Control, GV2	-19.3872	19.5509	35.8	-0.99	0.3280

Least Squares Means Estimate						
Effect	Label	Estimate	Standard Error	DF	t Value	Pr > |t|
GameVisit*Group	Veg - Control, GV3	-11.7275	19.5509	35.8	-0.60	0.5524

Least Squares Means							
Effect	Group	GameVisit	Estimate	Standard Error	DF	t Value	Pr > |t|
Group	Vegetable		-15.6653	13.9768	31	-1.12	0.2710
Group	Control		-1.2827	13.7634	31	-0.09	0.9263
GameVisit		1	-10.6118	10.5052	35.1	-1.01	0.3193

Least Squares Means							
Effect	Group	GameVisit	Estimate	Standard Error	DF	t Value	Pr > |t|
GameVisit		2	-11.6013	10.5052	35.1	-1.10	0.2770
GameVisit		3	-3.2090	10.5052	35.1	-0.31	0.7618
GameVisit*Group	Vegetable	1	-16.6283	14.4250	35.1	-1.15	0.2568
GameVisit*Group	Control	1	-4.5952	14.2742	35.8	-0.32	0.7494
GameVisit*Group	Vegetable	2	-21.2949	14.4250	35.1	-1.48	0.1488
GameVisit*Group	Control	2	-1.9077	14.2742	35.8	-0.13	0.8944
GameVisit*Group	Vegetable	3	-9.0727	14.4250	35.1	-0.63	0.5334
GameVisit*Group	Control	3	2.6548	14.2742	35.8	0.19	0.8535

Differences of Least Squares Means									
Effect	Group	GameVisit	_Group	_GameVisit	Estimate	Standard Error	DF	t Value	Pr > |t|
Group	Vegetable		Control		-14.3826	18.8463	31	-0.76	0.4511
GameVisit		1		2	0.9896	4.5045	64	0.22	0.8268
GameVisit		1		3	-7.4028	4.5045	64	-1.64	0.1052
GameVisit		2		3	-8.3924	4.5045	64	-1.86	0.0670
GameVisit*Group	Vegetable	1	Control	1	-12.0331	19.5509	35.8	-0.62	0.5421
GameVisit*Group	Vegetable	1	Vegetable	2	4.6667	6.1802	64	0.76	0.4530
GameVisit*Group	Vegetable	1	Control	2	-14.7206	19.5509	35.8	-0.75	0.4564
GameVisit*Group	Vegetable	1	Vegetable	3	-7.5556	6.1802	64	-1.22	0.2260
GameVisit*Group	Vegetable	1	Control	3	-19.2831	19.5509	35.8	-0.99	0.3306
GameVisit*Group	Control	1	Vegetable	2	16.6997	19.5509	35.8	0.85	0.3987
GameVisit*Group	Control	1	Control	2	-2.6875	6.5551	64	-0.41	0.6832
GameVisit*Group	Control	1	Vegetable	3	4.4775	19.5509	35.8	0.23	0.8202
GameVisit*Group	Control	1	Control	3	-7.2500	6.5551	64	-1.11	0.2729
GameVisit*Group	Vegetable	2	Control	2	-19.3872	19.5509	35.8	-0.99	0.3280
GameVisit*Group	Vegetable	2	Vegetable	3	-12.2222	6.1802	64	-1.98	0.0523
GameVisit*Group	Vegetable	2	Control	3	-23.9497	19.5509	35.8	-1.22	0.2286
GameVisit*Group	Control	2	Vegetable	3	7.1650	19.5509	35.8	0.37	0.7162
GameVisit*Group	Control	2	Control	3	-4.5625	6.5551	64	-0.70	0.4889
GameVisit*Group	Vegetable	3	Control	3	-11.7275	19.5509	35.8	-0.60	0.5524

OSF planned analysis: Liking

The Mixed Procedure

SampleName=Black bean puree

Model Information	
Data Set	WORK.GAMEVISITS
Dependent Variable	Liking
Covariance Structure	Compound Symmetry
Subject Effect	ParticipantID

Model Information	
Estimation Method	REML
Residual Variance Method	Profile
Fixed Effects SE Method	Kenward-Roger
Degrees of Freedom Method	Kenward-Roger

Class Level Information		
Class	Levels	Values
ParticipantID	34	1001 1002 1005 1006 1009 1010 1014 1016 1019 1020 1024 1025 1027 1029 1030 1034 1035 1041 1045 1046 1048 1049 1050 1051 1056 1061 1063 1064 1065 1066 1068 1069 1074 1078
GameVisit	3	1 2 3
Group	2	Vegetable Control
Gender	2	Man Woman
SampleName	1	Black bean puree

Dimensions	
Covariance Parameters	2
Columns in X	14
Columns in Z	0
Subjects	34
Max Obs per Subject	3

Number of Observations	
Number of Observations Read	102
Number of Observations Used	102
Number of Observations Not Used	0

Iteration History			
Iteration	Evaluations	-2 Res Log Like	Criterion
0	1	1011.88404648	
1	1	977.74309291	0.00000000

Convergence criteria met.

Covariance Parameter Estimates		
Cov Parm	Subject	Estimate
CS	ParticipantID	1235.67
Residual		794.28

Fit Statistics	
-2 Res Log Likelihood	977.7
AIC (Smaller is Better)	981.7
AICC (Smaller is Better)	981.9
BIC (Smaller is Better)	984.8

Null Model Likelihood Ratio Test		
DF	Chi-Square	Pr > ChiSq

Null Model Likelihood Ratio Test		
DF	Chi-Square	Pr > ChiSq
1	34.14	<.0001

Solution for Fixed Effects								
Effect	Group	Gender	GameVisit	Estimate	Standard Error	DF	t Value	Pr > |t|
Intercept				6.8287	12.5549	48	0.54	0.5890
GameVisit			1	-8.6875	9.9642	64	-0.87	0.3865
GameVisit			2	2.2500	9.9642	64	0.23	0.8221
GameVisit			3	0	.	.	.	.
Group	Vegetable			-12.6339	15.6445	52.9	-0.81	0.4230
Group	Control			0	.	.	.	.
GameVisit*Group	Vegetable		1	2.0208	13.6944	64	0.15	0.8831
GameVisit*Group	Control		1	0	.	.	.	.
GameVisit*Group	Vegetable		2	-4.9167	13.6944	64	-0.36	0.7208
GameVisit*Group	Control		2	0	.	.	.	.
GameVisit*Group	Vegetable		3	0	.	.	.	.
GameVisit*Group	Control		3	0	.	.	.	.
Gender		Man		4.1235	14.7880	31	0.28	0.7822
Gender		Woman		0	.	.	.	.

Type 3 Tests of Fixed Effects				
Effect	Num DF	Den DF	F Value	Pr > F
GameVisit	2	64	0.82	0.4468
Group	1	31	1.01	0.3216
GameVisit*Group	2	64	0.14	0.8733
Gender	1	31	0.08	0.7822

Least Squares Means Estimate						
Effect	Label	Estimate	Standard Error	DF	t Value	Pr > |t|
GameVisit*Group	Control, GV3-GV1	8.6875	9.9642	64	0.87	0.3865

Least Squares Means Estimate						
Effect	Label	Estimate	Standard Error	DF	t Value	Pr > |t|
GameVisit*Group	Control, GV2-GV1	10.9375	9.9642	64	1.10	0.2765

Least Squares Means Estimate						
Effect	Label	Estimate	Standard Error	DF	t Value	Pr > |t|
GameVisit*Group	Veg, GV3-GV1	6.6667	9.3943	64	0.71	0.4805

Least Squares Means Estimate						
Effect	Label	Estimate	Standard Error	DF	t Value	Pr > |t|
GameVisit*Group	Veg, GV2-GV1	4.0000	9.3943	64	0.43	0.6717

Least Squares Means Estimate						
Effect	Label	Estimate	Standard Error	DF	t Value	Pr > |t|
GameVisit*Group	Veg - Control, GV1	-10.6131	15.6445	52.9	-0.68	0.5005

Least Squares Means Estimate						
Effect	Label	Estimate	Standard Error	DF	t Value	Pr > |t|
GameVisit*Group	Veg - Control, GV2	-17.5506	15.6445	52.9	-1.12	0.2670

Least Squares Means Estimate						
Effect	Label	Estimate	Standard Error	DF	t Value	Pr > |t|
GameVisit*Group	Veg - Control, GV3	-12.6339	15.6445	52.9	-0.81	0.4230

Least Squares Means							
Effect	Group	GameVisit	Estimate	Standard Error	DF	t Value	Pr > |t|
Group	Vegetable		-6.8546	10.0115	31	-0.68	0.4986
Group	Control		6.7446	9.8587	31	0.68	0.4990
GameVisit		1	-5.1036	8.2934	49.8	-0.62	0.5411
GameVisit		2	2.3651	8.2934	49.8	0.29	0.7767
GameVisit		3	2.5735	8.2934	49.8	0.31	0.7576
GameVisit*Group	Vegetable	1	-10.4101	11.3863	49.8	-0.91	0.3650
GameVisit*Group	Control	1	0.2029	11.4144	52.7	0.02	0.9859
GameVisit*Group	Vegetable	2	-6.4101	11.3863	49.8	-0.56	0.5760
GameVisit*Group	Control	2	11.1404	11.4144	52.7	0.98	0.3335
GameVisit*Group	Vegetable	3	-3.7435	11.3863	49.8	-0.33	0.7437
GameVisit*Group	Control	3	8.8904	11.4144	52.7	0.78	0.4395

Differences of Least Squares Means									
Effect	Group	GameVisit	_Group	_GameVisit	Estimate	Standard Error	DF	t Value	Pr > |t|
Group	Vegetable		Control		-13.5992	13.4996	31	-1.01	0.3216
GameVisit		1		2	-7.4688	6.8472	64	-1.09	0.2795
GameVisit		1		3	-7.6771	6.8472	64	-1.12	0.2664
GameVisit		2		3	-0.2083	6.8472	64	-0.03	0.9758
GameVisit*Group	Vegetable	1	Control	1	-10.6131	15.6445	52.9	-0.68	0.5005
GameVisit*Group	Vegetable	1	Vegetable	2	-4.0000	9.3943	64	-0.43	0.6717
GameVisit*Group	Vegetable	1	Control	2	-21.5506	15.6445	52.9	-1.38	0.1742
GameVisit*Group	Vegetable	1	Vegetable	3	-6.6667	9.3943	64	-0.71	0.4805
GameVisit*Group	Vegetable	1	Control	3	-19.3006	15.6445	52.9	-1.23	0.2228
GameVisit*Group	Control	1	Vegetable	2	6.6131	15.6445	52.9	0.42	0.6742
GameVisit*Group	Control	1	Control	2	-10.9375	9.9642	64	-1.10	0.2765
GameVisit*Group	Control	1	Vegetable	3	3.9464	15.6445	52.9	0.25	0.8018
GameVisit*Group	Control	1	Control	3	-8.6875	9.9642	64	-0.87	0.3865
GameVisit*Group	Vegetable	2	Control	2	-17.5506	15.6445	52.9	-1.12	0.2670
GameVisit*Group	Vegetable	2	Vegetable	3	-2.6667	9.3943	64	-0.28	0.7774
GameVisit*Group	Vegetable	2	Control	3	-15.3006	15.6445	52.9	-0.98	0.3325

Differences of Least Squares Means									
Effect	Group	GameVisit	_Group	_GameVisit	Estimate	Standard Error	DF	t Value	Pr > |t|
GameVisit*Group	Control	2	Vegetable	3	14.8839	15.6445	52.9	0.95	0.3457
GameVisit*Group	Control	2	Control	3	2.2500	9.9642	64	0.23	0.8221
GameVisit*Group	Vegetable	3	Control	3	-12.6339	15.6445	52.9	-0.81	0.4230

OSF planned analysis: Liking

The Mixed Procedure

SampleName=Broccoli chopped

Model Information	
Data Set	WORK.GAMEVISITS
Dependent Variable	Liking
Covariance Structure	Compound Symmetry
Subject Effect	ParticipantID
Estimation Method	REML
Residual Variance Method	Profile
Fixed Effects SE Method	Kenward-Roger
Degrees of Freedom Method	Kenward-Roger

Class Level Information		
Class	Levels	Values
ParticipantID	34	1001 1002 1005 1006 1009 1010 1014 1016 1019 1020 1024 1025 1027 1029 1030 1034 1035 1041 1045 1046 1048 1049 1050 1051 1056 1061 1063 1064 1065 1066 1068 1069 1074 1078
GameVisit	3	1 2 3
Group	2	Vegetable Control
Gender	2	Man Woman
SampleName	1	Broccoli chopped

Dimensions	
Covariance Parameters	2
Columns in X	14
Columns in Z	0
Subjects	34
Max Obs per Subject	3

Number of Observations	
Number of Observations Read	102
Number of Observations Used	102
Number of Observations Not Used	0

Iteration History			
Iteration	Evaluations	-2 Res Log Like	Criterion
0	1	996.50058565	

Iteration History			
Iteration	Evaluations	-2 Res Log Like	Criterion
1	1	949.75561415	0.00000000

Convergence criteria met.

Covariance Parameter Estimates		
Cov Parm	Subject	Estimate
CS	ParticipantID	1193.92
Residual		535.56

Fit Statistics	
-2 Res Log Likelihood	949.8
AIC (Smaller is Better)	953.8
AICC (Smaller is Better)	953.9
BIC (Smaller is Better)	956.8

Null Model Likelihood Ratio Test		
DF	Chi-Square	Pr > ChiSq
1	46.74	<.0001

Solution for Fixed Effects								
Effect	Group	Gender	GameVisit	Estimate	Standard Error	DF	t Value	Pr > |t|
Intercept				25.1655	11.6714	43.5	2.16	0.0366
GameVisit			1	-3.3125	8.1820	64	-0.40	0.6869
GameVisit			2	4.9375	8.1820	64	0.60	0.5483
GameVisit			3	0	.	.	.	.
Group	Vegetable			-9.9193	14.4514	47.2	-0.69	0.4958
Group	Control			0	.	.	.	.
GameVisit*Group	Vegetable		1	5.0347	11.2451	64	0.45	0.6559
GameVisit*Group	Control		1	0	.	.	.	.
GameVisit*Group	Vegetable		2	-3.7708	11.2451	64	-0.34	0.7385
GameVisit*Group	Control		2	0	.	.	.	.
GameVisit*Group	Vegetable		3	0	.	.	.	.
GameVisit*Group	Control		3	0	.	.	.	.
Gender		Man		-17.6080	14.1432	31	-1.24	0.2225
Gender		Woman		0	.	.	.	.

Type 3 Tests of Fixed Effects				
Effect	Num DF	Den DF	F Value	Pr > F
GameVisit	2	64	0.26	0.7711
Group	1	31	0.54	0.4675
GameVisit*Group	2	64	0.31	0.7355
Gender	1	31	1.55	0.2225

Least Squares Means Estimate						
Effect	Label	Estimate	Standard Error	DF	t Value	Pr > |t|
GameVisit*Group	Control, GV3-GV1	3.3125	8.1820	64	0.40	0.6869

Least Squares Means Estimate						
Effect	Label	Estimate	Standard Error	DF	t Value	Pr > |t|
GameVisit*Group	Control, GV2-GV1	8.2500	8.1820	64	1.01	0.3171

Least Squares Means Estimate						
Effect	Label	Estimate	Standard Error	DF	t Value	Pr > |t|
GameVisit*Group	Veg, GV3-GV1	-1.7222	7.7141	64	-0.22	0.8240

Least Squares Means Estimate						
Effect	Label	Estimate	Standard Error	DF	t Value	Pr > |t|
GameVisit*Group	Veg, GV2-GV1	-0.5556	7.7141	64	-0.07	0.9428

Least Squares Means Estimate						
Effect	Label	Estimate	Standard Error	DF	t Value	Pr > |t|
GameVisit*Group	Veg - Control, GV1	-4.8845	14.4514	47.2	-0.34	0.7369

Least Squares Means Estimate						
Effect	Label	Estimate	Standard Error	DF	t Value	Pr > |t|
GameVisit*Group	Veg - Control, GV2	-13.6901	14.4514	47.2	-0.95	0.3483

Least Squares Means Estimate						
Effect	Label	Estimate	Standard Error	DF	t Value	Pr > |t|
GameVisit*Group	Veg - Control, GV3	-9.9193	14.4514	47.2	-0.69	0.4958

Least Squares Means							
Effect	Group	GameVisit	Estimate	Standard Error	DF	t Value	Pr > |t|
Group	Vegetable		7.4052	9.5750	31	0.77	0.4452
Group	Control		16.9032	9.4288	31	1.79	0.0828
GameVisit		1	10.6067	7.6913	44.9	1.38	0.1747
GameVisit		2	14.4540	7.6913	44.9	1.88	0.0667
GameVisit		3	11.4019	7.6913	44.9	1.48	0.1452
GameVisit*Group	Vegetable	1	8.1645	10.5601	44.8	0.77	0.4435
GameVisit*Group	Control	1	13.0490	10.5460	47.1	1.24	0.2221
GameVisit*Group	Vegetable	2	7.6089	10.5601	44.8	0.72	0.4749
GameVisit*Group	Control	2	21.2990	10.5460	47.1	2.02	0.0491
GameVisit*Group	Vegetable	3	6.4422	10.5601	44.8	0.61	0.5449
GameVisit*Group	Control	3	16.3615	10.5460	47.1	1.55	0.1275

Differences of Least Squares Means									
Effect	Group	GameVisit	_Group	_GameVisit	Estimate	Standard Error	DF	t Value	Pr > |t|
Group	Vegetable		Control		-9.4980	12.9110	31	-0.74	0.4675

Differences of Least Squares Means									
**Effect**	**Group**	**GameVisit**	**_Group**	**_GameVisit**	**Estimate**	**Standard Error**	**DF**	**t Value**	**Pr > |t|**
**GameVisit**		1		2	-3.8472	5.6225	64	-0.68	0.4963
**GameVisit**		1		3	-0.7951	5.6225	64	-0.14	0.8880
**GameVisit**		2		3	3.0521	5.6225	64	0.54	0.5891
**GameVisit*Group**	Vegetable	1	Control	1	-4.8845	14.4514	47.2	-0.34	0.7369
**GameVisit*Group**	Vegetable	1	Vegetable	2	0.5556	7.7141	64	0.07	0.9428
**GameVisit*Group**	Vegetable	1	Control	2	-13.1345	14.4514	47.2	-0.91	0.3680
**GameVisit*Group**	Vegetable	1	Vegetable	3	1.7222	7.7141	64	0.22	0.8240
**GameVisit*Group**	Vegetable	1	Control	3	-8.1970	14.4514	47.2	-0.57	0.5733
**GameVisit*Group**	Control	1	Vegetable	2	5.4401	14.4514	47.2	0.38	0.7083
**GameVisit*Group**	Control	1	Control	2	-8.2500	8.1820	64	-1.01	0.3171
**GameVisit*Group**	Control	1	Vegetable	3	6.6068	14.4514	47.2	0.46	0.6496
**GameVisit*Group**	Control	1	Control	3	-3.3125	8.1820	64	-0.40	0.6869
**GameVisit*Group**	Vegetable	2	Control	2	-13.6901	14.4514	47.2	-0.95	0.3483
**GameVisit*Group**	Vegetable	2	Vegetable	3	1.1667	7.7141	64	0.15	0.8803
**GameVisit*Group**	Vegetable	2	Control	3	-8.7526	14.4514	47.2	-0.61	0.5476
**GameVisit*Group**	Control	2	Vegetable	3	14.8568	14.4514	47.2	1.03	0.3092
**GameVisit*Group**	Control	2	Control	3	4.9375	8.1820	64	0.60	0.5483
**GameVisit*Group**	Vegetable	3	Control	3	-9.9193	14.4514	47.2	-0.69	0.4958

OSF planned analysis: Liking

The Mixed Procedure

SampleName=Broccoli puree

Model Information	
**Data Set**	WORK.GAMEVISITS
**Dependent Variable**	Liking
**Covariance Structure**	Compound Symmetry
**Subject Effect**	ParticipantID
**Estimation Method**	REML
**Residual Variance Method**	Profile
**Fixed Effects SE Method**	Kenward-Roger
**Degrees of Freedom Method**	Kenward-Roger

Class Level Information		
**Class**	**Levels**	**Values**
**ParticipantID**	34	1001 1002 1005 1006 1009 1010 1014 1016 1019 1020 1024 1025 1027 1029 1030 1034 1035 1041 1045 1046 1048 1049 1050 1051 1056 1061 1063 1064 1065 1066 1068 1069 1074 1078
**GameVisit**	3	1 2 3
**Group**	2	Vegetable Control
**Gender**	2	Man Woman
**SampleName**	1	Broccoli puree

Dimensions	
Covariance Parameters	2
Columns in X	14
Columns in Z	0
Subjects	34
Max Obs per Subject	3

Number of Observations	
Number of Observations Read	102
Number of Observations Used	102
Number of Observations Not Used	0

Iteration History			
Iteration	Evaluations	-2 Res Log Like	Criterion
0	1	964.08792202	
1	1	922.84396980	0.00000000

Convergence criteria met.

Covariance Parameter Estimates		
Cov Parm	Subject	Estimate
CS	ParticipantID	807.97
Residual		420.71

Fit Statistics	
-2 Res Log Likelihood	922.8
AIC (Smaller is Better)	926.8
AICC (Smaller is Better)	927.0
BIC (Smaller is Better)	929.9

Null Model Likelihood Ratio Test		
DF	Chi-Square	Pr > ChiSq
1	41.24	<.0001

Solution for Fixed Effects								
Effect	Group	Gender	GameVisit	Estimate	Standard Error	DF	t Value	Pr > |t|
Intercept				13.3403	9.8095	45.3	1.36	0.1806
GameVisit			1	-2.0625	7.2518	64	-0.28	0.7770
GameVisit			2	-8.2500	7.2518	64	-1.14	0.2595
GameVisit			3	0	.	.	.	.
Group	Vegetable			5.1576	12.1769	49.4	0.42	0.6737
Group	Control			0	.	.	.	.
GameVisit*Group	Vegetable		1	1.7292	9.9666	64	0.17	0.8628
GameVisit*Group	Control		1	0	.	.	.	.
GameVisit*Group	Vegetable		2	4.0833	9.9666	64	0.41	0.6834

Solution for Fixed Effects								
**Effect**	**Group**	**Gender**	**GameVisit**	**Estimate**	**Standard Error**	**DF**	**t Value**	**Pr > |t|**
GameVisit*Group	Control		2	0	.	.	.	.
GameVisit*Group	Vegetable		3	0	.	.	.	.
GameVisit*Group	Control		3	0	.	.	.	.
Gender		Man		-5.2409	11.7558	31	-0.45	0.6588
Gender		Woman		0	.	.	.	.

Type 3 Tests of Fixed Effects				
**Effect**	**Num DF**	**Den DF**	**F Value**	**Pr > F**
GameVisit	2	64	0.87	0.4224
Group	1	31	0.44	0.5134
GameVisit*Group	2	64	0.08	0.9190
Gender	1	31	0.20	0.6588

Least Squares Means Estimate						
**Effect**	**Label**	**Estimate**	**Standard Error**	**DF**	**t Value**	**Pr > |t|**
GameVisit*Group	Control, GV3-GV1	2.0625	7.2518	64	0.28	0.7770

Least Squares Means Estimate						
**Effect**	**Label**	**Estimate**	**Standard Error**	**DF**	**t Value**	**Pr > |t|**
GameVisit*Group	Control, GV2-GV1	-6.1875	7.2518	64	-0.85	0.3967

Least Squares Means Estimate						
**Effect**	**Label**	**Estimate**	**Standard Error**	**DF**	**t Value**	**Pr > |t|**
GameVisit*Group	Veg, GV3-GV1	0.3333	6.8370	64	0.05	0.9613

Least Squares Means Estimate						
**Effect**	**Label**	**Estimate**	**Standard Error**	**DF**	**t Value**	**Pr > |t|**
GameVisit*Group	Veg, GV2-GV1	-3.8333	6.8370	64	-0.56	0.5770

Least Squares Means Estimate						
**Effect**	**Label**	**Estimate**	**Standard Error**	**DF**	**t Value**	**Pr > |t|**
GameVisit*Group	Veg - Control, GV1	6.8868	12.1769	49.41	0.57	0.5743

Least Squares Means Estimate						
**Effect**	**Label**	**Estimate**	**Standard Error**	**DF**	**t Value**	**Pr > |t|**
GameVisit*Group	Veg - Control, GV2	9.2410	12.1769	49.41	0.76	0.4515

Least Squares Means Estimate						
**Effect**	**Label**	**Estimate**	**Standard Error**	**DF**	**t Value**	**Pr > |t|**
GameVisit*Group	Veg - Control, GV3	5.1576	12.1769	49.41	0.42	0.6737

Least Squares Means							
**Effect**	**Group**	**GameVisit**	**Estimate**	**Standard Error**	**DF**	**t Value**	**Pr > |t|**

Least Squares Means							
Effect	Group	GameVisit	Estimate	Standard Error	DF	t Value	Pr > |t|
Group	Vegetable		14.3775	7.9587	31	1.81	0.0806
Group	Control		7.2824	7.8372	31	0.93	0.3600
GameVisit		1	12.1008	6.4705	46.8	1.87	0.0677
GameVisit		2	7.0904	6.4705	46.8	1.10	0.2788
GameVisit		3	13.2987	6.4705	46.8	2.06	0.0455
GameVisit*Group	Vegetable	1	15.5442	8.8839	46.8	1.75	0.0867
GameVisit*Group	Control	1	8.6574	8.8855	49.3	0.97	0.3347
GameVisit*Group	Vegetable	2	11.7109	8.8839	46.8	1.32	0.1939
GameVisit*Group	Control	2	2.4699	8.8855	49.3	0.28	0.7822
GameVisit*Group	Vegetable	3	15.8775	8.8839	46.8	1.79	0.0804
GameVisit*Group	Control	3	10.7199	8.8855	49.3	1.21	0.2334

Differences of Least Squares Means									
Effect	Group	GameVisit	_Group	_GameVisit	Estimate	Standard Error	DF	t Value	Pr > |t|
Group	Vegetable		Control		7.0951	10.7316	31	0.66	0.5134
GameVisit		1		2	5.0104	4.9833	64	1.01	0.3185
GameVisit		1		3	-1.1979	4.9833	64	-0.24	0.8108
GameVisit		2		3	-6.2083	4.9833	64	-1.25	0.2174
GameVisit*Group	Vegetable	1	Control	1	6.8868	12.1769	49.4	0.57	0.5743
GameVisit*Group	Vegetable	1	Vegetable	2	3.8333	6.8370	64	0.56	0.5770
GameVisit*Group	Vegetable	1	Control	2	13.0743	12.1769	49.4	1.07	0.2882
GameVisit*Group	Vegetable	1	Vegetable	3	-0.3333	6.8370	64	-0.05	0.9613
GameVisit*Group	Vegetable	1	Control	3	4.8243	12.1769	49.4	0.40	0.6937
GameVisit*Group	Control	1	Vegetable	2	-3.0535	12.1769	49.4	-0.25	0.8030
GameVisit*Group	Control	1	Control	2	6.1875	7.2518	64	0.85	0.3967
GameVisit*Group	Control	1	Vegetable	3	-7.2201	12.1769	49.4	-0.59	0.5559
GameVisit*Group	Control	1	Control	3	-2.0625	7.2518	64	-0.28	0.7770
GameVisit*Group	Vegetable	2	Control	2	9.2410	12.1769	49.4	0.76	0.4515
GameVisit*Group	Vegetable	2	Vegetable	3	-4.1667	6.8370	64	-0.61	0.5444
GameVisit*Group	Vegetable	2	Control	3	0.9910	12.1769	49.4	0.08	0.9355
GameVisit*Group	Control	2	Vegetable	3	-13.4076	12.1769	49.4	-1.10	0.2762
GameVisit*Group	Control	2	Control	3	-8.2500	7.2518	64	-1.14	0.2595
GameVisit*Group	Vegetable	3	Control	3	5.1576	12.1769	49.4	0.42	0.6737

OSF planned analysis: Liking

The Mixed Procedure

SampleName=Chicken puree

Model Information	
Data Set	WORK.GAMEVISITS

Model Information	
Dependent Variable	Liking
Covariance Structure	Compound Symmetry
Subject Effect	ParticipantID
Estimation Method	REML
Residual Variance Method	Profile
Fixed Effects SE Method	Kenward-Roger
Degrees of Freedom Method	Kenward-Roger

Class Level Information		
Class	Levels	Values
ParticipantID	34	1001 1002 1005 1006 1009 1010 1014 1016 1019 1020 1024 1025 1027 1029 1030 1034 1035 1041 1045 1046 1048 1049 1050 1051 1056 1061 1063 1064 1065 1066 1068 1069 1074 1078
GameVisit	3	1 2 3
Group	2	Vegetable Control
Gender	2	Man Woman
SampleName	1	Chicken puree

Dimensions	
Covariance Parameters	2
Columns in X	14
Columns in Z	0
Subjects	34
Max Obs per Subject	3

Number of Observations	
Number of Observations Read	102
Number of Observations Used	102
Number of Observations Not Used	0

Iteration History			
Iteration	Evaluations	-2 Res Log Like	Criterion
0	1	1053.36054611	
1	1	974.01303114	0.00000000

Convergence criteria met.

Covariance Parameter Estimates		
Cov Parm	Subject	Estimate
CS	ParticipantID	2600.40
Residual		555.28

Fit Statistics	
-2 Res Log Likelihood	974.0
AIC (Smaller is Better)	978.0
AICC (Smaller is Better)	978.1

Fit Statistics	
BIC (Smaller is Better)	981.1

Null Model Likelihood Ratio Test		
DF	Chi-Square	Pr > ChiSq
1	79.35	<.0001

Solution for Fixed Effects								
Effect	Group	Gender	GameVisit	Estimate	Standard Error	DF	t Value	Pr > |t|
Intercept				-9.6323	15.9475	37.3	-0.60	0.5495
GameVisit			1	-6.5000	8.3313	64	-0.78	0.4382
GameVisit			2	-0.06250	8.3313	64	-0.01	0.9940
GameVisit			3	0	.	.	.	.
Group	Vegetable			-11.2424	19.5454	39.2	-0.58	0.5684
Group	Control			0	.	.	.	.
GameVisit*Group	Vegetable		1	-4.4444	11.4503	64	-0.39	0.6992
GameVisit*Group	Control		1	0	.	.	.	.
GameVisit*Group	Vegetable		2	-1.8264	11.4503	64	-0.16	0.8738
GameVisit*Group	Control		2	0	.	.	.	.
GameVisit*Group	Vegetable		3	0	.	.	.	.
GameVisit*Group	Control		3	0	.	.	.	.
Gender		Man		21.6862	20.1490	31	1.08	0.2901
Gender		Woman		0	.	.	.	.

Type 3 Tests of Fixed Effects				
Effect	Num DF	Den DF	F Value	Pr > F
GameVisit	2	64	1.39	0.2556
Group	1	31	0.53	0.4740
GameVisit*Group	2	64	0.08	0.9268
Gender	1	31	1.16	0.2901

Least Squares Means Estimate						
Effect	Label	Estimate	Standard Error	DF	t Value	Pr > |t|
GameVisit*Group	Control, GV3-GV1	6.5000	8.3313	64	0.78	0.4382

Least Squares Means Estimate						
Effect	Label	Estimate	Standard Error	DF	t Value	Pr > |t|
GameVisit*Group	Control, GV2-GV1	6.4375	8.3313	64	0.77	0.4426

Least Squares Means Estimate						
Effect	Label	Estimate	Standard Error	DF	t Value	Pr > |t|
GameVisit*Group	Veg, GV3-GV1	10.9444	7.8548	64	1.39	0.1683

Least Squares Means Estimate						
Effect	Label	Estimate	Standard Error	DF	t Value	Pr > |t|

Least Squares Means Estimate						
Effect	Label	Estimate	Standard Error	DF	t Value	Pr > |t|
GameVisit*Group	Veg, GV2-GV1	9.0556	7.8548	64	1.15	0.2533

Least Squares Means Estimate						
Effect	Label	Estimate	Standard Error	DF	t Value	Pr > |t|
GameVisit*Group	Veg - Control, GV1	-15.6868	19.5454	39.21	-0.80	0.4271

Least Squares Means Estimate						
Effect	Label	Estimate	Standard Error	DF	t Value	Pr > |t|
GameVisit*Group	Veg - Control, GV2	-13.0688	19.5454	39.21	-0.67	0.5076

Least Squares Means Estimate						
Effect	Label	Estimate	Standard Error	DF	t Value	Pr > |t|
GameVisit*Group	Veg - Control, GV3	-11.2424	19.5454	39.21	-0.58	0.5684

Least Squares Means							
Effect	Group	GameVisit	Estimate	Standard Error	DF	t Value	Pr > |t|
Group	Vegetable		-14.3094	13.6410	31	-1.05	0.3023
Group	Control		-0.9767	13.4327	31	-0.07	0.9425
GameVisit		1	-13.1326	10.4691	38	-1.25	0.2173
GameVisit		2	-5.3861	10.4691	38	-0.51	0.6099
GameVisit		3	-4.4104	10.4691	38	-0.42	0.6759
GameVisit*Group	Vegetable	1	-20.9760	14.3750	38	-1.46	0.1527
GameVisit*Group	Control	1	-5.2892	14.2679	39.1	-0.37	0.7129
GameVisit*Group	Vegetable	2	-11.9205	14.3750	38	-0.83	0.4121
GameVisit*Group	Control	2	1.1483	14.2679	39.1	0.08	0.9363
GameVisit*Group	Vegetable	3	-10.0316	14.3750	38	-0.70	0.4895
GameVisit*Group	Control	3	1.2108	14.2679	39.1	0.08	0.9328

Differences of Least Squares Means									
Effect	Group	GameVisit	_Group	_GameVisit	Estimate	Standard Error	DF	t Value	Pr > |t|
Group	Vegetable		Control		-13.3327	18.3935	31	-0.72	0.4740
GameVisit		1		2	-7.7465	5.7251	64	-1.35	0.1808
GameVisit		1		3	-8.7222	5.7251	64	-1.52	0.1326
GameVisit		2		3	-0.9757	5.7251	64	-0.17	0.8652
GameVisit*Group	Vegetable	1	Control	1	-15.6868	19.5454	39.2	-0.80	0.4271
GameVisit*Group	Vegetable	1	Vegetable	2	-9.0556	7.8548	64	-1.15	0.2533
GameVisit*Group	Vegetable	1	Control	2	-22.1243	19.5454	39.2	-1.13	0.2645
GameVisit*Group	Vegetable	1	Vegetable	3	-10.9444	7.8548	64	-1.39	0.1683
GameVisit*Group	Vegetable	1	Control	3	-22.1868	19.5454	39.2	-1.14	0.2632
GameVisit*Group	Control	1	Vegetable	2	6.6313	19.5454	39.2	0.34	0.7362
GameVisit*Group	Control	1	Control	2	-6.4375	8.3313	64	-0.77	0.4426
GameVisit*Group	Control	1	Vegetable	3	4.7424	19.5454	39.2	0.24	0.8096

Differences of Least Squares Means									
Effect	Group	GameVisit	_Group	_GameVisit	Estimate	Standard Error	DF	t Value	Pr > |t|
GameVisit*Group	Control	1	Control	3	-6.5000	8.3313	64	-0.78	0.4382
GameVisit*Group	Vegetable	2	Control	2	-13.0688	19.5454	39.2	-0.67	0.5076
GameVisit*Group	Vegetable	2	Vegetable	3	-1.8889	7.8548	64	-0.24	0.8107
GameVisit*Group	Vegetable	2	Control	3	-13.1313	19.5454	39.2	-0.67	0.5056
GameVisit*Group	Control	2	Vegetable	3	11.1799	19.5454	39.2	0.57	0.5706
GameVisit*Group	Control	2	Control	3	-0.06250	8.3313	64	-0.01	0.9940
GameVisit*Group	Vegetable	3	Control	3	-11.2424	19.5454	39.2	-0.58	0.5684

OSF planned analysis: Liking

The Mixed Procedure

SampleName=Kale chopped

Model Information	
Data Set	WORK.GAMEVISITS
Dependent Variable	Liking
Covariance Structure	Compound Symmetry
Subject Effect	ParticipantID
Estimation Method	REML
Residual Variance Method	Profile
Fixed Effects SE Method	Kenward-Roger
Degrees of Freedom Method	Kenward-Roger

Class Level Information		
Class	Levels	Values
ParticipantID	34	1001 1002 1005 1006 1009 1010 1014 1016 1019 1020 1024 1025 1027 1029 1030 1034 1035 1041 1045 1046 1048 1049 1050 1051 1056 1061 1063 1064 1065 1066 1068 1069 1074 1078
GameVisit	3	1 2 3
Group	2	Vegetable Control
Gender	2	Man Woman
SampleName	1	Kale chopped

Dimensions	
Covariance Parameters	2
Columns in X	14
Columns in Z	0
Subjects	34
Max Obs per Subject	3

Number of Observations	
Number of Observations Read	102
Number of Observations Used	102

Number of Observations	
Number of Observations Not Used	0

Iteration History			
Iteration	Evaluations	-2 Res Log Like	Criterion
0	1	971.95335673	
1	1	930.34271996	0.00000000

Convergence criteria met.

Covariance Parameter Estimates		
Cov Parm	Subject	Estimate
CS	ParticipantID	880.84
Residual		453.96

Fit Statistics	
-2 Res Log Likelihood	930.3
AIC (Smaller is Better)	934.3
AICC (Smaller is Better)	934.5
BIC (Smaller is Better)	937.4

Null Model Likelihood Ratio Test		
DF	Chi-Square	Pr > ChiSq
1	41.61	<.0001

Solution for Fixed Effects								
Effect	Group	Gender	GameVisit	Estimate	Standard Error	DF	t Value	Pr > |t|
Intercept				-28.9762	10.2264	45.1	-2.83	0.0069
GameVisit			1	9.3750	7.5329	64	1.24	0.2178
GameVisit			2	2.2500	7.5329	64	0.30	0.7661
GameVisit			3	0	.	.	.	.
Group	Vegetable			13.6755	12.6922	49.3	1.08	0.2865
Group	Control			0	.	.	.	.
GameVisit*Group	Vegetable		1	-28.9861	10.3530	64	-2.80	0.0068
GameVisit*Group	Control		1	0	.	.	.	.
GameVisit*Group	Vegetable		2	-4.3056	10.3530	64	-0.42	0.6789
GameVisit*Group	Control		2	0	.	.	.	.
GameVisit*Group	Vegetable		3	0	.	.	.	.
GameVisit*Group	Control		3	0	.	.	.	.
Gender		Man		13.6032	12.2652	31	1.11	0.2759
Gender		Woman		0	.	.	.	.

Type 3 Tests of Fixed Effects				
Effect	Num DF	Den DF	F Value	Pr > F
GameVisit	2	64	0.66	0.5181

Type 3 Tests of Fixed Effects				
Effect	Num DF	Den DF	F Value	Pr > F
Group	1	31	0.05	0.8194
GameVisit*Group	2	64	4.56	0.0140
Gender	1	31	1.23	0.2759

Least Squares Means Estimate						
Effect	Label	Estimate	Standard Error	DF	t Value	Pr > |t|
GameVisit*Group	Control, GV3-GV1	-9.3750	7.5329	64	-1.24	0.2178

Least Squares Means Estimate						
Effect	Label	Estimate	Standard Error	DF	t Value	Pr > |t|
GameVisit*Group	Control, GV2-GV1	-7.1250	7.5329	64	-0.95	0.3478

Least Squares Means Estimate						
Effect	Label	Estimate	Standard Error	DF	t Value	Pr > |t|
GameVisit*Group	Veg, GV3-GV1	19.6111	7.1021	64	2.76	0.0075

Least Squares Means Estimate						
Effect	Label	Estimate	Standard Error	DF	t Value	Pr > |t|
GameVisit*Group	Veg, GV2-GV1	17.5556	7.1021	64	2.47	0.0161

Least Squares Means Estimate						
Effect	Label	Estimate	Standard Error	DF	t Value	Pr > |t|
GameVisit*Group	Veg - Control, GV1	-15.3106	12.6922	49.25	-1.21	0.2335

Least Squares Means Estimate						
Effect	Label	Estimate	Standard Error	DF	t Value	Pr > |t|
GameVisit*Group	Veg - Control, GV2	9.3699	12.6922	49.25	0.74	0.4639

Least Squares Means Estimate						
Effect	Label	Estimate	Standard Error	DF	t Value	Pr > |t|
GameVisit*Group	Veg - Control, GV3	13.6755	12.6922	49.25	1.08	0.2865

Least Squares Means							
Effect	Group	GameVisit	Estimate	Standard Error	DF	t Value	Pr > |t|
Group	Vegetable		-15.7213	8.3036	31	-1.89	0.0677
Group	Control		-18.2996	8.1768	31	-2.24	0.0325
GameVisit		1	-20.4549	6.7451	46.6	-3.03	0.0040
GameVisit		2	-15.2396	6.7451	46.6	-2.26	0.0286
GameVisit		3	-15.3368	6.7451	46.6	-2.27	0.0276
GameVisit*Group	Vegetable	1	-28.1102	9.2608	46.6	-3.04	0.0039
GameVisit*Group	Control	1	-12.7996	9.2615	49.1	-1.38	0.1732
GameVisit*Group	Vegetable	2	-10.5547	9.2608	46.6	-1.14	0.2602
GameVisit*Group	Control	2	-19.9246	9.2615	49.1	-2.15	0.0364

Least Squares Means							
Effect	Group	GameVisit	Estimate	Standard Error	DF	t Value	Pr > |t|
GameVisit*Group	Vegetable	3	-8.4991	9.2608	46.6	-0.92	0.3635
GameVisit*Group	Control	3	-22.1746	9.2615	49.1	-2.39	0.0205

Differences of Least Squares Means									
Effect	Group	GameVisit	_Group	_GameVisit	Estimate	Standard Error	DF	t Value	Pr > |t|
Group	Vegetable		Control		2.5783	11.1966	31	0.23	0.8194
GameVisit		1		2	-5.2153	5.1765	64	-1.01	0.3175
GameVisit		1		3	-5.1181	5.1765	64	-0.99	0.3265
GameVisit		2		3	0.09722	5.1765	64	0.02	0.9851
GameVisit*Group	Vegetable	1	Control	1	-15.3106	12.6922	49.3	-1.21	0.2335
GameVisit*Group	Vegetable	1	Vegetable	2	-17.5556	7.1021	64	-2.47	0.0161
GameVisit*Group	Vegetable	1	Control	2	-8.1856	12.6922	49.3	-0.64	0.5220
GameVisit*Group	Vegetable	1	Vegetable	3	-19.6111	7.1021	64	-2.76	0.0075
GameVisit*Group	Vegetable	1	Control	3	-5.9356	12.6922	49.3	-0.47	0.6421
GameVisit*Group	Control	1	Vegetable	2	-2.2449	12.6922	49.3	-0.18	0.8603
GameVisit*Group	Control	1	Control	2	7.1250	7.5329	64	0.95	0.3478
GameVisit*Group	Control	1	Vegetable	3	-4.3005	12.6922	49.3	-0.34	0.7362
GameVisit*Group	Control	1	Control	3	9.3750	7.5329	64	1.24	0.2178
GameVisit*Group	Vegetable	2	Control	2	9.3699	12.6922	49.3	0.74	0.4639
GameVisit*Group	Vegetable	2	Vegetable	3	-2.0556	7.1021	64	-0.29	0.7732
GameVisit*Group	Vegetable	2	Control	3	11.6199	12.6922	49.3	0.92	0.3644
GameVisit*Group	Control	2	Vegetable	3	-11.4255	12.6922	49.3	-0.90	0.3724
GameVisit*Group	Control	2	Control	3	2.2500	7.5329	64	0.30	0.7661
GameVisit*Group	Vegetable	3	Control	3	13.6755	12.6922	49.3	1.08	0.2865

OSF planned analysis: Liking

The Mixed Procedure

SampleName=Kale puree

Model Information	
Data Set	WORK.GAMEVISITS
Dependent Variable	Liking
Covariance Structure	Compound Symmetry
Subject Effect	ParticipantID
Estimation Method	REML
Residual Variance Method	Profile
Fixed Effects SE Method	Kenward-Roger
Degrees of Freedom Method	Kenward-Roger

Class Level Information		
Class	Levels	Values

Class Level Information		
**Class**	**Levels**	**Values**
**ParticipantID**	34	1001 1002 1005 1006 1009 1010 1014 1016 1019 1020 1024 1025 1027 1029 1030 1034 1035 1041 1045 1046 1048 1049 1050 1051 1056 1061 1063 1064 1065 1066 1068 1069 1074 1078
**GameVisit**	3	1 2 3
**Group**	2	Vegetable Control
**Gender**	2	Man Woman
**SampleName**	1	Kale puree

Dimensions	
**Covariance Parameters**	2
**Columns in X**	14
**Columns in Z**	0
**Subjects**	34
**Max Obs per Subject**	3

Number of Observations	
**Number of Observations Read**	102
**Number of Observations Used**	102
**Number of Observations Not Used**	0

Iteration History			
**Iteration**	**Evaluations**	**-2 Res Log Like**	**Criterion**
**0**	1	955.05709780	
**1**	1	918.52940770	0.00000000

Convergence criteria met.

Covariance Parameter Estimates		
**Cov Parm**	**Subject**	**Estimate**
**CS**	ParticipantID	699.01
**Residual**		417.49

Fit Statistics	
**-2 Res Log Likelihood**	918.5
**AIC (Smaller is Better)**	922.5
**AICC (Smaller is Better)**	922.7
**BIC (Smaller is Better)**	925.6

Null Model Likelihood Ratio Test		
**DF**	**Chi-Square**	**Pr > ChiSq**
1	36.53	<.0001

Solution for Fixed Effects								
**Effect**	**Group**	**Gender**	**GameVisit**	**Estimate**	**Standard Error**	**DF**	**t Value**	**Pr > |t|**
**Intercept**				-25.9433	9.3253	47	-2.78	0.0078

Solution for Fixed Effects								
Effect	Group	Gender	GameVisit	Estimate	Standard Error	DF	t Value	Pr > |t|
GameVisit			1	19.1250	7.2240	64	2.65	0.0102
GameVisit			2	4.3750	7.2240	64	0.61	0.5469
GameVisit			3	0	.	.	.	.
Group	Vegetable			17.9399	11.6043	51.6	1.55	0.1282
Group	Control			0	.	.	.	.
GameVisit*Group	Vegetable		1	-29.5694	9.9284	64	-2.98	0.0041
GameVisit*Group	Control		1	0	.	.	.	.
GameVisit*Group	Vegetable		2	-12.0417	9.9284	64	-1.21	0.2296
GameVisit*Group	Control		2	0	.	.	.	.
GameVisit*Group	Vegetable		3	0	.	.	.	.
GameVisit*Group	Control		3	0	.	.	.	.
Gender		Man		2.0155	11.0528	31	0.18	0.8565
Gender		Woman		0	.	.	.	.

Type 3 Tests of Fixed Effects				
Effect	Num DF	Den DF	F Value	Pr > F
GameVisit	2	64	0.78	0.4645
Group	1	31	0.16	0.6895
GameVisit*Group	2	64	4.49	0.0150
Gender	1	31	0.03	0.8565

Least Squares Means Estimate						
Effect	Label	Estimate	Standard Error	DF	t Value	Pr > |t|
GameVisit*Group	Control, GV3-GV1	-19.1250	7.2240	64	-2.65	0.0102

Least Squares Means Estimate						
Effect	Label	Estimate	Standard Error	DF	t Value	Pr > |t|
GameVisit*Group	Control, GV2-GV1	-14.7500	7.2240	64	-2.04	0.0453

Least Squares Means Estimate						
Effect	Label	Estimate	Standard Error	DF	t Value	Pr > |t|
GameVisit*Group	Veg, GV3-GV1	10.4444	6.8109	64	1.53	0.1301

Least Squares Means Estimate						
Effect	Label	Estimate	Standard Error	DF	t Value	Pr > |t|
GameVisit*Group	Veg, GV2-GV1	2.7778	6.8109	64	0.41	0.6847

Least Squares Means Estimate						
Effect	Label	Estimate	Standard Error	DF	t Value	Pr > |t|
GameVisit*Group	Veg - Control, GV1	-11.6296	11.6043	51.64	-1.00	0.3209

Least Squares Means Estimate						
Effect	Label	Estimate	Standard Error	DF	t Value	Pr > |t|

Least Squares Means Estimate						
Effect	Label	Estimate	Standard Error	DF	t Value	Pr > |t|
GameVisit*Group	Veg - Control, GV2	5.8982	11.6043	51.64	0.51	0.6134

Least Squares Means Estimate						
Effect	Label	Estimate	Standard Error	DF	t Value	Pr > |t|
GameVisit*Group	Veg - Control, GV3	17.9399	11.6043	51.64	1.55	0.1282

Least Squares Means							
Effect	Group	GameVisit	Estimate	Standard Error	DF	t Value	Pr > |t|
Group	Vegetable		-13.0327	7.4827	31	-1.74	0.0915
Group	Control		-17.1022	7.3685	31	-2.32	0.0270
GameVisit		1	-11.6253	6.1569	48.7	-1.89	0.0650
GameVisit		2	-17.6115	6.1569	48.7	-2.86	0.0062
GameVisit		3	-15.9656	6.1569	48.7	-2.59	0.0125
GameVisit*Group	Vegetable	1	-17.4401	8.4531	48.7	-2.06	0.0444
GameVisit*Group	Control	1	-5.8106	8.4670	51.5	-0.69	0.4956
GameVisit*Group	Vegetable	2	-14.6624	8.4531	48.7	-1.73	0.0891
GameVisit*Group	Control	2	-20.5606	8.4670	51.5	-2.43	0.0187
GameVisit*Group	Vegetable	3	-6.9957	8.4531	48.7	-0.83	0.4119
GameVisit*Group	Control	3	-24.9356	8.4670	51.5	-2.95	0.0048

Differences of Least Squares Means									
Effect	Group	GameVisit	_Group	_GameVisit	Estimate	Standard Error	DF	t Value	Pr > |t|
Group	Vegetable		Control		4.0695	10.0897	31	0.40	0.6895
GameVisit		1		2	5.9861	4.9642	64	1.21	0.2323
GameVisit		1		3	4.3403	4.9642	64	0.87	0.3852
GameVisit		2		3	-1.6458	4.9642	64	-0.33	0.7413
GameVisit*Group	Vegetable	1	Control	1	-11.6296	11.6043	51.6	-1.00	0.3209
GameVisit*Group	Vegetable	1	Vegetable	2	-2.7778	6.8109	64	-0.41	0.6847
GameVisit*Group	Vegetable	1	Control	2	3.1204	11.6043	51.6	0.27	0.7891
GameVisit*Group	Vegetable	1	Vegetable	3	-10.4444	6.8109	64	-1.53	0.1301
GameVisit*Group	Vegetable	1	Control	3	7.4954	11.6043	51.6	0.65	0.5212
GameVisit*Group	Control	1	Vegetable	2	8.8518	11.6043	51.6	0.76	0.4491
GameVisit*Group	Control	1	Control	2	14.7500	7.2240	64	2.04	0.0453
GameVisit*Group	Control	1	Vegetable	3	1.1851	11.6043	51.6	0.10	0.9191
GameVisit*Group	Control	1	Control	3	19.1250	7.2240	64	2.65	0.0102
GameVisit*Group	Vegetable	2	Control	2	5.8982	11.6043	51.6	0.51	0.6134
GameVisit*Group	Vegetable	2	Vegetable	3	-7.6667	6.8109	64	-1.13	0.2645
GameVisit*Group	Vegetable	2	Control	3	10.2732	11.6043	51.6	0.89	0.3801
GameVisit*Group	Control	2	Vegetable	3	-13.5649	11.6043	51.6	-1.17	0.2478
GameVisit*Group	Control	2	Control	3	4.3750	7.2240	64	0.61	0.5469
GameVisit*Group	Vegetable	3	Control	3	17.9399	11.6043	51.6	1.55	0.1282

OSF planned analysis: Liking

The Mixed Procedure

SampleName=Oat puree

Model Information	
Data Set	WORK.GAMEVISITS
Dependent Variable	Liking
Covariance Structure	Compound Symmetry
Subject Effect	ParticipantID
Estimation Method	REML
Residual Variance Method	Profile
Fixed Effects SE Method	Kenward-Roger
Degrees of Freedom Method	Kenward-Roger

Class Level Information		
Class	Levels	Values
ParticipantID	34	1001 1002 1005 1006 1009 1010 1014 1016 1019 1020 1024 1025 1027 1029 1030 1034 1035 1041 1045 1046 1048 1049 1050 1051 1056 1061 1063 1064 1065 1066 1068 1069 1074 1078
GameVisit	3	1 2 3
Group	2	Vegetable Control
Gender	2	Man Woman
SampleName	1	Oat puree

Dimensions	
Covariance Parameters	2
Columns in X	14
Columns in Z	0
Subjects	34
Max Obs per Subject	3

Number of Observations	
Number of Observations Read	102
Number of Observations Used	102
Number of Observations Not Used	0

Iteration History			
Iteration	Evaluations	-2 Res Log Like	Criterion
0	1	960.99320580	
1	1	941.97468856	0.00000000

Convergence criteria met.

Covariance Parameter Estimates		
Cov Parm	Subject	Estimate
CS	ParticipantID	555.00

Covariance Parameter Estimates		
Cov Parm	Subject	Estimate
Residual		629.51

Fit Statistics	
-2 Res Log Likelihood	942.0
AIC (Smaller is Better)	946.0
AICC (Smaller is Better)	946.1
BIC (Smaller is Better)	949.0

Null Model Likelihood Ratio Test		
DF	Chi-Square	Pr > ChiSq
1	19.02	<.0001

Solution for Fixed Effects								
Effect	Group	Gender	GameVisit	Estimate	Standard Error	DF	t Value	Pr > |t|
Intercept				-10.4378	9.4714	57.2	-1.10	0.2751
GameVisit			1	-11.7500	8.8707	64	-1.32	0.1900
GameVisit			2	-5.1250	8.8707	64	-0.58	0.5655
GameVisit			3	0	.	.	.	.
Group	Vegetable			21.5858	11.9348	64.1	1.81	0.0752
Group	Control			0	.	.	.	.
GameVisit*Group	Vegetable		1	-5.1944	12.1916	64	-0.43	0.6715
GameVisit*Group	Control		1	0	.	.	.	.
GameVisit*Group	Vegetable		2	-10.0972	12.1916	64	-0.83	0.4106
GameVisit*Group	Control		2	0	.	.	.	.
GameVisit*Group	Vegetable		3	0	.	.	.	.
GameVisit*Group	Control		3	0	.	.	.	.
Gender		Man		4.3340	10.5581	31	0.41	0.6843
Gender		Woman		0	.	.	.	.

Type 3 Tests of Fixed Effects				
Effect	Num DF	Den DF	F Value	Pr > F
GameVisit	2	64	2.93	0.0605
Group	1	31	2.93	0.0971
GameVisit*Group	2	64	0.34	0.7109
Gender	1	31	0.17	0.6843

Least Squares Means Estimate						
Effect	Label	Estimate	Standard Error	DF	t Value	Pr > |t|
GameVisit*Group	Control, GV3-GV1	11.7500	8.8707	64	1.32	0.1900

Least Squares Means Estimate						
Effect	Label	Estimate	Standard Error	DF	t Value	Pr > |t|

Least Squares Means Estimate						
Effect	Label	Estimate	Standard Error	DF	t Value	Pr > |t|
GameVisit*Group	Control, GV2-GV1	6.6250	8.8707	64	0.75	0.4579

Least Squares Means Estimate						
Effect	Label	Estimate	Standard Error	DF	t Value	Pr > |t|
GameVisit*Group	Veg, GV3-GV1	16.9444	8.3634	64	2.03	0.0469

Least Squares Means Estimate						
Effect	Label	Estimate	Standard Error	DF	t Value	Pr > |t|
GameVisit*Group	Veg, GV2-GV1	1.7222	8.3634	64	0.21	0.8375

Least Squares Means Estimate						
Effect	Label	Estimate	Standard Error	DF	t Value	Pr > |t|
GameVisit*Group	Veg - Control, GV1	16.3913	11.9348	64.06	1.37	0.1744

Least Squares Means Estimate						
Effect	Label	Estimate	Standard Error	DF	t Value	Pr > |t|
GameVisit*Group	Veg - Control, GV2	11.4885	11.9348	64.06	0.96	0.3394

Least Squares Means Estimate						
Effect	Label	Estimate	Standard Error	DF	t Value	Pr > |t|
GameVisit*Group	Veg - Control, GV3	21.5858	11.9348	64.06	1.81	0.0752

Least Squares Means							
Effect	Group	GameVisit	Estimate	Standard Error	DF	t Value	Pr > |t|
Group	Vegetable		2.5928	7.1479	31	0.36	0.7193
Group	Control		-13.8957	7.0388	31	-1.97	0.0573
GameVisit		1	-11.8251	6.2834	59.8	-1.88	0.0647
GameVisit		2	-7.6515	6.2834	59.8	-1.22	0.2281
GameVisit		3	2.5221	6.2834	59.8	0.40	0.6896
GameVisit*Group	Vegetable	1	-3.6294	8.6260	59.7	-0.42	0.6754
GameVisit*Group	Control	1	-20.0207	8.7048	63.8	-2.30	0.0247
GameVisit*Group	Vegetable	2	-1.9072	8.6260	59.7	-0.22	0.8258
GameVisit*Group	Control	2	-13.3957	8.7048	63.8	-1.54	0.1288
GameVisit*Group	Vegetable	3	13.3150	8.6260	59.7	1.54	0.1280
GameVisit*Group	Control	3	-8.2707	8.7048	63.8	-0.95	0.3456

Differences of Least Squares Means									
Effect	Group	GameVisit	_Group	_GameVisit	Estimate	Standard Error	DF	t Value	Pr > |t|
Group	Vegetable		Control		16.4885	9.6382	31	1.71	0.0971
GameVisit		1		2	-4.1736	6.0958	64	-0.68	0.4960
GameVisit		1		3	-14.3472	6.0958	64	-2.35	0.0217
GameVisit		2		3	-10.1736	6.0958	64	-1.67	0.1000
GameVisit*Group	Vegetable	1	Control	1	16.3913	11.9348	64.1	1.37	0.1744

Differences of Least Squares Means									
Effect	Group	GameVisit	_Group	_GameVisit	Estimate	Standard Error	DF	t Value	Pr > |t|
GameVisit*Group	Vegetable	1	Vegetable	2	-1.7222	8.3634	64	-0.21	0.8375
GameVisit*Group	Vegetable	1	Control	2	9.7663	11.9348	64.1	0.82	0.4162
GameVisit*Group	Vegetable	1	Vegetable	3	-16.9444	8.3634	64	-2.03	0.0469
GameVisit*Group	Vegetable	1	Control	3	4.6413	11.9348	64.1	0.39	0.6986
GameVisit*Group	Control	1	Vegetable	2	-18.1135	11.9348	64.1	-1.52	0.1340
GameVisit*Group	Control	1	Control	2	-6.6250	8.8707	64	-0.75	0.4579
GameVisit*Group	Control	1	Vegetable	3	-33.3358	11.9348	64.1	-2.79	0.0069
GameVisit*Group	Control	1	Control	3	-11.7500	8.8707	64	-1.32	0.1900
GameVisit*Group	Vegetable	2	Control	2	11.4885	11.9348	64.1	0.96	0.3394
GameVisit*Group	Vegetable	2	Vegetable	3	-15.2222	8.3634	64	-1.82	0.0734
GameVisit*Group	Vegetable	2	Control	3	6.3635	11.9348	64.1	0.53	0.5957
GameVisit*Group	Control	2	Vegetable	3	-26.7108	11.9348	64.1	-2.24	0.0287
GameVisit*Group	Control	2	Control	3	-5.1250	8.8707	64	-0.58	0.5655
GameVisit*Group	Vegetable	3	Control	3	21.5858	11.9348	64.1	1.81	0.0752

OSF planned analysis: Liking

The Mixed Procedure

SampleName=Spinach chopped

Model Information	
Data Set	WORK.GAMEVISITS
Dependent Variable	Liking
Covariance Structure	Compound Symmetry
Subject Effect	ParticipantID
Estimation Method	REML
Residual Variance Method	Profile
Fixed Effects SE Method	Kenward-Roger
Degrees of Freedom Method	Kenward-Roger

Class Level Information		
Class	Levels	Values
ParticipantID	34	1001 1002 1005 1006 1009 1010 1014 1016 1019 1020 1024 1025 1027 1029 1030 1034 1035 1041 1045 1046 1048 1049 1050 1051 1056 1061 1063 1064 1065 1066 1068 1069 1074 1078
GameVisit	3	1 2 3
Group	2	Vegetable Control
Gender	2	Man Woman
SampleName	1	Spinach chopped

Dimensions	
Covariance Parameters	2
Columns in X	14
Columns in Z	0

Dimensions	
Subjects	34
Max Obs per Subject	3

Number of Observations	
Number of Observations Read	102
Number of Observations Used	102
Number of Observations Not Used	0

Iteration History

Iteration	Evaluations	-2 Res Log Like	Criterion
0	1	964.40829298	
1	1	928.20209111	0.00000000

Convergence criteria met.

Covariance Parameter Estimates

Cov Parm	Subject	Estimate
CS	ParticipantID	768.47
Residual		463.46

Fit Statistics	
-2 Res Log Likelihood	928.2
AIC (Smaller is Better)	932.2
AICC (Smaller is Better)	932.3
BIC (Smaller is Better)	935.3

Null Model Likelihood Ratio Test

DF	Chi-Square	Pr > ChiSq
1	36.21	<.0001

Solution for Fixed Effects

Effect	Group	Gender	GameVisit	Estimate	Standard Error	DF	t Value	Pr > |t|
Intercept				-4.7454	9.7935	47.1	-0.48	0.6302
GameVisit			1	-1.5000	7.6113	64	-0.20	0.8444
GameVisit			2	-3.8750	7.6113	64	-0.51	0.6124
GameVisit			3	0	.	.	.	.
Group	Vegetable			11.1926	12.1892	51.8	0.92	0.3628
Group	Control			0	.	.	.	.
GameVisit*Group	Vegetable		1	-10.6667	10.4608	64	-1.02	0.3117
GameVisit*Group	Control		1	0	.	.	.	.
GameVisit*Group	Vegetable		2	-8.7917	10.4608	64	-0.84	0.4038
GameVisit*Group	Control		2	0	.	.	.	.
GameVisit*Group	Vegetable		3	0	.	.	.	.
GameVisit*Group	Control		3	0	.	.	.	.

Solution for Fixed Effects								
Effect	Group	Gender	GameVisit	Estimate	Standard Error	DF	t Value	Pr > |t|
Gender		Man		-2.0121	11.5983	31	-0.17	0.8634
Gender		Woman		0	.	.	.	.

Type 3 Tests of Fixed Effects				
Effect	Num DF	Den DF	F Value	Pr > F
GameVisit	2	64	1.43	0.2474
Group	1	31	0.20	0.6598
GameVisit*Group	2	64	0.59	0.5558
Gender	1	31	0.03	0.8634

Least Squares Means Estimate						
Effect	Label	Estimate	Standard Error	DF	t Value	Pr > |t|
GameVisit*Group	Control, GV3-GV1	1.5000	7.6113	64	0.20	0.8444

Least Squares Means Estimate						
Effect	Label	Estimate	Standard Error	DF	t Value	Pr > |t|
GameVisit*Group	Control, GV2-GV1	-2.3750	7.6113	64	-0.31	0.7560

Least Squares Means Estimate						
Effect	Label	Estimate	Standard Error	DF	t Value	Pr > |t|
GameVisit*Group	Veg, GV3-GV1	12.1667	7.1760	64	1.70	0.0948

Least Squares Means Estimate						
Effect	Label	Estimate	Standard Error	DF	t Value	Pr > |t|
GameVisit*Group	Veg, GV2-GV1	-0.5000	7.1760	64	-0.07	0.9447

Least Squares Means Estimate						
Effect	Label	Estimate	Standard Error	DF	t Value	Pr > |t|
GameVisit*Group	Veg - Control, GV1	0.5259	12.1892	51.8	0.04	0.9658

Least Squares Means Estimate						
Effect	Label	Estimate	Standard Error	DF	t Value	Pr > |t|
GameVisit*Group	Veg - Control, GV2	2.4009	12.1892	51.8	0.20	0.8446

Least Squares Means Estimate						
Effect	Label	Estimate	Standard Error	DF	t Value	Pr > |t|
GameVisit*Group	Veg - Control, GV3	11.1926	12.1892	51.8	0.92	0.3628

Least Squares Means							
Effect	Group	GameVisit	Estimate	Standard Error	DF	t Value	Pr > |t|
Group	Vegetable		-2.8367	7.8521	31	-0.36	0.7203
Group	Control		-7.5432	7.7322	31	-0.98	0.3368
GameVisit		1	-6.9886	6.4665	48.9	-1.08	0.2851

Least Squares Means							
Effect	Group	GameVisit	Estimate	Standard Error	DF	t Value	Pr > |t|
GameVisit		2	-8.4261	6.4665	48.9	-1.30	0.1987
GameVisit		3	-0.1552	6.4665	48.9	-0.02	0.9809
GameVisit*Group	Vegetable	1	-6.7256	8.8781	48.8	-0.76	0.4524
GameVisit*Group	Control	1	-7.2515	8.8937	51.6	-0.82	0.4186
GameVisit*Group	Vegetable	2	-7.2256	8.8781	48.8	-0.81	0.4197
GameVisit*Group	Control	2	-9.6265	8.8937	51.6	-1.08	0.2841
GameVisit*Group	Vegetable	3	5.4411	8.8781	48.8	0.61	0.5428
GameVisit*Group	Control	3	-5.7515	8.8937	51.6	-0.65	0.5207

Differences of Least Squares Means									
Effect	Group	GameVisit	_Group	_GameVisit	Estimate	Standard Error	DF	t Value	Pr > |t|
Group	Vegetable		Control		4.7065	10.5878	31	0.44	0.6598
GameVisit		1		2	1.4375	5.2304	64	0.27	0.7843
GameVisit		1		3	-6.8333	5.2304	64	-1.31	0.1961
GameVisit		2		3	-8.2708	5.2304	64	-1.58	0.1187
GameVisit*Group	Vegetable	1	Control	1	0.5259	12.1892	51.8	0.04	0.9658
GameVisit*Group	Vegetable	1	Vegetable	2	0.5000	7.1760	64	0.07	0.9447
GameVisit*Group	Vegetable	1	Control	2	2.9009	12.1892	51.8	0.24	0.8128
GameVisit*Group	Vegetable	1	Vegetable	3	-12.1667	7.1760	64	-1.70	0.0948
GameVisit*Group	Vegetable	1	Control	3	-0.9741	12.1892	51.8	-0.08	0.9366
GameVisit*Group	Control	1	Vegetable	2	-0.02592	12.1892	51.8	-0.00	0.9983
GameVisit*Group	Control	1	Control	2	2.3750	7.6113	64	0.31	0.7560
GameVisit*Group	Control	1	Vegetable	3	-12.6926	12.1892	51.8	-1.04	0.3026
GameVisit*Group	Control	1	Control	3	-1.5000	7.6113	64	-0.20	0.8444
GameVisit*Group	Vegetable	2	Control	2	2.4009	12.1892	51.8	0.20	0.8446
GameVisit*Group	Vegetable	2	Vegetable	3	-12.6667	7.1760	64	-1.77	0.0823
GameVisit*Group	Vegetable	2	Control	3	-1.4741	12.1892	51.8	-0.12	0.9042
GameVisit*Group	Control	2	Vegetable	3	-15.0676	12.1892	51.8	-1.24	0.2220
GameVisit*Group	Control	2	Control	3	-3.8750	7.6113	64	-0.51	0.6124
GameVisit*Group	Vegetable	3	Control	3	11.1926	12.1892	51.8	0.92	0.3628

OSF planned analysis: Liking

The Mixed Procedure

SampleName=Spinach puree

Model Information	
Data Set	WORK.GAMEVISITS
Dependent Variable	Liking
Covariance Structure	Compound Symmetry
Subject Effect	ParticipantID

Model Information	
Estimation Method	REML
Residual Variance Method	Profile
Fixed Effects SE Method	Kenward-Roger
Degrees of Freedom Method	Kenward-Roger

Class Level Information		
Class	Levels	Values
ParticipantID	34	1001 1002 1005 1006 1009 1010 1014 1016 1019 1020 1024 1025 1027 1029 1030 1034 1035 1041 1045 1046 1048 1049 1050 1051 1056 1061 1063 1064 1065 1066 1068 1069 1074 1078
GameVisit	3	1 2 3
Group	2	Vegetable Control
Gender	2	Man Woman
SampleName	1	Spinach puree

Dimensions	
Covariance Parameters	2
Columns in X	14
Columns in Z	0
Subjects	34
Max Obs per Subject	3

Number of Observations	
Number of Observations Read	102
Number of Observations Used	102
Number of Observations Not Used	0

Iteration History			
Iteration	Evaluations	-2 Res Log Like	Criterion
0	1	914.55449668	
1	1	897.61433347	0.00000000

Convergence criteria met.

Covariance Parameter Estimates		
Cov Parm	Subject	Estimate
CS	ParticipantID	322.10
Residual		404.03

Fit Statistics	
-2 Res Log Likelihood	897.6
AIC (Smaller is Better)	901.6
AICC (Smaller is Better)	901.7
BIC (Smaller is Better)	904.7

Null Model Likelihood Ratio Test		
DF	Chi-Square	Pr > ChiSq

Null Model Likelihood Ratio Test		
DF	Chi-Square	Pr > ChiSq
1	16.94	<.0001

Solution for Fixed Effects								
Effect	Group	Gender	GameVisit	Estimate	Standard Error	DF	t Value	Pr > |t|
Intercept				-15.4137	7.3990	59	-2.08	0.0416
GameVisit			1	7.9375	7.1066	64	1.12	0.2682
GameVisit			2	4.0000	7.1066	64	0.56	0.5755
GameVisit			3	0	.	.	.	.
Group	Vegetable			8.0204	9.3422	66.2	0.86	0.3937
Group	Control			0	.	.	.	.
GameVisit*Group	Vegetable		1	-8.6597	9.7671	64	-0.89	0.3786
GameVisit*Group	Control		1	0	.	.	.	.
GameVisit*Group	Vegetable		2	-9.1667	9.7671	64	-0.94	0.3515
GameVisit*Group	Control		2	0	.	.	.	.
GameVisit*Group	Vegetable		3	0	.	.	.	.
GameVisit*Group	Control		3	0	.	.	.	.
Gender		Man		-0.2301	8.1593	31	-0.03	0.9777
Gender		Woman		0	.	.	.	.

Type 3 Tests of Fixed Effects				
Effect	Num DF	Den DF	F Value	Pr > F
GameVisit	2	64	0.43	0.6510
Group	1	31	0.08	0.7821
GameVisit*Group	2	64	0.56	0.5759
Gender	1	31	0.00	0.9777

Least Squares Means Estimate						
Effect	Label	Estimate	Standard Error	DF	t Value	Pr > |t|
GameVisit*Group	Control, GV3-GV1	-7.9375	7.1066	64	-1.12	0.2682

Least Squares Means Estimate						
Effect	Label	Estimate	Standard Error	DF	t Value	Pr > |t|
GameVisit*Group	Control, GV2-GV1	-3.9375	7.1066	64	-0.55	0.5815

Least Squares Means Estimate						
Effect	Label	Estimate	Standard Error	DF	t Value	Pr > |t|
GameVisit*Group	Veg, GV3-GV1	0.7222	6.7002	64	0.11	0.9145

Least Squares Means Estimate						
Effect	Label	Estimate	Standard Error	DF	t Value	Pr > |t|
GameVisit*Group	Veg, GV2-GV1	-4.4444	6.7002	64	-0.66	0.5095

Least Squares Means Estimate						
**Effect**	**Label**	**Estimate**	**Standard Error**	**DF**	**t Value**	**Pr > |t|**
**GameVisit*Group**	**Veg - Control, GV1**	-0.6393	9.3422	66.19	-0.07	0.9456

Least Squares Means Estimate						
**Effect**	**Label**	**Estimate**	**Standard Error**	**DF**	**t Value**	**Pr > |t|**
**GameVisit*Group**	**Veg - Control, GV2**	-1.1463	9.3422	66.19	-0.12	0.9027

Least Squares Means Estimate						
**Effect**	**Label**	**Estimate**	**Standard Error**	**DF**	**t Value**	**Pr > |t|**
**GameVisit*Group**	**Veg - Control, GV3**	8.0204	9.3422	66.19	0.86	0.3937

Least Squares Means							
**Effect**	**Group**	**GameVisit**	**Estimate**	**Standard Error**	**DF**	**t Value**	**Pr > |t|**
**Group**	**Vegetable**		-9.4713	5.5239	31	-1.71	0.0964
**Group**	**Control**		-11.5496	5.4395	31	-2.12	0.0418
**GameVisit**		**1**	-7.9109	4.9123	61.7	-1.61	0.1124
**GameVisit**		**2**	-12.1019	4.9123	61.7	-2.46	0.0166
**GameVisit**		**3**	-11.5186	4.9123	61.7	-2.34	0.0223
**GameVisit*Group**	**Vegetable**	**1**	-8.2306	6.7437	61.7	-1.22	0.2269
**GameVisit*Group**	**Control**	**1**	-7.5913	6.8135	66	-1.11	0.2693
**GameVisit*Group**	**Vegetable**	**2**	-12.6750	6.7437	61.7	-1.88	0.0649
**GameVisit*Group**	**Control**	**2**	-11.5288	6.8135	66	-1.69	0.0954
**GameVisit*Group**	**Vegetable**	**3**	-7.5084	6.7437	61.7	-1.11	0.2699
**GameVisit*Group**	**Control**	**3**	-15.5288	6.8135	66	-2.28	0.0259

Differences of Least Squares Means									
**Effect**	**Group**	**GameVisit**	**_Group**	**_GameVisit**	**Estimate**	**Standard Error**	**DF**	**t Value**	**Pr > |t|**
**Group**	**Vegetable**		**Control**		2.0783	7.4484	31	0.28	0.7821
**GameVisit**		**1**		**2**	4.1910	4.8836	64	0.86	0.3940
**GameVisit**		**1**		**3**	3.6076	4.8836	64	0.74	0.4628
**GameVisit**		**2**		**3**	-0.5833	4.8836	64	-0.12	0.9053
**GameVisit*Group**	**Vegetable**	**1**	**Control**	**1**	-0.6393	9.3422	66.2	-0.07	0.9456
**GameVisit*Group**	**Vegetable**	**1**	**Vegetable**	**2**	4.4444	6.7002	64	0.66	0.5095
**GameVisit*Group**	**Vegetable**	**1**	**Control**	**2**	3.2982	9.3422	66.2	0.35	0.7252
**GameVisit*Group**	**Vegetable**	**1**	**Vegetable**	**3**	-0.7222	6.7002	64	-0.11	0.9145
**GameVisit*Group**	**Vegetable**	**1**	**Control**	**3**	7.2982	9.3422	66.2	0.78	0.4375
**GameVisit*Group**	**Control**	**1**	**Vegetable**	**2**	5.0838	9.3422	66.2	0.54	0.5882
**GameVisit*Group**	**Control**	**1**	**Control**	**2**	3.9375	7.1066	64	0.55	0.5815
**GameVisit*Group**	**Control**	**1**	**Vegetable**	**3**	-0.08290	9.3422	66.2	-0.01	0.9929
**GameVisit*Group**	**Control**	**1**	**Control**	**3**	7.9375	7.1066	64	1.12	0.2682
**GameVisit*Group**	**Vegetable**	**2**	**Control**	**2**	-1.1463	9.3422	66.2	-0.12	0.9027
**GameVisit*Group**	**Vegetable**	**2**	**Vegetable**	**3**	-5.1667	6.7002	64	-0.77	0.4435
**GameVisit*Group**	**Vegetable**	**2**	**Control**	**3**	2.8537	9.3422	66.2	0.31	0.7610

Differences of Least Squares Means									
Effect	Group	GameVisit	_Group	_GameVisit	Estimate	Standard Error	DF	t Value	Pr > |t|
GameVisit*Group	Control	2	Vegetable	3	-4.0204	9.3422	66.2	-0.43	0.6683
GameVisit*Group	Control	2	Control	3	4.0000	7.1066	64	0.56	0.5755
GameVisit*Group	Vegetable	3	Control	3	8.0204	9.3422	66.2	0.86	0.3937

In [5]:

```
%%SAS sas

proc sort data=gamevisits;
by samplename;
run;

title 'OSF planned analysis: Sweetness';
ods output diffs=diff2 tests3=tests2
SolutionF=sol2 lsmeans=means2 FitStatistics=fit2 lsmESTIMATEs =LikeEstim2;
proc mixed data= gamevisits;
class participantID gamevisit group (ref = 'Control') gender samplename;
by samplename;
model Sweetness = gamevisit group gamevisit*group gender/ ddfm=kr solution; *fit is slightly better wi
repeated/ subject = participantID type = cs;
lsmeans group/pdiff ADJDFE=ROW;
lsmeans gamevisit/pdiff ADJDFE=ROW;
lsmeans gamevisit*group/pdiff adjdfe=row;
lsmestimate gamevisit*group 'Control, GV3-GV1' 0 -1 0 0 0 1 ;
lsmestimate gamevisit*group 'Control, GV2-GV1' 0 -1 0 1 0 0 ;
lsmestimate gamevisit*group 'Veg, GV3-GV1' -1 0 0 0 1 0 ;
lsmestimate gamevisit*group 'Veg, GV2-GV1' -1 0 1 0 0 0 ;
lsmestimate gamevisit*group 'Veg - Control, GV1' 1 -1 0 0 0 0 ;
lsmestimate gamevisit*group 'Veg - Control, GV2' 0 0 1 -1 0 0 ;
lsmestimate gamevisit*group 'Veg - Control, GV3' 0 0 0 0 1 -1 ;
run;
```

Out[5]:

OSF planned analysis: Sweetness

The Mixed Procedure

SampleName=Asparagus chopped

Model Information	
Data Set	WORK.GAMEVISITS
Dependent Variable	Sweetness
Covariance Structure	Compound Symmetry
Subject Effect	ParticipantID
Estimation Method	REML
Residual Variance Method	Profile
Fixed Effects SE Method	Kenward-Roger
Degrees of Freedom Method	Kenward-Roger

Class Level Information		
Class	Levels	Values
ParticipantID	34	1001 1002 1005 1006 1009 1010 1014 1016 1019 1020 1024 1025 1027 1029 1030 1034 1035 1041 1045 1046 1048 1049 1050 1051 1056 1061 1063 1064 1065 1066 1068 1069 1074 1078
GameVisit	3	1 2 3
Group	2	Vegetable Control
Gender	2	Man Woman

Class Level Information		
Class	Levels	Values
SampleName	1	Asparagus chopped

Dimensions	
Covariance Parameters	2
Columns in X	14
Columns in Z	0
Subjects	34
Max Obs per Subject	3

Number of Observations	
Number of Observations Read	102
Number of Observations Used	102
Number of Observations Not Used	0

Iteration History			
Iteration	Evaluations	-2 Res Log Like	Criterion
0	1	830.07025521	
1	1	798.08085265	0.00000000

Convergence criteria met.

Covariance Parameter Estimates		
Cov Parm	Subject	Estimate
CS	ParticipantID	177.26
Residual		122.08

Fit Statistics	
-2 Res Log Likelihood	798.1
AIC (Smaller is Better)	802.1
AICC (Smaller is Better)	802.2
BIC (Smaller is Better)	805.1

Null Model Likelihood Ratio Test		
DF	Chi-Square	Pr > ChiSq
1	31.99	<.0001

Solution for Fixed Effects								
Effect	Group	Gender	GameVisit	Estimate	Standard Error	DF	t Value	Pr > |t|
Intercept				26.1523	4.8141	49	5.43	<.0001
GameVisit			1	-0.6250	3.9064	64	-0.16	0.8734
GameVisit			2	-1.5000	3.9064	64	-0.38	0.7023
GameVisit			3	0	.	.	.	.
Group	Vegetable			-4.6176	6.0067	54.1	-0.77	0.4454
Group	Control			0	.	.	.	.

Solution for Fixed Effects								
Effect	Group	Gender	GameVisit	Estimate	Standard Error	DF	t Value	Pr > |t|
GameVisit*Group	Vegetable		1	2.4583	5.3688	64	0.46	0.6486
GameVisit*Group	Control		1	0	.	.	.	.
GameVisit*Group	Vegetable		2	0.1111	5.3688	64	0.02	0.9836
GameVisit*Group	Control		2	0	.	.	.	.
GameVisit*Group	Vegetable		3	0	.	.	.	.
GameVisit*Group	Control		3	0	.	.	.	.
Gender		Man		-2.9062	5.6362	31	-0.52	0.6098
Gender		Woman		0	.	.	.	.

Type 3 Tests of Fixed Effects				
Effect	Num DF	Den DF	F Value	Pr > F
GameVisit	2	64	0.31	0.7363
Group	1	31	0.53	0.4703
GameVisit*Group	2	64	0.13	0.8751
Gender	1	31	0.27	0.6098

Least Squares Means Estimate						
Effect	Label	Estimate	Standard Error	DF	t Value	Pr > |t|
GameVisit*Group	Control, GV3-GV1	0.6250	3.9064	64	0.16	0.8734

Least Squares Means Estimate						
Effect	Label	Estimate	Standard Error	DF	t Value	Pr > |t|
GameVisit*Group	Control, GV2-GV1	-0.8750	3.9064	64	-0.22	0.8235

Least Squares Means Estimate						
Effect	Label	Estimate	Standard Error	DF	t Value	Pr > |t|
GameVisit*Group	Veg, GV3-GV1	-1.8333	3.6830	64	-0.50	0.6203

Least Squares Means Estimate						
Effect	Label	Estimate	Standard Error	DF	t Value	Pr > |t|
GameVisit*Group	Veg, GV2-GV1	-3.2222	3.6830	64	-0.87	0.3849

Least Squares Means Estimate						
Effect	Label	Estimate	Standard Error	DF	t Value	Pr > |t|
GameVisit*Group	Veg - Control, GV1	-2.1593	6.0067	54.13	-0.36	0.7206

Least Squares Means Estimate						
Effect	Label	Estimate	Standard Error	DF	t Value	Pr > |t|
GameVisit*Group	Veg - Control, GV2	-4.5065	6.0067	54.13	-0.75	0.4564

Least Squares Means Estimate						
Effect	Label	Estimate	Standard Error	DF	t Value	Pr > |t|
GameVisit*Group	Veg - Control, GV3	-4.6176	6.0067	54.13	-0.77	0.4454

Least Squares Means							
Effect	Group	GameVisit	Estimate	Standard Error	DF	t Value	Pr > |t|
Group	Vegetable		20.2298	3.8157	31	5.30	<.0001
Group	Control		23.9909	3.7575	31	6.38	<.0001
GameVisit		1	22.9946	3.1817	50.9	7.23	<.0001
GameVisit		2	20.9460	3.1817	50.9	6.58	<.0001
GameVisit		3	22.3904	3.1817	50.9	7.04	<.0001
GameVisit*Group	Vegetable	1	21.9149	4.3682	50.9	5.02	<.0001
GameVisit*Group	Control	1	24.0742	4.3824	54	5.49	<.0001
GameVisit*Group	Vegetable	2	18.6927	4.3682	50.9	4.28	<.0001
GameVisit*Group	Control	2	23.1992	4.3824	54	5.29	<.0001
GameVisit*Group	Vegetable	3	20.0816	4.3682	50.9	4.60	<.0001
GameVisit*Group	Control	3	24.6992	4.3824	54	5.64	<.0001

Differences of Least Squares Means									
Effect	Group	GameVisit	_Group	_GameVisit	Estimate	Standard Error	DF	t Value	Pr > |t|
Group	Vegetable		Control		-3.7611	5.1451	31	-0.73	0.4703
GameVisit		1		2	2.0486	2.6844	64	0.76	0.4482
GameVisit		1		3	0.6042	2.6844	64	0.23	0.8226
GameVisit		2		3	-1.4444	2.6844	64	-0.54	0.5924
GameVisit*Group	Vegetable	1	Control	1	-2.1593	6.0067	54.1	-0.36	0.7206
GameVisit*Group	Vegetable	1	Vegetable	2	3.2222	3.6830	64	0.87	0.3849
GameVisit*Group	Vegetable	1	Control	2	-1.2843	6.0067	54.1	-0.21	0.8315
GameVisit*Group	Vegetable	1	Vegetable	3	1.8333	3.6830	64	0.50	0.6203
GameVisit*Group	Vegetable	1	Control	3	-2.7843	6.0067	54.1	-0.46	0.6448
GameVisit*Group	Control	1	Vegetable	2	5.3815	6.0067	54.1	0.90	0.3743
GameVisit*Group	Control	1	Control	2	0.8750	3.9064	64	0.22	0.8235
GameVisit*Group	Control	1	Vegetable	3	3.9926	6.0067	54.1	0.66	0.5091
GameVisit*Group	Control	1	Control	3	-0.6250	3.9064	64	-0.16	0.8734
GameVisit*Group	Vegetable	2	Control	2	-4.5065	6.0067	54.1	-0.75	0.4564
GameVisit*Group	Vegetable	2	Vegetable	3	-1.3889	3.6830	64	-0.38	0.7073
GameVisit*Group	Vegetable	2	Control	3	-6.0065	6.0067	54.1	-1.00	0.3218
GameVisit*Group	Control	2	Vegetable	3	3.1176	6.0067	54.1	0.52	0.6059
GameVisit*Group	Control	2	Control	3	-1.5000	3.9064	64	-0.38	0.7023
GameVisit*Group	Vegetable	3	Control	3	-4.6176	6.0067	54.1	-0.77	0.4454

OSF planned analysis: Sweetness

The Mixed Procedure

SampleName=Asparagus puree

Model Information	
Data Set	WORK.GAMEVISITS

Model Information	
Dependent Variable	Sweetness
Covariance Structure	Compound Symmetry
Subject Effect	ParticipantID
Estimation Method	REML
Residual Variance Method	Profile
Fixed Effects SE Method	Kenward-Roger
Degrees of Freedom Method	Kenward-Roger

Class Level Information		
Class	Levels	Values
ParticipantID	34	1001 1002 1005 1006 1009 1010 1014 1016 1019 1020 1024 1025 1027 1029 1030 1034 1035 1041 1045 1046 1048 1049 1050 1051 1056 1061 1063 1064 1065 1066 1068 1069 1074 1078
GameVisit	3	1 2 3
Group	2	Vegetable Control
Gender	2	Man Woman
SampleName	1	Asparagus puree

Dimensions	
Covariance Parameters	2
Columns in X	14
Columns in Z	0
Subjects	34
Max Obs per Subject	3

Number of Observations	
Number of Observations Read	102
Number of Observations Used	102
Number of Observations Not Used	0

Iteration History			
Iteration	Evaluations	-2 Res Log Like	Criterion
0	1	866.32281032	
1	1	821.59204303	0.00000000

Convergence criteria met.

Covariance Parameter Estimates		
Cov Parm	Subject	Estimate
CS	ParticipantID	298.16
Residual		141.08

Fit Statistics	
-2 Res Log Likelihood	821.6
AIC (Smaller is Better)	825.6
AICC (Smaller is Better)	825.7

Fit Statistics	
BIC (Smaller is Better)	828.6

Null Model Likelihood Ratio Test		
DF	Chi-Square	Pr > ChiSq
1	44.73	<.0001

Solution for Fixed Effects								
Effect	Group	Gender	GameVisit	Estimate	Standard Error	DF	t Value	Pr > |t|
Intercept				25.1199	5.8760	44.1	4.28	0.0001
GameVisit			1	3.6875	4.1994	64	0.88	0.3832
GameVisit			2	8.6875	4.1994	64	2.07	0.0426
GameVisit			3	0	.	.	.	.
Group	Vegetable			3.1363	7.2821	48	0.43	0.6686
Group	Control			0	.	.	.	.
GameVisit*Group	Vegetable		1	-6.1319	5.7715	64	-1.06	0.2920
GameVisit*Group	Control		1	0	.	.	.	.
GameVisit*Group	Vegetable		2	-13.9653	5.7715	64	-2.42	0.0184
GameVisit*Group	Control		2	0	.	.	.	.
GameVisit*Group	Vegetable		3	0	.	.	.	.
GameVisit*Group	Control		3	0	.	.	.	.
Gender		Man		-3.6532	7.0930	31	-0.52	0.6102
Gender		Woman		0	.	.	.	.

Type 3 Tests of Fixed Effects				
Effect	Num DF	Den DF	F Value	Pr > F
GameVisit	2	64	0.18	0.8367
Group	1	31	0.30	0.5861
GameVisit*Group	2	64	2.94	0.0599
Gender	1	31	0.27	0.6102

Least Squares Means Estimate						
Effect	Label	Estimate	Standard Error	DF	t Value	Pr > |t|
GameVisit*Group	Control, GV3-GV1	-3.6875	4.1994	64	-0.88	0.3832

Least Squares Means Estimate						
Effect	Label	Estimate	Standard Error	DF	t Value	Pr > |t|
GameVisit*Group	Control, GV2-GV1	5.0000	4.1994	64	1.19	0.2382

Least Squares Means Estimate						
Effect	Label	Estimate	Standard Error	DF	t Value	Pr > |t|
GameVisit*Group	Veg, GV3-GV1	2.4444	3.9592	64	0.62	0.5392

Least Squares Means Estimate						
Effect	Label	Estimate	Standard Error	DF	t Value	Pr > |t|

Least Squares Means Estimate						
Effect	Label	Estimate	Standard Error	DF	t Value	Pr > |t|
GameVisit*Group	Veg, GV2-GV1	-2.8333	3.9592	64	-0.72	0.4768

Least Squares Means Estimate						
Effect	Label	Estimate	Standard Error	DF	t Value	Pr > |t|
GameVisit*Group	Veg - Control, GV1	-2.9956	7.2821	47.97	-0.41	0.6826

Least Squares Means Estimate						
Effect	Label	Estimate	Standard Error	DF	t Value	Pr > |t|
GameVisit*Group	Veg - Control, GV2	-10.8290	7.2821	47.97	-1.49	0.1435

Least Squares Means Estimate						
Effect	Label	Estimate	Standard Error	DF	t Value	Pr > |t|
GameVisit*Group	Veg - Control, GV3	3.1363	7.2821	47.97	0.43	0.6686

Least Squares Means							
Effect	Group	GameVisit	Estimate	Standard Error	DF	t Value	Pr > |t|
Group	Vegetable		23.8556	4.8020	31	4.97	<.0001
Group	Control		27.4184	4.7286	31	5.80	<.0001
GameVisit		1	25.4830	3.8735	45.5	6.58	<.0001
GameVisit		2	26.5664	3.8735	45.5	6.86	<.0001
GameVisit		3	24.8615	3.8735	45.5	6.42	<.0001
GameVisit*Group	Vegetable	1	23.9852	5.3183	45.5	4.51	<.0001
GameVisit*Group	Control	1	26.9809	5.3140	47.8	5.08	<.0001
GameVisit*Group	Vegetable	2	21.1519	5.3183	45.5	3.98	0.0002
GameVisit*Group	Control	2	31.9809	5.3140	47.8	6.02	<.0001
GameVisit*Group	Vegetable	3	26.4297	5.3183	45.5	4.97	<.0001
GameVisit*Group	Control	3	23.2934	5.3140	47.8	4.38	<.0001

Differences of Least Squares Means									
Effect	Group	GameVisit	_Group	_GameVisit	Estimate	Standard Error	DF	t Value	Pr > |t|
Group	Vegetable		Control		-3.5628	6.4750	31	-0.55	0.5861
GameVisit		1		2	-1.0833	2.8858	64	-0.38	0.7086
GameVisit		1		3	0.6215	2.8858	64	0.22	0.8302
GameVisit		2		3	1.7049	2.8858	64	0.59	0.5567
GameVisit*Group	Vegetable	1	Control	1	-2.9956	7.2821	48	-0.41	0.6826
GameVisit*Group	Vegetable	1	Vegetable	2	2.8333	3.9592	64	0.72	0.4768
GameVisit*Group	Vegetable	1	Control	2	-7.9956	7.2821	48	-1.10	0.2777
GameVisit*Group	Vegetable	1	Vegetable	3	-2.4444	3.9592	64	-0.62	0.5392
GameVisit*Group	Vegetable	1	Control	3	0.6919	7.2821	48	0.10	0.9247
GameVisit*Group	Control	1	Vegetable	2	5.8290	7.2821	48	0.80	0.4274
GameVisit*Group	Control	1	Control	2	-5.0000	4.1994	64	-1.19	0.2382
GameVisit*Group	Control	1	Vegetable	3	0.5512	7.2821	48	0.08	0.9400

Differences of Least Squares Means									
Effect	Group	GameVisit	_Group	_GameVisit	Estimate	Standard Error	DF	t Value	Pr > |t|
GameVisit*Group	Control	1	Control	3	3.6875	4.1994	64	0.88	0.3832
GameVisit*Group	Vegetable	2	Control	2	-10.8290	7.2821	48	-1.49	0.1435
GameVisit*Group	Vegetable	2	Vegetable	3	-5.2778	3.9592	64	-1.33	0.1872
GameVisit*Group	Vegetable	2	Control	3	-2.1415	7.2821	48	-0.29	0.7700
GameVisit*Group	Control	2	Vegetable	3	5.5512	7.2821	48	0.76	0.4496
GameVisit*Group	Control	2	Control	3	8.6875	4.1994	64	2.07	0.0426
GameVisit*Group	Vegetable	3	Control	3	3.1363	7.2821	48	0.43	0.6686

OSF planned analysis: Sweetness

The Mixed Procedure

SampleName=Beef puree

Model Information	
Data Set	WORK.GAMEVISITS
Dependent Variable	Sweetness
Covariance Structure	Compound Symmetry
Subject Effect	ParticipantID
Estimation Method	REML
Residual Variance Method	Profile
Fixed Effects SE Method	Kenward-Roger
Degrees of Freedom Method	Kenward-Roger

Class Level Information		
Class	Levels	Values
ParticipantID	34	1001 1002 1005 1006 1009 1010 1014 1016 1019 1020 1024 1025 1027 1029 1030 1034 1035 1041 1045 1046 1048 1049 1050 1051 1056 1061 1063 1064 1065 1066 1068 1069 1074 1078
GameVisit	3	1 2 3
Group	2	Vegetable Control
Gender	2	Man Woman
SampleName	1	Beef puree

Dimensions	
Covariance Parameters	2
Columns in X	14
Columns in Z	0
Subjects	34
Max Obs per Subject	3

Number of Observations	
Number of Observations Read	102
Number of Observations Used	102

Number of Observations	
Number of Observations Not Used	0

Iteration History			
Iteration	Evaluations	-2 Res Log Like	Criterion
0	1	844.81658663	
1	1	780.26404904	0.00000000

Convergence criteria met.

Covariance Parameter Estimates		
Cov Parm	Subject	Estimate
CS	ParticipantID	271.54
Residual		79.4195

Fit Statistics	
-2 Res Log Likelihood	780.3
AIC (Smaller is Better)	784.3
AICC (Smaller is Better)	784.4
BIC (Smaller is Better)	787.3

Null Model Likelihood Ratio Test		
DF	Chi-Square	Pr > ChiSq
1	64.55	<.0001

Solution for Fixed Effects								
Effect	Group	Gender	GameVisit	Estimate	Standard Error	DF	t Value	Pr > |t|
Intercept				16.2829	5.2956	39.5	3.07	0.0038
GameVisit			1	-4.9375	3.1508	64	-1.57	0.1220
GameVisit			2	-0.9375	3.1508	64	-0.30	0.7670
GameVisit			3	0	.	.	.	.
Group	Vegetable			2.4773	6.5151	42	0.38	0.7057
Group	Control			0	.	.	.	.
GameVisit*Group	Vegetable		1	0.3819	4.3303	64	0.09	0.9300
GameVisit*Group	Control		1	0	.	.	.	.
GameVisit*Group	Vegetable		2	-1.8403	4.3303	64	-0.42	0.6723
GameVisit*Group	Control		2	0	.	.	.	.
GameVisit*Group	Vegetable		3	0	.	.	.	.
GameVisit*Group	Control		3	0	.	.	.	.
Gender		Man		-0.4211	6.5906	31	-0.06	0.9495
Gender		Woman		0	.	.	.	.

Type 3 Tests of Fixed Effects				
Effect	Num DF	Den DF	F Value	Pr > F
GameVisit	2	64	2.44	0.0952

Type 3 Tests of Fixed Effects				
Effect	Num DF	Den DF	F Value	Pr > F
Group	1	31	0.11	0.7429
GameVisit*Group	2	64	0.15	0.8605
Gender	1	31	0.00	0.9495

Least Squares Means Estimate						
Effect	Label	Estimate	Standard Error	DF	t Value	Pr > |t|
GameVisit*Group	Control, GV3-GV1	4.9375	3.1508	64	1.57	0.1220

Least Squares Means Estimate						
Effect	Label	Estimate	Standard Error	DF	t Value	Pr > |t|
GameVisit*Group	Control, GV2-GV1	4.0000	3.1508	64	1.27	0.2089

Least Squares Means Estimate						
Effect	Label	Estimate	Standard Error	DF	t Value	Pr > |t|
GameVisit*Group	Veg, GV3-GV1	4.5556	2.9706	64	1.53	0.1301

Least Squares Means Estimate						
Effect	Label	Estimate	Standard Error	DF	t Value	Pr > |t|
GameVisit*Group	Veg, GV2-GV1	1.7778	2.9706	64	0.60	0.5516

Least Squares Means Estimate						
Effect	Label	Estimate	Standard Error	DF	t Value	Pr > |t|
GameVisit*Group	Veg - Control, GV1	2.8593	6.5151	42.02	0.44	0.6630

Least Squares Means Estimate						
Effect	Label	Estimate	Standard Error	DF	t Value	Pr > |t|
GameVisit*Group	Veg - Control, GV2	0.6371	6.5151	42.02	0.10	0.9226

Least Squares Means Estimate						
Effect	Label	Estimate	Standard Error	DF	t Value	Pr > |t|
GameVisit*Group	Veg - Control, GV3	2.4773	6.5151	42.02	0.38	0.7057

Least Squares Means							
Effect	Group	GameVisit	Estimate	Standard Error	DF	t Value	Pr > |t|
Group	Vegetable		16.1053	4.4618	31	3.61	0.0011
Group	Control		14.1140	4.3937	31	3.21	0.0031
GameVisit		1	12.5645	3.4814	40.4	3.61	0.0008
GameVisit		2	15.4534	3.4814	40.4	4.44	<.0001
GameVisit		3	17.3110	3.4814	40.4	4.97	<.0001
GameVisit*Group	Vegetable	1	13.9942	4.7801	40.4	2.93	0.0056
GameVisit*Group	Control	1	11.1349	4.7554	41.9	2.34	0.0240
GameVisit*Group	Vegetable	2	15.7719	4.7801	40.4	3.30	0.0020
GameVisit*Group	Control	2	15.1349	4.7554	41.9	3.18	0.0027

Least Squares Means							
**Effect**	**Group**	**GameVisit**	**Estimate**	**Standard Error**	**DF**	**t Value**	**Pr > |t|**
**GameVisit*Group**	**Vegetable**	**3**	18.5497	4.7801	40.4	3.88	0.0004
**GameVisit*Group**	**Control**	**3**	16.0724	4.7554	41.9	3.38	0.0016

Differences of Least Squares Means									
**Effect**	**Group**	**GameVisit**	**_Group**	**_GameVisit**	**Estimate**	**Standard Error**	**DF**	**t Value**	**Pr > |t|**
**Group**	**Vegetable**		**Control**		1.9912	6.0164	31	0.33	0.7429
**GameVisit**		**1**		**2**	-2.8889	2.1652	64	-1.33	0.1868
**GameVisit**		**1**		**3**	-4.7465	2.1652	64	-2.19	0.0320
**GameVisit**		**2**		**3**	-1.8576	2.1652	64	-0.86	0.3941
**GameVisit*Group**	**Vegetable**	**1**	**Control**	**1**	2.8593	6.5151	42	0.44	0.6630
**GameVisit*Group**	**Vegetable**	**1**	**Vegetable**	**2**	-1.7778	2.9706	64	-0.60	0.5516
**GameVisit*Group**	**Vegetable**	**1**	**Control**	**2**	-1.1407	6.5151	42	-0.18	0.8619
**GameVisit*Group**	**Vegetable**	**1**	**Vegetable**	**3**	-4.5556	2.9706	64	-1.53	0.1301
**GameVisit*Group**	**Vegetable**	**1**	**Control**	**3**	-2.0782	6.5151	42	-0.32	0.7513
**GameVisit*Group**	**Control**	**1**	**Vegetable**	**2**	-4.6371	6.5151	42	-0.71	0.4806
**GameVisit*Group**	**Control**	**1**	**Control**	**2**	-4.0000	3.1508	64	-1.27	0.2089
**GameVisit*Group**	**Control**	**1**	**Vegetable**	**3**	-7.4148	6.5151	42	-1.14	0.2615
**GameVisit*Group**	**Control**	**1**	**Control**	**3**	-4.9375	3.1508	64	-1.57	0.1220
**GameVisit*Group**	**Vegetable**	**2**	**Control**	**2**	0.6371	6.5151	42	0.10	0.9226
**GameVisit*Group**	**Vegetable**	**2**	**Vegetable**	**3**	-2.7778	2.9706	64	-0.94	0.3533
**GameVisit*Group**	**Vegetable**	**2**	**Control**	**3**	-0.3004	6.5151	42	-0.05	0.9634
**GameVisit*Group**	**Control**	**2**	**Vegetable**	**3**	-3.4148	6.5151	42	-0.52	0.6029
**GameVisit*Group**	**Control**	**2**	**Control**	**3**	-0.9375	3.1508	64	-0.30	0.7670
**GameVisit*Group**	**Vegetable**	**3**	**Control**	**3**	2.4773	6.5151	42	0.38	0.7057

OSF planned analysis: Sweetness

The Mixed Procedure

SampleName=Black bean puree

Model Information	
**Data Set**	WORK.GAMEVISITS
**Dependent Variable**	Sweetness
**Covariance Structure**	Compound Symmetry
**Subject Effect**	ParticipantID
**Estimation Method**	REML
**Residual Variance Method**	Profile
**Fixed Effects SE Method**	Kenward-Roger
**Degrees of Freedom Method**	Kenward-Roger

Class Level Information		
**Class**	**Levels**	**Values**

Class Level Information		
Class	Levels	Values
ParticipantID	34	1001 1002 1005 1006 1009 1010 1014 1016 1019 1020 1024 1025 1027 1029 1030 1034 1035 1041 1045 1046 1048 1049 1050 1051 1056 1061 1063 1064 1065 1066 1068 1069 1074 1078
GameVisit	3	1 2 3
Group	2	Vegetable Control
Gender	2	Man Woman
SampleName	1	Black bean puree

Dimensions	
Covariance Parameters	2
Columns in X	14
Columns in Z	0
Subjects	34
Max Obs per Subject	3

Number of Observations	
Number of Observations Read	102
Number of Observations Used	102
Number of Observations Not Used	0

Iteration History			
Iteration	Evaluations	-2 Res Log Like	Criterion
0	1	844.72129962	
1	1	807.20490843	0.00000000

Convergence criteria met.

Covariance Parameter Estimates		
Cov Parm	Subject	Estimate
CS	ParticipantID	221.26
Residual		128.30

Fit Statistics	
-2 Res Log Likelihood	807.2
AIC (Smaller is Better)	811.2
AICC (Smaller is Better)	811.3
BIC (Smaller is Better)	814.3

Null Model Likelihood Ratio Test		
DF	Chi-Square	Pr > ChiSq
1	37.52	<.0001

Solution for Fixed Effects								
Effect	Group	Gender	GameVisit	Estimate	Standard Error	DF	t Value	Pr > |t|
Intercept				24.4775	5.2210	46.6	4.69	<.0001

Solution for Fixed Effects								
Effect	Group	Gender	GameVisit	Estimate	Standard Error	DF	t Value	Pr > |t|
GameVisit			1	-6.8125	4.0046	64	-1.70	0.0938
GameVisit			2	-8.8750	4.0046	64	-2.22	0.0302
GameVisit			3	0	.	.	.	.
Group	Vegetable			-4.9168	6.4935	51.1	-0.76	0.4524
Group	Control			0	.	.	.	.
GameVisit*Group	Vegetable		1	4.4792	5.5039	64	0.81	0.4188
GameVisit*Group	Control		1	0	.	.	.	.
GameVisit*Group	Vegetable		2	8.2083	5.5039	64	1.49	0.1408
GameVisit*Group	Control		2	0	.	.	.	.
GameVisit*Group	Vegetable		3	0	.	.	.	.
GameVisit*Group	Control		3	0	.	.	.	.
Gender		Man		-0.2733	6.2034	31	-0.04	0.9651
Gender		Woman		0	.	.	.	.

Type 3 Tests of Fixed Effects				
Effect	Num DF	Den DF	F Value	Pr > F
GameVisit	2	64	1.92	0.1544
Group	1	31	0.01	0.9041
GameVisit*Group	2	64	1.12	0.3341
Gender	1	31	0.00	0.9651

Least Squares Means Estimate						
Effect	Label	Estimate	Standard Error	DF	t Value	Pr > |t|
GameVisit*Group	Control, GV3-GV1	6.8125	4.0046	64	1.70	0.0938

Least Squares Means Estimate						
Effect	Label	Estimate	Standard Error	DF	t Value	Pr > |t|
GameVisit*Group	Control, GV2-GV1	-2.0625	4.0046	64	-0.52	0.6083

Least Squares Means Estimate						
Effect	Label	Estimate	Standard Error	DF	t Value	Pr > |t|
GameVisit*Group	Veg, GV3-GV1	2.3333	3.7756	64	0.62	0.5388

Least Squares Means Estimate						
Effect	Label	Estimate	Standard Error	DF	t Value	Pr > |t|
GameVisit*Group	Veg, GV2-GV1	1.6667	3.7756	64	0.44	0.6604

Least Squares Means Estimate						
Effect	Label	Estimate	Standard Error	DF	t Value	Pr > |t|
GameVisit*Group	Veg - Control, GV1	-0.4376	6.4935	51.14	-0.07	0.9465

Least Squares Means Estimate						
Effect	Label	Estimate	Standard Error	DF	t Value	Pr > |t|

Least Squares Means Estimate						
Effect	Label	Estimate	Standard Error	DF	t Value	Pr > |t|
GameVisit*Group	Veg - Control, GV2	3.2916	6.4935	51.14	0.51	0.6144

Least Squares Means Estimate						
Effect	Label	Estimate	Standard Error	DF	t Value	Pr > |t|
GameVisit*Group	Veg - Control, GV3	-4.9168	6.4935	51.14	-0.76	0.4524

Least Squares Means							
Effect	Group	GameVisit	Estimate	Standard Error	DF	t Value	Pr > |t|
Group	Vegetable		18.4241	4.1997	31	4.39	0.0001
Group	Control		19.1117	4.1356	31	4.62	<.0001
GameVisit		1	17.3095	3.4464	48.3	5.02	<.0001
GameVisit		2	17.1116	3.4464	48.3	4.97	<.0001
GameVisit		3	21.8825	3.4464	48.3	6.35	<.0001
GameVisit*Group	Vegetable	1	17.0908	4.7317	48.3	3.61	0.0007
GameVisit*Group	Control	1	17.5283	4.7380	51	3.70	0.0005
GameVisit*Group	Vegetable	2	18.7574	4.7317	48.3	3.96	0.0002
GameVisit*Group	Control	2	15.4658	4.7380	51	3.26	0.0020
GameVisit*Group	Vegetable	3	19.4241	4.7317	48.3	4.11	0.0002
GameVisit*Group	Control	3	24.3408	4.7380	51	5.14	<.0001

Differences of Least Squares Means									
Effect	Group	GameVisit	_Group	_GameVisit	Estimate	Standard Error	DF	t Value	Pr > |t|
Group	Vegetable		Control		-0.6876	5.6629	31	-0.12	0.9041
GameVisit		1		2	0.1979	2.7519	64	0.07	0.9429
GameVisit		1		3	-4.5729	2.7519	64	-1.66	0.1015
GameVisit		2		3	-4.7708	2.7519	64	-1.73	0.0878
GameVisit*Group	Vegetable	1	Control	1	-0.4376	6.4935	51.1	-0.07	0.9465
GameVisit*Group	Vegetable	1	Vegetable	2	-1.6667	3.7756	64	-0.44	0.6604
GameVisit*Group	Vegetable	1	Control	2	1.6249	6.4935	51.1	0.25	0.8034
GameVisit*Group	Vegetable	1	Vegetable	3	-2.3333	3.7756	64	-0.62	0.5388
GameVisit*Group	Vegetable	1	Control	3	-7.2501	6.4935	51.1	-1.12	0.2694
GameVisit*Group	Control	1	Vegetable	2	-1.2291	6.4935	51.1	-0.19	0.8506
GameVisit*Group	Control	1	Control	2	2.0625	4.0046	64	0.52	0.6083
GameVisit*Group	Control	1	Vegetable	3	-1.8957	6.4935	51.1	-0.29	0.7715
GameVisit*Group	Control	1	Control	3	-6.8125	4.0046	64	-1.70	0.0938
GameVisit*Group	Vegetable	2	Control	2	3.2916	6.4935	51.1	0.51	0.6144
GameVisit*Group	Vegetable	2	Vegetable	3	-0.6667	3.7756	64	-0.18	0.8604
GameVisit*Group	Vegetable	2	Control	3	-5.5834	6.4935	51.1	-0.86	0.3939
GameVisit*Group	Control	2	Vegetable	3	-3.9582	6.4935	51.1	-0.61	0.5448
GameVisit*Group	Control	2	Control	3	-8.8750	4.0046	64	-2.22	0.0302
GameVisit*Group	Vegetable	3	Control	3	-4.9168	6.4935	51.1	-0.76	0.4524

OSF planned analysis: Sweetness

The Mixed Procedure

SampleName=Broccoli chopped

Model Information	
Data Set	WORK.GAMEVISITS
Dependent Variable	Sweetness
Covariance Structure	Compound Symmetry
Subject Effect	ParticipantID
Estimation Method	REML
Residual Variance Method	Profile
Fixed Effects SE Method	Kenward-Roger
Degrees of Freedom Method	Kenward-Roger

Class Level Information

Class	Levels	Values
ParticipantID	34	1001 1002 1005 1006 1009 1010 1014 1016 1019 1020 1024 1025 1027 1029 1030 1034 1035 1041 1045 1046 1048 1049 1050 1051 1056 1061 1063 1064 1065 1066 1068 1069 1074 1078
GameVisit	3	1 2 3
Group	2	Vegetable Control
Gender	2	Man Woman
SampleName	1	Broccoli chopped

Dimensions	
Covariance Parameters	2
Columns in X	14
Columns in Z	0
Subjects	34
Max Obs per Subject	3

Number of Observations	
Number of Observations Read	102
Number of Observations Used	102
Number of Observations Not Used	0

Iteration History

Iteration	Evaluations	-2 Res Log Like	Criterion
0	1	852.49117811	
1	1	816.09609725	0.00000000

Convergence criteria met.

Covariance Parameter Estimates

Cov Parm	Subject	Estimate
CS	ParticipantID	237.11

Covariance Parameter Estimates		
Cov Parm	Subject	Estimate
Residual		142.18

Fit Statistics	
-2 Res Log Likelihood	816.1
AIC (Smaller is Better)	820.1
AICC (Smaller is Better)	820.2
BIC (Smaller is Better)	823.1

Null Model Likelihood Ratio Test		
DF	Chi-Square	Pr > ChiSq
1	36.40	<.0001

Solution for Fixed Effects								
Effect	Group	Gender	GameVisit	Estimate	Standard Error	DF	t Value	Pr > |t|
Intercept				31.3421	5.4348	47.1	5.77	<.0001
GameVisit			1	-2.1875	4.2158	64	-0.52	0.6056
GameVisit			2	-0.3125	4.2158	64	-0.07	0.9411
GameVisit			3	0	.	.	.	.
Group	Vegetable			-2.5838	6.7635	51.7	-0.38	0.7040
Group	Control			0	.	.	.	.
GameVisit*Group	Vegetable		1	7.4653	5.7940	64	1.29	0.2022
GameVisit*Group	Control		1	0	.	.	.	.
GameVisit*Group	Vegetable		2	3.8125	5.7940	64	0.66	0.5129
GameVisit*Group	Control		2	0	.	.	.	.
GameVisit*Group	Vegetable		3	0	.	.	.	.
GameVisit*Group	Control		3	0	.	.	.	.
Gender		Man		-12.4123	6.4394	31	-1.93	0.0631
Gender		Woman		0	.	.	.	.

Type 3 Tests of Fixed Effects				
Effect	Num DF	Den DF	F Value	Pr > F
GameVisit	2	64	0.20	0.8227
Group	1	31	0.04	0.8428
GameVisit*Group	2	64	0.83	0.4406
Gender	1	31	3.72	0.0631

Least Squares Means Estimate						
Effect	Label	Estimate	Standard Error	DF	t Value	Pr > |t|
GameVisit*Group	Control, GV3-GV1	2.1875	4.2158	64	0.52	0.6056

Least Squares Means Estimate						
Effect	Label	Estimate	Standard Error	DF	t Value	Pr > |t|

Least Squares Means Estimate						
Effect	Label	Estimate	Standard Error	DF	t Value	Pr > |t|
GameVisit*Group	Control, GV2-GV1	1.8750	4.2158	64	0.44	0.6580

Least Squares Means Estimate						
Effect	Label	Estimate	Standard Error	DF	t Value	Pr > |t|
GameVisit*Group	Veg, GV3-GV1	-5.2778	3.9747	64	-1.33	0.1889

Least Squares Means Estimate						
Effect	Label	Estimate	Standard Error	DF	t Value	Pr > |t|
GameVisit*Group	Veg, GV2-GV1	-1.7778	3.9747	64	-0.45	0.6562

Least Squares Means Estimate						
Effect	Label	Estimate	Standard Error	DF	t Value	Pr > |t|
GameVisit*Group	Veg - Control, GV1	4.8815	6.7635	51.7	0.72	0.4737

Least Squares Means Estimate						
Effect	Label	Estimate	Standard Error	DF	t Value	Pr > |t|
GameVisit*Group	Veg - Control, GV2	1.2287	6.7635	51.7	0.18	0.8566

Least Squares Means Estimate						
Effect	Label	Estimate	Standard Error	DF	t Value	Pr > |t|
GameVisit*Group	Veg - Control, GV3	-2.5838	6.7635	51.7	-0.38	0.7040

Least Squares Means							
Effect	Group	GameVisit	Estimate	Standard Error	DF	t Value	Pr > |t|
Group	Vegetable		25.4781	4.3595	31	5.84	<.0001
Group	Control		24.3026	4.2929	31	5.66	<.0001
GameVisit		1	25.3892	3.5883	48.8	7.08	<.0001
GameVisit		2	25.4378	3.5883	48.8	7.09	<.0001
GameVisit		3	23.8441	3.5883	48.8	6.64	<.0001
GameVisit*Group	Vegetable	1	27.8299	4.9266	48.7	5.65	<.0001
GameVisit*Group	Control	1	22.9485	4.9349	51.6	4.65	<.0001
GameVisit*Group	Vegetable	2	26.0521	4.9266	48.7	5.29	<.0001
GameVisit*Group	Control	2	24.8235	4.9349	51.6	5.03	<.0001
GameVisit*Group	Vegetable	3	22.5521	4.9266	48.7	4.58	<.0001
GameVisit*Group	Control	3	25.1360	4.9349	51.6	5.09	<.0001

Differences of Least Squares Means									
Effect	Group	GameVisit	_Group	_GameVisit	Estimate	Standard Error	DF	t Value	Pr > |t|
Group	Vegetable		Control		1.1754	5.8783	31	0.20	0.8428
GameVisit		1		2	-0.04861	2.8970	64	-0.02	0.9867
GameVisit		1		3	1.5451	2.8970	64	0.53	0.5956
GameVisit		2		3	1.5938	2.8970	64	0.55	0.5841
GameVisit*Group	Vegetable	1	Control	1	4.8815	6.7635	51.7	0.72	0.4737

Differences of Least Squares Means									
Effect	Group	GameVisit	_Group	_GameVisit	Estimate	Standard Error	DF	t Value	Pr > abs(t)
GameVisit*Group	Vegetable	1	Vegetable	2	1.7778	3.9747	64	0.45	0.6562
GameVisit*Group	Vegetable	1	Control	2	3.0065	6.7635	51.7	0.44	0.6585
GameVisit*Group	Vegetable	1	Vegetable	3	5.2778	3.9747	64	1.33	0.1889
GameVisit*Group	Vegetable	1	Control	3	2.6940	6.7635	51.7	0.40	0.6920
GameVisit*Group	Control	1	Vegetable	2	-3.1037	6.7635	51.7	-0.46	0.6482
GameVisit*Group	Control	1	Control	2	-1.8750	4.2158	64	-0.44	0.6580
GameVisit*Group	Control	1	Vegetable	3	0.3963	6.7635	51.7	0.06	0.9535
GameVisit*Group	Control	1	Control	3	-2.1875	4.2158	64	-0.52	0.6056
GameVisit*Group	Vegetable	2	Control	2	1.2287	6.7635	51.7	0.18	0.8566
GameVisit*Group	Vegetable	2	Vegetable	3	3.5000	3.9747	64	0.88	0.3818
GameVisit*Group	Vegetable	2	Control	3	0.9162	6.7635	51.7	0.14	0.8928
GameVisit*Group	Control	2	Vegetable	3	2.2713	6.7635	51.7	0.34	0.7384
GameVisit*Group	Control	2	Control	3	-0.3125	4.2158	64	-0.07	0.9411
GameVisit*Group	Vegetable	3	Control	3	-2.5838	6.7635	51.7	-0.38	0.7040

OSF planned analysis: Sweetness

The Mixed Procedure

SampleName=Broccoli puree

Model Information	
Data Set	WORK.GAMEVISITS
Dependent Variable	Sweetness
Covariance Structure	Compound Symmetry
Subject Effect	ParticipantID
Estimation Method	REML
Residual Variance Method	Profile
Fixed Effects SE Method	Kenward-Roger
Degrees of Freedom Method	Kenward-Roger

Class Level Information		
Class	Levels	Values
ParticipantID	34	1001 1002 1005 1006 1009 1010 1014 1016 1019 1020 1024 1025 1027 1029 1030 1034 1035 1041 1045 1046 1048 1049 1050 1051 1056 1061 1063 1064 1065 1066 1068 1069 1074 1078
GameVisit	3	1 2 3
Group	2	Vegetable Control
Gender	2	Man Woman
SampleName	1	Broccoli puree

Dimensions	
Covariance Parameters	2
Columns in X	14
Columns in Z	0

Dimensions	
Subjects	34
Max Obs per Subject	3

Number of Observations	
Number of Observations Read	102
Number of Observations Used	102
Number of Observations Not Used	0

Iteration History			
Iteration	Evaluations	-2 Res Log Like	Criterion
0	1	868.70989499	
1	1	819.52796704	0.00000000

Convergence criteria met.

Covariance Parameter Estimates		
Cov Parm	Subject	Estimate
CS	ParticipantID	317.09
Residual		133.56

Fit Statistics	
-2 Res Log Likelihood	819.5
AIC (Smaller is Better)	823.5
AICC (Smaller is Better)	823.7
BIC (Smaller is Better)	826.6

Null Model Likelihood Ratio Test		
DF	Chi-Square	Pr > ChiSq
1	49.18	<.0001

Solution for Fixed Effects								
Effect	Group	Gender	GameVisit	Estimate	Standard Error	DF	t Value	Pr > |t|
Intercept				26.3287	5.9647	42.8	4.41	<.0001
GameVisit			1	-2.3750	4.0860	64	-0.58	0.5631
GameVisit			2	-2.0625	4.0860	64	-0.50	0.6154
GameVisit			3	0	.	.	.	.
Group	Vegetable			3.2179	7.3778	46.3	0.44	0.6647
Group	Control			0	.	.	.	.
GameVisit*Group	Vegetable		1	3.0417	5.6156	64	0.54	0.5899
GameVisit*Group	Control		1	0	.	.	.	.
GameVisit*Group	Vegetable		2	2.5069	5.6156	64	0.45	0.6568
GameVisit*Group	Control		2	0	.	.	.	.
GameVisit*Group	Vegetable		3	0	.	.	.	.
GameVisit*Group	Control		3	0	.	.	.	.

Solution for Fixed Effects								
Effect	Group	Gender	GameVisit	Estimate	Standard Error	DF	t Value	Pr > |t|
Gender		Man		-4.7099	7.2598	31	-0.65	0.5213
Gender		Woman		0	.	.	.	.

Type 3 Tests of Fixed Effects				
Effect	Num DF	Den DF	F Value	Pr > F
GameVisit	2	64	0.06	0.9431
Group	1	31	0.58	0.4503
GameVisit*Group	2	64	0.17	0.8464
Gender	1	31	0.42	0.5213

Least Squares Means Estimate						
Effect	Label	Estimate	Standard Error	DF	t Value	Pr > |t|
GameVisit*Group	Control, GV3-GV1	2.3750	4.0860	64	0.58	0.5631

Least Squares Means Estimate						
Effect	Label	Estimate	Standard Error	DF	t Value	Pr > |t|
GameVisit*Group	Control, GV2-GV1	0.3125	4.0860	64	0.08	0.9393

Least Squares Means Estimate						
Effect	Label	Estimate	Standard Error	DF	t Value	Pr > |t|
GameVisit*Group	Veg, GV3-GV1	-0.6667	3.8523	64	-0.17	0.8632

Least Squares Means Estimate						
Effect	Label	Estimate	Standard Error	DF	t Value	Pr > |t|
GameVisit*Group	Veg, GV2-GV1	-0.2222	3.8523	64	-0.06	0.9542

Least Squares Means Estimate						
Effect	Label	Estimate	Standard Error	DF	t Value	Pr > |t|
GameVisit*Group	Veg - Control, GV1	6.2596	7.3778	46.33	0.85	0.4006

Least Squares Means Estimate						
Effect	Label	Estimate	Standard Error	DF	t Value	Pr > |t|
GameVisit*Group	Veg - Control, GV2	5.7249	7.3778	46.33	0.78	0.4417

Least Squares Means Estimate						
Effect	Label	Estimate	Standard Error	DF	t Value	Pr > |t|
GameVisit*Group	Veg - Control, GV3	3.2179	7.3778	46.33	0.44	0.6647

Least Squares Means							
Effect	Group	GameVisit	Estimate	Standard Error	DF	t Value	Pr > |t|
Group	Vegetable		27.5621	4.9149	31	5.61	<.0001
Group	Control		22.4946	4.8399	31	4.65	<.0001
GameVisit		1	24.7286	3.9291	44.1	6.29	<.0001

Least Squares Means							
Effect	Group	GameVisit	Estimate	Standard Error	DF	t Value	Pr > |t|
GameVisit		2	24.7737	3.9291	44.1	6.31	<.0001
GameVisit		3	25.5827	3.9291	44.1	6.51	<.0001
GameVisit*Group	Vegetable	1	27.8584	5.3947	44.1	5.16	<.0001
GameVisit*Group	Control	1	21.5988	5.3842	46.2	4.01	0.0002
GameVisit*Group	Vegetable	2	27.6362	5.3947	44.1	5.12	<.0001
GameVisit*Group	Control	2	21.9113	5.3842	46.2	4.07	0.0002
GameVisit*Group	Vegetable	3	27.1917	5.3947	44.1	5.04	<.0001
GameVisit*Group	Control	3	23.9738	5.3842	46.2	4.45	<.0001

Differences of Least Squares Means									
Effect	Group	GameVisit	_Group	_GameVisit	Estimate	Standard Error	DF	t Value	Pr > |t|
Group	Vegetable		Control		5.0675	6.6272	31	0.76	0.4503
GameVisit		1		2	-0.04514	2.8078	64	-0.02	0.9872
GameVisit		1		3	-0.8542	2.8078	64	-0.30	0.7620
GameVisit		2		3	-0.8090	2.8078	64	-0.29	0.7742
GameVisit*Group	Vegetable	1	Control	1	6.2596	7.3778	46.3	0.85	0.4006
GameVisit*Group	Vegetable	1	Vegetable	2	0.2222	3.8523	64	0.06	0.9542
GameVisit*Group	Vegetable	1	Control	2	5.9471	7.3778	46.3	0.81	0.4243
GameVisit*Group	Vegetable	1	Vegetable	3	0.6667	3.8523	64	0.17	0.8632
GameVisit*Group	Vegetable	1	Control	3	3.8846	7.3778	46.3	0.53	0.6010
GameVisit*Group	Control	1	Vegetable	2	-6.0374	7.3778	46.3	-0.82	0.4174
GameVisit*Group	Control	1	Control	2	-0.3125	4.0860	64	-0.08	0.9393
GameVisit*Group	Control	1	Vegetable	3	-5.5929	7.3778	46.3	-0.76	0.4522
GameVisit*Group	Control	1	Control	3	-2.3750	4.0860	64	-0.58	0.5631
GameVisit*Group	Vegetable	2	Control	2	5.7249	7.3778	46.3	0.78	0.4417
GameVisit*Group	Vegetable	2	Vegetable	3	0.4444	3.8523	64	0.12	0.9085
GameVisit*Group	Vegetable	2	Control	3	3.6624	7.3778	46.3	0.50	0.6220
GameVisit*Group	Control	2	Vegetable	3	-5.2804	7.3778	46.3	-0.72	0.4778
GameVisit*Group	Control	2	Control	3	-2.0625	4.0860	64	-0.50	0.6154
GameVisit*Group	Vegetable	3	Control	3	3.2179	7.3778	46.3	0.44	0.6647

OSF planned analysis: Sweetness

The Mixed Procedure

SampleName=Chicken puree

Model Information	
Data Set	WORK.GAMEVISITS
Dependent Variable	Sweetness
Covariance Structure	Compound Symmetry
Subject Effect	ParticipantID

Model Information	
Estimation Method	REML
Residual Variance Method	Profile
Fixed Effects SE Method	Kenward-Roger
Degrees of Freedom Method	Kenward-Roger

Class Level Information		
Class	Levels	Values
ParticipantID	34	1001 1002 1005 1006 1009 1010 1014 1016 1019 1020 1024 1025 1027 1029 1030 1034 1035 1041 1045 1046 1048 1049 1050 1051 1056 1061 1063 1064 1065 1066 1068 1069 1074 1078
GameVisit	3	1 2 3
Group	2	Vegetable Control
Gender	2	Man Woman
SampleName	1	Chicken puree

Dimensions	
Covariance Parameters	2
Columns in X	14
Columns in Z	0
Subjects	34
Max Obs per Subject	3

Number of Observations	
Number of Observations Read	102
Number of Observations Used	102
Number of Observations Not Used	0

Iteration History			
Iteration	Evaluations	-2 Res Log Like	Criterion
0	1	818.36146816	
1	1	756.12611674	0.00000000

Convergence criteria met.

Covariance Parameter Estimates		
Cov Parm	Subject	Estimate
CS	ParticipantID	203.05
Residual		62.5582

Fit Statistics	
-2 Res Log Likelihood	756.1
AIC (Smaller is Better)	760.1
AICC (Smaller is Better)	760.3
BIC (Smaller is Better)	763.2

Null Model Likelihood Ratio Test		
DF	Chi-Square	Pr > ChiSq

Null Model Likelihood Ratio Test		
DF	Chi-Square	Pr > ChiSq
1	62.24	<.0001

Solution for Fixed Effects

Effect	Group	Gender	GameVisit	Estimate	Standard Error	DF	t Value	Pr > |t|
Intercept				12.5316	4.6032	39.9	2.72	0.0096
GameVisit			1	-2.3750	2.7964	64	-0.85	0.3989
GameVisit			2	1.8125	2.7964	64	0.65	0.5192
GameVisit			3	0	.	.	.	.
Group	Vegetable			1.0056	5.6673	42.6	0.18	0.8600
Group	Control			0	.	.	.	.
GameVisit*Group	Vegetable		1	3.6528	3.8433	64	0.95	0.3455
GameVisit*Group	Control		1	0	.	.	.	.
GameVisit*Group	Vegetable		2	-2.3681	3.8433	64	-0.62	0.5400
GameVisit*Group	Control		2	0	.	.	.	.
GameVisit*Group	Vegetable		3	0	.	.	.	.
GameVisit*Group	Control		3	0	.	.	.	.
Gender		Man		-0.4177	5.7125	31	-0.07	0.9422
Gender		Woman		0	.	.	.	.

Type 3 Tests of Fixed Effects

Effect	Num DF	Den DF	F Value	Pr > F
GameVisit	2	64	0.19	0.8292
Group	1	31	0.08	0.7852
GameVisit*Group	2	64	1.25	0.2946
Gender	1	31	0.01	0.9422

Least Squares Means Estimate

Effect	Label	Estimate	Standard Error	DF	t Value	Pr > |t|
GameVisit*Group	Control, GV3-GV1	2.3750	2.7964	64	0.85	0.3989

Least Squares Means Estimate

Effect	Label	Estimate	Standard Error	DF	t Value	Pr > |t|
GameVisit*Group	Control, GV2-GV1	4.1875	2.7964	64	1.50	0.1392

Least Squares Means Estimate

Effect	Label	Estimate	Standard Error	DF	t Value	Pr > |t|
GameVisit*Group	Veg, GV3-GV1	-1.2778	2.6365	64	-0.48	0.6296

Least Squares Means Estimate

Effect	Label	Estimate	Standard Error	DF	t Value	Pr > |t|
GameVisit*Group	Veg, GV2-GV1	-1.8333	2.6365	64	-0.70	0.4893

Least Squares Means Estimate						
Effect	Label	Estimate	Standard Error	DF	t Value	Pr > |t|
GameVisit*Group	Veg - Control, GV1	4.6584	5.6673	42.57	0.82	0.4157

Least Squares Means Estimate						
Effect	Label	Estimate	Standard Error	DF	t Value	Pr > |t|
GameVisit*Group	Veg - Control, GV2	-1.3624	5.6673	42.57	-0.24	0.8112

Least Squares Means Estimate						
Effect	Label	Estimate	Standard Error	DF	t Value	Pr > |t|
GameVisit*Group	Veg - Control, GV3	1.0056	5.6673	42.57	0.18	0.8600

Least Squares Means							
Effect	Group	GameVisit	Estimate	Standard Error	DF	t Value	Pr > |t|
Group	Vegetable		13.5692	3.8674	31	3.51	0.0014
Group	Control		12.1353	3.8084	31	3.19	0.0033
GameVisit		1	12.2770	3.0270	40.9	4.06	0.0002
GameVisit		2	13.4541	3.0270	40.9	4.44	<.0001
GameVisit		3	12.8256	3.0270	40.9	4.24	0.0001
GameVisit*Group	Vegetable	1	14.6062	4.1562	40.9	3.51	0.0011
GameVisit*Group	Control	1	9.9478	4.1365	42.5	2.40	0.0206
GameVisit*Group	Vegetable	2	12.7729	4.1562	40.9	3.07	0.0038
GameVisit*Group	Control	2	14.1353	4.1365	42.5	3.42	0.0014
GameVisit*Group	Vegetable	3	13.3284	4.1562	40.9	3.21	0.0026
GameVisit*Group	Control	3	12.3228	4.1365	42.5	2.98	0.0048

Differences of Least Squares Means									
Effect	Group	GameVisit	_Group	_GameVisit	Estimate	Standard Error	DF	t Value	Pr > |t|
Group	Vegetable		Control		1.4339	5.2148	31	0.27	0.7852
GameVisit		1		2	-1.1771	1.9216	64	-0.61	0.5423
GameVisit		1		3	-0.5486	1.9216	64	-0.29	0.7762
GameVisit		2		3	0.6285	1.9216	64	0.33	0.7447
GameVisit*Group	Vegetable	1	Control	1	4.6584	5.6673	42.6	0.82	0.4157
GameVisit*Group	Vegetable	1	Vegetable	2	1.8333	2.6365	64	0.70	0.4893
GameVisit*Group	Vegetable	1	Control	2	0.4709	5.6673	42.6	0.08	0.9342
GameVisit*Group	Vegetable	1	Vegetable	3	1.2778	2.6365	64	0.48	0.6296
GameVisit*Group	Vegetable	1	Control	3	2.2834	5.6673	42.6	0.40	0.6890
GameVisit*Group	Control	1	Vegetable	2	-2.8251	5.6673	42.6	-0.50	0.6207
GameVisit*Group	Control	1	Control	2	-4.1875	2.7964	64	-1.50	0.1392
GameVisit*Group	Control	1	Vegetable	3	-3.3806	5.6673	42.6	-0.60	0.5540
GameVisit*Group	Control	1	Control	3	-2.3750	2.7964	64	-0.85	0.3989
GameVisit*Group	Vegetable	2	Control	2	-1.3624	5.6673	42.6	-0.24	0.8112
GameVisit*Group	Vegetable	2	Vegetable	3	-0.5556	2.6365	64	-0.21	0.8338
GameVisit*Group	Vegetable	2	Control	3	0.4501	5.6673	42.6	0.08	0.9371

Differences of Least Squares Means									
Effect	Group	GameVisit	_Group	_GameVisit	Estimate	Standard Error	DF	t Value	Pr > |t|
GameVisit*Group	Control	2	Vegetable	3	0.8069	5.6673	42.6	0.14	0.8875
GameVisit*Group	Control	2	Control	3	1.8125	2.7964	64	0.65	0.5192
GameVisit*Group	Vegetable	3	Control	3	1.0056	5.6673	42.6	0.18	0.8600

OSF planned analysis: Sweetness

The Mixed Procedure

SampleName=Kale chopped

Model Information	
Data Set	WORK.GAMEVISITS
Dependent Variable	Sweetness
Covariance Structure	Compound Symmetry
Subject Effect	ParticipantID
Estimation Method	REML
Residual Variance Method	Profile
Fixed Effects SE Method	Kenward-Roger
Degrees of Freedom Method	Kenward-Roger

Class Level Information		
Class	Levels	Values
ParticipantID	34	1001 1002 1005 1006 1009 1010 1014 1016 1019 1020 1024 1025 1027 1029 1030 1034 1035 1041 1045 1046 1048 1049 1050 1051 1056 1061 1063 1064 1065 1066 1068 1069 1074 1078
GameVisit	3	1 2 3
Group	2	Vegetable Control
Gender	2	Man Woman
SampleName	1	Kale chopped

Dimensions	
Covariance Parameters	2
Columns in X	14
Columns in Z	0
Subjects	34
Max Obs per Subject	3

Number of Observations	
Number of Observations Read	102
Number of Observations Used	102
Number of Observations Not Used	0

Iteration History			
Iteration	Evaluations	-2 Res Log Like	Criterion
0	1	821.13619266	

Iteration History			
**Iteration**	**Evaluations**	**-2 Res Log Like**	**Criterion**
**1**	1	783.91391804	0.00000000

Convergence criteria met.

Covariance Parameter Estimates		
**Cov Parm**	**Subject**	**Estimate**
**CS**	**ParticipantID**	172.06
**Residual**		100.64

Fit Statistics	
**-2 Res Log Likelihood**	783.9
**AIC (Smaller is Better)**	787.9
**AICC (Smaller is Better)**	788.0
**BIC (Smaller is Better)**	791.0

Null Model Likelihood Ratio Test		
**DF**	**Chi-Square**	**Pr > ChiSq**
1	37.22	<.0001

Solution for Fixed Effects								
**Effect**	**Group**	**Gender**	**GameVisit**	**Estimate**	**Standard Error**	**DF**	**t Value**	**Pr > |t|**
**Intercept**				14.0650	4.6106	46.7	3.05	0.0038
**GameVisit**			**1**	2.7500	3.5469	64	0.78	0.4410
**GameVisit**			**2**	-0.06250	3.5469	64	-0.02	0.9860
**GameVisit**			**3**	0	.	.	.	.
**Group**	**Vegetable**			2.8439	5.7352	51.3	0.50	0.6221
**Group**	**Control**			0	.	.	.	.
**GameVisit*Group**	**Vegetable**		**1**	-7.7500	4.8747	64	-1.59	0.1168
**GameVisit*Group**	**Control**		**1**	0	.	.	.	.
**GameVisit*Group**	**Vegetable**		**2**	1.5625	4.8747	64	0.32	0.7496
**GameVisit*Group**	**Control**		**2**	0	.	.	.	.
**GameVisit*Group**	**Vegetable**		**3**	0	.	.	.	.
**GameVisit*Group**	**Control**		**3**	0	.	.	.	.
**Gender**		**Man**		0.1599	5.4742	31	0.03	0.9769
**Gender**		**Woman**		0	.	.	.	.

Type 3 Tests of Fixed Effects				
**Effect**	**Num DF**	**Den DF**	**F Value**	**Pr > F**
**GameVisit**	2	64	0.29	0.7487
**Group**	1	31	0.02	0.8768
**GameVisit*Group**	2	64	2.09	0.1316
**Gender**	1	31	0.00	0.9769

Least Squares Means Estimate						
Effect	Label	Estimate	Standard Error	DF	t Value	Pr > |t|
GameVisit*Group	Control, GV3-GV1	-2.7500	3.5469	64	-0.78	0.4410

Least Squares Means Estimate						
Effect	Label	Estimate	Standard Error	DF	t Value	Pr > |t|
GameVisit*Group	Control, GV2-GV1	-2.8125	3.5469	64	-0.79	0.4307

Least Squares Means Estimate						
Effect	Label	Estimate	Standard Error	DF	t Value	Pr > |t|
GameVisit*Group	Veg, GV3-GV1	5.0000	3.3440	64	1.50	0.1398

Least Squares Means Estimate						
Effect	Label	Estimate	Standard Error	DF	t Value	Pr > |t|
GameVisit*Group	Veg, GV2-GV1	6.5000	3.3440	64	1.94	0.0563

Least Squares Means Estimate						
Effect	Label	Estimate	Standard Error	DF	t Value	Pr > |t|
GameVisit*Group	Veg - Control, GV1	-4.9061	5.7352	51.29	-0.86	0.3963

Least Squares Means Estimate						
Effect	Label	Estimate	Standard Error	DF	t Value	Pr > |t|
GameVisit*Group	Veg - Control, GV2	4.4064	5.7352	51.29	0.77	0.4458

Least Squares Means Estimate						
Effect	Label	Estimate	Standard Error	DF	t Value	Pr > |t|
GameVisit*Group	Veg - Control, GV3	2.8439	5.7352	51.29	0.50	0.6221

Least Squares Means							
Effect	Group	GameVisit	Estimate	Standard Error	DF	t Value	Pr > |t|
Group	Vegetable		15.8222	3.7060	31	4.27	0.0002
Group	Control		15.0408	3.6494	31	4.12	0.0003
GameVisit		1	14.4419	3.0436	48.4	4.74	<.0001
GameVisit		2	16.2857	3.0436	48.4	5.35	<.0001
GameVisit		3	15.5669	3.0436	48.4	5.11	<.0001
GameVisit*Group	Vegetable	1	11.9889	4.1788	48.4	2.87	0.0061
GameVisit*Group	Control	1	16.8950	4.1847	51.1	4.04	0.0002
GameVisit*Group	Vegetable	2	18.4889	4.1788	48.4	4.42	<.0001
GameVisit*Group	Control	2	14.0825	4.1847	51.1	3.37	0.0015
GameVisit*Group	Vegetable	3	16.9889	4.1788	48.4	4.07	0.0002
GameVisit*Group	Control	3	14.1450	4.1847	51.1	3.38	0.0014

Differences of Least Squares Means									
Effect	Group	GameVisit	_Group	_GameVisit	Estimate	Standard Error	DF	t Value	Pr > |t|
Group	Vegetable		Control		0.7814	4.9972	31	0.16	0.8768

Differences of Least Squares Means									
**Effect**	**Group**	**GameVisit**	**_Group**	**_GameVisit**	**Estimate**	**Standard Error**	**DF**	**t Value**	**Pr > |t|**
**GameVisit**		1		2	-1.8437	2.4374	64	-0.76	0.4522
**GameVisit**		1		3	-1.1250	2.4374	64	-0.46	0.6460
**GameVisit**		2		3	0.7187	2.4374	64	0.29	0.7690
**GameVisit*Group**	Vegetable	1	Control	1	-4.9061	5.7352	51.3	-0.86	0.3963
**GameVisit*Group**	Vegetable	1	Vegetable	2	-6.5000	3.3440	64	-1.94	0.0563
**GameVisit*Group**	Vegetable	1	Control	2	-2.0936	5.7352	51.3	-0.37	0.7166
**GameVisit*Group**	Vegetable	1	Vegetable	3	-5.0000	3.3440	64	-1.50	0.1398
**GameVisit*Group**	Vegetable	1	Control	3	-2.1561	5.7352	51.3	-0.38	0.7085
**GameVisit*Group**	Control	1	Vegetable	2	-1.5939	5.7352	51.3	-0.28	0.7822
**GameVisit*Group**	Control	1	Control	2	2.8125	3.5469	64	0.79	0.4307
**GameVisit*Group**	Control	1	Vegetable	3	-0.09388	5.7352	51.3	-0.02	0.9870
**GameVisit*Group**	Control	1	Control	3	2.7500	3.5469	64	0.78	0.4410
**GameVisit*Group**	Vegetable	2	Control	2	4.4064	5.7352	51.3	0.77	0.4458
**GameVisit*Group**	Vegetable	2	Vegetable	3	1.5000	3.3440	64	0.45	0.6553
**GameVisit*Group**	Vegetable	2	Control	3	4.3439	5.7352	51.3	0.76	0.4523
**GameVisit*Group**	Control	2	Vegetable	3	-2.9064	5.7352	51.3	-0.51	0.6145
**GameVisit*Group**	Control	2	Control	3	-0.06250	3.5469	64	-0.02	0.9860
**GameVisit*Group**	Vegetable	3	Control	3	2.8439	5.7352	51.3	0.50	0.6221

OSF planned analysis: Sweetness

The Mixed Procedure

SampleName=Kale puree

Model Information	
**Data Set**	WORK.GAMEVISITS
**Dependent Variable**	Sweetness
**Covariance Structure**	Compound Symmetry
**Subject Effect**	ParticipantID
**Estimation Method**	REML
**Residual Variance Method**	Profile
**Fixed Effects SE Method**	Kenward-Roger
**Degrees of Freedom Method**	Kenward-Roger

Class Level Information		
**Class**	**Levels**	**Values**
**ParticipantID**	34	1001 1002 1005 1006 1009 1010 1014 1016 1019 1020 1024 1025 1027 1029 1030 1034 1035 1041 1045 1046 1048 1049 1050 1051 1056 1061 1063 1064 1065 1066 1068 1069 1074 1078
**GameVisit**	3	1 2 3
**Group**	2	Vegetable Control
**Gender**	2	Man Woman
**SampleName**	1	Kale puree

Dimensions	
Covariance Parameters	2
Columns in X	14
Columns in Z	0
Subjects	34
Max Obs per Subject	3

Number of Observations	
Number of Observations Read	102
Number of Observations Used	102
Number of Observations Not Used	0

Iteration History			
Iteration	Evaluations	-2 Res Log Like	Criterion
0	1	819.35475859	
1	1	793.00804412	0.00000000

Convergence criteria met.

Covariance Parameter Estimates		
Cov Parm	Subject	Estimate
CS	ParticipantID	145.34
Residual		121.80

Fit Statistics	
-2 Res Log Likelihood	793.0
AIC (Smaller is Better)	797.0
AICC (Smaller is Better)	797.1
BIC (Smaller is Better)	800.1

Null Model Likelihood Ratio Test		
DF	Chi-Square	Pr > ChiSq
1	26.35	<.0001

Solution for Fixed Effects								
Effect	Group	Gender	GameVisit	Estimate	Standard Error	DF	t Value	Pr > |t|
Intercept				15.7270	4.5285	52	3.47	0.0010
GameVisit			1	2.0625	3.9019	64	0.53	0.5989
GameVisit			2	1.1250	3.9019	64	0.29	0.7740
GameVisit			3	0	.	.	.	.
Group	Vegetable			0.1853	5.6718	57.9	0.03	0.9740
Group	Control			0	.	.	.	.
GameVisit*Group	Vegetable		1	-5.0069	5.3626	64	-0.93	0.3540
GameVisit*Group	Control		1	0	.	.	.	.
GameVisit*Group	Vegetable		2	-1.6806	5.3626	64	-0.31	0.7550

Solution for Fixed Effects								
Effect	Group	Gender	GameVisit	Estimate	Standard Error	DF	t Value	Pr > |t|
GameVisit*Group	Control		2	0	.	.	.	.
GameVisit*Group	Vegetable		3	0	.	.	.	.
GameVisit*Group	Control		3	0	.	.	.	.
Gender		Man		-3.1053	5.2058	31	-0.60	0.5552
Gender		Woman		0	.	.	.	.

Type 3 Tests of Fixed Effects				
Effect	Num DF	Den DF	F Value	Pr > F
GameVisit	2	64	0.04	0.9635
Group	1	31	0.18	0.6701
GameVisit*Group	2	64	0.45	0.6386
Gender	1	31	0.36	0.5552

Least Squares Means Estimate						
Effect	Label	Estimate	Standard Error	DF	t Value	Pr > |t|
GameVisit*Group	Control, GV3-GV1	-2.0625	3.9019	64	-0.53	0.5989

Least Squares Means Estimate						
Effect	Label	Estimate	Standard Error	DF	t Value	Pr > |t|
GameVisit*Group	Control, GV2-GV1	-0.9375	3.9019	64	-0.24	0.8109

Least Squares Means Estimate						
Effect	Label	Estimate	Standard Error	DF	t Value	Pr > |t|
GameVisit*Group	Veg, GV3-GV1	2.9444	3.6787	64	0.80	0.4264

Least Squares Means Estimate						
Effect	Label	Estimate	Standard Error	DF	t Value	Pr > |t|
GameVisit*Group	Veg, GV2-GV1	2.3889	3.6787	64	0.65	0.5184

Least Squares Means Estimate						
Effect	Label	Estimate	Standard Error	DF	t Value	Pr > |t|
GameVisit*Group	Veg - Control, GV1	-4.8216	5.6718	57.85	-0.85	0.3988

Least Squares Means Estimate						
Effect	Label	Estimate	Standard Error	DF	t Value	Pr > |t|
GameVisit*Group	Veg - Control, GV2	-1.4952	5.6718	57.85	-0.26	0.7930

Least Squares Means Estimate						
Effect	Label	Estimate	Standard Error	DF	t Value	Pr > |t|
GameVisit*Group	Veg - Control, GV3	0.1853	5.6718	57.85	0.03	0.9740

Least Squares Means							
Effect	Group	GameVisit	Estimate	Standard Error	DF	t Value	Pr > |t|

Least Squares Means							
Effect	Group	GameVisit	Estimate	Standard Error	DF	t Value	Pr > |t|
Group	Vegetable		13.1930	3.5243	31	3.74	0.0007
Group	Control		15.2368	3.4705	31	4.39	0.0001
GameVisit		1	13.8260	2.9972	54.2	4.61	<.0001
GameVisit		2	14.5517	2.9972	54.2	4.86	<.0001
GameVisit		3	14.2670	2.9972	54.2	4.76	<.0001
GameVisit*Group	Vegetable	1	11.4152	4.1149	54.1	2.77	0.0076
GameVisit*Group	Control	1	16.2368	4.1376	57.7	3.92	0.0002
GameVisit*Group	Vegetable	2	13.8041	4.1149	54.1	3.35	0.0015
GameVisit*Group	Control	2	15.2993	4.1376	57.7	3.70	0.0005
GameVisit*Group	Vegetable	3	14.3596	4.1149	54.1	3.49	0.0010
GameVisit*Group	Control	3	14.1743	4.1376	57.7	3.43	0.0011

Differences of Least Squares Means									
Effect	Group	GameVisit	_Group	_GameVisit	Estimate	Standard Error	DF	t Value	Pr > |t|
Group	Vegetable		Control		-2.0439	4.7522	31	-0.43	0.6701
GameVisit		1		2	-0.7257	2.6813	64	-0.27	0.7875
GameVisit		1		3	-0.4410	2.6813	64	-0.16	0.8699
GameVisit		2		3	0.2847	2.6813	64	0.11	0.9158
GameVisit*Group	Vegetable	1	Control	1	-4.8216	5.6718	57.9	-0.85	0.3988
GameVisit*Group	Vegetable	1	Vegetable	2	-2.3889	3.6787	64	-0.65	0.5184
GameVisit*Group	Vegetable	1	Control	2	-3.8841	5.6718	57.9	-0.68	0.4962
GameVisit*Group	Vegetable	1	Vegetable	3	-2.9444	3.6787	64	-0.80	0.4264
GameVisit*Group	Vegetable	1	Control	3	-2.7591	5.6718	57.9	-0.49	0.6285
GameVisit*Group	Control	1	Vegetable	2	2.4327	5.6718	57.9	0.43	0.6696
GameVisit*Group	Control	1	Control	2	0.9375	3.9019	64	0.24	0.8109
GameVisit*Group	Control	1	Vegetable	3	1.8772	5.6718	57.9	0.33	0.7419
GameVisit*Group	Control	1	Control	3	2.0625	3.9019	64	0.53	0.5989
GameVisit*Group	Vegetable	2	Control	2	-1.4952	5.6718	57.9	-0.26	0.7930
GameVisit*Group	Vegetable	2	Vegetable	3	-0.5556	3.6787	64	-0.15	0.8804
GameVisit*Group	Vegetable	2	Control	3	-0.3702	5.6718	57.9	-0.07	0.9482
GameVisit*Group	Control	2	Vegetable	3	0.9397	5.6718	57.9	0.17	0.8690
GameVisit*Group	Control	2	Control	3	1.1250	3.9019	64	0.29	0.7740
GameVisit*Group	Vegetable	3	Control	3	0.1853	5.6718	57.9	0.03	0.9740

OSF planned analysis: Sweetness

The Mixed Procedure

SampleName=Oat puree

Model Information	
Data Set	WORK.GAMEVISITS

Model Information	
Dependent Variable	Sweetness
Covariance Structure	Compound Symmetry
Subject Effect	ParticipantID
Estimation Method	REML
Residual Variance Method	Profile
Fixed Effects SE Method	Kenward-Roger
Degrees of Freedom Method	Kenward-Roger

Class Level Information		
Class	Levels	Values
ParticipantID	34	1001 1002 1005 1006 1009 1010 1014 1016 1019 1020 1024 1025 1027 1029 1030 1034 1035 1041 1045 1046 1048 1049 1050 1051 1056 1061 1063 1064 1065 1066 1068 1069 1074 1078
GameVisit	3	1 2 3
Group	2	Vegetable Control
Gender	2	Man Woman
SampleName	1	Oat puree

Dimensions	
Covariance Parameters	2
Columns in X	14
Columns in Z	0
Subjects	34
Max Obs per Subject	3

Number of Observations	
Number of Observations Read	102
Number of Observations Used	102
Number of Observations Not Used	0

Iteration History			
Iteration	Evaluations	-2 Res Log Like	Criterion
0	1	823.31207379	
1	1	796.60931612	0.00000000

Convergence criteria met.

Covariance Parameter Estimates		
Cov Parm	Subject	Estimate
CS	ParticipantID	152.44
Residual		126.08

Fit Statistics	
-2 Res Log Likelihood	796.6
AIC (Smaller is Better)	800.6
AICC (Smaller is Better)	800.7

Fit Statistics	
BIC (Smaller is Better)	803.7

Null Model Likelihood Ratio Test		
DF	Chi-Square	Pr > ChiSq
1	26.70	<.0001

Solution for Fixed Effects								
Effect	Group	Gender	GameVisit	Estimate	Standard Error	DF	t Value	Pr > |t|
Intercept				17.9929	4.6253	51.8	3.89	0.0003
GameVisit			1	1.1875	3.9699	64	0.30	0.7658
GameVisit			2	-1.9375	3.9699	64	-0.49	0.6272
GameVisit			3	0	.	.	.	.
Group	Vegetable			4.5214	5.7916	57.6	0.78	0.4382
Group	Control			0	.	.	.	.
GameVisit*Group	Vegetable		1	-5.9653	5.4561	64	-1.09	0.2783
GameVisit*Group	Control		1	0	.	.	.	.
GameVisit*Group	Vegetable		2	-2.0625	5.4561	64	-0.38	0.7067
GameVisit*Group	Control		2	0	.	.	.	.
GameVisit*Group	Vegetable		3	0	.	.	.	.
GameVisit*Group	Control		3	0	.	.	.	.
Gender		Man		-4.3144	5.3238	31	-0.81	0.4239
Gender		Woman		0	.	.	.	.

Type 3 Tests of Fixed Effects				
Effect	Num DF	Den DF	F Value	Pr > F
GameVisit	2	64	0.60	0.5514
Group	1	31	0.14	0.7067
GameVisit*Group	2	64	0.62	0.5429
Gender	1	31	0.66	0.4239

Least Squares Means Estimate						
Effect	Label	Estimate	Standard Error	DF	t Value	Pr > |t|
GameVisit*Group	Control, GV3-GV1	-1.1875	3.9699	64	-0.30	0.7658

Least Squares Means Estimate						
Effect	Label	Estimate	Standard Error	DF	t Value	Pr > |t|
GameVisit*Group	Control, GV2-GV1	-3.1250	3.9699	64	-0.79	0.4341

Least Squares Means Estimate						
Effect	Label	Estimate	Standard Error	DF	t Value	Pr > |t|
GameVisit*Group	Veg, GV3-GV1	4.7778	3.7428	64	1.28	0.2064

Least Squares Means Estimate						
Effect	Label	Estimate	Standard Error	DF	t Value	Pr > |t|

Least Squares Means Estimate						
Effect	Label	Estimate	Standard Error	DF	t Value	Pr > |t|
GameVisit*Group	Veg, GV2-GV1	0.7778	3.7428	64	0.21	0.8360

Least Squares Means Estimate						
Effect	Label	Estimate	Standard Error	DF	t Value	Pr > |t|
GameVisit*Group	Veg - Control, GV1	-1.4439	5.7916	57.59	-0.25	0.8040

Least Squares Means Estimate						
Effect	Label	Estimate	Standard Error	DF	t Value	Pr > |t|
GameVisit*Group	Veg - Control, GV2	2.4589	5.7916	57.59	0.42	0.6727

Least Squares Means Estimate						
Effect	Label	Estimate	Standard Error	DF	t Value	Pr > |t|
GameVisit*Group	Veg - Control, GV3	4.5214	5.7916	57.59	0.78	0.4382

Least Squares Means							
Effect	Group	GameVisit	Estimate	Standard Error	DF	t Value	Pr > |t|
Group	Vegetable		17.4312	3.6043	31	4.84	<.0001
Group	Control		15.5857	3.5492	31	4.39	0.0001
GameVisit		1	16.3013	3.0610	54	5.33	<.0001
GameVisit		2	15.1276	3.0610	54	4.94	<.0001
GameVisit		3	18.0964	3.0610	54	5.91	<.0001
GameVisit*Group	Vegetable	1	15.5793	4.2024	53.9	3.71	0.0005
GameVisit*Group	Control	1	17.0232	4.2250	57.4	4.03	0.0002
GameVisit*Group	Vegetable	2	16.3571	4.2024	53.9	3.89	0.0003
GameVisit*Group	Control	2	13.8982	4.2250	57.4	3.29	0.0017
GameVisit*Group	Vegetable	3	20.3571	4.2024	53.9	4.84	<.0001
GameVisit*Group	Control	3	15.8357	4.2250	57.4	3.75	0.0004

Differences of Least Squares Means									
Effect	Group	GameVisit	_Group	_GameVisit	Estimate	Standard Error	DF	t Value	Pr > |t|
Group	Vegetable		Control		1.8455	4.8600	31	0.38	0.7067
GameVisit		1		2	1.1736	2.7280	64	0.43	0.6685
GameVisit		1		3	-1.7951	2.7280	64	-0.66	0.5129
GameVisit		2		3	-2.9688	2.7280	64	-1.09	0.2806
GameVisit*Group	Vegetable	1	Control	1	-1.4439	5.7916	57.6	-0.25	0.8040
GameVisit*Group	Vegetable	1	Vegetable	2	-0.7778	3.7428	64	-0.21	0.8360
GameVisit*Group	Vegetable	1	Control	2	1.6811	5.7916	57.6	0.29	0.7727
GameVisit*Group	Vegetable	1	Vegetable	3	-4.7778	3.7428	64	-1.28	0.2064
GameVisit*Group	Vegetable	1	Control	3	-0.2564	5.7916	57.6	-0.04	0.9648
GameVisit*Group	Control	1	Vegetable	2	0.6661	5.7916	57.6	0.12	0.9088
GameVisit*Group	Control	1	Control	2	3.1250	3.9699	64	0.79	0.4341
GameVisit*Group	Control	1	Vegetable	3	-3.3339	5.7916	57.6	-0.58	0.5671

Differences of Least Squares Means									
Effect	Group	GameVisit	_Group	_GameVisit	Estimate	Standard Error	DF	t Value	Pr > |t|
GameVisit*Group	Control	1	Control	3	1.1875	3.9699	64	0.30	0.7658
GameVisit*Group	Vegetable	2	Control	2	2.4589	5.7916	57.6	0.42	0.6727
GameVisit*Group	Vegetable	2	Vegetable	3	-4.0000	3.7428	64	-1.07	0.2892
GameVisit*Group	Vegetable	2	Control	3	0.5214	5.7916	57.6	0.09	0.9286
GameVisit*Group	Control	2	Vegetable	3	-6.4589	5.7916	57.6	-1.12	0.2694
GameVisit*Group	Control	2	Control	3	-1.9375	3.9699	64	-0.49	0.6272
GameVisit*Group	Vegetable	3	Control	3	4.5214	5.7916	57.6	0.78	0.4382

OSF planned analysis: Sweetness

The Mixed Procedure

SampleName=Spinach chopped

Model Information	
Data Set	WORK.GAMEVISITS
Dependent Variable	Sweetness
Covariance Structure	Compound Symmetry
Subject Effect	ParticipantID
Estimation Method	REML
Residual Variance Method	Profile
Fixed Effects SE Method	Kenward-Roger
Degrees of Freedom Method	Kenward-Roger

Class Level Information		
Class	Levels	Values
ParticipantID	34	1001 1002 1005 1006 1009 1010 1014 1016 1019 1020 1024 1025 1027 1029 1030 1034 1035 1041 1045 1046 1048 1049 1050 1051 1056 1061 1063 1064 1065 1066 1068 1069 1074 1078
GameVisit	3	1 2 3
Group	2	Vegetable Control
Gender	2	Man Woman
SampleName	1	Spinach chopped

Dimensions	
Covariance Parameters	2
Columns in X	14
Columns in Z	0
Subjects	34
Max Obs per Subject	3

Number of Observations	
Number of Observations Read	102
Number of Observations Used	102

Number of Observations	
Number of Observations Not Used	0

Iteration History			
Iteration	Evaluations	-2 Res Log Like	Criterion
0	1	813.95170671	
1	1	770.36251085	0.00000000

Convergence criteria met.

Covariance Parameter Estimates		
Cov Parm	Subject	Estimate
CS	ParticipantID	170.07
Residual		82.9924

Fit Statistics	
-2 Res Log Likelihood	770.4
AIC (Smaller is Better)	774.4
AICC (Smaller is Better)	774.5
BIC (Smaller is Better)	777.4

Null Model Likelihood Ratio Test		
DF	Chi-Square	Pr > ChiSq
1	43.59	<.0001

Solution for Fixed Effects								
Effect	Group	Gender	GameVisit	Estimate	Standard Error	DF	t Value	Pr > |t|
Intercept				16.1293	4.4574	44.5	3.62	0.0008
GameVisit			1	-2.6875	3.2209	64	-0.83	0.4072
GameVisit			2	-3.0000	3.2209	64	-0.93	0.3551
GameVisit			3	0	.	.	.	.
Group	Vegetable			5.0770	5.5270	48.4	0.92	0.3629
Group	Control			0	.	.	.	.
GameVisit*Group	Vegetable		1	-4.5347	4.4267	64	-1.02	0.3095
GameVisit*Group	Control		1	0	.	.	.	.
GameVisit*Group	Vegetable		2	-6.6111	4.4267	64	-1.49	0.1402
GameVisit*Group	Control		2	0	.	.	.	.
GameVisit*Group	Vegetable		3	0	.	.	.	.
GameVisit*Group	Control		3	0	.	.	.	.
Gender		Man		-3.6781	5.3683	31	-0.69	0.4983
Gender		Woman		0	.	.	.	.

Type 3 Tests of Fixed Effects				
Effect	Num DF	Den DF	F Value	Pr > F
GameVisit	2	64	4.50	0.0148

Type 3 Tests of Fixed Effects				
Effect	Num DF	Den DF	F Value	Pr > F
Group	1	31	0.08	0.7830
GameVisit*Group	2	64	1.17	0.3179
Gender	1	31	0.47	0.4983

Least Squares Means Estimate						
Effect	Label	Estimate	Standard Error	DF	t Value	Pr > |t|
GameVisit*Group	Control, GV3-GV1	2.6875	3.2209	64	0.83	0.4072

Least Squares Means Estimate						
Effect	Label	Estimate	Standard Error	DF	t Value	Pr > |t|
GameVisit*Group	Control, GV2-GV1	-0.3125	3.2209	64	-0.10	0.9230

Least Squares Means Estimate						
Effect	Label	Estimate	Standard Error	DF	t Value	Pr > |t|
GameVisit*Group	Veg, GV3-GV1	7.2222	3.0367	64	2.38	0.0204

Least Squares Means Estimate						
Effect	Label	Estimate	Standard Error	DF	t Value	Pr > |t|
GameVisit*Group	Veg, GV2-GV1	-2.3889	3.0367	64	-0.79	0.4344

Least Squares Means Estimate						
Effect	Label	Estimate	Standard Error	DF	t Value	Pr > |t|
GameVisit*Group	Veg - Control, GV1	0.5422	5.5270	48.42	0.10	0.9223

Least Squares Means Estimate						
Effect	Label	Estimate	Standard Error	DF	t Value	Pr > |t|
GameVisit*Group	Veg - Control, GV2	-1.5342	5.5270	48.42	-0.28	0.7825

Least Squares Means Estimate						
Effect	Label	Estimate	Standard Error	DF	t Value	Pr > |t|
GameVisit*Group	Veg - Control, GV3	5.0770	5.5270	48.42	0.92	0.3629

Least Squares Means							
Effect	Group	GameVisit	Estimate	Standard Error	DF	t Value	Pr > |t|
Group	Vegetable		13.7561	3.6344	31	3.78	0.0007
Group	Control		12.3944	3.5789	31	3.46	0.0016
GameVisit		1	11.8738	2.9390	45.9	4.04	0.0002
GameVisit		2	10.5232	2.9390	45.9	3.58	0.0008
GameVisit		3	16.8287	2.9390	45.9	5.73	<.0001
GameVisit*Group	Vegetable	1	12.1450	4.0352	45.9	3.01	0.0042
GameVisit*Group	Control	1	11.6027	4.0332	48.3	2.88	0.0060
GameVisit*Group	Vegetable	2	9.7561	4.0352	45.9	2.42	0.0196
GameVisit*Group	Control	2	11.2902	4.0332	48.3	2.80	0.0073

Least Squares Means							
Effect	Group	GameVisit	Estimate	Standard Error	DF	t Value	Pr > |t|
GameVisit*Group	Vegetable	3	19.3672	4.0352	45.9	4.80	<.0001
GameVisit*Group	Control	3	14.2902	4.0332	48.3	3.54	0.0009

Differences of Least Squares Means									
Effect	Group	GameVisit	_Group	_GameVisit	Estimate	Standard Error	DF	t Value	Pr > |t|
Group	Vegetable		Control		1.3617	4.9006	31	0.28	0.7830
GameVisit		1		2	1.3507	2.2133	64	0.61	0.5439
GameVisit		1		3	-4.9549	2.2133	64	-2.24	0.0287
GameVisit		2		3	-6.3056	2.2133	64	-2.85	0.0059
GameVisit*Group	Vegetable	1	Control	1	0.5422	5.5270	48.4	0.10	0.9223
GameVisit*Group	Vegetable	1	Vegetable	2	2.3889	3.0367	64	0.79	0.4344
GameVisit*Group	Vegetable	1	Control	2	0.8547	5.5270	48.4	0.15	0.8777
GameVisit*Group	Vegetable	1	Vegetable	3	-7.2222	3.0367	64	-2.38	0.0204
GameVisit*Group	Vegetable	1	Control	3	-2.1453	5.5270	48.4	-0.39	0.6996
GameVisit*Group	Control	1	Vegetable	2	1.8467	5.5270	48.4	0.33	0.7397
GameVisit*Group	Control	1	Control	2	0.3125	3.2209	64	0.10	0.9230
GameVisit*Group	Control	1	Vegetable	3	-7.7645	5.5270	48.4	-1.40	0.1665
GameVisit*Group	Control	1	Control	3	-2.6875	3.2209	64	-0.83	0.4072
GameVisit*Group	Vegetable	2	Control	2	-1.5342	5.5270	48.4	-0.28	0.7825
GameVisit*Group	Vegetable	2	Vegetable	3	-9.6111	3.0367	64	-3.17	0.0024
GameVisit*Group	Vegetable	2	Control	3	-4.5342	5.5270	48.4	-0.82	0.4160
GameVisit*Group	Control	2	Vegetable	3	-8.0770	5.5270	48.4	-1.46	0.1504
GameVisit*Group	Control	2	Control	3	-3.0000	3.2209	64	-0.93	0.3551
GameVisit*Group	Vegetable	3	Control	3	5.0770	5.5270	48.4	0.92	0.3629

OSF planned analysis: Sweetness

The Mixed Procedure

SampleName=Spinach puree

Model Information	
Data Set	WORK.GAMEVISITS
Dependent Variable	Sweetness
Covariance Structure	Compound Symmetry
Subject Effect	ParticipantID
Estimation Method	REML
Residual Variance Method	Profile
Fixed Effects SE Method	Kenward-Roger
Degrees of Freedom Method	Kenward-Roger

Class Level Information		
Class	Levels	Values

Class Level Information		
**Class**	**Levels**	**Values**
**ParticipantID**	34	1001 1002 1005 1006 1009 1010 1014 1016 1019 1020 1024 1025 1027 1029 1030 1034 1035 1041 1045 1046 1048 1049 1050 1051 1056 1061 1063 1064 1065 1066 1068 1069 1074 1078
**GameVisit**	3	1 2 3
**Group**	2	Vegetable Control
**Gender**	2	Man Woman
**SampleName**	1	Spinach puree

Dimensions	
**Covariance Parameters**	2
**Columns in X**	14
**Columns in Z**	0
**Subjects**	34
**Max Obs per Subject**	3

Number of Observations	
**Number of Observations Read**	102
**Number of Observations Used**	102
**Number of Observations Not Used**	0

Iteration History			
**Iteration**	**Evaluations**	**-2 Res Log Like**	**Criterion**
**0**	1	790.63337560	
**1**	1	741.45758694	0.00000000

Convergence criteria met.

Covariance Parameter Estimates		
**Cov Parm**	**Subject**	**Estimate**
**CS**	ParticipantID	139.39
**Residual**		58.7216

Fit Statistics	
**-2 Res Log Likelihood**	741.5
**AIC (Smaller is Better)**	745.5
**AICC (Smaller is Better)**	745.6
**BIC (Smaller is Better)**	748.5

Null Model Likelihood Ratio Test		
**DF**	**Chi-Square**	**Pr > ChiSq**
1	49.18	<.0001

Solution for Fixed Effects								
**Effect**	**Group**	**Gender**	**GameVisit**	**Estimate**	**Standard Error**	**DF**	**t Value**	**Pr > |t|**
**Intercept**				13.5233	3.9548	42.9	3.42	0.0014

Solution for Fixed Effects								
Effect	Group	Gender	GameVisit	Estimate	Standard Error	DF	t Value	Pr > |t|
GameVisit			1	0.5000	2.7093	64	0.18	0.8542
GameVisit			2	-0.2500	2.7093	64	-0.09	0.9268
GameVisit			3	0	.	.	.	.
Group	Vegetable			2.7683	4.8917	46.3	0.57	0.5742
Group	Control			0	.	.	.	.
GameVisit*Group	Vegetable		1	-6.5000	3.7236	64	-1.75	0.0857
GameVisit*Group	Control		1	0	.	.	.	.
GameVisit*Group	Vegetable		2	-4.1389	3.7236	64	-1.11	0.2705
GameVisit*Group	Control		2	0	.	.	.	.
GameVisit*Group	Vegetable		3	0	.	.	.	.
GameVisit*Group	Control		3	0	.	.	.	.
Gender		Man		-3.0621	4.8134	31	-0.64	0.5293
Gender		Woman		0	.	.	.	.

Type 3 Tests of Fixed Effects				
Effect	Num DF	Den DF	F Value	Pr > F
GameVisit	2	64	1.26	0.2899
Group	1	31	0.03	0.8606
GameVisit*Group	2	64	1.56	0.2177
Gender	1	31	0.40	0.5293

Least Squares Means Estimate						
Effect	Label	Estimate	Standard Error	DF	t Value	Pr > |t|
GameVisit*Group	Control, GV3-GV1	-0.5000	2.7093	64	-0.18	0.8542

Least Squares Means Estimate						
Effect	Label	Estimate	Standard Error	DF	t Value	Pr > |t|
GameVisit*Group	Control, GV2-GV1	-0.7500	2.7093	64	-0.28	0.7828

Least Squares Means Estimate						
Effect	Label	Estimate	Standard Error	DF	t Value	Pr > |t|
GameVisit*Group	Veg, GV3-GV1	6.0000	2.5543	64	2.35	0.0219

Least Squares Means Estimate						
Effect	Label	Estimate	Standard Error	DF	t Value	Pr > |t|
GameVisit*Group	Veg, GV2-GV1	1.6111	2.5543	64	0.63	0.5305

Least Squares Means Estimate						
Effect	Label	Estimate	Standard Error	DF	t Value	Pr > |t|
GameVisit*Group	Veg - Control, GV1	-3.7317	4.8917	46.33	-0.76	0.4494

Least Squares Means Estimate						
Effect	Label	Estimate	Standard Error	DF	t Value	Pr > |t|

Least Squares Means Estimate						
Effect	Label	Estimate	Standard Error	DF	t Value	Pr > |t|
GameVisit*Group	Veg - Control, GV2	-1.3706	4.8917	46.33	-0.28	0.7806

Least Squares Means Estimate						
Effect	Label	Estimate	Standard Error	DF	t Value	Pr > |t|
GameVisit*Group	Veg - Control, GV3	2.7683	4.8917	46.33	0.57	0.5742

Least Squares Means							
Effect	Group	GameVisit	Estimate	Standard Error	DF	t Value	Pr > |t|
Group	Vegetable		11.2976	3.2587	31	3.47	0.0016
Group	Control		12.0756	3.2089	31	3.76	0.0007
GameVisit		1	10.6264	2.6051	44.1	4.08	0.0002
GameVisit		2	11.0569	2.6051	44.1	4.24	0.0001
GameVisit		3	13.3764	2.6051	44.1	5.13	<.0001
GameVisit*Group	Vegetable	1	8.7605	3.5769	44.1	2.45	0.0184
GameVisit*Group	Control	1	12.4922	3.5699	46.2	3.50	0.0010
GameVisit*Group	Vegetable	2	10.3716	3.5769	44.1	2.90	0.0058
GameVisit*Group	Control	2	11.7422	3.5699	46.2	3.29	0.0019
GameVisit*Group	Vegetable	3	14.7605	3.5769	44.1	4.13	0.0002
GameVisit*Group	Control	3	11.9922	3.5699	46.2	3.36	0.0016

Differences of Least Squares Means									
Effect	Group	GameVisit	_Group	_GameVisit	Estimate	Standard Error	DF	t Value	Pr > |t|
Group	Vegetable		Control		-0.7780	4.3940	31	-0.18	0.8606
GameVisit		1		2	-0.4306	1.8618	64	-0.23	0.8179
GameVisit		1		3	-2.7500	1.8618	64	-1.48	0.1446
GameVisit		2		3	-2.3194	1.8618	64	-1.25	0.2174
GameVisit*Group	Vegetable	1	Control	1	-3.7317	4.8917	46.3	-0.76	0.4494
GameVisit*Group	Vegetable	1	Vegetable	2	-1.6111	2.5543	64	-0.63	0.5305
GameVisit*Group	Vegetable	1	Control	2	-2.9817	4.8917	46.3	-0.61	0.5451
GameVisit*Group	Vegetable	1	Vegetable	3	-6.0000	2.5543	64	-2.35	0.0219
GameVisit*Group	Vegetable	1	Control	3	-3.2317	4.8917	46.3	-0.66	0.5121
GameVisit*Group	Control	1	Vegetable	2	2.1206	4.8917	46.3	0.43	0.6667
GameVisit*Group	Control	1	Control	2	0.7500	2.7093	64	0.28	0.7828
GameVisit*Group	Control	1	Vegetable	3	-2.2683	4.8917	46.3	-0.46	0.6450
GameVisit*Group	Control	1	Control	3	0.5000	2.7093	64	0.18	0.8542
GameVisit*Group	Vegetable	2	Control	2	-1.3706	4.8917	46.3	-0.28	0.7806
GameVisit*Group	Vegetable	2	Vegetable	3	-4.3889	2.5543	64	-1.72	0.0906
GameVisit*Group	Vegetable	2	Control	3	-1.6206	4.8917	46.3	-0.33	0.7419
GameVisit*Group	Control	2	Vegetable	3	-3.0183	4.8917	46.3	-0.62	0.5402
GameVisit*Group	Control	2	Control	3	-0.2500	2.7093	64	-0.09	0.9268
GameVisit*Group	Vegetable	3	Control	3	2.7683	4.8917	46.3	0.57	0.5742

In [6]:

```
%%SAS sas

proc sort data=gamevisits;
by samplename;
run;

title 'OSF planned analysis: Bitterness';
ods output diffs=diff3 tests3=tests3
SolutionF=sol3 lsmeans=means3 FitStatistics=fit3 lsmESTIMATEs =LikeEstim3;
proc mixed data= gamevisits;
class participantID gamevisit group (ref = 'Control') gender samplename;
by samplename;
model Bitterness = gamevisit group gamevisit*group gender/ ddfm=kr solution; *fit is slightly better w
repeated/ subject = participantID type = cs;
lsmeans group/pdiff ADJDFE=ROW;
lsmeans gamevisit/pdiff ADJDFE=ROW;
lsmeans gamevisit*group/pdiff adjdfe=row;
lsmestimate gamevisit*group 'Control, GV3-GV1' 0 -1 0 0 0 1 ;
lsmestimate gamevisit*group 'Control, GV2-GV1' 0 -1 0 1 0 0 ;
lsmestimate gamevisit*group 'Veg, GV3-GV1' -1 0 0 0 1 0 ;
lsmestimate gamevisit*group 'Veg, GV2-GV1' -1 0 1 0 0 0 ;
lsmestimate gamevisit*group 'Veg - Control, GV1' 1 -1 0 0 0 0 ;
lsmestimate gamevisit*group 'Veg - Control, GV2' 0 0 1 -1 0 0 ;
lsmestimate gamevisit*group 'Veg - Control, GV3' 0 0 0 0 1 -1 ;
run;
```

Out[6]:

OSF planned analysis: Bitterness

The Mixed Procedure

SampleName=Asparagus chopped

Model Information	
Data Set	WORK.GAMEVISITS
Dependent Variable	Bitterness
Covariance Structure	Compound Symmetry
Subject Effect	ParticipantID
Estimation Method	REML
Residual Variance Method	Profile
Fixed Effects SE Method	Kenward-Roger
Degrees of Freedom Method	Kenward-Roger

Class Level Information		
Class	Levels	Values
ParticipantID	34	1001 1002 1005 1006 1009 1010 1014 1016 1019 1020 1024 1025 1027 1029 1030 1034 1035 1041 1045 1046 1048 1049 1050 1051 1056 1061 1063 1064 1065 1066 1068 1069 1074 1078
GameVisit	3	1 2 3
Group	2	Vegetable Control
Gender	2	Man Woman
SampleName	1	Asparagus chopped

Dimensions	
Covariance Parameters	2
Columns in X	14
Columns in Z	0
Subjects	34

Dimensions	
Max Obs per Subject	3

Number of Observations	
Number of Observations Read	102
Number of Observations Used	102
Number of Observations Not Used	0

Iteration History			
Iteration	Evaluations	-2 Res Log Like	Criterion
0	1	887.08201949	
1	1	854.17161817	0.00000000

Convergence criteria met.

Covariance Parameter Estimates		
Cov Parm	Subject	Estimate
CS	ParticipantID	327.00
Residual		218.58

Fit Statistics	
-2 Res Log Likelihood	854.2
AIC (Smaller is Better)	858.2
AICC (Smaller is Better)	858.3
BIC (Smaller is Better)	861.2

Null Model Likelihood Ratio Test		
DF	Chi-Square	Pr > ChiSq
1	32.91	<.0001

Solution for Fixed Effects								
Effect	Group	Gender	GameVisit	Estimate	Standard Error	DF	t Value	Pr > |t|
Intercept				26.5767	6.5034	48.6	4.09	0.0002
GameVisit			1	-8.5000	5.2271	64	-1.63	0.1088
GameVisit			2	4.8125	5.2271	64	0.92	0.3607
GameVisit			3	0	.	.	.	.
Group	Vegetable			8.3577	8.1099	53.6	1.03	0.3074
Group	Control			0	.	.	.	.
GameVisit*Group	Vegetable		1	9.2222	7.1840	64	1.28	0.2039
GameVisit*Group	Control		1	0	.	.	.	.
GameVisit*Group	Vegetable		2	4.6319	7.1840	64	0.64	0.5214
GameVisit*Group	Control		2	0	.	.	.	.
GameVisit*Group	Vegetable		3	0	.	.	.	.
GameVisit*Group	Control		3	0	.	.	.	.
Gender		Man		3.7955	7.6341	31	0.50	0.6226

Solution for Fixed Effects								
Effect	Group	Gender	GameVisit	Estimate	Standard Error	DF	t Value	Pr > |t|
Gender		Woman		0	.	.	.	.

Type 3 Tests of Fixed Effects				
Effect	Num DF	Den DF	F Value	Pr > F
GameVisit	2	64	4.84	0.0110
Group	1	31	3.47	0.0721
GameVisit*Group	2	64	0.82	0.4433
Gender	1	31	0.25	0.6226

Least Squares Means Estimate						
Effect	Label	Estimate	Standard Error	DF	t Value	Pr > |t|
GameVisit*Group	Control, GV3-GV1	8.5000	5.2271	64	1.63	0.1088

Least Squares Means Estimate						
Effect	Label	Estimate	Standard Error	DF	t Value	Pr > |t|
GameVisit*Group	Control, GV2-GV1	13.3125	5.2271	64	2.55	0.0133

Least Squares Means Estimate						
Effect	Label	Estimate	Standard Error	DF	t Value	Pr > |t|
GameVisit*Group	Veg, GV3-GV1	-0.7222	4.9282	64	-0.15	0.8839

Least Squares Means Estimate						
Effect	Label	Estimate	Standard Error	DF	t Value	Pr > |t|
GameVisit*Group	Veg, GV2-GV1	8.7222	4.9282	64	1.77	0.0815

Least Squares Means Estimate						
Effect	Label	Estimate	Standard Error	DF	t Value	Pr > |t|
GameVisit*Group	Veg - Control, GV1	17.5799	8.1099	53.59	2.17	0.0346

Least Squares Means Estimate						
Effect	Label	Estimate	Standard Error	DF	t Value	Pr > |t|
GameVisit*Group	Veg - Control, GV2	12.9896	8.1099	53.59	1.60	0.1151

Least Squares Means Estimate						
Effect	Label	Estimate	Standard Error	DF	t Value	Pr > |t|
GameVisit*Group	Veg - Control, GV3	8.3577	8.1099	53.59	1.03	0.3074

Least Squares Means							
Effect	Group	GameVisit	Estimate	Standard Error	DF	t Value	Pr > |t|
Group	Vegetable		40.2210	5.1683	31	7.78	<.0001
Group	Control		27.2453	5.0894	31	5.35	<.0001
GameVisit		1	28.7644	4.2972	50.4	6.69	<.0001
GameVisit		2	39.7817	4.2972	50.4	9.26	<.0001

Least Squares Means							
Effect	Group	GameVisit	Estimate	Standard Error	DF	t Value	Pr > |t|
GameVisit		3	32.6533	4.2972	50.4	7.60	<.0001
GameVisit*Group	Vegetable	1	37.5543	5.8998	50.4	6.37	<.0001
GameVisit*Group	Control	1	19.9744	5.9169	53.4	3.38	0.0014
GameVisit*Group	Vegetable	2	46.2765	5.8998	50.4	7.84	<.0001
GameVisit*Group	Control	2	33.2869	5.9169	53.4	5.63	<.0001
GameVisit*Group	Vegetable	3	36.8321	5.8998	50.4	6.24	<.0001
GameVisit*Group	Control	3	28.4744	5.9169	53.4	4.81	<.0001

Differences of Least Squares Means									
Effect	Group	GameVisit	_Group	_GameVisit	Estimate	Standard Error	DF	t Value	Pr > |t|
Group	Vegetable		Control		12.9757	6.9690	31	1.86	0.0721
GameVisit		1		2	-11.0174	3.5920	64	-3.07	0.0032
GameVisit		1		3	-3.8889	3.5920	64	-1.08	0.2830
GameVisit		2		3	7.1285	3.5920	64	1.98	0.0515
GameVisit*Group	Vegetable	1	Control	1	17.5799	8.1099	53.6	2.17	0.0346
GameVisit*Group	Vegetable	1	Vegetable	2	-8.7222	4.9282	64	-1.77	0.0815
GameVisit*Group	Vegetable	1	Control	2	4.2674	8.1099	53.6	0.53	0.6009
GameVisit*Group	Vegetable	1	Vegetable	3	0.7222	4.9282	64	0.15	0.8839
GameVisit*Group	Vegetable	1	Control	3	9.0799	8.1099	53.6	1.12	0.2679
GameVisit*Group	Control	1	Vegetable	2	-26.3021	8.1099	53.6	-3.24	0.0020
GameVisit*Group	Control	1	Control	2	-13.3125	5.2271	64	-2.55	0.0133
GameVisit*Group	Control	1	Vegetable	3	-16.8577	8.1099	53.6	-2.08	0.0425
GameVisit*Group	Control	1	Control	3	-8.5000	5.2271	64	-1.63	0.1088
GameVisit*Group	Vegetable	2	Control	2	12.9896	8.1099	53.6	1.60	0.1151
GameVisit*Group	Vegetable	2	Vegetable	3	9.4444	4.9282	64	1.92	0.0598
GameVisit*Group	Vegetable	2	Control	3	17.8021	8.1099	53.6	2.20	0.0325
GameVisit*Group	Control	2	Vegetable	3	-3.5452	8.1099	53.6	-0.44	0.6638
GameVisit*Group	Control	2	Control	3	4.8125	5.2271	64	0.92	0.3607
GameVisit*Group	Vegetable	3	Control	3	8.3577	8.1099	53.6	1.03	0.3074

OSF planned analysis: Bitterness

The Mixed Procedure

SampleName=Asparagus puree

Model Information	
Data Set	WORK.GAMEVISITS
Dependent Variable	Bitterness
Covariance Structure	Compound Symmetry
Subject Effect	ParticipantID
Estimation Method	REML

Model Information	
Residual Variance Method	Profile
Fixed Effects SE Method	Kenward-Roger
Degrees of Freedom Method	Kenward-Roger

Class Level Information		
Class	Levels	Values
ParticipantID	34	1001 1002 1005 1006 1009 1010 1014 1016 1019 1020 1024 1025 1027 1029 1030 1034 1035 1041 1045 1046 1048 1049 1050 1051 1056 1061 1063 1064 1065 1066 1068 1069 1074 1078
GameVisit	3	1 2 3
Group	2	Vegetable Control
Gender	2	Man Woman
SampleName	1	Asparagus puree

Dimensions	
Covariance Parameters	2
Columns in X	14
Columns in Z	0
Subjects	34
Max Obs per Subject	3

Number of Observations	
Number of Observations Read	102
Number of Observations Used	102
Number of Observations Not Used	0

Iteration History			
Iteration	Evaluations	-2 Res Log Like	Criterion
0	1	871.10572261	
1	1	838.57900832	0.00000000

Convergence criteria met.

Covariance Parameter Estimates		
Cov Parm	Subject	Estimate
CS	ParticipantID	275.00
Residual		186.11

Fit Statistics	
-2 Res Log Likelihood	838.6
AIC (Smaller is Better)	842.6
AICC (Smaller is Better)	842.7
BIC (Smaller is Better)	845.6

Null Model Likelihood Ratio Test		
DF	Chi-Square	Pr > ChiSq

Null Model Likelihood Ratio Test		
DF	Chi-Square	Pr > ChiSq
1	32.53	<.0001

Solution for Fixed Effects								
Effect	Group	Gender	GameVisit	Estimate	Standard Error	DF	t Value	Pr > |t|
Intercept				28.3231	5.9772	48.8	4.74	<.0001
GameVisit			1	-5.1875	4.8232	64	-1.08	0.2862
GameVisit			2	-0.3750	4.8232	64	-0.08	0.9383
GameVisit			3	0	.	.	.	.
Group	Vegetable			6.9609	7.4554	53.8	0.93	0.3546
Group	Control			0	.	.	.	.
GameVisit*Group	Vegetable		1	5.8542	6.6289	64	0.88	0.3805
GameVisit*Group	Control		1	0	.	.	.	.
GameVisit*Group	Vegetable		2	2.7083	6.6289	64	0.41	0.6842
GameVisit*Group	Control		2	0	.	.	.	.
GameVisit*Group	Vegetable		3	0	.	.	.	.
GameVisit*Group	Control		3	0	.	.	.	.
Gender		Man		-2.0283	7.0087	31	-0.29	0.7742
Gender		Woman		0	.	.	.	.

Type 3 Tests of Fixed Effects				
Effect	Num DF	Den DF	F Value	Pr > F
GameVisit	2	64	0.50	0.6073
Group	1	31	2.35	0.1352
GameVisit*Group	2	64	0.39	0.6782
Gender	1	31	0.08	0.7742

Least Squares Means Estimate						
Effect	Label	Estimate	Standard Error	DF	t Value	Pr > |t|
GameVisit*Group	Control, GV3-GV1	5.1875	4.8232	64	1.08	0.2862

Least Squares Means Estimate						
Effect	Label	Estimate	Standard Error	DF	t Value	Pr > |t|
GameVisit*Group	Control, GV2-GV1	4.8125	4.8232	64	1.00	0.3221

Least Squares Means Estimate						
Effect	Label	Estimate	Standard Error	DF	t Value	Pr > |t|
GameVisit*Group	Veg, GV3-GV1	-0.6667	4.5474	64	-0.15	0.8839

Least Squares Means Estimate						
Effect	Label	Estimate	Standard Error	DF	t Value	Pr > |t|
GameVisit*Group	Veg, GV2-GV1	1.6667	4.5474	64	0.37	0.7152

Least Squares Means Estimate						
Effect	Label	Estimate	Standard Error	DF	t Value	Pr > |t|
GameVisit*Group	Veg - Control, GV1	12.8151	7.4554	53.82	1.72	0.0914

Least Squares Means Estimate						
Effect	Label	Estimate	Standard Error	DF	t Value	Pr > |t|
GameVisit*Group	Veg - Control, GV2	9.6693	7.4554	53.82	1.30	0.2002

Least Squares Means Estimate						
Effect	Label	Estimate	Standard Error	DF	t Value	Pr > |t|
GameVisit*Group	Veg - Control, GV3	6.9609	7.4554	53.82	0.93	0.3546

Least Squares Means							
Effect	Group	GameVisit	Estimate	Standard Error	DF	t Value	Pr > |t|
Group	Vegetable		35.2699	4.7449	31	7.43	<.0001
Group	Control		25.4548	4.6725	31	5.45	<.0001
GameVisit		1	28.5290	3.9498	50.6	7.22	<.0001
GameVisit		2	31.7686	3.9498	50.6	8.04	<.0001
GameVisit		3	30.7894	3.9498	50.6	7.80	<.0001
GameVisit*Group	Vegetable	1	34.9366	5.4228	50.6	6.44	<.0001
GameVisit*Group	Control	1	22.1215	5.4394	53.7	4.07	0.0002
GameVisit*Group	Vegetable	2	36.6032	5.4228	50.6	6.75	<.0001
GameVisit*Group	Control	2	26.9340	5.4394	53.7	4.95	<.0001
GameVisit*Group	Vegetable	3	34.2699	5.4228	50.6	6.32	<.0001
GameVisit*Group	Control	3	27.3090	5.4394	53.7	5.02	<.0001

Differences of Least Squares Means									
Effect	Group	GameVisit	_Group	_GameVisit	Estimate	Standard Error	DF	t Value	Pr > |t|
Group	Vegetable		Control		9.8151	6.3981	31	1.53	0.1352
GameVisit		1		2	-3.2396	3.3144	64	-0.98	0.3320
GameVisit		1		3	-2.2604	3.3144	64	-0.68	0.4977
GameVisit		2		3	0.9792	3.3144	64	0.30	0.7686
GameVisit*Group	Vegetable	1	Control	1	12.8151	7.4554	53.8	1.72	0.0914
GameVisit*Group	Vegetable	1	Vegetable	2	-1.6667	4.5474	64	-0.37	0.7152
GameVisit*Group	Vegetable	1	Control	2	8.0026	7.4554	53.8	1.07	0.2879
GameVisit*Group	Vegetable	1	Vegetable	3	0.6667	4.5474	64	0.15	0.8839
GameVisit*Group	Vegetable	1	Control	3	7.6276	7.4554	53.8	1.02	0.3108
GameVisit*Group	Control	1	Vegetable	2	-14.4818	7.4554	53.8	-1.94	0.0573
GameVisit*Group	Control	1	Control	2	-4.8125	4.8232	64	-1.00	0.3221
GameVisit*Group	Control	1	Vegetable	3	-12.1484	7.4554	53.8	-1.63	0.1090
GameVisit*Group	Control	1	Control	3	-5.1875	4.8232	64	-1.08	0.2862
GameVisit*Group	Vegetable	2	Control	2	9.6693	7.4554	53.8	1.30	0.2002
GameVisit*Group	Vegetable	2	Vegetable	3	2.3333	4.5474	64	0.51	0.6096
GameVisit*Group	Vegetable	2	Control	3	9.2943	7.4554	53.8	1.25	0.2179

Differences of Least Squares Means									
Effect	Group	GameVisit	_Group	_GameVisit	Estimate	Standard Error	DF	t Value	Pr > |t|
GameVisit*Group	Control	2	Vegetable	3	-7.3359	7.4554	53.8	-0.98	0.3295
GameVisit*Group	Control	2	Control	3	-0.3750	4.8232	64	-0.08	0.9383
GameVisit*Group	Vegetable	3	Control	3	6.9609	7.4554	53.8	0.93	0.3546

OSF planned analysis: Bitterness

The Mixed Procedure

SampleName=Beef puree

Model Information	
Data Set	WORK.GAMEVISITS
Dependent Variable	Bitterness
Covariance Structure	Compound Symmetry
Subject Effect	ParticipantID
Estimation Method	REML
Residual Variance Method	Profile
Fixed Effects SE Method	Kenward-Roger
Degrees of Freedom Method	Kenward-Roger

Class Level Information		
Class	Levels	Values
ParticipantID	34	1001 1002 1005 1006 1009 1010 1014 1016 1019 1020 1024 1025 1027 1029 1030 1034 1035 1041 1045 1046 1048 1049 1050 1051 1056 1061 1063 1064 1065 1066 1068 1069 1074 1078
GameVisit	3	1 2 3
Group	2	Vegetable Control
Gender	2	Man Woman
SampleName	1	Beef puree

Dimensions	
Covariance Parameters	2
Columns in X	14
Columns in Z	0
Subjects	34
Max Obs per Subject	3

Number of Observations	
Number of Observations Read	102
Number of Observations Used	102
Number of Observations Not Used	0

Iteration History			
Iteration	Evaluations	-2 Res Log Like	Criterion
0	1	892.56023206	

Iteration History			
Iteration	Evaluations	-2 Res Log Like	Criterion
1	1	831.22195904	0.00000000

Convergence criteria met.

Covariance Parameter Estimates		
Cov Parm	Subject	Estimate
CS	ParticipantID	441.23
Residual		138.74

Fit Statistics	
-2 Res Log Likelihood	831.2
AIC (Smaller is Better)	835.2
AICC (Smaller is Better)	835.4
BIC (Smaller is Better)	838.3

Null Model Likelihood Ratio Test		
DF	Chi-Square	Pr > ChiSq
1	61.34	<.0001

Solution for Fixed Effects								
Effect	Group	Gender	GameVisit	Estimate	Standard Error	DF	t Value	Pr > |t|
Intercept				17.5382	6.7999	40.1	2.58	0.0137
GameVisit			1	6.5625	4.1644	64	1.58	0.1200
GameVisit			2	11.2500	4.1644	64	2.70	0.0088
GameVisit			3	0	.	.	.	.
Group	Vegetable			7.6881	8.3742	42.8	0.92	0.3637
Group	Control			0	.	.	.	.
GameVisit*Group	Vegetable		1	-9.2292	5.7235	64	-1.61	0.1118
GameVisit*Group	Control		1	0	.	.	.	.
GameVisit*Group	Vegetable		2	-12.6389	5.7235	64	-2.21	0.0308
GameVisit*Group	Control		2	0	.	.	.	.
GameVisit*Group	Vegetable		3	0	.	.	.	.
GameVisit*Group	Control		3	0	.	.	.	.
Gender		Man		3.2314	8.4290	31	0.38	0.7041
Gender		Woman		0	.	.	.	.

Type 3 Tests of Fixed Effects				
Effect	Num DF	Den DF	F Value	Pr > F
GameVisit	2	64	1.51	0.2295
Group	1	31	0.00	0.9590
GameVisit*Group	2	64	2.61	0.0813
Gender	1	31	0.15	0.7041

Least Squares Means Estimate						
Effect	Label	Estimate	Standard Error	DF	t Value	Pr > |t|
GameVisit*Group	Control, GV3-GV1	-6.5625	4.1644	64	-1.58	0.1200

Least Squares Means Estimate						
Effect	Label	Estimate	Standard Error	DF	t Value	Pr > |t|
GameVisit*Group	Control, GV2-GV1	4.6875	4.1644	64	1.13	0.2645

Least Squares Means Estimate						
Effect	Label	Estimate	Standard Error	DF	t Value	Pr > |t|
GameVisit*Group	Veg, GV3-GV1	2.6667	3.9263	64	0.68	0.4995

Least Squares Means Estimate						
Effect	Label	Estimate	Standard Error	DF	t Value	Pr > |t|
GameVisit*Group	Veg, GV2-GV1	1.2778	3.9263	64	0.33	0.7459

Least Squares Means Estimate						
Effect	Label	Estimate	Standard Error	DF	t Value	Pr > |t|
GameVisit*Group	Veg - Control, GV1	-1.5410	8.3742	42.78	-0.18	0.8549

Least Squares Means Estimate						
Effect	Label	Estimate	Standard Error	DF	t Value	Pr > |t|
GameVisit*Group	Veg - Control, GV2	-4.9508	8.3742	42.78	-0.59	0.5575

Least Squares Means Estimate						
Effect	Label	Estimate	Standard Error	DF	t Value	Pr > |t|
GameVisit*Group	Veg - Control, GV3	7.6881	8.3742	42.78	0.92	0.3637

Least Squares Means							
Effect	Group	GameVisit	Estimate	Standard Error	DF	t Value	Pr > |t|
Group	Vegetable		25.4902	5.7065	31	4.47	<.0001
Group	Control		25.0914	5.6194	31	4.47	<.0001
GameVisit		1	24.9459	4.4720	41.1	5.58	<.0001
GameVisit		2	27.9286	4.4720	41.1	6.25	<.0001
GameVisit		3	22.9980	4.4720	41.1	5.14	<.0001
GameVisit*Group	Vegetable	1	24.1754	6.1402	41.1	3.94	0.0003
GameVisit*Group	Control	1	25.7164	6.1121	42.7	4.21	0.0001
GameVisit*Group	Vegetable	2	25.4532	6.1402	41.1	4.15	0.0002
GameVisit*Group	Control	2	30.4039	6.1121	42.7	4.97	<.0001
GameVisit*Group	Vegetable	3	26.8421	6.1402	41.1	4.37	<.0001
GameVisit*Group	Control	3	19.1539	6.1121	42.7	3.13	0.0031

Differences of Least Squares Means									
Effect	Group	GameVisit	_Group	_GameVisit	Estimate	Standard Error	DF	t Value	Pr > |t|
Group	Vegetable		Control		0.3988	7.6946	31	0.05	0.9590

Differences of Least Squares Means									
Effect	Group	GameVisit	_Group	_GameVisit	Estimate	Standard Error	DF	t Value	Pr > |t|
GameVisit		1		2	-2.9826	2.8617	64	-1.04	0.3012
GameVisit		1		3	1.9479	2.8617	64	0.68	0.4985
GameVisit		2		3	4.9306	2.8617	64	1.72	0.0897
GameVisit*Group	Vegetable	1	Control	1	-1.5410	8.3742	42.8	-0.18	0.8549
GameVisit*Group	Vegetable	1	Vegetable	2	-1.2778	3.9263	64	-0.33	0.7459
GameVisit*Group	Vegetable	1	Control	2	-6.2285	8.3742	42.8	-0.74	0.4611
GameVisit*Group	Vegetable	1	Vegetable	3	-2.6667	3.9263	64	-0.68	0.4995
GameVisit*Group	Vegetable	1	Control	3	5.0215	8.3742	42.8	0.60	0.5519
GameVisit*Group	Control	1	Vegetable	2	0.2633	8.3742	42.8	0.03	0.9751
GameVisit*Group	Control	1	Control	2	-4.6875	4.1644	64	-1.13	0.2645
GameVisit*Group	Control	1	Vegetable	3	-1.1256	8.3742	42.8	-0.13	0.8937
GameVisit*Group	Control	1	Control	3	6.5625	4.1644	64	1.58	0.1200
GameVisit*Group	Vegetable	2	Control	2	-4.9508	8.3742	42.8	-0.59	0.5575
GameVisit*Group	Vegetable	2	Vegetable	3	-1.3889	3.9263	64	-0.35	0.7247
GameVisit*Group	Vegetable	2	Control	3	6.2992	8.3742	42.8	0.75	0.4560
GameVisit*Group	Control	2	Vegetable	3	3.5619	8.3742	42.8	0.43	0.6727
GameVisit*Group	Control	2	Control	3	11.2500	4.1644	64	2.70	0.0088
GameVisit*Group	Vegetable	3	Control	3	7.6881	8.3742	42.8	0.92	0.3637

OSF planned analysis: Bitterness

The Mixed Procedure

SampleName=Black bean puree

Model Information	
Data Set	WORK.GAMEVISITS
Dependent Variable	Bitterness
Covariance Structure	Compound Symmetry
Subject Effect	ParticipantID
Estimation Method	REML
Residual Variance Method	Profile
Fixed Effects SE Method	Kenward-Roger
Degrees of Freedom Method	Kenward-Roger

Class Level Information		
Class	Levels	Values
ParticipantID	34	1001 1002 1005 1006 1009 1010 1014 1016 1019 1020 1024 1025 1027 1029 1030 1034 1035 1041 1045 1046 1048 1049 1050 1051 1056 1061 1063 1064 1065 1066 1068 1069 1074 1078
GameVisit	3	1 2 3
Group	2	Vegetable Control
Gender	2	Man Woman
SampleName	1	Black bean puree

Dimensions	
Covariance Parameters	2
Columns in X	14
Columns in Z	0
Subjects	34
Max Obs per Subject	3

Number of Observations	
Number of Observations Read	102
Number of Observations Used	102
Number of Observations Not Used	0

Iteration History			
Iteration	Evaluations	-2 Res Log Like	Criterion
0	1	874.65103340	
1	1	833.06102087	0.00000000

Convergence criteria met.

Covariance Parameter Estimates		
Cov Parm	Subject	Estimate
CS	ParticipantID	316.22
Residual		163.07

Fit Statistics	
-2 Res Log Likelihood	833.1
AIC (Smaller is Better)	837.1
AICC (Smaller is Better)	837.2
BIC (Smaller is Better)	840.1

Null Model Likelihood Ratio Test		
DF	Chi-Square	Pr > ChiSq
1	41.59	<.0001

Solution for Fixed Effects								
Effect	Group	Gender	GameVisit	Estimate	Standard Error	DF	t Value	Pr > |t|
Intercept				22.2406	6.1279	45.1	3.63	0.0007
GameVisit			1	-0.6250	4.5148	64	-0.14	0.8903
GameVisit			2	9.9375	4.5148	64	2.20	0.0313
GameVisit			3	0	.	.	.	.
Group	Vegetable			-1.0054	7.6055	49.3	-0.13	0.8954
Group	Control			0	.	.	.	.
GameVisit*Group	Vegetable		1	1.0139	6.2050	64	0.16	0.8707
GameVisit*Group	Control		1	0	.	.	.	.
GameVisit*Group	Vegetable		2	-2.7708	6.2050	64	-0.45	0.6567

Solution for Fixed Effects								
Effect	Group	Gender	GameVisit	Estimate	Standard Error	DF	t Value	Pr > |t|
GameVisit*Group	Control		2	0	.	.	.	.
GameVisit*Group	Vegetable		3	0	.	.	.	.
GameVisit*Group	Control		3	0	.	.	.	.
Gender		Man		-5.3084	7.3492	31	-0.72	0.4755
Gender		Woman		0	.	.	.	.

Type 3 Tests of Fixed Effects				
Effect	Num DF	Den DF	F Value	Pr > F
GameVisit	2	64	5.14	0.0085
Group	1	31	0.06	0.8141
GameVisit*Group	2	64	0.20	0.8197
Gender	1	31	0.52	0.4755

Least Squares Means Estimate						
Effect	Label	Estimate	Standard Error	DF	t Value	Pr > |t|
GameVisit*Group	Control, GV3-GV1	0.6250	4.5148	64	0.14	0.8903

Least Squares Means Estimate						
Effect	Label	Estimate	Standard Error	DF	t Value	Pr > |t|
GameVisit*Group	Control, GV2-GV1	10.5625	4.5148	64	2.34	0.0224

Least Squares Means Estimate						
Effect	Label	Estimate	Standard Error	DF	t Value	Pr > |t|
GameVisit*Group	Veg, GV3-GV1	-0.3889	4.2566	64	-0.09	0.9275

Least Squares Means Estimate						
Effect	Label	Estimate	Standard Error	DF	t Value	Pr > |t|
GameVisit*Group	Veg, GV2-GV1	6.7778	4.2566	64	1.59	0.1162

Least Squares Means Estimate						
Effect	Label	Estimate	Standard Error	DF	t Value	Pr > |t|
GameVisit*Group	Veg - Control, GV1	0.008444	7.6055	49.26	0.00	0.9991

Least Squares Means Estimate						
Effect	Label	Estimate	Standard Error	DF	t Value	Pr > |t|
GameVisit*Group	Veg - Control, GV2	-3.7763	7.6055	49.26	-0.50	0.6217

Least Squares Means Estimate						
Effect	Label	Estimate	Standard Error	DF	t Value	Pr > |t|
GameVisit*Group	Veg - Control, GV3	-1.0054	7.6055	49.26	-0.13	0.8954

Least Squares Means							
Effect	Group	GameVisit	Estimate	Standard Error	DF	t Value	Pr > |t|

Least Squares Means							
Effect	Group	GameVisit	Estimate	Standard Error	DF	t Value	Pr > |t|
Group	Vegetable		21.0995	4.9754	31	4.24	0.0002
Group	Control		22.6906	4.8995	31	4.63	<.0001
GameVisit		1	18.9657	4.0418	46.7	4.69	<.0001
GameVisit		2	27.6358	4.0418	46.7	6.84	<.0001
GameVisit		3	19.0837	4.0418	46.7	4.72	<.0001
GameVisit*Group	Vegetable	1	18.9699	5.5493	46.6	3.42	0.0013
GameVisit*Group	Control	1	18.9615	5.5497	49.1	3.42	0.0013
GameVisit*Group	Vegetable	2	25.7477	5.5493	46.6	4.64	<.0001
GameVisit*Group	Control	2	29.5240	5.5497	49.1	5.32	<.0001
GameVisit*Group	Vegetable	3	18.5810	5.5493	46.6	3.35	0.0016
GameVisit*Group	Control	3	19.5865	5.5497	49.1	3.53	0.0009

Differences of Least Squares Means									
Effect	Group	GameVisit	_Group	_GameVisit	Estimate	Standard Error	DF	t Value	Pr > |t|
Group	Vegetable		Control		-1.5911	6.7089	31	-0.24	0.8141
GameVisit		1		2	-8.6701	3.1025	64	-2.79	0.0068
GameVisit		1		3	-0.1181	3.1025	64	-0.04	0.9698
GameVisit		2		3	8.5521	3.1025	64	2.76	0.0076
GameVisit*Group	Vegetable	1	Control	1	0.008444	7.6055	49.3	0.00	0.9991
GameVisit*Group	Vegetable	1	Vegetable	2	-6.7778	4.2566	64	-1.59	0.1162
GameVisit*Group	Vegetable	1	Control	2	-10.5541	7.6055	49.3	-1.39	0.1715
GameVisit*Group	Vegetable	1	Vegetable	3	0.3889	4.2566	64	0.09	0.9275
GameVisit*Group	Vegetable	1	Control	3	-0.6166	7.6055	49.3	-0.08	0.9357
GameVisit*Group	Control	1	Vegetable	2	-6.7862	7.6055	49.3	-0.89	0.3766
GameVisit*Group	Control	1	Control	2	-10.5625	4.5148	64	-2.34	0.0224
GameVisit*Group	Control	1	Vegetable	3	0.3804	7.6055	49.3	0.05	0.9603
GameVisit*Group	Control	1	Control	3	-0.6250	4.5148	64	-0.14	0.8903
GameVisit*Group	Vegetable	2	Control	2	-3.7763	7.6055	49.3	-0.50	0.6217
GameVisit*Group	Vegetable	2	Vegetable	3	7.1667	4.2566	64	1.68	0.0971
GameVisit*Group	Vegetable	2	Control	3	6.1612	7.6055	49.3	0.81	0.4218
GameVisit*Group	Control	2	Vegetable	3	10.9429	7.6055	49.3	1.44	0.1565
GameVisit*Group	Control	2	Control	3	9.9375	4.5148	64	2.20	0.0313
GameVisit*Group	Vegetable	3	Control	3	-1.0054	7.6055	49.3	-0.13	0.8954

OSF planned analysis: Bitterness

The Mixed Procedure

SampleName=Broccoli chopped

Model Information	
Data Set	WORK.GAMEVISITS

Model Information	
Dependent Variable	Bitterness
Covariance Structure	Compound Symmetry
Subject Effect	ParticipantID
Estimation Method	REML
Residual Variance Method	Profile
Fixed Effects SE Method	Kenward-Roger
Degrees of Freedom Method	Kenward-Roger

Class Level Information		
Class	Levels	Values
ParticipantID	34	1001 1002 1005 1006 1009 1010 1014 1016 1019 1020 1024 1025 1027 1029 1030 1034 1035 1041 1045 1046 1048 1049 1050 1051 1056 1061 1063 1064 1065 1066 1068 1069 1074 1078
GameVisit	3	1 2 3
Group	2	Vegetable Control
Gender	2	Man Woman
SampleName	1	Broccoli chopped

Dimensions	
Covariance Parameters	2
Columns in X	14
Columns in Z	0
Subjects	34
Max Obs per Subject	3

Number of Observations	
Number of Observations Read	102
Number of Observations Used	102
Number of Observations Not Used	0

Iteration History			
Iteration	Evaluations	-2 Res Log Like	Criterion
0	1	856.32239667	
1	1	789.21384109	0.00000000

Convergence criteria met.

Covariance Parameter Estimates		
Cov Parm	Subject	Estimate
CS	ParticipantID	310.42
Residual		85.8099

Fit Statistics	
-2 Res Log Likelihood	789.2
AIC (Smaller is Better)	793.2
AICC (Smaller is Better)	793.3

Fit Statistics	
BIC (Smaller is Better)	796.3

Null Model Likelihood Ratio Test		
DF	Chi-Square	Pr > ChiSq
1	67.11	<.0001

Solution for Fixed Effects

Effect	Group	Gender	GameVisit	Estimate	Standard Error	DF	t Value	Pr > |t|
Intercept				21.8892	5.6315	39.1	3.89	0.0004
GameVisit			1	1.1250	3.2751	64	0.34	0.7323
GameVisit			2	2.7500	3.2751	64	0.84	0.4042
GameVisit			3	0	.	.	.	.
Group	Vegetable			-0.1215	6.9232	41.5	-0.02	0.9861
Group	Control			0	.	.	.	.
GameVisit*Group	Vegetable		1	-3.4583	4.5012	64	-0.77	0.4451
GameVisit*Group	Control		1	0	.	.	.	.
GameVisit*Group	Vegetable		2	1.7500	4.5012	64	0.39	0.6987
GameVisit*Group	Control		2	0	.	.	.	.
GameVisit*Group	Vegetable		3	0	.	.	.	.
GameVisit*Group	Control		3	0	.	.	.	.
Gender		Man		6.7955	7.0294	31	0.97	0.3412
Gender		Woman		0	.	.	.	.

Type 3 Tests of Fixed Effects

Effect	Num DF	Den DF	F Value	Pr > F
GameVisit	2	64	2.07	0.1351
Group	1	31	0.01	0.9149
GameVisit*Group	2	64	0.69	0.5036
Gender	1	31	0.93	0.3412

Least Squares Means Estimate

Effect	Label	Estimate	Standard Error	DF	t Value	Pr > |t|
GameVisit*Group	Control, GV3-GV1	-1.1250	3.2751	64	-0.34	0.7323

Least Squares Means Estimate

Effect	Label	Estimate	Standard Error	DF	t Value	Pr > |t|
GameVisit*Group	Control, GV2-GV1	1.6250	3.2751	64	0.50	0.6215

Least Squares Means Estimate

Effect	Label	Estimate	Standard Error	DF	t Value	Pr > |t|
GameVisit*Group	Veg, GV3-GV1	2.3333	3.0878	64	0.76	0.4526

Least Squares Means Estimate

Effect	Label	Estimate	Standard Error	DF	t Value	Pr > |t|

Least Squares Means Estimate						
Effect	Label	Estimate	Standard Error	DF	t Value	Pr > |t|
GameVisit*Group	Veg, GV2-GV1	6.8333	3.0878	64	2.21	0.0305

Least Squares Means Estimate						
Effect	Label	Estimate	Standard Error	DF	t Value	Pr > |t|
GameVisit*Group	Veg - Control, GV1	-3.5798	6.9232	41.46	-0.52	0.6078

Least Squares Means Estimate						
Effect	Label	Estimate	Standard Error	DF	t Value	Pr > |t|
GameVisit*Group	Veg - Control, GV2	1.6285	6.9232	41.46	0.24	0.8152

Least Squares Means Estimate						
Effect	Label	Estimate	Standard Error	DF	t Value	Pr > |t|
GameVisit*Group	Veg - Control, GV3	-0.1215	6.9232	41.46	-0.02	0.9861

Least Squares Means							
Effect	Group	GameVisit	Estimate	Standard Error	DF	t Value	Pr > |t|
Group	Vegetable		25.8877	4.7590	31	5.44	<.0001
Group	Control		26.5786	4.6863	31	5.67	<.0001
GameVisit		1	24.6220	3.7011	39.9	6.65	<.0001
GameVisit		2	28.8512	3.7011	39.9	7.80	<.0001
GameVisit		3	25.2262	3.7011	39.9	6.82	<.0001
GameVisit*Group	Vegetable	1	22.8321	5.0819	39.9	4.49	<.0001
GameVisit*Group	Control	1	26.4119	5.0534	41.4	5.23	<.0001
GameVisit*Group	Vegetable	2	29.6654	5.0819	39.9	5.84	<.0001
GameVisit*Group	Control	2	28.0369	5.0534	41.4	5.55	<.0001
GameVisit*Group	Vegetable	3	25.1654	5.0819	39.9	4.95	<.0001
GameVisit*Group	Control	3	25.2869	5.0534	41.4	5.00	<.0001

Differences of Least Squares Means									
Effect	Group	GameVisit	_Group	_GameVisit	Estimate	Standard Error	DF	t Value	Pr > |t|
Group	Vegetable		Control		-0.6910	6.4170	31	-0.11	0.9149
GameVisit		1		2	-4.2292	2.2506	64	-1.88	0.0648
GameVisit		1		3	-0.6042	2.2506	64	-0.27	0.7892
GameVisit		2		3	3.6250	2.2506	64	1.61	0.1122
GameVisit*Group	Vegetable	1	Control	1	-3.5798	6.9232	41.5	-0.52	0.6078
GameVisit*Group	Vegetable	1	Vegetable	2	-6.8333	3.0878	64	-2.21	0.0305
GameVisit*Group	Vegetable	1	Control	2	-5.2048	6.9232	41.5	-0.75	0.4564
GameVisit*Group	Vegetable	1	Vegetable	3	-2.3333	3.0878	64	-0.76	0.4526
GameVisit*Group	Vegetable	1	Control	3	-2.4548	6.9232	41.5	-0.35	0.7247
GameVisit*Group	Control	1	Vegetable	2	-3.2535	6.9232	41.5	-0.47	0.6409
GameVisit*Group	Control	1	Control	2	-1.6250	3.2751	64	-0.50	0.6215
GameVisit*Group	Control	1	Vegetable	3	1.2465	6.9232	41.5	0.18	0.8580

Differences of Least Squares Means									
Effect	Group	GameVisit	_Group	_GameVisit	Estimate	Standard Error	DF	t Value	Pr > |t|
GameVisit*Group	Control	1	Control	3	1.1250	3.2751	64	0.34	0.7323
GameVisit*Group	Vegetable	2	Control	2	1.6285	6.9232	41.5	0.24	0.8152
GameVisit*Group	Vegetable	2	Vegetable	3	4.5000	3.0878	64	1.46	0.1499
GameVisit*Group	Vegetable	2	Control	3	4.3785	6.9232	41.5	0.63	0.5306
GameVisit*Group	Control	2	Vegetable	3	2.8715	6.9232	41.5	0.41	0.6805
GameVisit*Group	Control	2	Control	3	2.7500	3.2751	64	0.84	0.4042
GameVisit*Group	Vegetable	3	Control	3	-0.1215	6.9232	41.5	-0.02	0.9861

OSF planned analysis: Bitterness

The Mixed Procedure

SampleName=Broccoli puree

Model Information	
Data Set	WORK.GAMEVISITS
Dependent Variable	Bitterness
Covariance Structure	Compound Symmetry
Subject Effect	ParticipantID
Estimation Method	REML
Residual Variance Method	Profile
Fixed Effects SE Method	Kenward-Roger
Degrees of Freedom Method	Kenward-Roger

Class Level Information		
Class	Levels	Values
ParticipantID	34	1001 1002 1005 1006 1009 1010 1014 1016 1019 1020 1024 1025 1027 1029 1030 1034 1035 1041 1045 1046 1048 1049 1050 1051 1056 1061 1063 1064 1065 1066 1068 1069 1074 1078
GameVisit	3	1 2 3
Group	2	Vegetable Control
Gender	2	Man Woman
SampleName	1	Broccoli puree

Dimensions	
Covariance Parameters	2
Columns in X	14
Columns in Z	0
Subjects	34
Max Obs per Subject	3

Number of Observations	
Number of Observations Read	102
Number of Observations Used	102

Number of Observations	
Number of Observations Not Used	0

Iteration History			
Iteration	Evaluations	-2 Res Log Like	Criterion
0	1	857.18336551	
1	1	820.50977360	0.00000000

Convergence criteria met.

Covariance Parameter Estimates		
Cov Parm	Subject	Estimate
CS	ParticipantID	249.91
Residual		148.60

Fit Statistics	
-2 Res Log Likelihood	820.5
AIC (Smaller is Better)	824.5
AICC (Smaller is Better)	824.6
BIC (Smaller is Better)	827.6

Null Model Likelihood Ratio Test		
DF	Chi-Square	Pr > ChiSq
1	36.67	<.0001

Solution for Fixed Effects								
Effect	Group	Gender	GameVisit	Estimate	Standard Error	DF	t Value	Pr > |t|
Intercept				18.3530	5.5717	47	3.29	0.0019
GameVisit			1	3.5625	4.3099	64	0.83	0.4115
GameVisit			2	2.5000	4.3099	64	0.58	0.5639
GameVisit			3	0	.	.	.	.
Group	Vegetable			5.0043	6.9329	51.6	0.72	0.4737
Group	Control			0	.	.	.	.
GameVisit*Group	Vegetable		1	-2.6736	5.9234	64	-0.45	0.6533
GameVisit*Group	Control		1	0	.	.	.	.
GameVisit*Group	Vegetable		2	-3.8333	5.9234	64	-0.65	0.5198
GameVisit*Group	Control		2	0	.	.	.	.
GameVisit*Group	Vegetable		3	0	.	.	.	.
GameVisit*Group	Control		3	0	.	.	.	.
Gender		Man		-2.1080	6.6063	31	-0.32	0.7518
Gender		Woman		0	.	.	.	.

Type 3 Tests of Fixed Effects				
Effect	Num DF	Den DF	F Value	Pr > F
GameVisit	2	64	0.30	0.7392

Type 3 Tests of Fixed Effects				
Effect	Num DF	Den DF	F Value	Pr > F
Group	1	31	0.22	0.6415
GameVisit*Group	2	64	0.22	0.8029
Gender	1	31	0.10	0.7518

Least Squares Means Estimate						
Effect	Label	Estimate	Standard Error	DF	t Value	Pr > |t|
GameVisit*Group	Control, GV3-GV1	-3.5625	4.3099	64	-0.83	0.4115

Least Squares Means Estimate						
Effect	Label	Estimate	Standard Error	DF	t Value	Pr > |t|
GameVisit*Group	Control, GV2-GV1	-1.0625	4.3099	64	-0.25	0.8061

Least Squares Means Estimate						
Effect	Label	Estimate	Standard Error	DF	t Value	Pr > |t|
GameVisit*Group	Veg, GV3-GV1	-0.8889	4.0634	64	-0.22	0.8275

Least Squares Means Estimate						
Effect	Label	Estimate	Standard Error	DF	t Value	Pr > |t|
GameVisit*Group	Veg, GV2-GV1	-2.2222	4.0634	64	-0.55	0.5864

Least Squares Means Estimate						
Effect	Label	Estimate	Standard Error	DF	t Value	Pr > |t|
GameVisit*Group	Veg - Control, GV1	2.3307	6.9329	51.56	0.34	0.7381

Least Squares Means Estimate						
Effect	Label	Estimate	Standard Error	DF	t Value	Pr > |t|
GameVisit*Group	Veg - Control, GV2	1.1710	6.9329	51.56	0.17	0.8665

Least Squares Means Estimate						
Effect	Label	Estimate	Standard Error	DF	t Value	Pr > |t|
GameVisit*Group	Veg - Control, GV3	5.0043	6.9329	51.56	0.72	0.4737

Least Squares Means							
Effect	Group	GameVisit	Estimate	Standard Error	DF	t Value	Pr > |t|
Group	Vegetable		22.1552	4.4725	31	4.95	<.0001
Group	Control		19.3198	4.4042	31	4.39	0.0001
GameVisit		1	22.0269	3.6785	48.7	5.99	<.0001
GameVisit		2	20.3845	3.6785	48.7	5.54	<.0001
GameVisit		3	19.8012	3.6785	48.7	5.38	<.0001
GameVisit*Group	Vegetable	1	23.1922	5.0504	48.6	4.59	<.0001
GameVisit*Group	Control	1	20.8615	5.0585	51.4	4.12	0.0001
GameVisit*Group	Vegetable	2	20.9700	5.0504	48.6	4.15	0.0001
GameVisit*Group	Control	2	19.7990	5.0585	51.4	3.91	0.0003

Least Squares Means							
**Effect**	**Group**	**GameVisit**	**Estimate**	**Standard Error**	**DF**	**t Value**	**Pr > |t|**
**GameVisit*Group**	**Vegetable**	**3**	22.3033	5.0504	48.6	4.42	<.0001
**GameVisit*Group**	**Control**	**3**	17.2990	5.0585	51.4	3.42	0.0012

Differences of Least Squares Means									
**Effect**	**Group**	**GameVisit**	**_Group**	**_GameVisit**	**Estimate**	**Standard Error**	**DF**	**t Value**	**Pr > |t|**
**Group**	**Vegetable**		**Control**		2.8354	6.0307	31	0.47	0.6415
**GameVisit**		**1**		**2**	1.6424	2.9617	64	0.55	0.5811
**GameVisit**		**1**		**3**	2.2257	2.9617	64	0.75	0.4551
**GameVisit**		**2**		**3**	0.5833	2.9617	64	0.20	0.8445
**GameVisit*Group**	**Vegetable**	**1**	**Control**	**1**	2.3307	6.9329	51.6	0.34	0.7381
**GameVisit*Group**	**Vegetable**	**1**	**Vegetable**	**2**	2.2222	4.0634	64	0.55	0.5864
**GameVisit*Group**	**Vegetable**	**1**	**Control**	**2**	3.3932	6.9329	51.6	0.49	0.6266
**GameVisit*Group**	**Vegetable**	**1**	**Vegetable**	**3**	0.8889	4.0634	64	0.22	0.8275
**GameVisit*Group**	**Vegetable**	**1**	**Control**	**3**	5.8932	6.9329	51.6	0.85	0.3992
**GameVisit*Group**	**Control**	**1**	**Vegetable**	**2**	-0.1085	6.9329	51.6	-0.02	0.9876
**GameVisit*Group**	**Control**	**1**	**Control**	**2**	1.0625	4.3099	64	0.25	0.8061
**GameVisit*Group**	**Control**	**1**	**Vegetable**	**3**	-1.4418	6.9329	51.6	-0.21	0.8361
**GameVisit*Group**	**Control**	**1**	**Control**	**3**	3.5625	4.3099	64	0.83	0.4115
**GameVisit*Group**	**Vegetable**	**2**	**Control**	**2**	1.1710	6.9329	51.6	0.17	0.8665
**GameVisit*Group**	**Vegetable**	**2**	**Vegetable**	**3**	-1.3333	4.0634	64	-0.33	0.7439
**GameVisit*Group**	**Vegetable**	**2**	**Control**	**3**	3.6710	6.9329	51.6	0.53	0.5987
**GameVisit*Group**	**Control**	**2**	**Vegetable**	**3**	-2.5043	6.9329	51.6	-0.36	0.7194
**GameVisit*Group**	**Control**	**2**	**Control**	**3**	2.5000	4.3099	64	0.58	0.5639
**GameVisit*Group**	**Vegetable**	**3**	**Control**	**3**	5.0043	6.9329	51.6	0.72	0.4737

OSF planned analysis: Bitterness

The Mixed Procedure

SampleName=Chicken puree

Model Information	
**Data Set**	WORK.GAMEVISITS
**Dependent Variable**	Bitterness
**Covariance Structure**	Compound Symmetry
**Subject Effect**	ParticipantID
**Estimation Method**	REML
**Residual Variance Method**	Profile
**Fixed Effects SE Method**	Kenward-Roger
**Degrees of Freedom Method**	Kenward-Roger

Class Level Information		
**Class**	**Levels**	**Values**

Class Level Information		
Class	Levels	Values
ParticipantID	34	1001 1002 1005 1006 1009 1010 1014 1016 1019 1020 1024 1025 1027 1029 1030 1034 1035 1041 1045 1046 1048 1049 1050 1051 1056 1061 1063 1064 1065 1066 1068 1069 1074 1078
GameVisit	3	1 2 3
Group	2	Vegetable Control
Gender	2	Man Woman
SampleName	1	Chicken puree

Dimensions	
Covariance Parameters	2
Columns in X	14
Columns in Z	0
Subjects	34
Max Obs per Subject	3

Number of Observations	
Number of Observations Read	102
Number of Observations Used	102
Number of Observations Not Used	0

Iteration History			
Iteration	Evaluations	-2 Res Log Like	Criterion
0	1	917.14009376	
1	1	825.94710162	0.00000000

Convergence criteria met.

Covariance Parameter Estimates		
Cov Parm	Subject	Estimate
CS	ParticipantID	644.03
Residual		108.72

Fit Statistics	
-2 Res Log Likelihood	825.9
AIC (Smaller is Better)	829.9
AICC (Smaller is Better)	830.1
BIC (Smaller is Better)	833.0

Null Model Likelihood Ratio Test		
DF	Chi-Square	Pr > ChiSq
1	91.19	<.0001

Solution for Fixed Effects								
Effect	Group	Gender	GameVisit	Estimate	Standard Error	DF	t Value	Pr > |t|
Intercept				24.5984	7.8096	36.1	3.15	0.0033

Solution for Fixed Effects								
Effect	Group	Gender	GameVisit	Estimate	Standard Error	DF	t Value	Pr > |t|
GameVisit			1	0.1875	3.6864	64	0.05	0.9596
GameVisit			2	4.7500	3.6864	64	1.29	0.2022
GameVisit			3	0	.	.	.	.
Group	Vegetable			5.3303	9.5489	37.6	0.56	0.5800
Group	Control			0	.	.	.	.
GameVisit*Group	Vegetable		1	-5.2986	5.0665	64	-1.05	0.2996
GameVisit*Group	Control		1	0	.	.	.	.
GameVisit*Group	Vegetable		2	-7.3056	5.0665	64	-1.44	0.1542
GameVisit*Group	Control		2	0	.	.	.	.
GameVisit*Group	Vegetable		3	0	.	.	.	.
GameVisit*Group	Control		3	0	.	.	.	.
Gender		Man		3.0709	9.9574	31	0.31	0.7598
Gender		Woman		0	.	.	.	.

Type 3 Tests of Fixed Effects				
Effect	Num DF	Den DF	F Value	Pr > F
GameVisit	2	64	1.04	0.3610
Group	1	31	0.02	0.9020
GameVisit*Group	2	64	1.11	0.3358
Gender	1	31	0.10	0.7598

Least Squares Means Estimate						
Effect	Label	Estimate	Standard Error	DF	t Value	Pr > |t|
GameVisit*Group	Control, GV3-GV1	-0.1875	3.6864	64	-0.05	0.9596

Least Squares Means Estimate						
Effect	Label	Estimate	Standard Error	DF	t Value	Pr > |t|
GameVisit*Group	Control, GV2-GV1	4.5625	3.6864	64	1.24	0.2204

Least Squares Means Estimate						
Effect	Label	Estimate	Standard Error	DF	t Value	Pr > |t|
GameVisit*Group	Veg, GV3-GV1	5.1111	3.4756	64	1.47	0.1463

Least Squares Means Estimate						
Effect	Label	Estimate	Standard Error	DF	t Value	Pr > |t|
GameVisit*Group	Veg, GV2-GV1	2.5556	3.4756	64	0.74	0.4649

Least Squares Means Estimate						
Effect	Label	Estimate	Standard Error	DF	t Value	Pr > |t|
GameVisit*Group	Veg - Control, GV1	0.03166	9.5489	37.56	0.00	0.9974

Least Squares Means Estimate						
Effect	Label	Estimate	Standard Error	DF	t Value	Pr > |t|

Least Squares Means Estimate						
Effect	Label	Estimate	Standard Error	DF	t Value	Pr > |t|
GameVisit*Group	Veg - Control, GV2	-1.9753	9.5489	37.56	-0.21	0.8372

Least Squares Means Estimate						
Effect	Label	Estimate	Standard Error	DF	t Value	Pr > |t|
GameVisit*Group	Veg - Control, GV3	5.3303	9.5489	37.56	0.56	0.5800

Least Squares Means							
Effect	Group	GameVisit	Estimate	Standard Error	DF	t Value	Pr > |t|
Group	Vegetable		28.9086	6.7412	31	4.29	0.0002
Group	Control		27.7797	6.6383	31	4.18	0.0002
GameVisit		1	26.3372	5.1223	36.6	5.14	<.0001
GameVisit		2	29.8962	5.1223	36.6	5.84	<.0001
GameVisit		3	28.7990	5.1223	36.6	5.62	<.0001
GameVisit*Group	Vegetable	1	26.3530	7.0335	36.6	3.75	0.0006
GameVisit*Group	Control	1	26.3214	6.9711	37.5	3.78	0.0006
GameVisit*Group	Vegetable	2	28.9086	7.0335	36.6	4.11	0.0002
GameVisit*Group	Control	2	30.8839	6.9711	37.5	4.43	<.0001
GameVisit*Group	Vegetable	3	31.4641	7.0335	36.6	4.47	<.0001
GameVisit*Group	Control	3	26.1339	6.9711	37.5	3.75	0.0006

Differences of Least Squares Means									
Effect	Group	GameVisit	_Group	_GameVisit	Estimate	Standard Error	DF	t Value	Pr > |t|
Group	Vegetable		Control		1.1289	9.0898	31	0.12	0.9020
GameVisit		1		2	-3.5590	2.5333	64	-1.40	0.1649
GameVisit		1		3	-2.4618	2.5333	64	-0.97	0.3348
GameVisit		2		3	1.0972	2.5333	64	0.43	0.6664
GameVisit*Group	Vegetable	1	Control	1	0.03166	9.5489	37.6	0.00	0.9974
GameVisit*Group	Vegetable	1	Vegetable	2	-2.5556	3.4756	64	-0.74	0.4649
GameVisit*Group	Vegetable	1	Control	2	-4.5308	9.5489	37.6	-0.47	0.6379
GameVisit*Group	Vegetable	1	Vegetable	3	-5.1111	3.4756	64	-1.47	0.1463
GameVisit*Group	Vegetable	1	Control	3	0.2192	9.5489	37.6	0.02	0.9818
GameVisit*Group	Control	1	Vegetable	2	-2.5872	9.5489	37.6	-0.27	0.7879
GameVisit*Group	Control	1	Control	2	-4.5625	3.6864	64	-1.24	0.2204
GameVisit*Group	Control	1	Vegetable	3	-5.1428	9.5489	37.6	-0.54	0.5934
GameVisit*Group	Control	1	Control	3	0.1875	3.6864	64	0.05	0.9596
GameVisit*Group	Vegetable	2	Control	2	-1.9753	9.5489	37.6	-0.21	0.8372
GameVisit*Group	Vegetable	2	Vegetable	3	-2.5556	3.4756	64	-0.74	0.4649
GameVisit*Group	Vegetable	2	Control	3	2.7747	9.5489	37.6	0.29	0.7730
GameVisit*Group	Control	2	Vegetable	3	-0.5803	9.5489	37.6	-0.06	0.9519
GameVisit*Group	Control	2	Control	3	4.7500	3.6864	64	1.29	0.2022
GameVisit*Group	Vegetable	3	Control	3	5.3303	9.5489	37.6	0.56	0.5800

OSF planned analysis: Bitterness

The Mixed Procedure

SampleName=Kale chopped

Model Information	
Data Set	WORK.GAMEVISITS
Dependent Variable	Bitterness
Covariance Structure	Compound Symmetry
Subject Effect	ParticipantID
Estimation Method	REML
Residual Variance Method	Profile
Fixed Effects SE Method	Kenward-Roger
Degrees of Freedom Method	Kenward-Roger

Class Level Information		
Class	Levels	Values
ParticipantID	34	1001 1002 1005 1006 1009 1010 1014 1016 1019 1020 1024 1025 1027 1029 1030 1034 1035 1041 1045 1046 1048 1049 1050 1051 1056 1061 1063 1064 1065 1066 1068 1069 1074 1078
GameVisit	3	1 2 3
Group	2	Vegetable Control
Gender	2	Man Woman
SampleName	1	Kale chopped

Dimensions	
Covariance Parameters	2
Columns in X	14
Columns in Z	0
Subjects	34
Max Obs per Subject	3

Number of Observations	
Number of Observations Read	102
Number of Observations Used	102
Number of Observations Not Used	0

Iteration History			
Iteration	Evaluations	-2 Res Log Like	Criterion
0	1	895.63360987	
1	1	858.74935690	0.00000000

Convergence criteria met.

Covariance Parameter Estimates		
Cov Parm	Subject	Estimate
CS	ParticipantID	375.48

Covariance Parameter Estimates		
Cov Parm	Subject	Estimate
Residual		221.87

Fit Statistics	
-2 Res Log Likelihood	858.7
AIC (Smaller is Better)	862.7
AICC (Smaller is Better)	862.9
BIC (Smaller is Better)	865.8

Null Model Likelihood Ratio Test		
DF	Chi-Square	Pr > ChiSq
1	36.88	<.0001

Solution for Fixed Effects								
Effect	Group	Gender	GameVisit	Estimate	Standard Error	DF	t Value	Pr > |t|
Intercept				43.4577	6.8225	46.9	6.37	<.0001
GameVisit			1	-4.1250	5.2662	64	-0.78	0.4363
GameVisit			2	1.3125	5.2662	64	0.25	0.8040
GameVisit			3	0	.	.	.	.
Group	Vegetable			-0.4457	8.4882	51.5	-0.05	0.9583
Group	Control			0	.	.	.	.
GameVisit*Group	Vegetable		1	3.7917	7.2378	64	0.52	0.6022
GameVisit*Group	Control		1	0	.	.	.	.
GameVisit*Group	Vegetable		2	-2.7014	7.2378	64	-0.37	0.7102
GameVisit*Group	Control		2	0	.	.	.	.
GameVisit*Group	Vegetable		3	0	.	.	.	.
GameVisit*Group	Control		3	0	.	.	.	.
Gender		Man		-0.5540	8.0935	31	-0.07	0.9459
Gender		Woman		0	.	.	.	.

Type 3 Tests of Fixed Effects				
Effect	Num DF	Den DF	F Value	Pr > F
GameVisit	2	64	0.25	0.7806
Group	1	31	0.00	0.9912
GameVisit*Group	2	64	0.41	0.6679
Gender	1	31	0.00	0.9459

Least Squares Means Estimate						
Effect	Label	Estimate	Standard Error	DF	t Value	Pr > |t|
GameVisit*Group	Control, GV3-GV1	4.1250	5.2662	64	0.78	0.4363

Least Squares Means Estimate						
Effect	Label	Estimate	Standard Error	DF	t Value	Pr > |t|

Least Squares Means Estimate						
Effect	Label	Estimate	Standard Error	DF	t Value	Pr > |t|
GameVisit*Group	Control, GV2-GV1	5.4375	5.2662	64	1.03	0.3057

Least Squares Means Estimate						
Effect	Label	Estimate	Standard Error	DF	t Value	Pr > |t|
GameVisit*Group	Veg, GV3-GV1	0.3333	4.9651	64	0.07	0.9467

Least Squares Means Estimate						
Effect	Label	Estimate	Standard Error	DF	t Value	Pr > |t|
GameVisit*Group	Veg, GV2-GV1	-1.0556	4.9651	64	-0.21	0.8323

Least Squares Means Estimate						
Effect	Label	Estimate	Standard Error	DF	t Value	Pr > |t|
GameVisit*Group	Veg - Control, GV1	3.3459	8.4882	51.45	0.39	0.6951

Least Squares Means Estimate						
Effect	Label	Estimate	Standard Error	DF	t Value	Pr > |t|
GameVisit*Group	Veg - Control, GV2	-3.1471	8.4882	51.45	-0.37	0.7123

Least Squares Means Estimate						
Effect	Label	Estimate	Standard Error	DF	t Value	Pr > |t|
GameVisit*Group	Veg - Control, GV3	-0.4457	8.4882	51.45	-0.05	0.9583

Least Squares Means							
Effect	Group	GameVisit	Estimate	Standard Error	DF	t Value	Pr > |t|
Group	Vegetable		42.1609	5.4793	31	7.69	<.0001
Group	Control		42.2433	5.3957	31	7.83	<.0001
GameVisit		1	40.7287	4.5041	48.6	9.04	<.0001
GameVisit		2	42.9197	4.5041	48.6	9.53	<.0001
GameVisit		3	42.9579	4.5041	48.6	9.54	<.0001
GameVisit*Group	Vegetable	1	42.4017	6.1839	48.5	6.86	<.0001
GameVisit*Group	Control	1	39.0558	6.1934	51.3	6.31	<.0001
GameVisit*Group	Vegetable	2	41.3461	6.1839	48.5	6.69	<.0001
GameVisit*Group	Control	2	44.4933	6.1934	51.3	7.18	<.0001
GameVisit*Group	Vegetable	3	42.7350	6.1839	48.5	6.91	<.0001
GameVisit*Group	Control	3	43.1808	6.1934	51.3	6.97	<.0001

Differences of Least Squares Means									
Effect	Group	GameVisit	_Group	_GameVisit	Estimate	Standard Error	DF	t Value	Pr > |t|
Group	Vegetable		Control		-0.08232	7.3883	31	-0.01	0.9912
GameVisit		1		2	-2.1910	3.6189	64	-0.61	0.5470
GameVisit		1		3	-2.2292	3.6189	64	-0.62	0.5401
GameVisit		2		3	-0.03819	3.6189	64	-0.01	0.9916
GameVisit*Group	Vegetable	1	Control	1	3.3459	8.4882	51.5	0.39	0.6951

Differences of Least Squares Means									
**Effect**	**Group**	**GameVisit**	**_Group**	**_GameVisit**	**Estimate**	**Standard Error**	**DF**	**t Value**	**Pr > |t|**
GameVisit*Group	Vegetable	1	Vegetable	2	1.0556	4.9651	64	0.21	0.8323
GameVisit*Group	Vegetable	1	Control	2	-2.0916	8.4882	51.5	-0.25	0.8063
GameVisit*Group	Vegetable	1	Vegetable	3	-0.3333	4.9651	64	-0.07	0.9467
GameVisit*Group	Vegetable	1	Control	3	-0.7791	8.4882	51.5	-0.09	0.9272
GameVisit*Group	Control	1	Vegetable	2	-2.2904	8.4882	51.5	-0.27	0.7884
GameVisit*Group	Control	1	Control	2	-5.4375	5.2662	64	-1.03	0.3057
GameVisit*Group	Control	1	Vegetable	3	-3.6793	8.4882	51.5	-0.43	0.6665
GameVisit*Group	Control	1	Control	3	-4.1250	5.2662	64	-0.78	0.4363
GameVisit*Group	Vegetable	2	Control	2	-3.1471	8.4882	51.5	-0.37	0.7123
GameVisit*Group	Vegetable	2	Vegetable	3	-1.3889	4.9651	64	-0.28	0.7806
GameVisit*Group	Vegetable	2	Control	3	-1.8346	8.4882	51.5	-0.22	0.8297
GameVisit*Group	Control	2	Vegetable	3	1.7582	8.4882	51.5	0.21	0.8367
GameVisit*Group	Control	2	Control	3	1.3125	5.2662	64	0.25	0.8040
GameVisit*Group	Vegetable	3	Control	3	-0.4457	8.4882	51.5	-0.05	0.9583

OSF planned analysis: Bitterness

The Mixed Procedure

SampleName=Kale puree

Model Information	
**Data Set**	WORK.GAMEVISITS
**Dependent Variable**	Bitterness
**Covariance Structure**	Compound Symmetry
**Subject Effect**	ParticipantID
**Estimation Method**	REML
**Residual Variance Method**	Profile
**Fixed Effects SE Method**	Kenward-Roger
**Degrees of Freedom Method**	Kenward-Roger

Class Level Information		
**Class**	**Levels**	**Values**
ParticipantID	34	1001 1002 1005 1006 1009 1010 1014 1016 1019 1020 1024 1025 1027 1029 1030 1034 1035 1041 1045 1046 1048 1049 1050 1051 1056 1061 1063 1064 1065 1066 1068 1069 1074 1078
GameVisit	3	1 2 3
Group	2	Vegetable Control
Gender	2	Man Woman
SampleName	1	Kale puree

Dimensions	
**Covariance Parameters**	2
**Columns in X**	14
**Columns in Z**	0

Dimensions	
Subjects	34
Max Obs per Subject	3

Number of Observations	
Number of Observations Read	102
Number of Observations Used	102
Number of Observations Not Used	0

Iteration History			
Iteration	Evaluations	-2 Res Log Like	Criterion
0	1	888.97901474	
1	1	880.11082266	0.00000000

Convergence criteria met.

Covariance Parameter Estimates		
Cov Parm	Subject	Estimate
CS	ParticipantID	178.72
Residual		374.61

Fit Statistics	
-2 Res Log Likelihood	880.1
AIC (Smaller is Better)	884.1
AICC (Smaller is Better)	884.2
BIC (Smaller is Better)	887.2

Null Model Likelihood Ratio Test		
DF	Chi-Square	Pr > ChiSq
1	8.87	0.0029

Solution for Fixed Effects								
Effect	Group	Gender	GameVisit	Estimate	Standard Error	DF	t Value	Pr > |t|
Intercept				44.2874	6.3879	68.6	6.93	<.0001
GameVisit			1	-7.3125	6.8429	64	-1.07	0.2893
GameVisit			2	-1.6875	6.8429	64	-0.25	0.8060
GameVisit			3	0	.	.	.	.
Group	Vegetable			-2.5801	8.1459	76.7	-0.32	0.7523
Group	Control			0	.	.	.	.
GameVisit*Group	Vegetable		1	5.8681	9.4047	64	0.62	0.5349
GameVisit*Group	Control		1	0	.	.	.	.
GameVisit*Group	Vegetable		2	-5.0347	9.4047	64	-0.54	0.5943
GameVisit*Group	Control		2	0	.	.	.	.
GameVisit*Group	Vegetable		3	0	.	.	.	.
GameVisit*Group	Control		3	0	.	.	.	.

Solution for Fixed Effects								
Effect	Group	Gender	GameVisit	Estimate	Standard Error	DF	t Value	Pr > |t|
Gender		Man		-4.4332	6.6519	31	-0.67	0.5101
Gender		Woman		0	.	.	.	.

Type 3 Tests of Fixed Effects				
Effect	Num DF	Den DF	F Value	Pr > F
GameVisit	2	64	0.56	0.5763
Group	1	31	0.14	0.7072
GameVisit*Group	2	64	0.67	0.5136
Gender	1	31	0.44	0.5101

Least Squares Means Estimate						
Effect	Label	Estimate	Standard Error	DF	t Value	Pr > |t|
GameVisit*Group	Control, GV3-GV1	7.3125	6.8429	64	1.07	0.2893

Least Squares Means Estimate						
Effect	Label	Estimate	Standard Error	DF	t Value	Pr > |t|
GameVisit*Group	Control, GV2-GV1	5.6250	6.8429	64	0.82	0.4141

Least Squares Means Estimate						
Effect	Label	Estimate	Standard Error	DF	t Value	Pr > |t|
GameVisit*Group	Veg, GV3-GV1	1.4444	6.4516	64	0.22	0.8236

Least Squares Means Estimate						
Effect	Label	Estimate	Standard Error	DF	t Value	Pr > |t|
GameVisit*Group	Veg, GV2-GV1	-5.2778	6.4516	64	-0.82	0.4164

Least Squares Means Estimate						
Effect	Label	Estimate	Standard Error	DF	t Value	Pr > |t|
GameVisit*Group	Veg - Control, GV1	3.2880	8.1459	76.65	0.40	0.6876

Least Squares Means Estimate						
Effect	Label	Estimate	Standard Error	DF	t Value	Pr > |t|
GameVisit*Group	Veg - Control, GV2	-7.6148	8.1459	76.65	-0.93	0.3528

Least Squares Means Estimate						
Effect	Label	Estimate	Standard Error	DF	t Value	Pr > |t|
GameVisit*Group	Veg - Control, GV3	-2.5801	8.1459	76.65	-0.32	0.7523

Least Squares Means							
Effect	Group	GameVisit	Estimate	Standard Error	DF	t Value	Pr > |t|
Group	Vegetable		36.7686	4.5034	31	8.16	<.0001
Group	Control		39.0709	4.4346	31	8.81	<.0001
GameVisit		1	36.4023	4.2574	71.7	8.55	<.0001

Least Squares Means							
Effect	Group	GameVisit	Estimate	Standard Error	DF	t Value	Pr > |t|
GameVisit		2	36.5760	4.2574	71.7	8.59	<.0001
GameVisit		3	40.7808	4.2574	71.7	9.58	<.0001
GameVisit*Group	Vegetable	1	38.0463	5.8442	71.7	6.51	<.0001
GameVisit*Group	Control	1	34.7584	5.9392	76.4	5.85	<.0001
GameVisit*Group	Vegetable	2	32.7686	5.8442	71.7	5.61	<.0001
GameVisit*Group	Control	2	40.3834	5.9392	76.4	6.80	<.0001
GameVisit*Group	Vegetable	3	39.4908	5.8442	71.7	6.76	<.0001
GameVisit*Group	Control	3	42.0709	5.9392	76.4	7.08	<.0001

Differences of Least Squares Means									
Effect	Group	GameVisit	_Group	_GameVisit	Estimate	Standard Error	DF	t Value	Pr > |t|
Group	Vegetable		Control		-2.3023	6.0724	31	-0.38	0.7072
GameVisit		1		2	-0.1736	4.7024	64	-0.04	0.9707
GameVisit		1		3	-4.3785	4.7024	64	-0.93	0.3553
GameVisit		2		3	-4.2049	4.7024	64	-0.89	0.3746
GameVisit*Group	Vegetable	1	Control	1	3.2880	8.1459	76.7	0.40	0.6876
GameVisit*Group	Vegetable	1	Vegetable	2	5.2778	6.4516	64	0.82	0.4164
GameVisit*Group	Vegetable	1	Control	2	-2.3370	8.1459	76.7	-0.29	0.7750
GameVisit*Group	Vegetable	1	Vegetable	3	-1.4444	6.4516	64	-0.22	0.8236
GameVisit*Group	Vegetable	1	Control	3	-4.0245	8.1459	76.7	-0.49	0.6227
GameVisit*Group	Control	1	Vegetable	2	1.9898	8.1459	76.7	0.24	0.8077
GameVisit*Group	Control	1	Control	2	-5.6250	6.8429	64	-0.82	0.4141
GameVisit*Group	Control	1	Vegetable	3	-4.7324	8.1459	76.7	-0.58	0.5630
GameVisit*Group	Control	1	Control	3	-7.3125	6.8429	64	-1.07	0.2893
GameVisit*Group	Vegetable	2	Control	2	-7.6148	8.1459	76.7	-0.93	0.3528
GameVisit*Group	Vegetable	2	Vegetable	3	-6.7222	6.4516	64	-1.04	0.3014
GameVisit*Group	Vegetable	2	Control	3	-9.3023	8.1459	76.7	-1.14	0.2570
GameVisit*Group	Control	2	Vegetable	3	0.8926	8.1459	76.7	0.11	0.9130
GameVisit*Group	Control	2	Control	3	-1.6875	6.8429	64	-0.25	0.8060
GameVisit*Group	Vegetable	3	Control	3	-2.5801	8.1459	76.7	-0.32	0.7523

OSF planned analysis: Bitterness

The Mixed Procedure

SampleName=Oat puree

Model Information	
Data Set	WORK.GAMEVISITS
Dependent Variable	Bitterness
Covariance Structure	Compound Symmetry
Subject Effect	ParticipantID

Model Information	
Estimation Method	REML
Residual Variance Method	Profile
Fixed Effects SE Method	Kenward-Roger
Degrees of Freedom Method	Kenward-Roger

Class Level Information		
Class	Levels	Values
ParticipantID	34	1001 1002 1005 1006 1009 1010 1014 1016 1019 1020 1024 1025 1027 1029 1030 1034 1035 1041 1045 1046 1048 1049 1050 1051 1056 1061 1063 1064 1065 1066 1068 1069 1074 1078
GameVisit	3	1 2 3
Group	2	Vegetable Control
Gender	2	Man Woman
SampleName	1	Oat puree

Dimensions	
Covariance Parameters	2
Columns in X	14
Columns in Z	0
Subjects	34
Max Obs per Subject	3

Number of Observations	
Number of Observations Read	102
Number of Observations Used	102
Number of Observations Not Used	0

Iteration History			
Iteration	Evaluations	-2 Res Log Like	Criterion
0	1	879.76909243	
1	1	808.78670754	0.00000000

Convergence criteria met.

Covariance Parameter Estimates		
Cov Parm	Subject	Estimate
CS	ParticipantID	404.48
Residual		102.83

Fit Statistics	
-2 Res Log Likelihood	808.8
AIC (Smaller is Better)	812.8
AICC (Smaller is Better)	812.9
BIC (Smaller is Better)	815.8

Null Model Likelihood Ratio Test		
DF	Chi-Square	Pr > ChiSq

Null Model Likelihood Ratio Test		
DF	Chi-Square	Pr > ChiSq
1	70.98	<.0001

Solution for Fixed Effects								
Effect	Group	Gender	GameVisit	Estimate	Standard Error	DF	t Value	Pr > |t|
Intercept				22.5387	6.3796	38.5	3.53	0.0011
GameVisit			1	-2.8125	3.5852	64	-0.78	0.4357
GameVisit			2	0.5000	3.5852	64	0.14	0.8895
GameVisit			3	0	.	.	.	.
Group	Vegetable			-3.1084	7.8347	40.7	-0.40	0.6936
Group	Control			0	.	.	.	.
GameVisit*Group	Vegetable		1	4.4236	4.9274	64	0.90	0.3727
GameVisit*Group	Control		1	0	.	.	.	.
GameVisit*Group	Vegetable		2	-0.3333	4.9274	64	-0.07	0.9463
GameVisit*Group	Control		2	0	.	.	.	.
GameVisit*Group	Vegetable		3	0	.	.	.	.
GameVisit*Group	Control		3	0	.	.	.	.
Gender		Man		9.5634	7.9967	31	1.20	0.2408
Gender		Woman		0	.	.	.	.

Type 3 Tests of Fixed Effects				
Effect	Num DF	Den DF	F Value	Pr > F
GameVisit	2	64	0.07	0.9289
Group	1	31	0.06	0.8127
GameVisit*Group	2	64	0.58	0.5623
Gender	1	31	1.43	0.2408

Least Squares Means Estimate						
Effect	Label	Estimate	Standard Error	DF	t Value	Pr > |t|
GameVisit*Group	Control, GV3-GV1	2.8125	3.5852	64	0.78	0.4357

Least Squares Means Estimate						
Effect	Label	Estimate	Standard Error	DF	t Value	Pr > |t|
GameVisit*Group	Control, GV2-GV1	3.3125	3.5852	64	0.92	0.3590

Least Squares Means Estimate						
Effect	Label	Estimate	Standard Error	DF	t Value	Pr > |t|
GameVisit*Group	Veg, GV3-GV1	-1.6111	3.3801	64	-0.48	0.6352

Least Squares Means Estimate						
Effect	Label	Estimate	Standard Error	DF	t Value	Pr > |t|
GameVisit*Group	Veg, GV2-GV1	-1.4444	3.3801	64	-0.43	0.6706

Least Squares Means Estimate						
Effect	Label	Estimate	Standard Error	DF	t Value	Pr > |t|
GameVisit*Group	Veg - Control, GV1	1.3152	7.8347	40.68	0.17	0.8675

Least Squares Means Estimate						
Effect	Label	Estimate	Standard Error	DF	t Value	Pr > |t|
GameVisit*Group	Veg - Control, GV2	-3.4417	7.8347	40.68	-0.44	0.6628

Least Squares Means Estimate						
Effect	Label	Estimate	Standard Error	DF	t Value	Pr > |t|
GameVisit*Group	Veg - Control, GV3	-3.1084	7.8347	40.68	-0.40	0.6936

Least Squares Means							
Effect	Group	GameVisit	Estimate	Standard Error	DF	t Value	Pr > |t|
Group	Vegetable		24.8047	5.4138	31	4.58	<.0001
Group	Control		26.5496	5.3312	31	4.98	<.0001
GameVisit		1	25.1656	4.1912	39.3	6.00	<.0001
GameVisit		2	26.0996	4.1912	39.3	6.23	<.0001
GameVisit		3	25.7662	4.1912	39.3	6.15	<.0001
GameVisit*Group	Vegetable	1	25.8232	5.7548	39.3	4.49	<.0001
GameVisit*Group	Control	1	24.5079	5.7189	40.6	4.29	0.0001
GameVisit*Group	Vegetable	2	24.3787	5.7548	39.3	4.24	0.0001
GameVisit*Group	Control	2	27.8204	5.7189	40.6	4.86	<.0001
GameVisit*Group	Vegetable	3	24.2121	5.7548	39.3	4.21	0.0001
GameVisit*Group	Control	3	27.3204	5.7189	40.6	4.78	<.0001

Differences of Least Squares Means									
Effect	Group	GameVisit	_Group	_GameVisit	Estimate	Standard Error	DF	t Value	Pr > |t|
Group	Vegetable		Control		-1.7449	7.3000	31	-0.24	0.8127
GameVisit		1		2	-0.9340	2.4637	64	-0.38	0.7059
GameVisit		1		3	-0.6007	2.4637	64	-0.24	0.8082
GameVisit		2		3	0.3333	2.4637	64	0.14	0.8928
GameVisit*Group	Vegetable	1	Control	1	1.3152	7.8347	40.7	0.17	0.8675
GameVisit*Group	Vegetable	1	Vegetable	2	1.4444	3.3801	64	0.43	0.6706
GameVisit*Group	Vegetable	1	Control	2	-1.9973	7.8347	40.7	-0.25	0.8001
GameVisit*Group	Vegetable	1	Vegetable	3	1.6111	3.3801	64	0.48	0.6352
GameVisit*Group	Vegetable	1	Control	3	-1.4973	7.8347	40.7	-0.19	0.8494
GameVisit*Group	Control	1	Vegetable	2	0.1292	7.8347	40.7	0.02	0.9869
GameVisit*Group	Control	1	Control	2	-3.3125	3.5852	64	-0.92	0.3590
GameVisit*Group	Control	1	Vegetable	3	0.2959	7.8347	40.7	0.04	0.9701
GameVisit*Group	Control	1	Control	3	-2.8125	3.5852	64	-0.78	0.4357
GameVisit*Group	Vegetable	2	Control	2	-3.4417	7.8347	40.7	-0.44	0.6628
GameVisit*Group	Vegetable	2	Vegetable	3	0.1667	3.3801	64	0.05	0.9608
GameVisit*Group	Vegetable	2	Control	3	-2.9417	7.8347	40.7	-0.38	0.7093

Differences of Least Squares Means									
Effect	Group	GameVisit	_Group	_GameVisit	Estimate	Standard Error	DF	t Value	Pr > |t|
GameVisit*Group	Control	2	Vegetable	3	3.6084	7.8347	40.7	0.46	0.6476
GameVisit*Group	Control	2	Control	3	0.5000	3.5852	64	0.14	0.8895
GameVisit*Group	Vegetable	3	Control	3	-3.1084	7.8347	40.7	-0.40	0.6936

OSF planned analysis: Bitterness

The Mixed Procedure

SampleName=Spinach chopped

Model Information	
Data Set	WORK.GAMEVISITS
Dependent Variable	Bitterness
Covariance Structure	Compound Symmetry
Subject Effect	ParticipantID
Estimation Method	REML
Residual Variance Method	Profile
Fixed Effects SE Method	Kenward-Roger
Degrees of Freedom Method	Kenward-Roger

Class Level Information		
Class	Levels	Values
ParticipantID	34	1001 1002 1005 1006 1009 1010 1014 1016 1019 1020 1024 1025 1027 1029 1030 1034 1035 1041 1045 1046 1048 1049 1050 1051 1056 1061 1063 1064 1065 1066 1068 1069 1074 1078
GameVisit	3	1 2 3
Group	2	Vegetable Control
Gender	2	Man Woman
SampleName	1	Spinach chopped

Dimensions	
Covariance Parameters	2
Columns in X	14
Columns in Z	0
Subjects	34
Max Obs per Subject	3

Number of Observations	
Number of Observations Read	102
Number of Observations Used	102
Number of Observations Not Used	0

Iteration History			
Iteration	Evaluations	-2 Res Log Like	Criterion
0	1	861.75091233	

Iteration History			
Iteration	Evaluations	-2 Res Log Like	Criterion
1	1	828.55709671	0.00000000

Convergence criteria met.

Covariance Parameter Estimates		
Cov Parm	Subject	Estimate
CS	ParticipantID	251.39
Residual		166.52

Fit Statistics	
-2 Res Log Likelihood	828.6
AIC (Smaller is Better)	832.6
AICC (Smaller is Better)	832.7
BIC (Smaller is Better)	835.6

Null Model Likelihood Ratio Test		
DF	Chi-Square	Pr > ChiSq
1	33.19	<.0001

Solution for Fixed Effects								
Effect	Group	Gender	GameVisit	Estimate	Standard Error	DF	t Value	Pr > |t|
Intercept				27.1030	5.6929	48.4	4.76	<.0001
GameVisit			1	5.5000	4.5623	64	1.21	0.2324
GameVisit			2	11.8125	4.5623	64	2.59	0.0119
GameVisit			3	0	.	.	.	.
Group	Vegetable			-6.4308	7.0979	53.4	-0.91	0.3690
Group	Control			0	.	.	.	.
GameVisit*Group	Vegetable		1	5.5000	6.2703	64	0.88	0.3837
GameVisit*Group	Control		1	0	.	.	.	.
GameVisit*Group	Vegetable		2	2.2986	6.2703	64	0.37	0.7151
GameVisit*Group	Control		2	0	.	.	.	.
GameVisit*Group	Vegetable		3	0	.	.	.	.
GameVisit*Group	Control		3	0	.	.	.	.
Gender		Man		3.2254	6.6880	31	0.48	0.6330
Gender		Woman		0	.	.	.	.

Type 3 Tests of Fixed Effects				
Effect	Num DF	Den DF	F Value	Pr > F
GameVisit	2	64	8.76	0.0004
Group	1	31	0.39	0.5349
GameVisit*Group	2	64	0.39	0.6799
Gender	1	31	0.23	0.6330

Least Squares Means Estimate						
Effect	Label	Estimate	Standard Error	DF	t Value	Pr > |t|
GameVisit*Group	Control, GV3-GV1	-5.5000	4.5623	64	-1.21	0.2324

Least Squares Means Estimate						
Effect	Label	Estimate	Standard Error	DF	t Value	Pr > |t|
GameVisit*Group	Control, GV2-GV1	6.3125	4.5623	64	1.38	0.1713

Least Squares Means Estimate						
Effect	Label	Estimate	Standard Error	DF	t Value	Pr > |t|
GameVisit*Group	Veg, GV3-GV1	-11.0000	4.3014	64	-2.56	0.0129

Least Squares Means Estimate						
Effect	Label	Estimate	Standard Error	DF	t Value	Pr > |t|
GameVisit*Group	Veg, GV2-GV1	3.1111	4.3014	64	0.72	0.4721

Least Squares Means Estimate						
Effect	Label	Estimate	Standard Error	DF	t Value	Pr > |t|
GameVisit*Group	Veg - Control, GV1	-0.9308	7.0979	53.43	-0.13	0.8962

Least Squares Means Estimate						
Effect	Label	Estimate	Standard Error	DF	t Value	Pr > |t|
GameVisit*Group	Veg - Control, GV2	-4.1322	7.0979	53.43	-0.58	0.5629

Least Squares Means Estimate						
Effect	Label	Estimate	Standard Error	DF	t Value	Pr > |t|
GameVisit*Group	Veg - Control, GV3	-6.4308	7.0979	53.43	-0.91	0.3690

Least Squares Means							
Effect	Group	GameVisit	Estimate	Standard Error	DF	t Value	Pr > |t|
Group	Vegetable		30.6552	4.5278	31	6.77	<.0001
Group	Control		34.4865	4.4587	31	7.73	<.0001
GameVisit		1	33.7502	3.7614	50.3	8.97	<.0001
GameVisit		2	38.4621	3.7614	50.3	10.23	<.0001
GameVisit		3	25.5002	3.7614	50.3	6.78	<.0001
GameVisit*Group	Vegetable	1	33.2848	5.1642	50.3	6.45	<.0001
GameVisit*Group	Control	1	34.2157	5.1786	53.3	6.61	<.0001
GameVisit*Group	Vegetable	2	36.3959	5.1642	50.3	7.05	<.0001
GameVisit*Group	Control	2	40.5282	5.1786	53.3	7.83	<.0001
GameVisit*Group	Vegetable	3	22.2848	5.1642	50.3	4.32	<.0001
GameVisit*Group	Control	3	28.7157	5.1786	53.3	5.55	<.0001

Differences of Least Squares Means									
Effect	Group	GameVisit	_Group	_GameVisit	Estimate	Standard Error	DF	t Value	Pr > |t|
Group	Vegetable		Control		-3.8313	6.1053	31	-0.63	0.5349

Differences of Least Squares Means									
**Effect**	**Group**	**GameVisit**	**_Group**	**_GameVisit**	**Estimate**	**Standard Error**	**DF**	**t Value**	**Pr > |t|**
**GameVisit**		1		2	-4.7118	3.1352	64	-1.50	0.1378
**GameVisit**		1		3	8.2500	3.1352	64	2.63	0.0106
**GameVisit**		2		3	12.9618	3.1352	64	4.13	0.0001
**GameVisit*Group**	Vegetable	1	Control	1	-0.9308	7.0979	53.4	-0.13	0.8962
**GameVisit*Group**	Vegetable	1	Vegetable	2	-3.1111	4.3014	64	-0.72	0.4721
**GameVisit*Group**	Vegetable	1	Control	2	-7.2433	7.0979	53.4	-1.02	0.3121
**GameVisit*Group**	Vegetable	1	Vegetable	3	11.0000	4.3014	64	2.56	0.0129
**GameVisit*Group**	Vegetable	1	Control	3	4.5692	7.0979	53.4	0.64	0.5225
**GameVisit*Group**	Control	1	Vegetable	2	-2.1803	7.0979	53.4	-0.31	0.7599
**GameVisit*Group**	Control	1	Control	2	-6.3125	4.5623	64	-1.38	0.1713
**GameVisit*Group**	Control	1	Vegetable	3	11.9308	7.0979	53.4	1.68	0.0986
**GameVisit*Group**	Control	1	Control	3	5.5000	4.5623	64	1.21	0.2324
**GameVisit*Group**	Vegetable	2	Control	2	-4.1322	7.0979	53.4	-0.58	0.5629
**GameVisit*Group**	Vegetable	2	Vegetable	3	14.1111	4.3014	64	3.28	0.0017
**GameVisit*Group**	Vegetable	2	Control	3	7.6803	7.0979	53.4	1.08	0.2841
**GameVisit*Group**	Control	2	Vegetable	3	18.2433	7.0979	53.4	2.57	0.0130
**GameVisit*Group**	Control	2	Control	3	11.8125	4.5623	64	2.59	0.0119
**GameVisit*Group**	Vegetable	3	Control	3	-6.4308	7.0979	53.4	-0.91	0.3690

OSF planned analysis: Bitterness

The Mixed Procedure

SampleName=Spinach puree

Model Information	
**Data Set**	WORK.GAMEVISITS
**Dependent Variable**	Bitterness
**Covariance Structure**	Compound Symmetry
**Subject Effect**	ParticipantID
**Estimation Method**	REML
**Residual Variance Method**	Profile
**Fixed Effects SE Method**	Kenward-Roger
**Degrees of Freedom Method**	Kenward-Roger

Class Level Information		
**Class**	**Levels**	**Values**
**ParticipantID**	34	1001 1002 1005 1006 1009 1010 1014 1016 1019 1020 1024 1025 1027 1029 1030 1034 1035 1041 1045 1046 1048 1049 1050 1051 1056 1061 1063 1064 1065 1066 1068 1069 1074 1078
**GameVisit**	3	1 2 3
**Group**	2	Vegetable Control
**Gender**	2	Man Woman
**SampleName**	1	Spinach puree

Dimensions	
Covariance Parameters	2
Columns in X	14
Columns in Z	0
Subjects	34
Max Obs per Subject	3

Number of Observations	
Number of Observations Read	102
Number of Observations Used	102
Number of Observations Not Used	0

Iteration History			
Iteration	Evaluations	-2 Res Log Like	Criterion
0	1	908.42910093	
1	1	872.26402415	0.00000000

Convergence criteria met.

Covariance Parameter Estimates		
Cov Parm	Subject	Estimate
CS	ParticipantID	426.10
Residual		257.30

Fit Statistics	
-2 Res Log Likelihood	872.3
AIC (Smaller is Better)	876.3
AICC (Smaller is Better)	876.4
BIC (Smaller is Better)	879.3

Null Model Likelihood Ratio Test		
DF	Chi-Square	Pr > ChiSq
1	36.17	<.0001

Solution for Fixed Effects								
Effect	Group	Gender	GameVisit	Estimate	Standard Error	DF	t Value	Pr > |t|
Intercept				32.9704	7.2941	47.2	4.52	<.0001
GameVisit			1	-1.4375	5.6712	64	-0.25	0.8007
GameVisit			2	7.5000	5.6712	64	1.32	0.1907
GameVisit			3	0	.	.	.	.
Group	Vegetable			-0.2842	9.0786	51.8	-0.03	0.9751
Group	Control			0	.	.	.	.
GameVisit*Group	Vegetable		1	2.2708	7.7943	64	0.29	0.7717
GameVisit*Group	Control		1	0	.	.	.	.
GameVisit*Group	Vegetable		2	-5.9444	7.7943	64	-0.76	0.4485

Solution for Fixed Effects								
Effect	Group	Gender	GameVisit	Estimate	Standard Error	DF	t Value	Pr > |t|
GameVisit*Group	Control		2	0	.	.	.	.
GameVisit*Group	Vegetable		3	0	.	.	.	.
GameVisit*Group	Control		3	0	.	.	.	.
Gender		Man		1.4123	8.6374	31	0.16	0.8712
Gender		Woman		0	.	.	.	.

Type 3 Tests of Fixed Effects				
Effect	Num DF	Den DF	F Value	Pr > F
GameVisit	2	64	0.96	0.3869
Group	1	31	0.04	0.8495
GameVisit*Group	2	64	0.59	0.5560
Gender	1	31	0.03	0.8712

Least Squares Means Estimate						
Effect	Label	Estimate	Standard Error	DF	t Value	Pr > |t|
GameVisit*Group	Control, GV3-GV1	1.4375	5.6712	64	0.25	0.8007

Least Squares Means Estimate						
Effect	Label	Estimate	Standard Error	DF	t Value	Pr > |t|
GameVisit*Group	Control, GV2-GV1	8.9375	5.6712	64	1.58	0.1200

Least Squares Means Estimate						
Effect	Label	Estimate	Standard Error	DF	t Value	Pr > |t|
GameVisit*Group	Veg, GV3-GV1	-0.8333	5.3468	64	-0.16	0.8766

Least Squares Means Estimate						
Effect	Label	Estimate	Standard Error	DF	t Value	Pr > |t|
GameVisit*Group	Veg, GV2-GV1	0.7222	5.3468	64	0.14	0.8930

Least Squares Means Estimate						
Effect	Label	Estimate	Standard Error	DF	t Value	Pr > |t|
GameVisit*Group	Veg - Control, GV1	1.9866	9.0786	51.82	0.22	0.8276

Least Squares Means Estimate						
Effect	Label	Estimate	Standard Error	DF	t Value	Pr > |t|
GameVisit*Group	Veg - Control, GV2	-6.2287	9.0786	51.82	-0.69	0.4957

Least Squares Means Estimate						
Effect	Label	Estimate	Standard Error	DF	t Value	Pr > |t|
GameVisit*Group	Veg - Control, GV3	-0.2842	9.0786	51.82	-0.03	0.9751

Least Squares Means							
Effect	Group	GameVisit	Estimate	Standard Error	DF	t Value	Pr > |t|

Least Squares Means							
Effect	Group	GameVisit	Estimate	Standard Error	DF	t Value	Pr > |t|
Group	Vegetable		34.1886	5.8475	31	5.85	<.0001
Group	Control		35.6974	5.7582	31	6.20	<.0001
GameVisit		1	33.2323	4.8162	48.9	6.90	<.0001
GameVisit		2	38.0622	4.8162	48.9	7.90	<.0001
GameVisit		3	33.5344	4.8162	48.9	6.96	<.0001
GameVisit*Group	Vegetable	1	34.2256	6.6124	48.8	5.18	<.0001
GameVisit*Group	Control	1	32.2390	6.6241	51.7	4.87	<.0001
GameVisit*Group	Vegetable	2	34.9479	6.6124	48.8	5.29	<.0001
GameVisit*Group	Control	2	41.1765	6.6241	51.7	6.22	<.0001
GameVisit*Group	Vegetable	3	33.3923	6.6124	48.8	5.05	<.0001
GameVisit*Group	Control	3	33.6765	6.6241	51.7	5.08	<.0001

Differences of Least Squares Means									
Effect	Group	GameVisit	_Group	_GameVisit	Estimate	Standard Error	DF	t Value	Pr > |t|
Group	Vegetable		Control		-1.5088	7.8848	31	-0.19	0.8495
GameVisit		1		2	-4.8299	3.8972	64	-1.24	0.2197
GameVisit		1		3	-0.3021	3.8972	64	-0.08	0.9385
GameVisit		2		3	4.5278	3.8972	64	1.16	0.2496
GameVisit*Group	Vegetable	1	Control	1	1.9866	9.0786	51.8	0.22	0.8276
GameVisit*Group	Vegetable	1	Vegetable	2	-0.7222	5.3468	64	-0.14	0.8930
GameVisit*Group	Vegetable	1	Control	2	-6.9509	9.0786	51.8	-0.77	0.4474
GameVisit*Group	Vegetable	1	Vegetable	3	0.8333	5.3468	64	0.16	0.8766
GameVisit*Group	Vegetable	1	Control	3	0.5491	9.0786	51.8	0.06	0.9520
GameVisit*Group	Control	1	Vegetable	2	-2.7088	9.0786	51.8	-0.30	0.7666
GameVisit*Group	Control	1	Control	2	-8.9375	5.6712	64	-1.58	0.1200
GameVisit*Group	Control	1	Vegetable	3	-1.1533	9.0786	51.8	-0.13	0.8994
GameVisit*Group	Control	1	Control	3	-1.4375	5.6712	64	-0.25	0.8007
GameVisit*Group	Vegetable	2	Control	2	-6.2287	9.0786	51.8	-0.69	0.4957
GameVisit*Group	Vegetable	2	Vegetable	3	1.5556	5.3468	64	0.29	0.7720
GameVisit*Group	Vegetable	2	Control	3	1.2713	9.0786	51.8	0.14	0.8892
GameVisit*Group	Control	2	Vegetable	3	7.7842	9.0786	51.8	0.86	0.3952
GameVisit*Group	Control	2	Control	3	7.5000	5.6712	64	1.32	0.1907
GameVisit*Group	Vegetable	3	Control	3	-0.2842	9.0786	51.8	-0.03	0.9751

Adding an additional check for whether vegetables differ from each other in liking.

This may be useful in understanding differences in effects.

In [7]:

```
%%SAS sas

proc sort data=gamevisits;
by gamevisit;
run;
```

```
title 'Secondard analysis: Liking between vegetables';
ods output diffs=diff4 tests3=tests4
SolutionF=sol4 lsmeans=means4 FitStatistics=fit4 lsmESTIMATEs =LikeEstim4;
proc mixed data= gamevisits;
class participantID gamevisit group (ref = 'Control') gender samplename;
by gamevisit;
model liking = samplename group samplename*group gender/ ddfm=kr solution; *fit is slightly better wit
repeated/ subject = participantID type = cs;
lsmeans group/pdiff ADJDFE=ROW;
lsmeans samplename/pdiff ADJDFE=ROW;
lsmeans samplename*group/pdiff adjdfe=row;
run;
```

Out[7]:

Secondard analysis: Liking between vegetables

The Mixed Procedure

GameVisit=1

Model Information	
Data Set	WORK.GAMEVISITS
Dependent Variable	Liking
Covariance Structure	Compound Symmetry
Subject Effect	ParticipantID
Estimation Method	REML
Residual Variance Method	Profile
Fixed Effects SE Method	Kenward-Roger
Degrees of Freedom Method	Kenward-Roger

Class Level Information		
Class	Levels	Values
ParticipantID	34	1001 1002 1005 1006 1009 1010 1014 1016 1019 1020 1024 1025 1027 1029 1030 1034 1035 1041 1045 1046 1048 1049 1050 1051 1056 1061 1063 1064 1065 1066 1068 1069 1074 1078
GameVisit	1	1
Group	2	Vegetable Control
Gender	2	Man Woman
SampleName	12	Asparagus chopped Asparagus puree Beef puree Black bean puree Broccoli chopped Broccoli puree Chicken puree Kale chopped Kale puree Oat puree Spinach chopped Spinach puree

Dimensions	
Covariance Parameters	2
Columns in X	41
Columns in Z	0
Subjects	34
Max Obs per Subject	12

Number of Observations	
Number of Observations Read	408
Number of Observations Used	408
Number of Observations Not Used	0

Iteration History			
Iteration	Evaluations	-2 Res Log Like	Criterion

Iteration History			
**Iteration**	**Evaluations**	**-2 Res Log Like**	**Criterion**
0	1	4001.62782322	
1	1	3937.37708930	0.00000000

Convergence criteria met.

Covariance Parameter Estimates		
**Cov Parm**	**Subject**	**Estimate**
CS	ParticipantID	451.20
Residual		1232.95

Fit Statistics	
-2 Res Log Likelihood	3937.4
AIC (Smaller is Better)	3941.4
AICC (Smaller is Better)	3941.4
BIC (Smaller is Better)	3944.4

Null Model Likelihood Ratio Test		
**DF**	**Chi-Square**	**Pr > ChiSq**
1	64.25	<.0001

Solution for Fixed Effects								
**Effect**	**Group**	**Gender**	**SampleName**	**Estimate**	**Standard Error**	**DF**	**t Value**	**Pr > |t|**
Intercept				-9.0509	10.7987	165	-0.84	0.4032
SampleName			Asparagus chopped	24.8125	12.4145	352	2.00	0.0464
SampleName			Asparagus puree	19.0625	12.4145	352	1.54	0.1256
SampleName			Beef puree	-0.2500	12.4145	352	-0.02	0.9839
SampleName			Black bean puree	7.2500	12.4145	352	0.58	0.5596
SampleName			Broccoli chopped	22.8125	12.4145	352	1.84	0.0670
SampleName			Broccoli puree	16.8750	12.4145	352	1.36	0.1749
SampleName			Chicken puree	-0.4375	12.4145	352	-0.04	0.9719
SampleName			Kale chopped	-6.9375	12.4145	352	-0.56	0.5766
SampleName			Kale puree	1.5000	12.4145	352	0.12	0.9039
SampleName			Oat puree	-13.0000	12.4145	352	-1.05	0.2957
SampleName			Spinach chopped	0.5625	12.4145	352	0.05	0.9639
SampleName			Spinach puree	0	.	.	.	.
Group	Vegetable			0.002202	14.1671	205	0.00	0.9999
Group	Control			0	.	.	.	.
Group*SampleName	Vegetable		Asparagus chopped	-21.3125	17.0621	352	-1.25	0.2125
Group*SampleName	Vegetable		Asparagus puree	-15.8403	17.0621	352	-0.93	0.3538
Group*SampleName	Vegetable		Beef puree	-15.3611	17.0621	352	-0.90	0.3686
Group*SampleName	Vegetable		Black bean puree	-10.6389	17.0621	352	-0.62	0.5333
Group*SampleName	Vegetable		Broccoli chopped	-1.5903	17.0621	352	-0.09	0.9258
Group*SampleName	Vegetable		Broccoli puree	8.2917	17.0621	352	0.49	0.6273

Solution for Fixed Effects								
Effect	Group	Gender	SampleName	Estimate	Standard Error	DF	t Value	Pr > |t|
Group*SampleName	Vegetable		Chicken puree	-18.3958	17.0621	352	-1.08	0.2817
Group*SampleName	Vegetable		Kale chopped	-16.7847	17.0621	352	-0.98	0.3259
Group*SampleName	Vegetable		Kale puree	-11.3333	17.0621	352	-0.66	0.5070
Group*SampleName	Vegetable		Oat puree	16.3333	17.0621	352	0.96	0.3391
Group*SampleName	Vegetable		Spinach chopped	1.4375	17.0621	352	0.08	0.9329
Group*SampleName	Vegetable		Spinach puree	0	.	.	.	.
Group*SampleName	Control		Asparagus chopped	0	.	.	.	.
Group*SampleName	Control		Asparagus puree	0	.	.	.	.
Group*SampleName	Control		Beef puree	0	.	.	.	.
Group*SampleName	Control		Black bean puree	0	.	.	.	.
Group*SampleName	Control		Broccoli chopped	0	.	.	.	.
Group*SampleName	Control		Broccoli puree	0	.	.	.	.
Group*SampleName	Control		Chicken puree	0	.	.	.	.
Group*SampleName	Control		Kale chopped	0	.	.	.	.
Group*SampleName	Control		Kale puree	0	.	.	.	.
Group*SampleName	Control		Oat puree	0	.	.	.	.
Group*SampleName	Control		Spinach chopped	0	.	.	.	.
Group*SampleName	Control		Spinach puree	0	.	.	.	.
Gender		Man		3.9690	8.9853	31	0.44	0.6618
Gender		Woman		0	.	.	.	.

Type 3 Tests of Fixed Effects				
Effect	Num DF	Den DF	F Value	Pr > F
SampleName	11	352	4.03	<.0001
Group	1	31	0.75	0.3935
Group*SampleName	11	352	0.96	0.4822
Gender	1	31	0.20	0.6618

Least Squares Means							
Effect	Group	SampleName	Estimate	Standard Error	DF	t Value	Pr > |t|
Group	Vegetable		-8.1429	6.0831	31	-1.34	0.1904
Group	Control		-1.0455	5.9902	31	-0.17	0.8626
SampleName		Asparagus chopped	7.0910	7.2788	180	0.97	0.3313
SampleName		Asparagus puree	4.0771	7.2788	180	0.56	0.5761
SampleName		Beef puree	-14.9958	7.2788	180	-2.06	0.0408
SampleName		Black bean puree	-5.1347	7.2788	180	-0.71	0.4814
SampleName		Broccoli chopped	14.9521	7.2788	180	2.05	0.0414
SampleName		Broccoli puree	13.9556	7.2788	180	1.92	0.0568
SampleName		Chicken puree	-16.7007	7.2788	180	-2.29	0.0229
SampleName		Kale chopped	-22.3951	7.2788	180	-3.08	0.0024
SampleName		Kale puree	-11.2319	7.2788	180	-1.54	0.1246
SampleName		Oat puree	-11.8986	7.2788	180	-1.63	0.1039

Least Squares Means							
Effect	Group	SampleName	Estimate	Standard Error	DF	t Value	Pr > |t|
SampleName		Spinach chopped	-5.7840	7.2788	180	-0.79	0.4279
SampleName		Spinach puree	-7.0653	7.2788	180	-0.97	0.3330
Group*SampleName	Vegetable	Asparagus chopped	-3.5642	9.9897	180	-0.36	0.7217
Group*SampleName	Vegetable	Asparagus puree	-3.8420	9.9897	180	-0.38	0.7010
Group*SampleName	Vegetable	Beef puree	-22.6753	9.9897	180	-2.27	0.0244
Group*SampleName	Vegetable	Black bean puree	-10.4531	9.9897	180	-1.05	0.2968
Group*SampleName	Vegetable	Broccoli chopped	14.1580	9.9897	180	1.42	0.1581
Group*SampleName	Vegetable	Broccoli puree	18.1025	9.9897	180	1.81	0.0716
Group*SampleName	Vegetable	Chicken puree	-25.8975	9.9897	180	-2.59	0.0103
Group*SampleName	Vegetable	Kale chopped	-30.7864	9.9897	180	-3.08	0.0024
Group*SampleName	Vegetable	Kale puree	-16.8975	9.9897	180	-1.69	0.0925
Group*SampleName	Vegetable	Oat puree	-3.7308	9.9897	180	-0.37	0.7092
Group*SampleName	Vegetable	Spinach chopped	-5.0642	9.9897	180	-0.51	0.6128
Group*SampleName	Vegetable	Spinach puree	-7.0642	9.9897	180	-0.71	0.4804
Group*SampleName	Control	Asparagus chopped	17.7461	10.3209	204	1.72	0.0871
Group*SampleName	Control	Asparagus puree	11.9961	10.3209	204	1.16	0.2465
Group*SampleName	Control	Beef puree	-7.3164	10.3209	204	-0.71	0.4792
Group*SampleName	Control	Black bean puree	0.1836	10.3209	204	0.02	0.9858
Group*SampleName	Control	Broccoli chopped	15.7461	10.3209	204	1.53	0.1286
Group*SampleName	Control	Broccoli puree	9.8086	10.3209	204	0.95	0.3431
Group*SampleName	Control	Chicken puree	-7.5039	10.3209	204	-0.73	0.4680
Group*SampleName	Control	Kale chopped	-14.0039	10.3209	204	-1.36	0.1763
Group*SampleName	Control	Kale puree	-5.5664	10.3209	204	-0.54	0.5902
Group*SampleName	Control	Oat puree	-20.0664	10.3209	204	-1.94	0.0532
Group*SampleName	Control	Spinach chopped	-6.5039	10.3209	204	-0.63	0.5293
Group*SampleName	Control	Spinach puree	-7.0664	10.3209	204	-0.68	0.4943

Differences of Least Squares Means									
Effect	Group	SampleName	_Group	_SampleName	Estimate	Standard Error	DF	t Value	Pr > |t|
Group	Vegetable		Control		-7.0973	8.2025	31	-0.87	0.3935
SampleName		Asparagus chopped		Asparagus puree	3.0139	8.5310	352	0.35	0.7241
SampleName		Asparagus chopped		Beef puree	22.0868	8.5310	352	2.59	0.0100
SampleName		Asparagus chopped		Black bean puree	12.2257	8.5310	352	1.43	0.1527
SampleName		Asparagus chopped		Broccoli chopped	-7.8611	8.5310	352	-0.92	0.3574
SampleName		Asparagus chopped		Broccoli puree	-6.8646	8.5310	352	-0.80	0.4216
SampleName		Asparagus chopped		Chicken puree	23.7917	8.5310	352	2.79	0.0056
SampleName		Asparagus chopped		Kale chopped	29.4861	8.5310	352	3.46	0.0006
SampleName		Asparagus chopped		Kale puree	18.3229	8.5310	352	2.15	0.0324
SampleName		Asparagus chopped		Oat puree	18.9896	8.5310	352	2.23	0.0267
SampleName		Asparagus chopped		Spinach chopped	12.8750	8.5310	352	1.51	0.1321
SampleName		Asparagus chopped		Spinach puree	14.1562	8.5310	352	1.66	0.0979
SampleName		Asparagus puree		Beef puree	19.0729	8.5310	352	2.24	0.0260

Differences of Least Squares Means									
Effect	Group	SampleName	_Group	_SampleName	Estimate	Standard Error	DF	t Value	Pr > |t|
SampleName		Asparagus puree		Black bean puree	9.2118	8.5310	352	1.08	0.2810
SampleName		Asparagus puree		Broccoli chopped	-10.8750	8.5310	352	-1.27	0.2032
SampleName		Asparagus puree		Broccoli puree	-9.8785	8.5310	352	-1.16	0.2477
SampleName		Asparagus puree		Chicken puree	20.7778	8.5310	352	2.44	0.0154
SampleName		Asparagus puree		Kale chopped	26.4722	8.5310	352	3.10	0.0021
SampleName		Asparagus puree		Kale puree	15.3090	8.5310	352	1.79	0.0736
SampleName		Asparagus puree		Oat puree	15.9757	8.5310	352	1.87	0.0619
SampleName		Asparagus puree		Spinach chopped	9.8611	8.5310	352	1.16	0.2485
SampleName		Asparagus puree		Spinach puree	11.1424	8.5310	352	1.31	0.1924
SampleName		Beef puree		Black bean puree	-9.8611	8.5310	352	-1.16	0.2485
SampleName		Beef puree		Broccoli chopped	-29.9479	8.5310	352	-3.51	0.0005
SampleName		Beef puree		Broccoli puree	-28.9514	8.5310	352	-3.39	0.0008
SampleName		Beef puree		Chicken puree	1.7049	8.5310	352	0.20	0.8417
SampleName		Beef puree		Kale chopped	7.3993	8.5310	352	0.87	0.3863
SampleName		Beef puree		Kale puree	-3.7639	8.5310	352	-0.44	0.6593
SampleName		Beef puree		Oat puree	-3.0972	8.5310	352	-0.36	0.7168
SampleName		Beef puree		Spinach chopped	-9.2118	8.5310	352	-1.08	0.2810
SampleName		Beef puree		Spinach puree	-7.9306	8.5310	352	-0.93	0.3532
SampleName		Black bean puree		Broccoli chopped	-20.0868	8.5310	352	-2.35	0.0191
SampleName		Black bean puree		Broccoli puree	-19.0903	8.5310	352	-2.24	0.0259
SampleName		Black bean puree		Chicken puree	11.5660	8.5310	352	1.36	0.1760
SampleName		Black bean puree		Kale chopped	17.2604	8.5310	352	2.02	0.0438
SampleName		Black bean puree		Kale puree	6.0972	8.5310	352	0.71	0.4753
SampleName		Black bean puree		Oat puree	6.7639	8.5310	352	0.79	0.4284
SampleName		Black bean puree		Spinach chopped	0.6493	8.5310	352	0.08	0.9394
SampleName		Black bean puree		Spinach puree	1.9306	8.5310	352	0.23	0.8211
SampleName		Broccoli chopped		Broccoli puree	0.9965	8.5310	352	0.12	0.9071
SampleName		Broccoli chopped		Chicken puree	31.6528	8.5310	352	3.71	0.0002
SampleName		Broccoli chopped		Kale chopped	37.3472	8.5310	352	4.38	<.0001
SampleName		Broccoli chopped		Kale puree	26.1840	8.5310	352	3.07	0.0023
SampleName		Broccoli chopped		Oat puree	26.8507	8.5310	352	3.15	0.0018
SampleName		Broccoli chopped		Spinach chopped	20.7361	8.5310	352	2.43	0.0156
SampleName		Broccoli chopped		Spinach puree	22.0174	8.5310	352	2.58	0.0103
SampleName		Broccoli puree		Chicken puree	30.6563	8.5310	352	3.59	0.0004
SampleName		Broccoli puree		Kale chopped	36.3507	8.5310	352	4.26	<.0001
SampleName		Broccoli puree		Kale puree	25.1875	8.5310	352	2.95	0.0034
SampleName		Broccoli puree		Oat puree	25.8542	8.5310	352	3.03	0.0026
SampleName		Broccoli puree		Spinach chopped	19.7396	8.5310	352	2.31	0.0213
SampleName		Broccoli puree		Spinach puree	21.0208	8.5310	352	2.46	0.0142
SampleName		Chicken puree		Kale chopped	5.6944	8.5310	352	0.67	0.5049
SampleName		Chicken puree		Kale puree	-5.4688	8.5310	352	-0.64	0.5219
SampleName		Chicken puree		Oat puree	-4.8021	8.5310	352	-0.56	0.5739

Differences of Least Squares Means									
Effect	Group	SampleName	_Group	_SampleName	Estimate	Standard Error	DF	t Value	Pr > |t|
SampleName		Chicken puree		Spinach chopped	-10.9167	8.5310	352	-1.28	0.2015
SampleName		Chicken puree		Spinach puree	-9.6354	8.5310	352	-1.13	0.2595
SampleName		Kale chopped		Kale puree	-11.1632	8.5310	352	-1.31	0.1915
SampleName		Kale chopped		Oat puree	-10.4965	8.5310	352	-1.23	0.2194
SampleName		Kale chopped		Spinach chopped	-16.6111	8.5310	352	-1.95	0.0523
SampleName		Kale chopped		Spinach puree	-15.3299	8.5310	352	-1.80	0.0732
SampleName		Kale puree		Oat puree	0.6667	8.5310	352	0.08	0.9378
SampleName		Kale puree		Spinach chopped	-5.4479	8.5310	352	-0.64	0.5235
SampleName		Kale puree		Spinach puree	-4.1667	8.5310	352	-0.49	0.6256
SampleName		Oat puree		Spinach chopped	-6.1146	8.5310	352	-0.72	0.4740
SampleName		Oat puree		Spinach puree	-4.8333	8.5310	352	-0.57	0.5714
SampleName		Spinach chopped		Spinach puree	1.2812	8.5310	352	0.15	0.8807
Group*SampleName	Vegetable	Asparagus chopped	Vegetable	Asparagus puree	0.2778	11.7045	352	0.02	0.9811
Group*SampleName	Vegetable	Asparagus chopped	Vegetable	Beef puree	19.1111	11.7045	352	1.63	0.1034
Group*SampleName	Vegetable	Asparagus chopped	Vegetable	Black bean puree	6.8889	11.7045	352	0.59	0.5565
Group*SampleName	Vegetable	Asparagus chopped	Vegetable	Broccoli chopped	-17.7222	11.7045	352	-1.51	0.1309
Group*SampleName	Vegetable	Asparagus chopped	Vegetable	Broccoli puree	-21.6667	11.7045	352	-1.85	0.0650
Group*SampleName	Vegetable	Asparagus chopped	Vegetable	Chicken puree	22.3333	11.7045	352	1.91	0.0572
Group*SampleName	Vegetable	Asparagus chopped	Vegetable	Kale chopped	27.2222	11.7045	352	2.33	0.0206
Group*SampleName	Vegetable	Asparagus chopped	Vegetable	Kale puree	13.3333	11.7045	352	1.14	0.2554
Group*SampleName	Vegetable	Asparagus chopped	Vegetable	Oat puree	0.1667	11.7045	352	0.01	0.9886
Group*SampleName	Vegetable	Asparagus chopped	Vegetable	Spinach chopped	1.5000	11.7045	352	0.13	0.8981
Group*SampleName	Vegetable	Asparagus chopped	Vegetable	Spinach puree	3.5000	11.7045	352	0.30	0.7651
Group*SampleName	Vegetable	Asparagus chopped	Control	Asparagus chopped	-21.3103	14.1671	205	-1.50	0.1341
Group*SampleName	Vegetable	Asparagus chopped	Control	Asparagus puree	-15.5603	14.1671	205	-1.10	0.2733
Group*SampleName	Vegetable	Asparagus chopped	Control	Beef puree	3.7522	14.1671	205	0.26	0.7914
Group*SampleName	Vegetable	Asparagus chopped	Control	Black bean puree	-3.7478	14.1671	205	-0.26	0.7916
Group*SampleName	Vegetable	Asparagus chopped	Control	Broccoli chopped	-19.3103	14.1671	205	-1.36	0.1744
Group*SampleName	Vegetable	Asparagus chopped	Control	Broccoli puree	-13.3728	14.1671	205	-0.94	0.3463
Group*SampleName	Vegetable	Asparagus chopped	Control	Chicken puree	3.9397	14.1671	205	0.28	0.7812
Group*SampleName	Vegetable	Asparagus chopped	Control	Kale chopped	10.4397	14.1671	205	0.74	0.4620
Group*SampleName	Vegetable	Asparagus chopped	Control	Kale puree	2.0022	14.1671	205	0.14	0.8878
Group*SampleName	Vegetable	Asparagus chopped	Control	Oat puree	16.5022	14.1671	205	1.16	0.2454
Group*SampleName	Vegetable	Asparagus chopped	Control	Spinach chopped	2.9397	14.1671	205	0.21	0.8358
Group*SampleName	Vegetable	Asparagus chopped	Control	Spinach puree	3.5022	14.1671	205	0.25	0.8050
Group*SampleName	Vegetable	Asparagus puree	Vegetable	Beef puree	18.8333	11.7045	352	1.61	0.1085
Group*SampleName	Vegetable	Asparagus puree	Vegetable	Black bean puree	6.6111	11.7045	352	0.56	0.5725
Group*SampleName	Vegetable	Asparagus puree	Vegetable	Broccoli chopped	-18.0000	11.7045	352	-1.54	0.1250
Group*SampleName	Vegetable	Asparagus puree	Vegetable	Broccoli puree	-21.9444	11.7045	352	-1.87	0.0616
Group*SampleName	Vegetable	Asparagus puree	Vegetable	Chicken puree	22.0556	11.7045	352	1.88	0.0603
Group*SampleName	Vegetable	Asparagus puree	Vegetable	Kale chopped	26.9444	11.7045	352	2.30	0.0219
Group*SampleName	Vegetable	Asparagus puree	Vegetable	Kale puree	13.0556	11.7045	352	1.12	0.2654

Differences of Least Squares Means									
Effect	Group	SampleName	_Group	_SampleName	Estimate	Standard Error	DF	t Value	Pr > |t|
Group*SampleName	Vegetable	Asparagus puree	Vegetable	Oat puree	-0.1111	11.7045	352	-0.01	0.9924
Group*SampleName	Vegetable	Asparagus puree	Vegetable	Spinach chopped	1.2222	11.7045	352	0.10	0.9169
Group*SampleName	Vegetable	Asparagus puree	Vegetable	Spinach puree	3.2222	11.7045	352	0.28	0.7832
Group*SampleName	Vegetable	Asparagus puree	Control	Asparagus chopped	-21.5881	14.1671	205	-1.52	0.1291
Group*SampleName	Vegetable	Asparagus puree	Control	Asparagus puree	-15.8381	14.1671	205	-1.12	0.2649
Group*SampleName	Vegetable	Asparagus puree	Control	Beef puree	3.4744	14.1671	205	0.25	0.8065
Group*SampleName	Vegetable	Asparagus puree	Control	Black bean puree	-4.0256	14.1671	205	-0.28	0.7766
Group*SampleName	Vegetable	Asparagus puree	Control	Broccoli chopped	-19.5881	14.1671	205	-1.38	0.1683
Group*SampleName	Vegetable	Asparagus puree	Control	Broccoli puree	-13.6506	14.1671	205	-0.96	0.3364
Group*SampleName	Vegetable	Asparagus puree	Control	Chicken puree	3.6619	14.1671	205	0.26	0.7963
Group*SampleName	Vegetable	Asparagus puree	Control	Kale chopped	10.1619	14.1671	205	0.72	0.4740
Group*SampleName	Vegetable	Asparagus puree	Control	Kale puree	1.7244	14.1671	205	0.12	0.9032
Group*SampleName	Vegetable	Asparagus puree	Control	Oat puree	16.2244	14.1671	205	1.15	0.2535
Group*SampleName	Vegetable	Asparagus puree	Control	Spinach chopped	2.6619	14.1671	205	0.19	0.8511
Group*SampleName	Vegetable	Asparagus puree	Control	Spinach puree	3.2244	14.1671	205	0.23	0.8202
Group*SampleName	Vegetable	Beef puree	Vegetable	Black bean puree	-12.2222	11.7045	352	-1.04	0.2971
Group*SampleName	Vegetable	Beef puree	Vegetable	Broccoli chopped	-36.8333	11.7045	352	-3.15	0.0018
Group*SampleName	Vegetable	Beef puree	Vegetable	Broccoli puree	-40.7778	11.7045	352	-3.48	0.0006
Group*SampleName	Vegetable	Beef puree	Vegetable	Chicken puree	3.2222	11.7045	352	0.28	0.7832
Group*SampleName	Vegetable	Beef puree	Vegetable	Kale chopped	8.1111	11.7045	352	0.69	0.4888
Group*SampleName	Vegetable	Beef puree	Vegetable	Kale puree	-5.7778	11.7045	352	-0.49	0.6219
Group*SampleName	Vegetable	Beef puree	Vegetable	Oat puree	-18.9444	11.7045	352	-1.62	0.1064
Group*SampleName	Vegetable	Beef puree	Vegetable	Spinach chopped	-17.6111	11.7045	352	-1.50	0.1333
Group*SampleName	Vegetable	Beef puree	Vegetable	Spinach puree	-15.6111	11.7045	352	-1.33	0.1831
Group*SampleName	Vegetable	Beef puree	Control	Asparagus chopped	-40.4214	14.1671	205	-2.85	0.0048
Group*SampleName	Vegetable	Beef puree	Control	Asparagus puree	-34.6714	14.1671	205	-2.45	0.0152
Group*SampleName	Vegetable	Beef puree	Control	Beef puree	-15.3589	14.1671	205	-1.08	0.2796
Group*SampleName	Vegetable	Beef puree	Control	Black bean puree	-22.8589	14.1671	205	-1.61	0.1082
Group*SampleName	Vegetable	Beef puree	Control	Broccoli chopped	-38.4214	14.1671	205	-2.71	0.0073
Group*SampleName	Vegetable	Beef puree	Control	Broccoli puree	-32.4839	14.1671	205	-2.29	0.0229
Group*SampleName	Vegetable	Beef puree	Control	Chicken puree	-15.1714	14.1671	205	-1.07	0.2855
Group*SampleName	Vegetable	Beef puree	Control	Kale chopped	-8.6714	14.1671	205	-0.61	0.5412
Group*SampleName	Vegetable	Beef puree	Control	Kale puree	-17.1089	14.1671	205	-1.21	0.2286
Group*SampleName	Vegetable	Beef puree	Control	Oat puree	-2.6089	14.1671	205	-0.18	0.8541
Group*SampleName	Vegetable	Beef puree	Control	Spinach chopped	-16.1714	14.1671	205	-1.14	0.2550
Group*SampleName	Vegetable	Beef puree	Control	Spinach puree	-15.6089	14.1671	205	-1.10	0.2719
Group*SampleName	Vegetable	Black bean puree	Vegetable	Broccoli chopped	-24.6111	11.7045	352	-2.10	0.0362
Group*SampleName	Vegetable	Black bean puree	Vegetable	Broccoli puree	-28.5556	11.7045	352	-2.44	0.0152
Group*SampleName	Vegetable	Black bean puree	Vegetable	Chicken puree	15.4444	11.7045	352	1.32	0.1878
Group*SampleName	Vegetable	Black bean puree	Vegetable	Kale chopped	20.3333	11.7045	352	1.74	0.0832
Group*SampleName	Vegetable	Black bean puree	Vegetable	Kale puree	6.4444	11.7045	352	0.55	0.5823
Group*SampleName	Vegetable	Black bean puree	Vegetable	Oat puree	-6.7222	11.7045	352	-0.57	0.5661

Differences of Least Squares Means									
Effect	Group	SampleName	_Group	_SampleName	Estimate	Standard Error	DF	t Value	Pr > |t|
Group*SampleName	Vegetable	Black bean puree	Vegetable	Spinach chopped	-5.3889	11.7045	352	-0.46	0.6455
Group*SampleName	Vegetable	Black bean puree	Vegetable	Spinach puree	-3.3889	11.7045	352	-0.29	0.7723
Group*SampleName	Vegetable	Black bean puree	Control	Asparagus chopped	-28.1992	14.1671	205	-1.99	0.0479
Group*SampleName	Vegetable	Black bean puree	Control	Asparagus puree	-22.4492	14.1671	205	-1.58	0.1146
Group*SampleName	Vegetable	Black bean puree	Control	Beef puree	-3.1367	14.1671	205	-0.22	0.8250
Group*SampleName	Vegetable	Black bean puree	Control	Black bean puree	-10.6367	14.1671	205	-0.75	0.4536
Group*SampleName	Vegetable	Black bean puree	Control	Broccoli chopped	-26.1992	14.1671	205	-1.85	0.0659
Group*SampleName	Vegetable	Black bean puree	Control	Broccoli puree	-20.2617	14.1671	205	-1.43	0.1542
Group*SampleName	Vegetable	Black bean puree	Control	Chicken puree	-2.9492	14.1671	205	-0.21	0.8353
Group*SampleName	Vegetable	Black bean puree	Control	Kale chopped	3.5508	14.1671	205	0.25	0.8023
Group*SampleName	Vegetable	Black bean puree	Control	Kale puree	-4.8867	14.1671	205	-0.34	0.7305
Group*SampleName	Vegetable	Black bean puree	Control	Oat puree	9.6133	14.1671	205	0.68	0.4982
Group*SampleName	Vegetable	Black bean puree	Control	Spinach chopped	-3.9492	14.1671	205	-0.28	0.7807
Group*SampleName	Vegetable	Black bean puree	Control	Spinach puree	-3.3867	14.1671	205	-0.24	0.8113
Group*SampleName	Vegetable	Broccoli chopped	Vegetable	Broccoli puree	-3.9444	11.7045	352	-0.34	0.7363
Group*SampleName	Vegetable	Broccoli chopped	Vegetable	Chicken puree	40.0556	11.7045	352	3.42	0.0007
Group*SampleName	Vegetable	Broccoli chopped	Vegetable	Kale chopped	44.9444	11.7045	352	3.84	0.0001
Group*SampleName	Vegetable	Broccoli chopped	Vegetable	Kale puree	31.0556	11.7045	352	2.65	0.0083
Group*SampleName	Vegetable	Broccoli chopped	Vegetable	Oat puree	17.8889	11.7045	352	1.53	0.1273
Group*SampleName	Vegetable	Broccoli chopped	Vegetable	Spinach chopped	19.2222	11.7045	352	1.64	0.1014
Group*SampleName	Vegetable	Broccoli chopped	Vegetable	Spinach puree	21.2222	11.7045	352	1.81	0.0707
Group*SampleName	Vegetable	Broccoli chopped	Control	Asparagus chopped	-3.5881	14.1671	205	-0.25	0.8003
Group*SampleName	Vegetable	Broccoli chopped	Control	Asparagus puree	2.1619	14.1671	205	0.15	0.8789
Group*SampleName	Vegetable	Broccoli chopped	Control	Beef puree	21.4744	14.1671	205	1.52	0.1311
Group*SampleName	Vegetable	Broccoli chopped	Control	Black bean puree	13.9744	14.1671	205	0.99	0.3251
Group*SampleName	Vegetable	Broccoli chopped	Control	Broccoli chopped	-1.5881	14.1671	205	-0.11	0.9109
Group*SampleName	Vegetable	Broccoli chopped	Control	Broccoli puree	4.3494	14.1671	205	0.31	0.7591
Group*SampleName	Vegetable	Broccoli chopped	Control	Chicken puree	21.6619	14.1671	205	1.53	0.1278
Group*SampleName	Vegetable	Broccoli chopped	Control	Kale chopped	28.1619	14.1671	205	1.99	0.0482
Group*SampleName	Vegetable	Broccoli chopped	Control	Kale puree	19.7244	14.1671	205	1.39	0.1654
Group*SampleName	Vegetable	Broccoli chopped	Control	Oat puree	34.2244	14.1671	205	2.42	0.0166
Group*SampleName	Vegetable	Broccoli chopped	Control	Spinach chopped	20.6619	14.1671	205	1.46	0.1463
Group*SampleName	Vegetable	Broccoli chopped	Control	Spinach puree	21.2244	14.1671	205	1.50	0.1356
Group*SampleName	Vegetable	Broccoli puree	Vegetable	Chicken puree	44.0000	11.7045	352	3.76	0.0002
Group*SampleName	Vegetable	Broccoli puree	Vegetable	Kale chopped	48.8889	11.7045	352	4.18	<.0001
Group*SampleName	Vegetable	Broccoli puree	Vegetable	Kale puree	35.0000	11.7045	352	2.99	0.0030
Group*SampleName	Vegetable	Broccoli puree	Vegetable	Oat puree	21.8333	11.7045	352	1.87	0.0630
Group*SampleName	Vegetable	Broccoli puree	Vegetable	Spinach chopped	23.1667	11.7045	352	1.98	0.0486
Group*SampleName	Vegetable	Broccoli puree	Vegetable	Spinach puree	25.1667	11.7045	352	2.15	0.0322
Group*SampleName	Vegetable	Broccoli puree	Control	Asparagus chopped	0.3564	14.1671	205	0.03	0.9800
Group*SampleName	Vegetable	Broccoli puree	Control	Asparagus puree	6.1064	14.1671	205	0.43	0.6669
Group*SampleName	Vegetable	Broccoli puree	Control	Beef puree	25.4189	14.1671	205	1.79	0.0743

Differences of Least Squares Means									
Effect	Group	SampleName	_Group	_SampleName	Estimate	Standard Error	DF	t Value	Pr > |t|
Group*SampleName	Vegetable	Broccoli puree	Control	Black bean puree	17.9189	14.1671	205	1.26	0.2074
Group*SampleName	Vegetable	Broccoli puree	Control	Broccoli chopped	2.3564	14.1671	205	0.17	0.8681
Group*SampleName	Vegetable	Broccoli puree	Control	Broccoli puree	8.2939	14.1671	205	0.59	0.5589
Group*SampleName	Vegetable	Broccoli puree	Control	Chicken puree	25.6064	14.1671	205	1.81	0.0722
Group*SampleName	Vegetable	Broccoli puree	Control	Kale chopped	32.1064	14.1671	205	2.27	0.0245
Group*SampleName	Vegetable	Broccoli puree	Control	Kale puree	23.6689	14.1671	205	1.67	0.0963
Group*SampleName	Vegetable	Broccoli puree	Control	Oat puree	38.1689	14.1671	205	2.69	0.0076
Group*SampleName	Vegetable	Broccoli puree	Control	Spinach chopped	24.6064	14.1671	205	1.74	0.0839
Group*SampleName	Vegetable	Broccoli puree	Control	Spinach puree	25.1689	14.1671	205	1.78	0.0771
Group*SampleName	Vegetable	Chicken puree	Vegetable	Kale chopped	4.8889	11.7045	352	0.42	0.6764
Group*SampleName	Vegetable	Chicken puree	Vegetable	Kale puree	-9.0000	11.7045	352	-0.77	0.4424
Group*SampleName	Vegetable	Chicken puree	Vegetable	Oat puree	-22.1667	11.7045	352	-1.89	0.0591
Group*SampleName	Vegetable	Chicken puree	Vegetable	Spinach chopped	-20.8333	11.7045	352	-1.78	0.0759
Group*SampleName	Vegetable	Chicken puree	Vegetable	Spinach puree	-18.8333	11.7045	352	-1.61	0.1085
Group*SampleName	Vegetable	Chicken puree	Control	Asparagus chopped	-43.6436	14.1671	205	-3.08	0.0023
Group*SampleName	Vegetable	Chicken puree	Control	Asparagus puree	-37.8936	14.1671	205	-2.67	0.0081
Group*SampleName	Vegetable	Chicken puree	Control	Beef puree	-18.5811	14.1671	205	-1.31	0.1911
Group*SampleName	Vegetable	Chicken puree	Control	Black bean puree	-26.0811	14.1671	205	-1.84	0.0671
Group*SampleName	Vegetable	Chicken puree	Control	Broccoli chopped	-41.6436	14.1671	205	-2.94	0.0037
Group*SampleName	Vegetable	Chicken puree	Control	Broccoli puree	-35.7061	14.1671	205	-2.52	0.0125
Group*SampleName	Vegetable	Chicken puree	Control	Chicken puree	-18.3936	14.1671	205	-1.30	0.1956
Group*SampleName	Vegetable	Chicken puree	Control	Kale chopped	-11.8936	14.1671	205	-0.84	0.4022
Group*SampleName	Vegetable	Chicken puree	Control	Kale puree	-20.3311	14.1671	205	-1.44	0.1528
Group*SampleName	Vegetable	Chicken puree	Control	Oat puree	-5.8311	14.1671	205	-0.41	0.6811
Group*SampleName	Vegetable	Chicken puree	Control	Spinach chopped	-19.3936	14.1671	205	-1.37	0.1725
Group*SampleName	Vegetable	Chicken puree	Control	Spinach puree	-18.8311	14.1671	205	-1.33	0.1853
Group*SampleName	Vegetable	Kale chopped	Vegetable	Kale puree	-13.8889	11.7045	352	-1.19	0.2362
Group*SampleName	Vegetable	Kale chopped	Vegetable	Oat puree	-27.0556	11.7045	352	-2.31	0.0214
Group*SampleName	Vegetable	Kale chopped	Vegetable	Spinach chopped	-25.7222	11.7045	352	-2.20	0.0286
Group*SampleName	Vegetable	Kale chopped	Vegetable	Spinach puree	-23.7222	11.7045	352	-2.03	0.0434
Group*SampleName	Vegetable	Kale chopped	Control	Asparagus chopped	-48.5325	14.1671	205	-3.43	0.0007
Group*SampleName	Vegetable	Kale chopped	Control	Asparagus puree	-42.7825	14.1671	205	-3.02	0.0029
Group*SampleName	Vegetable	Kale chopped	Control	Beef puree	-23.4700	14.1671	205	-1.66	0.0991
Group*SampleName	Vegetable	Kale chopped	Control	Black bean puree	-30.9700	14.1671	205	-2.19	0.0299
Group*SampleName	Vegetable	Kale chopped	Control	Broccoli chopped	-46.5325	14.1671	205	-3.28	0.0012
Group*SampleName	Vegetable	Kale chopped	Control	Broccoli puree	-40.5950	14.1671	205	-2.87	0.0046
Group*SampleName	Vegetable	Kale chopped	Control	Chicken puree	-23.2825	14.1671	205	-1.64	0.1018
Group*SampleName	Vegetable	Kale chopped	Control	Kale chopped	-16.7825	14.1671	205	-1.18	0.2375
Group*SampleName	Vegetable	Kale chopped	Control	Kale puree	-25.2200	14.1671	205	-1.78	0.0765
Group*SampleName	Vegetable	Kale chopped	Control	Oat puree	-10.7200	14.1671	205	-0.76	0.4501
Group*SampleName	Vegetable	Kale chopped	Control	Spinach chopped	-24.2825	14.1671	205	-1.71	0.0880
Group*SampleName	Vegetable	Kale chopped	Control	Spinach puree	-23.7200	14.1671	205	-1.67	0.0956

Differences of Least Squares Means									
Effect	Group	SampleName	_Group	_SampleName	Estimate	Standard Error	DF	t Value	Pr > |t|
Group*SampleName	Vegetable	Kale puree	Vegetable	Oat puree	-13.1667	11.7045	352	-1.12	0.2614
Group*SampleName	Vegetable	Kale puree	Vegetable	Spinach chopped	-11.8333	11.7045	352	-1.01	0.3127
Group*SampleName	Vegetable	Kale puree	Vegetable	Spinach puree	-9.8333	11.7045	352	-0.84	0.4014
Group*SampleName	Vegetable	Kale puree	Control	Asparagus chopped	-34.6436	14.1671	205	-2.45	0.0153
Group*SampleName	Vegetable	Kale puree	Control	Asparagus puree	-28.8936	14.1671	205	-2.04	0.0427
Group*SampleName	Vegetable	Kale puree	Control	Beef puree	-9.5811	14.1671	205	-0.68	0.4996
Group*SampleName	Vegetable	Kale puree	Control	Black bean puree	-17.0811	14.1671	205	-1.21	0.2293
Group*SampleName	Vegetable	Kale puree	Control	Broccoli chopped	-32.6436	14.1671	205	-2.30	0.0222
Group*SampleName	Vegetable	Kale puree	Control	Broccoli puree	-26.7061	14.1671	205	-1.89	0.0608
Group*SampleName	Vegetable	Kale puree	Control	Chicken puree	-9.3936	14.1671	205	-0.66	0.5080
Group*SampleName	Vegetable	Kale puree	Control	Kale chopped	-2.8936	14.1671	205	-0.20	0.8384
Group*SampleName	Vegetable	Kale puree	Control	Kale puree	-11.3311	14.1671	205	-0.80	0.4247
Group*SampleName	Vegetable	Kale puree	Control	Oat puree	3.1689	14.1671	205	0.22	0.8232
Group*SampleName	Vegetable	Kale puree	Control	Spinach chopped	-10.3936	14.1671	205	-0.73	0.4640
Group*SampleName	Vegetable	Kale puree	Control	Spinach puree	-9.8311	14.1671	205	-0.69	0.4885
Group*SampleName	Vegetable	Oat puree	Vegetable	Spinach chopped	1.3333	11.7045	352	0.11	0.9094
Group*SampleName	Vegetable	Oat puree	Vegetable	Spinach puree	3.3333	11.7045	352	0.28	0.7760
Group*SampleName	Vegetable	Oat puree	Control	Asparagus chopped	-21.4770	14.1671	205	-1.52	0.1311
Group*SampleName	Vegetable	Oat puree	Control	Asparagus puree	-15.7270	14.1671	205	-1.11	0.2683
Group*SampleName	Vegetable	Oat puree	Control	Beef puree	3.5855	14.1671	205	0.25	0.8005
Group*SampleName	Vegetable	Oat puree	Control	Black bean puree	-3.9145	14.1671	205	-0.28	0.7826
Group*SampleName	Vegetable	Oat puree	Control	Broccoli chopped	-19.4770	14.1671	205	-1.37	0.1707
Group*SampleName	Vegetable	Oat puree	Control	Broccoli puree	-13.5395	14.1671	205	-0.96	0.3404
Group*SampleName	Vegetable	Oat puree	Control	Chicken puree	3.7730	14.1671	205	0.27	0.7903
Group*SampleName	Vegetable	Oat puree	Control	Kale chopped	10.2730	14.1671	205	0.73	0.4692
Group*SampleName	Vegetable	Oat puree	Control	Kale puree	1.8355	14.1671	205	0.13	0.8970
Group*SampleName	Vegetable	Oat puree	Control	Oat puree	16.3355	14.1671	205	1.15	0.2502
Group*SampleName	Vegetable	Oat puree	Control	Spinach chopped	2.7730	14.1671	205	0.20	0.8450
Group*SampleName	Vegetable	Oat puree	Control	Spinach puree	3.3355	14.1671	205	0.24	0.8141
Group*SampleName	Vegetable	Spinach chopped	Vegetable	Spinach puree	2.0000	11.7045	352	0.17	0.8644
Group*SampleName	Vegetable	Spinach chopped	Control	Asparagus chopped	-22.8103	14.1671	205	-1.61	0.1089
Group*SampleName	Vegetable	Spinach chopped	Control	Asparagus puree	-17.0603	14.1671	205	-1.20	0.2299
Group*SampleName	Vegetable	Spinach chopped	Control	Beef puree	2.2522	14.1671	205	0.16	0.8738
Group*SampleName	Vegetable	Spinach chopped	Control	Black bean puree	-5.2478	14.1671	205	-0.37	0.7115
Group*SampleName	Vegetable	Spinach chopped	Control	Broccoli chopped	-20.8103	14.1671	205	-1.47	0.1434
Group*SampleName	Vegetable	Spinach chopped	Control	Broccoli puree	-14.8728	14.1671	205	-1.05	0.2950
Group*SampleName	Vegetable	Spinach chopped	Control	Chicken puree	2.4397	14.1671	205	0.17	0.8634
Group*SampleName	Vegetable	Spinach chopped	Control	Kale chopped	8.9397	14.1671	205	0.63	0.5287
Group*SampleName	Vegetable	Spinach chopped	Control	Kale puree	0.5022	14.1671	205	0.04	0.9718
Group*SampleName	Vegetable	Spinach chopped	Control	Oat puree	15.0022	14.1671	205	1.06	0.2909
Group*SampleName	Vegetable	Spinach chopped	Control	Spinach chopped	1.4397	14.1671	205	0.10	0.9192
Group*SampleName	Vegetable	Spinach chopped	Control	Spinach puree	2.0022	14.1671	205	0.14	0.8878

Differences of Least Squares Means									
Effect	Group	SampleName	_Group	_SampleName	Estimate	Standard Error	DF	t Value	Pr > |t|
Group*SampleName	Vegetable	Spinach puree	Control	Asparagus chopped	-24.8103	14.1671	205	-1.75	0.0814
Group*SampleName	Vegetable	Spinach puree	Control	Asparagus puree	-19.0603	14.1671	205	-1.35	0.1800
Group*SampleName	Vegetable	Spinach puree	Control	Beef puree	0.2522	14.1671	205	0.02	0.9858
Group*SampleName	Vegetable	Spinach puree	Control	Black bean puree	-7.2478	14.1671	205	-0.51	0.6095
Group*SampleName	Vegetable	Spinach puree	Control	Broccoli chopped	-22.8103	14.1671	205	-1.61	0.1089
Group*SampleName	Vegetable	Spinach puree	Control	Broccoli puree	-16.8728	14.1671	205	-1.19	0.2350
Group*SampleName	Vegetable	Spinach puree	Control	Chicken puree	0.4397	14.1671	205	0.03	0.9753
Group*SampleName	Vegetable	Spinach puree	Control	Kale chopped	6.9397	14.1671	205	0.49	0.6248
Group*SampleName	Vegetable	Spinach puree	Control	Kale puree	-1.4978	14.1671	205	-0.11	0.9159
Group*SampleName	Vegetable	Spinach puree	Control	Oat puree	13.0022	14.1671	205	0.92	0.3598
Group*SampleName	Vegetable	Spinach puree	Control	Spinach chopped	-0.5603	14.1671	205	-0.04	0.9685
Group*SampleName	Vegetable	Spinach puree	Control	Spinach puree	0.002202	14.1671	205	0.00	0.9999
Group*SampleName	Control	Asparagus chopped	Control	Asparagus puree	5.7500	12.4145	352	0.46	0.6435
Group*SampleName	Control	Asparagus chopped	Control	Beef puree	25.0625	12.4145	352	2.02	0.0443
Group*SampleName	Control	Asparagus chopped	Control	Black bean puree	17.5625	12.4145	352	1.41	0.1580
Group*SampleName	Control	Asparagus chopped	Control	Broccoli chopped	2.0000	12.4145	352	0.16	0.8721
Group*SampleName	Control	Asparagus chopped	Control	Broccoli puree	7.9375	12.4145	352	0.64	0.5230
Group*SampleName	Control	Asparagus chopped	Control	Chicken puree	25.2500	12.4145	352	2.03	0.0427
Group*SampleName	Control	Asparagus chopped	Control	Kale chopped	31.7500	12.4145	352	2.56	0.0110
Group*SampleName	Control	Asparagus chopped	Control	Kale puree	23.3125	12.4145	352	1.88	0.0612
Group*SampleName	Control	Asparagus chopped	Control	Oat puree	37.8125	12.4145	352	3.05	0.0025
Group*SampleName	Control	Asparagus chopped	Control	Spinach chopped	24.2500	12.4145	352	1.95	0.0516
Group*SampleName	Control	Asparagus chopped	Control	Spinach puree	24.8125	12.4145	352	2.00	0.0464
Group*SampleName	Control	Asparagus puree	Control	Beef puree	19.3125	12.4145	352	1.56	0.1207
Group*SampleName	Control	Asparagus puree	Control	Black bean puree	11.8125	12.4145	352	0.95	0.3420
Group*SampleName	Control	Asparagus puree	Control	Broccoli chopped	-3.7500	12.4145	352	-0.30	0.7628
Group*SampleName	Control	Asparagus puree	Control	Broccoli puree	2.1875	12.4145	352	0.18	0.8602
Group*SampleName	Control	Asparagus puree	Control	Chicken puree	19.5000	12.4145	352	1.57	0.1171
Group*SampleName	Control	Asparagus puree	Control	Kale chopped	26.0000	12.4145	352	2.09	0.0369
Group*SampleName	Control	Asparagus puree	Control	Kale puree	17.5625	12.4145	352	1.41	0.1580
Group*SampleName	Control	Asparagus puree	Control	Oat puree	32.0625	12.4145	352	2.58	0.0102
Group*SampleName	Control	Asparagus puree	Control	Spinach chopped	18.5000	12.4145	352	1.49	0.1371
Group*SampleName	Control	Asparagus puree	Control	Spinach puree	19.0625	12.4145	352	1.54	0.1256
Group*SampleName	Control	Beef puree	Control	Black bean puree	-7.5000	12.4145	352	-0.60	0.5461
Group*SampleName	Control	Beef puree	Control	Broccoli chopped	-23.0625	12.4145	352	-1.86	0.0640
Group*SampleName	Control	Beef puree	Control	Broccoli puree	-17.1250	12.4145	352	-1.38	0.1686
Group*SampleName	Control	Beef puree	Control	Chicken puree	0.1875	12.4145	352	0.02	0.9880
Group*SampleName	Control	Beef puree	Control	Kale chopped	6.6875	12.4145	352	0.54	0.5904
Group*SampleName	Control	Beef puree	Control	Kale puree	-1.7500	12.4145	352	-0.14	0.8880
Group*SampleName	Control	Beef puree	Control	Oat puree	12.7500	12.4145	352	1.03	0.3051
Group*SampleName	Control	Beef puree	Control	Spinach chopped	-0.8125	12.4145	352	-0.07	0.9479
Group*SampleName	Control	Beef puree	Control	Spinach puree	-0.2500	12.4145	352	-0.02	0.9839

Differences of Least Squares Means									
Effect	Group	SampleName	_Group	_SampleName	Estimate	Standard Error	DF	t Value	Pr > |t|
Group*SampleName	Control	Black bean puree	Control	Broccoli chopped	-15.5625	12.4145	352	-1.25	0.2108
Group*SampleName	Control	Black bean puree	Control	Broccoli puree	-9.6250	12.4145	352	-0.78	0.4387
Group*SampleName	Control	Black bean puree	Control	Chicken puree	7.6875	12.4145	352	0.62	0.5362
Group*SampleName	Control	Black bean puree	Control	Kale chopped	14.1875	12.4145	352	1.14	0.2539
Group*SampleName	Control	Black bean puree	Control	Kale puree	5.7500	12.4145	352	0.46	0.6435
Group*SampleName	Control	Black bean puree	Control	Oat puree	20.2500	12.4145	352	1.63	0.1038
Group*SampleName	Control	Black bean puree	Control	Spinach chopped	6.6875	12.4145	352	0.54	0.5904
Group*SampleName	Control	Black bean puree	Control	Spinach puree	7.2500	12.4145	352	0.58	0.5596
Group*SampleName	Control	Broccoli chopped	Control	Broccoli puree	5.9375	12.4145	352	0.48	0.6328
Group*SampleName	Control	Broccoli chopped	Control	Chicken puree	23.2500	12.4145	352	1.87	0.0619
Group*SampleName	Control	Broccoli chopped	Control	Kale chopped	29.7500	12.4145	352	2.40	0.0171
Group*SampleName	Control	Broccoli chopped	Control	Kale puree	21.3125	12.4145	352	1.72	0.0869
Group*SampleName	Control	Broccoli chopped	Control	Oat puree	35.8125	12.4145	352	2.88	0.0042
Group*SampleName	Control	Broccoli chopped	Control	Spinach chopped	22.2500	12.4145	352	1.79	0.0739
Group*SampleName	Control	Broccoli chopped	Control	Spinach puree	22.8125	12.4145	352	1.84	0.0670
Group*SampleName	Control	Broccoli puree	Control	Chicken puree	17.3125	12.4145	352	1.39	0.1640
Group*SampleName	Control	Broccoli puree	Control	Kale chopped	23.8125	12.4145	352	1.92	0.0559
Group*SampleName	Control	Broccoli puree	Control	Kale puree	15.3750	12.4145	352	1.24	0.2164
Group*SampleName	Control	Broccoli puree	Control	Oat puree	29.8750	12.4145	352	2.41	0.0166
Group*SampleName	Control	Broccoli puree	Control	Spinach chopped	16.3125	12.4145	352	1.31	0.1897
Group*SampleName	Control	Broccoli puree	Control	Spinach puree	16.8750	12.4145	352	1.36	0.1749
Group*SampleName	Control	Chicken puree	Control	Kale chopped	6.5000	12.4145	352	0.52	0.6009
Group*SampleName	Control	Chicken puree	Control	Kale puree	-1.9375	12.4145	352	-0.16	0.8761
Group*SampleName	Control	Chicken puree	Control	Oat puree	12.5625	12.4145	352	1.01	0.3123
Group*SampleName	Control	Chicken puree	Control	Spinach chopped	-1.0000	12.4145	352	-0.08	0.9358
Group*SampleName	Control	Chicken puree	Control	Spinach puree	-0.4375	12.4145	352	-0.04	0.9719
Group*SampleName	Control	Kale chopped	Control	Kale puree	-8.4375	12.4145	352	-0.68	0.4972
Group*SampleName	Control	Kale chopped	Control	Oat puree	6.0625	12.4145	352	0.49	0.6256
Group*SampleName	Control	Kale chopped	Control	Spinach chopped	-7.5000	12.4145	352	-0.60	0.5461
Group*SampleName	Control	Kale chopped	Control	Spinach puree	-6.9375	12.4145	352	-0.56	0.5766
Group*SampleName	Control	Kale puree	Control	Oat puree	14.5000	12.4145	352	1.17	0.2436
Group*SampleName	Control	Kale puree	Control	Spinach chopped	0.9375	12.4145	352	0.08	0.9398
Group*SampleName	Control	Kale puree	Control	Spinach puree	1.5000	12.4145	352	0.12	0.9039
Group*SampleName	Control	Oat puree	Control	Spinach chopped	-13.5625	12.4145	352	-1.09	0.2754
Group*SampleName	Control	Oat puree	Control	Spinach puree	-13.0000	12.4145	352	-1.05	0.2957
Group*SampleName	Control	Spinach chopped	Control	Spinach puree	0.5625	12.4145	352	0.05	0.9639

Secondard analysis: Liking between vegetables

The Mixed Procedure

GameVisit=2

Model Information	
Data Set	WORK.GAMEVISITS
Dependent Variable	Liking
Covariance Structure	Compound Symmetry
Subject Effect	ParticipantID
Estimation Method	REML
Residual Variance Method	Profile
Fixed Effects SE Method	Kenward-Roger
Degrees of Freedom Method	Kenward-Roger

Class Level Information		
Class	Levels	Values
ParticipantID	34	1001 1002 1005 1006 1009 1010 1014 1016 1019 1020 1024 1025 1027 1029 1030 1034 1035 1041 1045 1046 1048 1049 1050 1051 1056 1061 1063 1064 1065 1066 1068 1069 1074 1078
GameVisit	1	2
Group	2	Vegetable Control
Gender	2	Man Woman
SampleName	12	Asparagus chopped Asparagus puree Beef puree Black bean puree Broccoli chopped Broccoli puree Chicken puree Kale chopped Kale puree Oat puree Spinach chopped Spinach puree

Dimensions	
Covariance Parameters	2
Columns in X	41
Columns in Z	0
Subjects	34
Max Obs per Subject	12

Number of Observations	
Number of Observations Read	408
Number of Observations Used	408
Number of Observations Not Used	0

Iteration History			
Iteration	Evaluations	-2 Res Log Like	Criterion
0	1	3979.23841475	
1	1	3918.14118954	0.00000000

Convergence criteria met.

Covariance Parameter Estimates		
Cov Parm	Subject	Estimate
CS	ParticipantID	412.28
Residual		1175.86

Fit Statistics	
-2 Res Log Likelihood	3918.1
AIC (Smaller is Better)	3922.1

Fit Statistics	
AICC (Smaller is Better)	3922.2
BIC (Smaller is Better)	3925.2

Null Model Likelihood Ratio Test		
DF	Chi-Square	Pr > ChiSq
1	61.10	<.0001

Solution for Fixed Effects								
Effect	Group	Gender	SampleName	Estimate	Standard Error	DF	t Value	Pr > |t|
Intercept				-12.3458	10.4746	170	-1.18	0.2402
SampleName			Asparagus chopped	23.2500	12.1236	352	1.92	0.0560
SampleName			Asparagus puree	28.0625	12.1236	352	2.31	0.0212
SampleName			Beef puree	6.3750	12.1236	352	0.53	0.5993
SampleName			Black bean puree	22.1250	12.1236	352	1.82	0.0689
SampleName			Broccoli chopped	35.0000	12.1236	352	2.89	0.0041
SampleName			Broccoli puree	14.6250	12.1236	352	1.21	0.2285
SampleName			Chicken puree	9.9375	12.1236	352	0.82	0.4130
SampleName			Kale chopped	-10.1250	12.1236	352	-0.84	0.4042
SampleName			Kale puree	-9.3125	12.1236	352	-0.77	0.4429
SampleName			Oat puree	-2.4375	12.1236	352	-0.20	0.8408
SampleName			Spinach chopped	2.1250	12.1236	352	0.18	0.8610
SampleName			Spinach puree	0	.	.	.	.
Group	Vegetable			-0.7665	13.7559	211	-0.06	0.9556
Group	Control			0	.	.	.	.
Group*SampleName	Vegetable		Asparagus chopped	-0.2500	16.6623	352	-0.02	0.9880
Group*SampleName	Vegetable		Asparagus puree	-18.2292	16.6623	352	-1.09	0.2747
Group*SampleName	Vegetable		Beef puree	-22.2083	16.6623	352	-1.33	0.1834
Group*SampleName	Vegetable		Black bean puree	-17.0694	16.6623	352	-1.02	0.3063
Group*SampleName	Vegetable		Broccoli chopped	-9.8889	16.6623	352	-0.59	0.5532
Group*SampleName	Vegetable		Broccoli puree	11.1528	16.6623	352	0.67	0.5037
Group*SampleName	Vegetable		Chicken puree	-15.2708	16.6623	352	-0.92	0.3600
Group*SampleName	Vegetable		Kale chopped	8.4028	16.6623	352	0.50	0.6144
Group*SampleName	Vegetable		Kale puree	6.7014	16.6623	352	0.40	0.6878
Group*SampleName	Vegetable		Oat puree	11.9375	16.6623	352	0.72	0.4742
Group*SampleName	Vegetable		Spinach chopped	3.8194	16.6623	352	0.23	0.8188
Group*SampleName	Vegetable		Spinach puree	0	.	.	.	.
Group*SampleName	Control		Asparagus chopped	0	.	.	.	.
Group*SampleName	Control		Asparagus puree	0	.	.	.	.
Group*SampleName	Control		Beef puree	0	.	.	.	.
Group*SampleName	Control		Black bean puree	0	.	.	.	.
Group*SampleName	Control		Broccoli chopped	0	.	.	.	.
Group*SampleName	Control		Broccoli puree	0	.	.	.	.
Group*SampleName	Control		Chicken puree	0	.	.	.	.

Solution for Fixed Effects								
Effect	Group	Gender	SampleName	Estimate	Standard Error	DF	t Value	Pr > |t|
Group*SampleName	Control		Kale chopped	0	.	.	.	.
Group*SampleName	Control		Kale puree	0	.	.	.	.
Group*SampleName	Control		Oat puree	0	.	.	.	.
Group*SampleName	Control		Spinach chopped	0	.	.	.	.
Group*SampleName	Control		Spinach puree	0	.	.	.	.
Gender		Man		2.2554	8.6239	31	0.26	0.7954
Gender		Woman		0	.	.	.	.

Type 3 Tests of Fixed Effects				
Effect	Num DF	Den DF	F Value	Pr > F
SampleName	11	352	4.45	<.0001
Group	1	31	0.28	0.5997
Group*SampleName	11	352	1.12	0.3466
Gender	1	31	0.07	0.7954

Least Squares Means							
Effect	Group	SampleName	Estimate	Standard Error	DF	t Value	Pr > |t|
Group	Vegetable		-5.4244	5.8384	31	-0.93	0.3600
Group	Control		-1.2493	5.7492	31	-0.22	0.8294
SampleName		Asparagus chopped	11.5237	7.0632	186	1.63	0.1045
SampleName		Asparagus puree	7.3466	7.0632	186	1.04	0.2996
SampleName		Beef puree	-16.3305	7.0632	186	-2.31	0.0219
SampleName		Black bean puree	1.9889	7.0632	186	0.28	0.7786
SampleName		Broccoli chopped	18.4542	7.0632	186	2.61	0.0097
SampleName		Broccoli puree	8.6000	7.0632	186	1.22	0.2249
SampleName		Chicken puree	-9.2993	7.0632	186	-1.32	0.1896
SampleName		Kale chopped	-17.5250	7.0632	186	-2.48	0.0140
SampleName		Kale puree	-17.5631	7.0632	186	-2.49	0.0138
SampleName		Oat puree	-8.0701	7.0632	186	-1.14	0.2547
SampleName		Spinach chopped	-7.5666	7.0632	186	-1.07	0.2854
SampleName		Spinach puree	-11.6013	7.0632	186	-1.64	0.1022
Group*SampleName	Vegetable	Asparagus chopped	11.0154	9.6937	185	1.14	0.2573
Group*SampleName	Vegetable	Asparagus puree	-2.1513	9.6937	185	-0.22	0.8246
Group*SampleName	Vegetable	Beef puree	-27.8179	9.6937	185	-2.87	0.0046
Group*SampleName	Vegetable	Black bean puree	-6.9291	9.6937	185	-0.71	0.4756
Group*SampleName	Vegetable	Broccoli chopped	13.1265	9.6937	185	1.35	0.1773
Group*SampleName	Vegetable	Broccoli puree	13.7932	9.6937	185	1.42	0.1564
Group*SampleName	Vegetable	Chicken puree	-17.3179	9.6937	185	-1.79	0.0757
Group*SampleName	Vegetable	Kale chopped	-13.7068	9.6937	185	-1.41	0.1590
Group*SampleName	Vegetable	Kale puree	-14.5957	9.6937	185	-1.51	0.1339
Group*SampleName	Vegetable	Oat puree	-2.4846	9.6937	185	-0.26	0.7980
Group*SampleName	Vegetable	Spinach chopped	-6.0402	9.6937	185	-0.62	0.5340

Least Squares Means							
Effect	Group	SampleName	Estimate	Standard Error	DF	t Value	Pr > |t|
Group*SampleName	Vegetable	Spinach puree	-11.9846	9.6937	185	-1.24	0.2179
Group*SampleName	Control	Asparagus chopped	12.0319	10.0210	209	1.20	0.2312
Group*SampleName	Control	Asparagus puree	16.8444	10.0210	209	1.68	0.0943
Group*SampleName	Control	Beef puree	-4.8431	10.0210	209	-0.48	0.6294
Group*SampleName	Control	Black bean puree	10.9069	10.0210	209	1.09	0.2777
Group*SampleName	Control	Broccoli chopped	23.7819	10.0210	209	2.37	0.0185
Group*SampleName	Control	Broccoli puree	3.4069	10.0210	209	0.34	0.7342
Group*SampleName	Control	Chicken puree	-1.2806	10.0210	209	-0.13	0.8984
Group*SampleName	Control	Kale chopped	-21.3431	10.0210	209	-2.13	0.0344
Group*SampleName	Control	Kale puree	-20.5306	10.0210	209	-2.05	0.0417
Group*SampleName	Control	Oat puree	-13.6556	10.0210	209	-1.36	0.1744
Group*SampleName	Control	Spinach chopped	-9.0931	10.0210	209	-0.91	0.3652
Group*SampleName	Control	Spinach puree	-11.2181	10.0210	209	-1.12	0.2642

Differences of Least Squares Means									
Effect	Group	SampleName	_Group	_SampleName	Estimate	Standard Error	DF	t Value	Pr > |t|
Group	Vegetable		Control		-4.1751	7.8725	31	-0.53	0.5997
SampleName		Asparagus chopped		Asparagus puree	4.1771	8.3312	352	0.50	0.6164
SampleName		Asparagus chopped		Beef puree	27.8542	8.3312	352	3.34	0.0009
SampleName		Asparagus chopped		Black bean puree	9.5347	8.3312	352	1.14	0.2532
SampleName		Asparagus chopped		Broccoli chopped	-6.9306	8.3312	352	-0.83	0.4060
SampleName		Asparagus chopped		Broccoli puree	2.9236	8.3312	352	0.35	0.7259
SampleName		Asparagus chopped		Chicken puree	20.8229	8.3312	352	2.50	0.0129
SampleName		Asparagus chopped		Kale chopped	29.0486	8.3312	352	3.49	0.0006
SampleName		Asparagus chopped		Kale puree	29.0868	8.3312	352	3.49	0.0005
SampleName		Asparagus chopped		Oat puree	19.5938	8.3312	352	2.35	0.0192
SampleName		Asparagus chopped		Spinach chopped	19.0903	8.3312	352	2.29	0.0225
SampleName		Asparagus chopped		Spinach puree	23.1250	8.3312	352	2.78	0.0058
SampleName		Asparagus puree		Beef puree	23.6771	8.3312	352	2.84	0.0047
SampleName		Asparagus puree		Black bean puree	5.3576	8.3312	352	0.64	0.5206
SampleName		Asparagus puree		Broccoli chopped	-11.1076	8.3312	352	-1.33	0.1833
SampleName		Asparagus puree		Broccoli puree	-1.2535	8.3312	352	-0.15	0.8805
SampleName		Asparagus puree		Chicken puree	16.6458	8.3312	352	2.00	0.0465
SampleName		Asparagus puree		Kale chopped	24.8715	8.3312	352	2.99	0.0030
SampleName		Asparagus puree		Kale puree	24.9097	8.3312	352	2.99	0.0030
SampleName		Asparagus puree		Oat puree	15.4167	8.3312	352	1.85	0.0651
SampleName		Asparagus puree		Spinach chopped	14.9132	8.3312	352	1.79	0.0743
SampleName		Asparagus puree		Spinach puree	18.9479	8.3312	352	2.27	0.0235
SampleName		Beef puree		Black bean puree	-18.3194	8.3312	352	-2.20	0.0285
SampleName		Beef puree		Broccoli chopped	-34.7847	8.3312	352	-4.18	<.0001
SampleName		Beef puree		Broccoli puree	-24.9306	8.3312	352	-2.99	0.0030
SampleName		Beef puree		Chicken puree	-7.0312	8.3312	352	-0.84	0.3993

Differences of Least Squares Means									
Effect	Group	SampleName	_Group	_SampleName	Estimate	Standard Error	DF	t Value	Pr > |t|
SampleName		Beef puree		Kale chopped	1.1944	8.3312	352	0.14	0.8861
SampleName		Beef puree		Kale puree	1.2326	8.3312	352	0.15	0.8825
SampleName		Beef puree		Oat puree	-8.2604	8.3312	352	-0.99	0.3221
SampleName		Beef puree		Spinach chopped	-8.7639	8.3312	352	-1.05	0.2935
SampleName		Beef puree		Spinach puree	-4.7292	8.3312	352	-0.57	0.5706
SampleName		Black bean puree		Broccoli chopped	-16.4653	8.3312	352	-1.98	0.0489
SampleName		Black bean puree		Broccoli puree	-6.6111	8.3312	352	-0.79	0.4280
SampleName		Black bean puree		Chicken puree	11.2882	8.3312	352	1.35	0.1763
SampleName		Black bean puree		Kale chopped	19.5139	8.3312	352	2.34	0.0197
SampleName		Black bean puree		Kale puree	19.5521	8.3312	352	2.35	0.0195
SampleName		Black bean puree		Oat puree	10.0590	8.3312	352	1.21	0.2281
SampleName		Black bean puree		Spinach chopped	9.5556	8.3312	352	1.15	0.2522
SampleName		Black bean puree		Spinach puree	13.5903	8.3312	352	1.63	0.1037
SampleName		Broccoli chopped		Broccoli puree	9.8542	8.3312	352	1.18	0.2377
SampleName		Broccoli chopped		Chicken puree	27.7535	8.3312	352	3.33	0.0010
SampleName		Broccoli chopped		Kale chopped	35.9792	8.3312	352	4.32	<.0001
SampleName		Broccoli chopped		Kale puree	36.0174	8.3312	352	4.32	<.0001
SampleName		Broccoli chopped		Oat puree	26.5243	8.3312	352	3.18	0.0016
SampleName		Broccoli chopped		Spinach chopped	26.0208	8.3312	352	3.12	0.0019
SampleName		Broccoli chopped		Spinach puree	30.0556	8.3312	352	3.61	0.0004
SampleName		Broccoli puree		Chicken puree	17.8993	8.3312	352	2.15	0.0324
SampleName		Broccoli puree		Kale chopped	26.1250	8.3312	352	3.14	0.0019
SampleName		Broccoli puree		Kale puree	26.1632	8.3312	352	3.14	0.0018
SampleName		Broccoli puree		Oat puree	16.6701	8.3312	352	2.00	0.0462
SampleName		Broccoli puree		Spinach chopped	16.1667	8.3312	352	1.94	0.0531
SampleName		Broccoli puree		Spinach puree	20.2014	8.3312	352	2.42	0.0158
SampleName		Chicken puree		Kale chopped	8.2257	8.3312	352	0.99	0.3242
SampleName		Chicken puree		Kale puree	8.2639	8.3312	352	0.99	0.3219
SampleName		Chicken puree		Oat puree	-1.2292	8.3312	352	-0.15	0.8828
SampleName		Chicken puree		Spinach chopped	-1.7326	8.3312	352	-0.21	0.8354
SampleName		Chicken puree		Spinach puree	2.3021	8.3312	352	0.28	0.7825
SampleName		Kale chopped		Kale puree	0.03819	8.3312	352	0.00	0.9963
SampleName		Kale chopped		Oat puree	-9.4549	8.3312	352	-1.13	0.2572
SampleName		Kale chopped		Spinach chopped	-9.9583	8.3312	352	-1.20	0.2328
SampleName		Kale chopped		Spinach puree	-5.9236	8.3312	352	-0.71	0.4775
SampleName		Kale puree		Oat puree	-9.4931	8.3312	352	-1.14	0.2553
SampleName		Kale puree		Spinach chopped	-9.9965	8.3312	352	-1.20	0.2310
SampleName		Kale puree		Spinach puree	-5.9618	8.3312	352	-0.72	0.4747
SampleName		Oat puree		Spinach chopped	-0.5035	8.3312	352	-0.06	0.9518
SampleName		Oat puree		Spinach puree	3.5312	8.3312	352	0.42	0.6719
SampleName		Spinach chopped		Spinach puree	4.0347	8.3312	352	0.48	0.6285
Group*SampleName	Vegetable	Asparagus chopped	Vegetable	Asparagus puree	13.1667	11.4303	352	1.15	0.2501

Differences of Least Squares Means									
Effect	Group	SampleName	_Group	_SampleName	Estimate	Standard Error	DF	t Value	Pr > |t|
Group*SampleName	Vegetable	Asparagus chopped	Vegetable	Beef puree	38.8333	11.4303	352	3.40	0.0008
Group*SampleName	Vegetable	Asparagus chopped	Vegetable	Black bean puree	17.9444	11.4303	352	1.57	0.1173
Group*SampleName	Vegetable	Asparagus chopped	Vegetable	Broccoli chopped	-2.1111	11.4303	352	-0.18	0.8536
Group*SampleName	Vegetable	Asparagus chopped	Vegetable	Broccoli puree	-2.7778	11.4303	352	-0.24	0.8081
Group*SampleName	Vegetable	Asparagus chopped	Vegetable	Chicken puree	28.3333	11.4303	352	2.48	0.0137
Group*SampleName	Vegetable	Asparagus chopped	Vegetable	Kale chopped	24.7222	11.4303	352	2.16	0.0312
Group*SampleName	Vegetable	Asparagus chopped	Vegetable	Kale puree	25.6111	11.4303	352	2.24	0.0257
Group*SampleName	Vegetable	Asparagus chopped	Vegetable	Oat puree	13.5000	11.4303	352	1.18	0.2384
Group*SampleName	Vegetable	Asparagus chopped	Vegetable	Spinach chopped	17.0556	11.4303	352	1.49	0.1366
Group*SampleName	Vegetable	Asparagus chopped	Vegetable	Spinach puree	23.0000	11.4303	352	2.01	0.0450
Group*SampleName	Vegetable	Asparagus chopped	Control	Asparagus chopped	-1.0165	13.7559	211	-0.07	0.9412
Group*SampleName	Vegetable	Asparagus chopped	Control	Asparagus puree	-5.8290	13.7559	211	-0.42	0.6722
Group*SampleName	Vegetable	Asparagus chopped	Control	Beef puree	15.8585	13.7559	211	1.15	0.2503
Group*SampleName	Vegetable	Asparagus chopped	Control	Black bean puree	0.1085	13.7559	211	0.01	0.9937
Group*SampleName	Vegetable	Asparagus chopped	Control	Broccoli chopped	-12.7665	13.7559	211	-0.93	0.3544
Group*SampleName	Vegetable	Asparagus chopped	Control	Broccoli puree	7.6085	13.7559	211	0.55	0.5808
Group*SampleName	Vegetable	Asparagus chopped	Control	Chicken puree	12.2960	13.7559	211	0.89	0.3724
Group*SampleName	Vegetable	Asparagus chopped	Control	Kale chopped	32.3585	13.7559	211	2.35	0.0196
Group*SampleName	Vegetable	Asparagus chopped	Control	Kale puree	31.5460	13.7559	211	2.29	0.0228
Group*SampleName	Vegetable	Asparagus chopped	Control	Oat puree	24.6710	13.7559	211	1.79	0.0743
Group*SampleName	Vegetable	Asparagus chopped	Control	Spinach chopped	20.1085	13.7559	211	1.46	0.1453
Group*SampleName	Vegetable	Asparagus chopped	Control	Spinach puree	22.2335	13.7559	211	1.62	0.1075
Group*SampleName	Vegetable	Asparagus puree	Vegetable	Beef puree	25.6667	11.4303	352	2.25	0.0254
Group*SampleName	Vegetable	Asparagus puree	Vegetable	Black bean puree	4.7778	11.4303	352	0.42	0.6762
Group*SampleName	Vegetable	Asparagus puree	Vegetable	Broccoli chopped	-15.2778	11.4303	352	-1.34	0.1822
Group*SampleName	Vegetable	Asparagus puree	Vegetable	Broccoli puree	-15.9444	11.4303	352	-1.39	0.1639
Group*SampleName	Vegetable	Asparagus puree	Vegetable	Chicken puree	15.1667	11.4303	352	1.33	0.1854
Group*SampleName	Vegetable	Asparagus puree	Vegetable	Kale chopped	11.5556	11.4303	352	1.01	0.3127
Group*SampleName	Vegetable	Asparagus puree	Vegetable	Kale puree	12.4444	11.4303	352	1.09	0.2770
Group*SampleName	Vegetable	Asparagus puree	Vegetable	Oat puree	0.3333	11.4303	352	0.03	0.9768
Group*SampleName	Vegetable	Asparagus puree	Vegetable	Spinach chopped	3.8889	11.4303	352	0.34	0.7339
Group*SampleName	Vegetable	Asparagus puree	Vegetable	Spinach puree	9.8333	11.4303	352	0.86	0.3902
Group*SampleName	Vegetable	Asparagus puree	Control	Asparagus chopped	-14.1832	13.7559	211	-1.03	0.3037
Group*SampleName	Vegetable	Asparagus puree	Control	Asparagus puree	-18.9957	13.7559	211	-1.38	0.1688
Group*SampleName	Vegetable	Asparagus puree	Control	Beef puree	2.6918	13.7559	211	0.20	0.8450
Group*SampleName	Vegetable	Asparagus puree	Control	Black bean puree	-13.0582	13.7559	211	-0.95	0.3436
Group*SampleName	Vegetable	Asparagus puree	Control	Broccoli chopped	-25.9332	13.7559	211	-1.89	0.0608
Group*SampleName	Vegetable	Asparagus puree	Control	Broccoli puree	-5.5582	13.7559	211	-0.40	0.6866
Group*SampleName	Vegetable	Asparagus puree	Control	Chicken puree	-0.8707	13.7559	211	-0.06	0.9496
Group*SampleName	Vegetable	Asparagus puree	Control	Kale chopped	19.1918	13.7559	211	1.40	0.1644
Group*SampleName	Vegetable	Asparagus puree	Control	Kale puree	18.3793	13.7559	211	1.34	0.1830
Group*SampleName	Vegetable	Asparagus puree	Control	Oat puree	11.5043	13.7559	211	0.84	0.4039

Differences of Least Squares Means									
Effect	Group	SampleName	_Group	_SampleName	Estimate	Standard Error	DF	t Value	Pr > |t|
Group*SampleName	Vegetable	Asparagus puree	Control	Spinach chopped	6.9418	13.7559	211	0.50	0.6143
Group*SampleName	Vegetable	Asparagus puree	Control	Spinach puree	9.0668	13.7559	211	0.66	0.5105
Group*SampleName	Vegetable	Beef puree	Vegetable	Black bean puree	-20.8889	11.4303	352	-1.83	0.0685
Group*SampleName	Vegetable	Beef puree	Vegetable	Broccoli chopped	-40.9444	11.4303	352	-3.58	0.0004
Group*SampleName	Vegetable	Beef puree	Vegetable	Broccoli puree	-41.6111	11.4303	352	-3.64	0.0003
Group*SampleName	Vegetable	Beef puree	Vegetable	Chicken puree	-10.5000	11.4303	352	-0.92	0.3589
Group*SampleName	Vegetable	Beef puree	Vegetable	Kale chopped	-14.1111	11.4303	352	-1.23	0.2178
Group*SampleName	Vegetable	Beef puree	Vegetable	Kale puree	-13.2222	11.4303	352	-1.16	0.2481
Group*SampleName	Vegetable	Beef puree	Vegetable	Oat puree	-25.3333	11.4303	352	-2.22	0.0273
Group*SampleName	Vegetable	Beef puree	Vegetable	Spinach chopped	-21.7778	11.4303	352	-1.91	0.0576
Group*SampleName	Vegetable	Beef puree	Vegetable	Spinach puree	-15.8333	11.4303	352	-1.39	0.1669
Group*SampleName	Vegetable	Beef puree	Control	Asparagus chopped	-39.8499	13.7559	211	-2.90	0.0042
Group*SampleName	Vegetable	Beef puree	Control	Asparagus puree	-44.6624	13.7559	211	-3.25	0.0014
Group*SampleName	Vegetable	Beef puree	Control	Beef puree	-22.9749	13.7559	211	-1.67	0.0964
Group*SampleName	Vegetable	Beef puree	Control	Black bean puree	-38.7249	13.7559	211	-2.82	0.0053
Group*SampleName	Vegetable	Beef puree	Control	Broccoli chopped	-51.5999	13.7559	211	-3.75	0.0002
Group*SampleName	Vegetable	Beef puree	Control	Broccoli puree	-31.2249	13.7559	211	-2.27	0.0242
Group*SampleName	Vegetable	Beef puree	Control	Chicken puree	-26.5374	13.7559	211	-1.93	0.0551
Group*SampleName	Vegetable	Beef puree	Control	Kale chopped	-6.4749	13.7559	211	-0.47	0.6383
Group*SampleName	Vegetable	Beef puree	Control	Kale puree	-7.2874	13.7559	211	-0.53	0.5968
Group*SampleName	Vegetable	Beef puree	Control	Oat puree	-14.1624	13.7559	211	-1.03	0.3044
Group*SampleName	Vegetable	Beef puree	Control	Spinach chopped	-18.7249	13.7559	211	-1.36	0.1749
Group*SampleName	Vegetable	Beef puree	Control	Spinach puree	-16.5999	13.7559	211	-1.21	0.2289
Group*SampleName	Vegetable	Black bean puree	Vegetable	Broccoli chopped	-20.0556	11.4303	352	-1.75	0.0802
Group*SampleName	Vegetable	Black bean puree	Vegetable	Broccoli puree	-20.7222	11.4303	352	-1.81	0.0707
Group*SampleName	Vegetable	Black bean puree	Vegetable	Chicken puree	10.3889	11.4303	352	0.91	0.3640
Group*SampleName	Vegetable	Black bean puree	Vegetable	Kale chopped	6.7778	11.4303	352	0.59	0.5536
Group*SampleName	Vegetable	Black bean puree	Vegetable	Kale puree	7.6667	11.4303	352	0.67	0.5028
Group*SampleName	Vegetable	Black bean puree	Vegetable	Oat puree	-4.4444	11.4303	352	-0.39	0.6976
Group*SampleName	Vegetable	Black bean puree	Vegetable	Spinach chopped	-0.8889	11.4303	352	-0.08	0.9381
Group*SampleName	Vegetable	Black bean puree	Vegetable	Spinach puree	5.0556	11.4303	352	0.44	0.6585
Group*SampleName	Vegetable	Black bean puree	Control	Asparagus chopped	-18.9610	13.7559	211	-1.38	0.1695
Group*SampleName	Vegetable	Black bean puree	Control	Asparagus puree	-23.7735	13.7559	211	-1.73	0.0854
Group*SampleName	Vegetable	Black bean puree	Control	Beef puree	-2.0860	13.7559	211	-0.15	0.8796
Group*SampleName	Vegetable	Black bean puree	Control	Black bean puree	-17.8360	13.7559	211	-1.30	0.1962
Group*SampleName	Vegetable	Black bean puree	Control	Broccoli chopped	-30.7110	13.7559	211	-2.23	0.0266
Group*SampleName	Vegetable	Black bean puree	Control	Broccoli puree	-10.3360	13.7559	211	-0.75	0.4533
Group*SampleName	Vegetable	Black bean puree	Control	Chicken puree	-5.6485	13.7559	211	-0.41	0.6818
Group*SampleName	Vegetable	Black bean puree	Control	Kale chopped	14.4140	13.7559	211	1.05	0.2959
Group*SampleName	Vegetable	Black bean puree	Control	Kale puree	13.6015	13.7559	211	0.99	0.3239
Group*SampleName	Vegetable	Black bean puree	Control	Oat puree	6.7265	13.7559	211	0.49	0.6254
Group*SampleName	Vegetable	Black bean puree	Control	Spinach chopped	2.1640	13.7559	211	0.16	0.8751

Differences of Least Squares Means									
Effect	Group	SampleName	_Group	_SampleName	Estimate	Standard Error	DF	t Value	Pr > |t|
Group*SampleName	Vegetable	Black bean puree	Control	Spinach puree	4.2890	13.7559	211	0.31	0.7555
Group*SampleName	Vegetable	Broccoli chopped	Vegetable	Broccoli puree	-0.6667	11.4303	352	-0.06	0.9535
Group*SampleName	Vegetable	Broccoli chopped	Vegetable	Chicken puree	30.4444	11.4303	352	2.66	0.0081
Group*SampleName	Vegetable	Broccoli chopped	Vegetable	Kale chopped	26.8333	11.4303	352	2.35	0.0194
Group*SampleName	Vegetable	Broccoli chopped	Vegetable	Kale puree	27.7222	11.4303	352	2.43	0.0158
Group*SampleName	Vegetable	Broccoli chopped	Vegetable	Oat puree	15.6111	11.4303	352	1.37	0.1729
Group*SampleName	Vegetable	Broccoli chopped	Vegetable	Spinach chopped	19.1667	11.4303	352	1.68	0.0945
Group*SampleName	Vegetable	Broccoli chopped	Vegetable	Spinach puree	25.1111	11.4303	352	2.20	0.0287
Group*SampleName	Vegetable	Broccoli chopped	Control	Asparagus chopped	1.0946	13.7559	211	0.08	0.9367
Group*SampleName	Vegetable	Broccoli chopped	Control	Asparagus puree	-3.7179	13.7559	211	-0.27	0.7872
Group*SampleName	Vegetable	Broccoli chopped	Control	Beef puree	17.9696	13.7559	211	1.31	0.1929
Group*SampleName	Vegetable	Broccoli chopped	Control	Black bean puree	2.2196	13.7559	211	0.16	0.8720
Group*SampleName	Vegetable	Broccoli chopped	Control	Broccoli chopped	-10.6554	13.7559	211	-0.77	0.4394
Group*SampleName	Vegetable	Broccoli chopped	Control	Broccoli puree	9.7196	13.7559	211	0.71	0.4806
Group*SampleName	Vegetable	Broccoli chopped	Control	Chicken puree	14.4071	13.7559	211	1.05	0.2961
Group*SampleName	Vegetable	Broccoli chopped	Control	Kale chopped	34.4696	13.7559	211	2.51	0.0130
Group*SampleName	Vegetable	Broccoli chopped	Control	Kale puree	33.6571	13.7559	211	2.45	0.0152
Group*SampleName	Vegetable	Broccoli chopped	Control	Oat puree	26.7821	13.7559	211	1.95	0.0529
Group*SampleName	Vegetable	Broccoli chopped	Control	Spinach chopped	22.2196	13.7559	211	1.62	0.1077
Group*SampleName	Vegetable	Broccoli chopped	Control	Spinach puree	24.3446	13.7559	211	1.77	0.0782
Group*SampleName	Vegetable	Broccoli puree	Vegetable	Chicken puree	31.1111	11.4303	352	2.72	0.0068
Group*SampleName	Vegetable	Broccoli puree	Vegetable	Kale chopped	27.5000	11.4303	352	2.41	0.0166
Group*SampleName	Vegetable	Broccoli puree	Vegetable	Kale puree	28.3889	11.4303	352	2.48	0.0135
Group*SampleName	Vegetable	Broccoli puree	Vegetable	Oat puree	16.2778	11.4303	352	1.42	0.1553
Group*SampleName	Vegetable	Broccoli puree	Vegetable	Spinach chopped	19.8333	11.4303	352	1.74	0.0836
Group*SampleName	Vegetable	Broccoli puree	Vegetable	Spinach puree	25.7778	11.4303	352	2.26	0.0247
Group*SampleName	Vegetable	Broccoli puree	Control	Asparagus chopped	1.7612	13.7559	211	0.13	0.8982
Group*SampleName	Vegetable	Broccoli puree	Control	Asparagus puree	-3.0513	13.7559	211	-0.22	0.8247
Group*SampleName	Vegetable	Broccoli puree	Control	Beef puree	18.6362	13.7559	211	1.35	0.1769
Group*SampleName	Vegetable	Broccoli puree	Control	Black bean puree	2.8862	13.7559	211	0.21	0.8340
Group*SampleName	Vegetable	Broccoli puree	Control	Broccoli chopped	-9.9888	13.7559	211	-0.73	0.4686
Group*SampleName	Vegetable	Broccoli puree	Control	Broccoli puree	10.3862	13.7559	211	0.76	0.4511
Group*SampleName	Vegetable	Broccoli puree	Control	Chicken puree	15.0737	13.7559	211	1.10	0.2744
Group*SampleName	Vegetable	Broccoli puree	Control	Kale chopped	35.1362	13.7559	211	2.55	0.0113
Group*SampleName	Vegetable	Broccoli puree	Control	Kale puree	34.3237	13.7559	211	2.50	0.0134
Group*SampleName	Vegetable	Broccoli puree	Control	Oat puree	27.4487	13.7559	211	2.00	0.0473
Group*SampleName	Vegetable	Broccoli puree	Control	Spinach chopped	22.8862	13.7559	211	1.66	0.0977
Group*SampleName	Vegetable	Broccoli puree	Control	Spinach puree	25.0112	13.7559	211	1.82	0.0705
Group*SampleName	Vegetable	Chicken puree	Vegetable	Kale chopped	-3.6111	11.4303	352	-0.32	0.7522
Group*SampleName	Vegetable	Chicken puree	Vegetable	Kale puree	-2.7222	11.4303	352	-0.24	0.8119
Group*SampleName	Vegetable	Chicken puree	Vegetable	Oat puree	-14.8333	11.4303	352	-1.30	0.1952
Group*SampleName	Vegetable	Chicken puree	Vegetable	Spinach chopped	-11.2778	11.4303	352	-0.99	0.3245

Differences of Least Squares Means									
Effect	Group	SampleName	_Group	_SampleName	Estimate	Standard Error	DF	t Value	Pr > |t|
Group*SampleName	Vegetable	Chicken puree	Vegetable	Spinach puree	-5.3333	11.4303	352	-0.47	0.6411
Group*SampleName	Vegetable	Chicken puree	Control	Asparagus chopped	-29.3499	13.7559	211	-2.13	0.0340
Group*SampleName	Vegetable	Chicken puree	Control	Asparagus puree	-34.1624	13.7559	211	-2.48	0.0138
Group*SampleName	Vegetable	Chicken puree	Control	Beef puree	-12.4749	13.7559	211	-0.91	0.3655
Group*SampleName	Vegetable	Chicken puree	Control	Black bean puree	-28.2249	13.7559	211	-2.05	0.0414
Group*SampleName	Vegetable	Chicken puree	Control	Broccoli chopped	-41.0999	13.7559	211	-2.99	0.0031
Group*SampleName	Vegetable	Chicken puree	Control	Broccoli puree	-20.7249	13.7559	211	-1.51	0.1334
Group*SampleName	Vegetable	Chicken puree	Control	Chicken puree	-16.0374	13.7559	211	-1.17	0.2450
Group*SampleName	Vegetable	Chicken puree	Control	Kale chopped	4.0251	13.7559	211	0.29	0.7701
Group*SampleName	Vegetable	Chicken puree	Control	Kale puree	3.2126	13.7559	211	0.23	0.8156
Group*SampleName	Vegetable	Chicken puree	Control	Oat puree	-3.6624	13.7559	211	-0.27	0.7903
Group*SampleName	Vegetable	Chicken puree	Control	Spinach chopped	-8.2249	13.7559	211	-0.60	0.5505
Group*SampleName	Vegetable	Chicken puree	Control	Spinach puree	-6.0999	13.7559	211	-0.44	0.6579
Group*SampleName	Vegetable	Kale chopped	Vegetable	Kale puree	0.8889	11.4303	352	0.08	0.9381
Group*SampleName	Vegetable	Kale chopped	Vegetable	Oat puree	-11.2222	11.4303	352	-0.98	0.3269
Group*SampleName	Vegetable	Kale chopped	Vegetable	Spinach chopped	-7.6667	11.4303	352	-0.67	0.5028
Group*SampleName	Vegetable	Kale chopped	Vegetable	Spinach puree	-1.7222	11.4303	352	-0.15	0.8803
Group*SampleName	Vegetable	Kale chopped	Control	Asparagus chopped	-25.7388	13.7559	211	-1.87	0.0627
Group*SampleName	Vegetable	Kale chopped	Control	Asparagus puree	-30.5513	13.7559	211	-2.22	0.0274
Group*SampleName	Vegetable	Kale chopped	Control	Beef puree	-8.8638	13.7559	211	-0.64	0.5200
Group*SampleName	Vegetable	Kale chopped	Control	Black bean puree	-24.6138	13.7559	211	-1.79	0.0750
Group*SampleName	Vegetable	Kale chopped	Control	Broccoli chopped	-37.4888	13.7559	211	-2.73	0.0070
Group*SampleName	Vegetable	Kale chopped	Control	Broccoli puree	-17.1138	13.7559	211	-1.24	0.2148
Group*SampleName	Vegetable	Kale chopped	Control	Chicken puree	-12.4263	13.7559	211	-0.90	0.3674
Group*SampleName	Vegetable	Kale chopped	Control	Kale chopped	7.6362	13.7559	211	0.56	0.5794
Group*SampleName	Vegetable	Kale chopped	Control	Kale puree	6.8237	13.7559	211	0.50	0.6204
Group*SampleName	Vegetable	Kale chopped	Control	Oat puree	-0.05126	13.7559	211	-0.00	0.9970
Group*SampleName	Vegetable	Kale chopped	Control	Spinach chopped	-4.6138	13.7559	211	-0.34	0.7377
Group*SampleName	Vegetable	Kale chopped	Control	Spinach puree	-2.4888	13.7559	211	-0.18	0.8566
Group*SampleName	Vegetable	Kale puree	Vegetable	Oat puree	-12.1111	11.4303	352	-1.06	0.2901
Group*SampleName	Vegetable	Kale puree	Vegetable	Spinach chopped	-8.5556	11.4303	352	-0.75	0.4547
Group*SampleName	Vegetable	Kale puree	Vegetable	Spinach puree	-2.6111	11.4303	352	-0.23	0.8194
Group*SampleName	Vegetable	Kale puree	Control	Asparagus chopped	-26.6276	13.7559	211	-1.94	0.0542
Group*SampleName	Vegetable	Kale puree	Control	Asparagus puree	-31.4401	13.7559	211	-2.29	0.0233
Group*SampleName	Vegetable	Kale puree	Control	Beef puree	-9.7526	13.7559	211	-0.71	0.4791
Group*SampleName	Vegetable	Kale puree	Control	Black bean puree	-25.5026	13.7559	211	-1.85	0.0651
Group*SampleName	Vegetable	Kale puree	Control	Broccoli chopped	-38.3776	13.7559	211	-2.79	0.0058
Group*SampleName	Vegetable	Kale puree	Control	Broccoli puree	-18.0026	13.7559	211	-1.31	0.1921
Group*SampleName	Vegetable	Kale puree	Control	Chicken puree	-13.3151	13.7559	211	-0.97	0.3342
Group*SampleName	Vegetable	Kale puree	Control	Kale chopped	6.7474	13.7559	211	0.49	0.6243
Group*SampleName	Vegetable	Kale puree	Control	Kale puree	5.9349	13.7559	211	0.43	0.6666
Group*SampleName	Vegetable	Kale puree	Control	Oat puree	-0.9401	13.7559	211	-0.07	0.9456

Differences of Least Squares Means									
Effect	Group	SampleName	_Group	_SampleName	Estimate	Standard Error	DF	t Value	Pr > |t|
Group*SampleName	Vegetable	Kale puree	Control	Spinach chopped	-5.5026	13.7559	211	-0.40	0.6895
Group*SampleName	Vegetable	Kale puree	Control	Spinach puree	-3.3776	13.7559	211	-0.25	0.8063
Group*SampleName	Vegetable	Oat puree	Vegetable	Spinach chopped	3.5556	11.4303	352	0.31	0.7559
Group*SampleName	Vegetable	Oat puree	Vegetable	Spinach puree	9.5000	11.4303	352	0.83	0.4065
Group*SampleName	Vegetable	Oat puree	Control	Asparagus chopped	-14.5165	13.7559	211	-1.06	0.2925
Group*SampleName	Vegetable	Oat puree	Control	Asparagus puree	-19.3290	13.7559	211	-1.41	0.1615
Group*SampleName	Vegetable	Oat puree	Control	Beef puree	2.3585	13.7559	211	0.17	0.8640
Group*SampleName	Vegetable	Oat puree	Control	Black bean puree	-13.3915	13.7559	211	-0.97	0.3314
Group*SampleName	Vegetable	Oat puree	Control	Broccoli chopped	-26.2665	13.7559	211	-1.91	0.0576
Group*SampleName	Vegetable	Oat puree	Control	Broccoli puree	-5.8915	13.7559	211	-0.43	0.6689
Group*SampleName	Vegetable	Oat puree	Control	Chicken puree	-1.2040	13.7559	211	-0.09	0.9303
Group*SampleName	Vegetable	Oat puree	Control	Kale chopped	18.8585	13.7559	211	1.37	0.1719
Group*SampleName	Vegetable	Oat puree	Control	Kale puree	18.0460	13.7559	211	1.31	0.1910
Group*SampleName	Vegetable	Oat puree	Control	Oat puree	11.1710	13.7559	211	0.81	0.4177
Group*SampleName	Vegetable	Oat puree	Control	Spinach chopped	6.6085	13.7559	211	0.48	0.6314
Group*SampleName	Vegetable	Oat puree	Control	Spinach puree	8.7335	13.7559	211	0.63	0.5262
Group*SampleName	Vegetable	Spinach chopped	Vegetable	Spinach puree	5.9444	11.4303	352	0.52	0.6033
Group*SampleName	Vegetable	Spinach chopped	Control	Asparagus chopped	-18.0721	13.7559	211	-1.31	0.1904
Group*SampleName	Vegetable	Spinach chopped	Control	Asparagus puree	-22.8846	13.7559	211	-1.66	0.0977
Group*SampleName	Vegetable	Spinach chopped	Control	Beef puree	-1.1971	13.7559	211	-0.09	0.9307
Group*SampleName	Vegetable	Spinach chopped	Control	Black bean puree	-16.9471	13.7559	211	-1.23	0.2193
Group*SampleName	Vegetable	Spinach chopped	Control	Broccoli chopped	-29.8221	13.7559	211	-2.17	0.0313
Group*SampleName	Vegetable	Spinach chopped	Control	Broccoli puree	-9.4471	13.7559	211	-0.69	0.4930
Group*SampleName	Vegetable	Spinach chopped	Control	Chicken puree	-4.7596	13.7559	211	-0.35	0.7297
Group*SampleName	Vegetable	Spinach chopped	Control	Kale chopped	15.3029	13.7559	211	1.11	0.2672
Group*SampleName	Vegetable	Spinach chopped	Control	Kale puree	14.4904	13.7559	211	1.05	0.2934
Group*SampleName	Vegetable	Spinach chopped	Control	Oat puree	7.6154	13.7559	211	0.55	0.5804
Group*SampleName	Vegetable	Spinach chopped	Control	Spinach chopped	3.0529	13.7559	211	0.22	0.8246
Group*SampleName	Vegetable	Spinach chopped	Control	Spinach puree	5.1779	13.7559	211	0.38	0.7070
Group*SampleName	Vegetable	Spinach puree	Control	Asparagus chopped	-24.0165	13.7559	211	-1.75	0.0823
Group*SampleName	Vegetable	Spinach puree	Control	Asparagus puree	-28.8290	13.7559	211	-2.10	0.0373
Group*SampleName	Vegetable	Spinach puree	Control	Beef puree	-7.1415	13.7559	211	-0.52	0.6042
Group*SampleName	Vegetable	Spinach puree	Control	Black bean puree	-22.8915	13.7559	211	-1.66	0.0976
Group*SampleName	Vegetable	Spinach puree	Control	Broccoli chopped	-35.7665	13.7559	211	-2.60	0.0100
Group*SampleName	Vegetable	Spinach puree	Control	Broccoli puree	-15.3915	13.7559	211	-1.12	0.2645
Group*SampleName	Vegetable	Spinach puree	Control	Chicken puree	-10.7040	13.7559	211	-0.78	0.4374
Group*SampleName	Vegetable	Spinach puree	Control	Kale chopped	9.3585	13.7559	211	0.68	0.4970
Group*SampleName	Vegetable	Spinach puree	Control	Kale puree	8.5460	13.7559	211	0.62	0.5351
Group*SampleName	Vegetable	Spinach puree	Control	Oat puree	1.6710	13.7559	211	0.12	0.9034
Group*SampleName	Vegetable	Spinach puree	Control	Spinach chopped	-2.8915	13.7559	211	-0.21	0.8337
Group*SampleName	Vegetable	Spinach puree	Control	Spinach puree	-0.7665	13.7559	211	-0.06	0.9556
Group*SampleName	Control	Asparagus chopped	Control	Asparagus puree	-4.8125	12.1236	352	-0.40	0.6916

Differences of Least Squares Means									
Effect	Group	SampleName	_Group	_SampleName	Estimate	Standard Error	DF	t Value	Pr > |t|
Group*SampleName	Control	Asparagus chopped	Control	Beef puree	16.8750	12.1236	352	1.39	0.1648
Group*SampleName	Control	Asparagus chopped	Control	Black bean puree	1.1250	12.1236	352	0.09	0.9261
Group*SampleName	Control	Asparagus chopped	Control	Broccoli chopped	-11.7500	12.1236	352	-0.97	0.3331
Group*SampleName	Control	Asparagus chopped	Control	Broccoli puree	8.6250	12.1236	352	0.71	0.4773
Group*SampleName	Control	Asparagus chopped	Control	Chicken puree	13.3125	12.1236	352	1.10	0.2729
Group*SampleName	Control	Asparagus chopped	Control	Kale chopped	33.3750	12.1236	352	2.75	0.0062
Group*SampleName	Control	Asparagus chopped	Control	Kale puree	32.5625	12.1236	352	2.69	0.0076
Group*SampleName	Control	Asparagus chopped	Control	Oat puree	25.6875	12.1236	352	2.12	0.0348
Group*SampleName	Control	Asparagus chopped	Control	Spinach chopped	21.1250	12.1236	352	1.74	0.0823
Group*SampleName	Control	Asparagus chopped	Control	Spinach puree	23.2500	12.1236	352	1.92	0.0560
Group*SampleName	Control	Asparagus puree	Control	Beef puree	21.6875	12.1236	352	1.79	0.0745
Group*SampleName	Control	Asparagus puree	Control	Black bean puree	5.9375	12.1236	352	0.49	0.6246
Group*SampleName	Control	Asparagus puree	Control	Broccoli chopped	-6.9375	12.1236	352	-0.57	0.5675
Group*SampleName	Control	Asparagus puree	Control	Broccoli puree	13.4375	12.1236	352	1.11	0.2685
Group*SampleName	Control	Asparagus puree	Control	Chicken puree	18.1250	12.1236	352	1.50	0.1358
Group*SampleName	Control	Asparagus puree	Control	Kale chopped	38.1875	12.1236	352	3.15	0.0018
Group*SampleName	Control	Asparagus puree	Control	Kale puree	37.3750	12.1236	352	3.08	0.0022
Group*SampleName	Control	Asparagus puree	Control	Oat puree	30.5000	12.1236	352	2.52	0.0123
Group*SampleName	Control	Asparagus puree	Control	Spinach chopped	25.9375	12.1236	352	2.14	0.0331
Group*SampleName	Control	Asparagus puree	Control	Spinach puree	28.0625	12.1236	352	2.31	0.0212
Group*SampleName	Control	Beef puree	Control	Black bean puree	-15.7500	12.1236	352	-1.30	0.1948
Group*SampleName	Control	Beef puree	Control	Broccoli chopped	-28.6250	12.1236	352	-2.36	0.0188
Group*SampleName	Control	Beef puree	Control	Broccoli puree	-8.2500	12.1236	352	-0.68	0.4966
Group*SampleName	Control	Beef puree	Control	Chicken puree	-3.5625	12.1236	352	-0.29	0.7690
Group*SampleName	Control	Beef puree	Control	Kale chopped	16.5000	12.1236	352	1.36	0.1744
Group*SampleName	Control	Beef puree	Control	Kale puree	15.6875	12.1236	352	1.29	0.1965
Group*SampleName	Control	Beef puree	Control	Oat puree	8.8125	12.1236	352	0.73	0.4678
Group*SampleName	Control	Beef puree	Control	Spinach chopped	4.2500	12.1236	352	0.35	0.7261
Group*SampleName	Control	Beef puree	Control	Spinach puree	6.3750	12.1236	352	0.53	0.5993
Group*SampleName	Control	Black bean puree	Control	Broccoli chopped	-12.8750	12.1236	352	-1.06	0.2890
Group*SampleName	Control	Black bean puree	Control	Broccoli puree	7.5000	12.1236	352	0.62	0.5366
Group*SampleName	Control	Black bean puree	Control	Chicken puree	12.1875	12.1236	352	1.01	0.3155
Group*SampleName	Control	Black bean puree	Control	Kale chopped	32.2500	12.1236	352	2.66	0.0082
Group*SampleName	Control	Black bean puree	Control	Kale puree	31.4375	12.1236	352	2.59	0.0099
Group*SampleName	Control	Black bean puree	Control	Oat puree	24.5625	12.1236	352	2.03	0.0435
Group*SampleName	Control	Black bean puree	Control	Spinach chopped	20.0000	12.1236	352	1.65	0.0999
Group*SampleName	Control	Black bean puree	Control	Spinach puree	22.1250	12.1236	352	1.82	0.0689
Group*SampleName	Control	Broccoli chopped	Control	Broccoli puree	20.3750	12.1236	352	1.68	0.0937
Group*SampleName	Control	Broccoli chopped	Control	Chicken puree	25.0625	12.1236	352	2.07	0.0394
Group*SampleName	Control	Broccoli chopped	Control	Kale chopped	45.1250	12.1236	352	3.72	0.0002
Group*SampleName	Control	Broccoli chopped	Control	Kale puree	44.3125	12.1236	352	3.66	0.0003
Group*SampleName	Control	Broccoli chopped	Control	Oat puree	37.4375	12.1236	352	3.09	0.0022

Differences of Least Squares Means									
Effect	Group	SampleName	_Group	_SampleName	Estimate	Standard Error	DF	t Value	Pr > |t|
Group*SampleName	Control	Broccoli chopped	Control	Spinach chopped	32.8750	12.1236	352	2.71	0.0070
Group*SampleName	Control	Broccoli chopped	Control	Spinach puree	35.0000	12.1236	352	2.89	0.0041
Group*SampleName	Control	Broccoli puree	Control	Chicken puree	4.6875	12.1236	352	0.39	0.6993
Group*SampleName	Control	Broccoli puree	Control	Kale chopped	24.7500	12.1236	352	2.04	0.0419
Group*SampleName	Control	Broccoli puree	Control	Kale puree	23.9375	12.1236	352	1.97	0.0491
Group*SampleName	Control	Broccoli puree	Control	Oat puree	17.0625	12.1236	352	1.41	0.1602
Group*SampleName	Control	Broccoli puree	Control	Spinach chopped	12.5000	12.1236	352	1.03	0.3032
Group*SampleName	Control	Broccoli puree	Control	Spinach puree	14.6250	12.1236	352	1.21	0.2285
Group*SampleName	Control	Chicken puree	Control	Kale chopped	20.0625	12.1236	352	1.65	0.0989
Group*SampleName	Control	Chicken puree	Control	Kale puree	19.2500	12.1236	352	1.59	0.1132
Group*SampleName	Control	Chicken puree	Control	Oat puree	12.3750	12.1236	352	1.02	0.3081
Group*SampleName	Control	Chicken puree	Control	Spinach chopped	7.8125	12.1236	352	0.64	0.5197
Group*SampleName	Control	Chicken puree	Control	Spinach puree	9.9375	12.1236	352	0.82	0.4130
Group*SampleName	Control	Kale chopped	Control	Kale puree	-0.8125	12.1236	352	-0.07	0.9466
Group*SampleName	Control	Kale chopped	Control	Oat puree	-7.6875	12.1236	352	-0.63	0.5264
Group*SampleName	Control	Kale chopped	Control	Spinach chopped	-12.2500	12.1236	352	-1.01	0.3130
Group*SampleName	Control	Kale chopped	Control	Spinach puree	-10.1250	12.1236	352	-0.84	0.4042
Group*SampleName	Control	Kale puree	Control	Oat puree	-6.8750	12.1236	352	-0.57	0.5710
Group*SampleName	Control	Kale puree	Control	Spinach chopped	-11.4375	12.1236	352	-0.94	0.3461
Group*SampleName	Control	Kale puree	Control	Spinach puree	-9.3125	12.1236	352	-0.77	0.4429
Group*SampleName	Control	Oat puree	Control	Spinach chopped	-4.5625	12.1236	352	-0.38	0.7069
Group*SampleName	Control	Oat puree	Control	Spinach puree	-2.4375	12.1236	352	-0.20	0.8408
Group*SampleName	Control	Spinach chopped	Control	Spinach puree	2.1250	12.1236	352	0.18	0.8610

Secondard analysis: Liking between vegetables

The Mixed Procedure

GameVisit=3

Model Information	
Data Set	WORK.GAMEVISITS
Dependent Variable	Liking
Covariance Structure	Compound Symmetry
Subject Effect	ParticipantID
Estimation Method	REML
Residual Variance Method	Profile
Fixed Effects SE Method	Kenward-Roger
Degrees of Freedom Method	Kenward-Roger

Class Level Information		
Class	Levels	Values
ParticipantID	34	1001 1002 1005 1006 1009 1010 1014 1016 1019 1020 1024 1025 1027 1029 1030 1034 1035 1041 1045 1046 1048

Class Level Information		
Class	Levels	Values
		1049 1050 1051 1056 1061 1063 1064 1065 1066 1068 1069 1074 1078
GameVisit	1	3
Group	2	Vegetable Control
Gender	2	Man Woman
SampleName	12	Asparagus chopped Asparagus puree Beef puree Black bean puree Broccoli chopped Broccoli puree Chicken puree Kale chopped Kale puree Oat puree Spinach chopped Spinach puree

Dimensions	
Covariance Parameters	2
Columns in X	41
Columns in Z	0
Subjects	34
Max Obs per Subject	12

Number of Observations	
Number of Observations Read	408
Number of Observations Used	408
Number of Observations Not Used	0

Iteration History			
Iteration	Evaluations	-2 Res Log Like	Criterion
0	1	4003.57740007	
1	1	3895.88327790	0.00000000

Convergence criteria met.

Covariance Parameter Estimates		
Cov Parm	Subject	Estimate
CS	ParticipantID	626.29
Residual		1071.42

Fit Statistics	
-2 Res Log Likelihood	3895.9
AIC (Smaller is Better)	3899.9
AICC (Smaller is Better)	3899.9
BIC (Smaller is Better)	3902.9

Null Model Likelihood Ratio Test		
DF	Chi-Square	Pr > ChiSq
1	107.69	<.0001

Solution for Fixed Effects								
Effect	Group	Gender	SampleName	Estimate	Standard Error	DF	t Value	Pr > |t|
Intercept				-16.9359	10.9897	117	-1.54	0.1260

Solution for Fixed Effects								
Effect	Group	Gender	SampleName	Estimate	Standard Error	DF	t Value	Pr > |t|
SampleName			Asparagus chopped	28.1875	11.5727	352	2.44	0.0154
SampleName			Asparagus puree	26.0625	11.5727	352	2.25	0.0249
SampleName			Beef puree	14.9375	11.5727	352	1.29	0.1976
SampleName			Black bean puree	23.8750	11.5727	352	2.06	0.0398
SampleName			Broccoli chopped	34.0625	11.5727	352	2.94	0.0035
SampleName			Broccoli puree	26.8750	11.5727	352	2.32	0.0208
SampleName			Chicken puree	14.0000	11.5727	352	1.21	0.2272
SampleName			Kale chopped	-8.3750	11.5727	352	-0.72	0.4697
SampleName			Kale puree	-9.6875	11.5727	352	-0.84	0.4031
SampleName			Oat puree	6.6875	11.5727	352	0.58	0.5637
SampleName			Spinach chopped	10.0000	11.5727	352	0.86	0.3881
SampleName			Spinach puree	0	.	.	.	.
Group	Vegetable			8.6405	14.2428	146	0.61	0.5450
Group	Control			0	.	.	.	.
Group*SampleName	Vegetable		Asparagus chopped	-22.3542	15.9052	352	-1.41	0.1608
Group*SampleName	Vegetable		Asparagus puree	-16.3403	15.9052	352	-1.03	0.3050
Group*SampleName	Vegetable		Beef puree	-23.7153	15.9052	352	-1.49	0.1368
Group*SampleName	Vegetable		Black bean puree	-21.3194	15.9052	352	-1.34	0.1810
Group*SampleName	Vegetable		Broccoli chopped	-15.2847	15.9052	352	-0.96	0.3372
Group*SampleName	Vegetable		Broccoli puree	-2.0972	15.9052	352	-0.13	0.8952
Group*SampleName	Vegetable		Chicken puree	-22.6111	15.9052	352	-1.42	0.1560
Group*SampleName	Vegetable		Kale chopped	3.5417	15.9052	352	0.22	0.8239
Group*SampleName	Vegetable		Kale puree	9.5764	15.9052	352	0.60	0.5475
Group*SampleName	Vegetable		Oat puree	12.8681	15.9052	352	0.81	0.4190
Group*SampleName	Vegetable		Spinach chopped	3.4444	15.9052	352	0.22	0.8287
Group*SampleName	Vegetable		Spinach puree	0	.	.	.	.
Group*SampleName	Control		Asparagus chopped	0	.	.	.	.
Group*SampleName	Control		Asparagus puree	0	.	.	.	.
Group*SampleName	Control		Beef puree	0	.	.	.	.
Group*SampleName	Control		Black bean puree	0	.	.	.	.
Group*SampleName	Control		Broccoli chopped	0	.	.	.	.
Group*SampleName	Control		Broccoli puree	0	.	.	.	.
Group*SampleName	Control		Chicken puree	0	.	.	.	.
Group*SampleName	Control		Kale chopped	0	.	.	.	.
Group*SampleName	Control		Kale puree	0	.	.	.	.
Group*SampleName	Control		Oat puree	0	.	.	.	.
Group*SampleName	Control		Spinach chopped	0	.	.	.	.
Group*SampleName	Control		Spinach puree	0	.	.	.	.
Gender		Man		3.8289	10.2124	31	0.37	0.7103
Gender		Woman		0	.	.	.	.

Type 3 Tests of Fixed Effects				
Effect	Num DF	Den DF	F Value	Pr > F
SampleName	11	352	3.89	<.0001
Group	1	31	0.01	0.9336
Group*SampleName	11	352	1.49	0.1329
Gender	1	31	0.14	0.7103

Least Squares Means							
Effect	Group	SampleName	Estimate	Standard Error	DF	t Value	Pr > |t|
Group	Vegetable		-0.3531	6.9139	31	-0.05	0.9596
Group	Control		-1.1360	6.8083	31	-0.17	0.8686
SampleName		Asparagus chopped	6.3093	7.3713	128	0.86	0.3936
SampleName		Asparagus puree	7.1912	7.3713	128	0.98	0.3311
SampleName		Beef puree	-7.6213	7.3713	128	-1.03	0.3031
SampleName		Black bean puree	2.5142	7.3713	128	0.34	0.7336
SampleName		Broccoli chopped	15.7190	7.3713	128	2.13	0.0349
SampleName		Broccoli puree	15.1253	7.3713	128	2.05	0.0422
SampleName		Chicken puree	-8.0067	7.3713	128	-1.09	0.2794
SampleName		Kale chopped	-17.3053	7.3713	128	-2.35	0.0204
SampleName		Kale puree	-15.6004	7.3713	128	-2.12	0.0363
SampleName		Oat puree	2.4204	7.3713	128	0.33	0.7432
SampleName		Spinach chopped	1.0211	7.3713	128	0.14	0.8900
SampleName		Spinach puree	-10.7011	7.3713	128	-1.45	0.1490
Group*SampleName	Vegetable	Asparagus chopped	-0.5475	10.1175	128	-0.05	0.9569
Group*SampleName	Vegetable	Asparagus puree	3.3414	10.1175	128	0.33	0.7417
Group*SampleName	Vegetable	Beef puree	-15.1586	10.1175	128	-1.50	0.1365
Group*SampleName	Vegetable	Black bean puree	-3.8253	10.1175	128	-0.38	0.7060
Group*SampleName	Vegetable	Broccoli chopped	12.3969	10.1175	128	1.23	0.2227
Group*SampleName	Vegetable	Broccoli puree	18.3969	10.1175	128	1.82	0.0714
Group*SampleName	Vegetable	Chicken puree	-14.9920	10.1175	128	-1.48	0.1409
Group*SampleName	Vegetable	Kale chopped	-11.2142	10.1175	128	-1.11	0.2698
Group*SampleName	Vegetable	Kale puree	-6.4920	10.1175	128	-0.64	0.5222
Group*SampleName	Vegetable	Oat puree	13.1747	10.1175	128	1.30	0.1952
Group*SampleName	Vegetable	Spinach chopped	7.0636	10.1175	128	0.70	0.4864
Group*SampleName	Vegetable	Spinach puree	-6.3808	10.1175	128	-0.63	0.5294
Group*SampleName	Control	Asparagus chopped	13.1661	10.3796	145	1.27	0.2067
Group*SampleName	Control	Asparagus puree	11.0411	10.3796	145	1.06	0.2892
Group*SampleName	Control	Beef puree	-0.08388	10.3796	145	-0.01	0.9936
Group*SampleName	Control	Black bean puree	8.8536	10.3796	145	0.85	0.3951
Group*SampleName	Control	Broccoli chopped	19.0411	10.3796	145	1.83	0.0686
Group*SampleName	Control	Broccoli puree	11.8536	10.3796	145	1.14	0.2553
Group*SampleName	Control	Chicken puree	-1.0214	10.3796	145	-0.10	0.9217
Group*SampleName	Control	Kale chopped	-23.3964	10.3796	145	-2.25	0.0257
Group*SampleName	Control	Kale puree	-24.7089	10.3796	145	-2.38	0.0186

Least Squares Means							
Effect	Group	SampleName	Estimate	Standard Error	DF	t Value	Pr > |t|
Group*SampleName	Control	Oat puree	-8.3339	10.3796	145	-0.80	0.4233
Group*SampleName	Control	Spinach chopped	-5.0214	10.3796	145	-0.48	0.6293
Group*SampleName	Control	Spinach puree	-15.0214	10.3796	145	-1.45	0.1500

Differences of Least Squares Means									
Effect	Group	SampleName	_Group	_SampleName	Estimate	Standard Error	DF	t Value	Pr > |t|
Group	Vegetable		Control		0.7829	9.3226	31	0.08	0.9336
SampleName		Asparagus chopped		Asparagus puree	-0.8819	7.9526	352	-0.11	0.9118
SampleName		Asparagus chopped		Beef puree	13.9306	7.9526	352	1.75	0.0807
SampleName		Asparagus chopped		Black bean puree	3.7951	7.9526	352	0.48	0.6335
SampleName		Asparagus chopped		Broccoli chopped	-9.4097	7.9526	352	-1.18	0.2375
SampleName		Asparagus chopped		Broccoli puree	-8.8160	7.9526	352	-1.11	0.2684
SampleName		Asparagus chopped		Chicken puree	14.3160	7.9526	352	1.80	0.0727
SampleName		Asparagus chopped		Kale chopped	23.6146	7.9526	352	2.97	0.0032
SampleName		Asparagus chopped		Kale puree	21.9097	7.9526	352	2.76	0.0062
SampleName		Asparagus chopped		Oat puree	3.8889	7.9526	352	0.49	0.6251
SampleName		Asparagus chopped		Spinach chopped	5.2882	7.9526	352	0.66	0.5065
SampleName		Asparagus chopped		Spinach puree	17.0104	7.9526	352	2.14	0.0331
SampleName		Asparagus puree		Beef puree	14.8125	7.9526	352	1.86	0.0634
SampleName		Asparagus puree		Black bean puree	4.6771	7.9526	352	0.59	0.5568
SampleName		Asparagus puree		Broccoli chopped	-8.5278	7.9526	352	-1.07	0.2843
SampleName		Asparagus puree		Broccoli puree	-7.9340	7.9526	352	-1.00	0.3191
SampleName		Asparagus puree		Chicken puree	15.1979	7.9526	352	1.91	0.0568
SampleName		Asparagus puree		Kale chopped	24.4965	7.9526	352	3.08	0.0022
SampleName		Asparagus puree		Kale puree	22.7917	7.9526	352	2.87	0.0044
SampleName		Asparagus puree		Oat puree	4.7708	7.9526	352	0.60	0.5490
SampleName		Asparagus puree		Spinach chopped	6.1701	7.9526	352	0.78	0.4383
SampleName		Asparagus puree		Spinach puree	17.8924	7.9526	352	2.25	0.0251
SampleName		Beef puree		Black bean puree	-10.1354	7.9526	352	-1.27	0.2033
SampleName		Beef puree		Broccoli chopped	-23.3403	7.9526	352	-2.93	0.0036
SampleName		Beef puree		Broccoli puree	-22.7465	7.9526	352	-2.86	0.0045
SampleName		Beef puree		Chicken puree	0.3854	7.9526	352	0.05	0.9614
SampleName		Beef puree		Kale chopped	9.6840	7.9526	352	1.22	0.2241
SampleName		Beef puree		Kale puree	7.9792	7.9526	352	1.00	0.3164
SampleName		Beef puree		Oat puree	-10.0417	7.9526	352	-1.26	0.2075
SampleName		Beef puree		Spinach chopped	-8.6424	7.9526	352	-1.09	0.2779
SampleName		Beef puree		Spinach puree	3.0799	7.9526	352	0.39	0.6988
SampleName		Black bean puree		Broccoli chopped	-13.2049	7.9526	352	-1.66	0.0977
SampleName		Black bean puree		Broccoli puree	-12.6111	7.9526	352	-1.59	0.1137
SampleName		Black bean puree		Chicken puree	10.5208	7.9526	352	1.32	0.1867
SampleName		Black bean puree		Kale chopped	19.8194	7.9526	352	2.49	0.0132
SampleName		Black bean puree		Kale puree	18.1146	7.9526	352	2.28	0.0233

Differences of Least Squares Means									
Effect	Group	SampleName	_Group	_SampleName	Estimate	Standard Error	DF	t Value	Pr > |t|
SampleName		Black bean puree		Oat puree	0.09375	7.9526	352	0.01	0.9906
SampleName		Black bean puree		Spinach chopped	1.4931	7.9526	352	0.19	0.8512
SampleName		Black bean puree		Spinach puree	13.2153	7.9526	352	1.66	0.0975
SampleName		Broccoli chopped		Broccoli puree	0.5937	7.9526	352	0.07	0.9405
SampleName		Broccoli chopped		Chicken puree	23.7257	7.9526	352	2.98	0.0030
SampleName		Broccoli chopped		Kale chopped	33.0243	7.9526	352	4.15	<.0001
SampleName		Broccoli chopped		Kale puree	31.3194	7.9526	352	3.94	<.0001
SampleName		Broccoli chopped		Oat puree	13.2986	7.9526	352	1.67	0.0954
SampleName		Broccoli chopped		Spinach chopped	14.6979	7.9526	352	1.85	0.0654
SampleName		Broccoli chopped		Spinach puree	26.4201	7.9526	352	3.32	0.0010
SampleName		Broccoli puree		Chicken puree	23.1319	7.9526	352	2.91	0.0039
SampleName		Broccoli puree		Kale chopped	32.4306	7.9526	352	4.08	<.0001
SampleName		Broccoli puree		Kale puree	30.7257	7.9526	352	3.86	0.0001
SampleName		Broccoli puree		Oat puree	12.7049	7.9526	352	1.60	0.1110
SampleName		Broccoli puree		Spinach chopped	14.1042	7.9526	352	1.77	0.0770
SampleName		Broccoli puree		Spinach puree	25.8264	7.9526	352	3.25	0.0013
SampleName		Chicken puree		Kale chopped	9.2986	7.9526	352	1.17	0.2431
SampleName		Chicken puree		Kale puree	7.5938	7.9526	352	0.95	0.3403
SampleName		Chicken puree		Oat puree	-10.4271	7.9526	352	-1.31	0.1907
SampleName		Chicken puree		Spinach chopped	-9.0278	7.9526	352	-1.14	0.2571
SampleName		Chicken puree		Spinach puree	2.6944	7.9526	352	0.34	0.7350
SampleName		Kale chopped		Kale puree	-1.7049	7.9526	352	-0.21	0.8304
SampleName		Kale chopped		Oat puree	-19.7257	7.9526	352	-2.48	0.0136
SampleName		Kale chopped		Spinach chopped	-18.3264	7.9526	352	-2.30	0.0218
SampleName		Kale chopped		Spinach puree	-6.6042	7.9526	352	-0.83	0.4069
SampleName		Kale puree		Oat puree	-18.0208	7.9526	352	-2.27	0.0241
SampleName		Kale puree		Spinach chopped	-16.6215	7.9526	352	-2.09	0.0373
SampleName		Kale puree		Spinach puree	-4.8993	7.9526	352	-0.62	0.5382
SampleName		Oat puree		Spinach chopped	1.3993	7.9526	352	0.18	0.8604
SampleName		Oat puree		Spinach puree	13.1215	7.9526	352	1.65	0.0998
SampleName		Spinach chopped		Spinach puree	11.7222	7.9526	352	1.47	0.1414
Group*SampleName	Vegetable	Asparagus chopped	Vegetable	Asparagus puree	-3.8889	10.9108	352	-0.36	0.7217
Group*SampleName	Vegetable	Asparagus chopped	Vegetable	Beef puree	14.6111	10.9108	352	1.34	0.1814
Group*SampleName	Vegetable	Asparagus chopped	Vegetable	Black bean puree	3.2778	10.9108	352	0.30	0.7640
Group*SampleName	Vegetable	Asparagus chopped	Vegetable	Broccoli chopped	-12.9444	10.9108	352	-1.19	0.2363
Group*SampleName	Vegetable	Asparagus chopped	Vegetable	Broccoli puree	-18.9444	10.9108	352	-1.74	0.0834
Group*SampleName	Vegetable	Asparagus chopped	Vegetable	Chicken puree	14.4444	10.9108	352	1.32	0.1864
Group*SampleName	Vegetable	Asparagus chopped	Vegetable	Kale chopped	10.6667	10.9108	352	0.98	0.3289
Group*SampleName	Vegetable	Asparagus chopped	Vegetable	Kale puree	5.9444	10.9108	352	0.54	0.5862
Group*SampleName	Vegetable	Asparagus chopped	Vegetable	Oat puree	-13.7222	10.9108	352	-1.26	0.2093
Group*SampleName	Vegetable	Asparagus chopped	Vegetable	Spinach chopped	-7.6111	10.9108	352	-0.70	0.4859
Group*SampleName	Vegetable	Asparagus chopped	Vegetable	Spinach puree	5.8333	10.9108	352	0.53	0.5932

Differences of Least Squares Means									
Effect	Group	SampleName	_Group	_SampleName	Estimate	Standard Error	DF	t Value	Pr > |t|
Group*SampleName	Vegetable	Asparagus chopped	Control	Asparagus chopped	-13.7136	14.2428	146	-0.96	0.3372
Group*SampleName	Vegetable	Asparagus chopped	Control	Asparagus puree	-11.5886	14.2428	146	-0.81	0.4172
Group*SampleName	Vegetable	Asparagus chopped	Control	Beef puree	-0.4636	14.2428	146	-0.03	0.9741
Group*SampleName	Vegetable	Asparagus chopped	Control	Black bean puree	-9.4011	14.2428	146	-0.66	0.5103
Group*SampleName	Vegetable	Asparagus chopped	Control	Broccoli chopped	-19.5886	14.2428	146	-1.38	0.1711
Group*SampleName	Vegetable	Asparagus chopped	Control	Broccoli puree	-12.4011	14.2428	146	-0.87	0.3854
Group*SampleName	Vegetable	Asparagus chopped	Control	Chicken puree	0.4739	14.2428	146	0.03	0.9735
Group*SampleName	Vegetable	Asparagus chopped	Control	Kale chopped	22.8489	14.2428	146	1.60	0.1108
Group*SampleName	Vegetable	Asparagus chopped	Control	Kale puree	24.1614	14.2428	146	1.70	0.0919
Group*SampleName	Vegetable	Asparagus chopped	Control	Oat puree	7.7864	14.2428	146	0.55	0.5854
Group*SampleName	Vegetable	Asparagus chopped	Control	Spinach chopped	4.4739	14.2428	146	0.31	0.7539
Group*SampleName	Vegetable	Asparagus chopped	Control	Spinach puree	14.4739	14.2428	146	1.02	0.3112
Group*SampleName	Vegetable	Asparagus puree	Vegetable	Beef puree	18.5000	10.9108	352	1.70	0.0909
Group*SampleName	Vegetable	Asparagus puree	Vegetable	Black bean puree	7.1667	10.9108	352	0.66	0.5117
Group*SampleName	Vegetable	Asparagus puree	Vegetable	Broccoli chopped	-9.0556	10.9108	352	-0.83	0.4071
Group*SampleName	Vegetable	Asparagus puree	Vegetable	Broccoli puree	-15.0556	10.9108	352	-1.38	0.1685
Group*SampleName	Vegetable	Asparagus puree	Vegetable	Chicken puree	18.3333	10.9108	352	1.68	0.0938
Group*SampleName	Vegetable	Asparagus puree	Vegetable	Kale chopped	14.5556	10.9108	352	1.33	0.1831
Group*SampleName	Vegetable	Asparagus puree	Vegetable	Kale puree	9.8333	10.9108	352	0.90	0.3681
Group*SampleName	Vegetable	Asparagus puree	Vegetable	Oat puree	-9.8333	10.9108	352	-0.90	0.3681
Group*SampleName	Vegetable	Asparagus puree	Vegetable	Spinach chopped	-3.7222	10.9108	352	-0.34	0.7332
Group*SampleName	Vegetable	Asparagus puree	Vegetable	Spinach puree	9.7222	10.9108	352	0.89	0.3735
Group*SampleName	Vegetable	Asparagus puree	Control	Asparagus chopped	-9.8247	14.2428	146	-0.69	0.4914
Group*SampleName	Vegetable	Asparagus puree	Control	Asparagus puree	-7.6997	14.2428	146	-0.54	0.5896
Group*SampleName	Vegetable	Asparagus puree	Control	Beef puree	3.4253	14.2428	146	0.24	0.8103
Group*SampleName	Vegetable	Asparagus puree	Control	Black bean puree	-5.5122	14.2428	146	-0.39	0.6993
Group*SampleName	Vegetable	Asparagus puree	Control	Broccoli chopped	-15.6997	14.2428	146	-1.10	0.2721
Group*SampleName	Vegetable	Asparagus puree	Control	Broccoli puree	-8.5122	14.2428	146	-0.60	0.5510
Group*SampleName	Vegetable	Asparagus puree	Control	Chicken puree	4.3628	14.2428	146	0.31	0.7598
Group*SampleName	Vegetable	Asparagus puree	Control	Kale chopped	26.7378	14.2428	146	1.88	0.0625
Group*SampleName	Vegetable	Asparagus puree	Control	Kale puree	28.0503	14.2428	146	1.97	0.0508
Group*SampleName	Vegetable	Asparagus puree	Control	Oat puree	11.6753	14.2428	146	0.82	0.4137
Group*SampleName	Vegetable	Asparagus puree	Control	Spinach chopped	8.3628	14.2428	146	0.59	0.5580
Group*SampleName	Vegetable	Asparagus puree	Control	Spinach puree	18.3628	14.2428	146	1.29	0.1993
Group*SampleName	Vegetable	Beef puree	Vegetable	Black bean puree	-11.3333	10.9108	352	-1.04	0.2996
Group*SampleName	Vegetable	Beef puree	Vegetable	Broccoli chopped	-27.5556	10.9108	352	-2.53	0.0120
Group*SampleName	Vegetable	Beef puree	Vegetable	Broccoli puree	-33.5556	10.9108	352	-3.08	0.0023
Group*SampleName	Vegetable	Beef puree	Vegetable	Chicken puree	-0.1667	10.9108	352	-0.02	0.9878
Group*SampleName	Vegetable	Beef puree	Vegetable	Kale chopped	-3.9444	10.9108	352	-0.36	0.7179
Group*SampleName	Vegetable	Beef puree	Vegetable	Kale puree	-8.6667	10.9108	352	-0.79	0.4275
Group*SampleName	Vegetable	Beef puree	Vegetable	Oat puree	-28.3333	10.9108	352	-2.60	0.0098
Group*SampleName	Vegetable	Beef puree	Vegetable	Spinach chopped	-22.2222	10.9108	352	-2.04	0.0424

Differences of Least Squares Means									
Effect	Group	SampleName	_Group	_SampleName	Estimate	Standard Error	DF	t Value	Pr > |t|
Group*SampleName	Vegetable	Beef puree	Vegetable	Spinach puree	-8.7778	10.9108	352	-0.80	0.4217
Group*SampleName	Vegetable	Beef puree	Control	Asparagus chopped	-28.3247	14.2428	146	-1.99	0.0486
Group*SampleName	Vegetable	Beef puree	Control	Asparagus puree	-26.1997	14.2428	146	-1.84	0.0679
Group*SampleName	Vegetable	Beef puree	Control	Beef puree	-15.0747	14.2428	146	-1.06	0.2916
Group*SampleName	Vegetable	Beef puree	Control	Black bean puree	-24.0122	14.2428	146	-1.69	0.0939
Group*SampleName	Vegetable	Beef puree	Control	Broccoli chopped	-34.1997	14.2428	146	-2.40	0.0176
Group*SampleName	Vegetable	Beef puree	Control	Broccoli puree	-27.0122	14.2428	146	-1.90	0.0599
Group*SampleName	Vegetable	Beef puree	Control	Chicken puree	-14.1372	14.2428	146	-0.99	0.3226
Group*SampleName	Vegetable	Beef puree	Control	Kale chopped	8.2378	14.2428	146	0.58	0.5639
Group*SampleName	Vegetable	Beef puree	Control	Kale puree	9.5503	14.2428	146	0.67	0.5036
Group*SampleName	Vegetable	Beef puree	Control	Oat puree	-6.8247	14.2428	146	-0.48	0.6325
Group*SampleName	Vegetable	Beef puree	Control	Spinach chopped	-10.1372	14.2428	146	-0.71	0.4778
Group*SampleName	Vegetable	Beef puree	Control	Spinach puree	-0.1372	14.2428	146	-0.01	0.9923
Group*SampleName	Vegetable	Black bean puree	Vegetable	Broccoli chopped	-16.2222	10.9108	352	-1.49	0.1380
Group*SampleName	Vegetable	Black bean puree	Vegetable	Broccoli puree	-22.2222	10.9108	352	-2.04	0.0424
Group*SampleName	Vegetable	Black bean puree	Vegetable	Chicken puree	11.1667	10.9108	352	1.02	0.3068
Group*SampleName	Vegetable	Black bean puree	Vegetable	Kale chopped	7.3889	10.9108	352	0.68	0.4987
Group*SampleName	Vegetable	Black bean puree	Vegetable	Kale puree	2.6667	10.9108	352	0.24	0.8071
Group*SampleName	Vegetable	Black bean puree	Vegetable	Oat puree	-17.0000	10.9108	352	-1.56	0.1201
Group*SampleName	Vegetable	Black bean puree	Vegetable	Spinach chopped	-10.8889	10.9108	352	-1.00	0.3190
Group*SampleName	Vegetable	Black bean puree	Vegetable	Spinach puree	2.5556	10.9108	352	0.23	0.8149
Group*SampleName	Vegetable	Black bean puree	Control	Asparagus chopped	-16.9914	14.2428	146	-1.19	0.2348
Group*SampleName	Vegetable	Black bean puree	Control	Asparagus puree	-14.8664	14.2428	146	-1.04	0.2983
Group*SampleName	Vegetable	Black bean puree	Control	Beef puree	-3.7414	14.2428	146	-0.26	0.7932
Group*SampleName	Vegetable	Black bean puree	Control	Black bean puree	-12.6789	14.2428	146	-0.89	0.3748
Group*SampleName	Vegetable	Black bean puree	Control	Broccoli chopped	-22.8664	14.2428	146	-1.61	0.1106
Group*SampleName	Vegetable	Black bean puree	Control	Broccoli puree	-15.6789	14.2428	146	-1.10	0.2728
Group*SampleName	Vegetable	Black bean puree	Control	Chicken puree	-2.8039	14.2428	146	-0.20	0.8442
Group*SampleName	Vegetable	Black bean puree	Control	Kale chopped	19.5711	14.2428	146	1.37	0.1715
Group*SampleName	Vegetable	Black bean puree	Control	Kale puree	20.8836	14.2428	146	1.47	0.1447
Group*SampleName	Vegetable	Black bean puree	Control	Oat puree	4.5086	14.2428	146	0.32	0.7520
Group*SampleName	Vegetable	Black bean puree	Control	Spinach chopped	1.1961	14.2428	146	0.08	0.9332
Group*SampleName	Vegetable	Black bean puree	Control	Spinach puree	11.1961	14.2428	146	0.79	0.4331
Group*SampleName	Vegetable	Broccoli chopped	Vegetable	Broccoli puree	-6.0000	10.9108	352	-0.55	0.5827
Group*SampleName	Vegetable	Broccoli chopped	Vegetable	Chicken puree	27.3889	10.9108	352	2.51	0.0125
Group*SampleName	Vegetable	Broccoli chopped	Vegetable	Kale chopped	23.6111	10.9108	352	2.16	0.0311
Group*SampleName	Vegetable	Broccoli chopped	Vegetable	Kale puree	18.8889	10.9108	352	1.73	0.0843
Group*SampleName	Vegetable	Broccoli chopped	Vegetable	Oat puree	-0.7778	10.9108	352	-0.07	0.9432
Group*SampleName	Vegetable	Broccoli chopped	Vegetable	Spinach chopped	5.3333	10.9108	352	0.49	0.6253
Group*SampleName	Vegetable	Broccoli chopped	Vegetable	Spinach puree	18.7778	10.9108	352	1.72	0.0861
Group*SampleName	Vegetable	Broccoli chopped	Control	Asparagus chopped	-0.7692	14.2428	146	-0.05	0.9570
Group*SampleName	Vegetable	Broccoli chopped	Control	Asparagus puree	1.3558	14.2428	146	0.10	0.9243

Differences of Least Squares Means									
Effect	Group	SampleName	_Group	_SampleName	Estimate	Standard Error	DF	t Value	Pr > |t|
Group*SampleName	Vegetable	Broccoli chopped	Control	Beef puree	12.4808	14.2428	146	0.88	0.3823
Group*SampleName	Vegetable	Broccoli chopped	Control	Black bean puree	3.5433	14.2428	146	0.25	0.8039
Group*SampleName	Vegetable	Broccoli chopped	Control	Broccoli chopped	-6.6442	14.2428	146	-0.47	0.6416
Group*SampleName	Vegetable	Broccoli chopped	Control	Broccoli puree	0.5433	14.2428	146	0.04	0.9696
Group*SampleName	Vegetable	Broccoli chopped	Control	Chicken puree	13.4183	14.2428	146	0.94	0.3477
Group*SampleName	Vegetable	Broccoli chopped	Control	Kale chopped	35.7933	14.2428	146	2.51	0.0131
Group*SampleName	Vegetable	Broccoli chopped	Control	Kale puree	37.1058	14.2428	146	2.61	0.0101
Group*SampleName	Vegetable	Broccoli chopped	Control	Oat puree	20.7308	14.2428	146	1.46	0.1477
Group*SampleName	Vegetable	Broccoli chopped	Control	Spinach chopped	17.4183	14.2428	146	1.22	0.2233
Group*SampleName	Vegetable	Broccoli chopped	Control	Spinach puree	27.4183	14.2428	146	1.93	0.0562
Group*SampleName	Vegetable	Broccoli puree	Vegetable	Chicken puree	33.3889	10.9108	352	3.06	0.0024
Group*SampleName	Vegetable	Broccoli puree	Vegetable	Kale chopped	29.6111	10.9108	352	2.71	0.0070
Group*SampleName	Vegetable	Broccoli puree	Vegetable	Kale puree	24.8889	10.9108	352	2.28	0.0231
Group*SampleName	Vegetable	Broccoli puree	Vegetable	Oat puree	5.2222	10.9108	352	0.48	0.6325
Group*SampleName	Vegetable	Broccoli puree	Vegetable	Spinach chopped	11.3333	10.9108	352	1.04	0.2996
Group*SampleName	Vegetable	Broccoli puree	Vegetable	Spinach puree	24.7778	10.9108	352	2.27	0.0238
Group*SampleName	Vegetable	Broccoli puree	Control	Asparagus chopped	5.2308	14.2428	146	0.37	0.7140
Group*SampleName	Vegetable	Broccoli puree	Control	Asparagus puree	7.3558	14.2428	146	0.52	0.6063
Group*SampleName	Vegetable	Broccoli puree	Control	Beef puree	18.4808	14.2428	146	1.30	0.1965
Group*SampleName	Vegetable	Broccoli puree	Control	Black bean puree	9.5433	14.2428	146	0.67	0.5039
Group*SampleName	Vegetable	Broccoli puree	Control	Broccoli chopped	-0.6442	14.2428	146	-0.05	0.9640
Group*SampleName	Vegetable	Broccoli puree	Control	Broccoli puree	6.5433	14.2428	146	0.46	0.6466
Group*SampleName	Vegetable	Broccoli puree	Control	Chicken puree	19.4183	14.2428	146	1.36	0.1749
Group*SampleName	Vegetable	Broccoli puree	Control	Kale chopped	41.7933	14.2428	146	2.93	0.0039
Group*SampleName	Vegetable	Broccoli puree	Control	Kale puree	43.1058	14.2428	146	3.03	0.0029
Group*SampleName	Vegetable	Broccoli puree	Control	Oat puree	26.7308	14.2428	146	1.88	0.0625
Group*SampleName	Vegetable	Broccoli puree	Control	Spinach chopped	23.4183	14.2428	146	1.64	0.1023
Group*SampleName	Vegetable	Broccoli puree	Control	Spinach puree	33.4183	14.2428	146	2.35	0.0203
Group*SampleName	Vegetable	Chicken puree	Vegetable	Kale chopped	-3.7778	10.9108	352	-0.35	0.7294
Group*SampleName	Vegetable	Chicken puree	Vegetable	Kale puree	-8.5000	10.9108	352	-0.78	0.4365
Group*SampleName	Vegetable	Chicken puree	Vegetable	Oat puree	-28.1667	10.9108	352	-2.58	0.0102
Group*SampleName	Vegetable	Chicken puree	Vegetable	Spinach chopped	-22.0556	10.9108	352	-2.02	0.0440
Group*SampleName	Vegetable	Chicken puree	Vegetable	Spinach puree	-8.6111	10.9108	352	-0.79	0.4305
Group*SampleName	Vegetable	Chicken puree	Control	Asparagus chopped	-28.1581	14.2428	146	-1.98	0.0499
Group*SampleName	Vegetable	Chicken puree	Control	Asparagus puree	-26.0331	14.2428	146	-1.83	0.0696
Group*SampleName	Vegetable	Chicken puree	Control	Beef puree	-14.9081	14.2428	146	-1.05	0.2970
Group*SampleName	Vegetable	Chicken puree	Control	Black bean puree	-23.8456	14.2428	146	-1.67	0.0962
Group*SampleName	Vegetable	Chicken puree	Control	Broccoli chopped	-34.0331	14.2428	146	-2.39	0.0181
Group*SampleName	Vegetable	Chicken puree	Control	Broccoli puree	-26.8456	14.2428	146	-1.88	0.0614
Group*SampleName	Vegetable	Chicken puree	Control	Chicken puree	-13.9706	14.2428	146	-0.98	0.3283
Group*SampleName	Vegetable	Chicken puree	Control	Kale chopped	8.4044	14.2428	146	0.59	0.5560
Group*SampleName	Vegetable	Chicken puree	Control	Kale puree	9.7169	14.2428	146	0.68	0.4962

Differences of Least Squares Means									
Effect	Group	SampleName	_Group	_SampleName	Estimate	Standard Error	DF	t Value	Pr > |t|
Group*SampleName	Vegetable	Chicken puree	Control	Oat puree	-6.6581	14.2428	146	-0.47	0.6409
Group*SampleName	Vegetable	Chicken puree	Control	Spinach chopped	-9.9706	14.2428	146	-0.70	0.4850
Group*SampleName	Vegetable	Chicken puree	Control	Spinach puree	0.02942	14.2428	146	0.00	0.9984
Group*SampleName	Vegetable	Kale chopped	Vegetable	Kale puree	-4.7222	10.9108	352	-0.43	0.6654
Group*SampleName	Vegetable	Kale chopped	Vegetable	Oat puree	-24.3889	10.9108	352	-2.24	0.0260
Group*SampleName	Vegetable	Kale chopped	Vegetable	Spinach chopped	-18.2778	10.9108	352	-1.68	0.0948
Group*SampleName	Vegetable	Kale chopped	Vegetable	Spinach puree	-4.8333	10.9108	352	-0.44	0.6580
Group*SampleName	Vegetable	Kale chopped	Control	Asparagus chopped	-24.3803	14.2428	146	-1.71	0.0891
Group*SampleName	Vegetable	Kale chopped	Control	Asparagus puree	-22.2553	14.2428	146	-1.56	0.1203
Group*SampleName	Vegetable	Kale chopped	Control	Beef puree	-11.1303	14.2428	146	-0.78	0.4358
Group*SampleName	Vegetable	Kale chopped	Control	Black bean puree	-20.0678	14.2428	146	-1.41	0.1610
Group*SampleName	Vegetable	Kale chopped	Control	Broccoli chopped	-30.2553	14.2428	146	-2.12	0.0353
Group*SampleName	Vegetable	Kale chopped	Control	Broccoli puree	-23.0678	14.2428	146	-1.62	0.1075
Group*SampleName	Vegetable	Kale chopped	Control	Chicken puree	-10.1928	14.2428	146	-0.72	0.4754
Group*SampleName	Vegetable	Kale chopped	Control	Kale chopped	12.1822	14.2428	146	0.86	0.3938
Group*SampleName	Vegetable	Kale chopped	Control	Kale puree	13.4947	14.2428	146	0.95	0.3450
Group*SampleName	Vegetable	Kale chopped	Control	Oat puree	-2.8803	14.2428	146	-0.20	0.8400
Group*SampleName	Vegetable	Kale chopped	Control	Spinach chopped	-6.1928	14.2428	146	-0.43	0.6643
Group*SampleName	Vegetable	Kale chopped	Control	Spinach puree	3.8072	14.2428	146	0.27	0.7896
Group*SampleName	Vegetable	Kale puree	Vegetable	Oat puree	-19.6667	10.9108	352	-1.80	0.0723
Group*SampleName	Vegetable	Kale puree	Vegetable	Spinach chopped	-13.5556	10.9108	352	-1.24	0.2149
Group*SampleName	Vegetable	Kale puree	Vegetable	Spinach puree	-0.1111	10.9108	352	-0.01	0.9919
Group*SampleName	Vegetable	Kale puree	Control	Asparagus chopped	-19.6581	14.2428	146	-1.38	0.1696
Group*SampleName	Vegetable	Kale puree	Control	Asparagus puree	-17.5331	14.2428	146	-1.23	0.2203
Group*SampleName	Vegetable	Kale puree	Control	Beef puree	-6.4081	14.2428	146	-0.45	0.6534
Group*SampleName	Vegetable	Kale puree	Control	Black bean puree	-15.3456	14.2428	146	-1.08	0.2831
Group*SampleName	Vegetable	Kale puree	Control	Broccoli chopped	-25.5331	14.2428	146	-1.79	0.0751
Group*SampleName	Vegetable	Kale puree	Control	Broccoli puree	-18.3456	14.2428	146	-1.29	0.1998
Group*SampleName	Vegetable	Kale puree	Control	Chicken puree	-5.4706	14.2428	146	-0.38	0.7015
Group*SampleName	Vegetable	Kale puree	Control	Kale chopped	16.9044	14.2428	146	1.19	0.2372
Group*SampleName	Vegetable	Kale puree	Control	Kale puree	18.2169	14.2428	146	1.28	0.2029
Group*SampleName	Vegetable	Kale puree	Control	Oat puree	1.8419	14.2428	146	0.13	0.8973
Group*SampleName	Vegetable	Kale puree	Control	Spinach chopped	-1.4706	14.2428	146	-0.10	0.9179
Group*SampleName	Vegetable	Kale puree	Control	Spinach puree	8.5294	14.2428	146	0.60	0.5502
Group*SampleName	Vegetable	Oat puree	Vegetable	Spinach chopped	6.1111	10.9108	352	0.56	0.5758
Group*SampleName	Vegetable	Oat puree	Vegetable	Spinach puree	19.5556	10.9108	352	1.79	0.0739
Group*SampleName	Vegetable	Oat puree	Control	Asparagus chopped	0.008589	14.2428	146	0.00	0.9995
Group*SampleName	Vegetable	Oat puree	Control	Asparagus puree	2.1336	14.2428	146	0.15	0.8811
Group*SampleName	Vegetable	Oat puree	Control	Beef puree	13.2586	14.2428	146	0.93	0.3534
Group*SampleName	Vegetable	Oat puree	Control	Black bean puree	4.3211	14.2428	146	0.30	0.7620
Group*SampleName	Vegetable	Oat puree	Control	Broccoli chopped	-5.8664	14.2428	146	-0.41	0.6810
Group*SampleName	Vegetable	Oat puree	Control	Broccoli puree	1.3211	14.2428	146	0.09	0.9262

Differences of Least Squares Means									
Effect	Group	SampleName	_Group	_SampleName	Estimate	Standard Error	DF	t Value	Pr > |t|
Group*SampleName	Vegetable	Oat puree	Control	Chicken puree	14.1961	14.2428	146	1.00	0.3206
Group*SampleName	Vegetable	Oat puree	Control	Kale chopped	36.5711	14.2428	146	2.57	0.0112
Group*SampleName	Vegetable	Oat puree	Control	Kale puree	37.8836	14.2428	146	2.66	0.0087
Group*SampleName	Vegetable	Oat puree	Control	Oat puree	21.5086	14.2428	146	1.51	0.1332
Group*SampleName	Vegetable	Oat puree	Control	Spinach chopped	18.1961	14.2428	146	1.28	0.2034
Group*SampleName	Vegetable	Oat puree	Control	Spinach puree	28.1961	14.2428	146	1.98	0.0496
Group*SampleName	Vegetable	Spinach chopped	Vegetable	Spinach puree	13.4444	10.9108	352	1.23	0.2187
Group*SampleName	Vegetable	Spinach chopped	Control	Asparagus chopped	-6.1025	14.2428	146	-0.43	0.6689
Group*SampleName	Vegetable	Spinach chopped	Control	Asparagus puree	-3.9775	14.2428	146	-0.28	0.7804
Group*SampleName	Vegetable	Spinach chopped	Control	Beef puree	7.1475	14.2428	146	0.50	0.6165
Group*SampleName	Vegetable	Spinach chopped	Control	Black bean puree	-1.7900	14.2428	146	-0.13	0.9002
Group*SampleName	Vegetable	Spinach chopped	Control	Broccoli chopped	-11.9775	14.2428	146	-0.84	0.4018
Group*SampleName	Vegetable	Spinach chopped	Control	Broccoli puree	-4.7900	14.2428	146	-0.34	0.7371
Group*SampleName	Vegetable	Spinach chopped	Control	Chicken puree	8.0850	14.2428	146	0.57	0.5711
Group*SampleName	Vegetable	Spinach chopped	Control	Kale chopped	30.4600	14.2428	146	2.14	0.0341
Group*SampleName	Vegetable	Spinach chopped	Control	Kale puree	31.7725	14.2428	146	2.23	0.0272
Group*SampleName	Vegetable	Spinach chopped	Control	Oat puree	15.3975	14.2428	146	1.08	0.2814
Group*SampleName	Vegetable	Spinach chopped	Control	Spinach chopped	12.0850	14.2428	146	0.85	0.3976
Group*SampleName	Vegetable	Spinach chopped	Control	Spinach puree	22.0850	14.2428	146	1.55	0.1232
Group*SampleName	Vegetable	Spinach puree	Control	Asparagus chopped	-19.5470	14.2428	146	-1.37	0.1720
Group*SampleName	Vegetable	Spinach puree	Control	Asparagus puree	-17.4220	14.2428	146	-1.22	0.2232
Group*SampleName	Vegetable	Spinach puree	Control	Beef puree	-6.2970	14.2428	146	-0.44	0.6591
Group*SampleName	Vegetable	Spinach puree	Control	Black bean puree	-15.2345	14.2428	146	-1.07	0.2866
Group*SampleName	Vegetable	Spinach puree	Control	Broccoli chopped	-25.4220	14.2428	146	-1.78	0.0764
Group*SampleName	Vegetable	Spinach puree	Control	Broccoli puree	-18.2345	14.2428	146	-1.28	0.2025
Group*SampleName	Vegetable	Spinach puree	Control	Chicken puree	-5.3595	14.2428	146	-0.38	0.7072
Group*SampleName	Vegetable	Spinach puree	Control	Kale chopped	17.0155	14.2428	146	1.19	0.2342
Group*SampleName	Vegetable	Spinach puree	Control	Kale puree	18.3280	14.2428	146	1.29	0.2002
Group*SampleName	Vegetable	Spinach puree	Control	Oat puree	1.9530	14.2428	146	0.14	0.8911
Group*SampleName	Vegetable	Spinach puree	Control	Spinach chopped	-1.3595	14.2428	146	-0.10	0.9241
Group*SampleName	Vegetable	Spinach puree	Control	Spinach puree	8.6405	14.2428	146	0.61	0.5450
Group*SampleName	Control	Asparagus chopped	Control	Asparagus puree	2.1250	11.5727	352	0.18	0.8544
Group*SampleName	Control	Asparagus chopped	Control	Beef puree	13.2500	11.5727	352	1.14	0.2530
Group*SampleName	Control	Asparagus chopped	Control	Black bean puree	4.3125	11.5727	352	0.37	0.7096
Group*SampleName	Control	Asparagus chopped	Control	Broccoli chopped	-5.8750	11.5727	352	-0.51	0.6120
Group*SampleName	Control	Asparagus chopped	Control	Broccoli puree	1.3125	11.5727	352	0.11	0.9098
Group*SampleName	Control	Asparagus chopped	Control	Chicken puree	14.1875	11.5727	352	1.23	0.2210
Group*SampleName	Control	Asparagus chopped	Control	Kale chopped	36.5625	11.5727	352	3.16	0.0017
Group*SampleName	Control	Asparagus chopped	Control	Kale puree	37.8750	11.5727	352	3.27	0.0012
Group*SampleName	Control	Asparagus chopped	Control	Oat puree	21.5000	11.5727	352	1.86	0.0640
Group*SampleName	Control	Asparagus chopped	Control	Spinach chopped	18.1875	11.5727	352	1.57	0.1169
Group*SampleName	Control	Asparagus chopped	Control	Spinach puree	28.1875	11.5727	352	2.44	0.0154

Differences of Least Squares Means									
Effect	Group	SampleName	_Group	_SampleName	Estimate	Standard Error	DF	t Value	Pr > |t|
Group*SampleName	Control	Asparagus puree	Control	Beef puree	11.1250	11.5727	352	0.96	0.3371
Group*SampleName	Control	Asparagus puree	Control	Black bean puree	2.1875	11.5727	352	0.19	0.8502
Group*SampleName	Control	Asparagus puree	Control	Broccoli chopped	-8.0000	11.5727	352	-0.69	0.4898
Group*SampleName	Control	Asparagus puree	Control	Broccoli puree	-0.8125	11.5727	352	-0.07	0.9441
Group*SampleName	Control	Asparagus puree	Control	Chicken puree	12.0625	11.5727	352	1.04	0.2980
Group*SampleName	Control	Asparagus puree	Control	Kale chopped	34.4375	11.5727	352	2.98	0.0031
Group*SampleName	Control	Asparagus puree	Control	Kale puree	35.7500	11.5727	352	3.09	0.0022
Group*SampleName	Control	Asparagus puree	Control	Oat puree	19.3750	11.5727	352	1.67	0.0950
Group*SampleName	Control	Asparagus puree	Control	Spinach chopped	16.0625	11.5727	352	1.39	0.1660
Group*SampleName	Control	Asparagus puree	Control	Spinach puree	26.0625	11.5727	352	2.25	0.0249
Group*SampleName	Control	Beef puree	Control	Black bean puree	-8.9375	11.5727	352	-0.77	0.4405
Group*SampleName	Control	Beef puree	Control	Broccoli chopped	-19.1250	11.5727	352	-1.65	0.0993
Group*SampleName	Control	Beef puree	Control	Broccoli puree	-11.9375	11.5727	352	-1.03	0.3030
Group*SampleName	Control	Beef puree	Control	Chicken puree	0.9375	11.5727	352	0.08	0.9355
Group*SampleName	Control	Beef puree	Control	Kale chopped	23.3125	11.5727	352	2.01	0.0447
Group*SampleName	Control	Beef puree	Control	Kale puree	24.6250	11.5727	352	2.13	0.0340
Group*SampleName	Control	Beef puree	Control	Oat puree	8.2500	11.5727	352	0.71	0.4764
Group*SampleName	Control	Beef puree	Control	Spinach chopped	4.9375	11.5727	352	0.43	0.6699
Group*SampleName	Control	Beef puree	Control	Spinach puree	14.9375	11.5727	352	1.29	0.1976
Group*SampleName	Control	Black bean puree	Control	Broccoli chopped	-10.1875	11.5727	352	-0.88	0.3793
Group*SampleName	Control	Black bean puree	Control	Broccoli puree	-3.0000	11.5727	352	-0.26	0.7956
Group*SampleName	Control	Black bean puree	Control	Chicken puree	9.8750	11.5727	352	0.85	0.3941
Group*SampleName	Control	Black bean puree	Control	Kale chopped	32.2500	11.5727	352	2.79	0.0056
Group*SampleName	Control	Black bean puree	Control	Kale puree	33.5625	11.5727	352	2.90	0.0040
Group*SampleName	Control	Black bean puree	Control	Oat puree	17.1875	11.5727	352	1.49	0.1384
Group*SampleName	Control	Black bean puree	Control	Spinach chopped	13.8750	11.5727	352	1.20	0.2314
Group*SampleName	Control	Black bean puree	Control	Spinach puree	23.8750	11.5727	352	2.06	0.0398
Group*SampleName	Control	Broccoli chopped	Control	Broccoli puree	7.1875	11.5727	352	0.62	0.5350
Group*SampleName	Control	Broccoli chopped	Control	Chicken puree	20.0625	11.5727	352	1.73	0.0839
Group*SampleName	Control	Broccoli chopped	Control	Kale chopped	42.4375	11.5727	352	3.67	0.0003
Group*SampleName	Control	Broccoli chopped	Control	Kale puree	43.7500	11.5727	352	3.78	0.0002
Group*SampleName	Control	Broccoli chopped	Control	Oat puree	27.3750	11.5727	352	2.37	0.0185
Group*SampleName	Control	Broccoli chopped	Control	Spinach chopped	24.0625	11.5727	352	2.08	0.0383
Group*SampleName	Control	Broccoli chopped	Control	Spinach puree	34.0625	11.5727	352	2.94	0.0035
Group*SampleName	Control	Broccoli puree	Control	Chicken puree	12.8750	11.5727	352	1.11	0.2667
Group*SampleName	Control	Broccoli puree	Control	Kale chopped	35.2500	11.5727	352	3.05	0.0025
Group*SampleName	Control	Broccoli puree	Control	Kale puree	36.5625	11.5727	352	3.16	0.0017
Group*SampleName	Control	Broccoli puree	Control	Oat puree	20.1875	11.5727	352	1.74	0.0820
Group*SampleName	Control	Broccoli puree	Control	Spinach chopped	16.8750	11.5727	352	1.46	0.1457
Group*SampleName	Control	Broccoli puree	Control	Spinach puree	26.8750	11.5727	352	2.32	0.0208
Group*SampleName	Control	Chicken puree	Control	Kale chopped	22.3750	11.5727	352	1.93	0.0540
Group*SampleName	Control	Chicken puree	Control	Kale puree	23.6875	11.5727	352	2.05	0.0414

Differences of Least Squares Means									
Effect	Group	SampleName	_Group	_SampleName	Estimate	Standard Error	DF	t Value	Pr > |t|
Group*SampleName	Control	Chicken puree	Control	Oat puree	7.3125	11.5727	352	0.63	0.5279
Group*SampleName	Control	Chicken puree	Control	Spinach chopped	4.0000	11.5727	352	0.35	0.7298
Group*SampleName	Control	Chicken puree	Control	Spinach puree	14.0000	11.5727	352	1.21	0.2272
Group*SampleName	Control	Kale chopped	Control	Kale puree	1.3125	11.5727	352	0.11	0.9098
Group*SampleName	Control	Kale chopped	Control	Oat puree	-15.0625	11.5727	352	-1.30	0.1939
Group*SampleName	Control	Kale chopped	Control	Spinach chopped	-18.3750	11.5727	352	-1.59	0.1132
Group*SampleName	Control	Kale chopped	Control	Spinach puree	-8.3750	11.5727	352	-0.72	0.4697
Group*SampleName	Control	Kale puree	Control	Oat puree	-16.3750	11.5727	352	-1.41	0.1580
Group*SampleName	Control	Kale puree	Control	Spinach chopped	-19.6875	11.5727	352	-1.70	0.0898
Group*SampleName	Control	Kale puree	Control	Spinach puree	-9.6875	11.5727	352	-0.84	0.4031
Group*SampleName	Control	Oat puree	Control	Spinach chopped	-3.3125	11.5727	352	-0.29	0.7749
Group*SampleName	Control	Oat puree	Control	Spinach puree	6.6875	11.5727	352	0.58	0.5637
Group*SampleName	Control	Spinach chopped	Control	Spinach puree	10.0000	11.5727	352	0.86	0.3881

In [ ]:

In [11]:

```
%%SAS sas

title 'OSF planned analysis: Liking';
proc print data = tests1;
proc print data =likeestim1;
proc print data=means1;
run;

title 'OSF planned analysis: Sweetness';
proc print data = tests2;
proc print data =likeestim2;
proc print data=means2;
run;

title 'OSF planned analysis: Bitterness';
proc print data = tests3;
proc print data =likeestim3;
proc print data=means3;
run;

title 'Secondary analysis: Differences between vegetables, and from 0';
proc print data = tests4;
proc print data=means4;
proc print data = diff4;
run;
```

Out[11]:

OSF planned analysis: Liking

Obs	SampleName	Effect	NumDF	DenDF	FValue	ProbF
1	Asparagus chopped	GameVisit	2	64	0.69	0.5037
2	Asparagus chopped	Group	1	31	1.27	0.2683
3	Asparagus chopped	GameVisit*Group	2	64	2.08	0.1333
4	Asparagus chopped	Gender	1	31	0.14	0.7127
5	Asparagus puree	GameVisit	2	64	0.26	0.7743
6	Asparagus puree	Group	1	31	1.59	0.2167

Obs	SampleName	Effect	NumDF	DenDF	FValue	ProbF
7	Asparagus puree	GameVisit*Group	2	64	0.55	0.5816
8	Asparagus puree	Gender	1	31	0.01	0.9141
9	Beef puree	GameVisit	2	64	2.07	0.1341
10	Beef puree	Group	1	31	0.58	0.4511
11	Beef puree	GameVisit*Group	2	64	0.46	0.6312
12	Beef puree	Gender	1	31	1.55	0.2218
13	Black bean puree	GameVisit	2	64	0.82	0.4468
14	Black bean puree	Group	1	31	1.01	0.3216
15	Black bean puree	GameVisit*Group	2	64	0.14	0.8733
16	Black bean puree	Gender	1	31	0.08	0.7822
17	Broccoli chopped	GameVisit	2	64	0.26	0.7711
18	Broccoli chopped	Group	1	31	0.54	0.4675
19	Broccoli chopped	GameVisit*Group	2	64	0.31	0.7355
20	Broccoli chopped	Gender	1	31	1.55	0.2225
21	Broccoli puree	GameVisit	2	64	0.87	0.4224
22	Broccoli puree	Group	1	31	0.44	0.5134
23	Broccoli puree	GameVisit*Group	2	64	0.08	0.9190
24	Broccoli puree	Gender	1	31	0.20	0.6588
25	Chicken puree	GameVisit	2	64	1.39	0.2556
26	Chicken puree	Group	1	31	0.53	0.4740
27	Chicken puree	GameVisit*Group	2	64	0.08	0.9268
28	Chicken puree	Gender	1	31	1.16	0.2901
29	Kale chopped	GameVisit	2	64	0.66	0.5181
30	Kale chopped	Group	1	31	0.05	0.8194
31	Kale chopped	GameVisit*Group	2	64	4.56	0.0140
32	Kale chopped	Gender	1	31	1.23	0.2759
33	Kale puree	GameVisit	2	64	0.78	0.4645
34	Kale puree	Group	1	31	0.16	0.6895
35	Kale puree	GameVisit*Group	2	64	4.49	0.0150
36	Kale puree	Gender	1	31	0.03	0.8565
37	Oat puree	GameVisit	2	64	2.93	0.0605
38	Oat puree	Group	1	31	2.93	0.0971
39	Oat puree	GameVisit*Group	2	64	0.34	0.7109
40	Oat puree	Gender	1	31	0.17	0.6843
41	Spinach chopped	GameVisit	2	64	1.43	0.2474
42	Spinach chopped	Group	1	31	0.20	0.6598
43	Spinach chopped	GameVisit*Group	2	64	0.59	0.5558
44	Spinach chopped	Gender	1	31	0.03	0.8634
45	Spinach puree	GameVisit	2	64	0.43	0.6510
46	Spinach puree	Group	1	31	0.08	0.7821
47	Spinach puree	GameVisit*Group	2	64	0.56	0.5759
48	Spinach puree	Gender	1	31	0.00	0.9777

OSF planned analysis: Liking

Obs	SampleName	StmtNo	Effect	Label	Estimate	StdErr	DF	tValue	Probt
1	Asparagus chopped	1	GameVisit*Group	Control, GV3-GV1	-4.5625	7.4146	64	-0.62	0.5405
2	Asparagus chopped	2	GameVisit*Group	Control, GV2-GV1	-5.5000	7.4146	64	-0.74	0.4609
3	Asparagus chopped	3	GameVisit*Group	Veg, GV3-GV1	3.0556	6.9905	64	0.44	0.6635
4	Asparagus chopped	4	GameVisit*Group	Veg, GV2-GV1	15.0556	6.9905	64	2.15	0.0350
5	Asparagus chopped	5	GameVisit*Group	Veg - Control, GV1	-22.6483	13.1510	47.03	-1.72	0.0916
6	Asparagus chopped	6	GameVisit*Group	Veg - Control, GV2	-2.0927	13.1510	47.03	-0.16	0.8742
7	Asparagus chopped	7	GameVisit*Group	Veg - Control, GV3	-15.0302	13.1510	47.03	-1.14	0.2589
8	Asparagus puree	1	GameVisit*Group	Control, GV3-GV1	-0.9375	7.9788	64	-0.12	0.9068
9	Asparagus puree	2	GameVisit*Group	Control, GV2-GV1	5.0625	7.9788	64	0.63	0.5280
10	Asparagus puree	3	GameVisit*Group	Veg, GV3-GV1	7.2222	7.5225	64	0.96	0.3406
11	Asparagus puree	4	GameVisit*Group	Veg, GV2-GV1	2.1667	7.5225	64	0.29	0.7743
12	Asparagus puree	5	GameVisit*Group	Veg - Control, GV1	-16.6595	13.4086	49.37	-1.24	0.2199
13	Asparagus puree	6	GameVisit*Group	Veg - Control, GV2	-19.5553	13.4086	49.37	-1.46	0.1511
14	Asparagus puree	7	GameVisit*Group	Veg - Control, GV3	-8.4998	13.4086	49.37	-0.63	0.5291
15	Beef puree	1	GameVisit*Group	Control, GV3-GV1	7.2500	6.5551	64	1.11	0.2729
16	Beef puree	2	GameVisit*Group	Control, GV2-GV1	2.6875	6.5551	64	0.41	0.6832
17	Beef puree	3	GameVisit*Group	Veg, GV3-GV1	7.5556	6.1802	64	1.22	0.2260
18	Beef puree	4	GameVisit*Group	Veg, GV2-GV1	-4.6667	6.1802	64	-0.76	0.4530
19	Beef puree	5	GameVisit*Group	Veg - Control, GV1	-12.0331	19.5509	35.8	-0.62	0.5421
20	Beef puree	6	GameVisit*Group	Veg - Control, GV2	-19.3872	19.5509	35.8	-0.99	0.3280
21	Beef puree	7	GameVisit*Group	Veg - Control, GV3	-11.7275	19.5509	35.8	-0.60	0.5524
22	Black bean puree	1	GameVisit*Group	Control, GV3-GV1	8.6875	9.9642	64	0.87	0.3865
23	Black bean puree	2	GameVisit*Group	Control, GV2-GV1	10.9375	9.9642	64	1.10	0.2765
24	Black bean puree	3	GameVisit*Group	Veg, GV3-GV1	6.6667	9.3943	64	0.71	0.4805
25	Black bean puree	4	GameVisit*Group	Veg, GV2-GV1	4.0000	9.3943	64	0.43	0.6717
26	Black bean puree	5	GameVisit*Group	Veg - Control, GV1	-10.6131	15.6445	52.9	-0.68	0.5005
27	Black bean puree	6	GameVisit*Group	Veg - Control, GV2	-17.5506	15.6445	52.9	-1.12	0.2670
28	Black bean puree	7	GameVisit*Group	Veg - Control, GV3	-12.6339	15.6445	52.9	-0.81	0.4230
29	Broccoli chopped	1	GameVisit*Group	Control, GV3-GV1	3.3125	8.1820	64	0.40	0.6869
30	Broccoli chopped	2	GameVisit*Group	Control, GV2-GV1	8.2500	8.1820	64	1.01	0.3171
31	Broccoli chopped	3	GameVisit*Group	Veg, GV3-GV1	-1.7222	7.7141	64	-0.22	0.8240
32	Broccoli chopped	4	GameVisit*Group	Veg, GV2-GV1	-0.5556	7.7141	64	-0.07	0.9428
33	Broccoli chopped	5	GameVisit*Group	Veg - Control, GV1	-4.8845	14.4514	47.2	-0.34	0.7369
34	Broccoli chopped	6	GameVisit*Group	Veg - Control, GV2	-13.6901	14.4514	47.2	-0.95	0.3483
35	Broccoli chopped	7	GameVisit*Group	Veg - Control, GV3	-9.9193	14.4514	47.2	-0.69	0.4958
36	Broccoli puree	1	GameVisit*Group	Control, GV3-GV1	2.0625	7.2518	64	0.28	0.7770
37	Broccoli puree	2	GameVisit*Group	Control, GV2-GV1	-6.1875	7.2518	64	-0.85	0.3967
38	Broccoli puree	3	GameVisit*Group	Veg, GV3-GV1	0.3333	6.8370	64	0.05	0.9613
39	Broccoli puree	4	GameVisit*Group	Veg, GV2-GV1	-3.8333	6.8370	64	-0.56	0.5770
40	Broccoli puree	5	GameVisit*Group	Veg - Control, GV1	6.8868	12.1769	49.41	0.57	0.5743
41	Broccoli puree	6	GameVisit*Group	Veg - Control, GV2	9.2410	12.1769	49.41	0.76	0.4515
42	Broccoli puree	7	GameVisit*Group	Veg - Control, GV3	5.1576	12.1769	49.41	0.42	0.6737

Obs	SampleName	StmtNo	Effect	Label	Estimate	StdErr	DF	tValue	Probt
43	Chicken puree	1	GameVisit*Group	Control, GV3-GV1	6.5000	8.3313	64	0.78	0.4382
44	Chicken puree	2	GameVisit*Group	Control, GV2-GV1	6.4375	8.3313	64	0.77	0.4426
45	Chicken puree	3	GameVisit*Group	Veg, GV3-GV1	10.9444	7.8548	64	1.39	0.1683
46	Chicken puree	4	GameVisit*Group	Veg, GV2-GV1	9.0556	7.8548	64	1.15	0.2533
47	Chicken puree	5	GameVisit*Group	Veg - Control, GV1	-15.6868	19.5454	39.21	-0.80	0.4271
48	Chicken puree	6	GameVisit*Group	Veg - Control, GV2	-13.0688	19.5454	39.21	-0.67	0.5076
49	Chicken puree	7	GameVisit*Group	Veg - Control, GV3	-11.2424	19.5454	39.21	-0.58	0.5684
50	Kale chopped	1	GameVisit*Group	Control, GV3-GV1	-9.3750	7.5329	64	-1.24	0.2178
51	Kale chopped	2	GameVisit*Group	Control, GV2-GV1	-7.1250	7.5329	64	-0.95	0.3478
52	Kale chopped	3	GameVisit*Group	Veg, GV3-GV1	19.6111	7.1021	64	2.76	0.0075
53	Kale chopped	4	GameVisit*Group	Veg, GV2-GV1	17.5556	7.1021	64	2.47	0.0161
54	Kale chopped	5	GameVisit*Group	Veg - Control, GV1	-15.3106	12.6922	49.25	-1.21	0.2335
55	Kale chopped	6	GameVisit*Group	Veg - Control, GV2	9.3699	12.6922	49.25	0.74	0.4639
56	Kale chopped	7	GameVisit*Group	Veg - Control, GV3	13.6755	12.6922	49.25	1.08	0.2865
57	Kale puree	1	GameVisit*Group	Control, GV3-GV1	-19.1250	7.2240	64	-2.65	0.0102
58	Kale puree	2	GameVisit*Group	Control, GV2-GV1	-14.7500	7.2240	64	-2.04	0.0453
59	Kale puree	3	GameVisit*Group	Veg, GV3-GV1	10.4444	6.8109	64	1.53	0.1301
60	Kale puree	4	GameVisit*Group	Veg, GV2-GV1	2.7778	6.8109	64	0.41	0.6847
61	Kale puree	5	GameVisit*Group	Veg - Control, GV1	-11.6296	11.6043	51.64	-1.00	0.3209
62	Kale puree	6	GameVisit*Group	Veg - Control, GV2	5.8982	11.6043	51.64	0.51	0.6134
63	Kale puree	7	GameVisit*Group	Veg - Control, GV3	17.9399	11.6043	51.64	1.55	0.1282
64	Oat puree	1	GameVisit*Group	Control, GV3-GV1	11.7500	8.8707	64	1.32	0.1900
65	Oat puree	2	GameVisit*Group	Control, GV2-GV1	6.6250	8.8707	64	0.75	0.4579
66	Oat puree	3	GameVisit*Group	Veg, GV3-GV1	16.9444	8.3634	64	2.03	0.0469
67	Oat puree	4	GameVisit*Group	Veg, GV2-GV1	1.7222	8.3634	64	0.21	0.8375
68	Oat puree	5	GameVisit*Group	Veg - Control, GV1	16.3913	11.9348	64.06	1.37	0.1744
69	Oat puree	6	GameVisit*Group	Veg - Control, GV2	11.4885	11.9348	64.06	0.96	0.3394
70	Oat puree	7	GameVisit*Group	Veg - Control, GV3	21.5858	11.9348	64.06	1.81	0.0752
71	Spinach chopped	1	GameVisit*Group	Control, GV3-GV1	1.5000	7.6113	64	0.20	0.8444
72	Spinach chopped	2	GameVisit*Group	Control, GV2-GV1	-2.3750	7.6113	64	-0.31	0.7560
73	Spinach chopped	3	GameVisit*Group	Veg, GV3-GV1	12.1667	7.1760	64	1.70	0.0948
74	Spinach chopped	4	GameVisit*Group	Veg, GV2-GV1	-0.5000	7.1760	64	-0.07	0.9447
75	Spinach chopped	5	GameVisit*Group	Veg - Control, GV1	0.5259	12.1892	51.8	0.04	0.9658
76	Spinach chopped	6	GameVisit*Group	Veg - Control, GV2	2.4009	12.1892	51.8	0.20	0.8446
77	Spinach chopped	7	GameVisit*Group	Veg - Control, GV3	11.1926	12.1892	51.8	0.92	0.3628
78	Spinach puree	1	GameVisit*Group	Control, GV3-GV1	-7.9375	7.1066	64	-1.12	0.2682
79	Spinach puree	2	GameVisit*Group	Control, GV2-GV1	-3.9375	7.1066	64	-0.55	0.5815
80	Spinach puree	3	GameVisit*Group	Veg, GV3-GV1	0.7222	6.7002	64	0.11	0.9145
81	Spinach puree	4	GameVisit*Group	Veg, GV2-GV1	-4.4444	6.7002	64	-0.66	0.5095
82	Spinach puree	5	GameVisit*Group	Veg - Control, GV1	-0.6393	9.3422	66.19	-0.07	0.9456
83	Spinach puree	6	GameVisit*Group	Veg - Control, GV2	-1.1463	9.3422	66.19	-0.12	0.9027
84	Spinach puree	7	GameVisit*Group	Veg - Control, GV3	8.0204	9.3422	66.19	0.86	0.3937

OSF planned analysis: Liking

Obs	SampleName	Effect	GameVisit	Group	Estimate	StdErr	DF	tValue	Probt
1	Asparagus chopped	Group	_	Vegetable	0.04015	8.7226	31	0.00	0.9964
2	Asparagus chopped	Group	_	Control	13.2972	8.5894	31	1.55	0.1317
3	Asparagus chopped	GameVisit	1		5.3273	7.0001	44.7	0.76	0.4506
4	Asparagus chopped	GameVisit	2		10.1050	7.0001	44.7	1.44	0.1558
5	Asparagus chopped	GameVisit	3		4.5738	7.0001	44.7	0.65	0.5168
6	Asparagus chopped	GameVisit*Group	1	Vegetable	-5.9969	9.6111	44.7	-0.62	0.5358
7	Asparagus chopped	GameVisit*Group	1	Control	16.6514	9.5971	46.9	1.74	0.0893
8	Asparagus chopped	GameVisit*Group	2	Vegetable	9.0587	9.6111	44.7	0.94	0.3510
9	Asparagus chopped	GameVisit*Group	2	Control	11.1514	9.5971	46.9	1.16	0.2511
10	Asparagus chopped	GameVisit*Group	3	Vegetable	-2.9413	9.6111	44.7	-0.31	0.7610
11	Asparagus chopped	GameVisit*Group	3	Control	12.0889	9.5971	46.9	1.26	0.2140
12	Asparagus puree	Group	_	Vegetable	-2.2058	8.7658	31	-0.25	0.8030
13	Asparagus puree	Group	_	Control	12.6991	8.6320	31	1.47	0.1513
14	Asparagus puree	GameVisit	1		2.9943	7.1252	46.8	0.42	0.6762
15	Asparagus puree	GameVisit	2		6.6089	7.1252	46.8	0.93	0.3584
16	Asparagus puree	GameVisit	3		6.1367	7.1252	46.8	0.86	0.3935
17	Asparagus puree	GameVisit*Group	1	Vegetable	-5.3354	9.7827	46.7	-0.55	0.5881
18	Asparagus puree	GameVisit*Group	1	Control	11.3241	9.7842	49.2	1.16	0.2527
19	Asparagus puree	GameVisit*Group	2	Vegetable	-3.1688	9.7827	46.7	-0.32	0.7474
20	Asparagus puree	GameVisit*Group	2	Control	16.3866	9.7842	49.2	1.67	0.1003
21	Asparagus puree	GameVisit*Group	3	Vegetable	1.8868	9.7827	46.7	0.19	0.8479
22	Asparagus puree	GameVisit*Group	3	Control	10.3866	9.7842	49.2	1.06	0.2936
23	Beef puree	Group	_	Vegetable	-15.6653	13.9768	31	-1.12	0.2710
24	Beef puree	Group	_	Control	-1.2827	13.7634	31	-0.09	0.9263
25	Beef puree	GameVisit	1		-10.6118	10.5052	35.1	-1.01	0.3193
26	Beef puree	GameVisit	2		-11.6013	10.5052	35.1	-1.10	0.2770
27	Beef puree	GameVisit	3		-3.2090	10.5052	35.1	-0.31	0.7618
28	Beef puree	GameVisit*Group	1	Vegetable	-16.6283	14.4250	35.1	-1.15	0.2568
29	Beef puree	GameVisit*Group	1	Control	-4.5952	14.2742	35.8	-0.32	0.7494
30	Beef puree	GameVisit*Group	2	Vegetable	-21.2949	14.4250	35.1	-1.48	0.1488
31	Beef puree	GameVisit*Group	2	Control	-1.9077	14.2742	35.8	-0.13	0.8944
32	Beef puree	GameVisit*Group	3	Vegetable	-9.0727	14.4250	35.1	-0.63	0.5334
33	Beef puree	GameVisit*Group	3	Control	2.6548	14.2742	35.8	0.19	0.8535
34	Black bean puree	Group	_	Vegetable	-6.8546	10.0115	31	-0.68	0.4986
35	Black bean puree	Group	_	Control	6.7446	9.8587	31	0.68	0.4990
36	Black bean puree	GameVisit	1		-5.1036	8.2934	49.8	-0.62	0.5411
37	Black bean puree	GameVisit	2		2.3651	8.2934	49.8	0.29	0.7767
38	Black bean puree	GameVisit	3		2.5735	8.2934	49.8	0.31	0.7576
39	Black bean puree	GameVisit*Group	1	Vegetable	-10.4101	11.3863	49.8	-0.91	0.3650
40	Black bean puree	GameVisit*Group	1	Control	0.2029	11.4144	52.7	0.02	0.9859
41	Black bean puree	GameVisit*Group	2	Vegetable	-6.4101	11.3863	49.8	-0.56	0.5760
42	Black bean puree	GameVisit*Group	2	Control	11.1404	11.4144	52.7	0.98	0.3335

Obs	SampleName	Effect	GameVisit	Group	Estimate	StdErr	DF	tValue	Probt
43	Black bean puree	GameVisit*Group	3	Vegetable	-3.7435	11.3863	49.8	-0.33	0.7437
44	Black bean puree	GameVisit*Group	3	Control	8.8904	11.4144	52.7	0.78	0.4395
45	Broccoli chopped	Group	_	Vegetable	7.4052	9.5750	31	0.77	0.4452
46	Broccoli chopped	Group	_	Control	16.9032	9.4288	31	1.79	0.0828
47	Broccoli chopped	GameVisit	1		10.6067	7.6913	44.9	1.38	0.1747
48	Broccoli chopped	GameVisit	2		14.4540	7.6913	44.9	1.88	0.0667
49	Broccoli chopped	GameVisit	3		11.4019	7.6913	44.9	1.48	0.1452
50	Broccoli chopped	GameVisit*Group	1	Vegetable	8.1645	10.5601	44.8	0.77	0.4435
51	Broccoli chopped	GameVisit*Group	1	Control	13.0490	10.5460	47.1	1.24	0.2221
52	Broccoli chopped	GameVisit*Group	2	Vegetable	7.6089	10.5601	44.8	0.72	0.4749
53	Broccoli chopped	GameVisit*Group	2	Control	21.2990	10.5460	47.1	2.02	0.0491
54	Broccoli chopped	GameVisit*Group	3	Vegetable	6.4422	10.5601	44.8	0.61	0.5449
55	Broccoli chopped	GameVisit*Group	3	Control	16.3615	10.5460	47.1	1.55	0.1275
56	Broccoli puree	Group	_	Vegetable	14.3775	7.9587	31	1.81	0.0806
57	Broccoli puree	Group	_	Control	7.2824	7.8372	31	0.93	0.3600
58	Broccoli puree	GameVisit	1		12.1008	6.4705	46.8	1.87	0.0677
59	Broccoli puree	GameVisit	2		7.0904	6.4705	46.8	1.10	0.2788
60	Broccoli puree	GameVisit	3		13.2987	6.4705	46.8	2.06	0.0455
61	Broccoli puree	GameVisit*Group	1	Vegetable	15.5442	8.8839	46.8	1.75	0.0867
62	Broccoli puree	GameVisit*Group	1	Control	8.6574	8.8855	49.3	0.97	0.3347
63	Broccoli puree	GameVisit*Group	2	Vegetable	11.7109	8.8839	46.8	1.32	0.1939
64	Broccoli puree	GameVisit*Group	2	Control	2.4699	8.8855	49.3	0.28	0.7822
65	Broccoli puree	GameVisit*Group	3	Vegetable	15.8775	8.8839	46.8	1.79	0.0804
66	Broccoli puree	GameVisit*Group	3	Control	10.7199	8.8855	49.3	1.21	0.2334
67	Chicken puree	Group	_	Vegetable	-14.3094	13.6410	31	-1.05	0.3023
68	Chicken puree	Group	_	Control	-0.9767	13.4327	31	-0.07	0.9425
69	Chicken puree	GameVisit	1		-13.1326	10.4691	38	-1.25	0.2173
70	Chicken puree	GameVisit	2		-5.3861	10.4691	38	-0.51	0.6099
71	Chicken puree	GameVisit	3		-4.4104	10.4691	38	-0.42	0.6759
72	Chicken puree	GameVisit*Group	1	Vegetable	-20.9760	14.3750	38	-1.46	0.1527
73	Chicken puree	GameVisit*Group	1	Control	-5.2892	14.2679	39.1	-0.37	0.7129
74	Chicken puree	GameVisit*Group	2	Vegetable	-11.9205	14.3750	38	-0.83	0.4121
75	Chicken puree	GameVisit*Group	2	Control	1.1483	14.2679	39.1	0.08	0.9363
76	Chicken puree	GameVisit*Group	3	Vegetable	-10.0316	14.3750	38	-0.70	0.4895
77	Chicken puree	GameVisit*Group	3	Control	1.2108	14.2679	39.1	0.08	0.9328
78	Kale chopped	Group	_	Vegetable	-15.7213	8.3036	31	-1.89	0.0677
79	Kale chopped	Group	_	Control	-18.2996	8.1768	31	-2.24	0.0325
80	Kale chopped	GameVisit	1		-20.4549	6.7451	46.6	-3.03	0.0040
81	Kale chopped	GameVisit	2		-15.2396	6.7451	46.6	-2.26	0.0286
82	Kale chopped	GameVisit	3		-15.3368	6.7451	46.6	-2.27	0.0276
83	Kale chopped	GameVisit*Group	1	Vegetable	-28.1102	9.2608	46.6	-3.04	0.0039
84	Kale chopped	GameVisit*Group	1	Control	-12.7996	9.2615	49.1	-1.38	0.1732
85	Kale chopped	GameVisit*Group	2	Vegetable	-10.5547	9.2608	46.6	-1.14	0.2602

Obs	SampleName	Effect	GameVisit	Group	Estimate	StdErr	DF	tValue	Probt
86	Kale chopped	GameVisit*Group	2	Control	-19.9246	9.2615	49.1	-2.15	0.0364
87	Kale chopped	GameVisit*Group	3	Vegetable	-8.4991	9.2608	46.6	-0.92	0.3635
88	Kale chopped	GameVisit*Group	3	Control	-22.1746	9.2615	49.1	-2.39	0.0205
89	Kale puree	Group	_	Vegetable	-13.0327	7.4827	31	-1.74	0.0915
90	Kale puree	Group	_	Control	-17.1022	7.3685	31	-2.32	0.0270
91	Kale puree	GameVisit	1		-11.6253	6.1569	48.7	-1.89	0.0650
92	Kale puree	GameVisit	2		-17.6115	6.1569	48.7	-2.86	0.0062
93	Kale puree	GameVisit	3		-15.9656	6.1569	48.7	-2.59	0.0125
94	Kale puree	GameVisit*Group	1	Vegetable	-17.4401	8.4531	48.7	-2.06	0.0444
95	Kale puree	GameVisit*Group	1	Control	-5.8106	8.4670	51.5	-0.69	0.4956
96	Kale puree	GameVisit*Group	2	Vegetable	-14.6624	8.4531	48.7	-1.73	0.0891
97	Kale puree	GameVisit*Group	2	Control	-20.5606	8.4670	51.5	-2.43	0.0187
98	Kale puree	GameVisit*Group	3	Vegetable	-6.9957	8.4531	48.7	-0.83	0.4119
99	Kale puree	GameVisit*Group	3	Control	-24.9356	8.4670	51.5	-2.95	0.0048
100	Oat puree	Group	_	Vegetable	2.5928	7.1479	31	0.36	0.7193
101	Oat puree	Group	_	Control	-13.8957	7.0388	31	-1.97	0.0573
102	Oat puree	GameVisit	1		-11.8251	6.2834	59.8	-1.88	0.0647
103	Oat puree	GameVisit	2		-7.6515	6.2834	59.8	-1.22	0.2281
104	Oat puree	GameVisit	3		2.5221	6.2834	59.8	0.40	0.6896
105	Oat puree	GameVisit*Group	1	Vegetable	-3.6294	8.6260	59.7	-0.42	0.6754
106	Oat puree	GameVisit*Group	1	Control	-20.0207	8.7048	63.8	-2.30	0.0247
107	Oat puree	GameVisit*Group	2	Vegetable	-1.9072	8.6260	59.7	-0.22	0.8258
108	Oat puree	GameVisit*Group	2	Control	-13.3957	8.7048	63.8	-1.54	0.1288
109	Oat puree	GameVisit*Group	3	Vegetable	13.3150	8.6260	59.7	1.54	0.1280
110	Oat puree	GameVisit*Group	3	Control	-8.2707	8.7048	63.8	-0.95	0.3456
111	Spinach chopped	Group	_	Vegetable	-2.8367	7.8521	31	-0.36	0.7203
112	Spinach chopped	Group	_	Control	-7.5432	7.7322	31	-0.98	0.3368
113	Spinach chopped	GameVisit	1		-6.9886	6.4665	48.9	-1.08	0.2851
114	Spinach chopped	GameVisit	2		-8.4261	6.4665	48.9	-1.30	0.1987
115	Spinach chopped	GameVisit	3		-0.1552	6.4665	48.9	-0.02	0.9809
116	Spinach chopped	GameVisit*Group	1	Vegetable	-6.7256	8.8781	48.8	-0.76	0.4524
117	Spinach chopped	GameVisit*Group	1	Control	-7.2515	8.8937	51.6	-0.82	0.4186
118	Spinach chopped	GameVisit*Group	2	Vegetable	-7.2256	8.8781	48.8	-0.81	0.4197
119	Spinach chopped	GameVisit*Group	2	Control	-9.6265	8.8937	51.6	-1.08	0.2841
120	Spinach chopped	GameVisit*Group	3	Vegetable	5.4411	8.8781	48.8	0.61	0.5428
121	Spinach chopped	GameVisit*Group	3	Control	-5.7515	8.8937	51.6	-0.65	0.5207
122	Spinach puree	Group	_	Vegetable	-9.4713	5.5239	31	-1.71	0.0964
123	Spinach puree	Group	_	Control	-11.5496	5.4395	31	-2.12	0.0418
124	Spinach puree	GameVisit	1		-7.9109	4.9123	61.7	-1.61	0.1124
125	Spinach puree	GameVisit	2		-12.1019	4.9123	61.7	-2.46	0.0166
126	Spinach puree	GameVisit	3		-11.5186	4.9123	61.7	-2.34	0.0223
127	Spinach puree	GameVisit*Group	1	Vegetable	-8.2306	6.7437	61.7	-1.22	0.2269
128	Spinach puree	GameVisit*Group	1	Control	-7.5913	6.8135	66	-1.11	0.2693

Obs	SampleName	Effect	GameVisit	Group	Estimate	StdErr	DF	tValue	Probt
129	Spinach puree	GameVisit*Group	2	Vegetable	-12.6750	6.7437	61.7	-1.88	0.0649
130	Spinach puree	GameVisit*Group	2	Control	-11.5288	6.8135	66	-1.69	0.0954
131	Spinach puree	GameVisit*Group	3	Vegetable	-7.5084	6.7437	61.7	-1.11	0.2699
132	Spinach puree	GameVisit*Group	3	Control	-15.5288	6.8135	66	-2.28	0.0259

OSF planned analysis: Sweetness

Obs	SampleName	Effect	NumDF	DenDF	FValue	ProbF
1	Asparagus chopped	GameVisit	2	64	0.31	0.7363
2	Asparagus chopped	Group	1	31	0.53	0.4703
3	Asparagus chopped	GameVisit*Group	2	64	0.13	0.8751
4	Asparagus chopped	Gender	1	31	0.27	0.6098
5	Asparagus puree	GameVisit	2	64	0.18	0.8367
6	Asparagus puree	Group	1	31	0.30	0.5861
7	Asparagus puree	GameVisit*Group	2	64	2.94	0.0599
8	Asparagus puree	Gender	1	31	0.27	0.6102
9	Beef puree	GameVisit	2	64	2.44	0.0952
10	Beef puree	Group	1	31	0.11	0.7429
11	Beef puree	GameVisit*Group	2	64	0.15	0.8605
12	Beef puree	Gender	1	31	0.00	0.9495
13	Black bean puree	GameVisit	2	64	1.92	0.1544
14	Black bean puree	Group	1	31	0.01	0.9041
15	Black bean puree	GameVisit*Group	2	64	1.12	0.3341
16	Black bean puree	Gender	1	31	0.00	0.9651
17	Broccoli chopped	GameVisit	2	64	0.20	0.8227
18	Broccoli chopped	Group	1	31	0.04	0.8428
19	Broccoli chopped	GameVisit*Group	2	64	0.83	0.4406
20	Broccoli chopped	Gender	1	31	3.72	0.0631
21	Broccoli puree	GameVisit	2	64	0.06	0.9431
22	Broccoli puree	Group	1	31	0.58	0.4503
23	Broccoli puree	GameVisit*Group	2	64	0.17	0.8464
24	Broccoli puree	Gender	1	31	0.42	0.5213
25	Chicken puree	GameVisit	2	64	0.19	0.8292
26	Chicken puree	Group	1	31	0.08	0.7852
27	Chicken puree	GameVisit*Group	2	64	1.25	0.2946
28	Chicken puree	Gender	1	31	0.01	0.9422
29	Kale chopped	GameVisit	2	64	0.29	0.7487
30	Kale chopped	Group	1	31	0.02	0.8768
31	Kale chopped	GameVisit*Group	2	64	2.09	0.1316
32	Kale chopped	Gender	1	31	0.00	0.9769
33	Kale puree	GameVisit	2	64	0.04	0.9635
34	Kale puree	Group	1	31	0.18	0.6701
35	Kale puree	GameVisit*Group	2	64	0.45	0.6386

Obs	SampleName	Effect	NumDF	DenDF	FValue	ProbF
36	Kale puree	Gender	1	31	0.36	0.5552
37	Oat puree	GameVisit	2	64	0.60	0.5514
38	Oat puree	Group	1	31	0.14	0.7067
39	Oat puree	GameVisit*Group	2	64	0.62	0.5429
40	Oat puree	Gender	1	31	0.66	0.4239
41	Spinach chopped	GameVisit	2	64	4.50	0.0148
42	Spinach chopped	Group	1	31	0.08	0.7830
43	Spinach chopped	GameVisit*Group	2	64	1.17	0.3179
44	Spinach chopped	Gender	1	31	0.47	0.4983
45	Spinach puree	GameVisit	2	64	1.26	0.2899
46	Spinach puree	Group	1	31	0.03	0.8606
47	Spinach puree	GameVisit*Group	2	64	1.56	0.2177
48	Spinach puree	Gender	1	31	0.40	0.5293

OSF planned analysis: Sweetness

Obs	SampleName	StmtNo	Effect	Label	Estimate	StdErr	DF	tValue	Probt
1	Asparagus chopped	1	GameVisit*Group	Control, GV3-GV1	0.6250	3.9064	64	0.16	0.8734
2	Asparagus chopped	2	GameVisit*Group	Control, GV2-GV1	-0.8750	3.9064	64	-0.22	0.8235
3	Asparagus chopped	3	GameVisit*Group	Veg, GV3-GV1	-1.8333	3.6830	64	-0.50	0.6203
4	Asparagus chopped	4	GameVisit*Group	Veg, GV2-GV1	-3.2222	3.6830	64	-0.87	0.3849
5	Asparagus chopped	5	GameVisit*Group	Veg - Control, GV1	-2.1593	6.0067	54.13	-0.36	0.7206
6	Asparagus chopped	6	GameVisit*Group	Veg - Control, GV2	-4.5065	6.0067	54.13	-0.75	0.4564
7	Asparagus chopped	7	GameVisit*Group	Veg - Control, GV3	-4.6176	6.0067	54.13	-0.77	0.4454
8	Asparagus puree	1	GameVisit*Group	Control, GV3-GV1	-3.6875	4.1994	64	-0.88	0.3832
9	Asparagus puree	2	GameVisit*Group	Control, GV2-GV1	5.0000	4.1994	64	1.19	0.2382
10	Asparagus puree	3	GameVisit*Group	Veg, GV3-GV1	2.4444	3.9592	64	0.62	0.5392
11	Asparagus puree	4	GameVisit*Group	Veg, GV2-GV1	-2.8333	3.9592	64	-0.72	0.4768
12	Asparagus puree	5	GameVisit*Group	Veg - Control, GV1	-2.9956	7.2821	47.97	-0.41	0.6826
13	Asparagus puree	6	GameVisit*Group	Veg - Control, GV2	-10.8290	7.2821	47.97	-1.49	0.1435
14	Asparagus puree	7	GameVisit*Group	Veg - Control, GV3	3.1363	7.2821	47.97	0.43	0.6686
15	Beef puree	1	GameVisit*Group	Control, GV3-GV1	4.9375	3.1508	64	1.57	0.1220
16	Beef puree	2	GameVisit*Group	Control, GV2-GV1	4.0000	3.1508	64	1.27	0.2089
17	Beef puree	3	GameVisit*Group	Veg, GV3-GV1	4.5556	2.9706	64	1.53	0.1301
18	Beef puree	4	GameVisit*Group	Veg, GV2-GV1	1.7778	2.9706	64	0.60	0.5516
19	Beef puree	5	GameVisit*Group	Veg - Control, GV1	2.8593	6.5151	42.02	0.44	0.6630
20	Beef puree	6	GameVisit*Group	Veg - Control, GV2	0.6371	6.5151	42.02	0.10	0.9226
21	Beef puree	7	GameVisit*Group	Veg - Control, GV3	2.4773	6.5151	42.02	0.38	0.7057
22	Black bean puree	1	GameVisit*Group	Control, GV3-GV1	6.8125	4.0046	64	1.70	0.0938
23	Black bean puree	2	GameVisit*Group	Control, GV2-GV1	-2.0625	4.0046	64	-0.52	0.6083
24	Black bean puree	3	GameVisit*Group	Veg, GV3-GV1	2.3333	3.7756	64	0.62	0.5388
25	Black bean puree	4	GameVisit*Group	Veg, GV2-GV1	1.6667	3.7756	64	0.44	0.6604
26	Black bean puree	5	GameVisit*Group	Veg - Control, GV1	-0.4376	6.4935	51.14	-0.07	0.9465

Obs	SampleName	StmtNo	Effect	Label	Estimate	StdErr	DF	tValue	Probt
27	Black bean puree	6	GameVisit*Group	Veg - Control, GV2	3.2916	6.4935	51.14	0.51	0.6144
28	Black bean puree	7	GameVisit*Group	Veg - Control, GV3	-4.9168	6.4935	51.14	-0.76	0.4524
29	Broccoli chopped	1	GameVisit*Group	Control, GV3-GV1	2.1875	4.2158	64	0.52	0.6056
30	Broccoli chopped	2	GameVisit*Group	Control, GV2-GV1	1.8750	4.2158	64	0.44	0.6580
31	Broccoli chopped	3	GameVisit*Group	Veg, GV3-GV1	-5.2778	3.9747	64	-1.33	0.1889
32	Broccoli chopped	4	GameVisit*Group	Veg, GV2-GV1	-1.7778	3.9747	64	-0.45	0.6562
33	Broccoli chopped	5	GameVisit*Group	Veg - Control, GV1	4.8815	6.7635	51.7	0.72	0.4737
34	Broccoli chopped	6	GameVisit*Group	Veg - Control, GV2	1.2287	6.7635	51.7	0.18	0.8566
35	Broccoli chopped	7	GameVisit*Group	Veg - Control, GV3	-2.5838	6.7635	51.7	-0.38	0.7040
36	Broccoli puree	1	GameVisit*Group	Control, GV3-GV1	2.3750	4.0860	64	0.58	0.5631
37	Broccoli puree	2	GameVisit*Group	Control, GV2-GV1	0.3125	4.0860	64	0.08	0.9393
38	Broccoli puree	3	GameVisit*Group	Veg, GV3-GV1	-0.6667	3.8523	64	-0.17	0.8632
39	Broccoli puree	4	GameVisit*Group	Veg, GV2-GV1	-0.2222	3.8523	64	-0.06	0.9542
40	Broccoli puree	5	GameVisit*Group	Veg - Control, GV1	6.2596	7.3778	46.33	0.85	0.4006
41	Broccoli puree	6	GameVisit*Group	Veg - Control, GV2	5.7249	7.3778	46.33	0.78	0.4417
42	Broccoli puree	7	GameVisit*Group	Veg - Control, GV3	3.2179	7.3778	46.33	0.44	0.6647
43	Chicken puree	1	GameVisit*Group	Control, GV3-GV1	2.3750	2.7964	64	0.85	0.3989
44	Chicken puree	2	GameVisit*Group	Control, GV2-GV1	4.1875	2.7964	64	1.50	0.1392
45	Chicken puree	3	GameVisit*Group	Veg, GV3-GV1	-1.2778	2.6365	64	-0.48	0.6296
46	Chicken puree	4	GameVisit*Group	Veg, GV2-GV1	-1.8333	2.6365	64	-0.70	0.4893
47	Chicken puree	5	GameVisit*Group	Veg - Control, GV1	4.6584	5.6673	42.57	0.82	0.4157
48	Chicken puree	6	GameVisit*Group	Veg - Control, GV2	-1.3624	5.6673	42.57	-0.24	0.8112
49	Chicken puree	7	GameVisit*Group	Veg - Control, GV3	1.0056	5.6673	42.57	0.18	0.8600
50	Kale chopped	1	GameVisit*Group	Control, GV3-GV1	-2.7500	3.5469	64	-0.78	0.4410
51	Kale chopped	2	GameVisit*Group	Control, GV2-GV1	-2.8125	3.5469	64	-0.79	0.4307
52	Kale chopped	3	GameVisit*Group	Veg, GV3-GV1	5.0000	3.3440	64	1.50	0.1398
53	Kale chopped	4	GameVisit*Group	Veg, GV2-GV1	6.5000	3.3440	64	1.94	0.0563
54	Kale chopped	5	GameVisit*Group	Veg - Control, GV1	-4.9061	5.7352	51.29	-0.86	0.3963
55	Kale chopped	6	GameVisit*Group	Veg - Control, GV2	4.4064	5.7352	51.29	0.77	0.4458
56	Kale chopped	7	GameVisit*Group	Veg - Control, GV3	2.8439	5.7352	51.29	0.50	0.6221
57	Kale puree	1	GameVisit*Group	Control, GV3-GV1	-2.0625	3.9019	64	-0.53	0.5989
58	Kale puree	2	GameVisit*Group	Control, GV2-GV1	-0.9375	3.9019	64	-0.24	0.8109
59	Kale puree	3	GameVisit*Group	Veg, GV3-GV1	2.9444	3.6787	64	0.80	0.4264
60	Kale puree	4	GameVisit*Group	Veg, GV2-GV1	2.3889	3.6787	64	0.65	0.5184
61	Kale puree	5	GameVisit*Group	Veg - Control, GV1	-4.8216	5.6718	57.85	-0.85	0.3988
62	Kale puree	6	GameVisit*Group	Veg - Control, GV2	-1.4952	5.6718	57.85	-0.26	0.7930
63	Kale puree	7	GameVisit*Group	Veg - Control, GV3	0.1853	5.6718	57.85	0.03	0.9740
64	Oat puree	1	GameVisit*Group	Control, GV3-GV1	-1.1875	3.9699	64	-0.30	0.7658
65	Oat puree	2	GameVisit*Group	Control, GV2-GV1	-3.1250	3.9699	64	-0.79	0.4341
66	Oat puree	3	GameVisit*Group	Veg, GV3-GV1	4.7778	3.7428	64	1.28	0.2064
67	Oat puree	4	GameVisit*Group	Veg, GV2-GV1	0.7778	3.7428	64	0.21	0.8360
68	Oat puree	5	GameVisit*Group	Veg - Control, GV1	-1.4439	5.7916	57.59	-0.25	0.8040
69	Oat puree	6	GameVisit*Group	Veg - Control, GV2	2.4589	5.7916	57.59	0.42	0.6727

Obs	SampleName	StmtNo	Effect	Label	Estimate	StdErr	DF	tValue	Probt
70	Oat puree	7	GameVisit*Group	Veg - Control, GV3	4.5214	5.7916	57.59	0.78	0.4382
71	Spinach chopped	1	GameVisit*Group	Control, GV3-GV1	2.6875	3.2209	64	0.83	0.4072
72	Spinach chopped	2	GameVisit*Group	Control, GV2-GV1	-0.3125	3.2209	64	-0.10	0.9230
73	Spinach chopped	3	GameVisit*Group	Veg, GV3-GV1	7.2222	3.0367	64	2.38	0.0204
74	Spinach chopped	4	GameVisit*Group	Veg, GV2-GV1	-2.3889	3.0367	64	-0.79	0.4344
75	Spinach chopped	5	GameVisit*Group	Veg - Control, GV1	0.5422	5.5270	48.42	0.10	0.9223
76	Spinach chopped	6	GameVisit*Group	Veg - Control, GV2	-1.5342	5.5270	48.42	-0.28	0.7825
77	Spinach chopped	7	GameVisit*Group	Veg - Control, GV3	5.0770	5.5270	48.42	0.92	0.3629
78	Spinach puree	1	GameVisit*Group	Control, GV3-GV1	-0.5000	2.7093	64	-0.18	0.8542
79	Spinach puree	2	GameVisit*Group	Control, GV2-GV1	-0.7500	2.7093	64	-0.28	0.7828
80	Spinach puree	3	GameVisit*Group	Veg, GV3-GV1	6.0000	2.5543	64	2.35	0.0219
81	Spinach puree	4	GameVisit*Group	Veg, GV2-GV1	1.6111	2.5543	64	0.63	0.5305
82	Spinach puree	5	GameVisit*Group	Veg - Control, GV1	-3.7317	4.8917	46.33	-0.76	0.4494
83	Spinach puree	6	GameVisit*Group	Veg - Control, GV2	-1.3706	4.8917	46.33	-0.28	0.7806
84	Spinach puree	7	GameVisit*Group	Veg - Control, GV3	2.7683	4.8917	46.33	0.57	0.5742

OSF planned analysis: Sweetness

Obs	SampleName	Effect	GameVisit	Group	Estimate	StdErr	DF	tValue	Probt
1	Asparagus chopped	Group	_	Vegetable	20.2298	3.8157	31	5.30	<.0001
2	Asparagus chopped	Group	_	Control	23.9909	3.7575	31	6.38	<.0001
3	Asparagus chopped	GameVisit	1		22.9946	3.1817	50.9	7.23	<.0001
4	Asparagus chopped	GameVisit	2		20.9460	3.1817	50.9	6.58	<.0001
5	Asparagus chopped	GameVisit	3		22.3904	3.1817	50.9	7.04	<.0001
6	Asparagus chopped	GameVisit*Group	1	Vegetable	21.9149	4.3682	50.9	5.02	<.0001
7	Asparagus chopped	GameVisit*Group	1	Control	24.0742	4.3824	54	5.49	<.0001
8	Asparagus chopped	GameVisit*Group	2	Vegetable	18.6927	4.3682	50.9	4.28	<.0001
9	Asparagus chopped	GameVisit*Group	2	Control	23.1992	4.3824	54	5.29	<.0001
10	Asparagus chopped	GameVisit*Group	3	Vegetable	20.0816	4.3682	50.9	4.60	<.0001
11	Asparagus chopped	GameVisit*Group	3	Control	24.6992	4.3824	54	5.64	<.0001
12	Asparagus puree	Group	_	Vegetable	23.8556	4.8020	31	4.97	<.0001
13	Asparagus puree	Group	_	Control	27.4184	4.7286	31	5.80	<.0001
14	Asparagus puree	GameVisit	1		25.4830	3.8735	45.5	6.58	<.0001
15	Asparagus puree	GameVisit	2		26.5664	3.8735	45.5	6.86	<.0001
16	Asparagus puree	GameVisit	3		24.8615	3.8735	45.5	6.42	<.0001
17	Asparagus puree	GameVisit*Group	1	Vegetable	23.9852	5.3183	45.5	4.51	<.0001
18	Asparagus puree	GameVisit*Group	1	Control	26.9809	5.3140	47.8	5.08	<.0001
19	Asparagus puree	GameVisit*Group	2	Vegetable	21.1519	5.3183	45.5	3.98	0.0002
20	Asparagus puree	GameVisit*Group	2	Control	31.9809	5.3140	47.8	6.02	<.0001
21	Asparagus puree	GameVisit*Group	3	Vegetable	26.4297	5.3183	45.5	4.97	<.0001
22	Asparagus puree	GameVisit*Group	3	Control	23.2934	5.3140	47.8	4.38	<.0001
23	Beef puree	Group	_	Vegetable	16.1053	4.4618	31	3.61	0.0011
24	Beef puree	Group	_	Control	14.1140	4.3937	31	3.21	0.0031

Obs	SampleName	Effect	GameVisit	Group	Estimate	StdErr	DF	tValue	Probt
25	Beef puree	GameVisit	1		12.5645	3.4814	40.4	3.61	0.0008
26	Beef puree	GameVisit	2		15.4534	3.4814	40.4	4.44	<.0001
27	Beef puree	GameVisit	3		17.3110	3.4814	40.4	4.97	<.0001
28	Beef puree	GameVisit*Group	1	Vegetable	13.9942	4.7801	40.4	2.93	0.0056
29	Beef puree	GameVisit*Group	1	Control	11.1349	4.7554	41.9	2.34	0.0240
30	Beef puree	GameVisit*Group	2	Vegetable	15.7719	4.7801	40.4	3.30	0.0020
31	Beef puree	GameVisit*Group	2	Control	15.1349	4.7554	41.9	3.18	0.0027
32	Beef puree	GameVisit*Group	3	Vegetable	18.5497	4.7801	40.4	3.88	0.0004
33	Beef puree	GameVisit*Group	3	Control	16.0724	4.7554	41.9	3.38	0.0016
34	Black bean puree	Group	_	Vegetable	18.4241	4.1997	31	4.39	0.0001
35	Black bean puree	Group	_	Control	19.1117	4.1356	31	4.62	<.0001
36	Black bean puree	GameVisit	1		17.3095	3.4464	48.3	5.02	<.0001
37	Black bean puree	GameVisit	2		17.1116	3.4464	48.3	4.97	<.0001
38	Black bean puree	GameVisit	3		21.8825	3.4464	48.3	6.35	<.0001
39	Black bean puree	GameVisit*Group	1	Vegetable	17.0908	4.7317	48.3	3.61	0.0007
40	Black bean puree	GameVisit*Group	1	Control	17.5283	4.7380	51	3.70	0.0005
41	Black bean puree	GameVisit*Group	2	Vegetable	18.7574	4.7317	48.3	3.96	0.0002
42	Black bean puree	GameVisit*Group	2	Control	15.4658	4.7380	51	3.26	0.0020
43	Black bean puree	GameVisit*Group	3	Vegetable	19.4241	4.7317	48.3	4.11	0.0002
44	Black bean puree	GameVisit*Group	3	Control	24.3408	4.7380	51	5.14	<.0001
45	Broccoli chopped	Group	_	Vegetable	25.4781	4.3595	31	5.84	<.0001
46	Broccoli chopped	Group	_	Control	24.3026	4.2929	31	5.66	<.0001
47	Broccoli chopped	GameVisit	1		25.3892	3.5883	48.8	7.08	<.0001
48	Broccoli chopped	GameVisit	2		25.4378	3.5883	48.8	7.09	<.0001
49	Broccoli chopped	GameVisit	3		23.8441	3.5883	48.8	6.64	<.0001
50	Broccoli chopped	GameVisit*Group	1	Vegetable	27.8299	4.9266	48.7	5.65	<.0001
51	Broccoli chopped	GameVisit*Group	1	Control	22.9485	4.9349	51.6	4.65	<.0001
52	Broccoli chopped	GameVisit*Group	2	Vegetable	26.0521	4.9266	48.7	5.29	<.0001
53	Broccoli chopped	GameVisit*Group	2	Control	24.8235	4.9349	51.6	5.03	<.0001
54	Broccoli chopped	GameVisit*Group	3	Vegetable	22.5521	4.9266	48.7	4.58	<.0001
55	Broccoli chopped	GameVisit*Group	3	Control	25.1360	4.9349	51.6	5.09	<.0001
56	Broccoli puree	Group	_	Vegetable	27.5621	4.9149	31	5.61	<.0001
57	Broccoli puree	Group	_	Control	22.4946	4.8399	31	4.65	<.0001
58	Broccoli puree	GameVisit	1		24.7286	3.9291	44.1	6.29	<.0001
59	Broccoli puree	GameVisit	2		24.7737	3.9291	44.1	6.31	<.0001
60	Broccoli puree	GameVisit	3		25.5827	3.9291	44.1	6.51	<.0001
61	Broccoli puree	GameVisit*Group	1	Vegetable	27.8584	5.3947	44.1	5.16	<.0001
62	Broccoli puree	GameVisit*Group	1	Control	21.5988	5.3842	46.2	4.01	0.0002
63	Broccoli puree	GameVisit*Group	2	Vegetable	27.6362	5.3947	44.1	5.12	<.0001
64	Broccoli puree	GameVisit*Group	2	Control	21.9113	5.3842	46.2	4.07	0.0002
65	Broccoli puree	GameVisit*Group	3	Vegetable	27.1917	5.3947	44.1	5.04	<.0001
66	Broccoli puree	GameVisit*Group	3	Control	23.9738	5.3842	46.2	4.45	<.0001
67	Chicken puree	Group	_	Vegetable	13.5692	3.8674	31	3.51	0.0014

Obs	SampleName	Effect	GameVisit	Group	Estimate	StdErr	DF	tValue	Probt
68	Chicken puree	Group	_	Control	12.1353	3.8084	31	3.19	0.0033
69	Chicken puree	GameVisit	1		12.2770	3.0270	40.9	4.06	0.0002
70	Chicken puree	GameVisit	2		13.4541	3.0270	40.9	4.44	<.0001
71	Chicken puree	GameVisit	3		12.8256	3.0270	40.9	4.24	0.0001
72	Chicken puree	GameVisit*Group	1	Vegetable	14.6062	4.1562	40.9	3.51	0.0011
73	Chicken puree	GameVisit*Group	1	Control	9.9478	4.1365	42.5	2.40	0.0206
74	Chicken puree	GameVisit*Group	2	Vegetable	12.7729	4.1562	40.9	3.07	0.0038
75	Chicken puree	GameVisit*Group	2	Control	14.1353	4.1365	42.5	3.42	0.0014
76	Chicken puree	GameVisit*Group	3	Vegetable	13.3284	4.1562	40.9	3.21	0.0026
77	Chicken puree	GameVisit*Group	3	Control	12.3228	4.1365	42.5	2.98	0.0048
78	Kale chopped	Group	_	Vegetable	15.8222	3.7060	31	4.27	0.0002
79	Kale chopped	Group	_	Control	15.0408	3.6494	31	4.12	0.0003
80	Kale chopped	GameVisit	1		14.4419	3.0436	48.4	4.74	<.0001
81	Kale chopped	GameVisit	2		16.2857	3.0436	48.4	5.35	<.0001
82	Kale chopped	GameVisit	3		15.5669	3.0436	48.4	5.11	<.0001
83	Kale chopped	GameVisit*Group	1	Vegetable	11.9889	4.1788	48.4	2.87	0.0061
84	Kale chopped	GameVisit*Group	1	Control	16.8950	4.1847	51.1	4.04	0.0002
85	Kale chopped	GameVisit*Group	2	Vegetable	18.4889	4.1788	48.4	4.42	<.0001
86	Kale chopped	GameVisit*Group	2	Control	14.0825	4.1847	51.1	3.37	0.0015
87	Kale chopped	GameVisit*Group	3	Vegetable	16.9889	4.1788	48.4	4.07	0.0002
88	Kale chopped	GameVisit*Group	3	Control	14.1450	4.1847	51.1	3.38	0.0014
89	Kale puree	Group	_	Vegetable	13.1930	3.5243	31	3.74	0.0007
90	Kale puree	Group	_	Control	15.2368	3.4705	31	4.39	0.0001
91	Kale puree	GameVisit	1		13.8260	2.9972	54.2	4.61	<.0001
92	Kale puree	GameVisit	2		14.5517	2.9972	54.2	4.86	<.0001
93	Kale puree	GameVisit	3		14.2670	2.9972	54.2	4.76	<.0001
94	Kale puree	GameVisit*Group	1	Vegetable	11.4152	4.1149	54.1	2.77	0.0076
95	Kale puree	GameVisit*Group	1	Control	16.2368	4.1376	57.7	3.92	0.0002
96	Kale puree	GameVisit*Group	2	Vegetable	13.8041	4.1149	54.1	3.35	0.0015
97	Kale puree	GameVisit*Group	2	Control	15.2993	4.1376	57.7	3.70	0.0005
98	Kale puree	GameVisit*Group	3	Vegetable	14.3596	4.1149	54.1	3.49	0.0010
99	Kale puree	GameVisit*Group	3	Control	14.1743	4.1376	57.7	3.43	0.0011
100	Oat puree	Group	_	Vegetable	17.4312	3.6043	31	4.84	<.0001
101	Oat puree	Group	_	Control	15.5857	3.5492	31	4.39	0.0001
102	Oat puree	GameVisit	1		16.3013	3.0610	54	5.33	<.0001
103	Oat puree	GameVisit	2		15.1276	3.0610	54	4.94	<.0001
104	Oat puree	GameVisit	3		18.0964	3.0610	54	5.91	<.0001
105	Oat puree	GameVisit*Group	1	Vegetable	15.5793	4.2024	53.9	3.71	0.0005
106	Oat puree	GameVisit*Group	1	Control	17.0232	4.2250	57.4	4.03	0.0002
107	Oat puree	GameVisit*Group	2	Vegetable	16.3571	4.2024	53.9	3.89	0.0003
108	Oat puree	GameVisit*Group	2	Control	13.8982	4.2250	57.4	3.29	0.0017
109	Oat puree	GameVisit*Group	3	Vegetable	20.3571	4.2024	53.9	4.84	<.0001
110	Oat puree	GameVisit*Group	3	Control	15.8357	4.2250	57.4	3.75	0.0004

Obs	SampleName	Effect	GameVisit	Group	Estimate	StdErr	DF	tValue	Probt
111	Spinach chopped	Group	_	Vegetable	13.7561	3.6344	31	3.78	0.0007
112	Spinach chopped	Group	_	Control	12.3944	3.5789	31	3.46	0.0016
113	Spinach chopped	GameVisit	1		11.8738	2.9390	45.9	4.04	0.0002
114	Spinach chopped	GameVisit	2		10.5232	2.9390	45.9	3.58	0.0008
115	Spinach chopped	GameVisit	3		16.8287	2.9390	45.9	5.73	<.0001
116	Spinach chopped	GameVisit*Group	1	Vegetable	12.1450	4.0352	45.9	3.01	0.0042
117	Spinach chopped	GameVisit*Group	1	Control	11.6027	4.0332	48.3	2.88	0.0060
118	Spinach chopped	GameVisit*Group	2	Vegetable	9.7561	4.0352	45.9	2.42	0.0196
119	Spinach chopped	GameVisit*Group	2	Control	11.2902	4.0332	48.3	2.80	0.0073
120	Spinach chopped	GameVisit*Group	3	Vegetable	19.3672	4.0352	45.9	4.80	<.0001
121	Spinach chopped	GameVisit*Group	3	Control	14.2902	4.0332	48.3	3.54	0.0009
122	Spinach puree	Group	_	Vegetable	11.2976	3.2587	31	3.47	0.0016
123	Spinach puree	Group	_	Control	12.0756	3.2089	31	3.76	0.0007
124	Spinach puree	GameVisit	1		10.6264	2.6051	44.1	4.08	0.0002
125	Spinach puree	GameVisit	2		11.0569	2.6051	44.1	4.24	0.0001
126	Spinach puree	GameVisit	3		13.3764	2.6051	44.1	5.13	<.0001
127	Spinach puree	GameVisit*Group	1	Vegetable	8.7605	3.5769	44.1	2.45	0.0184
128	Spinach puree	GameVisit*Group	1	Control	12.4922	3.5699	46.2	3.50	0.0010
129	Spinach puree	GameVisit*Group	2	Vegetable	10.3716	3.5769	44.1	2.90	0.0058
130	Spinach puree	GameVisit*Group	2	Control	11.7422	3.5699	46.2	3.29	0.0019
131	Spinach puree	GameVisit*Group	3	Vegetable	14.7605	3.5769	44.1	4.13	0.0002
132	Spinach puree	GameVisit*Group	3	Control	11.9922	3.5699	46.2	3.36	0.0016

OSF planned analysis: Bitterness

Obs	SampleName	Effect	NumDF	DenDF	FValue	ProbF
1	Asparagus chopped	GameVisit	2	64	4.84	0.0110
2	Asparagus chopped	Group	1	31	3.47	0.0721
3	Asparagus chopped	GameVisit*Group	2	64	0.82	0.4433
4	Asparagus chopped	Gender	1	31	0.25	0.6226
5	Asparagus puree	GameVisit	2	64	0.50	0.6073
6	Asparagus puree	Group	1	31	2.35	0.1352
7	Asparagus puree	GameVisit*Group	2	64	0.39	0.6782
8	Asparagus puree	Gender	1	31	0.08	0.7742
9	Beef puree	GameVisit	2	64	1.51	0.2295
10	Beef puree	Group	1	31	0.00	0.9590
11	Beef puree	GameVisit*Group	2	64	2.61	0.0813
12	Beef puree	Gender	1	31	0.15	0.7041
13	Black bean puree	GameVisit	2	64	5.14	0.0085
14	Black bean puree	Group	1	31	0.06	0.8141
15	Black bean puree	GameVisit*Group	2	64	0.20	0.8197
16	Black bean puree	Gender	1	31	0.52	0.4755
17	Broccoli chopped	GameVisit	2	64	2.07	0.1351

Obs	SampleName	Effect	NumDF	DenDF	FValue	ProbF
18	Broccoli chopped	Group	1	31	0.01	0.9149
19	Broccoli chopped	GameVisit*Group	2	64	0.69	0.5036
20	Broccoli chopped	Gender	1	31	0.93	0.3412
21	Broccoli puree	GameVisit	2	64	0.30	0.7392
22	Broccoli puree	Group	1	31	0.22	0.6415
23	Broccoli puree	GameVisit*Group	2	64	0.22	0.8029
24	Broccoli puree	Gender	1	31	0.10	0.7518
25	Chicken puree	GameVisit	2	64	1.04	0.3610
26	Chicken puree	Group	1	31	0.02	0.9020
27	Chicken puree	GameVisit*Group	2	64	1.11	0.3358
28	Chicken puree	Gender	1	31	0.10	0.7598
29	Kale chopped	GameVisit	2	64	0.25	0.7806
30	Kale chopped	Group	1	31	0.00	0.9912
31	Kale chopped	GameVisit*Group	2	64	0.41	0.6679
32	Kale chopped	Gender	1	31	0.00	0.9459
33	Kale puree	GameVisit	2	64	0.56	0.5763
34	Kale puree	Group	1	31	0.14	0.7072
35	Kale puree	GameVisit*Group	2	64	0.67	0.5136
36	Kale puree	Gender	1	31	0.44	0.5101
37	Oat puree	GameVisit	2	64	0.07	0.9289
38	Oat puree	Group	1	31	0.06	0.8127
39	Oat puree	GameVisit*Group	2	64	0.58	0.5623
40	Oat puree	Gender	1	31	1.43	0.2408
41	Spinach chopped	GameVisit	2	64	8.76	0.0004
42	Spinach chopped	Group	1	31	0.39	0.5349
43	Spinach chopped	GameVisit*Group	2	64	0.39	0.6799
44	Spinach chopped	Gender	1	31	0.23	0.6330
45	Spinach puree	GameVisit	2	64	0.96	0.3869
46	Spinach puree	Group	1	31	0.04	0.8495
47	Spinach puree	GameVisit*Group	2	64	0.59	0.5560
48	Spinach puree	Gender	1	31	0.03	0.8712

OSF planned analysis: Bitterness

Obs	SampleName	StmtNo	Effect	Label	Estimate	StdErr	DF	tValue	Probt
1	Asparagus chopped	1	GameVisit*Group	Control, GV3-GV1	8.5000	5.2271	64	1.63	0.1088
2	Asparagus chopped	2	GameVisit*Group	Control, GV2-GV1	13.3125	5.2271	64	2.55	0.0133
3	Asparagus chopped	3	GameVisit*Group	Veg, GV3-GV1	-0.7222	4.9282	64	-0.15	0.8839
4	Asparagus chopped	4	GameVisit*Group	Veg, GV2-GV1	8.7222	4.9282	64	1.77	0.0815
5	Asparagus chopped	5	GameVisit*Group	Veg - Control, GV1	17.5799	8.1099	53.59	2.17	0.0346
6	Asparagus chopped	6	GameVisit*Group	Veg - Control, GV2	12.9896	8.1099	53.59	1.60	0.1151
7	Asparagus chopped	7	GameVisit*Group	Veg - Control, GV3	8.3577	8.1099	53.59	1.03	0.3074
8	Asparagus puree	1	GameVisit*Group	Control, GV3-GV1	5.1875	4.8232	64	1.08	0.2862

Obs	SampleName	StmtNo	Effect	Label	Estimate	StdErr	DF	tValue	Probt
9	Asparagus puree	2	GameVisit*Group	Control, GV2-GV1	4.8125	4.8232	64	1.00	0.3221
10	Asparagus puree	3	GameVisit*Group	Veg, GV3-GV1	-0.6667	4.5474	64	-0.15	0.8839
11	Asparagus puree	4	GameVisit*Group	Veg, GV2-GV1	1.6667	4.5474	64	0.37	0.7152
12	Asparagus puree	5	GameVisit*Group	Veg - Control, GV1	12.8151	7.4554	53.82	1.72	0.0914
13	Asparagus puree	6	GameVisit*Group	Veg - Control, GV2	9.6693	7.4554	53.82	1.30	0.2002
14	Asparagus puree	7	GameVisit*Group	Veg - Control, GV3	6.9609	7.4554	53.82	0.93	0.3546
15	Beef puree	1	GameVisit*Group	Control, GV3-GV1	-6.5625	4.1644	64	-1.58	0.1200
16	Beef puree	2	GameVisit*Group	Control, GV2-GV1	4.6875	4.1644	64	1.13	0.2645
17	Beef puree	3	GameVisit*Group	Veg, GV3-GV1	2.6667	3.9263	64	0.68	0.4995
18	Beef puree	4	GameVisit*Group	Veg, GV2-GV1	1.2778	3.9263	64	0.33	0.7459
19	Beef puree	5	GameVisit*Group	Veg - Control, GV1	-1.5410	8.3742	42.78	-0.18	0.8549
20	Beef puree	6	GameVisit*Group	Veg - Control, GV2	-4.9508	8.3742	42.78	-0.59	0.5575
21	Beef puree	7	GameVisit*Group	Veg - Control, GV3	7.6881	8.3742	42.78	0.92	0.3637
22	Black bean puree	1	GameVisit*Group	Control, GV3-GV1	0.6250	4.5148	64	0.14	0.8903
23	Black bean puree	2	GameVisit*Group	Control, GV2-GV1	10.5625	4.5148	64	2.34	0.0224
24	Black bean puree	3	GameVisit*Group	Veg, GV3-GV1	-0.3889	4.2566	64	-0.09	0.9275
25	Black bean puree	4	GameVisit*Group	Veg, GV2-GV1	6.7778	4.2566	64	1.59	0.1162
26	Black bean puree	5	GameVisit*Group	Veg - Control, GV1	0.008444	7.6055	49.26	0.00	0.9991
27	Black bean puree	6	GameVisit*Group	Veg - Control, GV2	-3.7763	7.6055	49.26	-0.50	0.6217
28	Black bean puree	7	GameVisit*Group	Veg - Control, GV3	-1.0054	7.6055	49.26	-0.13	0.8954
29	Broccoli chopped	1	GameVisit*Group	Control, GV3-GV1	-1.1250	3.2751	64	-0.34	0.7323
30	Broccoli chopped	2	GameVisit*Group	Control, GV2-GV1	1.6250	3.2751	64	0.50	0.6215
31	Broccoli chopped	3	GameVisit*Group	Veg, GV3-GV1	2.3333	3.0878	64	0.76	0.4526
32	Broccoli chopped	4	GameVisit*Group	Veg, GV2-GV1	6.8333	3.0878	64	2.21	0.0305
33	Broccoli chopped	5	GameVisit*Group	Veg - Control, GV1	-3.5798	6.9232	41.46	-0.52	0.6078
34	Broccoli chopped	6	GameVisit*Group	Veg - Control, GV2	1.6285	6.9232	41.46	0.24	0.8152
35	Broccoli chopped	7	GameVisit*Group	Veg - Control, GV3	-0.1215	6.9232	41.46	-0.02	0.9861
36	Broccoli puree	1	GameVisit*Group	Control, GV3-GV1	-3.5625	4.3099	64	-0.83	0.4115
37	Broccoli puree	2	GameVisit*Group	Control, GV2-GV1	-1.0625	4.3099	64	-0.25	0.8061
38	Broccoli puree	3	GameVisit*Group	Veg, GV3-GV1	-0.8889	4.0634	64	-0.22	0.8275
39	Broccoli puree	4	GameVisit*Group	Veg, GV2-GV1	-2.2222	4.0634	64	-0.55	0.5864
40	Broccoli puree	5	GameVisit*Group	Veg - Control, GV1	2.3307	6.9329	51.56	0.34	0.7381
41	Broccoli puree	6	GameVisit*Group	Veg - Control, GV2	1.1710	6.9329	51.56	0.17	0.8665
42	Broccoli puree	7	GameVisit*Group	Veg - Control, GV3	5.0043	6.9329	51.56	0.72	0.4737
43	Chicken puree	1	GameVisit*Group	Control, GV3-GV1	-0.1875	3.6864	64	-0.05	0.9596
44	Chicken puree	2	GameVisit*Group	Control, GV2-GV1	4.5625	3.6864	64	1.24	0.2204
45	Chicken puree	3	GameVisit*Group	Veg, GV3-GV1	5.1111	3.4756	64	1.47	0.1463
46	Chicken puree	4	GameVisit*Group	Veg, GV2-GV1	2.5556	3.4756	64	0.74	0.4649
47	Chicken puree	5	GameVisit*Group	Veg - Control, GV1	0.03166	9.5489	37.56	0.00	0.9974
48	Chicken puree	6	GameVisit*Group	Veg - Control, GV2	-1.9753	9.5489	37.56	-0.21	0.8372
49	Chicken puree	7	GameVisit*Group	Veg - Control, GV3	5.3303	9.5489	37.56	0.56	0.5800
50	Kale chopped	1	GameVisit*Group	Control, GV3-GV1	4.1250	5.2662	64	0.78	0.4363
51	Kale chopped	2	GameVisit*Group	Control, GV2-GV1	5.4375	5.2662	64	1.03	0.3057

Obs	SampleName	StmtNo	Effect	Label	Estimate	StdErr	DF	tValue	Probt
52	Kale chopped	3	GameVisit*Group	Veg, GV3-GV1	0.3333	4.9651	64	0.07	0.9467
53	Kale chopped	4	GameVisit*Group	Veg, GV2-GV1	-1.0556	4.9651	64	-0.21	0.8323
54	Kale chopped	5	GameVisit*Group	Veg - Control, GV1	3.3459	8.4882	51.45	0.39	0.6951
55	Kale chopped	6	GameVisit*Group	Veg - Control, GV2	-3.1471	8.4882	51.45	-0.37	0.7123
56	Kale chopped	7	GameVisit*Group	Veg - Control, GV3	-0.4457	8.4882	51.45	-0.05	0.9583
57	Kale puree	1	GameVisit*Group	Control, GV3-GV1	7.3125	6.8429	64	1.07	0.2893
58	Kale puree	2	GameVisit*Group	Control, GV2-GV1	5.6250	6.8429	64	0.82	0.4141
59	Kale puree	3	GameVisit*Group	Veg, GV3-GV1	1.4444	6.4516	64	0.22	0.8236
60	Kale puree	4	GameVisit*Group	Veg, GV2-GV1	-5.2778	6.4516	64	-0.82	0.4164
61	Kale puree	5	GameVisit*Group	Veg - Control, GV1	3.2880	8.1459	76.65	0.40	0.6876
62	Kale puree	6	GameVisit*Group	Veg - Control, GV2	-7.6148	8.1459	76.65	-0.93	0.3528
63	Kale puree	7	GameVisit*Group	Veg - Control, GV3	-2.5801	8.1459	76.65	-0.32	0.7523
64	Oat puree	1	GameVisit*Group	Control, GV3-GV1	2.8125	3.5852	64	0.78	0.4357
65	Oat puree	2	GameVisit*Group	Control, GV2-GV1	3.3125	3.5852	64	0.92	0.3590
66	Oat puree	3	GameVisit*Group	Veg, GV3-GV1	-1.6111	3.3801	64	-0.48	0.6352
67	Oat puree	4	GameVisit*Group	Veg, GV2-GV1	-1.4444	3.3801	64	-0.43	0.6706
68	Oat puree	5	GameVisit*Group	Veg - Control, GV1	1.3152	7.8347	40.68	0.17	0.8675
69	Oat puree	6	GameVisit*Group	Veg - Control, GV2	-3.4417	7.8347	40.68	-0.44	0.6628
70	Oat puree	7	GameVisit*Group	Veg - Control, GV3	-3.1084	7.8347	40.68	-0.40	0.6936
71	Spinach chopped	1	GameVisit*Group	Control, GV3-GV1	-5.5000	4.5623	64	-1.21	0.2324
72	Spinach chopped	2	GameVisit*Group	Control, GV2-GV1	6.3125	4.5623	64	1.38	0.1713
73	Spinach chopped	3	GameVisit*Group	Veg, GV3-GV1	-11.0000	4.3014	64	-2.56	0.0129
74	Spinach chopped	4	GameVisit*Group	Veg, GV2-GV1	3.1111	4.3014	64	0.72	0.4721
75	Spinach chopped	5	GameVisit*Group	Veg - Control, GV1	-0.9308	7.0979	53.43	-0.13	0.8962
76	Spinach chopped	6	GameVisit*Group	Veg - Control, GV2	-4.1322	7.0979	53.43	-0.58	0.5629
77	Spinach chopped	7	GameVisit*Group	Veg - Control, GV3	-6.4308	7.0979	53.43	-0.91	0.3690
78	Spinach puree	1	GameVisit*Group	Control, GV3-GV1	1.4375	5.6712	64	0.25	0.8007
79	Spinach puree	2	GameVisit*Group	Control, GV2-GV1	8.9375	5.6712	64	1.58	0.1200
80	Spinach puree	3	GameVisit*Group	Veg, GV3-GV1	-0.8333	5.3468	64	-0.16	0.8766
81	Spinach puree	4	GameVisit*Group	Veg, GV2-GV1	0.7222	5.3468	64	0.14	0.8930
82	Spinach puree	5	GameVisit*Group	Veg - Control, GV1	1.9866	9.0786	51.82	0.22	0.8276
83	Spinach puree	6	GameVisit*Group	Veg - Control, GV2	-6.2287	9.0786	51.82	-0.69	0.4957
84	Spinach puree	7	GameVisit*Group	Veg - Control, GV3	-0.2842	9.0786	51.82	-0.03	0.9751

OSF planned analysis: Bitterness

Obs	SampleName	Effect	GameVisit	Group	Estimate	StdErr	DF	tValue	Probt
1	Asparagus chopped	Group	_	Vegetable	40.2210	5.1683	31	7.78	<.0001
2	Asparagus chopped	Group	_	Control	27.2453	5.0894	31	5.35	<.0001
3	Asparagus chopped	GameVisit	1		28.7644	4.2972	50.4	6.69	<.0001
4	Asparagus chopped	GameVisit	2		39.7817	4.2972	50.4	9.26	<.0001
5	Asparagus chopped	GameVisit	3		32.6533	4.2972	50.4	7.60	<.0001
6	Asparagus chopped	GameVisit*Group	1	Vegetable	37.5543	5.8998	50.4	6.37	<.0001

Obs	SampleName	Effect	GameVisit	Group	Estimate	StdErr	DF	tValue	Probt
7	Asparagus chopped	GameVisit*Group	1	Control	19.9744	5.9169	53.4	3.38	0.0014
8	Asparagus chopped	GameVisit*Group	2	Vegetable	46.2765	5.8998	50.4	7.84	<.0001
9	Asparagus chopped	GameVisit*Group	2	Control	33.2869	5.9169	53.4	5.63	<.0001
10	Asparagus chopped	GameVisit*Group	3	Vegetable	36.8321	5.8998	50.4	6.24	<.0001
11	Asparagus chopped	GameVisit*Group	3	Control	28.4744	5.9169	53.4	4.81	<.0001
12	Asparagus puree	Group	_	Vegetable	35.2699	4.7449	31	7.43	<.0001
13	Asparagus puree	Group	_	Control	25.4548	4.6725	31	5.45	<.0001
14	Asparagus puree	GameVisit	1		28.5290	3.9498	50.6	7.22	<.0001
15	Asparagus puree	GameVisit	2		31.7686	3.9498	50.6	8.04	<.0001
16	Asparagus puree	GameVisit	3		30.7894	3.9498	50.6	7.80	<.0001
17	Asparagus puree	GameVisit*Group	1	Vegetable	34.9366	5.4228	50.6	6.44	<.0001
18	Asparagus puree	GameVisit*Group	1	Control	22.1215	5.4394	53.7	4.07	0.0002
19	Asparagus puree	GameVisit*Group	2	Vegetable	36.6032	5.4228	50.6	6.75	<.0001
20	Asparagus puree	GameVisit*Group	2	Control	26.9340	5.4394	53.7	4.95	<.0001
21	Asparagus puree	GameVisit*Group	3	Vegetable	34.2699	5.4228	50.6	6.32	<.0001
22	Asparagus puree	GameVisit*Group	3	Control	27.3090	5.4394	53.7	5.02	<.0001
23	Beef puree	Group	_	Vegetable	25.4902	5.7065	31	4.47	<.0001
24	Beef puree	Group	_	Control	25.0914	5.6194	31	4.47	<.0001
25	Beef puree	GameVisit	1		24.9459	4.4720	41.1	5.58	<.0001
26	Beef puree	GameVisit	2		27.9286	4.4720	41.1	6.25	<.0001
27	Beef puree	GameVisit	3		22.9980	4.4720	41.1	5.14	<.0001
28	Beef puree	GameVisit*Group	1	Vegetable	24.1754	6.1402	41.1	3.94	0.0003
29	Beef puree	GameVisit*Group	1	Control	25.7164	6.1121	42.7	4.21	0.0001
30	Beef puree	GameVisit*Group	2	Vegetable	25.4532	6.1402	41.1	4.15	0.0002
31	Beef puree	GameVisit*Group	2	Control	30.4039	6.1121	42.7	4.97	<.0001
32	Beef puree	GameVisit*Group	3	Vegetable	26.8421	6.1402	41.1	4.37	<.0001
33	Beef puree	GameVisit*Group	3	Control	19.1539	6.1121	42.7	3.13	0.0031
34	Black bean puree	Group	_	Vegetable	21.0995	4.9754	31	4.24	0.0002
35	Black bean puree	Group	_	Control	22.6906	4.8995	31	4.63	<.0001
36	Black bean puree	GameVisit	1		18.9657	4.0418	46.7	4.69	<.0001
37	Black bean puree	GameVisit	2		27.6358	4.0418	46.7	6.84	<.0001
38	Black bean puree	GameVisit	3		19.0837	4.0418	46.7	4.72	<.0001
39	Black bean puree	GameVisit*Group	1	Vegetable	18.9699	5.5493	46.6	3.42	0.0013
40	Black bean puree	GameVisit*Group	1	Control	18.9615	5.5497	49.1	3.42	0.0013
41	Black bean puree	GameVisit*Group	2	Vegetable	25.7477	5.5493	46.6	4.64	<.0001
42	Black bean puree	GameVisit*Group	2	Control	29.5240	5.5497	49.1	5.32	<.0001
43	Black bean puree	GameVisit*Group	3	Vegetable	18.5810	5.5493	46.6	3.35	0.0016
44	Black bean puree	GameVisit*Group	3	Control	19.5865	5.5497	49.1	3.53	0.0009
45	Broccoli chopped	Group	_	Vegetable	25.8877	4.7590	31	5.44	<.0001
46	Broccoli chopped	Group	_	Control	26.5786	4.6863	31	5.67	<.0001
47	Broccoli chopped	GameVisit	1		24.6220	3.7011	39.9	6.65	<.0001
48	Broccoli chopped	GameVisit	2		28.8512	3.7011	39.9	7.80	<.0001
49	Broccoli chopped	GameVisit	3		25.2262	3.7011	39.9	6.82	<.0001

Obs	SampleName	Effect	GameVisit	Group	Estimate	StdErr	DF	tValue	Probt
50	Broccoli chopped	GameVisit*Group	1	Vegetable	22.8321	5.0819	39.9	4.49	<.0001
51	Broccoli chopped	GameVisit*Group	1	Control	26.4119	5.0534	41.4	5.23	<.0001
52	Broccoli chopped	GameVisit*Group	2	Vegetable	29.6654	5.0819	39.9	5.84	<.0001
53	Broccoli chopped	GameVisit*Group	2	Control	28.0369	5.0534	41.4	5.55	<.0001
54	Broccoli chopped	GameVisit*Group	3	Vegetable	25.1654	5.0819	39.9	4.95	<.0001
55	Broccoli chopped	GameVisit*Group	3	Control	25.2869	5.0534	41.4	5.00	<.0001
56	Broccoli puree	Group	_	Vegetable	22.1552	4.4725	31	4.95	<.0001
57	Broccoli puree	Group	_	Control	19.3198	4.4042	31	4.39	0.0001
58	Broccoli puree	GameVisit	1		22.0269	3.6785	48.7	5.99	<.0001
59	Broccoli puree	GameVisit	2		20.3845	3.6785	48.7	5.54	<.0001
60	Broccoli puree	GameVisit	3		19.8012	3.6785	48.7	5.38	<.0001
61	Broccoli puree	GameVisit*Group	1	Vegetable	23.1922	5.0504	48.6	4.59	<.0001
62	Broccoli puree	GameVisit*Group	1	Control	20.8615	5.0585	51.4	4.12	0.0001
63	Broccoli puree	GameVisit*Group	2	Vegetable	20.9700	5.0504	48.6	4.15	0.0001
64	Broccoli puree	GameVisit*Group	2	Control	19.7990	5.0585	51.4	3.91	0.0003
65	Broccoli puree	GameVisit*Group	3	Vegetable	22.3033	5.0504	48.6	4.42	<.0001
66	Broccoli puree	GameVisit*Group	3	Control	17.2990	5.0585	51.4	3.42	0.0012
67	Chicken puree	Group	_	Vegetable	28.9086	6.7412	31	4.29	0.0002
68	Chicken puree	Group	_	Control	27.7797	6.6383	31	4.18	0.0002
69	Chicken puree	GameVisit	1		26.3372	5.1223	36.6	5.14	<.0001
70	Chicken puree	GameVisit	2		29.8962	5.1223	36.6	5.84	<.0001
71	Chicken puree	GameVisit	3		28.7990	5.1223	36.6	5.62	<.0001
72	Chicken puree	GameVisit*Group	1	Vegetable	26.3530	7.0335	36.6	3.75	0.0006
73	Chicken puree	GameVisit*Group	1	Control	26.3214	6.9711	37.5	3.78	0.0006
74	Chicken puree	GameVisit*Group	2	Vegetable	28.9086	7.0335	36.6	4.11	0.0002
75	Chicken puree	GameVisit*Group	2	Control	30.8839	6.9711	37.5	4.43	<.0001
76	Chicken puree	GameVisit*Group	3	Vegetable	31.4641	7.0335	36.6	4.47	<.0001
77	Chicken puree	GameVisit*Group	3	Control	26.1339	6.9711	37.5	3.75	0.0006
78	Kale chopped	Group	_	Vegetable	42.1609	5.4793	31	7.69	<.0001
79	Kale chopped	Group	_	Control	42.2433	5.3957	31	7.83	<.0001
80	Kale chopped	GameVisit	1		40.7287	4.5041	48.6	9.04	<.0001
81	Kale chopped	GameVisit	2		42.9197	4.5041	48.6	9.53	<.0001
82	Kale chopped	GameVisit	3		42.9579	4.5041	48.6	9.54	<.0001
83	Kale chopped	GameVisit*Group	1	Vegetable	42.4017	6.1839	48.5	6.86	<.0001
84	Kale chopped	GameVisit*Group	1	Control	39.0558	6.1934	51.3	6.31	<.0001
85	Kale chopped	GameVisit*Group	2	Vegetable	41.3461	6.1839	48.5	6.69	<.0001
86	Kale chopped	GameVisit*Group	2	Control	44.4933	6.1934	51.3	7.18	<.0001
87	Kale chopped	GameVisit*Group	3	Vegetable	42.7350	6.1839	48.5	6.91	<.0001
88	Kale chopped	GameVisit*Group	3	Control	43.1808	6.1934	51.3	6.97	<.0001
89	Kale puree	Group	_	Vegetable	36.7686	4.5034	31	8.16	<.0001
90	Kale puree	Group	_	Control	39.0709	4.4346	31	8.81	<.0001
91	Kale puree	GameVisit	1		36.4023	4.2574	71.7	8.55	<.0001
92	Kale puree	GameVisit	2		36.5760	4.2574	71.7	8.59	<.0001

Obs	SampleName	Effect	GameVisit	Group	Estimate	StdErr	DF	tValue	Probt
93	Kale puree	GameVisit	3		40.7808	4.2574	71.7	9.58	<.0001
94	Kale puree	GameVisit*Group	1	Vegetable	38.0463	5.8442	71.7	6.51	<.0001
95	Kale puree	GameVisit*Group	1	Control	34.7584	5.9392	76.4	5.85	<.0001
96	Kale puree	GameVisit*Group	2	Vegetable	32.7686	5.8442	71.7	5.61	<.0001
97	Kale puree	GameVisit*Group	2	Control	40.3834	5.9392	76.4	6.80	<.0001
98	Kale puree	GameVisit*Group	3	Vegetable	39.4908	5.8442	71.7	6.76	<.0001
99	Kale puree	GameVisit*Group	3	Control	42.0709	5.9392	76.4	7.08	<.0001
100	Oat puree	Group	_	Vegetable	24.8047	5.4138	31	4.58	<.0001
101	Oat puree	Group	_	Control	26.5496	5.3312	31	4.98	<.0001
102	Oat puree	GameVisit	1		25.1656	4.1912	39.3	6.00	<.0001
103	Oat puree	GameVisit	2		26.0996	4.1912	39.3	6.23	<.0001
104	Oat puree	GameVisit	3		25.7662	4.1912	39.3	6.15	<.0001
105	Oat puree	GameVisit*Group	1	Vegetable	25.8232	5.7548	39.3	4.49	<.0001
106	Oat puree	GameVisit*Group	1	Control	24.5079	5.7189	40.6	4.29	0.0001
107	Oat puree	GameVisit*Group	2	Vegetable	24.3787	5.7548	39.3	4.24	0.0001
108	Oat puree	GameVisit*Group	2	Control	27.8204	5.7189	40.6	4.86	<.0001
109	Oat puree	GameVisit*Group	3	Vegetable	24.2121	5.7548	39.3	4.21	0.0001
110	Oat puree	GameVisit*Group	3	Control	27.3204	5.7189	40.6	4.78	<.0001
111	Spinach chopped	Group	_	Vegetable	30.6552	4.5278	31	6.77	<.0001
112	Spinach chopped	Group	_	Control	34.4865	4.4587	31	7.73	<.0001
113	Spinach chopped	GameVisit	1		33.7502	3.7614	50.3	8.97	<.0001
114	Spinach chopped	GameVisit	2		38.4621	3.7614	50.3	10.23	<.0001
115	Spinach chopped	GameVisit	3		25.5002	3.7614	50.3	6.78	<.0001
116	Spinach chopped	GameVisit*Group	1	Vegetable	33.2848	5.1642	50.3	6.45	<.0001
117	Spinach chopped	GameVisit*Group	1	Control	34.2157	5.1786	53.3	6.61	<.0001
118	Spinach chopped	GameVisit*Group	2	Vegetable	36.3959	5.1642	50.3	7.05	<.0001
119	Spinach chopped	GameVisit*Group	2	Control	40.5282	5.1786	53.3	7.83	<.0001
120	Spinach chopped	GameVisit*Group	3	Vegetable	22.2848	5.1642	50.3	4.32	<.0001
121	Spinach chopped	GameVisit*Group	3	Control	28.7157	5.1786	53.3	5.55	<.0001
122	Spinach puree	Group	_	Vegetable	34.1886	5.8475	31	5.85	<.0001
123	Spinach puree	Group	_	Control	35.6974	5.7582	31	6.20	<.0001
124	Spinach puree	GameVisit	1		33.2323	4.8162	48.9	6.90	<.0001
125	Spinach puree	GameVisit	2		38.0622	4.8162	48.9	7.90	<.0001
126	Spinach puree	GameVisit	3		33.5344	4.8162	48.9	6.96	<.0001
127	Spinach puree	GameVisit*Group	1	Vegetable	34.2256	6.6124	48.8	5.18	<.0001
128	Spinach puree	GameVisit*Group	1	Control	32.2390	6.6241	51.7	4.87	<.0001
129	Spinach puree	GameVisit*Group	2	Vegetable	34.9479	6.6124	48.8	5.29	<.0001
130	Spinach puree	GameVisit*Group	2	Control	41.1765	6.6241	51.7	6.22	<.0001
131	Spinach puree	GameVisit*Group	3	Vegetable	33.3923	6.6124	48.8	5.05	<.0001
132	Spinach puree	GameVisit*Group	3	Control	33.6765	6.6241	51.7	5.08	<.0001

Secondary analysis: Differences between vegetables, and from 0

Obs	GameVisit	Effect	NumDF	DenDF	FValue	ProbF
1	1	SampleName	11	352	4.03	<.0001
2	1	Group	1	31	0.75	0.3935
3	1	Group*SampleName	11	352	0.96	0.4822
4	1	Gender	1	31	0.20	0.6618
5	2	SampleName	11	352	4.45	<.0001
6	2	Group	1	31	0.28	0.5997
7	2	Group*SampleName	11	352	1.12	0.3466
8	2	Gender	1	31	0.07	0.7954
9	3	SampleName	11	352	3.89	<.0001
10	3	Group	1	31	0.01	0.9336
11	3	Group*SampleName	11	352	1.49	0.1329
12	3	Gender	1	31	0.14	0.7103

Secondary analysis: Differences between vegetables, and from 0

Obs	GameVisit	Effect	Group	SampleName	Estimate	StdErr	DF	tValue	Probt
1	1	Group	Vegetable		-8.1429	6.0831	31	-1.34	0.1904
2	1	Group	Control		-1.0455	5.9902	31	-0.17	0.8626
3	1	SampleName		Asparagus chopped	7.0910	7.2788	180	0.97	0.3313
4	1	SampleName		Asparagus puree	4.0771	7.2788	180	0.56	0.5761
5	1	SampleName		Beef puree	-14.9958	7.2788	180	-2.06	0.0408
6	1	SampleName		Black bean puree	-5.1347	7.2788	180	-0.71	0.4814
7	1	SampleName		Broccoli chopped	14.9521	7.2788	180	2.05	0.0414
8	1	SampleName		Broccoli puree	13.9556	7.2788	180	1.92	0.0568
9	1	SampleName		Chicken puree	-16.7007	7.2788	180	-2.29	0.0229
10	1	SampleName		Kale chopped	-22.3951	7.2788	180	-3.08	0.0024
11	1	SampleName		Kale puree	-11.2319	7.2788	180	-1.54	0.1246
12	1	SampleName		Oat puree	-11.8986	7.2788	180	-1.63	0.1039
13	1	SampleName		Spinach chopped	-5.7840	7.2788	180	-0.79	0.4279
14	1	SampleName		Spinach puree	-7.0653	7.2788	180	-0.97	0.3330
15	1	Group*SampleName	Vegetable	Asparagus chopped	-3.5642	9.9897	180	-0.36	0.7217
16	1	Group*SampleName	Vegetable	Asparagus puree	-3.8420	9.9897	180	-0.38	0.7010
17	1	Group*SampleName	Vegetable	Beef puree	-22.6753	9.9897	180	-2.27	0.0244
18	1	Group*SampleName	Vegetable	Black bean puree	-10.4531	9.9897	180	-1.05	0.2968
19	1	Group*SampleName	Vegetable	Broccoli chopped	14.1580	9.9897	180	1.42	0.1581
20	1	Group*SampleName	Vegetable	Broccoli puree	18.1025	9.9897	180	1.81	0.0716
21	1	Group*SampleName	Vegetable	Chicken puree	-25.8975	9.9897	180	-2.59	0.0103
22	1	Group*SampleName	Vegetable	Kale chopped	-30.7864	9.9897	180	-3.08	0.0024
23	1	Group*SampleName	Vegetable	Kale puree	-16.8975	9.9897	180	-1.69	0.0925
24	1	Group*SampleName	Vegetable	Oat puree	-3.7308	9.9897	180	-0.37	0.7092
25	1	Group*SampleName	Vegetable	Spinach chopped	-5.0642	9.9897	180	-0.51	0.6128
26	1	Group*SampleName	Vegetable	Spinach puree	-7.0642	9.9897	180	-0.71	0.4804
27	1	Group*SampleName	Control	Asparagus chopped	17.7461	10.3209	204	1.72	0.0871

Obs	GameVisit	Effect	Group	SampleName	Estimate	StdErr	DF	tValue	Probt
28	1	Group*SampleName	Control	Asparagus puree	11.9961	10.3209	204	1.16	0.2465
29	1	Group*SampleName	Control	Beef puree	-7.3164	10.3209	204	-0.71	0.4792
30	1	Group*SampleName	Control	Black bean puree	0.1836	10.3209	204	0.02	0.9858
31	1	Group*SampleName	Control	Broccoli chopped	15.7461	10.3209	204	1.53	0.1286
32	1	Group*SampleName	Control	Broccoli puree	9.8086	10.3209	204	0.95	0.3431
33	1	Group*SampleName	Control	Chicken puree	-7.5039	10.3209	204	-0.73	0.4680
34	1	Group*SampleName	Control	Kale chopped	-14.0039	10.3209	204	-1.36	0.1763
35	1	Group*SampleName	Control	Kale puree	-5.5664	10.3209	204	-0.54	0.5902
36	1	Group*SampleName	Control	Oat puree	-20.0664	10.3209	204	-1.94	0.0532
37	1	Group*SampleName	Control	Spinach chopped	-6.5039	10.3209	204	-0.63	0.5293
38	1	Group*SampleName	Control	Spinach puree	-7.0664	10.3209	204	-0.68	0.4943
39	2	Group	Vegetable		-5.4244	5.8384	31	-0.93	0.3600
40	2	Group	Control		-1.2493	5.7492	31	-0.22	0.8294
41	2	SampleName		Asparagus chopped	11.5237	7.0632	186	1.63	0.1045
42	2	SampleName		Asparagus puree	7.3466	7.0632	186	1.04	0.2996
43	2	SampleName		Beef puree	-16.3305	7.0632	186	-2.31	0.0219
44	2	SampleName		Black bean puree	1.9889	7.0632	186	0.28	0.7786
45	2	SampleName		Broccoli chopped	18.4542	7.0632	186	2.61	0.0097
46	2	SampleName		Broccoli puree	8.6000	7.0632	186	1.22	0.2249
47	2	SampleName		Chicken puree	-9.2993	7.0632	186	-1.32	0.1896
48	2	SampleName		Kale chopped	-17.5250	7.0632	186	-2.48	0.0140
49	2	SampleName		Kale puree	-17.5631	7.0632	186	-2.49	0.0138
50	2	SampleName		Oat puree	-8.0701	7.0632	186	-1.14	0.2547
51	2	SampleName		Spinach chopped	-7.5666	7.0632	186	-1.07	0.2854
52	2	SampleName		Spinach puree	-11.6013	7.0632	186	-1.64	0.1022
53	2	Group*SampleName	Vegetable	Asparagus chopped	11.0154	9.6937	185	1.14	0.2573
54	2	Group*SampleName	Vegetable	Asparagus puree	-2.1513	9.6937	185	-0.22	0.8246
55	2	Group*SampleName	Vegetable	Beef puree	-27.8179	9.6937	185	-2.87	0.0046
56	2	Group*SampleName	Vegetable	Black bean puree	-6.9291	9.6937	185	-0.71	0.4756
57	2	Group*SampleName	Vegetable	Broccoli chopped	13.1265	9.6937	185	1.35	0.1773
58	2	Group*SampleName	Vegetable	Broccoli puree	13.7932	9.6937	185	1.42	0.1564
59	2	Group*SampleName	Vegetable	Chicken puree	-17.3179	9.6937	185	-1.79	0.0757
60	2	Group*SampleName	Vegetable	Kale chopped	-13.7068	9.6937	185	-1.41	0.1590
61	2	Group*SampleName	Vegetable	Kale puree	-14.5957	9.6937	185	-1.51	0.1339
62	2	Group*SampleName	Vegetable	Oat puree	-2.4846	9.6937	185	-0.26	0.7980
63	2	Group*SampleName	Vegetable	Spinach chopped	-6.0402	9.6937	185	-0.62	0.5340
64	2	Group*SampleName	Vegetable	Spinach puree	-11.9846	9.6937	185	-1.24	0.2179
65	2	Group*SampleName	Control	Asparagus chopped	12.0319	10.0210	209	1.20	0.2312
66	2	Group*SampleName	Control	Asparagus puree	16.8444	10.0210	209	1.68	0.0943
67	2	Group*SampleName	Control	Beef puree	-4.8431	10.0210	209	-0.48	0.6294
68	2	Group*SampleName	Control	Black bean puree	10.9069	10.0210	209	1.09	0.2777
69	2	Group*SampleName	Control	Broccoli chopped	23.7819	10.0210	209	2.37	0.0185
70	2	Group*SampleName	Control	Broccoli puree	3.4069	10.0210	209	0.34	0.7342

Obs	GameVisit	Effect	Group	SampleName	Estimate	StdErr	DF	tValue	Probt
71	2	Group*SampleName	Control	Chicken puree	-1.2806	10.0210	209	-0.13	0.8984
72	2	Group*SampleName	Control	Kale chopped	-21.3431	10.0210	209	-2.13	0.0344
73	2	Group*SampleName	Control	Kale puree	-20.5306	10.0210	209	-2.05	0.0417
74	2	Group*SampleName	Control	Oat puree	-13.6556	10.0210	209	-1.36	0.1744
75	2	Group*SampleName	Control	Spinach chopped	-9.0931	10.0210	209	-0.91	0.3652
76	2	Group*SampleName	Control	Spinach puree	-11.2181	10.0210	209	-1.12	0.2642
77	3	Group	Vegetable		-0.3531	6.9139	31	-0.05	0.9596
78	3	Group	Control		-1.1360	6.8083	31	-0.17	0.8686
79	3	SampleName		Asparagus chopped	6.3093	7.3713	128	0.86	0.3936
80	3	SampleName		Asparagus puree	7.1912	7.3713	128	0.98	0.3311
81	3	SampleName		Beef puree	-7.6213	7.3713	128	-1.03	0.3031
82	3	SampleName		Black bean puree	2.5142	7.3713	128	0.34	0.7336
83	3	SampleName		Broccoli chopped	15.7190	7.3713	128	2.13	0.0349
84	3	SampleName		Broccoli puree	15.1253	7.3713	128	2.05	0.0422
85	3	SampleName		Chicken puree	-8.0067	7.3713	128	-1.09	0.2794
86	3	SampleName		Kale chopped	-17.3053	7.3713	128	-2.35	0.0204
87	3	SampleName		Kale puree	-15.6004	7.3713	128	-2.12	0.0363
88	3	SampleName		Oat puree	2.4204	7.3713	128	0.33	0.7432
89	3	SampleName		Spinach chopped	1.0211	7.3713	128	0.14	0.8900
90	3	SampleName		Spinach puree	-10.7011	7.3713	128	-1.45	0.1490
91	3	Group*SampleName	Vegetable	Asparagus chopped	-0.5475	10.1175	128	-0.05	0.9569
92	3	Group*SampleName	Vegetable	Asparagus puree	3.3414	10.1175	128	0.33	0.7417
93	3	Group*SampleName	Vegetable	Beef puree	-15.1586	10.1175	128	-1.50	0.1365
94	3	Group*SampleName	Vegetable	Black bean puree	-3.8253	10.1175	128	-0.38	0.7060
95	3	Group*SampleName	Vegetable	Broccoli chopped	12.3969	10.1175	128	1.23	0.2227
96	3	Group*SampleName	Vegetable	Broccoli puree	18.3969	10.1175	128	1.82	0.0714
97	3	Group*SampleName	Vegetable	Chicken puree	-14.9920	10.1175	128	-1.48	0.1409
98	3	Group*SampleName	Vegetable	Kale chopped	-11.2142	10.1175	128	-1.11	0.2698
99	3	Group*SampleName	Vegetable	Kale puree	-6.4920	10.1175	128	-0.64	0.5222
100	3	Group*SampleName	Vegetable	Oat puree	13.1747	10.1175	128	1.30	0.1952
101	3	Group*SampleName	Vegetable	Spinach chopped	7.0636	10.1175	128	0.70	0.4864
102	3	Group*SampleName	Vegetable	Spinach puree	-6.3808	10.1175	128	-0.63	0.5294
103	3	Group*SampleName	Control	Asparagus chopped	13.1661	10.3796	145	1.27	0.2067
104	3	Group*SampleName	Control	Asparagus puree	11.0411	10.3796	145	1.06	0.2892
105	3	Group*SampleName	Control	Beef puree	-0.08388	10.3796	145	-0.01	0.9936
106	3	Group*SampleName	Control	Black bean puree	8.8536	10.3796	145	0.85	0.3951
107	3	Group*SampleName	Control	Broccoli chopped	19.0411	10.3796	145	1.83	0.0686
108	3	Group*SampleName	Control	Broccoli puree	11.8536	10.3796	145	1.14	0.2553
109	3	Group*SampleName	Control	Chicken puree	-1.0214	10.3796	145	-0.10	0.9217
110	3	Group*SampleName	Control	Kale chopped	-23.3964	10.3796	145	-2.25	0.0257
111	3	Group*SampleName	Control	Kale puree	-24.7089	10.3796	145	-2.38	0.0186
112	3	Group*SampleName	Control	Oat puree	-8.3339	10.3796	145	-0.80	0.4233
113	3	Group*SampleName	Control	Spinach chopped	-5.0214	10.3796	145	-0.48	0.6293

Obs	GameVisit	Effect	Group	SampleName	Estimate	StdErr	DF	tValue	Probt
114	3	Group*SampleName	Control	Spinach puree	-15.0214	10.3796	145	-1.45	0.1500

Secondary analysis: Differences between vegetables, and from 0

Obs	GameVisit	Effect	Group	SampleName	_Group	_SampleName	Estimate	StdErr	DF	tValue	Probt
1	1	Group	Vegetable		Control		-7.0973	8.2025	31	-0.87	0.3935
2	1	SampleName		Asparagus chopped		Asparagus puree	3.0139	8.5310	352	0.35	0.7241
3	1	SampleName		Asparagus chopped		Beef puree	22.0868	8.5310	352	2.59	0.0100
4	1	SampleName		Asparagus chopped		Black bean puree	12.2257	8.5310	352	1.43	0.1527
5	1	SampleName		Asparagus chopped		Broccoli chopped	-7.8611	8.5310	352	-0.92	0.3574
6	1	SampleName		Asparagus chopped		Broccoli puree	-6.8646	8.5310	352	-0.80	0.4216
7	1	SampleName		Asparagus chopped		Chicken puree	23.7917	8.5310	352	2.79	0.0056
8	1	SampleName		Asparagus chopped		Kale chopped	29.4861	8.5310	352	3.46	0.0006
9	1	SampleName		Asparagus chopped		Kale puree	18.3229	8.5310	352	2.15	0.0324
10	1	SampleName		Asparagus chopped		Oat puree	18.9896	8.5310	352	2.23	0.0267
11	1	SampleName		Asparagus chopped		Spinach chopped	12.8750	8.5310	352	1.51	0.1321
12	1	SampleName		Asparagus chopped		Spinach puree	14.1562	8.5310	352	1.66	0.0979
13	1	SampleName		Asparagus puree		Beef puree	19.0729	8.5310	352	2.24	0.0260
14	1	SampleName		Asparagus puree		Black bean puree	9.2118	8.5310	352	1.08	0.2810
15	1	SampleName		Asparagus puree		Broccoli chopped	-10.8750	8.5310	352	-1.27	0.2032
16	1	SampleName		Asparagus puree		Broccoli puree	-9.8785	8.5310	352	-1.16	0.2477
17	1	SampleName		Asparagus puree		Chicken puree	20.7778	8.5310	352	2.44	0.0154
18	1	SampleName		Asparagus puree		Kale chopped	26.4722	8.5310	352	3.10	0.0021
19	1	SampleName		Asparagus puree		Kale puree	15.3090	8.5310	352	1.79	0.0736
20	1	SampleName		Asparagus puree		Oat puree	15.9757	8.5310	352	1.87	0.0619
21	1	SampleName		Asparagus puree		Spinach chopped	9.8611	8.5310	352	1.16	0.2485
22	1	SampleName		Asparagus puree		Spinach puree	11.1424	8.5310	352	1.31	0.1924
23	1	SampleName		Beef puree		Black bean puree	-9.8611	8.5310	352	-1.16	0.2485
24	1	SampleName		Beef puree		Broccoli chopped	-29.9479	8.5310	352	-3.51	0.0005
25	1	SampleName		Beef puree		Broccoli puree	-28.9514	8.5310	352	-3.39	0.0008
26	1	SampleName		Beef puree		Chicken puree	1.7049	8.5310	352	0.20	0.8417

Obs	GameVisit	Effect	Group	SampleName	_Group	_SampleName	Estimate	StdErr	DF	tValue	Probt
27	1	SampleName		Beef puree		Kale chopped	7.3993	8.5310	352	0.87	0.3863
28	1	SampleName		Beef puree		Kale puree	-3.7639	8.5310	352	-0.44	0.6593
29	1	SampleName		Beef puree		Oat puree	-3.0972	8.5310	352	-0.36	0.7168
30	1	SampleName		Beef puree		Spinach chopped	-9.2118	8.5310	352	-1.08	0.2810
31	1	SampleName		Beef puree		Spinach puree	-7.9306	8.5310	352	-0.93	0.3532
32	1	SampleName		Black bean puree		Broccoli chopped	-20.0868	8.5310	352	-2.35	0.0191
33	1	SampleName		Black bean puree		Broccoli puree	-19.0903	8.5310	352	-2.24	0.0259
34	1	SampleName		Black bean puree		Chicken puree	11.5660	8.5310	352	1.36	0.1760
35	1	SampleName		Black bean puree		Kale chopped	17.2604	8.5310	352	2.02	0.0438
36	1	SampleName		Black bean puree		Kale puree	6.0972	8.5310	352	0.71	0.4753
37	1	SampleName		Black bean puree		Oat puree	6.7639	8.5310	352	0.79	0.4284
38	1	SampleName		Black bean puree		Spinach chopped	0.6493	8.5310	352	0.08	0.9394
39	1	SampleName		Black bean puree		Spinach puree	1.9306	8.5310	352	0.23	0.8211
40	1	SampleName		Broccoli chopped		Broccoli puree	0.9965	8.5310	352	0.12	0.9071
41	1	SampleName		Broccoli chopped		Chicken puree	31.6528	8.5310	352	3.71	0.0002
42	1	SampleName		Broccoli chopped		Kale chopped	37.3472	8.5310	352	4.38	<.0001
43	1	SampleName		Broccoli chopped		Kale puree	26.1840	8.5310	352	3.07	0.0023
44	1	SampleName		Broccoli chopped		Oat puree	26.8507	8.5310	352	3.15	0.0018
45	1	SampleName		Broccoli chopped		Spinach chopped	20.7361	8.5310	352	2.43	0.0156
46	1	SampleName		Broccoli chopped		Spinach puree	22.0174	8.5310	352	2.58	0.0103
47	1	SampleName		Broccoli puree		Chicken puree	30.6563	8.5310	352	3.59	0.0004
48	1	SampleName		Broccoli puree		Kale chopped	36.3507	8.5310	352	4.26	<.0001
49	1	SampleName		Broccoli puree		Kale puree	25.1875	8.5310	352	2.95	0.0034
50	1	SampleName		Broccoli puree		Oat puree	25.8542	8.5310	352	3.03	0.0026
51	1	SampleName		Broccoli puree		Spinach chopped	19.7396	8.5310	352	2.31	0.0213
52	1	SampleName		Broccoli puree		Spinach puree	21.0208	8.5310	352	2.46	0.0142
53	1	SampleName		Chicken puree		Kale chopped	5.6944	8.5310	352	0.67	0.5049
54	1	SampleName		Chicken puree		Kale puree	-5.4688	8.5310	352	-0.64	0.5219
55	1	SampleName		Chicken puree		Oat puree	-4.8021	8.5310	352	-0.56	0.5739
56	1	SampleName		Chicken puree		Spinach chopped	-10.9167	8.5310	352	-1.28	0.2015

Obs	GameVisit	Effect	Group	SampleName	_Group	_SampleName	Estimate	StdErr	DF	tValue	Probt
57	1	SampleName		Chicken puree		Spinach puree	-9.6354	8.5310	352	-1.13	0.2595
58	1	SampleName		Kale chopped		Kale puree	-11.1632	8.5310	352	-1.31	0.1915
59	1	SampleName		Kale chopped		Oat puree	-10.4965	8.5310	352	-1.23	0.2194
60	1	SampleName		Kale chopped		Spinach chopped	-16.6111	8.5310	352	-1.95	0.0523
61	1	SampleName		Kale chopped		Spinach puree	-15.3299	8.5310	352	-1.80	0.0732
62	1	SampleName		Kale puree		Oat puree	0.6667	8.5310	352	0.08	0.9378
63	1	SampleName		Kale puree		Spinach chopped	-5.4479	8.5310	352	-0.64	0.5235
64	1	SampleName		Kale puree		Spinach puree	-4.1667	8.5310	352	-0.49	0.6256
65	1	SampleName		Oat puree		Spinach chopped	-6.1146	8.5310	352	-0.72	0.4740
66	1	SampleName		Oat puree		Spinach puree	-4.8333	8.5310	352	-0.57	0.5714
67	1	SampleName		Spinach chopped		Spinach puree	1.2812	8.5310	352	0.15	0.8807
68	1	Group*SampleName	Vegetable	Asparagus chopped	Vegetable	Asparagus puree	0.2778	11.7045	352	0.02	0.9811
69	1	Group*SampleName	Vegetable	Asparagus chopped	Vegetable	Beef puree	19.1111	11.7045	352	1.63	0.1034
70	1	Group*SampleName	Vegetable	Asparagus chopped	Vegetable	Black bean puree	6.8889	11.7045	352	0.59	0.5565
71	1	Group*SampleName	Vegetable	Asparagus chopped	Vegetable	Broccoli chopped	-17.7222	11.7045	352	-1.51	0.1309
72	1	Group*SampleName	Vegetable	Asparagus chopped	Vegetable	Broccoli puree	-21.6667	11.7045	352	-1.85	0.0650
73	1	Group*SampleName	Vegetable	Asparagus chopped	Vegetable	Chicken puree	22.3333	11.7045	352	1.91	0.0572
74	1	Group*SampleName	Vegetable	Asparagus chopped	Vegetable	Kale chopped	27.2222	11.7045	352	2.33	0.0206
75	1	Group*SampleName	Vegetable	Asparagus chopped	Vegetable	Kale puree	13.3333	11.7045	352	1.14	0.2554
76	1	Group*SampleName	Vegetable	Asparagus chopped	Vegetable	Oat puree	0.1667	11.7045	352	0.01	0.9886
77	1	Group*SampleName	Vegetable	Asparagus chopped	Vegetable	Spinach chopped	1.5000	11.7045	352	0.13	0.8981
78	1	Group*SampleName	Vegetable	Asparagus chopped	Vegetable	Spinach puree	3.5000	11.7045	352	0.30	0.7651
79	1	Group*SampleName	Vegetable	Asparagus chopped	Control	Asparagus chopped	-21.3103	14.1671	205	-1.50	0.1341
80	1	Group*SampleName	Vegetable	Asparagus chopped	Control	Asparagus puree	-15.5603	14.1671	205	-1.10	0.2733
81	1	Group*SampleName	Vegetable	Asparagus chopped	Control	Beef puree	3.7522	14.1671	205	0.26	0.7914
82	1	Group*SampleName	Vegetable	Asparagus chopped	Control	Black bean puree	-3.7478	14.1671	205	-0.26	0.7916
83	1	Group*SampleName	Vegetable	Asparagus chopped	Control	Broccoli chopped	-19.3103	14.1671	205	-1.36	0.1744
84	1	Group*SampleName	Vegetable	Asparagus chopped	Control	Broccoli puree	-13.3728	14.1671	205	-0.94	0.3463
85	1	Group*SampleName	Vegetable	Asparagus chopped	Control	Chicken puree	3.9397	14.1671	205	0.28	0.7812
86	1	Group*SampleName	Vegetable	Asparagus chopped	Control	Kale chopped	10.4397	14.1671	205	0.74	0.4620
87	1	Group*SampleName	Vegetable	Asparagus	Control	Kale puree	2.0022	14.1671	205	0.14	0.8878

Obs	GameVisit	Effect	Group	SampleName	_Group	_SampleName	Estimate	StdErr	DF	tValue	Probt
				chopped							
88	1	Group*SampleName	Vegetable	Asparagus chopped	Control	Oat puree	16.5022	14.1671	205	1.16	0.2454
89	1	Group*SampleName	Vegetable	Asparagus chopped	Control	Spinach chopped	2.9397	14.1671	205	0.21	0.8358
90	1	Group*SampleName	Vegetable	Asparagus chopped	Control	Spinach puree	3.5022	14.1671	205	0.25	0.8050
91	1	Group*SampleName	Vegetable	Asparagus puree	Vegetable	Beef puree	18.8333	11.7045	352	1.61	0.1085
92	1	Group*SampleName	Vegetable	Asparagus puree	Vegetable	Black bean puree	6.6111	11.7045	352	0.56	0.5725
93	1	Group*SampleName	Vegetable	Asparagus puree	Vegetable	Broccoli chopped	-18.0000	11.7045	352	-1.54	0.1250
94	1	Group*SampleName	Vegetable	Asparagus puree	Vegetable	Broccoli puree	-21.9444	11.7045	352	-1.87	0.0616
95	1	Group*SampleName	Vegetable	Asparagus puree	Vegetable	Chicken puree	22.0556	11.7045	352	1.88	0.0603
96	1	Group*SampleName	Vegetable	Asparagus puree	Vegetable	Kale chopped	26.9444	11.7045	352	2.30	0.0219
97	1	Group*SampleName	Vegetable	Asparagus puree	Vegetable	Kale puree	13.0556	11.7045	352	1.12	0.2654
98	1	Group*SampleName	Vegetable	Asparagus puree	Vegetable	Oat puree	-0.1111	11.7045	352	-0.01	0.9924
99	1	Group*SampleName	Vegetable	Asparagus puree	Vegetable	Spinach chopped	1.2222	11.7045	352	0.10	0.9169
100	1	Group*SampleName	Vegetable	Asparagus puree	Vegetable	Spinach puree	3.2222	11.7045	352	0.28	0.7832
101	1	Group*SampleName	Vegetable	Asparagus puree	Control	Asparagus chopped	-21.5881	14.1671	205	-1.52	0.1291
102	1	Group*SampleName	Vegetable	Asparagus puree	Control	Asparagus puree	-15.8381	14.1671	205	-1.12	0.2649
103	1	Group*SampleName	Vegetable	Asparagus puree	Control	Beef puree	3.4744	14.1671	205	0.25	0.8065
104	1	Group*SampleName	Vegetable	Asparagus puree	Control	Black bean puree	-4.0256	14.1671	205	-0.28	0.7766
105	1	Group*SampleName	Vegetable	Asparagus puree	Control	Broccoli chopped	-19.5881	14.1671	205	-1.38	0.1683
106	1	Group*SampleName	Vegetable	Asparagus puree	Control	Broccoli puree	-13.6506	14.1671	205	-0.96	0.3364
107	1	Group*SampleName	Vegetable	Asparagus puree	Control	Chicken puree	3.6619	14.1671	205	0.26	0.7963
108	1	Group*SampleName	Vegetable	Asparagus puree	Control	Kale chopped	10.1619	14.1671	205	0.72	0.4740
109	1	Group*SampleName	Vegetable	Asparagus puree	Control	Kale puree	1.7244	14.1671	205	0.12	0.9032
110	1	Group*SampleName	Vegetable	Asparagus puree	Control	Oat puree	16.2244	14.1671	205	1.15	0.2535
111	1	Group*SampleName	Vegetable	Asparagus puree	Control	Spinach chopped	2.6619	14.1671	205	0.19	0.8511
112	1	Group*SampleName	Vegetable	Asparagus puree	Control	Spinach puree	3.2244	14.1671	205	0.23	0.8202
113	1	Group*SampleName	Vegetable	Beef puree	Vegetable	Black bean puree	-12.2222	11.7045	352	-1.04	0.2971
114	1	Group*SampleName	Vegetable	Beef puree	Vegetable	Broccoli chopped	-36.8333	11.7045	352	-3.15	0.0018
115	1	Group*SampleName	Vegetable	Beef puree	Vegetable	Broccoli puree	-40.7778	11.7045	352	-3.48	0.0006
116	1	Group*SampleName	Vegetable	Beef puree	Vegetable	Chicken puree	3.2222	11.7045	352	0.28	0.7832

Obs	GameVisit	Effect	Group	SampleName	_Group	_SampleName	Estimate	StdErr	DF	tValue	Probt
117	1	Group*SampleName	Vegetable	Beef puree	Vegetable	Kale chopped	8.1111	11.7045	352	0.69	0.4888
118	1	Group*SampleName	Vegetable	Beef puree	Vegetable	Kale puree	-5.7778	11.7045	352	-0.49	0.6219
119	1	Group*SampleName	Vegetable	Beef puree	Vegetable	Oat puree	-18.9444	11.7045	352	-1.62	0.1064
120	1	Group*SampleName	Vegetable	Beef puree	Vegetable	Spinach chopped	-17.6111	11.7045	352	-1.50	0.1333
121	1	Group*SampleName	Vegetable	Beef puree	Vegetable	Spinach puree	-15.6111	11.7045	352	-1.33	0.1831
122	1	Group*SampleName	Vegetable	Beef puree	Control	Asparagus chopped	-40.4214	14.1671	205	-2.85	0.0048
123	1	Group*SampleName	Vegetable	Beef puree	Control	Asparagus puree	-34.6714	14.1671	205	-2.45	0.0152
124	1	Group*SampleName	Vegetable	Beef puree	Control	Beef puree	-15.3589	14.1671	205	-1.08	0.2796
125	1	Group*SampleName	Vegetable	Beef puree	Control	Black bean puree	-22.8589	14.1671	205	-1.61	0.1082
126	1	Group*SampleName	Vegetable	Beef puree	Control	Broccoli chopped	-38.4214	14.1671	205	-2.71	0.0073
127	1	Group*SampleName	Vegetable	Beef puree	Control	Broccoli puree	-32.4839	14.1671	205	-2.29	0.0229
128	1	Group*SampleName	Vegetable	Beef puree	Control	Chicken puree	-15.1714	14.1671	205	-1.07	0.2855
129	1	Group*SampleName	Vegetable	Beef puree	Control	Kale chopped	-8.6714	14.1671	205	-0.61	0.5412
130	1	Group*SampleName	Vegetable	Beef puree	Control	Kale puree	-17.1089	14.1671	205	-1.21	0.2286
131	1	Group*SampleName	Vegetable	Beef puree	Control	Oat puree	-2.6089	14.1671	205	-0.18	0.8541
132	1	Group*SampleName	Vegetable	Beef puree	Control	Spinach chopped	-16.1714	14.1671	205	-1.14	0.2550
133	1	Group*SampleName	Vegetable	Beef puree	Control	Spinach puree	-15.6089	14.1671	205	-1.10	0.2719
134	1	Group*SampleName	Vegetable	Black bean puree	Vegetable	Broccoli chopped	-24.6111	11.7045	352	-2.10	0.0362
135	1	Group*SampleName	Vegetable	Black bean puree	Vegetable	Broccoli puree	-28.5556	11.7045	352	-2.44	0.0152
136	1	Group*SampleName	Vegetable	Black bean puree	Vegetable	Chicken puree	15.4444	11.7045	352	1.32	0.1878
137	1	Group*SampleName	Vegetable	Black bean puree	Vegetable	Kale chopped	20.3333	11.7045	352	1.74	0.0832
138	1	Group*SampleName	Vegetable	Black bean puree	Vegetable	Kale puree	6.4444	11.7045	352	0.55	0.5823
139	1	Group*SampleName	Vegetable	Black bean puree	Vegetable	Oat puree	-6.7222	11.7045	352	-0.57	0.5661
140	1	Group*SampleName	Vegetable	Black bean puree	Vegetable	Spinach chopped	-5.3889	11.7045	352	-0.46	0.6455
141	1	Group*SampleName	Vegetable	Black bean puree	Vegetable	Spinach puree	-3.3889	11.7045	352	-0.29	0.7723
142	1	Group*SampleName	Vegetable	Black bean puree	Control	Asparagus chopped	-28.1992	14.1671	205	-1.99	0.0479
143	1	Group*SampleName	Vegetable	Black bean puree	Control	Asparagus puree	-22.4492	14.1671	205	-1.58	0.1146
144	1	Group*SampleName	Vegetable	Black bean puree	Control	Beef puree	-3.1367	14.1671	205	-0.22	0.8250
145	1	Group*SampleName	Vegetable	Black bean puree	Control	Black bean puree	-10.6367	14.1671	205	-0.75	0.4536
146	1	Group*SampleName	Vegetable	Black bean puree	Control	Broccoli chopped	-26.1992	14.1671	205	-1.85	0.0659
147	1	Group*SampleName	Vegetable	Black bean puree	Control	Broccoli puree	-20.2617	14.1671	205	-1.43	0.1542
148	1	Group*SampleName	Vegetable	Black bean puree	Control	Chicken puree	-2.9492	14.1671	205	-0.21	0.8353

Obs	GameVisit	Effect	Group	SampleName	_Group	_SampleName	Estimate	StdErr	DF	tValue	Probt
149	1	Group*SampleName	Vegetable	Black bean puree	Control	Kale chopped	3.5508	14.1671	205	0.25	0.8023
150	1	Group*SampleName	Vegetable	Black bean puree	Control	Kale puree	-4.8867	14.1671	205	-0.34	0.7305
151	1	Group*SampleName	Vegetable	Black bean puree	Control	Oat puree	9.6133	14.1671	205	0.68	0.4982
152	1	Group*SampleName	Vegetable	Black bean puree	Control	Spinach chopped	-3.9492	14.1671	205	-0.28	0.7807
153	1	Group*SampleName	Vegetable	Black bean puree	Control	Spinach puree	-3.3867	14.1671	205	-0.24	0.8113
154	1	Group*SampleName	Vegetable	Broccoli chopped	Vegetable	Broccoli puree	-3.9444	11.7045	352	-0.34	0.7363
155	1	Group*SampleName	Vegetable	Broccoli chopped	Vegetable	Chicken puree	40.0556	11.7045	352	3.42	0.0007
156	1	Group*SampleName	Vegetable	Broccoli chopped	Vegetable	Kale chopped	44.9444	11.7045	352	3.84	0.0001
157	1	Group*SampleName	Vegetable	Broccoli chopped	Vegetable	Kale puree	31.0556	11.7045	352	2.65	0.0083
158	1	Group*SampleName	Vegetable	Broccoli chopped	Vegetable	Oat puree	17.8889	11.7045	352	1.53	0.1273
159	1	Group*SampleName	Vegetable	Broccoli chopped	Vegetable	Spinach chopped	19.2222	11.7045	352	1.64	0.1014
160	1	Group*SampleName	Vegetable	Broccoli chopped	Vegetable	Spinach puree	21.2222	11.7045	352	1.81	0.0707
161	1	Group*SampleName	Vegetable	Broccoli chopped	Control	Asparagus chopped	-3.5881	14.1671	205	-0.25	0.8003
162	1	Group*SampleName	Vegetable	Broccoli chopped	Control	Asparagus puree	2.1619	14.1671	205	0.15	0.8789
163	1	Group*SampleName	Vegetable	Broccoli chopped	Control	Beef puree	21.4744	14.1671	205	1.52	0.1311
164	1	Group*SampleName	Vegetable	Broccoli chopped	Control	Black bean puree	13.9744	14.1671	205	0.99	0.3251
165	1	Group*SampleName	Vegetable	Broccoli chopped	Control	Broccoli chopped	-1.5881	14.1671	205	-0.11	0.9109
166	1	Group*SampleName	Vegetable	Broccoli chopped	Control	Broccoli puree	4.3494	14.1671	205	0.31	0.7591
167	1	Group*SampleName	Vegetable	Broccoli chopped	Control	Chicken puree	21.6619	14.1671	205	1.53	0.1278
168	1	Group*SampleName	Vegetable	Broccoli chopped	Control	Kale chopped	28.1619	14.1671	205	1.99	0.0482
169	1	Group*SampleName	Vegetable	Broccoli chopped	Control	Kale puree	19.7244	14.1671	205	1.39	0.1654
170	1	Group*SampleName	Vegetable	Broccoli chopped	Control	Oat puree	34.2244	14.1671	205	2.42	0.0166
171	1	Group*SampleName	Vegetable	Broccoli chopped	Control	Spinach chopped	20.6619	14.1671	205	1.46	0.1463
172	1	Group*SampleName	Vegetable	Broccoli chopped	Control	Spinach puree	21.2244	14.1671	205	1.50	0.1356
173	1	Group*SampleName	Vegetable	Broccoli puree	Vegetable	Chicken puree	44.0000	11.7045	352	3.76	0.0002
174	1	Group*SampleName	Vegetable	Broccoli puree	Vegetable	Kale chopped	48.8889	11.7045	352	4.18	<.0001
175	1	Group*SampleName	Vegetable	Broccoli puree	Vegetable	Kale puree	35.0000	11.7045	352	2.99	0.0030
176	1	Group*SampleName	Vegetable	Broccoli puree	Vegetable	Oat puree	21.8333	11.7045	352	1.87	0.0630
177	1	Group*SampleName	Vegetable	Broccoli	Vegetable	Spinach	23.1667	11.7045	352	1.98	0.0486

Obs	GameVisit	Effect	Group	SampleName	_Group	_SampleName	Estimate	StdErr	DF	tValue	Probt
				puree		chopped					
178	1	Group*SampleName	Vegetable	Broccoli puree	Vegetable	Spinach puree	25.1667	11.7045	352	2.15	0.0322
179	1	Group*SampleName	Vegetable	Broccoli puree	Control	Asparagus chopped	0.3564	14.1671	205	0.03	0.9800
180	1	Group*SampleName	Vegetable	Broccoli puree	Control	Asparagus puree	6.1064	14.1671	205	0.43	0.6669
181	1	Group*SampleName	Vegetable	Broccoli puree	Control	Beef puree	25.4189	14.1671	205	1.79	0.0743
182	1	Group*SampleName	Vegetable	Broccoli puree	Control	Black bean puree	17.9189	14.1671	205	1.26	0.2074
183	1	Group*SampleName	Vegetable	Broccoli puree	Control	Broccoli chopped	2.3564	14.1671	205	0.17	0.8681
184	1	Group*SampleName	Vegetable	Broccoli puree	Control	Broccoli puree	8.2939	14.1671	205	0.59	0.5589
185	1	Group*SampleName	Vegetable	Broccoli puree	Control	Chicken puree	25.6064	14.1671	205	1.81	0.0722
186	1	Group*SampleName	Vegetable	Broccoli puree	Control	Kale chopped	32.1064	14.1671	205	2.27	0.0245
187	1	Group*SampleName	Vegetable	Broccoli puree	Control	Kale puree	23.6689	14.1671	205	1.67	0.0963
188	1	Group*SampleName	Vegetable	Broccoli puree	Control	Oat puree	38.1689	14.1671	205	2.69	0.0076
189	1	Group*SampleName	Vegetable	Broccoli puree	Control	Spinach chopped	24.6064	14.1671	205	1.74	0.0839
190	1	Group*SampleName	Vegetable	Broccoli puree	Control	Spinach puree	25.1689	14.1671	205	1.78	0.0771
191	1	Group*SampleName	Vegetable	Chicken puree	Vegetable	Kale chopped	4.8889	11.7045	352	0.42	0.6764
192	1	Group*SampleName	Vegetable	Chicken puree	Vegetable	Kale puree	-9.0000	11.7045	352	-0.77	0.4424
193	1	Group*SampleName	Vegetable	Chicken puree	Vegetable	Oat puree	-22.1667	11.7045	352	-1.89	0.0591
194	1	Group*SampleName	Vegetable	Chicken puree	Vegetable	Spinach chopped	-20.8333	11.7045	352	-1.78	0.0759
195	1	Group*SampleName	Vegetable	Chicken puree	Vegetable	Spinach puree	-18.8333	11.7045	352	-1.61	0.1085
196	1	Group*SampleName	Vegetable	Chicken puree	Control	Asparagus chopped	-43.6436	14.1671	205	-3.08	0.0023
197	1	Group*SampleName	Vegetable	Chicken puree	Control	Asparagus puree	-37.8936	14.1671	205	-2.67	0.0081
198	1	Group*SampleName	Vegetable	Chicken puree	Control	Beef puree	-18.5811	14.1671	205	-1.31	0.1911
199	1	Group*SampleName	Vegetable	Chicken puree	Control	Black bean puree	-26.0811	14.1671	205	-1.84	0.0671
200	1	Group*SampleName	Vegetable	Chicken puree	Control	Broccoli chopped	-41.6436	14.1671	205	-2.94	0.0037
201	1	Group*SampleName	Vegetable	Chicken puree	Control	Broccoli puree	-35.7061	14.1671	205	-2.52	0.0125
202	1	Group*SampleName	Vegetable	Chicken puree	Control	Chicken puree	-18.3936	14.1671	205	-1.30	0.1956
203	1	Group*SampleName	Vegetable	Chicken puree	Control	Kale chopped	-11.8936	14.1671	205	-0.84	0.4022
204	1	Group*SampleName	Vegetable	Chicken puree	Control	Kale puree	-20.3311	14.1671	205	-1.44	0.1528
205	1	Group*SampleName	Vegetable	Chicken puree	Control	Oat puree	-5.8311	14.1671	205	-0.41	0.6811

Obs	GameVisit	Effect	Group	SampleName	_Group	_SampleName	Estimate	StdErr	DF	tValue	Probt
206	1	Group*SampleName	Vegetable	Chicken puree	Control	Spinach chopped	-19.3936	14.1671	205	-1.37	0.1725
207	1	Group*SampleName	Vegetable	Chicken puree	Control	Spinach puree	-18.8311	14.1671	205	-1.33	0.1853
208	1	Group*SampleName	Vegetable	Kale chopped	Vegetable	Kale puree	-13.8889	11.7045	352	-1.19	0.2362
209	1	Group*SampleName	Vegetable	Kale chopped	Vegetable	Oat puree	-27.0556	11.7045	352	-2.31	0.0214
210	1	Group*SampleName	Vegetable	Kale chopped	Vegetable	Spinach chopped	-25.7222	11.7045	352	-2.20	0.0286
211	1	Group*SampleName	Vegetable	Kale chopped	Vegetable	Spinach puree	-23.7222	11.7045	352	-2.03	0.0434
212	1	Group*SampleName	Vegetable	Kale chopped	Control	Asparagus chopped	-48.5325	14.1671	205	-3.43	0.0007
213	1	Group*SampleName	Vegetable	Kale chopped	Control	Asparagus puree	-42.7825	14.1671	205	-3.02	0.0029
214	1	Group*SampleName	Vegetable	Kale chopped	Control	Beef puree	-23.4700	14.1671	205	-1.66	0.0991
215	1	Group*SampleName	Vegetable	Kale chopped	Control	Black bean puree	-30.9700	14.1671	205	-2.19	0.0299
216	1	Group*SampleName	Vegetable	Kale chopped	Control	Broccoli chopped	-46.5325	14.1671	205	-3.28	0.0012
217	1	Group*SampleName	Vegetable	Kale chopped	Control	Broccoli puree	-40.5950	14.1671	205	-2.87	0.0046
218	1	Group*SampleName	Vegetable	Kale chopped	Control	Chicken puree	-23.2825	14.1671	205	-1.64	0.1018
219	1	Group*SampleName	Vegetable	Kale chopped	Control	Kale chopped	-16.7825	14.1671	205	-1.18	0.2375
220	1	Group*SampleName	Vegetable	Kale chopped	Control	Kale puree	-25.2200	14.1671	205	-1.78	0.0765
221	1	Group*SampleName	Vegetable	Kale chopped	Control	Oat puree	-10.7200	14.1671	205	-0.76	0.4501
222	1	Group*SampleName	Vegetable	Kale chopped	Control	Spinach chopped	-24.2825	14.1671	205	-1.71	0.0880
223	1	Group*SampleName	Vegetable	Kale chopped	Control	Spinach puree	-23.7200	14.1671	205	-1.67	0.0956
224	1	Group*SampleName	Vegetable	Kale puree	Vegetable	Oat puree	-13.1667	11.7045	352	-1.12	0.2614
225	1	Group*SampleName	Vegetable	Kale puree	Vegetable	Spinach chopped	-11.8333	11.7045	352	-1.01	0.3127
226	1	Group*SampleName	Vegetable	Kale puree	Vegetable	Spinach puree	-9.8333	11.7045	352	-0.84	0.4014
227	1	Group*SampleName	Vegetable	Kale puree	Control	Asparagus chopped	-34.6436	14.1671	205	-2.45	0.0153
228	1	Group*SampleName	Vegetable	Kale puree	Control	Asparagus puree	-28.8936	14.1671	205	-2.04	0.0427
229	1	Group*SampleName	Vegetable	Kale puree	Control	Beef puree	-9.5811	14.1671	205	-0.68	0.4996
230	1	Group*SampleName	Vegetable	Kale puree	Control	Black bean puree	-17.0811	14.1671	205	-1.21	0.2293
231	1	Group*SampleName	Vegetable	Kale puree	Control	Broccoli chopped	-32.6436	14.1671	205	-2.30	0.0222
232	1	Group*SampleName	Vegetable	Kale puree	Control	Broccoli puree	-26.7061	14.1671	205	-1.89	0.0608
233	1	Group*SampleName	Vegetable	Kale puree	Control	Chicken puree	-9.3936	14.1671	205	-0.66	0.5080
234	1	Group*SampleName	Vegetable	Kale puree	Control	Kale chopped	-2.8936	14.1671	205	-0.20	0.8384
235	1	Group*SampleName	Vegetable	Kale puree	Control	Kale puree	-11.3311	14.1671	205	-0.80	0.4247
236	1	Group*SampleName	Vegetable	Kale puree	Control	Oat puree	3.1689	14.1671	205	0.22	0.8232
237	1	Group*SampleName	Vegetable	Kale puree	Control	Spinach chopped	-10.3936	14.1671	205	-0.73	0.4640
238	1	Group*SampleName	Vegetable	Kale puree	Control	Spinach puree	-9.8311	14.1671	205	-0.69	0.4885
239	1	Group*SampleName	Vegetable	Oat puree	Vegetable	Spinach chopped	1.3333	11.7045	352	0.11	0.9094
240	1	Group*SampleName	Vegetable	Oat puree	Vegetable	Spinach puree	3.3333	11.7045	352	0.28	0.7760

Obs	GameVisit	Effect	Group	SampleName	_Group	_SampleName	Estimate	StdErr	DF	tValue	Probt
241	1	Group*SampleName	Vegetable	Oat puree	Control	Asparagus chopped	-21.4770	14.1671	205	-1.52	0.1311
242	1	Group*SampleName	Vegetable	Oat puree	Control	Asparagus puree	-15.7270	14.1671	205	-1.11	0.2683
243	1	Group*SampleName	Vegetable	Oat puree	Control	Beef puree	3.5855	14.1671	205	0.25	0.8005
244	1	Group*SampleName	Vegetable	Oat puree	Control	Black bean puree	-3.9145	14.1671	205	-0.28	0.7826
245	1	Group*SampleName	Vegetable	Oat puree	Control	Broccoli chopped	-19.4770	14.1671	205	-1.37	0.1707
246	1	Group*SampleName	Vegetable	Oat puree	Control	Broccoli puree	-13.5395	14.1671	205	-0.96	0.3404
247	1	Group*SampleName	Vegetable	Oat puree	Control	Chicken puree	3.7730	14.1671	205	0.27	0.7903
248	1	Group*SampleName	Vegetable	Oat puree	Control	Kale chopped	10.2730	14.1671	205	0.73	0.4692
249	1	Group*SampleName	Vegetable	Oat puree	Control	Kale puree	1.8355	14.1671	205	0.13	0.8970
250	1	Group*SampleName	Vegetable	Oat puree	Control	Oat puree	16.3355	14.1671	205	1.15	0.2502
251	1	Group*SampleName	Vegetable	Oat puree	Control	Spinach chopped	2.7730	14.1671	205	0.20	0.8450
252	1	Group*SampleName	Vegetable	Oat puree	Control	Spinach puree	3.3355	14.1671	205	0.24	0.8141
253	1	Group*SampleName	Vegetable	Spinach chopped	Vegetable	Spinach puree	2.0000	11.7045	352	0.17	0.8644
254	1	Group*SampleName	Vegetable	Spinach chopped	Control	Asparagus chopped	-22.8103	14.1671	205	-1.61	0.1089
255	1	Group*SampleName	Vegetable	Spinach chopped	Control	Asparagus puree	-17.0603	14.1671	205	-1.20	0.2299
256	1	Group*SampleName	Vegetable	Spinach chopped	Control	Beef puree	2.2522	14.1671	205	0.16	0.8738
257	1	Group*SampleName	Vegetable	Spinach chopped	Control	Black bean puree	-5.2478	14.1671	205	-0.37	0.7115
258	1	Group*SampleName	Vegetable	Spinach chopped	Control	Broccoli chopped	-20.8103	14.1671	205	-1.47	0.1434
259	1	Group*SampleName	Vegetable	Spinach chopped	Control	Broccoli puree	-14.8728	14.1671	205	-1.05	0.2950
260	1	Group*SampleName	Vegetable	Spinach chopped	Control	Chicken puree	2.4397	14.1671	205	0.17	0.8634
261	1	Group*SampleName	Vegetable	Spinach chopped	Control	Kale chopped	8.9397	14.1671	205	0.63	0.5287
262	1	Group*SampleName	Vegetable	Spinach chopped	Control	Kale puree	0.5022	14.1671	205	0.04	0.9718
263	1	Group*SampleName	Vegetable	Spinach chopped	Control	Oat puree	15.0022	14.1671	205	1.06	0.2909
264	1	Group*SampleName	Vegetable	Spinach chopped	Control	Spinach chopped	1.4397	14.1671	205	0.10	0.9192
265	1	Group*SampleName	Vegetable	Spinach chopped	Control	Spinach puree	2.0022	14.1671	205	0.14	0.8878
266	1	Group*SampleName	Vegetable	Spinach puree	Control	Asparagus chopped	-24.8103	14.1671	205	-1.75	0.0814
267	1	Group*SampleName	Vegetable	Spinach puree	Control	Asparagus puree	-19.0603	14.1671	205	-1.35	0.1800
268	1	Group*SampleName	Vegetable	Spinach puree	Control	Beef puree	0.2522	14.1671	205	0.02	0.9858
269	1	Group*SampleName	Vegetable	Spinach puree	Control	Black bean puree	-7.2478	14.1671	205	-0.51	0.6095
270	1	Group*SampleName	Vegetable	Spinach puree	Control	Broccoli chopped	-22.8103	14.1671	205	-1.61	0.1089
271	1	Group*SampleName	Vegetable	Spinach puree	Control	Broccoli puree	-16.8728	14.1671	205	-1.19	0.2350

Obs	GameVisit	Effect	Group	SampleName	_Group	_SampleName	Estimate	StdErr	DF	tValue	Probt
272	1	Group*SampleName	Vegetable	Spinach puree	Control	Chicken puree	0.4397	14.1671	205	0.03	0.9753
273	1	Group*SampleName	Vegetable	Spinach puree	Control	Kale chopped	6.9397	14.1671	205	0.49	0.6248
274	1	Group*SampleName	Vegetable	Spinach puree	Control	Kale puree	-1.4978	14.1671	205	-0.11	0.9159
275	1	Group*SampleName	Vegetable	Spinach puree	Control	Oat puree	13.0022	14.1671	205	0.92	0.3598
276	1	Group*SampleName	Vegetable	Spinach puree	Control	Spinach chopped	-0.5603	14.1671	205	-0.04	0.9685
277	1	Group*SampleName	Vegetable	Spinach puree	Control	Spinach puree	0.002202	14.1671	205	0.00	0.9999
278	1	Group*SampleName	Control	Asparagus chopped	Control	Asparagus puree	5.7500	12.4145	352	0.46	0.6435
279	1	Group*SampleName	Control	Asparagus chopped	Control	Beef puree	25.0625	12.4145	352	2.02	0.0443
280	1	Group*SampleName	Control	Asparagus chopped	Control	Black bean puree	17.5625	12.4145	352	1.41	0.1580
281	1	Group*SampleName	Control	Asparagus chopped	Control	Broccoli chopped	2.0000	12.4145	352	0.16	0.8721
282	1	Group*SampleName	Control	Asparagus chopped	Control	Broccoli puree	7.9375	12.4145	352	0.64	0.5230
283	1	Group*SampleName	Control	Asparagus chopped	Control	Chicken puree	25.2500	12.4145	352	2.03	0.0427
284	1	Group*SampleName	Control	Asparagus chopped	Control	Kale chopped	31.7500	12.4145	352	2.56	0.0110
285	1	Group*SampleName	Control	Asparagus chopped	Control	Kale puree	23.3125	12.4145	352	1.88	0.0612
286	1	Group*SampleName	Control	Asparagus chopped	Control	Oat puree	37.8125	12.4145	352	3.05	0.0025
287	1	Group*SampleName	Control	Asparagus chopped	Control	Spinach chopped	24.2500	12.4145	352	1.95	0.0516
288	1	Group*SampleName	Control	Asparagus chopped	Control	Spinach puree	24.8125	12.4145	352	2.00	0.0464
289	1	Group*SampleName	Control	Asparagus puree	Control	Beef puree	19.3125	12.4145	352	1.56	0.1207
290	1	Group*SampleName	Control	Asparagus puree	Control	Black bean puree	11.8125	12.4145	352	0.95	0.3420
291	1	Group*SampleName	Control	Asparagus puree	Control	Broccoli chopped	-3.7500	12.4145	352	-0.30	0.7628
292	1	Group*SampleName	Control	Asparagus puree	Control	Broccoli puree	2.1875	12.4145	352	0.18	0.8602
293	1	Group*SampleName	Control	Asparagus puree	Control	Chicken puree	19.5000	12.4145	352	1.57	0.1171
294	1	Group*SampleName	Control	Asparagus puree	Control	Kale chopped	26.0000	12.4145	352	2.09	0.0369
295	1	Group*SampleName	Control	Asparagus puree	Control	Kale puree	17.5625	12.4145	352	1.41	0.1580
296	1	Group*SampleName	Control	Asparagus puree	Control	Oat puree	32.0625	12.4145	352	2.58	0.0102
297	1	Group*SampleName	Control	Asparagus puree	Control	Spinach chopped	18.5000	12.4145	352	1.49	0.1371
298	1	Group*SampleName	Control	Asparagus puree	Control	Spinach puree	19.0625	12.4145	352	1.54	0.1256
299	1	Group*SampleName	Control	Beef puree	Control	Black bean puree	-7.5000	12.4145	352	-0.60	0.5461
300	1	Group*SampleName	Control	Beef puree	Control	Broccoli	-23.0625	12.4145	352	-1.86	0.0640

Obs	GameVisit	Effect	Group	SampleName	_Group	_SampleName	Estimate	StdErr	DF	tValue	Probt
						chopped					
301	1	Group*SampleName	Control	Beef puree	Control	Broccoli puree	-17.1250	12.4145	352	-1.38	0.1686
302	1	Group*SampleName	Control	Beef puree	Control	Chicken puree	0.1875	12.4145	352	0.02	0.9880
303	1	Group*SampleName	Control	Beef puree	Control	Kale chopped	6.6875	12.4145	352	0.54	0.5904
304	1	Group*SampleName	Control	Beef puree	Control	Kale puree	-1.7500	12.4145	352	-0.14	0.8880
305	1	Group*SampleName	Control	Beef puree	Control	Oat puree	12.7500	12.4145	352	1.03	0.3051
306	1	Group*SampleName	Control	Beef puree	Control	Spinach chopped	-0.8125	12.4145	352	-0.07	0.9479
307	1	Group*SampleName	Control	Beef puree	Control	Spinach puree	-0.2500	12.4145	352	-0.02	0.9839
308	1	Group*SampleName	Control	Black bean puree	Control	Broccoli chopped	-15.5625	12.4145	352	-1.25	0.2108
309	1	Group*SampleName	Control	Black bean puree	Control	Broccoli puree	-9.6250	12.4145	352	-0.78	0.4387
310	1	Group*SampleName	Control	Black bean puree	Control	Chicken puree	7.6875	12.4145	352	0.62	0.5362
311	1	Group*SampleName	Control	Black bean puree	Control	Kale chopped	14.1875	12.4145	352	1.14	0.2539
312	1	Group*SampleName	Control	Black bean puree	Control	Kale puree	5.7500	12.4145	352	0.46	0.6435
313	1	Group*SampleName	Control	Black bean puree	Control	Oat puree	20.2500	12.4145	352	1.63	0.1038
314	1	Group*SampleName	Control	Black bean puree	Control	Spinach chopped	6.6875	12.4145	352	0.54	0.5904
315	1	Group*SampleName	Control	Black bean puree	Control	Spinach puree	7.2500	12.4145	352	0.58	0.5596
316	1	Group*SampleName	Control	Broccoli chopped	Control	Broccoli puree	5.9375	12.4145	352	0.48	0.6328
317	1	Group*SampleName	Control	Broccoli chopped	Control	Chicken puree	23.2500	12.4145	352	1.87	0.0619
318	1	Group*SampleName	Control	Broccoli chopped	Control	Kale chopped	29.7500	12.4145	352	2.40	0.0171
319	1	Group*SampleName	Control	Broccoli chopped	Control	Kale puree	21.3125	12.4145	352	1.72	0.0869
320	1	Group*SampleName	Control	Broccoli chopped	Control	Oat puree	35.8125	12.4145	352	2.88	0.0042
321	1	Group*SampleName	Control	Broccoli chopped	Control	Spinach chopped	22.2500	12.4145	352	1.79	0.0739
322	1	Group*SampleName	Control	Broccoli chopped	Control	Spinach puree	22.8125	12.4145	352	1.84	0.0670
323	1	Group*SampleName	Control	Broccoli puree	Control	Chicken puree	17.3125	12.4145	352	1.39	0.1640
324	1	Group*SampleName	Control	Broccoli puree	Control	Kale chopped	23.8125	12.4145	352	1.92	0.0559
325	1	Group*SampleName	Control	Broccoli puree	Control	Kale puree	15.3750	12.4145	352	1.24	0.2164
326	1	Group*SampleName	Control	Broccoli puree	Control	Oat puree	29.8750	12.4145	352	2.41	0.0166
327	1	Group*SampleName	Control	Broccoli puree	Control	Spinach chopped	16.3125	12.4145	352	1.31	0.1897
328	1	Group*SampleName	Control	Broccoli puree	Control	Spinach puree	16.8750	12.4145	352	1.36	0.1749
329	1	Group*SampleName	Control	Chicken puree	Control	Kale chopped	6.5000	12.4145	352	0.52	0.6009
330	1	Group*SampleName	Control	Chicken puree	Control	Kale puree	-1.9375	12.4145	352	-0.16	0.8761

Obs	GameVisit	Effect	Group	SampleName	_Group	_SampleName	Estimate	StdErr	DF	tValue	Probt
331	1	Group*SampleName	Control	Chicken puree	Control	Oat puree	12.5625	12.4145	352	1.01	0.3123
332	1	Group*SampleName	Control	Chicken puree	Control	Spinach chopped	-1.0000	12.4145	352	-0.08	0.9358
333	1	Group*SampleName	Control	Chicken puree	Control	Spinach puree	-0.4375	12.4145	352	-0.04	0.9719
334	1	Group*SampleName	Control	Kale chopped	Control	Kale puree	-8.4375	12.4145	352	-0.68	0.4972
335	1	Group*SampleName	Control	Kale chopped	Control	Oat puree	6.0625	12.4145	352	0.49	0.6256
336	1	Group*SampleName	Control	Kale chopped	Control	Spinach chopped	-7.5000	12.4145	352	-0.60	0.5461
337	1	Group*SampleName	Control	Kale chopped	Control	Spinach puree	-6.9375	12.4145	352	-0.56	0.5766
338	1	Group*SampleName	Control	Kale puree	Control	Oat puree	14.5000	12.4145	352	1.17	0.2436
339	1	Group*SampleName	Control	Kale puree	Control	Spinach chopped	0.9375	12.4145	352	0.08	0.9398
340	1	Group*SampleName	Control	Kale puree	Control	Spinach puree	1.5000	12.4145	352	0.12	0.9039
341	1	Group*SampleName	Control	Oat puree	Control	Spinach chopped	-13.5625	12.4145	352	-1.09	0.2754
342	1	Group*SampleName	Control	Oat puree	Control	Spinach puree	-13.0000	12.4145	352	-1.05	0.2957
343	1	Group*SampleName	Control	Spinach chopped	Control	Spinach puree	0.5625	12.4145	352	0.05	0.9639
344	2	Group	Vegetable		Control		-4.1751	7.8725	31	-0.53	0.5997
345	2	SampleName		Asparagus chopped		Asparagus puree	4.1771	8.3312	352	0.50	0.6164
346	2	SampleName		Asparagus chopped		Beef puree	27.8542	8.3312	352	3.34	0.0009
347	2	SampleName		Asparagus chopped		Black bean puree	9.5347	8.3312	352	1.14	0.2532
348	2	SampleName		Asparagus chopped		Broccoli chopped	-6.9306	8.3312	352	-0.83	0.4060
349	2	SampleName		Asparagus chopped		Broccoli puree	2.9236	8.3312	352	0.35	0.7259
350	2	SampleName		Asparagus chopped		Chicken puree	20.8229	8.3312	352	2.50	0.0129
351	2	SampleName		Asparagus chopped		Kale chopped	29.0486	8.3312	352	3.49	0.0006
352	2	SampleName		Asparagus chopped		Kale puree	29.0868	8.3312	352	3.49	0.0005
353	2	SampleName		Asparagus chopped		Oat puree	19.5938	8.3312	352	2.35	0.0192
354	2	SampleName		Asparagus chopped		Spinach chopped	19.0903	8.3312	352	2.29	0.0225
355	2	SampleName		Asparagus chopped		Spinach puree	23.1250	8.3312	352	2.78	0.0058
356	2	SampleName		Asparagus puree		Beef puree	23.6771	8.3312	352	2.84	0.0047
357	2	SampleName		Asparagus puree		Black bean puree	5.3576	8.3312	352	0.64	0.5206
358	2	SampleName		Asparagus puree		Broccoli chopped	-11.1076	8.3312	352	-1.33	0.1833
359	2	SampleName		Asparagus puree		Broccoli puree	-1.2535	8.3312	352	-0.15	0.8805
360	2	SampleName		Asparagus puree		Chicken puree	16.6458	8.3312	352	2.00	0.0465
361	2	SampleName		Asparagus puree		Kale chopped	24.8715	8.3312	352	2.99	0.0030

Obs	GameVisit	Effect	Group	SampleName	_Group	_SampleName	Estimate	StdErr	DF	tValue	Probt
362	2	SampleName		Asparagus puree		Kale puree	24.9097	8.3312	352	2.99	0.0030
363	2	SampleName		Asparagus puree		Oat puree	15.4167	8.3312	352	1.85	0.0651
364	2	SampleName		Asparagus puree		Spinach chopped	14.9132	8.3312	352	1.79	0.0743
365	2	SampleName		Asparagus puree		Spinach puree	18.9479	8.3312	352	2.27	0.0235
366	2	SampleName		Beef puree		Black bean puree	-18.3194	8.3312	352	-2.20	0.0285
367	2	SampleName		Beef puree		Broccoli chopped	-34.7847	8.3312	352	-4.18	<.0001
368	2	SampleName		Beef puree		Broccoli puree	-24.9306	8.3312	352	-2.99	0.0030
369	2	SampleName		Beef puree		Chicken puree	-7.0312	8.3312	352	-0.84	0.3993
370	2	SampleName		Beef puree		Kale chopped	1.1944	8.3312	352	0.14	0.8861
371	2	SampleName		Beef puree		Kale puree	1.2326	8.3312	352	0.15	0.8825
372	2	SampleName		Beef puree		Oat puree	-8.2604	8.3312	352	-0.99	0.3221
373	2	SampleName		Beef puree		Spinach chopped	-8.7639	8.3312	352	-1.05	0.2935
374	2	SampleName		Beef puree		Spinach puree	-4.7292	8.3312	352	-0.57	0.5706
375	2	SampleName		Black bean puree		Broccoli chopped	-16.4653	8.3312	352	-1.98	0.0489
376	2	SampleName		Black bean puree		Broccoli puree	-6.6111	8.3312	352	-0.79	0.4280
377	2	SampleName		Black bean puree		Chicken puree	11.2882	8.3312	352	1.35	0.1763
378	2	SampleName		Black bean puree		Kale chopped	19.5139	8.3312	352	2.34	0.0197
379	2	SampleName		Black bean puree		Kale puree	19.5521	8.3312	352	2.35	0.0195
380	2	SampleName		Black bean puree		Oat puree	10.0590	8.3312	352	1.21	0.2281
381	2	SampleName		Black bean puree		Spinach chopped	9.5556	8.3312	352	1.15	0.2522
382	2	SampleName		Black bean puree		Spinach puree	13.5903	8.3312	352	1.63	0.1037
383	2	SampleName		Broccoli chopped		Broccoli puree	9.8542	8.3312	352	1.18	0.2377
384	2	SampleName		Broccoli chopped		Chicken puree	27.7535	8.3312	352	3.33	0.0010
385	2	SampleName		Broccoli chopped		Kale chopped	35.9792	8.3312	352	4.32	<.0001
386	2	SampleName		Broccoli chopped		Kale puree	36.0174	8.3312	352	4.32	<.0001
387	2	SampleName		Broccoli chopped		Oat puree	26.5243	8.3312	352	3.18	0.0016
388	2	SampleName		Broccoli chopped		Spinach chopped	26.0208	8.3312	352	3.12	0.0019
389	2	SampleName		Broccoli chopped		Spinach puree	30.0556	8.3312	352	3.61	0.0004
390	2	SampleName		Broccoli puree		Chicken puree	17.8993	8.3312	352	2.15	0.0324
391	2	SampleName		Broccoli puree		Kale chopped	26.1250	8.3312	352	3.14	0.0019
392	2	SampleName		Broccoli		Kale puree	26.1632	8.3312	352	3.14	0.0018

Obs	GameVisit	Effect	Group	SampleName	_Group	_SampleName	Estimate	StdErr	DF	tValue	Probt
				puree							
393	2	SampleName		Broccoli puree		Oat puree	16.6701	8.3312	352	2.00	0.0462
394	2	SampleName		Broccoli puree		Spinach chopped	16.1667	8.3312	352	1.94	0.0531
395	2	SampleName		Broccoli puree		Spinach puree	20.2014	8.3312	352	2.42	0.0158
396	2	SampleName		Chicken puree		Kale chopped	8.2257	8.3312	352	0.99	0.3242
397	2	SampleName		Chicken puree		Kale puree	8.2639	8.3312	352	0.99	0.3219
398	2	SampleName		Chicken puree		Oat puree	-1.2292	8.3312	352	-0.15	0.8828
399	2	SampleName		Chicken puree		Spinach chopped	-1.7326	8.3312	352	-0.21	0.8354
400	2	SampleName		Chicken puree		Spinach puree	2.3021	8.3312	352	0.28	0.7825
401	2	SampleName		Kale chopped		Kale puree	0.03819	8.3312	352	0.00	0.9963
402	2	SampleName		Kale chopped		Oat puree	-9.4549	8.3312	352	-1.13	0.2572
403	2	SampleName		Kale chopped		Spinach chopped	-9.9583	8.3312	352	-1.20	0.2328
404	2	SampleName		Kale chopped		Spinach puree	-5.9236	8.3312	352	-0.71	0.4775
405	2	SampleName		Kale puree		Oat puree	-9.4931	8.3312	352	-1.14	0.2553
406	2	SampleName		Kale puree		Spinach chopped	-9.9965	8.3312	352	-1.20	0.2310
407	2	SampleName		Kale puree		Spinach puree	-5.9618	8.3312	352	-0.72	0.4747
408	2	SampleName		Oat puree		Spinach chopped	-0.5035	8.3312	352	-0.06	0.9518
409	2	SampleName		Oat puree		Spinach puree	3.5312	8.3312	352	0.42	0.6719
410	2	SampleName		Spinach chopped		Spinach puree	4.0347	8.3312	352	0.48	0.6285
411	2	Group*SampleName	Vegetable	Asparagus chopped	Vegetable	Asparagus puree	13.1667	11.4303	352	1.15	0.2501
412	2	Group*SampleName	Vegetable	Asparagus chopped	Vegetable	Beef puree	38.8333	11.4303	352	3.40	0.0008
413	2	Group*SampleName	Vegetable	Asparagus chopped	Vegetable	Black bean puree	17.9444	11.4303	352	1.57	0.1173
414	2	Group*SampleName	Vegetable	Asparagus chopped	Vegetable	Broccoli chopped	-2.1111	11.4303	352	-0.18	0.8536
415	2	Group*SampleName	Vegetable	Asparagus chopped	Vegetable	Broccoli puree	-2.7778	11.4303	352	-0.24	0.8081
416	2	Group*SampleName	Vegetable	Asparagus chopped	Vegetable	Chicken puree	28.3333	11.4303	352	2.48	0.0137
417	2	Group*SampleName	Vegetable	Asparagus chopped	Vegetable	Kale chopped	24.7222	11.4303	352	2.16	0.0312
418	2	Group*SampleName	Vegetable	Asparagus chopped	Vegetable	Kale puree	25.6111	11.4303	352	2.24	0.0257
419	2	Group*SampleName	Vegetable	Asparagus chopped	Vegetable	Oat puree	13.5000	11.4303	352	1.18	0.2384
420	2	Group*SampleName	Vegetable	Asparagus chopped	Vegetable	Spinach chopped	17.0556	11.4303	352	1.49	0.1366
421	2	Group*SampleName	Vegetable	Asparagus chopped	Vegetable	Spinach puree	23.0000	11.4303	352	2.01	0.0450
422	2	Group*SampleName	Vegetable	Asparagus chopped	Control	Asparagus chopped	-1.0165	13.7559	211	-0.07	0.9412

Obs	GameVisit	Effect	Group	SampleName	_Group	_SampleName	Estimate	StdErr	DF	tValue	Probt
423	2	Group*SampleName	Vegetable	Asparagus chopped	Control	Asparagus puree	-5.8290	13.7559	211	-0.42	0.6722
424	2	Group*SampleName	Vegetable	Asparagus chopped	Control	Beef puree	15.8585	13.7559	211	1.15	0.2503
425	2	Group*SampleName	Vegetable	Asparagus chopped	Control	Black bean puree	0.1085	13.7559	211	0.01	0.9937
426	2	Group*SampleName	Vegetable	Asparagus chopped	Control	Broccoli chopped	-12.7665	13.7559	211	-0.93	0.3544
427	2	Group*SampleName	Vegetable	Asparagus chopped	Control	Broccoli puree	7.6085	13.7559	211	0.55	0.5808
428	2	Group*SampleName	Vegetable	Asparagus chopped	Control	Chicken puree	12.2960	13.7559	211	0.89	0.3724
429	2	Group*SampleName	Vegetable	Asparagus chopped	Control	Kale chopped	32.3585	13.7559	211	2.35	0.0196
430	2	Group*SampleName	Vegetable	Asparagus chopped	Control	Kale puree	31.5460	13.7559	211	2.29	0.0228
431	2	Group*SampleName	Vegetable	Asparagus chopped	Control	Oat puree	24.6710	13.7559	211	1.79	0.0743
432	2	Group*SampleName	Vegetable	Asparagus chopped	Control	Spinach chopped	20.1085	13.7559	211	1.46	0.1453
433	2	Group*SampleName	Vegetable	Asparagus chopped	Control	Spinach puree	22.2335	13.7559	211	1.62	0.1075
434	2	Group*SampleName	Vegetable	Asparagus puree	Vegetable	Beef puree	25.6667	11.4303	352	2.25	0.0254
435	2	Group*SampleName	Vegetable	Asparagus puree	Vegetable	Black bean puree	4.7778	11.4303	352	0.42	0.6762
436	2	Group*SampleName	Vegetable	Asparagus puree	Vegetable	Broccoli chopped	-15.2778	11.4303	352	-1.34	0.1822
437	2	Group*SampleName	Vegetable	Asparagus puree	Vegetable	Broccoli puree	-15.9444	11.4303	352	-1.39	0.1639
438	2	Group*SampleName	Vegetable	Asparagus puree	Vegetable	Chicken puree	15.1667	11.4303	352	1.33	0.1854
439	2	Group*SampleName	Vegetable	Asparagus puree	Vegetable	Kale chopped	11.5556	11.4303	352	1.01	0.3127
440	2	Group*SampleName	Vegetable	Asparagus puree	Vegetable	Kale puree	12.4444	11.4303	352	1.09	0.2770
441	2	Group*SampleName	Vegetable	Asparagus puree	Vegetable	Oat puree	0.3333	11.4303	352	0.03	0.9768
442	2	Group*SampleName	Vegetable	Asparagus puree	Vegetable	Spinach chopped	3.8889	11.4303	352	0.34	0.7339
443	2	Group*SampleName	Vegetable	Asparagus puree	Vegetable	Spinach puree	9.8333	11.4303	352	0.86	0.3902
444	2	Group*SampleName	Vegetable	Asparagus puree	Control	Asparagus chopped	-14.1832	13.7559	211	-1.03	0.3037
445	2	Group*SampleName	Vegetable	Asparagus puree	Control	Asparagus puree	-18.9957	13.7559	211	-1.38	0.1688
446	2	Group*SampleName	Vegetable	Asparagus puree	Control	Beef puree	2.6918	13.7559	211	0.20	0.8450
447	2	Group*SampleName	Vegetable	Asparagus puree	Control	Black bean puree	-13.0582	13.7559	211	-0.95	0.3436
448	2	Group*SampleName	Vegetable	Asparagus puree	Control	Broccoli chopped	-25.9332	13.7559	211	-1.89	0.0608
449	2	Group*SampleName	Vegetable	Asparagus puree	Control	Broccoli puree	-5.5582	13.7559	211	-0.40	0.6866
450	2	Group*SampleName	Vegetable	Asparagus puree	Control	Chicken puree	-0.8707	13.7559	211	-0.06	0.9496
451	2	Group*SampleName	Vegetable	Asparagus	Control	Kale chopped	19.1918	13.7559	211	1.40	0.1644

Obs	GameVisit	Effect	Group	SampleName	_Group	_SampleName	Estimate	StdErr	DF	tValue	Probt
				puree							
452	2	Group*SampleName	Vegetable	Asparagus puree	Control	Kale puree	18.3793	13.7559	211	1.34	0.1830
453	2	Group*SampleName	Vegetable	Asparagus puree	Control	Oat puree	11.5043	13.7559	211	0.84	0.4039
454	2	Group*SampleName	Vegetable	Asparagus puree	Control	Spinach chopped	6.9418	13.7559	211	0.50	0.6143
455	2	Group*SampleName	Vegetable	Asparagus puree	Control	Spinach puree	9.0668	13.7559	211	0.66	0.5105
456	2	Group*SampleName	Vegetable	Beef puree	Vegetable	Black bean puree	-20.8889	11.4303	352	-1.83	0.0685
457	2	Group*SampleName	Vegetable	Beef puree	Vegetable	Broccoli chopped	-40.9444	11.4303	352	-3.58	0.0004
458	2	Group*SampleName	Vegetable	Beef puree	Vegetable	Broccoli puree	-41.6111	11.4303	352	-3.64	0.0003
459	2	Group*SampleName	Vegetable	Beef puree	Vegetable	Chicken puree	-10.5000	11.4303	352	-0.92	0.3589
460	2	Group*SampleName	Vegetable	Beef puree	Vegetable	Kale chopped	-14.1111	11.4303	352	-1.23	0.2178
461	2	Group*SampleName	Vegetable	Beef puree	Vegetable	Kale puree	-13.2222	11.4303	352	-1.16	0.2481
462	2	Group*SampleName	Vegetable	Beef puree	Vegetable	Oat puree	-25.3333	11.4303	352	-2.22	0.0273
463	2	Group*SampleName	Vegetable	Beef puree	Vegetable	Spinach chopped	-21.7778	11.4303	352	-1.91	0.0576
464	2	Group*SampleName	Vegetable	Beef puree	Vegetable	Spinach puree	-15.8333	11.4303	352	-1.39	0.1669
465	2	Group*SampleName	Vegetable	Beef puree	Control	Asparagus chopped	-39.8499	13.7559	211	-2.90	0.0042
466	2	Group*SampleName	Vegetable	Beef puree	Control	Asparagus puree	-44.6624	13.7559	211	-3.25	0.0014
467	2	Group*SampleName	Vegetable	Beef puree	Control	Beef puree	-22.9749	13.7559	211	-1.67	0.0964
468	2	Group*SampleName	Vegetable	Beef puree	Control	Black bean puree	-38.7249	13.7559	211	-2.82	0.0053
469	2	Group*SampleName	Vegetable	Beef puree	Control	Broccoli chopped	-51.5999	13.7559	211	-3.75	0.0002
470	2	Group*SampleName	Vegetable	Beef puree	Control	Broccoli puree	-31.2249	13.7559	211	-2.27	0.0242
471	2	Group*SampleName	Vegetable	Beef puree	Control	Chicken puree	-26.5374	13.7559	211	-1.93	0.0551
472	2	Group*SampleName	Vegetable	Beef puree	Control	Kale chopped	-6.4749	13.7559	211	-0.47	0.6383
473	2	Group*SampleName	Vegetable	Beef puree	Control	Kale puree	-7.2874	13.7559	211	-0.53	0.5968
474	2	Group*SampleName	Vegetable	Beef puree	Control	Oat puree	-14.1624	13.7559	211	-1.03	0.3044
475	2	Group*SampleName	Vegetable	Beef puree	Control	Spinach chopped	-18.7249	13.7559	211	-1.36	0.1749
476	2	Group*SampleName	Vegetable	Beef puree	Control	Spinach puree	-16.5999	13.7559	211	-1.21	0.2289
477	2	Group*SampleName	Vegetable	Black bean puree	Vegetable	Broccoli chopped	-20.0556	11.4303	352	-1.75	0.0802
478	2	Group*SampleName	Vegetable	Black bean puree	Vegetable	Broccoli puree	-20.7222	11.4303	352	-1.81	0.0707
479	2	Group*SampleName	Vegetable	Black bean puree	Vegetable	Chicken puree	10.3889	11.4303	352	0.91	0.3640
480	2	Group*SampleName	Vegetable	Black bean puree	Vegetable	Kale chopped	6.7778	11.4303	352	0.59	0.5536
481	2	Group*SampleName	Vegetable	Black bean puree	Vegetable	Kale puree	7.6667	11.4303	352	0.67	0.5028
482	2	Group*SampleName	Vegetable	Black bean puree	Vegetable	Oat puree	-4.4444	11.4303	352	-0.39	0.6976
483	2	Group*SampleName	Vegetable	Black bean puree	Vegetable	Spinach chopped	-0.8889	11.4303	352	-0.08	0.9381

Obs	GameVisit	Effect	Group	SampleName	_Group	_SampleName	Estimate	StdErr	DF	tValue	Probt
484	2	Group*SampleName	Vegetable	Black bean puree	Vegetable	Spinach puree	5.0556	11.4303	352	0.44	0.6585
485	2	Group*SampleName	Vegetable	Black bean puree	Control	Asparagus chopped	-18.9610	13.7559	211	-1.38	0.1695
486	2	Group*SampleName	Vegetable	Black bean puree	Control	Asparagus puree	-23.7735	13.7559	211	-1.73	0.0854
487	2	Group*SampleName	Vegetable	Black bean puree	Control	Beef puree	-2.0860	13.7559	211	-0.15	0.8796
488	2	Group*SampleName	Vegetable	Black bean puree	Control	Black bean puree	-17.8360	13.7559	211	-1.30	0.1962
489	2	Group*SampleName	Vegetable	Black bean puree	Control	Broccoli chopped	-30.7110	13.7559	211	-2.23	0.0266
490	2	Group*SampleName	Vegetable	Black bean puree	Control	Broccoli puree	-10.3360	13.7559	211	-0.75	0.4533
491	2	Group*SampleName	Vegetable	Black bean puree	Control	Chicken puree	-5.6485	13.7559	211	-0.41	0.6818
492	2	Group*SampleName	Vegetable	Black bean puree	Control	Kale chopped	14.4140	13.7559	211	1.05	0.2959
493	2	Group*SampleName	Vegetable	Black bean puree	Control	Kale puree	13.6015	13.7559	211	0.99	0.3239
494	2	Group*SampleName	Vegetable	Black bean puree	Control	Oat puree	6.7265	13.7559	211	0.49	0.6254
495	2	Group*SampleName	Vegetable	Black bean puree	Control	Spinach chopped	2.1640	13.7559	211	0.16	0.8751
496	2	Group*SampleName	Vegetable	Black bean puree	Control	Spinach puree	4.2890	13.7559	211	0.31	0.7555
497	2	Group*SampleName	Vegetable	Broccoli chopped	Vegetable	Broccoli puree	-0.6667	11.4303	352	-0.06	0.9535
498	2	Group*SampleName	Vegetable	Broccoli chopped	Vegetable	Chicken puree	30.4444	11.4303	352	2.66	0.0081
499	2	Group*SampleName	Vegetable	Broccoli chopped	Vegetable	Kale chopped	26.8333	11.4303	352	2.35	0.0194
500	2	Group*SampleName	Vegetable	Broccoli chopped	Vegetable	Kale puree	27.7222	11.4303	352	2.43	0.0158
501	2	Group*SampleName	Vegetable	Broccoli chopped	Vegetable	Oat puree	15.6111	11.4303	352	1.37	0.1729
502	2	Group*SampleName	Vegetable	Broccoli chopped	Vegetable	Spinach chopped	19.1667	11.4303	352	1.68	0.0945
503	2	Group*SampleName	Vegetable	Broccoli chopped	Vegetable	Spinach puree	25.1111	11.4303	352	2.20	0.0287
504	2	Group*SampleName	Vegetable	Broccoli chopped	Control	Asparagus chopped	1.0946	13.7559	211	0.08	0.9367
505	2	Group*SampleName	Vegetable	Broccoli chopped	Control	Asparagus puree	-3.7179	13.7559	211	-0.27	0.7872
506	2	Group*SampleName	Vegetable	Broccoli chopped	Control	Beef puree	17.9696	13.7559	211	1.31	0.1929
507	2	Group*SampleName	Vegetable	Broccoli chopped	Control	Black bean puree	2.2196	13.7559	211	0.16	0.8720
508	2	Group*SampleName	Vegetable	Broccoli chopped	Control	Broccoli chopped	-10.6554	13.7559	211	-0.77	0.4394
509	2	Group*SampleName	Vegetable	Broccoli chopped	Control	Broccoli puree	9.7196	13.7559	211	0.71	0.4806
510	2	Group*SampleName	Vegetable	Broccoli chopped	Control	Chicken puree	14.4071	13.7559	211	1.05	0.2961
511	2	Group*SampleName	Vegetable	Broccoli chopped	Control	Kale chopped	34.4696	13.7559	211	2.51	0.0130
512	2	Group*SampleName	Vegetable	Broccoli	Control	Kale puree	33.6571	13.7559	211	2.45	0.0152

Obs	GameVisit	Effect	Group	SampleName	_Group	_SampleName	Estimate	StdErr	DF	tValue	Probt
				chopped							
513	2	Group*SampleName	Vegetable	Broccoli chopped	Control	Oat puree	26.7821	13.7559	211	1.95	0.0529
514	2	Group*SampleName	Vegetable	Broccoli chopped	Control	Spinach chopped	22.2196	13.7559	211	1.62	0.1077
515	2	Group*SampleName	Vegetable	Broccoli chopped	Control	Spinach puree	24.3446	13.7559	211	1.77	0.0782
516	2	Group*SampleName	Vegetable	Broccoli puree	Vegetable	Chicken puree	31.1111	11.4303	352	2.72	0.0068
517	2	Group*SampleName	Vegetable	Broccoli puree	Vegetable	Kale chopped	27.5000	11.4303	352	2.41	0.0166
518	2	Group*SampleName	Vegetable	Broccoli puree	Vegetable	Kale puree	28.3889	11.4303	352	2.48	0.0135
519	2	Group*SampleName	Vegetable	Broccoli puree	Vegetable	Oat puree	16.2778	11.4303	352	1.42	0.1553
520	2	Group*SampleName	Vegetable	Broccoli puree	Vegetable	Spinach chopped	19.8333	11.4303	352	1.74	0.0836
521	2	Group*SampleName	Vegetable	Broccoli puree	Vegetable	Spinach puree	25.7778	11.4303	352	2.26	0.0247
522	2	Group*SampleName	Vegetable	Broccoli puree	Control	Asparagus chopped	1.7612	13.7559	211	0.13	0.8982
523	2	Group*SampleName	Vegetable	Broccoli puree	Control	Asparagus puree	-3.0513	13.7559	211	-0.22	0.8247
524	2	Group*SampleName	Vegetable	Broccoli puree	Control	Beef puree	18.6362	13.7559	211	1.35	0.1769
525	2	Group*SampleName	Vegetable	Broccoli puree	Control	Black bean puree	2.8862	13.7559	211	0.21	0.8340
526	2	Group*SampleName	Vegetable	Broccoli puree	Control	Broccoli chopped	-9.9888	13.7559	211	-0.73	0.4686
527	2	Group*SampleName	Vegetable	Broccoli puree	Control	Broccoli puree	10.3862	13.7559	211	0.76	0.4511
528	2	Group*SampleName	Vegetable	Broccoli puree	Control	Chicken puree	15.0737	13.7559	211	1.10	0.2744
529	2	Group*SampleName	Vegetable	Broccoli puree	Control	Kale chopped	35.1362	13.7559	211	2.55	0.0113
530	2	Group*SampleName	Vegetable	Broccoli puree	Control	Kale puree	34.3237	13.7559	211	2.50	0.0134
531	2	Group*SampleName	Vegetable	Broccoli puree	Control	Oat puree	27.4487	13.7559	211	2.00	0.0473
532	2	Group*SampleName	Vegetable	Broccoli puree	Control	Spinach chopped	22.8862	13.7559	211	1.66	0.0977
533	2	Group*SampleName	Vegetable	Broccoli puree	Control	Spinach puree	25.0112	13.7559	211	1.82	0.0705
534	2	Group*SampleName	Vegetable	Chicken puree	Vegetable	Kale chopped	-3.6111	11.4303	352	-0.32	0.7522
535	2	Group*SampleName	Vegetable	Chicken puree	Vegetable	Kale puree	-2.7222	11.4303	352	-0.24	0.8119
536	2	Group*SampleName	Vegetable	Chicken puree	Vegetable	Oat puree	-14.8333	11.4303	352	-1.30	0.1952
537	2	Group*SampleName	Vegetable	Chicken puree	Vegetable	Spinach chopped	-11.2778	11.4303	352	-0.99	0.3245
538	2	Group*SampleName	Vegetable	Chicken puree	Vegetable	Spinach puree	-5.3333	11.4303	352	-0.47	0.6411
539	2	Group*SampleName	Vegetable	Chicken puree	Control	Asparagus chopped	-29.3499	13.7559	211	-2.13	0.0340
540	2	Group*SampleName	Vegetable	Chicken puree	Control	Asparagus puree	-34.1624	13.7559	211	-2.48	0.0138

Obs	GameVisit	Effect	Group	SampleName	_Group	_SampleName	Estimate	StdErr	DF	tValue	Probt
541	2	Group*SampleName	Vegetable	Chicken puree	Control	Beef puree	-12.4749	13.7559	211	-0.91	0.3655
542	2	Group*SampleName	Vegetable	Chicken puree	Control	Black bean puree	-28.2249	13.7559	211	-2.05	0.0414
543	2	Group*SampleName	Vegetable	Chicken puree	Control	Broccoli chopped	-41.0999	13.7559	211	-2.99	0.0031
544	2	Group*SampleName	Vegetable	Chicken puree	Control	Broccoli puree	-20.7249	13.7559	211	-1.51	0.1334
545	2	Group*SampleName	Vegetable	Chicken puree	Control	Chicken puree	-16.0374	13.7559	211	-1.17	0.2450
546	2	Group*SampleName	Vegetable	Chicken puree	Control	Kale chopped	4.0251	13.7559	211	0.29	0.7701
547	2	Group*SampleName	Vegetable	Chicken puree	Control	Kale puree	3.2126	13.7559	211	0.23	0.8156
548	2	Group*SampleName	Vegetable	Chicken puree	Control	Oat puree	-3.6624	13.7559	211	-0.27	0.7903
549	2	Group*SampleName	Vegetable	Chicken puree	Control	Spinach chopped	-8.2249	13.7559	211	-0.60	0.5505
550	2	Group*SampleName	Vegetable	Chicken puree	Control	Spinach puree	-6.0999	13.7559	211	-0.44	0.6579
551	2	Group*SampleName	Vegetable	Kale chopped	Vegetable	Kale puree	0.8889	11.4303	352	0.08	0.9381
552	2	Group*SampleName	Vegetable	Kale chopped	Vegetable	Oat puree	-11.2222	11.4303	352	-0.98	0.3269
553	2	Group*SampleName	Vegetable	Kale chopped	Vegetable	Spinach chopped	-7.6667	11.4303	352	-0.67	0.5028
554	2	Group*SampleName	Vegetable	Kale chopped	Vegetable	Spinach puree	-1.7222	11.4303	352	-0.15	0.8803
555	2	Group*SampleName	Vegetable	Kale chopped	Control	Asparagus chopped	-25.7388	13.7559	211	-1.87	0.0627
556	2	Group*SampleName	Vegetable	Kale chopped	Control	Asparagus puree	-30.5513	13.7559	211	-2.22	0.0274
557	2	Group*SampleName	Vegetable	Kale chopped	Control	Beef puree	-8.8638	13.7559	211	-0.64	0.5200
558	2	Group*SampleName	Vegetable	Kale chopped	Control	Black bean puree	-24.6138	13.7559	211	-1.79	0.0750
559	2	Group*SampleName	Vegetable	Kale chopped	Control	Broccoli chopped	-37.4888	13.7559	211	-2.73	0.0070
560	2	Group*SampleName	Vegetable	Kale chopped	Control	Broccoli puree	-17.1138	13.7559	211	-1.24	0.2148
561	2	Group*SampleName	Vegetable	Kale chopped	Control	Chicken puree	-12.4263	13.7559	211	-0.90	0.3674
562	2	Group*SampleName	Vegetable	Kale chopped	Control	Kale chopped	7.6362	13.7559	211	0.56	0.5794
563	2	Group*SampleName	Vegetable	Kale chopped	Control	Kale puree	6.8237	13.7559	211	0.50	0.6204
564	2	Group*SampleName	Vegetable	Kale chopped	Control	Oat puree	-0.05126	13.7559	211	-0.00	0.9970
565	2	Group*SampleName	Vegetable	Kale chopped	Control	Spinach chopped	-4.6138	13.7559	211	-0.34	0.7377
566	2	Group*SampleName	Vegetable	Kale chopped	Control	Spinach puree	-2.4888	13.7559	211	-0.18	0.8566
567	2	Group*SampleName	Vegetable	Kale puree	Vegetable	Oat puree	-12.1111	11.4303	352	-1.06	0.2901
568	2	Group*SampleName	Vegetable	Kale puree	Vegetable	Spinach chopped	-8.5556	11.4303	352	-0.75	0.4547
569	2	Group*SampleName	Vegetable	Kale puree	Vegetable	Spinach puree	-2.6111	11.4303	352	-0.23	0.8194
570	2	Group*SampleName	Vegetable	Kale puree	Control	Asparagus chopped	-26.6276	13.7559	211	-1.94	0.0542
571	2	Group*SampleName	Vegetable	Kale puree	Control	Asparagus puree	-31.4401	13.7559	211	-2.29	0.0233
572	2	Group*SampleName	Vegetable	Kale puree	Control	Beef puree	-9.7526	13.7559	211	-0.71	0.4791
573	2	Group*SampleName	Vegetable	Kale puree	Control	Black bean puree	-25.5026	13.7559	211	-1.85	0.0651

Obs	GameVisit	Effect	Group	SampleName	_Group	_SampleName	Estimate	StdErr	DF	tValue	Probt
574	2	Group*SampleName	Vegetable	Kale puree	Control	Broccoli chopped	-38.3776	13.7559	211	-2.79	0.0058
575	2	Group*SampleName	Vegetable	Kale puree	Control	Broccoli puree	-18.0026	13.7559	211	-1.31	0.1921
576	2	Group*SampleName	Vegetable	Kale puree	Control	Chicken puree	-13.3151	13.7559	211	-0.97	0.3342
577	2	Group*SampleName	Vegetable	Kale puree	Control	Kale chopped	6.7474	13.7559	211	0.49	0.6243
578	2	Group*SampleName	Vegetable	Kale puree	Control	Kale puree	5.9349	13.7559	211	0.43	0.6666
579	2	Group*SampleName	Vegetable	Kale puree	Control	Oat puree	-0.9401	13.7559	211	-0.07	0.9456
580	2	Group*SampleName	Vegetable	Kale puree	Control	Spinach chopped	-5.5026	13.7559	211	-0.40	0.6895
581	2	Group*SampleName	Vegetable	Kale puree	Control	Spinach puree	-3.3776	13.7559	211	-0.25	0.8063
582	2	Group*SampleName	Vegetable	Oat puree	Vegetable	Spinach chopped	3.5556	11.4303	352	0.31	0.7559
583	2	Group*SampleName	Vegetable	Oat puree	Vegetable	Spinach puree	9.5000	11.4303	352	0.83	0.4065
584	2	Group*SampleName	Vegetable	Oat puree	Control	Asparagus chopped	-14.5165	13.7559	211	-1.06	0.2925
585	2	Group*SampleName	Vegetable	Oat puree	Control	Asparagus puree	-19.3290	13.7559	211	-1.41	0.1615
586	2	Group*SampleName	Vegetable	Oat puree	Control	Beef puree	2.3585	13.7559	211	0.17	0.8640
587	2	Group*SampleName	Vegetable	Oat puree	Control	Black bean puree	-13.3915	13.7559	211	-0.97	0.3314
588	2	Group*SampleName	Vegetable	Oat puree	Control	Broccoli chopped	-26.2665	13.7559	211	-1.91	0.0576
589	2	Group*SampleName	Vegetable	Oat puree	Control	Broccoli puree	-5.8915	13.7559	211	-0.43	0.6689
590	2	Group*SampleName	Vegetable	Oat puree	Control	Chicken puree	-1.2040	13.7559	211	-0.09	0.9303
591	2	Group*SampleName	Vegetable	Oat puree	Control	Kale chopped	18.8585	13.7559	211	1.37	0.1719
592	2	Group*SampleName	Vegetable	Oat puree	Control	Kale puree	18.0460	13.7559	211	1.31	0.1910
593	2	Group*SampleName	Vegetable	Oat puree	Control	Oat puree	11.1710	13.7559	211	0.81	0.4177
594	2	Group*SampleName	Vegetable	Oat puree	Control	Spinach chopped	6.6085	13.7559	211	0.48	0.6314
595	2	Group*SampleName	Vegetable	Oat puree	Control	Spinach puree	8.7335	13.7559	211	0.63	0.5262
596	2	Group*SampleName	Vegetable	Spinach chopped	Vegetable	Spinach puree	5.9444	11.4303	352	0.52	0.6033
597	2	Group*SampleName	Vegetable	Spinach chopped	Control	Asparagus chopped	-18.0721	13.7559	211	-1.31	0.1904
598	2	Group*SampleName	Vegetable	Spinach chopped	Control	Asparagus puree	-22.8846	13.7559	211	-1.66	0.0977
599	2	Group*SampleName	Vegetable	Spinach chopped	Control	Beef puree	-1.1971	13.7559	211	-0.09	0.9307
600	2	Group*SampleName	Vegetable	Spinach chopped	Control	Black bean puree	-16.9471	13.7559	211	-1.23	0.2193
601	2	Group*SampleName	Vegetable	Spinach chopped	Control	Broccoli chopped	-29.8221	13.7559	211	-2.17	0.0313
602	2	Group*SampleName	Vegetable	Spinach chopped	Control	Broccoli puree	-9.4471	13.7559	211	-0.69	0.4930
603	2	Group*SampleName	Vegetable	Spinach chopped	Control	Chicken puree	-4.7596	13.7559	211	-0.35	0.7297
604	2	Group*SampleName	Vegetable	Spinach chopped	Control	Kale chopped	15.3029	13.7559	211	1.11	0.2672
605	2	Group*SampleName	Vegetable	Spinach chopped	Control	Kale puree	14.4904	13.7559	211	1.05	0.2934
606	2	Group*SampleName	Vegetable	Spinach chopped	Control	Oat puree	7.6154	13.7559	211	0.55	0.5804

Obs	GameVisit	Effect	Group	SampleName	_Group	_SampleName	Estimate	StdErr	DF	tValue	Probt
607	2	Group*SampleName	Vegetable	Spinach chopped	Control	Spinach chopped	3.0529	13.7559	211	0.22	0.8246
608	2	Group*SampleName	Vegetable	Spinach chopped	Control	Spinach puree	5.1779	13.7559	211	0.38	0.7070
609	2	Group*SampleName	Vegetable	Spinach puree	Control	Asparagus chopped	-24.0165	13.7559	211	-1.75	0.0823
610	2	Group*SampleName	Vegetable	Spinach puree	Control	Asparagus puree	-28.8290	13.7559	211	-2.10	0.0373
611	2	Group*SampleName	Vegetable	Spinach puree	Control	Beef puree	-7.1415	13.7559	211	-0.52	0.6042
612	2	Group*SampleName	Vegetable	Spinach puree	Control	Black bean puree	-22.8915	13.7559	211	-1.66	0.0976
613	2	Group*SampleName	Vegetable	Spinach puree	Control	Broccoli chopped	-35.7665	13.7559	211	-2.60	0.0100
614	2	Group*SampleName	Vegetable	Spinach puree	Control	Broccoli puree	-15.3915	13.7559	211	-1.12	0.2645
615	2	Group*SampleName	Vegetable	Spinach puree	Control	Chicken puree	-10.7040	13.7559	211	-0.78	0.4374
616	2	Group*SampleName	Vegetable	Spinach puree	Control	Kale chopped	9.3585	13.7559	211	0.68	0.4970
617	2	Group*SampleName	Vegetable	Spinach puree	Control	Kale puree	8.5460	13.7559	211	0.62	0.5351
618	2	Group*SampleName	Vegetable	Spinach puree	Control	Oat puree	1.6710	13.7559	211	0.12	0.9034
619	2	Group*SampleName	Vegetable	Spinach puree	Control	Spinach chopped	-2.8915	13.7559	211	-0.21	0.8337
620	2	Group*SampleName	Vegetable	Spinach puree	Control	Spinach puree	-0.7665	13.7559	211	-0.06	0.9556
621	2	Group*SampleName	Control	Asparagus chopped	Control	Asparagus puree	-4.8125	12.1236	352	-0.40	0.6916
622	2	Group*SampleName	Control	Asparagus chopped	Control	Beef puree	16.8750	12.1236	352	1.39	0.1648
623	2	Group*SampleName	Control	Asparagus chopped	Control	Black bean puree	1.1250	12.1236	352	0.09	0.9261
624	2	Group*SampleName	Control	Asparagus chopped	Control	Broccoli chopped	-11.7500	12.1236	352	-0.97	0.3331
625	2	Group*SampleName	Control	Asparagus chopped	Control	Broccoli puree	8.6250	12.1236	352	0.71	0.4773
626	2	Group*SampleName	Control	Asparagus chopped	Control	Chicken puree	13.3125	12.1236	352	1.10	0.2729
627	2	Group*SampleName	Control	Asparagus chopped	Control	Kale chopped	33.3750	12.1236	352	2.75	0.0062
628	2	Group*SampleName	Control	Asparagus chopped	Control	Kale puree	32.5625	12.1236	352	2.69	0.0076
629	2	Group*SampleName	Control	Asparagus chopped	Control	Oat puree	25.6875	12.1236	352	2.12	0.0348
630	2	Group*SampleName	Control	Asparagus chopped	Control	Spinach chopped	21.1250	12.1236	352	1.74	0.0823
631	2	Group*SampleName	Control	Asparagus chopped	Control	Spinach puree	23.2500	12.1236	352	1.92	0.0560
632	2	Group*SampleName	Control	Asparagus puree	Control	Beef puree	21.6875	12.1236	352	1.79	0.0745
633	2	Group*SampleName	Control	Asparagus puree	Control	Black bean puree	5.9375	12.1236	352	0.49	0.6246
634	2	Group*SampleName	Control	Asparagus puree	Control	Broccoli chopped	-6.9375	12.1236	352	-0.57	0.5675
635	2	Group*SampleName	Control	Asparagus	Control	Broccoli puree	13.4375	12.1236	352	1.11	0.2685

Obs	GameVisit	Effect	Group	SampleName	_Group	_SampleName	Estimate	StdErr	DF	tValue	Probt
				puree							
636	2	Group*SampleName	Control	Asparagus puree	Control	Chicken puree	18.1250	12.1236	352	1.50	0.1358
637	2	Group*SampleName	Control	Asparagus puree	Control	Kale chopped	38.1875	12.1236	352	3.15	0.0018
638	2	Group*SampleName	Control	Asparagus puree	Control	Kale puree	37.3750	12.1236	352	3.08	0.0022
639	2	Group*SampleName	Control	Asparagus puree	Control	Oat puree	30.5000	12.1236	352	2.52	0.0123
640	2	Group*SampleName	Control	Asparagus puree	Control	Spinach chopped	25.9375	12.1236	352	2.14	0.0331
641	2	Group*SampleName	Control	Asparagus puree	Control	Spinach puree	28.0625	12.1236	352	2.31	0.0212
642	2	Group*SampleName	Control	Beef puree	Control	Black bean puree	-15.7500	12.1236	352	-1.30	0.1948
643	2	Group*SampleName	Control	Beef puree	Control	Broccoli chopped	-28.6250	12.1236	352	-2.36	0.0188
644	2	Group*SampleName	Control	Beef puree	Control	Broccoli puree	-8.2500	12.1236	352	-0.68	0.4966
645	2	Group*SampleName	Control	Beef puree	Control	Chicken puree	-3.5625	12.1236	352	-0.29	0.7690
646	2	Group*SampleName	Control	Beef puree	Control	Kale chopped	16.5000	12.1236	352	1.36	0.1744
647	2	Group*SampleName	Control	Beef puree	Control	Kale puree	15.6875	12.1236	352	1.29	0.1965
648	2	Group*SampleName	Control	Beef puree	Control	Oat puree	8.8125	12.1236	352	0.73	0.4678
649	2	Group*SampleName	Control	Beef puree	Control	Spinach chopped	4.2500	12.1236	352	0.35	0.7261
650	2	Group*SampleName	Control	Beef puree	Control	Spinach puree	6.3750	12.1236	352	0.53	0.5993
651	2	Group*SampleName	Control	Black bean puree	Control	Broccoli chopped	-12.8750	12.1236	352	-1.06	0.2890
652	2	Group*SampleName	Control	Black bean puree	Control	Broccoli puree	7.5000	12.1236	352	0.62	0.5366
653	2	Group*SampleName	Control	Black bean puree	Control	Chicken puree	12.1875	12.1236	352	1.01	0.3155
654	2	Group*SampleName	Control	Black bean puree	Control	Kale chopped	32.2500	12.1236	352	2.66	0.0082
655	2	Group*SampleName	Control	Black bean puree	Control	Kale puree	31.4375	12.1236	352	2.59	0.0099
656	2	Group*SampleName	Control	Black bean puree	Control	Oat puree	24.5625	12.1236	352	2.03	0.0435
657	2	Group*SampleName	Control	Black bean puree	Control	Spinach chopped	20.0000	12.1236	352	1.65	0.0999
658	2	Group*SampleName	Control	Black bean puree	Control	Spinach puree	22.1250	12.1236	352	1.82	0.0689
659	2	Group*SampleName	Control	Broccoli chopped	Control	Broccoli puree	20.3750	12.1236	352	1.68	0.0937
660	2	Group*SampleName	Control	Broccoli chopped	Control	Chicken puree	25.0625	12.1236	352	2.07	0.0394
661	2	Group*SampleName	Control	Broccoli chopped	Control	Kale chopped	45.1250	12.1236	352	3.72	0.0002
662	2	Group*SampleName	Control	Broccoli chopped	Control	Kale puree	44.3125	12.1236	352	3.66	0.0003
663	2	Group*SampleName	Control	Broccoli chopped	Control	Oat puree	37.4375	12.1236	352	3.09	0.0022
664	2	Group*SampleName	Control	Broccoli chopped	Control	Spinach chopped	32.8750	12.1236	352	2.71	0.0070
665	2	Group*SampleName	Control	Broccoli chopped	Control	Spinach puree	35.0000	12.1236	352	2.89	0.0041

Obs	GameVisit	Effect	Group	SampleName	_Group	_SampleName	Estimate	StdErr	DF	tValue	Probt
666	2	Group*SampleName	Control	Broccoli puree	Control	Chicken puree	4.6875	12.1236	352	0.39	0.6993
667	2	Group*SampleName	Control	Broccoli puree	Control	Kale chopped	24.7500	12.1236	352	2.04	0.0419
668	2	Group*SampleName	Control	Broccoli puree	Control	Kale puree	23.9375	12.1236	352	1.97	0.0491
669	2	Group*SampleName	Control	Broccoli puree	Control	Oat puree	17.0625	12.1236	352	1.41	0.1602
670	2	Group*SampleName	Control	Broccoli puree	Control	Spinach chopped	12.5000	12.1236	352	1.03	0.3032
671	2	Group*SampleName	Control	Broccoli puree	Control	Spinach puree	14.6250	12.1236	352	1.21	0.2285
672	2	Group*SampleName	Control	Chicken puree	Control	Kale chopped	20.0625	12.1236	352	1.65	0.0989
673	2	Group*SampleName	Control	Chicken puree	Control	Kale puree	19.2500	12.1236	352	1.59	0.1132
674	2	Group*SampleName	Control	Chicken puree	Control	Oat puree	12.3750	12.1236	352	1.02	0.3081
675	2	Group*SampleName	Control	Chicken puree	Control	Spinach chopped	7.8125	12.1236	352	0.64	0.5197
676	2	Group*SampleName	Control	Chicken puree	Control	Spinach puree	9.9375	12.1236	352	0.82	0.4130
677	2	Group*SampleName	Control	Kale chopped	Control	Kale puree	-0.8125	12.1236	352	-0.07	0.9466
678	2	Group*SampleName	Control	Kale chopped	Control	Oat puree	-7.6875	12.1236	352	-0.63	0.5264
679	2	Group*SampleName	Control	Kale chopped	Control	Spinach chopped	-12.2500	12.1236	352	-1.01	0.3130
680	2	Group*SampleName	Control	Kale chopped	Control	Spinach puree	-10.1250	12.1236	352	-0.84	0.4042
681	2	Group*SampleName	Control	Kale puree	Control	Oat puree	-6.8750	12.1236	352	-0.57	0.5710
682	2	Group*SampleName	Control	Kale puree	Control	Spinach chopped	-11.4375	12.1236	352	-0.94	0.3461
683	2	Group*SampleName	Control	Kale puree	Control	Spinach puree	-9.3125	12.1236	352	-0.77	0.4429
684	2	Group*SampleName	Control	Oat puree	Control	Spinach chopped	-4.5625	12.1236	352	-0.38	0.7069
685	2	Group*SampleName	Control	Oat puree	Control	Spinach puree	-2.4375	12.1236	352	-0.20	0.8408
686	2	Group*SampleName	Control	Spinach chopped	Control	Spinach puree	2.1250	12.1236	352	0.18	0.8610
687	3	Group	Vegetable		Control		0.7829	9.3226	31	0.08	0.9336
688	3	SampleName		Asparagus chopped		Asparagus puree	-0.8819	7.9526	352	-0.11	0.9118
689	3	SampleName		Asparagus chopped		Beef puree	13.9306	7.9526	352	1.75	0.0807
690	3	SampleName		Asparagus chopped		Black bean puree	3.7951	7.9526	352	0.48	0.6335
691	3	SampleName		Asparagus chopped		Broccoli chopped	-9.4097	7.9526	352	-1.18	0.2375
692	3	SampleName		Asparagus chopped		Broccoli puree	-8.8160	7.9526	352	-1.11	0.2684
693	3	SampleName		Asparagus chopped		Chicken puree	14.3160	7.9526	352	1.80	0.0727
694	3	SampleName		Asparagus chopped		Kale chopped	23.6146	7.9526	352	2.97	0.0032
695	3	SampleName		Asparagus chopped		Kale puree	21.9097	7.9526	352	2.76	0.0062
696	3	SampleName		Asparagus chopped		Oat puree	3.8889	7.9526	352	0.49	0.6251

Obs	GameVisit	Effect	Group	SampleName	_Group	_SampleName	Estimate	StdErr	DF	tValue	Probt
697	3	SampleName		Asparagus chopped		Spinach chopped	5.2882	7.9526	352	0.66	0.5065
698	3	SampleName		Asparagus chopped		Spinach puree	17.0104	7.9526	352	2.14	0.0331
699	3	SampleName		Asparagus puree		Beef puree	14.8125	7.9526	352	1.86	0.0634
700	3	SampleName		Asparagus puree		Black bean puree	4.6771	7.9526	352	0.59	0.5568
701	3	SampleName		Asparagus puree		Broccoli chopped	-8.5278	7.9526	352	-1.07	0.2843
702	3	SampleName		Asparagus puree		Broccoli puree	-7.9340	7.9526	352	-1.00	0.3191
703	3	SampleName		Asparagus puree		Chicken puree	15.1979	7.9526	352	1.91	0.0568
704	3	SampleName		Asparagus puree		Kale chopped	24.4965	7.9526	352	3.08	0.0022
705	3	SampleName		Asparagus puree		Kale puree	22.7917	7.9526	352	2.87	0.0044
706	3	SampleName		Asparagus puree		Oat puree	4.7708	7.9526	352	0.60	0.5490
707	3	SampleName		Asparagus puree		Spinach chopped	6.1701	7.9526	352	0.78	0.4383
708	3	SampleName		Asparagus puree		Spinach puree	17.8924	7.9526	352	2.25	0.0251
709	3	SampleName		Beef puree		Black bean puree	-10.1354	7.9526	352	-1.27	0.2033
710	3	SampleName		Beef puree		Broccoli chopped	-23.3403	7.9526	352	-2.93	0.0036
711	3	SampleName		Beef puree		Broccoli puree	-22.7465	7.9526	352	-2.86	0.0045
712	3	SampleName		Beef puree		Chicken puree	0.3854	7.9526	352	0.05	0.9614
713	3	SampleName		Beef puree		Kale chopped	9.6840	7.9526	352	1.22	0.2241
714	3	SampleName		Beef puree		Kale puree	7.9792	7.9526	352	1.00	0.3164
715	3	SampleName		Beef puree		Oat puree	-10.0417	7.9526	352	-1.26	0.2075
716	3	SampleName		Beef puree		Spinach chopped	-8.6424	7.9526	352	-1.09	0.2779
717	3	SampleName		Beef puree		Spinach puree	3.0799	7.9526	352	0.39	0.6988
718	3	SampleName		Black bean puree		Broccoli chopped	-13.2049	7.9526	352	-1.66	0.0977
719	3	SampleName		Black bean puree		Broccoli puree	-12.6111	7.9526	352	-1.59	0.1137
720	3	SampleName		Black bean puree		Chicken puree	10.5208	7.9526	352	1.32	0.1867
721	3	SampleName		Black bean puree		Kale chopped	19.8194	7.9526	352	2.49	0.0132
722	3	SampleName		Black bean puree		Kale puree	18.1146	7.9526	352	2.28	0.0233
723	3	SampleName		Black bean puree		Oat puree	0.09375	7.9526	352	0.01	0.9906
724	3	SampleName		Black bean puree		Spinach chopped	1.4931	7.9526	352	0.19	0.8512
725	3	SampleName		Black bean puree		Spinach puree	13.2153	7.9526	352	1.66	0.0975
726	3	SampleName		Broccoli chopped		Broccoli puree	0.5937	7.9526	352	0.07	0.9405
727	3	SampleName		Broccoli		Chicken puree	23.7257	7.9526	352	2.98	0.0030

Obs	GameVisit	Effect	Group	SampleName	_Group	_SampleName	Estimate	StdErr	DF	tValue	Probt
				chopped							
728	3	SampleName		Broccoli chopped		Kale chopped	33.0243	7.9526	352	4.15	<.0001
729	3	SampleName		Broccoli chopped		Kale puree	31.3194	7.9526	352	3.94	<.0001
730	3	SampleName		Broccoli chopped		Oat puree	13.2986	7.9526	352	1.67	0.0954
731	3	SampleName		Broccoli chopped		Spinach chopped	14.6979	7.9526	352	1.85	0.0654
732	3	SampleName		Broccoli chopped		Spinach puree	26.4201	7.9526	352	3.32	0.0010
733	3	SampleName		Broccoli puree		Chicken puree	23.1319	7.9526	352	2.91	0.0039
734	3	SampleName		Broccoli puree		Kale chopped	32.4306	7.9526	352	4.08	<.0001
735	3	SampleName		Broccoli puree		Kale puree	30.7257	7.9526	352	3.86	0.0001
736	3	SampleName		Broccoli puree		Oat puree	12.7049	7.9526	352	1.60	0.1110
737	3	SampleName		Broccoli puree		Spinach chopped	14.1042	7.9526	352	1.77	0.0770
738	3	SampleName		Broccoli puree		Spinach puree	25.8264	7.9526	352	3.25	0.0013
739	3	SampleName		Chicken puree		Kale chopped	9.2986	7.9526	352	1.17	0.2431
740	3	SampleName		Chicken puree		Kale puree	7.5938	7.9526	352	0.95	0.3403
741	3	SampleName		Chicken puree		Oat puree	-10.4271	7.9526	352	-1.31	0.1907
742	3	SampleName		Chicken puree		Spinach chopped	-9.0278	7.9526	352	-1.14	0.2571
743	3	SampleName		Chicken puree		Spinach puree	2.6944	7.9526	352	0.34	0.7350
744	3	SampleName		Kale chopped		Kale puree	-1.7049	7.9526	352	-0.21	0.8304
745	3	SampleName		Kale chopped		Oat puree	-19.7257	7.9526	352	-2.48	0.0136
746	3	SampleName		Kale chopped		Spinach chopped	-18.3264	7.9526	352	-2.30	0.0218
747	3	SampleName		Kale chopped		Spinach puree	-6.6042	7.9526	352	-0.83	0.4069
748	3	SampleName		Kale puree		Oat puree	-18.0208	7.9526	352	-2.27	0.0241
749	3	SampleName		Kale puree		Spinach chopped	-16.6215	7.9526	352	-2.09	0.0373
750	3	SampleName		Kale puree		Spinach puree	-4.8993	7.9526	352	-0.62	0.5382
751	3	SampleName		Oat puree		Spinach chopped	1.3993	7.9526	352	0.18	0.8604
752	3	SampleName		Oat puree		Spinach puree	13.1215	7.9526	352	1.65	0.0998
753	3	SampleName		Spinach chopped		Spinach puree	11.7222	7.9526	352	1.47	0.1414
754	3	Group*SampleName	Vegetable	Asparagus chopped	Vegetable	Asparagus puree	-3.8889	10.9108	352	-0.36	0.7217
755	3	Group*SampleName	Vegetable	Asparagus chopped	Vegetable	Beef puree	14.6111	10.9108	352	1.34	0.1814
756	3	Group*SampleName	Vegetable	Asparagus chopped	Vegetable	Black bean puree	3.2778	10.9108	352	0.30	0.7640
757	3	Group*SampleName	Vegetable	Asparagus chopped	Vegetable	Broccoli chopped	-12.9444	10.9108	352	-1.19	0.2363

Obs	GameVisit	Effect	Group	SampleName	_Group	_SampleName	Estimate	StdErr	DF	tValue	Probt
758	3	Group*SampleName	Vegetable	Asparagus chopped	Vegetable	Broccoli puree	-18.9444	10.9108	352	-1.74	0.0834
759	3	Group*SampleName	Vegetable	Asparagus chopped	Vegetable	Chicken puree	14.4444	10.9108	352	1.32	0.1864
760	3	Group*SampleName	Vegetable	Asparagus chopped	Vegetable	Kale chopped	10.6667	10.9108	352	0.98	0.3289
761	3	Group*SampleName	Vegetable	Asparagus chopped	Vegetable	Kale puree	5.9444	10.9108	352	0.54	0.5862
762	3	Group*SampleName	Vegetable	Asparagus chopped	Vegetable	Oat puree	-13.7222	10.9108	352	-1.26	0.2093
763	3	Group*SampleName	Vegetable	Asparagus chopped	Vegetable	Spinach chopped	-7.6111	10.9108	352	-0.70	0.4859
764	3	Group*SampleName	Vegetable	Asparagus chopped	Vegetable	Spinach puree	5.8333	10.9108	352	0.53	0.5932
765	3	Group*SampleName	Vegetable	Asparagus chopped	Control	Asparagus chopped	-13.7136	14.2428	146	-0.96	0.3372
766	3	Group*SampleName	Vegetable	Asparagus chopped	Control	Asparagus puree	-11.5886	14.2428	146	-0.81	0.4172
767	3	Group*SampleName	Vegetable	Asparagus chopped	Control	Beef puree	-0.4636	14.2428	146	-0.03	0.9741
768	3	Group*SampleName	Vegetable	Asparagus chopped	Control	Black bean puree	-9.4011	14.2428	146	-0.66	0.5103
769	3	Group*SampleName	Vegetable	Asparagus chopped	Control	Broccoli chopped	-19.5886	14.2428	146	-1.38	0.1711
770	3	Group*SampleName	Vegetable	Asparagus chopped	Control	Broccoli puree	-12.4011	14.2428	146	-0.87	0.3854
771	3	Group*SampleName	Vegetable	Asparagus chopped	Control	Chicken puree	0.4739	14.2428	146	0.03	0.9735
772	3	Group*SampleName	Vegetable	Asparagus chopped	Control	Kale chopped	22.8489	14.2428	146	1.60	0.1108
773	3	Group*SampleName	Vegetable	Asparagus chopped	Control	Kale puree	24.1614	14.2428	146	1.70	0.0919
774	3	Group*SampleName	Vegetable	Asparagus chopped	Control	Oat puree	7.7864	14.2428	146	0.55	0.5854
775	3	Group*SampleName	Vegetable	Asparagus chopped	Control	Spinach chopped	4.4739	14.2428	146	0.31	0.7539
776	3	Group*SampleName	Vegetable	Asparagus chopped	Control	Spinach puree	14.4739	14.2428	146	1.02	0.3112
777	3	Group*SampleName	Vegetable	Asparagus puree	Vegetable	Beef puree	18.5000	10.9108	352	1.70	0.0909
778	3	Group*SampleName	Vegetable	Asparagus puree	Vegetable	Black bean puree	7.1667	10.9108	352	0.66	0.5117
779	3	Group*SampleName	Vegetable	Asparagus puree	Vegetable	Broccoli chopped	-9.0556	10.9108	352	-0.83	0.4071
780	3	Group*SampleName	Vegetable	Asparagus puree	Vegetable	Broccoli puree	-15.0556	10.9108	352	-1.38	0.1685
781	3	Group*SampleName	Vegetable	Asparagus puree	Vegetable	Chicken puree	18.3333	10.9108	352	1.68	0.0938
782	3	Group*SampleName	Vegetable	Asparagus puree	Vegetable	Kale chopped	14.5556	10.9108	352	1.33	0.1831
783	3	Group*SampleName	Vegetable	Asparagus puree	Vegetable	Kale puree	9.8333	10.9108	352	0.90	0.3681
784	3	Group*SampleName	Vegetable	Asparagus puree	Vegetable	Oat puree	-9.8333	10.9108	352	-0.90	0.3681
785	3	Group*SampleName	Vegetable	Asparagus puree	Vegetable	Spinach chopped	-3.7222	10.9108	352	-0.34	0.7332
786	3	Group*SampleName	Vegetable	Asparagus	Vegetable	Spinach puree	9.7222	10.9108	352	0.89	0.3735

Obs	GameVisit	Effect	Group	SampleName	_Group	_SampleName	Estimate	StdErr	DF	tValue	Probt
				puree							
787	3	Group*SampleName	Vegetable	Asparagus puree	Control	Asparagus chopped	-9.8247	14.2428	146	-0.69	0.4914
788	3	Group*SampleName	Vegetable	Asparagus puree	Control	Asparagus puree	-7.6997	14.2428	146	-0.54	0.5896
789	3	Group*SampleName	Vegetable	Asparagus puree	Control	Beef puree	3.4253	14.2428	146	0.24	0.8103
790	3	Group*SampleName	Vegetable	Asparagus puree	Control	Black bean puree	-5.5122	14.2428	146	-0.39	0.6993
791	3	Group*SampleName	Vegetable	Asparagus puree	Control	Broccoli chopped	-15.6997	14.2428	146	-1.10	0.2721
792	3	Group*SampleName	Vegetable	Asparagus puree	Control	Broccoli puree	-8.5122	14.2428	146	-0.60	0.5510
793	3	Group*SampleName	Vegetable	Asparagus puree	Control	Chicken puree	4.3628	14.2428	146	0.31	0.7598
794	3	Group*SampleName	Vegetable	Asparagus puree	Control	Kale chopped	26.7378	14.2428	146	1.88	0.0625
795	3	Group*SampleName	Vegetable	Asparagus puree	Control	Kale puree	28.0503	14.2428	146	1.97	0.0508
796	3	Group*SampleName	Vegetable	Asparagus puree	Control	Oat puree	11.6753	14.2428	146	0.82	0.4137
797	3	Group*SampleName	Vegetable	Asparagus puree	Control	Spinach chopped	8.3628	14.2428	146	0.59	0.5580
798	3	Group*SampleName	Vegetable	Asparagus puree	Control	Spinach puree	18.3628	14.2428	146	1.29	0.1993
799	3	Group*SampleName	Vegetable	Beef puree	Vegetable	Black bean puree	-11.3333	10.9108	352	-1.04	0.2996
800	3	Group*SampleName	Vegetable	Beef puree	Vegetable	Broccoli chopped	-27.5556	10.9108	352	-2.53	0.0120
801	3	Group*SampleName	Vegetable	Beef puree	Vegetable	Broccoli puree	-33.5556	10.9108	352	-3.08	0.0023
802	3	Group*SampleName	Vegetable	Beef puree	Vegetable	Chicken puree	-0.1667	10.9108	352	-0.02	0.9878
803	3	Group*SampleName	Vegetable	Beef puree	Vegetable	Kale chopped	-3.9444	10.9108	352	-0.36	0.7179
804	3	Group*SampleName	Vegetable	Beef puree	Vegetable	Kale puree	-8.6667	10.9108	352	-0.79	0.4275
805	3	Group*SampleName	Vegetable	Beef puree	Vegetable	Oat puree	-28.3333	10.9108	352	-2.60	0.0098
806	3	Group*SampleName	Vegetable	Beef puree	Vegetable	Spinach chopped	-22.2222	10.9108	352	-2.04	0.0424
807	3	Group*SampleName	Vegetable	Beef puree	Vegetable	Spinach puree	-8.7778	10.9108	352	-0.80	0.4217
808	3	Group*SampleName	Vegetable	Beef puree	Control	Asparagus chopped	-28.3247	14.2428	146	-1.99	0.0486
809	3	Group*SampleName	Vegetable	Beef puree	Control	Asparagus puree	-26.1997	14.2428	146	-1.84	0.0679
810	3	Group*SampleName	Vegetable	Beef puree	Control	Beef puree	-15.0747	14.2428	146	-1.06	0.2916
811	3	Group*SampleName	Vegetable	Beef puree	Control	Black bean puree	-24.0122	14.2428	146	-1.69	0.0939
812	3	Group*SampleName	Vegetable	Beef puree	Control	Broccoli chopped	-34.1997	14.2428	146	-2.40	0.0176
813	3	Group*SampleName	Vegetable	Beef puree	Control	Broccoli puree	-27.0122	14.2428	146	-1.90	0.0599
814	3	Group*SampleName	Vegetable	Beef puree	Control	Chicken puree	-14.1372	14.2428	146	-0.99	0.3226
815	3	Group*SampleName	Vegetable	Beef puree	Control	Kale chopped	8.2378	14.2428	146	0.58	0.5639
816	3	Group*SampleName	Vegetable	Beef puree	Control	Kale puree	9.5503	14.2428	146	0.67	0.5036
817	3	Group*SampleName	Vegetable	Beef puree	Control	Oat puree	-6.8247	14.2428	146	-0.48	0.6325
818	3	Group*SampleName	Vegetable	Beef puree	Control	Spinach chopped	-10.1372	14.2428	146	-0.71	0.4778

Obs	GameVisit	Effect	Group	SampleName	_Group	_SampleName	Estimate	StdErr	DF	tValue	Probt
819	3	Group*SampleName	Vegetable	Beef puree	Control	Spinach puree	-0.1372	14.2428	146	-0.01	0.9923
820	3	Group*SampleName	Vegetable	Black bean puree	Vegetable	Broccoli chopped	-16.2222	10.9108	352	-1.49	0.1380
821	3	Group*SampleName	Vegetable	Black bean puree	Vegetable	Broccoli puree	-22.2222	10.9108	352	-2.04	0.0424
822	3	Group*SampleName	Vegetable	Black bean puree	Vegetable	Chicken puree	11.1667	10.9108	352	1.02	0.3068
823	3	Group*SampleName	Vegetable	Black bean puree	Vegetable	Kale chopped	7.3889	10.9108	352	0.68	0.4987
824	3	Group*SampleName	Vegetable	Black bean puree	Vegetable	Kale puree	2.6667	10.9108	352	0.24	0.8071
825	3	Group*SampleName	Vegetable	Black bean puree	Vegetable	Oat puree	-17.0000	10.9108	352	-1.56	0.1201
826	3	Group*SampleName	Vegetable	Black bean puree	Vegetable	Spinach chopped	-10.8889	10.9108	352	-1.00	0.3190
827	3	Group*SampleName	Vegetable	Black bean puree	Vegetable	Spinach puree	2.5556	10.9108	352	0.23	0.8149
828	3	Group*SampleName	Vegetable	Black bean puree	Control	Asparagus chopped	-16.9914	14.2428	146	-1.19	0.2348
829	3	Group*SampleName	Vegetable	Black bean puree	Control	Asparagus puree	-14.8664	14.2428	146	-1.04	0.2983
830	3	Group*SampleName	Vegetable	Black bean puree	Control	Beef puree	-3.7414	14.2428	146	-0.26	0.7932
831	3	Group*SampleName	Vegetable	Black bean puree	Control	Black bean puree	-12.6789	14.2428	146	-0.89	0.3748
832	3	Group*SampleName	Vegetable	Black bean puree	Control	Broccoli chopped	-22.8664	14.2428	146	-1.61	0.1106
833	3	Group*SampleName	Vegetable	Black bean puree	Control	Broccoli puree	-15.6789	14.2428	146	-1.10	0.2728
834	3	Group*SampleName	Vegetable	Black bean puree	Control	Chicken puree	-2.8039	14.2428	146	-0.20	0.8442
835	3	Group*SampleName	Vegetable	Black bean puree	Control	Kale chopped	19.5711	14.2428	146	1.37	0.1715
836	3	Group*SampleName	Vegetable	Black bean puree	Control	Kale puree	20.8836	14.2428	146	1.47	0.1447
837	3	Group*SampleName	Vegetable	Black bean puree	Control	Oat puree	4.5086	14.2428	146	0.32	0.7520
838	3	Group*SampleName	Vegetable	Black bean puree	Control	Spinach chopped	1.1961	14.2428	146	0.08	0.9332
839	3	Group*SampleName	Vegetable	Black bean puree	Control	Spinach puree	11.1961	14.2428	146	0.79	0.4331
840	3	Group*SampleName	Vegetable	Broccoli chopped	Vegetable	Broccoli puree	-6.0000	10.9108	352	-0.55	0.5827
841	3	Group*SampleName	Vegetable	Broccoli chopped	Vegetable	Chicken puree	27.3889	10.9108	352	2.51	0.0125
842	3	Group*SampleName	Vegetable	Broccoli chopped	Vegetable	Kale chopped	23.6111	10.9108	352	2.16	0.0311
843	3	Group*SampleName	Vegetable	Broccoli chopped	Vegetable	Kale puree	18.8889	10.9108	352	1.73	0.0843
844	3	Group*SampleName	Vegetable	Broccoli chopped	Vegetable	Oat puree	-0.7778	10.9108	352	-0.07	0.9432
845	3	Group*SampleName	Vegetable	Broccoli chopped	Vegetable	Spinach chopped	5.3333	10.9108	352	0.49	0.6253
846	3	Group*SampleName	Vegetable	Broccoli chopped	Vegetable	Spinach puree	18.7778	10.9108	352	1.72	0.0861
847	3	Group*SampleName	Vegetable	Broccoli chopped	Control	Asparagus chopped	-0.7692	14.2428	146	-0.05	0.9570

Obs	GameVisit	Effect	Group	SampleName	_Group	_SampleName	Estimate	StdErr	DF	tValue	Probt
848	3	Group*SampleName	Vegetable	Broccoli chopped	Control	Asparagus puree	1.3558	14.2428	146	0.10	0.9243
849	3	Group*SampleName	Vegetable	Broccoli chopped	Control	Beef puree	12.4808	14.2428	146	0.88	0.3823
850	3	Group*SampleName	Vegetable	Broccoli chopped	Control	Black bean puree	3.5433	14.2428	146	0.25	0.8039
851	3	Group*SampleName	Vegetable	Broccoli chopped	Control	Broccoli chopped	-6.6442	14.2428	146	-0.47	0.6416
852	3	Group*SampleName	Vegetable	Broccoli chopped	Control	Broccoli puree	0.5433	14.2428	146	0.04	0.9696
853	3	Group*SampleName	Vegetable	Broccoli chopped	Control	Chicken puree	13.4183	14.2428	146	0.94	0.3477
854	3	Group*SampleName	Vegetable	Broccoli chopped	Control	Kale chopped	35.7933	14.2428	146	2.51	0.0131
855	3	Group*SampleName	Vegetable	Broccoli chopped	Control	Kale puree	37.1058	14.2428	146	2.61	0.0101
856	3	Group*SampleName	Vegetable	Broccoli chopped	Control	Oat puree	20.7308	14.2428	146	1.46	0.1477
857	3	Group*SampleName	Vegetable	Broccoli chopped	Control	Spinach chopped	17.4183	14.2428	146	1.22	0.2233
858	3	Group*SampleName	Vegetable	Broccoli chopped	Control	Spinach puree	27.4183	14.2428	146	1.93	0.0562
859	3	Group*SampleName	Vegetable	Broccoli puree	Vegetable	Chicken puree	33.3889	10.9108	352	3.06	0.0024
860	3	Group*SampleName	Vegetable	Broccoli puree	Vegetable	Kale chopped	29.6111	10.9108	352	2.71	0.0070
861	3	Group*SampleName	Vegetable	Broccoli puree	Vegetable	Kale puree	24.8889	10.9108	352	2.28	0.0231
862	3	Group*SampleName	Vegetable	Broccoli puree	Vegetable	Oat puree	5.2222	10.9108	352	0.48	0.6325
863	3	Group*SampleName	Vegetable	Broccoli puree	Vegetable	Spinach chopped	11.3333	10.9108	352	1.04	0.2996
864	3	Group*SampleName	Vegetable	Broccoli puree	Vegetable	Spinach puree	24.7778	10.9108	352	2.27	0.0238
865	3	Group*SampleName	Vegetable	Broccoli puree	Control	Asparagus chopped	5.2308	14.2428	146	0.37	0.7140
866	3	Group*SampleName	Vegetable	Broccoli puree	Control	Asparagus puree	7.3558	14.2428	146	0.52	0.6063
867	3	Group*SampleName	Vegetable	Broccoli puree	Control	Beef puree	18.4808	14.2428	146	1.30	0.1965
868	3	Group*SampleName	Vegetable	Broccoli puree	Control	Black bean puree	9.5433	14.2428	146	0.67	0.5039
869	3	Group*SampleName	Vegetable	Broccoli puree	Control	Broccoli chopped	-0.6442	14.2428	146	-0.05	0.9640
870	3	Group*SampleName	Vegetable	Broccoli puree	Control	Broccoli puree	6.5433	14.2428	146	0.46	0.6466
871	3	Group*SampleName	Vegetable	Broccoli puree	Control	Chicken puree	19.4183	14.2428	146	1.36	0.1749
872	3	Group*SampleName	Vegetable	Broccoli puree	Control	Kale chopped	41.7933	14.2428	146	2.93	0.0039
873	3	Group*SampleName	Vegetable	Broccoli puree	Control	Kale puree	43.1058	14.2428	146	3.03	0.0029
874	3	Group*SampleName	Vegetable	Broccoli puree	Control	Oat puree	26.7308	14.2428	146	1.88	0.0625
875	3	Group*SampleName	Vegetable	Broccoli puree	Control	Spinach chopped	23.4183	14.2428	146	1.64	0.1023
876	3	Group*SampleName	Vegetable	Broccoli	Control	Spinach puree	33.4183	14.2428	146	2.35	0.0203

Obs	GameVisit	Effect	Group	SampleName	_Group	_SampleName	Estimate	StdErr	DF	tValue	Probt
				puree							
877	3	Group*SampleName	Vegetable	Chicken puree	Vegetable	Kale chopped	-3.7778	10.9108	352	-0.35	0.7294
878	3	Group*SampleName	Vegetable	Chicken puree	Vegetable	Kale puree	-8.5000	10.9108	352	-0.78	0.4365
879	3	Group*SampleName	Vegetable	Chicken puree	Vegetable	Oat puree	-28.1667	10.9108	352	-2.58	0.0102
880	3	Group*SampleName	Vegetable	Chicken puree	Vegetable	Spinach chopped	-22.0556	10.9108	352	-2.02	0.0440
881	3	Group*SampleName	Vegetable	Chicken puree	Vegetable	Spinach puree	-8.6111	10.9108	352	-0.79	0.4305
882	3	Group*SampleName	Vegetable	Chicken puree	Control	Asparagus chopped	-28.1581	14.2428	146	-1.98	0.0499
883	3	Group*SampleName	Vegetable	Chicken puree	Control	Asparagus puree	-26.0331	14.2428	146	-1.83	0.0696
884	3	Group*SampleName	Vegetable	Chicken puree	Control	Beef puree	-14.9081	14.2428	146	-1.05	0.2970
885	3	Group*SampleName	Vegetable	Chicken puree	Control	Black bean puree	-23.8456	14.2428	146	-1.67	0.0962
886	3	Group*SampleName	Vegetable	Chicken puree	Control	Broccoli chopped	-34.0331	14.2428	146	-2.39	0.0181
887	3	Group*SampleName	Vegetable	Chicken puree	Control	Broccoli puree	-26.8456	14.2428	146	-1.88	0.0614
888	3	Group*SampleName	Vegetable	Chicken puree	Control	Chicken puree	-13.9706	14.2428	146	-0.98	0.3283
889	3	Group*SampleName	Vegetable	Chicken puree	Control	Kale chopped	8.4044	14.2428	146	0.59	0.5560
890	3	Group*SampleName	Vegetable	Chicken puree	Control	Kale puree	9.7169	14.2428	146	0.68	0.4962
891	3	Group*SampleName	Vegetable	Chicken puree	Control	Oat puree	-6.6581	14.2428	146	-0.47	0.6409
892	3	Group*SampleName	Vegetable	Chicken puree	Control	Spinach chopped	-9.9706	14.2428	146	-0.70	0.4850
893	3	Group*SampleName	Vegetable	Chicken puree	Control	Spinach puree	0.02942	14.2428	146	0.00	0.9984
894	3	Group*SampleName	Vegetable	Kale chopped	Vegetable	Kale puree	-4.7222	10.9108	352	-0.43	0.6654
895	3	Group*SampleName	Vegetable	Kale chopped	Vegetable	Oat puree	-24.3889	10.9108	352	-2.24	0.0260
896	3	Group*SampleName	Vegetable	Kale chopped	Vegetable	Spinach chopped	-18.2778	10.9108	352	-1.68	0.0948
897	3	Group*SampleName	Vegetable	Kale chopped	Vegetable	Spinach puree	-4.8333	10.9108	352	-0.44	0.6580
898	3	Group*SampleName	Vegetable	Kale chopped	Control	Asparagus chopped	-24.3803	14.2428	146	-1.71	0.0891
899	3	Group*SampleName	Vegetable	Kale chopped	Control	Asparagus puree	-22.2553	14.2428	146	-1.56	0.1203
900	3	Group*SampleName	Vegetable	Kale chopped	Control	Beef puree	-11.1303	14.2428	146	-0.78	0.4358
901	3	Group*SampleName	Vegetable	Kale chopped	Control	Black bean puree	-20.0678	14.2428	146	-1.41	0.1610
902	3	Group*SampleName	Vegetable	Kale chopped	Control	Broccoli chopped	-30.2553	14.2428	146	-2.12	0.0353
903	3	Group*SampleName	Vegetable	Kale chopped	Control	Broccoli puree	-23.0678	14.2428	146	-1.62	0.1075
904	3	Group*SampleName	Vegetable	Kale chopped	Control	Chicken puree	-10.1928	14.2428	146	-0.72	0.4754
905	3	Group*SampleName	Vegetable	Kale chopped	Control	Kale chopped	12.1822	14.2428	146	0.86	0.3938
906	3	Group*SampleName	Vegetable	Kale chopped	Control	Kale puree	13.4947	14.2428	146	0.95	0.3450
907	3	Group*SampleName	Vegetable	Kale chopped	Control	Oat puree	-2.8803	14.2428	146	-0.20	0.8400

Obs	GameVisit	Effect	Group	SampleName	_Group	_SampleName	Estimate	StdErr	DF	tValue	Probt
908	3	Group*SampleName	Vegetable	Kale chopped	Control	Spinach chopped	-6.1928	14.2428	146	-0.43	0.6643
909	3	Group*SampleName	Vegetable	Kale chopped	Control	Spinach puree	3.8072	14.2428	146	0.27	0.7896
910	3	Group*SampleName	Vegetable	Kale puree	Vegetable	Oat puree	-19.6667	10.9108	352	-1.80	0.0723
911	3	Group*SampleName	Vegetable	Kale puree	Vegetable	Spinach chopped	-13.5556	10.9108	352	-1.24	0.2149
912	3	Group*SampleName	Vegetable	Kale puree	Vegetable	Spinach puree	-0.1111	10.9108	352	-0.01	0.9919
913	3	Group*SampleName	Vegetable	Kale puree	Control	Asparagus chopped	-19.6581	14.2428	146	-1.38	0.1696
914	3	Group*SampleName	Vegetable	Kale puree	Control	Asparagus puree	-17.5331	14.2428	146	-1.23	0.2203
915	3	Group*SampleName	Vegetable	Kale puree	Control	Beef puree	-6.4081	14.2428	146	-0.45	0.6534
916	3	Group*SampleName	Vegetable	Kale puree	Control	Black bean puree	-15.3456	14.2428	146	-1.08	0.2831
917	3	Group*SampleName	Vegetable	Kale puree	Control	Broccoli chopped	-25.5331	14.2428	146	-1.79	0.0751
918	3	Group*SampleName	Vegetable	Kale puree	Control	Broccoli puree	-18.3456	14.2428	146	-1.29	0.1998
919	3	Group*SampleName	Vegetable	Kale puree	Control	Chicken puree	-5.4706	14.2428	146	-0.38	0.7015
920	3	Group*SampleName	Vegetable	Kale puree	Control	Kale chopped	16.9044	14.2428	146	1.19	0.2372
921	3	Group*SampleName	Vegetable	Kale puree	Control	Kale puree	18.2169	14.2428	146	1.28	0.2029
922	3	Group*SampleName	Vegetable	Kale puree	Control	Oat puree	1.8419	14.2428	146	0.13	0.8973
923	3	Group*SampleName	Vegetable	Kale puree	Control	Spinach chopped	-1.4706	14.2428	146	-0.10	0.9179
924	3	Group*SampleName	Vegetable	Kale puree	Control	Spinach puree	8.5294	14.2428	146	0.60	0.5502
925	3	Group*SampleName	Vegetable	Oat puree	Vegetable	Spinach chopped	6.1111	10.9108	352	0.56	0.5758
926	3	Group*SampleName	Vegetable	Oat puree	Vegetable	Spinach puree	19.5556	10.9108	352	1.79	0.0739
927	3	Group*SampleName	Vegetable	Oat puree	Control	Asparagus chopped	0.008589	14.2428	146	0.00	0.9995
928	3	Group*SampleName	Vegetable	Oat puree	Control	Asparagus puree	2.1336	14.2428	146	0.15	0.8811
929	3	Group*SampleName	Vegetable	Oat puree	Control	Beef puree	13.2586	14.2428	146	0.93	0.3534
930	3	Group*SampleName	Vegetable	Oat puree	Control	Black bean puree	4.3211	14.2428	146	0.30	0.7620
931	3	Group*SampleName	Vegetable	Oat puree	Control	Broccoli chopped	-5.8664	14.2428	146	-0.41	0.6810
932	3	Group*SampleName	Vegetable	Oat puree	Control	Broccoli puree	1.3211	14.2428	146	0.09	0.9262
933	3	Group*SampleName	Vegetable	Oat puree	Control	Chicken puree	14.1961	14.2428	146	1.00	0.3206
934	3	Group*SampleName	Vegetable	Oat puree	Control	Kale chopped	36.5711	14.2428	146	2.57	0.0112
935	3	Group*SampleName	Vegetable	Oat puree	Control	Kale puree	37.8836	14.2428	146	2.66	0.0087
936	3	Group*SampleName	Vegetable	Oat puree	Control	Oat puree	21.5086	14.2428	146	1.51	0.1332
937	3	Group*SampleName	Vegetable	Oat puree	Control	Spinach chopped	18.1961	14.2428	146	1.28	0.2034
938	3	Group*SampleName	Vegetable	Oat puree	Control	Spinach puree	28.1961	14.2428	146	1.98	0.0496
939	3	Group*SampleName	Vegetable	Spinach chopped	Vegetable	Spinach puree	13.4444	10.9108	352	1.23	0.2187
940	3	Group*SampleName	Vegetable	Spinach chopped	Control	Asparagus chopped	-6.1025	14.2428	146	-0.43	0.6689
941	3	Group*SampleName	Vegetable	Spinach chopped	Control	Asparagus puree	-3.9775	14.2428	146	-0.28	0.7804
942	3	Group*SampleName	Vegetable	Spinach chopped	Control	Beef puree	7.1475	14.2428	146	0.50	0.6165

Obs	GameVisit	Effect	Group	SampleName	_Group	_SampleName	Estimate	StdErr	DF	tValue	Probt
943	3	Group*SampleName	Vegetable	Spinach chopped	Control	Black bean puree	-1.7900	14.2428	146	-0.13	0.9002
944	3	Group*SampleName	Vegetable	Spinach chopped	Control	Broccoli chopped	-11.9775	14.2428	146	-0.84	0.4018
945	3	Group*SampleName	Vegetable	Spinach chopped	Control	Broccoli puree	-4.7900	14.2428	146	-0.34	0.7371
946	3	Group*SampleName	Vegetable	Spinach chopped	Control	Chicken puree	8.0850	14.2428	146	0.57	0.5711
947	3	Group*SampleName	Vegetable	Spinach chopped	Control	Kale chopped	30.4600	14.2428	146	2.14	0.0341
948	3	Group*SampleName	Vegetable	Spinach chopped	Control	Kale puree	31.7725	14.2428	146	2.23	0.0272
949	3	Group*SampleName	Vegetable	Spinach chopped	Control	Oat puree	15.3975	14.2428	146	1.08	0.2814
950	3	Group*SampleName	Vegetable	Spinach chopped	Control	Spinach chopped	12.0850	14.2428	146	0.85	0.3976
951	3	Group*SampleName	Vegetable	Spinach chopped	Control	Spinach puree	22.0850	14.2428	146	1.55	0.1232
952	3	Group*SampleName	Vegetable	Spinach puree	Control	Asparagus chopped	-19.5470	14.2428	146	-1.37	0.1720
953	3	Group*SampleName	Vegetable	Spinach puree	Control	Asparagus puree	-17.4220	14.2428	146	-1.22	0.2232
954	3	Group*SampleName	Vegetable	Spinach puree	Control	Beef puree	-6.2970	14.2428	146	-0.44	0.6591
955	3	Group*SampleName	Vegetable	Spinach puree	Control	Black bean puree	-15.2345	14.2428	146	-1.07	0.2866
956	3	Group*SampleName	Vegetable	Spinach puree	Control	Broccoli chopped	-25.4220	14.2428	146	-1.78	0.0764
957	3	Group*SampleName	Vegetable	Spinach puree	Control	Broccoli puree	-18.2345	14.2428	146	-1.28	0.2025
958	3	Group*SampleName	Vegetable	Spinach puree	Control	Chicken puree	-5.3595	14.2428	146	-0.38	0.7072
959	3	Group*SampleName	Vegetable	Spinach puree	Control	Kale chopped	17.0155	14.2428	146	1.19	0.2342
960	3	Group*SampleName	Vegetable	Spinach puree	Control	Kale puree	18.3280	14.2428	146	1.29	0.2002
961	3	Group*SampleName	Vegetable	Spinach puree	Control	Oat puree	1.9530	14.2428	146	0.14	0.8911
962	3	Group*SampleName	Vegetable	Spinach puree	Control	Spinach chopped	-1.3595	14.2428	146	-0.10	0.9241
963	3	Group*SampleName	Vegetable	Spinach puree	Control	Spinach puree	8.6405	14.2428	146	0.61	0.5450
964	3	Group*SampleName	Control	Asparagus chopped	Control	Asparagus puree	2.1250	11.5727	352	0.18	0.8544
965	3	Group*SampleName	Control	Asparagus chopped	Control	Beef puree	13.2500	11.5727	352	1.14	0.2530
966	3	Group*SampleName	Control	Asparagus chopped	Control	Black bean puree	4.3125	11.5727	352	0.37	0.7096
967	3	Group*SampleName	Control	Asparagus chopped	Control	Broccoli chopped	-5.8750	11.5727	352	-0.51	0.6120
968	3	Group*SampleName	Control	Asparagus chopped	Control	Broccoli puree	1.3125	11.5727	352	0.11	0.9098
969	3	Group*SampleName	Control	Asparagus chopped	Control	Chicken puree	14.1875	11.5727	352	1.23	0.2210
970	3	Group*SampleName	Control	Asparagus chopped	Control	Kale chopped	36.5625	11.5727	352	3.16	0.0017
971	3	Group*SampleName	Control	Asparagus	Control	Kale puree	37.8750	11.5727	352	3.27	0.0012

Obs	GameVisit	Effect	Group	SampleName	_Group	_SampleName	Estimate	StdErr	DF	tValue	Probt
				chopped							
972	3	Group*SampleName	Control	Asparagus chopped	Control	Oat puree	21.5000	11.5727	352	1.86	0.0640
973	3	Group*SampleName	Control	Asparagus chopped	Control	Spinach chopped	18.1875	11.5727	352	1.57	0.1169
974	3	Group*SampleName	Control	Asparagus chopped	Control	Spinach puree	28.1875	11.5727	352	2.44	0.0154
975	3	Group*SampleName	Control	Asparagus puree	Control	Beef puree	11.1250	11.5727	352	0.96	0.3371
976	3	Group*SampleName	Control	Asparagus puree	Control	Black bean puree	2.1875	11.5727	352	0.19	0.8502
977	3	Group*SampleName	Control	Asparagus puree	Control	Broccoli chopped	-8.0000	11.5727	352	-0.69	0.4898
978	3	Group*SampleName	Control	Asparagus puree	Control	Broccoli puree	-0.8125	11.5727	352	-0.07	0.9441
979	3	Group*SampleName	Control	Asparagus puree	Control	Chicken puree	12.0625	11.5727	352	1.04	0.2980
980	3	Group*SampleName	Control	Asparagus puree	Control	Kale chopped	34.4375	11.5727	352	2.98	0.0031
981	3	Group*SampleName	Control	Asparagus puree	Control	Kale puree	35.7500	11.5727	352	3.09	0.0022
982	3	Group*SampleName	Control	Asparagus puree	Control	Oat puree	19.3750	11.5727	352	1.67	0.0950
983	3	Group*SampleName	Control	Asparagus puree	Control	Spinach chopped	16.0625	11.5727	352	1.39	0.1660
984	3	Group*SampleName	Control	Asparagus puree	Control	Spinach puree	26.0625	11.5727	352	2.25	0.0249
985	3	Group*SampleName	Control	Beef puree	Control	Black bean puree	-8.9375	11.5727	352	-0.77	0.4405
986	3	Group*SampleName	Control	Beef puree	Control	Broccoli chopped	-19.1250	11.5727	352	-1.65	0.0993
987	3	Group*SampleName	Control	Beef puree	Control	Broccoli puree	-11.9375	11.5727	352	-1.03	0.3030
988	3	Group*SampleName	Control	Beef puree	Control	Chicken puree	0.9375	11.5727	352	0.08	0.9355
989	3	Group*SampleName	Control	Beef puree	Control	Kale chopped	23.3125	11.5727	352	2.01	0.0447
990	3	Group*SampleName	Control	Beef puree	Control	Kale puree	24.6250	11.5727	352	2.13	0.0340
991	3	Group*SampleName	Control	Beef puree	Control	Oat puree	8.2500	11.5727	352	0.71	0.4764
992	3	Group*SampleName	Control	Beef puree	Control	Spinach chopped	4.9375	11.5727	352	0.43	0.6699
993	3	Group*SampleName	Control	Beef puree	Control	Spinach puree	14.9375	11.5727	352	1.29	0.1976
994	3	Group*SampleName	Control	Black bean puree	Control	Broccoli chopped	-10.1875	11.5727	352	-0.88	0.3793
995	3	Group*SampleName	Control	Black bean puree	Control	Broccoli puree	-3.0000	11.5727	352	-0.26	0.7956
996	3	Group*SampleName	Control	Black bean puree	Control	Chicken puree	9.8750	11.5727	352	0.85	0.3941
997	3	Group*SampleName	Control	Black bean puree	Control	Kale chopped	32.2500	11.5727	352	2.79	0.0056
998	3	Group*SampleName	Control	Black bean puree	Control	Kale puree	33.5625	11.5727	352	2.90	0.0040
999	3	Group*SampleName	Control	Black bean puree	Control	Oat puree	17.1875	11.5727	352	1.49	0.1384
1000	3	Group*SampleName	Control	Black bean puree	Control	Spinach chopped	13.8750	11.5727	352	1.20	0.2314
1001	3	Group*SampleName	Control	Black bean puree	Control	Spinach puree	23.8750	11.5727	352	2.06	0.0398

Obs	GameVisit	Effect	Group	SampleName	_Group	_SampleName	Estimate	StdErr	DF	tValue	Probt
1002	3	Group*SampleName	Control	Broccoli chopped	Control	Broccoli puree	7.1875	11.5727	352	0.62	0.5350
1003	3	Group*SampleName	Control	Broccoli chopped	Control	Chicken puree	20.0625	11.5727	352	1.73	0.0839
1004	3	Group*SampleName	Control	Broccoli chopped	Control	Kale chopped	42.4375	11.5727	352	3.67	0.0003
1005	3	Group*SampleName	Control	Broccoli chopped	Control	Kale puree	43.7500	11.5727	352	3.78	0.0002
1006	3	Group*SampleName	Control	Broccoli chopped	Control	Oat puree	27.3750	11.5727	352	2.37	0.0185
1007	3	Group*SampleName	Control	Broccoli chopped	Control	Spinach chopped	24.0625	11.5727	352	2.08	0.0383
1008	3	Group*SampleName	Control	Broccoli chopped	Control	Spinach puree	34.0625	11.5727	352	2.94	0.0035
1009	3	Group*SampleName	Control	Broccoli puree	Control	Chicken puree	12.8750	11.5727	352	1.11	0.2667
1010	3	Group*SampleName	Control	Broccoli puree	Control	Kale chopped	35.2500	11.5727	352	3.05	0.0025
1011	3	Group*SampleName	Control	Broccoli puree	Control	Kale puree	36.5625	11.5727	352	3.16	0.0017
1012	3	Group*SampleName	Control	Broccoli puree	Control	Oat puree	20.1875	11.5727	352	1.74	0.0820
1013	3	Group*SampleName	Control	Broccoli puree	Control	Spinach chopped	16.8750	11.5727	352	1.46	0.1457
1014	3	Group*SampleName	Control	Broccoli puree	Control	Spinach puree	26.8750	11.5727	352	2.32	0.0208
1015	3	Group*SampleName	Control	Chicken puree	Control	Kale chopped	22.3750	11.5727	352	1.93	0.0540
1016	3	Group*SampleName	Control	Chicken puree	Control	Kale puree	23.6875	11.5727	352	2.05	0.0414
1017	3	Group*SampleName	Control	Chicken puree	Control	Oat puree	7.3125	11.5727	352	0.63	0.5279
1018	3	Group*SampleName	Control	Chicken puree	Control	Spinach chopped	4.0000	11.5727	352	0.35	0.7298
1019	3	Group*SampleName	Control	Chicken puree	Control	Spinach puree	14.0000	11.5727	352	1.21	0.2272
1020	3	Group*SampleName	Control	Kale chopped	Control	Kale puree	1.3125	11.5727	352	0.11	0.9098
1021	3	Group*SampleName	Control	Kale chopped	Control	Oat puree	-15.0625	11.5727	352	-1.30	0.1939
1022	3	Group*SampleName	Control	Kale chopped	Control	Spinach chopped	-18.3750	11.5727	352	-1.59	0.1132
1023	3	Group*SampleName	Control	Kale chopped	Control	Spinach puree	-8.3750	11.5727	352	-0.72	0.4697
1024	3	Group*SampleName	Control	Kale puree	Control	Oat puree	-16.3750	11.5727	352	-1.41	0.1580
1025	3	Group*SampleName	Control	Kale puree	Control	Spinach chopped	-19.6875	11.5727	352	-1.70	0.0898
1026	3	Group*SampleName	Control	Kale puree	Control	Spinach puree	-9.6875	11.5727	352	-0.84	0.4031
1027	3	Group*SampleName	Control	Oat puree	Control	Spinach chopped	-3.3125	11.5727	352	-0.29	0.7749
1028	3	Group*SampleName	Control	Oat puree	Control	Spinach puree	6.6875	11.5727	352	0.58	0.5637
1029	3	Group*SampleName	Control	Spinach chopped	Control	Spinach puree	10.0000	11.5727	352	0.86	0.3881

Next, we run simple paired t-tests on the data, comparing baseline to visit 2 and baseline to visit 3.

This is just as a double check, since these tests are typically less robust than mixed models.

In [12]:
```
# importing additional package for simple paired t-test
from scipy.stats import ttest_rel
```

In [13]:
```
#the following runs paired t-tests for all samples comparing visit 1 to 2 or 3, by group
pd.set_option('display.max_rows', None)

veg = gamevisits['SampleName'].unique()
qual = ['Liking','Sweetness','Bitterness']
group = ['Vegetable','Control']
output = pd.DataFrame(columns=['Vegetable', 'Quality', 'Group', 'B to 2, t',
                               'B to 2, p','B to 3, t', 'B to 3, p'])
for k in veg:
    for i in qual:
        for j in group:
            grouped = gamevisits[gamevisits['Group'] == j]
            sample = grouped[grouped['SampleName'] == k]

            visit1 = sample[sample['GameVisit'] == 1][i]
            visit2 = sample[sample['GameVisit'] == 2][i]
            visit3 = sample[sample['GameVisit'] == 3][i]

            tstat12, pval12 = ttest_rel(visit1, visit2)
            tstat13, pval13 = ttest_rel(visit1, visit3)

            #print(j, 'group', i, 'ratings for', k)
            #print("Comparing visit 1 to visit 2:")
            #print('t, 1-2: {:.3f}'.format(tstat12))
            #print('p, 1-2: {:.5f}'.format(pval12))
            #print("\nComparing visit 1 to visit 3:")
            #print('t, 1-3: {:.3f}'.format(tstat13))
            #print('p, 1-3: {:.5f}'.format(pval13), '\n')
            newrow = {"Vegetable": k, "Quality": i, 'Group': j, 'B to 2, t': tstat12,
                      'B to 2, p': pval12,'B to 3, t':tstat13, 'B to 3, p':pval13}
            output = output.append(newrow,ignore_index=True)

# You want to compare visit 1 to visit 2 and visit 1 to visit 3 for each veg
output
```

Out[13]:

	Vegetable	Quality	Group	B to 2, t	B to 2, p	B to 3, t	B to 3, p
**0**	Broccoli puree	Liking	Vegetable	0.709259	0.487778	-0.042506	0.966590
**1**	Broccoli puree	Liking	Control	0.914019	0.375165	-0.258954	0.799191
**2**	Broccoli puree	Sweetness	Vegetable	0.053747	0.957764	0.122818	0.903691
**3**	Broccoli puree	Sweetness	Control	-0.083726	0.934382	-0.863458	0.401482
**4**	Broccoli puree	Bitterness	Vegetable	0.598180	0.557606	0.176835	0.861728
**5**	Broccoli puree	Bitterness	Control	0.266078	0.793802	0.971099	0.346899
**6**	Kale puree	Liking	Vegetable	-0.427440	0.674424	-1.247173	0.229243
**7**	Kale puree	Liking	Control	2.502007	0.024409	2.533376	0.022941
**8**	Kale puree	Sweetness	Vegetable	-0.615450	0.546409	-0.624109	0.540842
**9**	Kale puree	Sweetness	Control	0.236011	0.816616	0.563876	0.581172
**10**	Kale puree	Bitterness	Vegetable	0.856699	0.403527	-0.168914	0.867858
**11**	Kale puree	Bitterness	Control	-0.902083	0.381270	-0.991309	0.337257
**12**	Asparagus puree	Liking	Vegetable	-0.246964	0.807894	-0.824149	0.421268
**13**	Asparagus puree	Liking	Control	-0.784556	0.444928	0.122420	0.904191
**14**	Asparagus puree	Sweetness	Vegetable	0.778377	0.447046	-0.469399	0.644748
**15**	Asparagus puree	Sweetness	Control	-1.493480	0.156047	1.123059	0.279067
**16**	Asparagus puree	Bitterness	Vegetable	-0.357734	0.724945	0.100422	0.921184

	Vegetable	Quality	Group	B to 2, t	B to 2, p	B to 3, t	B to 3, p
**17**	Asparagus puree	Bitterness	Control	-1.737864	0.102715	-1.800410	0.091935
**18**	Spinach puree	Liking	Vegetable	0.483534	0.634886	-0.108527	0.914849
**19**	Spinach puree	Liking	Control	0.706677	0.490600	1.826415	0.087756
**20**	Spinach puree	Sweetness	Vegetable	-0.695022	0.496432	-1.761561	0.096116
**21**	Spinach puree	Sweetness	Control	0.416828	0.682708	0.182271	0.857811
**22**	Spinach puree	Bitterness	Vegetable	-0.103745	0.918586	0.127448	0.900081
**23**	Spinach puree	Bitterness	Control	-2.427637	0.028254	-0.249201	0.806585
**24**	Chicken puree	Liking	Vegetable	-1.083689	0.293634	-1.350282	0.194629
**25**	Chicken puree	Liking	Control	-0.721164	0.481898	-0.850928	0.408188
**26**	Chicken puree	Sweetness	Vegetable	0.605584	0.552791	0.353521	0.728044
**27**	Chicken puree	Sweetness	Control	-1.964536	0.068272	-1.413300	0.177983
**28**	Chicken puree	Bitterness	Vegetable	-0.648057	0.525605	-1.251253	0.227788
**29**	Chicken puree	Bitterness	Control	-1.398084	0.182420	0.062844	0.950721
**30**	Beef puree	Liking	Vegetable	0.673667	0.509579	-1.339864	0.197926
**31**	Beef puree	Liking	Control	-0.450969	0.658463	-0.883178	0.391075
**32**	Beef puree	Sweetness	Vegetable	-0.533246	0.600766	-1.627186	0.122089
**33**	Beef puree	Sweetness	Control	-1.554057	0.141013	-1.705742	0.108670
**34**	Beef puree	Bitterness	Vegetable	-0.257590	0.799815	-0.662886	0.516291
**35**	Beef puree	Bitterness	Control	-1.424867	0.174670	1.315580	0.208069
**36**	Black bean puree	Liking	Vegetable	-0.441392	0.664492	-0.763309	0.455742
**37**	Black bean puree	Liking	Control	-0.996570	0.334779	-0.792487	0.440431
**38**	Black bean puree	Sweetness	Vegetable	-0.447530	0.660143	-0.536412	0.598624
**39**	Black bean puree	Sweetness	Control	0.738703	0.471489	-1.364727	0.192462
**40**	Black bean puree	Bitterness	Vegetable	-2.020564	0.059367	0.101339	0.920467
**41**	Black bean puree	Bitterness	Control	-2.355979	0.032498	-0.177795	0.861263
**42**	Oat puree	Liking	Vegetable	-0.267862	0.792028	-2.282962	0.035575
**43**	Oat puree	Liking	Control	-0.742872	0.469034	-1.032215	0.318328
**44**	Oat puree	Sweetness	Vegetable	-0.246475	0.808266	-1.188258	0.251068
**45**	Oat puree	Sweetness	Control	1.014385	0.326483	0.246532	0.808613
**46**	Oat puree	Bitterness	Vegetable	0.325485	0.748786	0.376198	0.711423
**47**	Oat puree	Bitterness	Control	-1.012772	0.327228	-0.966948	0.348903
**48**	Broccoli chopped	Liking	Vegetable	0.090779	0.928729	0.256737	0.800463
**49**	Broccoli chopped	Liking	Control	-1.965819	0.068111	-0.272357	0.789061
**50**	Broccoli chopped	Sweetness	Vegetable	0.400243	0.693962	1.147817	0.266941
**51**	Broccoli chopped	Sweetness	Control	-0.521896	0.609364	-0.557847	0.585179
**52**	Broccoli chopped	Bitterness	Vegetable	-1.872189	0.078487	-0.555650	0.585689
**53**	Broccoli chopped	Bitterness	Control	-0.741145	0.470050	0.415700	0.683516
**54**	Kale chopped	Liking	Vegetable	-2.494131	0.023225	-2.182879	0.043359
**55**	Kale chopped	Liking	Control	1.176805	0.257611	1.181696	0.255722
**56**	Kale chopped	Sweetness	Vegetable	-1.894928	0.075239	-1.536591	0.142796
**57**	Kale chopped	Sweetness	Control	1.224352	0.239702	0.664615	0.516385
**58**	Kale chopped	Bitterness	Vegetable	0.154243	0.879235	-0.054209	0.957400
**59**	Kale chopped	Bitterness	Control	-1.168619	0.260795	-0.906854	0.378822

	Vegetable	Quality	Group	B to 2, t	B to 2, p	B to 3, t	B to 3, p
**60**	Asparagus chopped	Liking	Vegetable	-1.974615	0.064784	-0.507170	0.618551
**61**	Asparagus chopped	Liking	Control	0.614759	0.547927	0.575050	0.573782
**62**	Asparagus chopped	Sweetness	Vegetable	0.888218	0.386819	0.507551	0.618290
**63**	Asparagus chopped	Sweetness	Control	0.186697	0.854400	-0.238964	0.814368
**64**	Asparagus chopped	Bitterness	Vegetable	-1.298460	0.211468	0.117122	0.908136
**65**	Asparagus chopped	Bitterness	Control	-3.023929	0.008546	-3.397736	0.003976
**66**	Spinach chopped	Liking	Vegetable	0.061563	0.951629	-1.937821	0.069438
**67**	Spinach chopped	Liking	Control	0.463930	0.649359	-0.266207	0.793704
**68**	Spinach chopped	Sweetness	Vegetable	0.707743	0.488695	-1.981993	0.063885
**69**	Spinach chopped	Sweetness	Control	0.186555	0.854509	-0.867870	0.399138
**70**	Spinach chopped	Bitterness	Vegetable	-0.585497	0.565905	2.416622	0.027196
**71**	Spinach chopped	Bitterness	Control	-1.848296	0.084371	1.778207	0.095641

```
In [ ]:
```
