# Supplementary material for: A method to “gamify” exposure to vegetable flavor and its potential influence on liking
Source: Food Sci Nutr. 2024 Jun 21;12(9):6873–85. doi: 10.1002/fsn3.4272 (PMC11561783; doi:10.1002/fsn3.4272)
Supplement: Supplementary file 2 — Data S2. [file FSN3-12-6873-s002.docx]

Supplemental file: Table of contents

[Pureed and chopped sample preparation 2](#_Toc167182916)

[Game Qualification 4](#_Toc167182917)

[Smell and Taste Acuity Tests 4](#_Toc167182918)

[Sensory evaluation 4](#_Toc167182919)

[Sample preparation 5](#_Toc167182920)

[Food Attitudes and Behaviors Survey 5](#_Toc167182921)

[Gummy Game Play 6](#_Toc167182922)

[Gummy formulations 12](#_Toc167182923)

[Subject Characteristics 15](#_Toc167182924)

[Code 16](#_Toc167182925)

[Additional results details 20](#_Toc167182926)

[Full statistics from Table 2 in main manuscript 20](#_Toc167182927)

[Liking Ratings: Type 3 effects 21](#_Toc167182928)

[Liking Ratings: Contrasts 22](#_Toc167182929)

[Liking Least Squared Means 26](#_Toc167182930)

[Sweetness Type 3 effects 32](#_Toc167182931)

[Sweetness: Contrasts 33](#_Toc167182932)

[Sweetness Least Squared Means 37](#_Toc167182933)

[Bitterness Type 3 effects 43](#_Toc167182934)

[Bitterness Contrasts 44](#_Toc167182935)

[Bitterness Least Square means 48](#_Toc167182936)

[Secondary analysis outputs 52](#_Toc167182937)

[Type 3 fixed effects 52](#_Toc167182938)

[Least Squared Means 52](#_Toc167182939)

[Differences between Least Squared Means 56](#_Toc167182940)

[Paired t-tests 78](#_Toc167182941)

# Pureed and chopped sample preparation

Pureed vegetables were cooked using a 1200W microwave from frozen, slightly longer than package directions to ensure uniform sample texture (see Table S1). Each vegetable type was pureed with enough de-ionized water to produce a thick puree (see Table S1). Purees were cooled to room temperature, packaged into opaque barrier pouches (ULINE, Pleasant Prairie, WI) to minimize visual cues, and frozen at -20C until use. Chopped vegetables were cooked using a 1200W microwave from frozen according to package directions (see Table S1), cooled to room temperature, packaged in 30 mL clear plastic souffle cups (Dart Container Corporation, Mason, MI), and frozen at -20°C until further use. Test samples were thawed overnight in a 4°C refrigerator before packaging to give to participants.

| **Table S1.** Sensory evaluation samples | | | | | | |
| --- | --- | --- | --- | --- | --- | --- |
| Sample Type | Sample Identifier | Sample Food | % w/w ingredient | % w/w DI water | Ingredient source | Cooking time (minutes @1200W) |
| Pureed | Target | Kale | 50 | 50 | Frozen chopped kale;  Great Value™, Walmart | 8 |
|  | Non-target | Spinach | 50 | 50 | Frozen chopped spinach;  Great Value™, Walmart | 8 |
|  | Target | Broccoli | 60 | 40 | Frozen broccoli florets;  Great Value™, Walmart | 6 |
|  | Non-target | Asparagus | 80 | 20 | Frozen asparagus spears;  Great Value™, Walmart | 5 |
|  | Distractor | Oat | 40 | 60 | Oat flour; Bob’s Red Mill | - |
|  | Distractor | Black bean | 90 | 10 | Fat free refried black beans;  Good & Gather, Target | - |
|  | Distractor | Beef | 100 | 0 | Beef & Gravy baby food;  Gerber Products Company | - |
|  | Distractor | Chicken | 100 | 0 | Chicken & Gravy baby food;  Gerber Products Company | - |
| Sample Type | Sample Identifier | Sample Food | % w/w ingredient | % w/w DI water | Ingredient source | Cooking time (minutes @1200W) |
| Chopped | Target | Kale | - | - | Frozen chopped kale;  Great Value™, Walmart | 6.5 |
|  | Non-target | Spinach | - | - | Frozen chopped spinach;  Great Value™, Walmart | 6.5 |
|  | Target | Broccoli | - | - | Frozen broccoli florets;  Great Value™, Walmart | 5 |
|  | Non-target | Asparagus | - | - | Frozen asparagus spears;  Great Value™, Walmart | 4 |

Participants rated bitterness, sweetness, and liking/disliking of each sample, followed by a 4-option multiple-choice question that asked participants to identify the sample tasted. Bitterness and sweetness intensity were rated on 110-pt visual analog scales, with labels corresponding to the following points: ‘None’ = 0, ‘Barely detectable’ = 5, ‘Weak’ = 25, ‘Moderate’ = 45, ‘Strong’ = 65, ‘Very strong’ = 85, and ‘Strongest ever’ = 105 (adapted from (Kershaw & Running, 2019)). Liking/disliking was rated on a hedonic visual analog scale ranging from -110 to 110, with labels corresponding to the following points: ‘Worst ever’ = -100, ‘Dislike’ = -50, ‘Neutral’ = 0, ‘Like’ = 50, ‘Best ever’ = 100. Participants evaluated all 8 pureed samples first and then evaluated all 4 chopped samples. Sample re-tasting during rating was allowed. Sample presentation was randomized within puree and chopped sample groups, respectively.

# Game Qualification

Once participants consented to be a part of the study, they scheduled a time to complete the study visit that determined whether they qualified for the Gummy Flavor Game. Participants completed all study procedures remotely using a remote testing kit. Samples were packed with ice packs. Participants were instructed to take the kit home and refrigerate it until the testing began. Participants were also instructed to refrain from eating or drinking anything besides water for an hour before testing, and to complete the test by the end of the day on which they picked up their kit to maintain sample freshness. A question at the beginning of the testing survey confirmed that the kit either still had a frozen ice pack or had been refrigerated until starting the test.

RedJade® (Redwood City, CA) sensory software was used to administer study tasks and to collect all sensory evaluation data.

First, participants answered questions about year of birth, gender, race/ethnicity, height, weight, and questions about health and smoking habits. 77 participants completed the baseline visit (51 women, 25 men, 1 nonbinary person; average age 23, age range 19-25).

## Smell and Taste Acuity Tests

Next, participants completed smell and taste acuity tests. Smell acuity was assessed using the Sensonics International Pocket Smell Test™ (Haddon Heights, NJ), a commercially available, 3-odor, forced-choice screening test that very briefly determines if an individual displays any olfactory dysfunction. It works similarly to a scratch-and-sniff card: each odor patch is scratched and smelled, then the individual chooses 1 response from 4 options. Getting one or more odor choices incorrect implies a degree of olfactory dysfunction.

Taste acuity was assessed using sweet, salty, sour, and bitter taste solutions. Solutions were prepared with de-ionized water and included: 5.0% w/w sucrose (sweet), 1.2% w/w sodium chloride (salty), 0.27% w/w citric acid (sour), and 0.016% w/w sucrose octaacetate (bitter). Participants were asked to taste each solution and match the solutions to their tastes (sweet, sour, salty, or bitter). We classified getting one or more taste choices incorrect as an indication of some level of taste dysfunction.

## Sensory evaluation

Participants evaluated eight pureed food samples and four chopped vegetable samples: kale, spinach, broccoli, and asparagus. Two of these would serve as target vegetables (kale and broccoli, included in the vegetable group’s game) and two of these as non-target vegetables (spinach and asparagus, not included in the game) in the subsequent game phase of the study. The remaining four pureed samples (black beans, beef, chicken, oats) were included to serve as non-vegetable distractor samples, and to increase difficulty during the sample identification task. All vegetable samples were made from locally purchased frozen vegetables. Distractor samples were made from ingredients purchased from local and online retailers.

## Sample preparation

Please see section above on “Pureed and chopped sample preparation.” These samples were the same in the qualification visit and in the baseline, week 1, and week 2 visits.

## Food Attitudes and Behaviors Survey

At the end of the Game phase qualification visit, participants answered questions from the National Cancer Institute’s Food Attitudes and Behaviors survey (Erinosho et al., 2015). This survey contained questions about frequency and quantity of fruit and vegetable consumption, among other questions about food beliefs and behaviors. Specific questions included in this study can be found in the supplemental files. Questions related to consumption frequency and quantity of salad, other vegetables (not including lettuce salads, beans/legumes, or potatoes), and tomato sauce were converted into a single value for Cup Equivalents (servings) of non-starchy vegetables according to the survey’s analytic guidance document (*FAB Analytic Guidance Document*, n.d.) for use in participant qualification.

# Gummy Game Play

No play on day 1 (baseline visit)

Day 2:

Day 3:

Day 4:

Day 5:

Day 6:

Day 7:

No play on day 8 (week 1 visit)

Day 9:

Day 10:

Day 11:

Day 12:

Day 13:

Day 14:

# Gummy formulations

For gummies where fresh, canned, or frozen products were used to create the flavor, “juice” and “puree” was prepared from the products as described in Table S2. These juices and purees were added to the final gummy formulation as described in Section 2.4.2 in the main manuscript.

Lab-made juices were made using raw or frozen ingredients listed in Table S2. For flavors made using frozen ingredients (Broccoli, Kale, Carrot, Green Pea, and Green Bean), ingredients were microwaved on defrost (<350W) for the times listed in Table 2. Next, defrosted ingredients were combined with deionized water and blended using an Instant™ Pot Ace™ Nova Cooking Blender (Instant Brands, Downers Grove, IL) according to the times and speeds listed in Table S2. After blending, the pulp was strained from the ‘juice’ using a fine-mesh sieve. The remaining pulp was microwaved (1200W) according to the times listed in Table S2, deionized water was added (if required), and mixture was blended until as smooth as possible.

For flavors made using raw vegetables as the ingredient source (Orange Bell Pepper, Cucumber, Butternut Squash, Mushroom), ingredients were not microwaved. Instead, whole vegetables were de-stemmed and chopped roughly before blending according to times and speeds listed in Table S2. After blending, the pulp was separated from the juice, and the remaining pulp was blended until as smooth as possible (except for Butternut Squash, which was microwaved according to the time listed in Table S2 before blending).

For flavors made using raw herbs as the ingredient source (Basil, Cilantro, Thyme, Dill), herbs were again not microwaved, but de-stemmed and roughly chopped before blending according to times and speeds listed in Table S2. The pulp was then separated from the juice. For all herb flavors, the herb “juice” was added to the gummy syrup. For basil, remaining puree leftover from juice making was added as a flavor addition post-cooking, whereas no puree was added after cooking for cilantro, thyme, or dill.

For all other flavors (Beef, Oat, Black Bean, Bacon, Garlic) deionized water was instead used as the liquid during the gummy syrup stage as described in Section 2.4.2. Beef and black bean purees were used as-is from the manufacturer, and oat puree was made by mixing specified quantities of oat flour and deionized water and leaving to hydrate at room temperature for 15 minutes.

Bacon and Garlic flavors were concentrated liquid natural/artificial flavors. These were added directly to the gummy syrup after cooking, along with the acid and coloring.

All lab-made juices and purees were frozen and vacuum-sealed for use in future gummy preparation.

| **Table S2.** Ingredient sources and formulation details for gummy juices and purees | | | | | | | | |
| --- | --- | --- | --- | --- | --- | --- | --- | --- |
| **Gummy type** | **Flavor ingredient source** | **Microwave defrosting time (minutes @ <350W)** | **Juice details** | | | **Puree details** | | |
|  |  |  | **%w/w food** | **%w/w water** | **Blend time (seconds - blend type)** | **Strained pulp microwave time (minutes @1200W)** | **Puree - %w/w food** | **Puree - %w/w water** |
| Broccoli | Frozen broccoli florets; Great Value™, Walmart | 7.5 | 66.7 | 33.3 | 20-30 - speed 2 | 6.0 | 90.9 | 9.1 |
| Kale | Frozen chopped kale; Great Value™, Walmart | 8 | 66.7 | 33.3 | 30-45 - speed 2 | 6.0 | 83.3 | 16.7 |
| Carrot | Frozen sliced carrots; Great Value™, Walmart | 6 | 66.7 | 33.3 | 30-40 - speed 2 | 7.0 | 90.9 | 9.1 |
| Green pea | Frozen sweet peas; Great Value™, Walmart | 6 | 62.5 | 37.5 | 10 - speed 2 | 6.0 | 100.0 | 0.0 |
| Green bean | Frozen cut green beans; Great Value™, Walmart | 8 | 66.7 | 33.3 | 15-20 - speed 2 | 6.0 | 83.3 | 16.7 |
| Orange bell pepper | Raw orange bell pepper; Walmart | - | 80.0 | 20.0 | 10-15 - speed 2 | - | 100.0 | 0.0 |
| Cucumber | Raw English cucumber; Walmart | - | 80.0 | 20.0 | 20-30 - speed 2 | - | 100.0 | 0.0 |
| Basil | Fresh basil leaves; Shenandoah Growers | - | 91.2 | 8.8 | 10 - speed 2 | - | 100.0 | 0.0 |
| Butternut squash | Raw butternut squash; Walmart | - | 66.7 | 33.3 | 30 - speed 3 | 6.0 | 100.0 | 0.0 |
| Beef | Beef and gravy baby food; Gerber Products Company | - | - | - | - | - | 100.0 | 0.0 |
| Oat | Oat flour; Bob's Red Mill | - | 25.0 | 75.0 | - | - | 100.0 | 0.0 |
| Cilantro | Fresh cilantro leaves; Walmart | - | 87.6 | 12.4 | 20 - speed 2 | - | - | - |
| Bacon | Bacon flavor, Sensient Flavors & Extracts | - | - | - | - | - | - | - |
| Mushroom | Whole white mushrooms; Meijer | - | 50.0 | 50.0 | 5-10 - pulse | - | 100.0 | 0.0 |
| Thyme | Fresh thyme leaves; Shenandoah Growers | - | 98.5 | 1.5 | 20 - speed 2 | - | 100.0 | 0.0 |
| Black bean | Canned fat-free refried black beans; Good and Gather, Target | - | - | - | - | - | 100.0 | 0.0 |
| Garlic | Garlic flavor, Sensient Flavors & Extracts | - | - | - | - | - | - | - |
| Dill | Fresh dill leaves, Shenandoah Growers | - | 93.2 | 6.8 | 5 - speed 2 | - | - | - |

| **Table S3.** Detailed gummy flavor formulations. Juices and purees were formulated per Table S2 in the supplemental files. | | | | | | | | | | | |
| --- | --- | --- | --- | --- | --- | --- | --- | --- | --- | --- | --- |
| **Group** | **Gummy Flavor** | **Syrup Liquid Type** | **Final Syrup Cook Temp (°C)** | **Post-cooking flavor addition** | **Syrup details (%w/w)** | | | | **Post-cooking additions (%w/w)** | | |
|  |  |  |  |  | **Gelatin** | **Sucrose** | **Light corn syrup** | **Syrup liquid** | **50% citric acid** | **Flavor addition** | **Color addition** |
| Vegetable | Broccoli | Juice | 105.0 | Puree | 6.0 | 31.7 | 32.9 | 20.6 | 0.8 | 7.9 | <0.1 |
|  | Kale | Juice | 105.0 | Puree | 6.0 | 31.7 | 32.9 | 20.6 | 0.8 | 7.9 | <0.1 |
|  | Carrot | Juice | 105.0 | Puree | 6.0 | 31.7 | 32.9 | 20.6 | 0.8 | 7.9 | <0.1 |
|  | Green pea | Juice | 105.0 | Puree | 6.0 | 31.7 | 32.9 | 20.6 | 0.8 | 7.9 | <0.1 |
|  | Green bean | Juice | 105.0 | Puree | 6.0 | 31.7 | 32.9 | 20.6 | 0.8 | 7.9 | <0.1 |
|  | Orange bell pepper | Juice | 105.0 | Puree | 6.0 | 31.7 | 32.9 | 20.6 | 0.8 | 7.9 | <0.1 |
|  | Cucumber | Juice | 105.0 | Puree | 6.0 | 31.7 | 32.9 | 20.6 | 0.8 | 7.9 | <0.1 |
|  | Basil | Juice | 104.5 | Puree | 6.3 | 33.5 | 34.8 | 21.8 | 0.8 | 2.8 | <0.1 |
|  | Butternut squash | Juice | 105.0 | Puree | 6.0 | 31.7 | 32.9 | 20.6 | 0.8 | 7.9 | <0.1 |
| Control | Beef | Water | 105.0 | Puree | 6.0 | 31.7 | 32.9 | 20.6 | 0.8 | 7.9 | <0.1 |
|  | Oat | Water | 105.0 | Puree | 6.0 | 31.7 | 32.9 | 20.6 | 0.8 | 7.9 | <0.1 |
|  | Cilantro | Juice | 103.7 | None | 6.5 | 34.5 | 35.8 | 22.4 | 0.9 | NA | <0.1 |
|  | Bacon | Water | 103.7 | Flavor | 6.4 | 34.4 | 35.7 | 22.4 | 0.9 | 0.3 | <0.1 |
|  | Mushroom | Water | 105.0 | Puree | 6.1 | 32.6 | 33.8 | 21.2 | 0.8 | 5.5 | <0.1 |
|  | Thyme | Juice | 103.7 | None | 6.5 | 34.5 | 35.8 | 22.4 | 0.9 | NA | <0.1 |
|  | Black bean | Water | 105.0 | Puree | 6.0 | 31.7 | 32.9 | 20.6 | 0.8 | 7.9 | <0.1 |
|  | Garlic | Water | 103.7 | Flavor | 6.5 | 34.4 | 35.7 | 22.4 | 0.9 | 0.1 | <0.1 |
|  | Dill | Juice | 105.0 | None | 6.5 | 34.5 | 35.8 | 22.4 | 0.9 | NA | <0.1 |
| *Target vegetables--flavors exposed daily in gameplay for the Vegetable Intervention Group | | | | | | | | | | | |

# Subject Characteristics

| **Table S4.** Demographic characteristics of study participants (N=34) | | |
| --- | --- | --- |
| ***Demographic*** | **Control (N=16)** | **Vegetable (N=18)** |
| **Age** |  |  |
| *Mean ± SD* | 22 ± 1.9 | 22 ± 2.1 |
| *Range* | 19 - 25 | 19 - 25 |
| **Gender** |  |  |
| *N* | 10 Women, 6 Men | 14 Women, 4 Men |
| **BMI** |  |  |
| *Mean ± SD* | 25.1 ± 4.2 | 25.8 ± 5.4 |
| *Range* | 18.3 – 33.4 | 19.6 – 35.9 |
| **Race/Ethnicity (Self-described)** |  |  |
| *N* | 9 White, 5 Asian/Southeast Asian/Chinese/Hakkanese, 2 Hispanic/Latino | 10 White, 6 Asian/Southeast Asian/Indian/Brown/Chinese, 1 Latin, 1 Mixed (Hispanic/Black/White) |

39 participants originally qualified for the game phase. Two participants elected not to start the game phase for unrelated personal reasons, and three participants enrolled in the Control intervention arm dropped out during the game phase either due to unrelated illness (1) or non-compliance with the protocol (2).

# Code

This python code analyses data from our Veggie Flavor Game intervention.

We ran the code in Jupyter Notebook.

*#These packages are used in the analysis*

**import** pandas **as** pd

**import** numpy **as** np

**import** saspy

sas **=** saspy**.**SASsession()

*#This is our raw data in long form (each *rating* is it's own row, so participants have many rows each)*

gamevisits **=** pd**.**read_csv('./GameVisitsLong.csv')

*#gamevisits.info()*

*# reading the data into the SAS package*

vegsas **=** sas**.**df2sd(gamevisits, 'gamevisits')

**%%SAS** sas

proc sort data=gamevisits;

by samplename;

run;

title 'OSF planned analysis: Liking';

ods output diffs=diff1 tests3=tests1

SolutionF=sol1 lsmeans=means1 FitStatistics=fit1 lsmESTIMATEs =LikeEstim1;

proc mixed data= gamevisits;

*where participantid ne 1010 ;

*excluding this person does not change kale chopped outcome

(we thought they might have been an outlier, with almost 100 pts increase, but

turns out they are not per the rule of more than 1.5 IQR above Q3);

class participantID gamevisit group (ref = 'Control') gender samplename;

by samplename;

model liking = gamevisit group gamevisit*group gender/ ddfm=kr solution; *fit is slightly better with gender;

repeated/ subject = participantID type = cs;

lsmeans group/pdiff ADJDFE=ROW;

lsmeans gamevisit/pdiff ADJDFE=ROW;

lsmeans gamevisit*group/pdiff adjdfe=row;

lsmestimate gamevisit*group 'Control, WEEK2-BASELINE' 0 -1 0 0 0 1 ;

lsmestimate gamevisit*group 'Control, WEEK1-BASELINE' 0 -1 0 1 0 0 ;

lsmestimate gamevisit*group 'Veg, WEEK2-BASELINE' -1 0 0 0 1 0 ;

lsmestimate gamevisit*group 'Veg, WEEK1-BASELINE' -1 0 1 0 0 0 ;

lsmestimate gamevisit*group 'Veg - Control, BASELINE' 1 -1 0 0 0 0 ;

lsmestimate gamevisit*group 'Veg - Control, WEEK1' 0 0 1 -1 0 0 ;

lsmestimate gamevisit*group 'Veg - Control, WEEK2' 0 0 0 0 1 -1 ;

run;

proc sort data=gamevisits;

by samplename;

run;

title 'OSF planned analysis: Sweetness';

ods output diffs=diff2 tests3=tests2

SolutionF=sol2 lsmeans=means2 FitStatistics=fit2 lsmESTIMATEs =LikeEstim2;

proc mixed data= gamevisits;

class participantID gamevisit group (ref = 'Control') gender samplename;

by samplename;

model Sweetness = gamevisit group gamevisit*group gender/ ddfm=kr solution; *fit is slightly better with gender;

repeated/ subject = participantID type = cs;

lsmeans group/pdiff ADJDFE=ROW;

lsmeans gamevisit/pdiff ADJDFE=ROW;

lsmeans gamevisit*group/pdiff adjdfe=row;

lsmestimate gamevisit*group 'Control, WEEK2-BASELINE' 0 -1 0 0 0 1 ;

lsmestimate gamevisit*group 'Control, WEEK1-BASELINE' 0 -1 0 1 0 0 ;

lsmestimate gamevisit*group 'Veg, WEEK2-BASELINE' -1 0 0 0 1 0 ;

lsmestimate gamevisit*group 'Veg, WEEK1-BASELINE' -1 0 1 0 0 0 ;

lsmestimate gamevisit*group 'Veg - Control, BASELINE' 1 -1 0 0 0 0 ;

lsmestimate gamevisit*group 'Veg - Control, WEEK1' 0 0 1 -1 0 0 ;

lsmestimate gamevisit*group 'Veg - Control, WEEK2' 0 0 0 0 1 -1 ;

run;

proc sort data=gamevisits;

by samplename;

run;

title 'OSF planned analysis: Bitterness';

ods output diffs=diff3 tests3=tests3

SolutionF=sol3 lsmeans=means3 FitStatistics=fit3 lsmESTIMATEs =LikeEstim3;

proc mixed data= gamevisits;

class participantID gamevisit group (ref = 'Control') gender samplename;

by samplename;

model Bitterness = gamevisit group gamevisit*group gender/ ddfm=kr solution; *fit is slightly better with gender;

repeated/ subject = participantID type = cs;

lsmeans group/pdiff ADJDFE=ROW;

lsmeans gamevisit/pdiff ADJDFE=ROW;

lsmeans gamevisit*group/pdiff adjdfe=row;

lsmestimate gamevisit*group 'Control, WEEK2-BASELINE' 0 -1 0 0 0 1 ;

lsmestimate gamevisit*group 'Control, WEEK1-BASELINE' 0 -1 0 1 0 0 ;

lsmestimate gamevisit*group 'Veg, WEEK2-BASELINE' -1 0 0 0 1 0 ;

lsmestimate gamevisit*group 'Veg, WEEK1-BASELINE' -1 0 1 0 0 0 ;

lsmestimate gamevisit*group 'Veg - Control, BASELINE' 1 -1 0 0 0 0 ;

lsmestimate gamevisit*group 'Veg - Control, WEEK1' 0 0 1 -1 0 0 ;

lsmestimate gamevisit*group 'Veg - Control, WEEK2' 0 0 0 0 1 -1 ;

run;

proc sort data=gamevisits;

by gamevisit;

run;

title 'Secondard analysis: Liking between vegetables';

ods output diffs=diff4 tests3=tests4

SolutionF=sol4 lsmeans=means4 FitStatistics=fit4 lsmESTIMATEs =LikeEstim4;

proc mixed data= gamevisits;

class participantID gamevisit group (ref = 'Control') gender samplename;

by gamevisit;

model liking = samplename group samplename*group gender/ ddfm=kr solution; *fit is slightly better with gender;

repeated/ subject = participantID type = cs;

lsmeans group/pdiff ADJDFE=ROW;

lsmeans samplename/pdiff ADJDFE=ROW;

lsmeans samplename*group/pdiff adjdfe=row;

run;

title 'OSF planned analysis: Liking';

proc print data = tests1;

proc print data =likeestim1;

proc print data=means1;

run;

title 'OSF planned analysis: Sweetness';

proc print data = tests2;

proc print data =likeestim2;

proc print data=means2;

run;

title 'OSF planned analysis: Bitterness';

proc print data = tests3;

proc print data =likeestim3;

proc print data=means3;

run;

title 'Secondary analysis: Differences between vegetables, and from 0';

proc print data = tests4;

proc print data=means4;

proc print data = diff4;

run;

*# importing additional package for simple paired t-test*

**from** scipy.stats **import** ttest_rel

*#the following runs paired t-tests for all samples comparing baseline to 2 or 3, by group*

pd**.**set_option('display.max_rows', **None**)

veg **=** gamevisits['SampleName']**.**unique()

qual **=** ['Liking','Sweetness','Bitterness']

group **=** ['Vegetable','Control']

output **=** pd**.**DataFrame(columns**=**['Vegetable', 'Quality', 'Group', 'B to 2, t',

'B to 2, p','B to 3, t', 'B to 3, p'])

**for** k **in** veg:

**for** i **in** qual:

**for** j **in** group:

grouped **=** gamevisits[gamevisits['Group'] **==** j]

sample **=** grouped[grouped['SampleName'] **==** k]

visit1 **=** sample[sample['GameVisit'] **==** 1][i]

visit2 **=** sample[sample['GameVisit'] **==** 2][i]

visit3 **=** sample[sample['GameVisit'] **==** 3][i]

tstat12, pval12 **=** ttest_rel(visit1, visit2)

tstat13, pval13 **=** ttest_rel(visit1, visit3)

*#print(j, 'group', i, 'ratings for', k)*

*#print("Comparing baseline to week 1:")*

*#print('t, 1-2: {:.3f}'.format(tstat12))*

*#print('p, 1-2: {:.5f}'.format(pval12))*

*#print("\nComparing baseline to week 2:")*

*#print('t, 1-3: {:.3f}'.format(tstat13))*

*#print('p, 1-3: {:.5f}'.format(pval13), '\n')*

newrow **=** {"Vegetable": k, "Quality": i, 'Group': j, 'B to 2, t': tstat12,

'B to 2, p': pval12,'B to 3, t':tstat13, 'B to 3, p':pval13}

output **=** output**.**append(newrow,ignore_index**=True**)

*# You want to compare baseline to week 1 and baseline to week 2 for each veg*

output

# Additional results details

## Full statistics from Table 2 in main manuscript

| **Table 2, expanded detail: Least Squared Mean liking ratings between and within groups** | | | | | | | | |
| --- | --- | --- | --- | --- | --- | --- | --- | --- |
| **Sample** | **Sensory Visit** | **Group** | **Mean†** | **SE** | **Between Groups, within Sensory Visit** | **p-value‡** | **Within Group,**  **Compared to Baseline** | **p-value‡** |
| Asparagus chopped | Baseline | Vegetable | -6 | 10 | Baseline | 0.092 | Vegetable, Week 1: | **0.035** |
|  |  | Control | 17 | 10 | Week 1 | 0.87 | Vegetable, Week 2: | 0.66 |
|  | Week 1 | Vegetable | 9 | 10 | Week 2 | 0.26 | Control, Week 1: | 0.46 |
|  |  | Control | 11 | 10 |  |  | Control, Week 2: | 0.54 |
|  | Week 2 | Vegetable | -3 | 10 |  |  |  |  |
|  |  | Control | 12 | 10 |  |  |  |  |
| Asparagus puree | Baseline | Vegetable | -5 | 10 | Baseline | 0.22 | Vegetable, Week 1: | 0.77 |
|  |  | Control | 11 | 10 | Week 1 | 0.15 | Vegetable, Week 2: | 0.34 |
|  | Week 1 | Vegetable | -3 | 10 | Week 2 | 0.53 | Control, Week 1: | 0.53 |
|  |  | Control | 16 | 10 |  |  | Control, Week 2: | 0.91 |
|  | Week 2 | Vegetable | 2 | 10 |  |  |  |  |
|  |  | Control | 10 | 10 |  |  |  |  |
| Broccoli chopped | Baseline | Vegetable | 8 | 11 | Baseline | 0.74 | Vegetable, Week 1: | 0.94 |
|  |  | Control | 15 | 11 | Week 1 | 0.35 | Vegetable, Week 2: | 0.82 |
|  | Week 1 | Vegetable | 8 | 11 | Week 2 | 0.50 | Control, Week 1: | 0.32 |
|  |  | Control | 21**†** | 11 |  |  | Control, Week 2: | 0.69 |
|  | Week 2 | Vegetable | 6 | 11 |  |  |  |  |
|  |  | Control | 16 | 11 |  |  |  |  |
| Broccoli puree | Baseline | Vegetable | 16 | 9 | Baseline | 0.57 | Vegetable, Week 1: | 0.58 |
|  |  | Control | 9 | 9 | Week 1 | 0.45 | Vegetable, Week 2: | 0.96 |
|  | Week 1 | Vegetable | 12 | 9 | Week 2 | 0.67 | Control, Week 1: | 0.40 |
|  |  | Control | 2 | 9 |  |  | Control, Week 2: | 0.78 |
|  | Week 2 | Vegetable | 16 | 9 |  |  |  |  |
|  |  | Control | 11 | 9 |  |  |  |  |
| Kale chopped | Baseline | Vegetable | -28**†** | 9 | Baseline | 0.23 | Vegetable, Week 1: | **0.016** |
|  |  | Control | -13 | 9 | Week 1 | 0.46 | Vegetable, Week 2: | **0.0075** |
|  | Week 1 | Vegetable | -11 | 9 | Week 2 | 0.29 | Control, Week 1: | 0.35 |
|  |  | Control | -20**†** | 9 |  |  | Control, Week 2: | 0.22 |
|  | Week 2 | Vegetable | -8 | 9 |  |  |  |  |
|  |  | Control | -22**†** | 9 |  |  |  |  |
| Kale puree | Baseline | Vegetable | -17**†** | 8 | Baseline | 0.32 | Vegetable, Week 1: | 0.68 |
|  |  | Control | -6 | 8 | Week 1 | 0.61 | Vegetable, Week 2: | 0.13 |
|  | Week 1 | Vegetable | -15 | 8 | Week 2 | 0.13 | Control, Week 1: | **0.045** |
|  |  | Control | -21**†** | 8 |  |  | Control, Week 2: | **0.010** |
|  | Week 2 | Vegetable | -7 | 8 |  |  |  |  |
|  |  | Control | -25**†** | 8 |  |  |  |  |
| Spinach chopped | Baseline | Vegetable | -7 | 9 | Baseline | 0.97 | Vegetable, Week 1: | 0.94 |
|  |  | Control | -7 | 9 | Week 1 | 0.84 | Vegetable, Week 2: | *0.095* |
|  | Week 1 | Vegetable | -7 | 9 | Week 2 | 0.36 | Control, Week 1: | 0.76 |
|  |  | Control | -10 | 9 |  |  | Control, Week 2: | 0.84 |
|  | Week 2 | Vegetable | 5 | 9 |  |  |  |  |
|  |  | Control | -6 | 9 |  |  |  |  |
| Spinach puree | Baseline | Vegetable | -8 | 7 | Baseline | 0.95 | Vegetable, Week 1: | 0.51 |
|  |  | Control | -8 | 7 | Week 1 | 0.90 | Vegetable, Week 2: | 0.91 |
|  | Week 1 | Vegetable | -13 | 7 | Week 2 | 0.39 | Control, Week 1: | 0.58 |
|  |  | Control | -12 | 7 |  |  | Control, Week 2: | 0.27 |
|  | Week 2 | Vegetable | -8 | 7 |  |  |  |  |
|  |  | Control | -16**†** | 7 |  |  |  |  |
| Model (run by sample) Liking = Visit Group (Visit*Group) Gender (gender included as covariate only)  **†**Indicates least squared mean is significantly different (p<0.05) from 0, which is “Neutral” on our scale  **‡**Means and p-values generated from contrast statements for interaction term Visit*Group, setting the visits equal and comparing the two groups or by setting the group equal and comparing across visits.  Non-vegetable samples shown in supplemental files.  Ratings are from a -110 to 110 visual analog scale, with labels corresponding to:  ‘Worst ever’ = -100, ‘Dislike’ = -50, ‘Neutral’ = 0, ‘Like’ = 50, ‘Best ever’ = 100 | | | | | | | | |

Full output can be found in the HTML file which includes all outputs from our original code.

Key tables are pasted below, with headers/labels edited for readability. Note that in the HTML code output, the baseline visit is “1,” week 1 visit is “2,” and week 2 visit is “3.”

## Liking Ratings: Type 3 effects

P-values (ProbF) in this table indicate whether the effect is significant in the overall model.

| **SampleName** | **Effect** | **NumDF** | **DenDF** | **FValue** | **ProbF** |
| --- | --- | --- | --- | --- | --- |
| Asparagus chopped | GameVisit | 2 | 64 | 0.69 | 0.5037 |
| Asparagus chopped | Group | 1 | 31 | 1.27 | 0.2683 |
| Asparagus chopped | GameVisit*Group | 2 | 64 | 2.08 | 0.1333 |
| Asparagus chopped | Gender | 1 | 31 | 0.14 | 0.7127 |
| Asparagus puree | GameVisit | 2 | 64 | 0.26 | 0.7743 |
| Asparagus puree | Group | 1 | 31 | 1.59 | 0.2167 |
| Asparagus puree | GameVisit*Group | 2 | 64 | 0.55 | 0.5816 |
| Asparagus puree | Gender | 1 | 31 | 0.01 | 0.9141 |
| Beef puree | GameVisit | 2 | 64 | 2.07 | 0.1341 |
| Beef puree | Group | 1 | 31 | 0.58 | 0.4511 |
| Beef puree | GameVisit*Group | 2 | 64 | 0.46 | 0.6312 |
| Beef puree | Gender | 1 | 31 | 1.55 | 0.2218 |
| Black bean puree | GameVisit | 2 | 64 | 0.82 | 0.4468 |
| Black bean puree | Group | 1 | 31 | 1.01 | 0.3216 |
| Black bean puree | GameVisit*Group | 2 | 64 | 0.14 | 0.8733 |
| Black bean puree | Gender | 1 | 31 | 0.08 | 0.7822 |
| Broccoli chopped | GameVisit | 2 | 64 | 0.26 | 0.7711 |
| Broccoli chopped | Group | 1 | 31 | 0.54 | 0.4675 |
| Broccoli chopped | GameVisit*Group | 2 | 64 | 0.31 | 0.7355 |
| Broccoli chopped | Gender | 1 | 31 | 1.55 | 0.2225 |
| Broccoli puree | GameVisit | 2 | 64 | 0.87 | 0.4224 |
| Broccoli puree | Group | 1 | 31 | 0.44 | 0.5134 |
| Broccoli puree | GameVisit*Group | 2 | 64 | 0.08 | 0.919 |
| Broccoli puree | Gender | 1 | 31 | 0.2 | 0.6588 |
| Chicken puree | GameVisit | 2 | 64 | 1.39 | 0.2556 |
| Chicken puree | Group | 1 | 31 | 0.53 | 0.474 |
| Chicken puree | GameVisit*Group | 2 | 64 | 0.08 | 0.9268 |
| Chicken puree | Gender | 1 | 31 | 1.16 | 0.2901 |
| Kale chopped | GameVisit | 2 | 64 | 0.66 | 0.5181 |
| Kale chopped | Group | 1 | 31 | 0.05 | 0.8194 |
| Kale chopped | GameVisit*Group | 2 | 64 | 4.56 | 0.014 |
| Kale chopped | Gender | 1 | 31 | 1.23 | 0.2759 |
| Kale puree | GameVisit | 2 | 64 | 0.78 | 0.4645 |
| Kale puree | Group | 1 | 31 | 0.16 | 0.6895 |
| Kale puree | GameVisit*Group | 2 | 64 | 4.49 | 0.015 |
| Kale puree | Gender | 1 | 31 | 0.03 | 0.8565 |
| Oat puree | GameVisit | 2 | 64 | 2.93 | 0.0605 |
| Oat puree | Group | 1 | 31 | 2.93 | 0.0971 |
| Oat puree | GameVisit*Group | 2 | 64 | 0.34 | 0.7109 |
| Oat puree | Gender | 1 | 31 | 0.17 | 0.6843 |
| Spinach chopped | GameVisit | 2 | 64 | 1.43 | 0.2474 |
| Spinach chopped | Group | 1 | 31 | 0.2 | 0.6598 |
| Spinach chopped | GameVisit*Group | 2 | 64 | 0.59 | 0.5558 |
| Spinach chopped | Gender | 1 | 31 | 0.03 | 0.8634 |
| Spinach puree | GameVisit | 2 | 64 | 0.43 | 0.651 |
| Spinach puree | Group | 1 | 31 | 0.08 | 0.7821 |
| Spinach puree | GameVisit*Group | 2 | 64 | 0.56 | 0.5759 |
| Spinach puree | Gender | 1 | 31 | 0 | 0.9777 |

## Liking Ratings: Contrasts

P-values in this table indicate whether the estimate of the difference between the two means is significantly different from 0.

| **SampleName** | **Effect** | **Label** | **Estimate** | **StdErr** | **DF** | **tValue** | **Probt** |
| --- | --- | --- | --- | --- | --- | --- | --- |
| Asparagus chopped | GameVisit*Group | Control, Week 2-Baseline | -4.5625 | 7.4146 | 64 | -0.62 | 0.5405 |
| Asparagus chopped | GameVisit*Group | Control, Week 1-Baseline | -5.5 | 7.4146 | 64 | -0.74 | 0.4609 |
| Asparagus chopped | GameVisit*Group | Veg, Week 2-Baseline | 3.0556 | 6.9905 | 64 | 0.44 | 0.6635 |
| Asparagus chopped | GameVisit*Group | Veg, Week 1-Baseline | 15.0556 | 6.9905 | 64 | 2.15 | 0.035 |
| Asparagus chopped | GameVisit*Group | Veg - Control, Baseline | -22.6483 | 13.151 | 47.03 | -1.72 | 0.0916 |
| Asparagus chopped | GameVisit*Group | Veg - Control, Week 1 | -2.0927 | 13.151 | 47.03 | -0.16 | 0.8742 |
| Asparagus chopped | GameVisit*Group | Veg - Control, Week 2 | -15.0302 | 13.151 | 47.03 | -1.14 | 0.2589 |
| Asparagus puree | GameVisit*Group | Control, Week 2-Baseline | -0.9375 | 7.9788 | 64 | -0.12 | 0.9068 |
| Asparagus puree | GameVisit*Group | Control, Week 1-Baseline | 5.0625 | 7.9788 | 64 | 0.63 | 0.528 |
| Asparagus puree | GameVisit*Group | Veg, Week 2-Baseline | 7.2222 | 7.5225 | 64 | 0.96 | 0.3406 |
| Asparagus puree | GameVisit*Group | Veg, Week 1-Baseline | 2.1667 | 7.5225 | 64 | 0.29 | 0.7743 |
| Asparagus puree | GameVisit*Group | Veg - Control, Baseline | -16.6595 | 13.4086 | 49.37 | -1.24 | 0.2199 |
| Asparagus puree | GameVisit*Group | Veg - Control, Week 1 | -19.5553 | 13.4086 | 49.37 | -1.46 | 0.1511 |
| Asparagus puree | GameVisit*Group | Veg - Control, Week 2 | -8.4998 | 13.4086 | 49.37 | -0.63 | 0.5291 |
| Beef puree | GameVisit*Group | Control, Week 2-Baseline | 7.25 | 6.5551 | 64 | 1.11 | 0.2729 |
| Beef puree | GameVisit*Group | Control, Week 1-Baseline | 2.6875 | 6.5551 | 64 | 0.41 | 0.6832 |
| Beef puree | GameVisit*Group | Veg, Week 2-Baseline | 7.5556 | 6.1802 | 64 | 1.22 | 0.226 |
| Beef puree | GameVisit*Group | Veg, Week 1-Baseline | -4.6667 | 6.1802 | 64 | -0.76 | 0.453 |
| Beef puree | GameVisit*Group | Veg - Control, Baseline | -12.0331 | 19.5509 | 35.8 | -0.62 | 0.5421 |
| Beef puree | GameVisit*Group | Veg - Control, Week 1 | -19.3872 | 19.5509 | 35.8 | -0.99 | 0.328 |
| Beef puree | GameVisit*Group | Veg - Control, Week 2 | -11.7275 | 19.5509 | 35.8 | -0.6 | 0.5524 |
| Black bean puree | GameVisit*Group | Control, Week 2-Baseline | 8.6875 | 9.9642 | 64 | 0.87 | 0.3865 |
| Black bean puree | GameVisit*Group | Control, Week 1-Baseline | 10.9375 | 9.9642 | 64 | 1.1 | 0.2765 |
| Black bean puree | GameVisit*Group | Veg, Week 2-Baseline | 6.6667 | 9.3943 | 64 | 0.71 | 0.4805 |
| Black bean puree | GameVisit*Group | Veg, Week 1-Baseline | 4 | 9.3943 | 64 | 0.43 | 0.6717 |
| Black bean puree | GameVisit*Group | Veg - Control, Baseline | -10.6131 | 15.6445 | 52.9 | -0.68 | 0.5005 |
| Black bean puree | GameVisit*Group | Veg - Control, Week 1 | -17.5506 | 15.6445 | 52.9 | -1.12 | 0.267 |
| Black bean puree | GameVisit*Group | Veg - Control, Week 2 | -12.6339 | 15.6445 | 52.9 | -0.81 | 0.423 |
| Broccoli chopped | GameVisit*Group | Control, Week 2-Baseline | 3.3125 | 8.182 | 64 | 0.4 | 0.6869 |
| Broccoli chopped | GameVisit*Group | Control, Week 1-Baseline | 8.25 | 8.182 | 64 | 1.01 | 0.3171 |
| Broccoli chopped | GameVisit*Group | Veg, Week 2-Baseline | -1.7222 | 7.7141 | 64 | -0.22 | 0.824 |
| Broccoli chopped | GameVisit*Group | Veg, Week 1-Baseline | -0.5556 | 7.7141 | 64 | -0.07 | 0.9428 |
| Broccoli chopped | GameVisit*Group | Veg - Control, Baseline | -4.8845 | 14.4514 | 47.2 | -0.34 | 0.7369 |
| Broccoli chopped | GameVisit*Group | Veg - Control, Week 1 | -13.6901 | 14.4514 | 47.2 | -0.95 | 0.3483 |
| Broccoli chopped | GameVisit*Group | Veg - Control, Week 2 | -9.9193 | 14.4514 | 47.2 | -0.69 | 0.4958 |
| Broccoli puree | GameVisit*Group | Control, Week 2-Baseline | 2.0625 | 7.2518 | 64 | 0.28 | 0.777 |
| Broccoli puree | GameVisit*Group | Control, Week 1-Baseline | -6.1875 | 7.2518 | 64 | -0.85 | 0.3967 |
| Broccoli puree | GameVisit*Group | Veg, Week 2-Baseline | 0.3333 | 6.837 | 64 | 0.05 | 0.9613 |
| Broccoli puree | GameVisit*Group | Veg, Week 1-Baseline | -3.8333 | 6.837 | 64 | -0.56 | 0.577 |
| Broccoli puree | GameVisit*Group | Veg - Control, Baseline | 6.8868 | 12.1769 | 49.41 | 0.57 | 0.5743 |
| Broccoli puree | GameVisit*Group | Veg - Control, Week 1 | 9.241 | 12.1769 | 49.41 | 0.76 | 0.4515 |
| Broccoli puree | GameVisit*Group | Veg - Control, Week 2 | 5.1576 | 12.1769 | 49.41 | 0.42 | 0.6737 |
| Chicken puree | GameVisit*Group | Control, Week 2-Baseline | 6.5 | 8.3313 | 64 | 0.78 | 0.4382 |
| Chicken puree | GameVisit*Group | Control, Week 1-Baseline | 6.4375 | 8.3313 | 64 | 0.77 | 0.4426 |
| Chicken puree | GameVisit*Group | Veg, Week 2-Baseline | 10.9444 | 7.8548 | 64 | 1.39 | 0.1683 |
| Chicken puree | GameVisit*Group | Veg, Week 1-Baseline | 9.0556 | 7.8548 | 64 | 1.15 | 0.2533 |
| Chicken puree | GameVisit*Group | Veg - Control, Baseline | -15.6868 | 19.5454 | 39.21 | -0.8 | 0.4271 |
| Chicken puree | GameVisit*Group | Veg - Control, Week 1 | -13.0688 | 19.5454 | 39.21 | -0.67 | 0.5076 |
| Chicken puree | GameVisit*Group | Veg - Control, Week 2 | -11.2424 | 19.5454 | 39.21 | -0.58 | 0.5684 |
| Kale chopped | GameVisit*Group | Control, Week 2-Baseline | -9.375 | 7.5329 | 64 | -1.24 | 0.2178 |
| Kale chopped | GameVisit*Group | Control, Week 1-Baseline | -7.125 | 7.5329 | 64 | -0.95 | 0.3478 |
| Kale chopped | GameVisit*Group | Veg, Week 2-Baseline | 19.6111 | 7.1021 | 64 | 2.76 | 0.0075 |
| Kale chopped | GameVisit*Group | Veg, Week 1-Baseline | 17.5556 | 7.1021 | 64 | 2.47 | 0.0161 |
| Kale chopped | GameVisit*Group | Veg - Control, Baseline | -15.3106 | 12.6922 | 49.25 | -1.21 | 0.2335 |
| Kale chopped | GameVisit*Group | Veg - Control, Week 1 | 9.3699 | 12.6922 | 49.25 | 0.74 | 0.4639 |
| Kale chopped | GameVisit*Group | Veg - Control, Week 2 | 13.6755 | 12.6922 | 49.25 | 1.08 | 0.2865 |
| Kale puree | GameVisit*Group | Control, Week 2-Baseline | -19.125 | 7.224 | 64 | -2.65 | 0.0102 |
| Kale puree | GameVisit*Group | Control, Week 1-Baseline | -14.75 | 7.224 | 64 | -2.04 | 0.0453 |
| Kale puree | GameVisit*Group | Veg, Week 2-Baseline | 10.4444 | 6.8109 | 64 | 1.53 | 0.1301 |
| Kale puree | GameVisit*Group | Veg, Week 1-Baseline | 2.7778 | 6.8109 | 64 | 0.41 | 0.6847 |
| Kale puree | GameVisit*Group | Veg - Control, Baseline | -11.6296 | 11.6043 | 51.64 | -1 | 0.3209 |
| Kale puree | GameVisit*Group | Veg - Control, Week 1 | 5.8982 | 11.6043 | 51.64 | 0.51 | 0.6134 |
| Kale puree | GameVisit*Group | Veg - Control, Week 2 | 17.9399 | 11.6043 | 51.64 | 1.55 | 0.1282 |
| Oat puree | GameVisit*Group | Control, Week 2-Baseline | 11.75 | 8.8707 | 64 | 1.32 | 0.19 |
| Oat puree | GameVisit*Group | Control, Week 1-Baseline | 6.625 | 8.8707 | 64 | 0.75 | 0.4579 |
| Oat puree | GameVisit*Group | Veg, Week 2-Baseline | 16.9444 | 8.3634 | 64 | 2.03 | 0.0469 |
| Oat puree | GameVisit*Group | Veg, Week 1-Baseline | 1.7222 | 8.3634 | 64 | 0.21 | 0.8375 |
| Oat puree | GameVisit*Group | Veg - Control, Baseline | 16.3913 | 11.9348 | 64.06 | 1.37 | 0.1744 |
| Oat puree | GameVisit*Group | Veg - Control, Week 1 | 11.4885 | 11.9348 | 64.06 | 0.96 | 0.3394 |
| Oat puree | GameVisit*Group | Veg - Control, Week 2 | 21.5858 | 11.9348 | 64.06 | 1.81 | 0.0752 |
| Spinach chopped | GameVisit*Group | Control, Week 2-Baseline | 1.5 | 7.6113 | 64 | 0.2 | 0.8444 |
| Spinach chopped | GameVisit*Group | Control, Week 1-Baseline | -2.375 | 7.6113 | 64 | -0.31 | 0.756 |
| Spinach chopped | GameVisit*Group | Veg, Week 2-Baseline | 12.1667 | 7.176 | 64 | 1.7 | 0.0948 |
| Spinach chopped | GameVisit*Group | Veg, Week 1-Baseline | -0.5 | 7.176 | 64 | -0.07 | 0.9447 |
| Spinach chopped | GameVisit*Group | Veg - Control, Baseline | 0.5259 | 12.1892 | 51.8 | 0.04 | 0.9658 |
| Spinach chopped | GameVisit*Group | Veg - Control, Week 1 | 2.4009 | 12.1892 | 51.8 | 0.2 | 0.8446 |
| Spinach chopped | GameVisit*Group | Veg - Control, Week 2 | 11.1926 | 12.1892 | 51.8 | 0.92 | 0.3628 |
| Spinach puree | GameVisit*Group | Control, Week 2-Baseline | -7.9375 | 7.1066 | 64 | -1.12 | 0.2682 |
| Spinach puree | GameVisit*Group | Control, Week 1-Baseline | -3.9375 | 7.1066 | 64 | -0.55 | 0.5815 |
| Spinach puree | GameVisit*Group | Veg, Week 2-Baseline | 0.7222 | 6.7002 | 64 | 0.11 | 0.9145 |
| Spinach puree | GameVisit*Group | Veg, Week 1-Baseline | -4.4444 | 6.7002 | 64 | -0.66 | 0.5095 |
| Spinach puree | GameVisit*Group | Veg - Control, Baseline | -0.6393 | 9.3422 | 66.19 | -0.07 | 0.9456 |
| Spinach puree | GameVisit*Group | Veg - Control, Week 1 | -1.1463 | 9.3422 | 66.19 | -0.12 | 0.9027 |
| Spinach puree | GameVisit*Group | Veg - Control, Week 2 | 8.0204 | 9.3422 | 66.19 | 0.86 | 0.3937 |

## Liking Least Squared Means

P-values in this table indicate whether the estimate is significantly different from 0.

| **SampleName** | **Effect** | **GameVisit** | **Group** | **Estimate** | **StdErr** | **DF** | **tValue** | **Probt** |
| --- | --- | --- | --- | --- | --- | --- | --- | --- |
| Asparagus chopped | Group | _ | Vegetable | 0.04015 | 8.7226 | 31 | 0 | 0.9964 |
| Asparagus chopped | Group | _ | Control | 13.2972 | 8.5894 | 31 | 1.55 | 0.1317 |
| Asparagus chopped | GameVisit | Baseline |  | 5.3273 | 7.0001 | 44.7 | 0.76 | 0.4506 |
| Asparagus chopped | GameVisit | Week 1 |  | 10.105 | 7.0001 | 44.7 | 1.44 | 0.1558 |
| Asparagus chopped | GameVisit | Week 2 |  | 4.5738 | 7.0001 | 44.7 | 0.65 | 0.5168 |
| Asparagus chopped | GameVisit*Group | Baseline | Vegetable | -5.9969 | 9.6111 | 44.7 | -0.62 | 0.5358 |
| Asparagus chopped | GameVisit*Group | Baseline | Control | 16.6514 | 9.5971 | 46.9 | 1.74 | 0.0893 |
| Asparagus chopped | GameVisit*Group | Week 1 | Vegetable | 9.0587 | 9.6111 | 44.7 | 0.94 | 0.351 |
| Asparagus chopped | GameVisit*Group | Week 1 | Control | 11.1514 | 9.5971 | 46.9 | 1.16 | 0.2511 |
| Asparagus chopped | GameVisit*Group | Week 2 | Vegetable | -2.9413 | 9.6111 | 44.7 | -0.31 | 0.761 |
| Asparagus chopped | GameVisit*Group | Week 2 | Control | 12.0889 | 9.5971 | 46.9 | 1.26 | 0.214 |
| Asparagus puree | Group | _ | Vegetable | -2.2058 | 8.7658 | 31 | -0.25 | 0.803 |
| Asparagus puree | Group | _ | Control | 12.6991 | 8.632 | 31 | 1.47 | 0.1513 |
| Asparagus puree | GameVisit | Baseline |  | 2.9943 | 7.1252 | 46.8 | 0.42 | 0.6762 |
| Asparagus puree | GameVisit | Week 1 |  | 6.6089 | 7.1252 | 46.8 | 0.93 | 0.3584 |
| Asparagus puree | GameVisit | Week 2 |  | 6.1367 | 7.1252 | 46.8 | 0.86 | 0.3935 |
| Asparagus puree | GameVisit*Group | Baseline | Vegetable | -5.3354 | 9.7827 | 46.7 | -0.55 | 0.5881 |
| Asparagus puree | GameVisit*Group | Baseline | Control | 11.3241 | 9.7842 | 49.2 | 1.16 | 0.2527 |
| Asparagus puree | GameVisit*Group | Week 1 | Vegetable | -3.1688 | 9.7827 | 46.7 | -0.32 | 0.7474 |
| Asparagus puree | GameVisit*Group | Week 1 | Control | 16.3866 | 9.7842 | 49.2 | 1.67 | 0.1003 |
| Asparagus puree | GameVisit*Group | Week 2 | Vegetable | 1.8868 | 9.7827 | 46.7 | 0.19 | 0.8479 |
| Asparagus puree | GameVisit*Group | Week 2 | Control | 10.3866 | 9.7842 | 49.2 | 1.06 | 0.2936 |
| Beef puree | Group | _ | Vegetable | -15.6653 | 13.9768 | 31 | -1.12 | 0.271 |
| Beef puree | Group | _ | Control | -1.2827 | 13.7634 | 31 | -0.09 | 0.9263 |
| Beef puree | GameVisit | Baseline |  | -10.6118 | 10.5052 | 35.1 | -1.01 | 0.3193 |
| Beef puree | GameVisit | Week 1 |  | -11.6013 | 10.5052 | 35.1 | -1.1 | 0.277 |
| Beef puree | GameVisit | Week 2 |  | -3.209 | 10.5052 | 35.1 | -0.31 | 0.7618 |
| Beef puree | GameVisit*Group | Baseline | Vegetable | -16.6283 | 14.425 | 35.1 | -1.15 | 0.2568 |
| Beef puree | GameVisit*Group | Baseline | Control | -4.5952 | 14.2742 | 35.8 | -0.32 | 0.7494 |
| Beef puree | GameVisit*Group | Week 1 | Vegetable | -21.2949 | 14.425 | 35.1 | -1.48 | 0.1488 |
| Beef puree | GameVisit*Group | Week 1 | Control | -1.9077 | 14.2742 | 35.8 | -0.13 | 0.8944 |
| Beef puree | GameVisit*Group | Week 2 | Vegetable | -9.0727 | 14.425 | 35.1 | -0.63 | 0.5334 |
| Beef puree | GameVisit*Group | Week 2 | Control | 2.6548 | 14.2742 | 35.8 | 0.19 | 0.8535 |
| Black bean puree | Group | _ | Vegetable | -6.8546 | 10.0115 | 31 | -0.68 | 0.4986 |
| Black bean puree | Group | _ | Control | 6.7446 | 9.8587 | 31 | 0.68 | 0.499 |
| Black bean puree | GameVisit | Baseline |  | -5.1036 | 8.2934 | 49.8 | -0.62 | 0.5411 |
| Black bean puree | GameVisit | Week 1 |  | 2.3651 | 8.2934 | 49.8 | 0.29 | 0.7767 |
| Black bean puree | GameVisit | Week 2 |  | 2.5735 | 8.2934 | 49.8 | 0.31 | 0.7576 |
| Black bean puree | GameVisit*Group | Baseline | Vegetable | -10.4101 | 11.3863 | 49.8 | -0.91 | 0.365 |
| Black bean puree | GameVisit*Group | Baseline | Control | 0.2029 | 11.4144 | 52.7 | 0.02 | 0.9859 |
| Black bean puree | GameVisit*Group | Week 1 | Vegetable | -6.4101 | 11.3863 | 49.8 | -0.56 | 0.576 |
| Black bean puree | GameVisit*Group | Week 1 | Control | 11.1404 | 11.4144 | 52.7 | 0.98 | 0.3335 |
| Black bean puree | GameVisit*Group | Week 2 | Vegetable | -3.7435 | 11.3863 | 49.8 | -0.33 | 0.7437 |
| Black bean puree | GameVisit*Group | Week 2 | Control | 8.8904 | 11.4144 | 52.7 | 0.78 | 0.4395 |
| Broccoli chopped | Group | _ | Vegetable | 7.4052 | 9.575 | 31 | 0.77 | 0.4452 |
| Broccoli chopped | Group | _ | Control | 16.9032 | 9.4288 | 31 | 1.79 | 0.0828 |
| Broccoli chopped | GameVisit | Baseline |  | 10.6067 | 7.6913 | 44.9 | 1.38 | 0.1747 |
| Broccoli chopped | GameVisit | Week 1 |  | 14.454 | 7.6913 | 44.9 | 1.88 | 0.0667 |
| Broccoli chopped | GameVisit | Week 2 |  | 11.4019 | 7.6913 | 44.9 | 1.48 | 0.1452 |
| Broccoli chopped | GameVisit*Group | Baseline | Vegetable | 8.1645 | 10.5601 | 44.8 | 0.77 | 0.4435 |
| Broccoli chopped | GameVisit*Group | Baseline | Control | 13.049 | 10.546 | 47.1 | 1.24 | 0.2221 |
| Broccoli chopped | GameVisit*Group | Week 1 | Vegetable | 7.6089 | 10.5601 | 44.8 | 0.72 | 0.4749 |
| Broccoli chopped | GameVisit*Group | Week 1 | Control | 21.299 | 10.546 | 47.1 | 2.02 | 0.0491 |
| Broccoli chopped | GameVisit*Group | Week 2 | Vegetable | 6.4422 | 10.5601 | 44.8 | 0.61 | 0.5449 |
| Broccoli chopped | GameVisit*Group | Week 2 | Control | 16.3615 | 10.546 | 47.1 | 1.55 | 0.1275 |
| Broccoli puree | Group | _ | Vegetable | 14.3775 | 7.9587 | 31 | 1.81 | 0.0806 |
| Broccoli puree | Group | _ | Control | 7.2824 | 7.8372 | 31 | 0.93 | 0.36 |
| Broccoli puree | GameVisit | Baseline |  | 12.1008 | 6.4705 | 46.8 | 1.87 | 0.0677 |
| Broccoli puree | GameVisit | Week 1 |  | 7.0904 | 6.4705 | 46.8 | 1.1 | 0.2788 |
| Broccoli puree | GameVisit | Week 2 |  | 13.2987 | 6.4705 | 46.8 | 2.06 | 0.0455 |
| Broccoli puree | GameVisit*Group | Baseline | Vegetable | 15.5442 | 8.8839 | 46.8 | 1.75 | 0.0867 |
| Broccoli puree | GameVisit*Group | Baseline | Control | 8.6574 | 8.8855 | 49.3 | 0.97 | 0.3347 |
| Broccoli puree | GameVisit*Group | Week 1 | Vegetable | 11.7109 | 8.8839 | 46.8 | 1.32 | 0.1939 |
| Broccoli puree | GameVisit*Group | Week 1 | Control | 2.4699 | 8.8855 | 49.3 | 0.28 | 0.7822 |
| Broccoli puree | GameVisit*Group | Week 2 | Vegetable | 15.8775 | 8.8839 | 46.8 | 1.79 | 0.0804 |
| Broccoli puree | GameVisit*Group | Week 2 | Control | 10.7199 | 8.8855 | 49.3 | 1.21 | 0.2334 |
| Chicken puree | Group | _ | Vegetable | -14.3094 | 13.641 | 31 | -1.05 | 0.3023 |
| Chicken puree | Group | _ | Control | -0.9767 | 13.4327 | 31 | -0.07 | 0.9425 |
| Chicken puree | GameVisit | Baseline |  | -13.1326 | 10.4691 | 38 | -1.25 | 0.2173 |
| Chicken puree | GameVisit | Week 1 |  | -5.3861 | 10.4691 | 38 | -0.51 | 0.6099 |
| Chicken puree | GameVisit | Week 2 |  | -4.4104 | 10.4691 | 38 | -0.42 | 0.6759 |
| Chicken puree | GameVisit*Group | Baseline | Vegetable | -20.976 | 14.375 | 38 | -1.46 | 0.1527 |
| Chicken puree | GameVisit*Group | Baseline | Control | -5.2892 | 14.2679 | 39.1 | -0.37 | 0.7129 |
| Chicken puree | GameVisit*Group | Week 1 | Vegetable | -11.9205 | 14.375 | 38 | -0.83 | 0.4121 |
| Chicken puree | GameVisit*Group | Week 1 | Control | 1.1483 | 14.2679 | 39.1 | 0.08 | 0.9363 |
| Chicken puree | GameVisit*Group | Week 2 | Vegetable | -10.0316 | 14.375 | 38 | -0.7 | 0.4895 |
| Chicken puree | GameVisit*Group | Week 2 | Control | 1.2108 | 14.2679 | 39.1 | 0.08 | 0.9328 |
| Kale chopped | Group | _ | Vegetable | -15.7213 | 8.3036 | 31 | -1.89 | 0.0677 |
| Kale chopped | Group | _ | Control | -18.2996 | 8.1768 | 31 | -2.24 | 0.0325 |
| Kale chopped | GameVisit | Baseline |  | -20.4549 | 6.7451 | 46.6 | -3.03 | 0.004 |
| Kale chopped | GameVisit | Week 1 |  | -15.2396 | 6.7451 | 46.6 | -2.26 | 0.0286 |
| Kale chopped | GameVisit | Week 2 |  | -15.3368 | 6.7451 | 46.6 | -2.27 | 0.0276 |
| Kale chopped | GameVisit*Group | Baseline | Vegetable | -28.1102 | 9.2608 | 46.6 | -3.04 | 0.0039 |
| Kale chopped | GameVisit*Group | Baseline | Control | -12.7996 | 9.2615 | 49.1 | -1.38 | 0.1732 |
| Kale chopped | GameVisit*Group | Week 1 | Vegetable | -10.5547 | 9.2608 | 46.6 | -1.14 | 0.2602 |
| Kale chopped | GameVisit*Group | Week 1 | Control | -19.9246 | 9.2615 | 49.1 | -2.15 | 0.0364 |
| Kale chopped | GameVisit*Group | Week 2 | Vegetable | -8.4991 | 9.2608 | 46.6 | -0.92 | 0.3635 |
| Kale chopped | GameVisit*Group | Week 2 | Control | -22.1746 | 9.2615 | 49.1 | -2.39 | 0.0205 |
| Kale puree | Group | _ | Vegetable | -13.0327 | 7.4827 | 31 | -1.74 | 0.0915 |
| Kale puree | Group | _ | Control | -17.1022 | 7.3685 | 31 | -2.32 | 0.027 |
| Kale puree | GameVisit | Baseline |  | -11.6253 | 6.1569 | 48.7 | -1.89 | 0.065 |
| Kale puree | GameVisit | Week 1 |  | -17.6115 | 6.1569 | 48.7 | -2.86 | 0.0062 |
| Kale puree | GameVisit | Week 2 |  | -15.9656 | 6.1569 | 48.7 | -2.59 | 0.0125 |
| Kale puree | GameVisit*Group | Baseline | Vegetable | -17.4401 | 8.4531 | 48.7 | -2.06 | 0.0444 |
| Kale puree | GameVisit*Group | Baseline | Control | -5.8106 | 8.467 | 51.5 | -0.69 | 0.4956 |
| Kale puree | GameVisit*Group | Week 1 | Vegetable | -14.6624 | 8.4531 | 48.7 | -1.73 | 0.0891 |
| Kale puree | GameVisit*Group | Week 1 | Control | -20.5606 | 8.467 | 51.5 | -2.43 | 0.0187 |
| Kale puree | GameVisit*Group | Week 2 | Vegetable | -6.9957 | 8.4531 | 48.7 | -0.83 | 0.4119 |
| Kale puree | GameVisit*Group | Week 2 | Control | -24.9356 | 8.467 | 51.5 | -2.95 | 0.0048 |
| Oat puree | Group | _ | Vegetable | 2.5928 | 7.1479 | 31 | 0.36 | 0.7193 |
| Oat puree | Group | _ | Control | -13.8957 | 7.0388 | 31 | -1.97 | 0.0573 |
| Oat puree | GameVisit | Baseline |  | -11.8251 | 6.2834 | 59.8 | -1.88 | 0.0647 |
| Oat puree | GameVisit | Week 1 |  | -7.6515 | 6.2834 | 59.8 | -1.22 | 0.2281 |
| Oat puree | GameVisit | Week 2 |  | 2.5221 | 6.2834 | 59.8 | 0.4 | 0.6896 |
| Oat puree | GameVisit*Group | Baseline | Vegetable | -3.6294 | 8.626 | 59.7 | -0.42 | 0.6754 |
| Oat puree | GameVisit*Group | Baseline | Control | -20.0207 | 8.7048 | 63.8 | -2.3 | 0.0247 |
| Oat puree | GameVisit*Group | Week 1 | Vegetable | -1.9072 | 8.626 | 59.7 | -0.22 | 0.8258 |
| Oat puree | GameVisit*Group | Week 1 | Control | -13.3957 | 8.7048 | 63.8 | -1.54 | 0.1288 |
| Oat puree | GameVisit*Group | Week 2 | Vegetable | 13.315 | 8.626 | 59.7 | 1.54 | 0.128 |
| Oat puree | GameVisit*Group | Week 2 | Control | -8.2707 | 8.7048 | 63.8 | -0.95 | 0.3456 |
| Spinach chopped | Group | _ | Vegetable | -2.8367 | 7.8521 | 31 | -0.36 | 0.7203 |
| Spinach chopped | Group | _ | Control | -7.5432 | 7.7322 | 31 | -0.98 | 0.3368 |
| Spinach chopped | GameVisit | Baseline |  | -6.9886 | 6.4665 | 48.9 | -1.08 | 0.2851 |
| Spinach chopped | GameVisit | Week 1 |  | -8.4261 | 6.4665 | 48.9 | -1.3 | 0.1987 |
| Spinach chopped | GameVisit | Week 2 |  | -0.1552 | 6.4665 | 48.9 | -0.02 | 0.9809 |
| Spinach chopped | GameVisit*Group | Baseline | Vegetable | -6.7256 | 8.8781 | 48.8 | -0.76 | 0.4524 |
| Spinach chopped | GameVisit*Group | Baseline | Control | -7.2515 | 8.8937 | 51.6 | -0.82 | 0.4186 |
| Spinach chopped | GameVisit*Group | Week 1 | Vegetable | -7.2256 | 8.8781 | 48.8 | -0.81 | 0.4197 |
| Spinach chopped | GameVisit*Group | Week 1 | Control | -9.6265 | 8.8937 | 51.6 | -1.08 | 0.2841 |
| Spinach chopped | GameVisit*Group | Week 2 | Vegetable | 5.4411 | 8.8781 | 48.8 | 0.61 | 0.5428 |
| Spinach chopped | GameVisit*Group | Week 2 | Control | -5.7515 | 8.8937 | 51.6 | -0.65 | 0.5207 |
| Spinach puree | Group | _ | Vegetable | -9.4713 | 5.5239 | 31 | -1.71 | 0.0964 |
| Spinach puree | Group | _ | Control | -11.5496 | 5.4395 | 31 | -2.12 | 0.0418 |
| Spinach puree | GameVisit | Baseline |  | -7.9109 | 4.9123 | 61.7 | -1.61 | 0.1124 |
| Spinach puree | GameVisit | Week 1 |  | -12.1019 | 4.9123 | 61.7 | -2.46 | 0.0166 |
| Spinach puree | GameVisit | Week 2 |  | -11.5186 | 4.9123 | 61.7 | -2.34 | 0.0223 |
| Spinach puree | GameVisit*Group | Baseline | Vegetable | -8.2306 | 6.7437 | 61.7 | -1.22 | 0.2269 |
| Spinach puree | GameVisit*Group | Baseline | Control | -7.5913 | 6.8135 | 66 | -1.11 | 0.2693 |
| Spinach puree | GameVisit*Group | Week 1 | Vegetable | -12.675 | 6.7437 | 61.7 | -1.88 | 0.0649 |
| Spinach puree | GameVisit*Group | Week 1 | Control | -11.5288 | 6.8135 | 66 | -1.69 | 0.0954 |
| Spinach puree | GameVisit*Group | Week 2 | Vegetable | -7.5084 | 6.7437 | 61.7 | -1.11 | 0.2699 |
| Spinach puree | GameVisit*Group | Week 2 | Control | -15.5288 | 6.8135 | 66 | -2.28 | 0.0259 |

## Sweetness Type 3 effects

P-values (ProbF) in this table indicate whether the effect is significant in the overall model.

| **SampleName** | **Effect** | **NumDF** | **DenDF** | **FValue** | **ProbF** |
| --- | --- | --- | --- | --- | --- |
| Asparagus chopped | GameVisit | 2 | 64 | 0.31 | 0.7363 |
| Asparagus chopped | Group | 1 | 31 | 0.53 | 0.4703 |
| Asparagus chopped | GameVisit*Group | 2 | 64 | 0.13 | 0.8751 |
| Asparagus chopped | Gender | 1 | 31 | 0.27 | 0.6098 |
| Asparagus puree | GameVisit | 2 | 64 | 0.18 | 0.8367 |
| Asparagus puree | Group | 1 | 31 | 0.3 | 0.5861 |
| Asparagus puree | GameVisit*Group | 2 | 64 | 2.94 | 0.0599 |
| Asparagus puree | Gender | 1 | 31 | 0.27 | 0.6102 |
| Beef puree | GameVisit | 2 | 64 | 2.44 | 0.0952 |
| Beef puree | Group | 1 | 31 | 0.11 | 0.7429 |
| Beef puree | GameVisit*Group | 2 | 64 | 0.15 | 0.8605 |
| Beef puree | Gender | 1 | 31 | 0 | 0.9495 |
| Black bean puree | GameVisit | 2 | 64 | 1.92 | 0.1544 |
| Black bean puree | Group | 1 | 31 | 0.01 | 0.9041 |
| Black bean puree | GameVisit*Group | 2 | 64 | 1.12 | 0.3341 |
| Black bean puree | Gender | 1 | 31 | 0 | 0.9651 |
| Broccoli chopped | GameVisit | 2 | 64 | 0.2 | 0.8227 |
| Broccoli chopped | Group | 1 | 31 | 0.04 | 0.8428 |
| Broccoli chopped | GameVisit*Group | 2 | 64 | 0.83 | 0.4406 |
| Broccoli chopped | Gender | 1 | 31 | 3.72 | 0.0631 |
| Broccoli puree | GameVisit | 2 | 64 | 0.06 | 0.9431 |
| Broccoli puree | Group | 1 | 31 | 0.58 | 0.4503 |
| Broccoli puree | GameVisit*Group | 2 | 64 | 0.17 | 0.8464 |
| Broccoli puree | Gender | 1 | 31 | 0.42 | 0.5213 |
| Chicken puree | GameVisit | 2 | 64 | 0.19 | 0.8292 |
| Chicken puree | Group | 1 | 31 | 0.08 | 0.7852 |
| Chicken puree | GameVisit*Group | 2 | 64 | 1.25 | 0.2946 |
| Chicken puree | Gender | 1 | 31 | 0.01 | 0.9422 |
| Kale chopped | GameVisit | 2 | 64 | 0.29 | 0.7487 |
| Kale chopped | Group | 1 | 31 | 0.02 | 0.8768 |
| Kale chopped | GameVisit*Group | 2 | 64 | 2.09 | 0.1316 |
| Kale chopped | Gender | 1 | 31 | 0 | 0.9769 |
| Kale puree | GameVisit | 2 | 64 | 0.04 | 0.9635 |
| Kale puree | Group | 1 | 31 | 0.18 | 0.6701 |
| Kale puree | GameVisit*Group | 2 | 64 | 0.45 | 0.6386 |
| Kale puree | Gender | 1 | 31 | 0.36 | 0.5552 |
| Oat puree | GameVisit | 2 | 64 | 0.6 | 0.5514 |
| Oat puree | Group | 1 | 31 | 0.14 | 0.7067 |
| Oat puree | GameVisit*Group | 2 | 64 | 0.62 | 0.5429 |
| Oat puree | Gender | 1 | 31 | 0.66 | 0.4239 |
| Spinach chopped | GameVisit | 2 | 64 | 4.5 | 0.0148 |
| Spinach chopped | Group | 1 | 31 | 0.08 | 0.783 |
| Spinach chopped | GameVisit*Group | 2 | 64 | 1.17 | 0.3179 |
| Spinach chopped | Gender | 1 | 31 | 0.47 | 0.4983 |
| Spinach puree | GameVisit | 2 | 64 | 1.26 | 0.2899 |
| Spinach puree | Group | 1 | 31 | 0.03 | 0.8606 |
| Spinach puree | GameVisit*Group | 2 | 64 | 1.56 | 0.2177 |
| Spinach puree | Gender | 1 | 31 | 0.4 | 0.5293 |

## Sweetness: Contrasts

P-values in this table indicate whether the estimate of the difference between the two means is significantly different from 0.

| **SampleName** | **Effect** | **Label** | **Estimate** | **StdErr** | **DF** | **tValue** | **Probt** |
| --- | --- | --- | --- | --- | --- | --- | --- |
| Asparagus chopped | GameVisit*Group | Control, Week 2-Baseline | 0.625 | 3.9064 | 64 | 0.16 | 0.8734 |
| Asparagus chopped | GameVisit*Group | Control, Week 1-Baseline | -0.875 | 3.9064 | 64 | -0.22 | 0.8235 |
| Asparagus chopped | GameVisit*Group | Veg, Week 2-Baseline | -1.8333 | 3.683 | 64 | -0.5 | 0.6203 |
| Asparagus chopped | GameVisit*Group | Veg, Week 1-Baseline | -3.2222 | 3.683 | 64 | -0.87 | 0.3849 |
| Asparagus chopped | GameVisit*Group | Veg - Control, Baseline | -2.1593 | 6.0067 | 54.13 | -0.36 | 0.7206 |
| Asparagus chopped | GameVisit*Group | Veg - Control, Week 1 | -4.5065 | 6.0067 | 54.13 | -0.75 | 0.4564 |
| Asparagus chopped | GameVisit*Group | Veg - Control, Week 2 | -4.6176 | 6.0067 | 54.13 | -0.77 | 0.4454 |
| Asparagus puree | GameVisit*Group | Control, Week 2-Baseline | -3.6875 | 4.1994 | 64 | -0.88 | 0.3832 |
| Asparagus puree | GameVisit*Group | Control, Week 1-Baseline | 5 | 4.1994 | 64 | 1.19 | 0.2382 |
| Asparagus puree | GameVisit*Group | Veg, Week 2-Baseline | 2.4444 | 3.9592 | 64 | 0.62 | 0.5392 |
| Asparagus puree | GameVisit*Group | Veg, Week 1-Baseline | -2.8333 | 3.9592 | 64 | -0.72 | 0.4768 |
| Asparagus puree | GameVisit*Group | Veg - Control, Baseline | -2.9956 | 7.2821 | 47.97 | -0.41 | 0.6826 |
| Asparagus puree | GameVisit*Group | Veg - Control, Week 1 | -10.829 | 7.2821 | 47.97 | -1.49 | 0.1435 |
| Asparagus puree | GameVisit*Group | Veg - Control, Week 2 | 3.1363 | 7.2821 | 47.97 | 0.43 | 0.6686 |
| Beef puree | GameVisit*Group | Control, Week 2-Baseline | 4.9375 | 3.1508 | 64 | 1.57 | 0.122 |
| Beef puree | GameVisit*Group | Control, Week 1-Baseline | 4 | 3.1508 | 64 | 1.27 | 0.2089 |
| Beef puree | GameVisit*Group | Veg, Week 2-Baseline | 4.5556 | 2.9706 | 64 | 1.53 | 0.1301 |
| Beef puree | GameVisit*Group | Veg, Week 1-Baseline | 1.7778 | 2.9706 | 64 | 0.6 | 0.5516 |
| Beef puree | GameVisit*Group | Veg - Control, Baseline | 2.8593 | 6.5151 | 42.02 | 0.44 | 0.663 |
| Beef puree | GameVisit*Group | Veg - Control, Week 1 | 0.6371 | 6.5151 | 42.02 | 0.1 | 0.9226 |
| Beef puree | GameVisit*Group | Veg - Control, Week 2 | 2.4773 | 6.5151 | 42.02 | 0.38 | 0.7057 |
| Black bean puree | GameVisit*Group | Control, Week 2-Baseline | 6.8125 | 4.0046 | 64 | 1.7 | 0.0938 |
| Black bean puree | GameVisit*Group | Control, Week 1-Baseline | -2.0625 | 4.0046 | 64 | -0.52 | 0.6083 |
| Black bean puree | GameVisit*Group | Veg, Week 2-Baseline | 2.3333 | 3.7756 | 64 | 0.62 | 0.5388 |
| Black bean puree | GameVisit*Group | Veg, Week 1-Baseline | 1.6667 | 3.7756 | 64 | 0.44 | 0.6604 |
| Black bean puree | GameVisit*Group | Veg - Control, Baseline | -0.4376 | 6.4935 | 51.14 | -0.07 | 0.9465 |
| Black bean puree | GameVisit*Group | Veg - Control, Week 1 | 3.2916 | 6.4935 | 51.14 | 0.51 | 0.6144 |
| Black bean puree | GameVisit*Group | Veg - Control, Week 2 | -4.9168 | 6.4935 | 51.14 | -0.76 | 0.4524 |
| Broccoli chopped | GameVisit*Group | Control, Week 2-Baseline | 2.1875 | 4.2158 | 64 | 0.52 | 0.6056 |
| Broccoli chopped | GameVisit*Group | Control, Week 1-Baseline | 1.875 | 4.2158 | 64 | 0.44 | 0.658 |
| Broccoli chopped | GameVisit*Group | Veg, Week 2-Baseline | -5.2778 | 3.9747 | 64 | -1.33 | 0.1889 |
| Broccoli chopped | GameVisit*Group | Veg, Week 1-Baseline | -1.7778 | 3.9747 | 64 | -0.45 | 0.6562 |
| Broccoli chopped | GameVisit*Group | Veg - Control, Baseline | 4.8815 | 6.7635 | 51.7 | 0.72 | 0.4737 |
| Broccoli chopped | GameVisit*Group | Veg - Control, Week 1 | 1.2287 | 6.7635 | 51.7 | 0.18 | 0.8566 |
| Broccoli chopped | GameVisit*Group | Veg - Control, Week 2 | -2.5838 | 6.7635 | 51.7 | -0.38 | 0.704 |
| Broccoli puree | GameVisit*Group | Control, Week 2-Baseline | 2.375 | 4.086 | 64 | 0.58 | 0.5631 |
| Broccoli puree | GameVisit*Group | Control, Week 1-Baseline | 0.3125 | 4.086 | 64 | 0.08 | 0.9393 |
| Broccoli puree | GameVisit*Group | Veg, Week 2-Baseline | -0.6667 | 3.8523 | 64 | -0.17 | 0.8632 |
| Broccoli puree | GameVisit*Group | Veg, Week 1-Baseline | -0.2222 | 3.8523 | 64 | -0.06 | 0.9542 |
| Broccoli puree | GameVisit*Group | Veg - Control, Baseline | 6.2596 | 7.3778 | 46.33 | 0.85 | 0.4006 |
| Broccoli puree | GameVisit*Group | Veg - Control, Week 1 | 5.7249 | 7.3778 | 46.33 | 0.78 | 0.4417 |
| Broccoli puree | GameVisit*Group | Veg - Control, Week 2 | 3.2179 | 7.3778 | 46.33 | 0.44 | 0.6647 |
| Chicken puree | GameVisit*Group | Control, Week 2-Baseline | 2.375 | 2.7964 | 64 | 0.85 | 0.3989 |
| Chicken puree | GameVisit*Group | Control, Week 1-Baseline | 4.1875 | 2.7964 | 64 | 1.5 | 0.1392 |
| Chicken puree | GameVisit*Group | Veg, Week 2-Baseline | -1.2778 | 2.6365 | 64 | -0.48 | 0.6296 |
| Chicken puree | GameVisit*Group | Veg, Week 1-Baseline | -1.8333 | 2.6365 | 64 | -0.7 | 0.4893 |
| Chicken puree | GameVisit*Group | Veg - Control, Baseline | 4.6584 | 5.6673 | 42.57 | 0.82 | 0.4157 |
| Chicken puree | GameVisit*Group | Veg - Control, Week 1 | -1.3624 | 5.6673 | 42.57 | -0.24 | 0.8112 |
| Chicken puree | GameVisit*Group | Veg - Control, Week 2 | 1.0056 | 5.6673 | 42.57 | 0.18 | 0.86 |
| Kale chopped | GameVisit*Group | Control, Week 2-Baseline | -2.75 | 3.5469 | 64 | -0.78 | 0.441 |
| Kale chopped | GameVisit*Group | Control, Week 1-Baseline | -2.8125 | 3.5469 | 64 | -0.79 | 0.4307 |
| Kale chopped | GameVisit*Group | Veg, Week 2-Baseline | 5 | 3.344 | 64 | 1.5 | 0.1398 |
| Kale chopped | GameVisit*Group | Veg, Week 1-Baseline | 6.5 | 3.344 | 64 | 1.94 | 0.0563 |
| Kale chopped | GameVisit*Group | Veg - Control, Baseline | -4.9061 | 5.7352 | 51.29 | -0.86 | 0.3963 |
| Kale chopped | GameVisit*Group | Veg - Control, Week 1 | 4.4064 | 5.7352 | 51.29 | 0.77 | 0.4458 |
| Kale chopped | GameVisit*Group | Veg - Control, Week 2 | 2.8439 | 5.7352 | 51.29 | 0.5 | 0.6221 |
| Kale puree | GameVisit*Group | Control, Week 2-Baseline | -2.0625 | 3.9019 | 64 | -0.53 | 0.5989 |
| Kale puree | GameVisit*Group | Control, Week 1-Baseline | -0.9375 | 3.9019 | 64 | -0.24 | 0.8109 |
| Kale puree | GameVisit*Group | Veg, Week 2-Baseline | 2.9444 | 3.6787 | 64 | 0.8 | 0.4264 |
| Kale puree | GameVisit*Group | Veg, Week 1-Baseline | 2.3889 | 3.6787 | 64 | 0.65 | 0.5184 |
| Kale puree | GameVisit*Group | Veg - Control, Baseline | -4.8216 | 5.6718 | 57.85 | -0.85 | 0.3988 |
| Kale puree | GameVisit*Group | Veg - Control, Week 1 | -1.4952 | 5.6718 | 57.85 | -0.26 | 0.793 |
| Kale puree | GameVisit*Group | Veg - Control, Week 2 | 0.1853 | 5.6718 | 57.85 | 0.03 | 0.974 |
| Oat puree | GameVisit*Group | Control, Week 2-Baseline | -1.1875 | 3.9699 | 64 | -0.3 | 0.7658 |
| Oat puree | GameVisit*Group | Control, Week 1-Baseline | -3.125 | 3.9699 | 64 | -0.79 | 0.4341 |
| Oat puree | GameVisit*Group | Veg, Week 2-Baseline | 4.7778 | 3.7428 | 64 | 1.28 | 0.2064 |
| Oat puree | GameVisit*Group | Veg, Week 1-Baseline | 0.7778 | 3.7428 | 64 | 0.21 | 0.836 |
| Oat puree | GameVisit*Group | Veg - Control, Baseline | -1.4439 | 5.7916 | 57.59 | -0.25 | 0.804 |
| Oat puree | GameVisit*Group | Veg - Control, Week 1 | 2.4589 | 5.7916 | 57.59 | 0.42 | 0.6727 |
| Oat puree | GameVisit*Group | Veg - Control, Week 2 | 4.5214 | 5.7916 | 57.59 | 0.78 | 0.4382 |
| Spinach chopped | GameVisit*Group | Control, Week 2-Baseline | 2.6875 | 3.2209 | 64 | 0.83 | 0.4072 |
| Spinach chopped | GameVisit*Group | Control, Week 1-Baseline | -0.3125 | 3.2209 | 64 | -0.1 | 0.923 |
| Spinach chopped | GameVisit*Group | Veg, Week 2-Baseline | 7.2222 | 3.0367 | 64 | 2.38 | 0.0204 |
| Spinach chopped | GameVisit*Group | Veg, Week 1-Baseline | -2.3889 | 3.0367 | 64 | -0.79 | 0.4344 |
| Spinach chopped | GameVisit*Group | Veg - Control, Baseline | 0.5422 | 5.527 | 48.42 | 0.1 | 0.9223 |
| Spinach chopped | GameVisit*Group | Veg - Control, Week 1 | -1.5342 | 5.527 | 48.42 | -0.28 | 0.7825 |
| Spinach chopped | GameVisit*Group | Veg - Control, Week 2 | 5.077 | 5.527 | 48.42 | 0.92 | 0.3629 |
| Spinach puree | GameVisit*Group | Control, Week 2-Baseline | -0.5 | 2.7093 | 64 | -0.18 | 0.8542 |
| Spinach puree | GameVisit*Group | Control, Week 1-Baseline | -0.75 | 2.7093 | 64 | -0.28 | 0.7828 |
| Spinach puree | GameVisit*Group | Veg, Week 2-Baseline | 6 | 2.5543 | 64 | 2.35 | 0.0219 |
| Spinach puree | GameVisit*Group | Veg, Week 1-Baseline | 1.6111 | 2.5543 | 64 | 0.63 | 0.5305 |
| Spinach puree | GameVisit*Group | Veg - Control, Baseline | -3.7317 | 4.8917 | 46.33 | -0.76 | 0.4494 |
| Spinach puree | GameVisit*Group | Veg - Control, Week 1 | -1.3706 | 4.8917 | 46.33 | -0.28 | 0.7806 |
| Spinach puree | GameVisit*Group | Veg - Control, Week 2 | 2.7683 | 4.8917 | 46.33 | 0.57 | 0.5742 |

## Sweetness Least Squared Means

P-values in this table indicate whether the estimate is significantly different from 0.

| **SampleName** | **Effect** | **GameVisit** | **Group** | **Estimate** | **StdErr** | **DF** | **tValue** | **Probt** |
| --- | --- | --- | --- | --- | --- | --- | --- | --- |
| Asparagus chopped | Group | _ | Vegetable | 20.2298 | 3.8157 | 31 | 5.3 | <.0001 |
| Asparagus chopped | Group | _ | Control | 23.9909 | 3.7575 | 31 | 6.38 | <.0001 |
| Asparagus chopped | GameVisit | Baseline |  | 22.9946 | 3.1817 | 50.9 | 7.23 | <.0001 |
| Asparagus chopped | GameVisit | Week 1 |  | 20.946 | 3.1817 | 50.9 | 6.58 | <.0001 |
| Asparagus chopped | GameVisit | Week 2 |  | 22.3904 | 3.1817 | 50.9 | 7.04 | <.0001 |
| Asparagus chopped | GameVisit*Group | Baseline | Vegetable | 21.9149 | 4.3682 | 50.9 | 5.02 | <.0001 |
| Asparagus chopped | GameVisit*Group | Baseline | Control | 24.0742 | 4.3824 | 54 | 5.49 | <.0001 |
| Asparagus chopped | GameVisit*Group | Week 1 | Vegetable | 18.6927 | 4.3682 | 50.9 | 4.28 | <.0001 |
| Asparagus chopped | GameVisit*Group | Week 1 | Control | 23.1992 | 4.3824 | 54 | 5.29 | <.0001 |
| Asparagus chopped | GameVisit*Group | Week 2 | Vegetable | 20.0816 | 4.3682 | 50.9 | 4.6 | <.0001 |
| Asparagus chopped | GameVisit*Group | Week 2 | Control | 24.6992 | 4.3824 | 54 | 5.64 | <.0001 |
| Asparagus puree | Group | _ | Vegetable | 23.8556 | 4.802 | 31 | 4.97 | <.0001 |
| Asparagus puree | Group | _ | Control | 27.4184 | 4.7286 | 31 | 5.8 | <.0001 |
| Asparagus puree | GameVisit | Baseline |  | 25.483 | 3.8735 | 45.5 | 6.58 | <.0001 |
| Asparagus puree | GameVisit | Week 1 |  | 26.5664 | 3.8735 | 45.5 | 6.86 | <.0001 |
| Asparagus puree | GameVisit | Week 2 |  | 24.8615 | 3.8735 | 45.5 | 6.42 | <.0001 |
| Asparagus puree | GameVisit*Group | Baseline | Vegetable | 23.9852 | 5.3183 | 45.5 | 4.51 | <.0001 |
| Asparagus puree | GameVisit*Group | Baseline | Control | 26.9809 | 5.314 | 47.8 | 5.08 | <.0001 |
| Asparagus puree | GameVisit*Group | Week 1 | Vegetable | 21.1519 | 5.3183 | 45.5 | 3.98 | 0.0002 |
| Asparagus puree | GameVisit*Group | Week 1 | Control | 31.9809 | 5.314 | 47.8 | 6.02 | <.0001 |
| Asparagus puree | GameVisit*Group | Week 2 | Vegetable | 26.4297 | 5.3183 | 45.5 | 4.97 | <.0001 |
| Asparagus puree | GameVisit*Group | Week 2 | Control | 23.2934 | 5.314 | 47.8 | 4.38 | <.0001 |
| Beef puree | Group | _ | Vegetable | 16.1053 | 4.4618 | 31 | 3.61 | 0.0011 |
| Beef puree | Group | _ | Control | 14.114 | 4.3937 | 31 | 3.21 | 0.0031 |
| Beef puree | GameVisit | Baseline |  | 12.5645 | 3.4814 | 40.4 | 3.61 | 0.0008 |
| Beef puree | GameVisit | Week 1 |  | 15.4534 | 3.4814 | 40.4 | 4.44 | <.0001 |
| Beef puree | GameVisit | Week 2 |  | 17.311 | 3.4814 | 40.4 | 4.97 | <.0001 |
| Beef puree | GameVisit*Group | Baseline | Vegetable | 13.9942 | 4.7801 | 40.4 | 2.93 | 0.0056 |
| Beef puree | GameVisit*Group | Baseline | Control | 11.1349 | 4.7554 | 41.9 | 2.34 | 0.024 |
| Beef puree | GameVisit*Group | Week 1 | Vegetable | 15.7719 | 4.7801 | 40.4 | 3.3 | 0.002 |
| Beef puree | GameVisit*Group | Week 1 | Control | 15.1349 | 4.7554 | 41.9 | 3.18 | 0.0027 |
| Beef puree | GameVisit*Group | Week 2 | Vegetable | 18.5497 | 4.7801 | 40.4 | 3.88 | 0.0004 |
| Beef puree | GameVisit*Group | Week 2 | Control | 16.0724 | 4.7554 | 41.9 | 3.38 | 0.0016 |
| Black bean puree | Group | _ | Vegetable | 18.4241 | 4.1997 | 31 | 4.39 | 0.0001 |
| Black bean puree | Group | _ | Control | 19.1117 | 4.1356 | 31 | 4.62 | <.0001 |
| Black bean puree | GameVisit | Baseline |  | 17.3095 | 3.4464 | 48.3 | 5.02 | <.0001 |
| Black bean puree | GameVisit | Week 1 |  | 17.1116 | 3.4464 | 48.3 | 4.97 | <.0001 |
| Black bean puree | GameVisit | Week 2 |  | 21.8825 | 3.4464 | 48.3 | 6.35 | <.0001 |
| Black bean puree | GameVisit*Group | Baseline | Vegetable | 17.0908 | 4.7317 | 48.3 | 3.61 | 0.0007 |
| Black bean puree | GameVisit*Group | Baseline | Control | 17.5283 | 4.738 | 51 | 3.7 | 0.0005 |
| Black bean puree | GameVisit*Group | Week 1 | Vegetable | 18.7574 | 4.7317 | 48.3 | 3.96 | 0.0002 |
| Black bean puree | GameVisit*Group | Week 1 | Control | 15.4658 | 4.738 | 51 | 3.26 | 0.002 |
| Black bean puree | GameVisit*Group | Week 2 | Vegetable | 19.4241 | 4.7317 | 48.3 | 4.11 | 0.0002 |
| Black bean puree | GameVisit*Group | Week 2 | Control | 24.3408 | 4.738 | 51 | 5.14 | <.0001 |
| Broccoli chopped | Group | _ | Vegetable | 25.4781 | 4.3595 | 31 | 5.84 | <.0001 |
| Broccoli chopped | Group | _ | Control | 24.3026 | 4.2929 | 31 | 5.66 | <.0001 |
| Broccoli chopped | GameVisit | Baseline |  | 25.3892 | 3.5883 | 48.8 | 7.08 | <.0001 |
| Broccoli chopped | GameVisit | Week 1 |  | 25.4378 | 3.5883 | 48.8 | 7.09 | <.0001 |
| Broccoli chopped | GameVisit | Week 2 |  | 23.8441 | 3.5883 | 48.8 | 6.64 | <.0001 |
| Broccoli chopped | GameVisit*Group | Baseline | Vegetable | 27.8299 | 4.9266 | 48.7 | 5.65 | <.0001 |
| Broccoli chopped | GameVisit*Group | Baseline | Control | 22.9485 | 4.9349 | 51.6 | 4.65 | <.0001 |
| Broccoli chopped | GameVisit*Group | Week 1 | Vegetable | 26.0521 | 4.9266 | 48.7 | 5.29 | <.0001 |
| Broccoli chopped | GameVisit*Group | Week 1 | Control | 24.8235 | 4.9349 | 51.6 | 5.03 | <.0001 |
| Broccoli chopped | GameVisit*Group | Week 2 | Vegetable | 22.5521 | 4.9266 | 48.7 | 4.58 | <.0001 |
| Broccoli chopped | GameVisit*Group | Week 2 | Control | 25.136 | 4.9349 | 51.6 | 5.09 | <.0001 |
| Broccoli puree | Group | _ | Vegetable | 27.5621 | 4.9149 | 31 | 5.61 | <.0001 |
| Broccoli puree | Group | _ | Control | 22.4946 | 4.8399 | 31 | 4.65 | <.0001 |
| Broccoli puree | GameVisit | Baseline |  | 24.7286 | 3.9291 | 44.1 | 6.29 | <.0001 |
| Broccoli puree | GameVisit | Week 1 |  | 24.7737 | 3.9291 | 44.1 | 6.31 | <.0001 |
| Broccoli puree | GameVisit | Week 2 |  | 25.5827 | 3.9291 | 44.1 | 6.51 | <.0001 |
| Broccoli puree | GameVisit*Group | Baseline | Vegetable | 27.8584 | 5.3947 | 44.1 | 5.16 | <.0001 |
| Broccoli puree | GameVisit*Group | Baseline | Control | 21.5988 | 5.3842 | 46.2 | 4.01 | 0.0002 |
| Broccoli puree | GameVisit*Group | Week 1 | Vegetable | 27.6362 | 5.3947 | 44.1 | 5.12 | <.0001 |
| Broccoli puree | GameVisit*Group | Week 1 | Control | 21.9113 | 5.3842 | 46.2 | 4.07 | 0.0002 |
| Broccoli puree | GameVisit*Group | Week 2 | Vegetable | 27.1917 | 5.3947 | 44.1 | 5.04 | <.0001 |
| Broccoli puree | GameVisit*Group | Week 2 | Control | 23.9738 | 5.3842 | 46.2 | 4.45 | <.0001 |
| Chicken puree | Group | _ | Vegetable | 13.5692 | 3.8674 | 31 | 3.51 | 0.0014 |
| Chicken puree | Group | _ | Control | 12.1353 | 3.8084 | 31 | 3.19 | 0.0033 |
| Chicken puree | GameVisit | Baseline |  | 12.277 | 3.027 | 40.9 | 4.06 | 0.0002 |
| Chicken puree | GameVisit | Week 1 |  | 13.4541 | 3.027 | 40.9 | 4.44 | <.0001 |
| Chicken puree | GameVisit | Week 2 |  | 12.8256 | 3.027 | 40.9 | 4.24 | 0.0001 |
| Chicken puree | GameVisit*Group | Baseline | Vegetable | 14.6062 | 4.1562 | 40.9 | 3.51 | 0.0011 |
| Chicken puree | GameVisit*Group | Baseline | Control | 9.9478 | 4.1365 | 42.5 | 2.4 | 0.0206 |
| Chicken puree | GameVisit*Group | Week 1 | Vegetable | 12.7729 | 4.1562 | 40.9 | 3.07 | 0.0038 |
| Chicken puree | GameVisit*Group | Week 1 | Control | 14.1353 | 4.1365 | 42.5 | 3.42 | 0.0014 |
| Chicken puree | GameVisit*Group | Week 2 | Vegetable | 13.3284 | 4.1562 | 40.9 | 3.21 | 0.0026 |
| Chicken puree | GameVisit*Group | Week 2 | Control | 12.3228 | 4.1365 | 42.5 | 2.98 | 0.0048 |
| Kale chopped | Group | _ | Vegetable | 15.8222 | 3.706 | 31 | 4.27 | 0.0002 |
| Kale chopped | Group | _ | Control | 15.0408 | 3.6494 | 31 | 4.12 | 0.0003 |
| Kale chopped | GameVisit | Baseline |  | 14.4419 | 3.0436 | 48.4 | 4.74 | <.0001 |
| Kale chopped | GameVisit | Week 1 |  | 16.2857 | 3.0436 | 48.4 | 5.35 | <.0001 |
| Kale chopped | GameVisit | Week 2 |  | 15.5669 | 3.0436 | 48.4 | 5.11 | <.0001 |
| Kale chopped | GameVisit*Group | Baseline | Vegetable | 11.9889 | 4.1788 | 48.4 | 2.87 | 0.0061 |
| Kale chopped | GameVisit*Group | Baseline | Control | 16.895 | 4.1847 | 51.1 | 4.04 | 0.0002 |
| Kale chopped | GameVisit*Group | Week 1 | Vegetable | 18.4889 | 4.1788 | 48.4 | 4.42 | <.0001 |
| Kale chopped | GameVisit*Group | Week 1 | Control | 14.0825 | 4.1847 | 51.1 | 3.37 | 0.0015 |
| Kale chopped | GameVisit*Group | Week 2 | Vegetable | 16.9889 | 4.1788 | 48.4 | 4.07 | 0.0002 |
| Kale chopped | GameVisit*Group | Week 2 | Control | 14.145 | 4.1847 | 51.1 | 3.38 | 0.0014 |
| Kale puree | Group | _ | Vegetable | 13.193 | 3.5243 | 31 | 3.74 | 0.0007 |
| Kale puree | Group | _ | Control | 15.2368 | 3.4705 | 31 | 4.39 | 0.0001 |
| Kale puree | GameVisit | Baseline |  | 13.826 | 2.9972 | 54.2 | 4.61 | <.0001 |
| Kale puree | GameVisit | Week 1 |  | 14.5517 | 2.9972 | 54.2 | 4.86 | <.0001 |
| Kale puree | GameVisit | Week 2 |  | 14.267 | 2.9972 | 54.2 | 4.76 | <.0001 |
| Kale puree | GameVisit*Group | Baseline | Vegetable | 11.4152 | 4.1149 | 54.1 | 2.77 | 0.0076 |
| Kale puree | GameVisit*Group | Baseline | Control | 16.2368 | 4.1376 | 57.7 | 3.92 | 0.0002 |
| Kale puree | GameVisit*Group | Week 1 | Vegetable | 13.8041 | 4.1149 | 54.1 | 3.35 | 0.0015 |
| Kale puree | GameVisit*Group | Week 1 | Control | 15.2993 | 4.1376 | 57.7 | 3.7 | 0.0005 |
| Kale puree | GameVisit*Group | Week 2 | Vegetable | 14.3596 | 4.1149 | 54.1 | 3.49 | 0.001 |
| Kale puree | GameVisit*Group | Week 2 | Control | 14.1743 | 4.1376 | 57.7 | 3.43 | 0.0011 |
| Oat puree | Group | _ | Vegetable | 17.4312 | 3.6043 | 31 | 4.84 | <.0001 |
| Oat puree | Group | _ | Control | 15.5857 | 3.5492 | 31 | 4.39 | 0.0001 |
| Oat puree | GameVisit | Baseline |  | 16.3013 | 3.061 | 54 | 5.33 | <.0001 |
| Oat puree | GameVisit | Week 1 |  | 15.1276 | 3.061 | 54 | 4.94 | <.0001 |
| Oat puree | GameVisit | Week 2 |  | 18.0964 | 3.061 | 54 | 5.91 | <.0001 |
| Oat puree | GameVisit*Group | Baseline | Vegetable | 15.5793 | 4.2024 | 53.9 | 3.71 | 0.0005 |
| Oat puree | GameVisit*Group | Baseline | Control | 17.0232 | 4.225 | 57.4 | 4.03 | 0.0002 |
| Oat puree | GameVisit*Group | Week 1 | Vegetable | 16.3571 | 4.2024 | 53.9 | 3.89 | 0.0003 |
| Oat puree | GameVisit*Group | Week 1 | Control | 13.8982 | 4.225 | 57.4 | 3.29 | 0.0017 |
| Oat puree | GameVisit*Group | Week 2 | Vegetable | 20.3571 | 4.2024 | 53.9 | 4.84 | <.0001 |
| Oat puree | GameVisit*Group | Week 2 | Control | 15.8357 | 4.225 | 57.4 | 3.75 | 0.0004 |
| Spinach chopped | Group | _ | Vegetable | 13.7561 | 3.6344 | 31 | 3.78 | 0.0007 |
| Spinach chopped | Group | _ | Control | 12.3944 | 3.5789 | 31 | 3.46 | 0.0016 |
| Spinach chopped | GameVisit | Baseline |  | 11.8738 | 2.939 | 45.9 | 4.04 | 0.0002 |
| Spinach chopped | GameVisit | Week 1 |  | 10.5232 | 2.939 | 45.9 | 3.58 | 0.0008 |
| Spinach chopped | GameVisit | Week 2 |  | 16.8287 | 2.939 | 45.9 | 5.73 | <.0001 |
| Spinach chopped | GameVisit*Group | Baseline | Vegetable | 12.145 | 4.0352 | 45.9 | 3.01 | 0.0042 |
| Spinach chopped | GameVisit*Group | Baseline | Control | 11.6027 | 4.0332 | 48.3 | 2.88 | 0.006 |
| Spinach chopped | GameVisit*Group | Week 1 | Vegetable | 9.7561 | 4.0352 | 45.9 | 2.42 | 0.0196 |
| Spinach chopped | GameVisit*Group | Week 1 | Control | 11.2902 | 4.0332 | 48.3 | 2.8 | 0.0073 |
| Spinach chopped | GameVisit*Group | Week 2 | Vegetable | 19.3672 | 4.0352 | 45.9 | 4.8 | <.0001 |
| Spinach chopped | GameVisit*Group | Week 2 | Control | 14.2902 | 4.0332 | 48.3 | 3.54 | 0.0009 |
| Spinach puree | Group | _ | Vegetable | 11.2976 | 3.2587 | 31 | 3.47 | 0.0016 |
| Spinach puree | Group | _ | Control | 12.0756 | 3.2089 | 31 | 3.76 | 0.0007 |
| Spinach puree | GameVisit | Baseline |  | 10.6264 | 2.6051 | 44.1 | 4.08 | 0.0002 |
| Spinach puree | GameVisit | Week 1 |  | 11.0569 | 2.6051 | 44.1 | 4.24 | 0.0001 |
| Spinach puree | GameVisit | Week 2 |  | 13.3764 | 2.6051 | 44.1 | 5.13 | <.0001 |
| Spinach puree | GameVisit*Group | Baseline | Vegetable | 8.7605 | 3.5769 | 44.1 | 2.45 | 0.0184 |
| Spinach puree | GameVisit*Group | Baseline | Control | 12.4922 | 3.5699 | 46.2 | 3.5 | 0.001 |
| Spinach puree | GameVisit*Group | Week 1 | Vegetable | 10.3716 | 3.5769 | 44.1 | 2.9 | 0.0058 |
| Spinach puree | GameVisit*Group | Week 1 | Control | 11.7422 | 3.5699 | 46.2 | 3.29 | 0.0019 |
| Spinach puree | GameVisit*Group | Week 2 | Vegetable | 14.7605 | 3.5769 | 44.1 | 4.13 | 0.0002 |
| Spinach puree | GameVisit*Group | Week 2 | Control | 11.9922 | 3.5699 | 46.2 | 3.36 | 0.0016 |

## Bitterness Type 3 effects

P-values (ProbF) in this table indicate whether the effect is significant in the overall model.

| **SampleName** | **Effect** | **NumDF** | **DenDF** | **FValue** | **ProbF** |
| --- | --- | --- | --- | --- | --- |
| Asparagus chopped | GameVisit | 2 | 64 | 4.84 | 0.011 |
| Asparagus chopped | Group | 1 | 31 | 3.47 | 0.0721 |
| Asparagus chopped | GameVisit*Group | 2 | 64 | 0.82 | 0.4433 |
| Asparagus chopped | Gender | 1 | 31 | 0.25 | 0.6226 |
| Asparagus puree | GameVisit | 2 | 64 | 0.5 | 0.6073 |
| Asparagus puree | Group | 1 | 31 | 2.35 | 0.1352 |
| Asparagus puree | GameVisit*Group | 2 | 64 | 0.39 | 0.6782 |
| Asparagus puree | Gender | 1 | 31 | 0.08 | 0.7742 |
| Beef puree | GameVisit | 2 | 64 | 1.51 | 0.2295 |
| Beef puree | Group | 1 | 31 | 0 | 0.959 |
| Beef puree | GameVisit*Group | 2 | 64 | 2.61 | 0.0813 |
| Beef puree | Gender | 1 | 31 | 0.15 | 0.7041 |
| Black bean puree | GameVisit | 2 | 64 | 5.14 | 0.0085 |
| Black bean puree | Group | 1 | 31 | 0.06 | 0.8141 |
| Black bean puree | GameVisit*Group | 2 | 64 | 0.2 | 0.8197 |
| Black bean puree | Gender | 1 | 31 | 0.52 | 0.4755 |
| Broccoli chopped | GameVisit | 2 | 64 | 2.07 | 0.1351 |
| Broccoli chopped | Group | 1 | 31 | 0.01 | 0.9149 |
| Broccoli chopped | GameVisit*Group | 2 | 64 | 0.69 | 0.5036 |
| Broccoli chopped | Gender | 1 | 31 | 0.93 | 0.3412 |
| Broccoli puree | GameVisit | 2 | 64 | 0.3 | 0.7392 |
| Broccoli puree | Group | 1 | 31 | 0.22 | 0.6415 |
| Broccoli puree | GameVisit*Group | 2 | 64 | 0.22 | 0.8029 |
| Broccoli puree | Gender | 1 | 31 | 0.1 | 0.7518 |
| Chicken puree | GameVisit | 2 | 64 | 1.04 | 0.361 |
| Chicken puree | Group | 1 | 31 | 0.02 | 0.902 |
| Chicken puree | GameVisit*Group | 2 | 64 | 1.11 | 0.3358 |
| Chicken puree | Gender | 1 | 31 | 0.1 | 0.7598 |
| Kale chopped | GameVisit | 2 | 64 | 0.25 | 0.7806 |
| Kale chopped | Group | 1 | 31 | 0 | 0.9912 |
| Kale chopped | GameVisit*Group | 2 | 64 | 0.41 | 0.6679 |
| Kale chopped | Gender | 1 | 31 | 0 | 0.9459 |
| Kale puree | GameVisit | 2 | 64 | 0.56 | 0.5763 |
| Kale puree | Group | 1 | 31 | 0.14 | 0.7072 |
| Kale puree | GameVisit*Group | 2 | 64 | 0.67 | 0.5136 |
| Kale puree | Gender | 1 | 31 | 0.44 | 0.5101 |
| Oat puree | GameVisit | 2 | 64 | 0.07 | 0.9289 |
| Oat puree | Group | 1 | 31 | 0.06 | 0.8127 |
| Oat puree | GameVisit*Group | 2 | 64 | 0.58 | 0.5623 |
| Oat puree | Gender | 1 | 31 | 1.43 | 0.2408 |
| Spinach chopped | GameVisit | 2 | 64 | 8.76 | 0.0004 |
| Spinach chopped | Group | 1 | 31 | 0.39 | 0.5349 |
| Spinach chopped | GameVisit*Group | 2 | 64 | 0.39 | 0.6799 |
| Spinach chopped | Gender | 1 | 31 | 0.23 | 0.633 |
| Spinach puree | GameVisit | 2 | 64 | 0.96 | 0.3869 |
| Spinach puree | Group | 1 | 31 | 0.04 | 0.8495 |
| Spinach puree | GameVisit*Group | 2 | 64 | 0.59 | 0.556 |
| Spinach puree | Gender | 1 | 31 | 0.03 | 0.8712 |

## Bitterness Contrasts

P-values in this table indicate whether the estimate of the difference between the two means is significantly different from 0.

| **SampleName** | **Effect** | **Label** | **Estimate** | **StdErr** | **DF** | **tValue** | **Probt** |
| --- | --- | --- | --- | --- | --- | --- | --- |
| Asparagus chopped | GameVisit*Group | Control, Week 2-Baseline | 8.5 | 5.2271 | 64 | 1.63 | 0.1088 |
| Asparagus chopped | GameVisit*Group | Control, Week 1-Baseline | 13.3125 | 5.2271 | 64 | 2.55 | 0.0133 |
| Asparagus chopped | GameVisit*Group | Veg, Week 2-Baseline | -0.7222 | 4.9282 | 64 | -0.15 | 0.8839 |
| Asparagus chopped | GameVisit*Group | Veg, Week 1-Baseline | 8.7222 | 4.9282 | 64 | 1.77 | 0.0815 |
| Asparagus chopped | GameVisit*Group | Veg - Control, Baseline | 17.5799 | 8.1099 | 53.59 | 2.17 | 0.0346 |
| Asparagus chopped | GameVisit*Group | Veg - Control, Week 1 | 12.9896 | 8.1099 | 53.59 | 1.6 | 0.1151 |
| Asparagus chopped | GameVisit*Group | Veg - Control, Week 2 | 8.3577 | 8.1099 | 53.59 | 1.03 | 0.3074 |
| Asparagus puree | GameVisit*Group | Control, Week 2-Baseline | 5.1875 | 4.8232 | 64 | 1.08 | 0.2862 |
| Asparagus puree | GameVisit*Group | Control, Week 1-Baseline | 4.8125 | 4.8232 | 64 | 1 | 0.3221 |
| Asparagus puree | GameVisit*Group | Veg, Week 2-Baseline | -0.6667 | 4.5474 | 64 | -0.15 | 0.8839 |
| Asparagus puree | GameVisit*Group | Veg, Week 1-Baseline | 1.6667 | 4.5474 | 64 | 0.37 | 0.7152 |
| Asparagus puree | GameVisit*Group | Veg - Control, Baseline | 12.8151 | 7.4554 | 53.82 | 1.72 | 0.0914 |
| Asparagus puree | GameVisit*Group | Veg - Control, Week 1 | 9.6693 | 7.4554 | 53.82 | 1.3 | 0.2002 |
| Asparagus puree | GameVisit*Group | Veg - Control, Week 2 | 6.9609 | 7.4554 | 53.82 | 0.93 | 0.3546 |
| Beef puree | GameVisit*Group | Control, Week 2-Baseline | -6.5625 | 4.1644 | 64 | -1.58 | 0.12 |
| Beef puree | GameVisit*Group | Control, Week 1-Baseline | 4.6875 | 4.1644 | 64 | 1.13 | 0.2645 |
| Beef puree | GameVisit*Group | Veg, Week 2-Baseline | 2.6667 | 3.9263 | 64 | 0.68 | 0.4995 |
| Beef puree | GameVisit*Group | Veg, Week 1-Baseline | 1.2778 | 3.9263 | 64 | 0.33 | 0.7459 |
| Beef puree | GameVisit*Group | Veg - Control, Baseline | -1.541 | 8.3742 | 42.78 | -0.18 | 0.8549 |
| Beef puree | GameVisit*Group | Veg - Control, Week 1 | -4.9508 | 8.3742 | 42.78 | -0.59 | 0.5575 |
| Beef puree | GameVisit*Group | Veg - Control, Week 2 | 7.6881 | 8.3742 | 42.78 | 0.92 | 0.3637 |
| Black bean puree | GameVisit*Group | Control, Week 2-Baseline | 0.625 | 4.5148 | 64 | 0.14 | 0.8903 |
| Black bean puree | GameVisit*Group | Control, Week 1-Baseline | 10.5625 | 4.5148 | 64 | 2.34 | 0.0224 |
| Black bean puree | GameVisit*Group | Veg, Week 2-Baseline | -0.3889 | 4.2566 | 64 | -0.09 | 0.9275 |
| Black bean puree | GameVisit*Group | Veg, Week 1-Baseline | 6.7778 | 4.2566 | 64 | 1.59 | 0.1162 |
| Black bean puree | GameVisit*Group | Veg - Control, Baseline | 0.008444 | 7.6055 | 49.26 | 0 | 0.9991 |
| Black bean puree | GameVisit*Group | Veg - Control, Week 1 | -3.7763 | 7.6055 | 49.26 | -0.5 | 0.6217 |
| Black bean puree | GameVisit*Group | Veg - Control, Week 2 | -1.0054 | 7.6055 | 49.26 | -0.13 | 0.8954 |
| Broccoli chopped | GameVisit*Group | Control, Week 2-Baseline | -1.125 | 3.2751 | 64 | -0.34 | 0.7323 |
| Broccoli chopped | GameVisit*Group | Control, Week 1-Baseline | 1.625 | 3.2751 | 64 | 0.5 | 0.6215 |
| Broccoli chopped | GameVisit*Group | Veg, Week 2-Baseline | 2.3333 | 3.0878 | 64 | 0.76 | 0.4526 |
| Broccoli chopped | GameVisit*Group | Veg, Week 1-Baseline | 6.8333 | 3.0878 | 64 | 2.21 | 0.0305 |
| Broccoli chopped | GameVisit*Group | Veg - Control, Baseline | -3.5798 | 6.9232 | 41.46 | -0.52 | 0.6078 |
| Broccoli chopped | GameVisit*Group | Veg - Control, Week 1 | 1.6285 | 6.9232 | 41.46 | 0.24 | 0.8152 |
| Broccoli chopped | GameVisit*Group | Veg - Control, Week 2 | -0.1215 | 6.9232 | 41.46 | -0.02 | 0.9861 |
| Broccoli puree | GameVisit*Group | Control, Week 2-Baseline | -3.5625 | 4.3099 | 64 | -0.83 | 0.4115 |
| Broccoli puree | GameVisit*Group | Control, Week 1-Baseline | -1.0625 | 4.3099 | 64 | -0.25 | 0.8061 |
| Broccoli puree | GameVisit*Group | Veg, Week 2-Baseline | -0.8889 | 4.0634 | 64 | -0.22 | 0.8275 |
| Broccoli puree | GameVisit*Group | Veg, Week 1-Baseline | -2.2222 | 4.0634 | 64 | -0.55 | 0.5864 |
| Broccoli puree | GameVisit*Group | Veg - Control, Baseline | 2.3307 | 6.9329 | 51.56 | 0.34 | 0.7381 |
| Broccoli puree | GameVisit*Group | Veg - Control, Week 1 | 1.171 | 6.9329 | 51.56 | 0.17 | 0.8665 |
| Broccoli puree | GameVisit*Group | Veg - Control, Week 2 | 5.0043 | 6.9329 | 51.56 | 0.72 | 0.4737 |
| Chicken puree | GameVisit*Group | Control, Week 2-Baseline | -0.1875 | 3.6864 | 64 | -0.05 | 0.9596 |
| Chicken puree | GameVisit*Group | Control, Week 1-Baseline | 4.5625 | 3.6864 | 64 | 1.24 | 0.2204 |
| Chicken puree | GameVisit*Group | Veg, Week 2-Baseline | 5.1111 | 3.4756 | 64 | 1.47 | 0.1463 |
| Chicken puree | GameVisit*Group | Veg, Week 1-Baseline | 2.5556 | 3.4756 | 64 | 0.74 | 0.4649 |
| Chicken puree | GameVisit*Group | Veg - Control, Baseline | 0.03166 | 9.5489 | 37.56 | 0 | 0.9974 |
| Chicken puree | GameVisit*Group | Veg - Control, Week 1 | -1.9753 | 9.5489 | 37.56 | -0.21 | 0.8372 |
| Chicken puree | GameVisit*Group | Veg - Control, Week 2 | 5.3303 | 9.5489 | 37.56 | 0.56 | 0.58 |
| Kale chopped | GameVisit*Group | Control, Week 2-Baseline | 4.125 | 5.2662 | 64 | 0.78 | 0.4363 |
| Kale chopped | GameVisit*Group | Control, Week 1-Baseline | 5.4375 | 5.2662 | 64 | 1.03 | 0.3057 |
| Kale chopped | GameVisit*Group | Veg, Week 2-Baseline | 0.3333 | 4.9651 | 64 | 0.07 | 0.9467 |
| Kale chopped | GameVisit*Group | Veg, Week 1-Baseline | -1.0556 | 4.9651 | 64 | -0.21 | 0.8323 |
| Kale chopped | GameVisit*Group | Veg - Control, Baseline | 3.3459 | 8.4882 | 51.45 | 0.39 | 0.6951 |
| Kale chopped | GameVisit*Group | Veg - Control, Week 1 | -3.1471 | 8.4882 | 51.45 | -0.37 | 0.7123 |
| Kale chopped | GameVisit*Group | Veg - Control, Week 2 | -0.4457 | 8.4882 | 51.45 | -0.05 | 0.9583 |
| Kale puree | GameVisit*Group | Control, Week 2-Baseline | 7.3125 | 6.8429 | 64 | 1.07 | 0.2893 |
| Kale puree | GameVisit*Group | Control, Week 1-Baseline | 5.625 | 6.8429 | 64 | 0.82 | 0.4141 |
| Kale puree | GameVisit*Group | Veg, Week 2-Baseline | 1.4444 | 6.4516 | 64 | 0.22 | 0.8236 |
| Kale puree | GameVisit*Group | Veg, Week 1-Baseline | -5.2778 | 6.4516 | 64 | -0.82 | 0.4164 |
| Kale puree | GameVisit*Group | Veg - Control, Baseline | 3.288 | 8.1459 | 76.65 | 0.4 | 0.6876 |
| Kale puree | GameVisit*Group | Veg - Control, Week 1 | -7.6148 | 8.1459 | 76.65 | -0.93 | 0.3528 |
| Kale puree | GameVisit*Group | Veg - Control, Week 2 | -2.5801 | 8.1459 | 76.65 | -0.32 | 0.7523 |
| Oat puree | GameVisit*Group | Control, Week 2-Baseline | 2.8125 | 3.5852 | 64 | 0.78 | 0.4357 |
| Oat puree | GameVisit*Group | Control, Week 1-Baseline | 3.3125 | 3.5852 | 64 | 0.92 | 0.359 |
| Oat puree | GameVisit*Group | Veg, Week 2-Baseline | -1.6111 | 3.3801 | 64 | -0.48 | 0.6352 |
| Oat puree | GameVisit*Group | Veg, Week 1-Baseline | -1.4444 | 3.3801 | 64 | -0.43 | 0.6706 |
| Oat puree | GameVisit*Group | Veg - Control, Baseline | 1.3152 | 7.8347 | 40.68 | 0.17 | 0.8675 |
| Oat puree | GameVisit*Group | Veg - Control, Week 1 | -3.4417 | 7.8347 | 40.68 | -0.44 | 0.6628 |
| Oat puree | GameVisit*Group | Veg - Control, Week 2 | -3.1084 | 7.8347 | 40.68 | -0.4 | 0.6936 |
| Spinach chopped | GameVisit*Group | Control, Week 2-Baseline | -5.5 | 4.5623 | 64 | -1.21 | 0.2324 |
| Spinach chopped | GameVisit*Group | Control, Week 1-Baseline | 6.3125 | 4.5623 | 64 | 1.38 | 0.1713 |
| Spinach chopped | GameVisit*Group | Veg, Week 2-Baseline | -11 | 4.3014 | 64 | -2.56 | 0.0129 |
| Spinach chopped | GameVisit*Group | Veg, Week 1-Baseline | 3.1111 | 4.3014 | 64 | 0.72 | 0.4721 |
| Spinach chopped | GameVisit*Group | Veg - Control, Baseline | -0.9308 | 7.0979 | 53.43 | -0.13 | 0.8962 |
| Spinach chopped | GameVisit*Group | Veg - Control, Week 1 | -4.1322 | 7.0979 | 53.43 | -0.58 | 0.5629 |
| Spinach chopped | GameVisit*Group | Veg - Control, Week 2 | -6.4308 | 7.0979 | 53.43 | -0.91 | 0.369 |
| Spinach puree | GameVisit*Group | Control, Week 2-Baseline | 1.4375 | 5.6712 | 64 | 0.25 | 0.8007 |
| Spinach puree | GameVisit*Group | Control, Week 1-Baseline | 8.9375 | 5.6712 | 64 | 1.58 | 0.12 |
| Spinach puree | GameVisit*Group | Veg, Week 2-Baseline | -0.8333 | 5.3468 | 64 | -0.16 | 0.8766 |
| Spinach puree | GameVisit*Group | Veg, Week 1-Baseline | 0.7222 | 5.3468 | 64 | 0.14 | 0.893 |
| Spinach puree | GameVisit*Group | Veg - Control, Baseline | 1.9866 | 9.0786 | 51.82 | 0.22 | 0.8276 |
| Spinach puree | GameVisit*Group | Veg - Control, Week 1 | -6.2287 | 9.0786 | 51.82 | -0.69 | 0.4957 |
| Spinach puree | GameVisit*Group | Veg - Control, Week 2 | -0.2842 | 9.0786 | 51.82 | -0.03 | 0.9751 |

## Bitterness Least Square means

P-values in this table indicate whether the estimate is significantly different from 0.

| **SampleName** | **Effect** | **GameVisit** | **Group** | **Estimate** | **StdErr** | **DF** | **tValue** | **Probt** |
| --- | --- | --- | --- | --- | --- | --- | --- | --- |
| Asparagus chopped | Group | _ | Vegetable | 40.221 | 5.1683 | 31 | 7.78 | <.0001 |
| Asparagus chopped | Group | _ | Control | 27.2453 | 5.0894 | 31 | 5.35 | <.0001 |
| Asparagus chopped | GameVisit | Baseline |  | 28.7644 | 4.2972 | 50.4 | 6.69 | <.0001 |
| Asparagus chopped | GameVisit | Week 1 |  | 39.7817 | 4.2972 | 50.4 | 9.26 | <.0001 |
| Asparagus chopped | GameVisit | Week 2 |  | 32.6533 | 4.2972 | 50.4 | 7.6 | <.0001 |
| Asparagus chopped | GameVisit*Group | Baseline | Vegetable | 37.5543 | 5.8998 | 50.4 | 6.37 | <.0001 |
| Asparagus chopped | GameVisit*Group | Baseline | Control | 19.9744 | 5.9169 | 53.4 | 3.38 | 0.0014 |
| Asparagus chopped | GameVisit*Group | Week 1 | Vegetable | 46.2765 | 5.8998 | 50.4 | 7.84 | <.0001 |
| Asparagus chopped | GameVisit*Group | Week 1 | Control | 33.2869 | 5.9169 | 53.4 | 5.63 | <.0001 |
| Asparagus chopped | GameVisit*Group | Week 2 | Vegetable | 36.8321 | 5.8998 | 50.4 | 6.24 | <.0001 |
| Asparagus chopped | GameVisit*Group | Week 2 | Control | 28.4744 | 5.9169 | 53.4 | 4.81 | <.0001 |
| Asparagus puree | Group | _ | Vegetable | 35.2699 | 4.7449 | 31 | 7.43 | <.0001 |
| Asparagus puree | Group | _ | Control | 25.4548 | 4.6725 | 31 | 5.45 | <.0001 |
| Asparagus puree | GameVisit | Baseline |  | 28.529 | 3.9498 | 50.6 | 7.22 | <.0001 |
| Asparagus puree | GameVisit | Week 1 |  | 31.7686 | 3.9498 | 50.6 | 8.04 | <.0001 |
| Asparagus puree | GameVisit | Week 2 |  | 30.7894 | 3.9498 | 50.6 | 7.8 | <.0001 |
| Asparagus puree | GameVisit*Group | Baseline | Vegetable | 34.9366 | 5.4228 | 50.6 | 6.44 | <.0001 |
| Asparagus puree | GameVisit*Group | Baseline | Control | 22.1215 | 5.4394 | 53.7 | 4.07 | 0.0002 |
| Asparagus puree | GameVisit*Group | Week 1 | Vegetable | 36.6032 | 5.4228 | 50.6 | 6.75 | <.0001 |
| Asparagus puree | GameVisit*Group | Week 1 | Control | 26.934 | 5.4394 | 53.7 | 4.95 | <.0001 |
| Asparagus puree | GameVisit*Group | Week 2 | Vegetable | 34.2699 | 5.4228 | 50.6 | 6.32 | <.0001 |
| Asparagus puree | GameVisit*Group | Week 2 | Control | 27.309 | 5.4394 | 53.7 | 5.02 | <.0001 |
| Beef puree | Group | _ | Vegetable | 25.4902 | 5.7065 | 31 | 4.47 | <.0001 |
| Beef puree | Group | _ | Control | 25.0914 | 5.6194 | 31 | 4.47 | <.0001 |
| Beef puree | GameVisit | Baseline |  | 24.9459 | 4.472 | 41.1 | 5.58 | <.0001 |
| Beef puree | GameVisit | Week 1 |  | 27.9286 | 4.472 | 41.1 | 6.25 | <.0001 |
| Beef puree | GameVisit | Week 2 |  | 22.998 | 4.472 | 41.1 | 5.14 | <.0001 |
| Beef puree | GameVisit*Group | Baseline | Vegetable | 24.1754 | 6.1402 | 41.1 | 3.94 | 0.0003 |
| Beef puree | GameVisit*Group | Baseline | Control | 25.7164 | 6.1121 | 42.7 | 4.21 | 0.0001 |
| Beef puree | GameVisit*Group | Week 1 | Vegetable | 25.4532 | 6.1402 | 41.1 | 4.15 | 0.0002 |
| Beef puree | GameVisit*Group | Week 1 | Control | 30.4039 | 6.1121 | 42.7 | 4.97 | <.0001 |
| Beef puree | GameVisit*Group | Week 2 | Vegetable | 26.8421 | 6.1402 | 41.1 | 4.37 | <.0001 |
| Beef puree | GameVisit*Group | Week 2 | Control | 19.1539 | 6.1121 | 42.7 | 3.13 | 0.0031 |
| Black bean puree | Group | _ | Vegetable | 21.0995 | 4.9754 | 31 | 4.24 | 0.0002 |
| Black bean puree | Group | _ | Control | 22.6906 | 4.8995 | 31 | 4.63 | <.0001 |
| Black bean puree | GameVisit | Baseline |  | 18.9657 | 4.0418 | 46.7 | 4.69 | <.0001 |
| Black bean puree | GameVisit | Week 1 |  | 27.6358 | 4.0418 | 46.7 | 6.84 | <.0001 |
| Black bean puree | GameVisit | Week 2 |  | 19.0837 | 4.0418 | 46.7 | 4.72 | <.0001 |
| Black bean puree | GameVisit*Group | Baseline | Vegetable | 18.9699 | 5.5493 | 46.6 | 3.42 | 0.0013 |
| Black bean puree | GameVisit*Group | Baseline | Control | 18.9615 | 5.5497 | 49.1 | 3.42 | 0.0013 |
| Black bean puree | GameVisit*Group | Week 1 | Vegetable | 25.7477 | 5.5493 | 46.6 | 4.64 | <.0001 |
| Black bean puree | GameVisit*Group | Week 1 | Control | 29.524 | 5.5497 | 49.1 | 5.32 | <.0001 |
| Black bean puree | GameVisit*Group | Week 2 | Vegetable | 18.581 | 5.5493 | 46.6 | 3.35 | 0.0016 |
| Black bean puree | GameVisit*Group | Week 2 | Control | 19.5865 | 5.5497 | 49.1 | 3.53 | 0.0009 |
| Broccoli chopped | Group | _ | Vegetable | 25.8877 | 4.759 | 31 | 5.44 | <.0001 |
| Broccoli chopped | Group | _ | Control | 26.5786 | 4.6863 | 31 | 5.67 | <.0001 |
| Broccoli chopped | GameVisit | Baseline |  | 24.622 | 3.7011 | 39.9 | 6.65 | <.0001 |
| Broccoli chopped | GameVisit | Week 1 |  | 28.8512 | 3.7011 | 39.9 | 7.8 | <.0001 |
| Broccoli chopped | GameVisit | Week 2 |  | 25.2262 | 3.7011 | 39.9 | 6.82 | <.0001 |
| Broccoli chopped | GameVisit*Group | Baseline | Vegetable | 22.8321 | 5.0819 | 39.9 | 4.49 | <.0001 |
| Broccoli chopped | GameVisit*Group | Baseline | Control | 26.4119 | 5.0534 | 41.4 | 5.23 | <.0001 |
| Broccoli chopped | GameVisit*Group | Week 1 | Vegetable | 29.6654 | 5.0819 | 39.9 | 5.84 | <.0001 |
| Broccoli chopped | GameVisit*Group | Week 1 | Control | 28.0369 | 5.0534 | 41.4 | 5.55 | <.0001 |
| Broccoli chopped | GameVisit*Group | Week 2 | Vegetable | 25.1654 | 5.0819 | 39.9 | 4.95 | <.0001 |
| Broccoli chopped | GameVisit*Group | Week 2 | Control | 25.2869 | 5.0534 | 41.4 | 5 | <.0001 |
| Broccoli puree | Group | _ | Vegetable | 22.1552 | 4.4725 | 31 | 4.95 | <.0001 |
| Broccoli puree | Group | _ | Control | 19.3198 | 4.4042 | 31 | 4.39 | 0.0001 |
| Broccoli puree | GameVisit | Baseline |  | 22.0269 | 3.6785 | 48.7 | 5.99 | <.0001 |
| Broccoli puree | GameVisit | Week 1 |  | 20.3845 | 3.6785 | 48.7 | 5.54 | <.0001 |
| Broccoli puree | GameVisit | Week 2 |  | 19.8012 | 3.6785 | 48.7 | 5.38 | <.0001 |
| Broccoli puree | GameVisit*Group | Baseline | Vegetable | 23.1922 | 5.0504 | 48.6 | 4.59 | <.0001 |
| Broccoli puree | GameVisit*Group | Baseline | Control | 20.8615 | 5.0585 | 51.4 | 4.12 | 0.0001 |
| Broccoli puree | GameVisit*Group | Week 1 | Vegetable | 20.97 | 5.0504 | 48.6 | 4.15 | 0.0001 |
| Broccoli puree | GameVisit*Group | Week 1 | Control | 19.799 | 5.0585 | 51.4 | 3.91 | 0.0003 |
| Broccoli puree | GameVisit*Group | Week 2 | Vegetable | 22.3033 | 5.0504 | 48.6 | 4.42 | <.0001 |
| Broccoli puree | GameVisit*Group | Week 2 | Control | 17.299 | 5.0585 | 51.4 | 3.42 | 0.0012 |
| Chicken puree | Group | _ | Vegetable | 28.9086 | 6.7412 | 31 | 4.29 | 0.0002 |
| Chicken puree | Group | _ | Control | 27.7797 | 6.6383 | 31 | 4.18 | 0.0002 |
| Chicken puree | GameVisit | Baseline |  | 26.3372 | 5.1223 | 36.6 | 5.14 | <.0001 |
| Chicken puree | GameVisit | Week 1 |  | 29.8962 | 5.1223 | 36.6 | 5.84 | <.0001 |
| Chicken puree | GameVisit | Week 2 |  | 28.799 | 5.1223 | 36.6 | 5.62 | <.0001 |
| Chicken puree | GameVisit*Group | Baseline | Vegetable | 26.353 | 7.0335 | 36.6 | 3.75 | 0.0006 |
| Chicken puree | GameVisit*Group | Baseline | Control | 26.3214 | 6.9711 | 37.5 | 3.78 | 0.0006 |
| Chicken puree | GameVisit*Group | Week 1 | Vegetable | 28.9086 | 7.0335 | 36.6 | 4.11 | 0.0002 |
| Chicken puree | GameVisit*Group | Week 1 | Control | 30.8839 | 6.9711 | 37.5 | 4.43 | <.0001 |
| Chicken puree | GameVisit*Group | Week 2 | Vegetable | 31.4641 | 7.0335 | 36.6 | 4.47 | <.0001 |
| Chicken puree | GameVisit*Group | Week 2 | Control | 26.1339 | 6.9711 | 37.5 | 3.75 | 0.0006 |
| Kale chopped | Group | _ | Vegetable | 42.1609 | 5.4793 | 31 | 7.69 | <.0001 |
| Kale chopped | Group | _ | Control | 42.2433 | 5.3957 | 31 | 7.83 | <.0001 |
| Kale chopped | GameVisit | Baseline |  | 40.7287 | 4.5041 | 48.6 | 9.04 | <.0001 |
| Kale chopped | GameVisit | Week 1 |  | 42.9197 | 4.5041 | 48.6 | 9.53 | <.0001 |
| Kale chopped | GameVisit | Week 2 |  | 42.9579 | 4.5041 | 48.6 | 9.54 | <.0001 |
| Kale chopped | GameVisit*Group | Baseline | Vegetable | 42.4017 | 6.1839 | 48.5 | 6.86 | <.0001 |
| Kale chopped | GameVisit*Group | Baseline | Control | 39.0558 | 6.1934 | 51.3 | 6.31 | <.0001 |
| Kale chopped | GameVisit*Group | Week 1 | Vegetable | 41.3461 | 6.1839 | 48.5 | 6.69 | <.0001 |
| Kale chopped | GameVisit*Group | Week 1 | Control | 44.4933 | 6.1934 | 51.3 | 7.18 | <.0001 |
| Kale chopped | GameVisit*Group | Week 2 | Vegetable | 42.735 | 6.1839 | 48.5 | 6.91 | <.0001 |
| Kale chopped | GameVisit*Group | Week 2 | Control | 43.1808 | 6.1934 | 51.3 | 6.97 | <.0001 |
| Kale puree | Group | _ | Vegetable | 36.7686 | 4.5034 | 31 | 8.16 | <.0001 |
| Kale puree | Group | _ | Control | 39.0709 | 4.4346 | 31 | 8.81 | <.0001 |
| Kale puree | GameVisit | Baseline |  | 36.4023 | 4.2574 | 71.7 | 8.55 | <.0001 |
| Kale puree | GameVisit | Week 1 |  | 36.576 | 4.2574 | 71.7 | 8.59 | <.0001 |
| Kale puree | GameVisit | Week 2 |  | 40.7808 | 4.2574 | 71.7 | 9.58 | <.0001 |
| Kale puree | GameVisit*Group | Baseline | Vegetable | 38.0463 | 5.8442 | 71.7 | 6.51 | <.0001 |
| Kale puree | GameVisit*Group | Baseline | Control | 34.7584 | 5.9392 | 76.4 | 5.85 | <.0001 |
| Kale puree | GameVisit*Group | Week 1 | Vegetable | 32.7686 | 5.8442 | 71.7 | 5.61 | <.0001 |
| Kale puree | GameVisit*Group | Week 1 | Control | 40.3834 | 5.9392 | 76.4 | 6.8 | <.0001 |
| Kale puree | GameVisit*Group | Week 2 | Vegetable | 39.4908 | 5.8442 | 71.7 | 6.76 | <.0001 |
| Kale puree | GameVisit*Group | Week 2 | Control | 42.0709 | 5.9392 | 76.4 | 7.08 | <.0001 |
| Oat puree | Group | _ | Vegetable | 24.8047 | 5.4138 | 31 | 4.58 | <.0001 |
| Oat puree | Group | _ | Control | 26.5496 | 5.3312 | 31 | 4.98 | <.0001 |
| Oat puree | GameVisit | Baseline |  | 25.1656 | 4.1912 | 39.3 | 6 | <.0001 |
| Oat puree | GameVisit | Week 1 |  | 26.0996 | 4.1912 | 39.3 | 6.23 | <.0001 |
| Oat puree | GameVisit | Week 2 |  | 25.7662 | 4.1912 | 39.3 | 6.15 | <.0001 |
| Oat puree | GameVisit*Group | Baseline | Vegetable | 25.8232 | 5.7548 | 39.3 | 4.49 | <.0001 |
| Oat puree | GameVisit*Group | Baseline | Control | 24.5079 | 5.7189 | 40.6 | 4.29 | 0.0001 |
| Oat puree | GameVisit*Group | Week 1 | Vegetable | 24.3787 | 5.7548 | 39.3 | 4.24 | 0.0001 |
| Oat puree | GameVisit*Group | Week 1 | Control | 27.8204 | 5.7189 | 40.6 | 4.86 | <.0001 |
| Oat puree | GameVisit*Group | Week 2 | Vegetable | 24.2121 | 5.7548 | 39.3 | 4.21 | 0.0001 |
| Oat puree | GameVisit*Group | Week 2 | Control | 27.3204 | 5.7189 | 40.6 | 4.78 | <.0001 |
| Spinach chopped | Group | _ | Vegetable | 30.6552 | 4.5278 | 31 | 6.77 | <.0001 |
| Spinach chopped | Group | _ | Control | 34.4865 | 4.4587 | 31 | 7.73 | <.0001 |
| Spinach chopped | GameVisit | Baseline |  | 33.7502 | 3.7614 | 50.3 | 8.97 | <.0001 |
| Spinach chopped | GameVisit | Week 1 |  | 38.4621 | 3.7614 | 50.3 | 10.23 | <.0001 |
| Spinach chopped | GameVisit | Week 2 |  | 25.5002 | 3.7614 | 50.3 | 6.78 | <.0001 |
| Spinach chopped | GameVisit*Group | Baseline | Vegetable | 33.2848 | 5.1642 | 50.3 | 6.45 | <.0001 |
| Spinach chopped | GameVisit*Group | Baseline | Control | 34.2157 | 5.1786 | 53.3 | 6.61 | <.0001 |
| Spinach chopped | GameVisit*Group | Week 1 | Vegetable | 36.3959 | 5.1642 | 50.3 | 7.05 | <.0001 |
| Spinach chopped | GameVisit*Group | Week 1 | Control | 40.5282 | 5.1786 | 53.3 | 7.83 | <.0001 |
| Spinach chopped | GameVisit*Group | Week 2 | Vegetable | 22.2848 | 5.1642 | 50.3 | 4.32 | <.0001 |
| Spinach chopped | GameVisit*Group | Week 2 | Control | 28.7157 | 5.1786 | 53.3 | 5.55 | <.0001 |
| Spinach puree | Group | _ | Vegetable | 34.1886 | 5.8475 | 31 | 5.85 | <.0001 |
| Spinach puree | Group | _ | Control | 35.6974 | 5.7582 | 31 | 6.2 | <.0001 |
| Spinach puree | GameVisit | Baseline |  | 33.2323 | 4.8162 | 48.9 | 6.9 | <.0001 |
| Spinach puree | GameVisit | Week 1 |  | 38.0622 | 4.8162 | 48.9 | 7.9 | <.0001 |
| Spinach puree | GameVisit | Week 2 |  | 33.5344 | 4.8162 | 48.9 | 6.96 | <.0001 |
| Spinach puree | GameVisit*Group | Baseline | Vegetable | 34.2256 | 6.6124 | 48.8 | 5.18 | <.0001 |
| Spinach puree | GameVisit*Group | Baseline | Control | 32.239 | 6.6241 | 51.7 | 4.87 | <.0001 |
| Spinach puree | GameVisit*Group | Week 1 | Vegetable | 34.9479 | 6.6124 | 48.8 | 5.29 | <.0001 |
| Spinach puree | GameVisit*Group | Week 1 | Control | 41.1765 | 6.6241 | 51.7 | 6.22 | <.0001 |
| Spinach puree | GameVisit*Group | Week 2 | Vegetable | 33.3923 | 6.6124 | 48.8 | 5.05 | <.0001 |
| Spinach puree | GameVisit*Group | Week 2 | Control | 33.6765 | 6.6241 | 51.7 | 5.08 | <.0001 |

## Secondary analysis outputs

Differences between vegetables, and from 0

### Type 3 fixed effects

| **GameVisit** | **Effect** | **NumDF** | **DenDF** | **FValue** | **ProbF** |
| --- | --- | --- | --- | --- | --- |
| Baseline | SampleName | 11 | 352 | 4.03 | <.0001 |
| Baseline | Group | 1 | 31 | 0.75 | 0.3935 |
| Baseline | Group*SampleName | 11 | 352 | 0.96 | 0.4822 |
| Baseline | Gender | 1 | 31 | 0.2 | 0.6618 |
| Week 1 | SampleName | 11 | 352 | 4.45 | <.0001 |
| Week 1 | Group | 1 | 31 | 0.28 | 0.5997 |
| Week 1 | Group*SampleName | 11 | 352 | 1.12 | 0.3466 |
| Week 1 | Gender | 1 | 31 | 0.07 | 0.7954 |
| Week 2 | SampleName | 11 | 352 | 3.89 | <.0001 |
| Week 2 | Group | 1 | 31 | 0.01 | 0.9336 |
| Week 2 | Group*SampleName | 11 | 352 | 1.49 | 0.1329 |
| Week 2 | Gender | 1 | 31 | 0.14 | 0.7103 |

### Least Squared Means

P-values in this table indicate whether the estimate is significantly different from 0.

| **GameVisit** | **Effect** | **Group** | **SampleName** | **Estimate** | **StdErr** | **DF** | **tValue** | **Probt** |
| --- | --- | --- | --- | --- | --- | --- | --- | --- |
| Baseline | Group | Vegetable |  | -8.1429 | 6.0831 | 31 | -1.34 | 0.1904 |
| Baseline | Group | Control |  | -1.0455 | 5.9902 | 31 | -0.17 | 0.8626 |
| Baseline | SampleName | | Asparagus chopped | 7.091 | 7.2788 | 180 | 0.97 | 0.3313 |
| Baseline | SampleName | | Asparagus puree | 4.0771 | 7.2788 | 180 | 0.56 | 0.5761 |
| Baseline | SampleName | | Beef puree | -14.9958 | 7.2788 | 180 | -2.06 | 0.0408 |
| Baseline | SampleName | | Black bean puree | -5.1347 | 7.2788 | 180 | -0.71 | 0.4814 |
| Baseline | SampleName | | Broccoli chopped | 14.9521 | 7.2788 | 180 | 2.05 | 0.0414 |
| Baseline | SampleName | | Broccoli puree | 13.9556 | 7.2788 | 180 | 1.92 | 0.0568 |
| Baseline | SampleName | | Chicken puree | -16.7007 | 7.2788 | 180 | -2.29 | 0.0229 |
| Baseline | SampleName | | Kale chopped | -22.3951 | 7.2788 | 180 | -3.08 | 0.0024 |
| Baseline | SampleName | | Kale puree | -11.2319 | 7.2788 | 180 | -1.54 | 0.1246 |
| Baseline | SampleName | | Oat puree | -11.8986 | 7.2788 | 180 | -1.63 | 0.1039 |
| Baseline | SampleName | | Spinach chopped | -5.784 | 7.2788 | 180 | -0.79 | 0.4279 |
| Baseline | SampleName | | Spinach puree | -7.0653 | 7.2788 | 180 | -0.97 | 0.333 |
| Baseline | Group*SampleName | Vegetable | Asparagus chopped | -3.5642 | 9.9897 | 180 | -0.36 | 0.7217 |
| Baseline | Group*SampleName | Vegetable | Asparagus puree | -3.842 | 9.9897 | 180 | -0.38 | 0.701 |
| Baseline | Group*SampleName | Vegetable | Beef puree | -22.6753 | 9.9897 | 180 | -2.27 | 0.0244 |
| Baseline | Group*SampleName | Vegetable | Black bean puree | -10.4531 | 9.9897 | 180 | -1.05 | 0.2968 |
| Baseline | Group*SampleName | Vegetable | Broccoli chopped | 14.158 | 9.9897 | 180 | 1.42 | 0.1581 |
| Baseline | Group*SampleName | Vegetable | Broccoli puree | 18.1025 | 9.9897 | 180 | 1.81 | 0.0716 |
| Baseline | Group*SampleName | Vegetable | Chicken puree | -25.8975 | 9.9897 | 180 | -2.59 | 0.0103 |
| Baseline | Group*SampleName | Vegetable | Kale chopped | -30.7864 | 9.9897 | 180 | -3.08 | 0.0024 |
| Baseline | Group*SampleName | Vegetable | Kale puree | -16.8975 | 9.9897 | 180 | -1.69 | 0.0925 |
| Baseline | Group*SampleName | Vegetable | Oat puree | -3.7308 | 9.9897 | 180 | -0.37 | 0.7092 |
| Baseline | Group*SampleName | Vegetable | Spinach chopped | -5.0642 | 9.9897 | 180 | -0.51 | 0.6128 |
| Baseline | Group*SampleName | Vegetable | Spinach puree | -7.0642 | 9.9897 | 180 | -0.71 | 0.4804 |
| Baseline | Group*SampleName | Control | Asparagus chopped | 17.7461 | 10.3209 | 204 | 1.72 | 0.0871 |
| Baseline | Group*SampleName | Control | Asparagus puree | 11.9961 | 10.3209 | 204 | 1.16 | 0.2465 |
| Baseline | Group*SampleName | Control | Beef puree | -7.3164 | 10.3209 | 204 | -0.71 | 0.4792 |
| Baseline | Group*SampleName | Control | Black bean puree | 0.1836 | 10.3209 | 204 | 0.02 | 0.9858 |
| Baseline | Group*SampleName | Control | Broccoli chopped | 15.7461 | 10.3209 | 204 | 1.53 | 0.1286 |
| Baseline | Group*SampleName | Control | Broccoli puree | 9.8086 | 10.3209 | 204 | 0.95 | 0.3431 |
| Baseline | Group*SampleName | Control | Chicken puree | -7.5039 | 10.3209 | 204 | -0.73 | 0.468 |
| Baseline | Group*SampleName | Control | Kale chopped | -14.0039 | 10.3209 | 204 | -1.36 | 0.1763 |
| Baseline | Group*SampleName | Control | Kale puree | -5.5664 | 10.3209 | 204 | -0.54 | 0.5902 |
| Baseline | Group*SampleName | Control | Oat puree | -20.0664 | 10.3209 | 204 | -1.94 | 0.0532 |
| Baseline | Group*SampleName | Control | Spinach chopped | -6.5039 | 10.3209 | 204 | -0.63 | 0.5293 |
| Baseline | Group*SampleName | Control | Spinach puree | -7.0664 | 10.3209 | 204 | -0.68 | 0.4943 |
| Week 1 | Group | Vegetable |  | -5.4244 | 5.8384 | 31 | -0.93 | 0.36 |
| Week 1 | Group | Control |  | -1.2493 | 5.7492 | 31 | -0.22 | 0.8294 |
| Week 1 | SampleName | | Asparagus chopped | 11.5237 | 7.0632 | 186 | 1.63 | 0.1045 |
| Week 1 | SampleName | | Asparagus puree | 7.3466 | 7.0632 | 186 | 1.04 | 0.2996 |
| Week 1 | SampleName | | Beef puree | -16.3305 | 7.0632 | 186 | -2.31 | 0.0219 |
| Week 1 | SampleName | | Black bean puree | 1.9889 | 7.0632 | 186 | 0.28 | 0.7786 |
| Week 1 | SampleName | | Broccoli chopped | 18.4542 | 7.0632 | 186 | 2.61 | 0.0097 |
| Week 1 | SampleName | | Broccoli puree | 8.6 | 7.0632 | 186 | 1.22 | 0.2249 |
| Week 1 | SampleName | | Chicken puree | -9.2993 | 7.0632 | 186 | -1.32 | 0.1896 |
| Week 1 | SampleName | | Kale chopped | -17.525 | 7.0632 | 186 | -2.48 | 0.014 |
| Week 1 | SampleName | | Kale puree | -17.5631 | 7.0632 | 186 | -2.49 | 0.0138 |
| Week 1 | SampleName | | Oat puree | -8.0701 | 7.0632 | 186 | -1.14 | 0.2547 |
| Week 1 | SampleName | | Spinach chopped | -7.5666 | 7.0632 | 186 | -1.07 | 0.2854 |
| Week 1 | SampleName | | Spinach puree | -11.6013 | 7.0632 | 186 | -1.64 | 0.1022 |
| Week 1 | Group*SampleName | Vegetable | Asparagus chopped | 11.0154 | 9.6937 | 185 | 1.14 | 0.2573 |
| Week 1 | Group*SampleName | Vegetable | Asparagus puree | -2.1513 | 9.6937 | 185 | -0.22 | 0.8246 |
| Week 1 | Group*SampleName | Vegetable | Beef puree | -27.8179 | 9.6937 | 185 | -2.87 | 0.0046 |
| Week 1 | Group*SampleName | Vegetable | Black bean puree | -6.9291 | 9.6937 | 185 | -0.71 | 0.4756 |
| Week 1 | Group*SampleName | Vegetable | Broccoli chopped | 13.1265 | 9.6937 | 185 | 1.35 | 0.1773 |
| Week 1 | Group*SampleName | Vegetable | Broccoli puree | 13.7932 | 9.6937 | 185 | 1.42 | 0.1564 |
| Week 1 | Group*SampleName | Vegetable | Chicken puree | -17.3179 | 9.6937 | 185 | -1.79 | 0.0757 |
| Week 1 | Group*SampleName | Vegetable | Kale chopped | -13.7068 | 9.6937 | 185 | -1.41 | 0.159 |
| Week 1 | Group*SampleName | Vegetable | Kale puree | -14.5957 | 9.6937 | 185 | -1.51 | 0.1339 |
| Week 1 | Group*SampleName | Vegetable | Oat puree | -2.4846 | 9.6937 | 185 | -0.26 | 0.798 |
| Week 1 | Group*SampleName | Vegetable | Spinach chopped | -6.0402 | 9.6937 | 185 | -0.62 | 0.534 |
| Week 1 | Group*SampleName | Vegetable | Spinach puree | -11.9846 | 9.6937 | 185 | -1.24 | 0.2179 |
| Week 1 | Group*SampleName | Control | Asparagus chopped | 12.0319 | 10.021 | 209 | 1.2 | 0.2312 |
| Week 1 | Group*SampleName | Control | Asparagus puree | 16.8444 | 10.021 | 209 | 1.68 | 0.0943 |
| Week 1 | Group*SampleName | Control | Beef puree | -4.8431 | 10.021 | 209 | -0.48 | 0.6294 |
| Week 1 | Group*SampleName | Control | Black bean puree | 10.9069 | 10.021 | 209 | 1.09 | 0.2777 |
| Week 1 | Group*SampleName | Control | Broccoli chopped | 23.7819 | 10.021 | 209 | 2.37 | 0.0185 |
| Week 1 | Group*SampleName | Control | Broccoli puree | 3.4069 | 10.021 | 209 | 0.34 | 0.7342 |
| Week 1 | Group*SampleName | Control | Chicken puree | -1.2806 | 10.021 | 209 | -0.13 | 0.8984 |
| Week 1 | Group*SampleName | Control | Kale chopped | -21.3431 | 10.021 | 209 | -2.13 | 0.0344 |
| Week 1 | Group*SampleName | Control | Kale puree | -20.5306 | 10.021 | 209 | -2.05 | 0.0417 |
| Week 1 | Group*SampleName | Control | Oat puree | -13.6556 | 10.021 | 209 | -1.36 | 0.1744 |
| Week 1 | Group*SampleName | Control | Spinach chopped | -9.0931 | 10.021 | 209 | -0.91 | 0.3652 |
| Week 1 | Group*SampleName | Control | Spinach puree | -11.2181 | 10.021 | 209 | -1.12 | 0.2642 |
| Week 2 | Group | Vegetable |  | -0.3531 | 6.9139 | 31 | -0.05 | 0.9596 |
| Week 2 | Group | Control |  | -1.136 | 6.8083 | 31 | -0.17 | 0.8686 |
| Week 2 | SampleName | | Asparagus chopped | 6.3093 | 7.3713 | 128 | 0.86 | 0.3936 |
| Week 2 | SampleName | | Asparagus puree | 7.1912 | 7.3713 | 128 | 0.98 | 0.3311 |
| Week 2 | SampleName | | Beef puree | -7.6213 | 7.3713 | 128 | -1.03 | 0.3031 |
| Week 2 | SampleName | | Black bean puree | 2.5142 | 7.3713 | 128 | 0.34 | 0.7336 |
| Week 2 | SampleName | | Broccoli chopped | 15.719 | 7.3713 | 128 | 2.13 | 0.0349 |
| Week 2 | SampleName | | Broccoli puree | 15.1253 | 7.3713 | 128 | 2.05 | 0.0422 |
| Week 2 | SampleName | | Chicken puree | -8.0067 | 7.3713 | 128 | -1.09 | 0.2794 |
| Week 2 | SampleName | | Kale chopped | -17.3053 | 7.3713 | 128 | -2.35 | 0.0204 |
| Week 2 | SampleName | | Kale puree | -15.6004 | 7.3713 | 128 | -2.12 | 0.0363 |
| Week 2 | SampleName | | Oat puree | 2.4204 | 7.3713 | 128 | 0.33 | 0.7432 |
| Week 2 | SampleName | | Spinach chopped | 1.0211 | 7.3713 | 128 | 0.14 | 0.89 |
| Week 2 | SampleName | | Spinach puree | -10.7011 | 7.3713 | 128 | -1.45 | 0.149 |
| Week 2 | Group*SampleName | Vegetable | Asparagus chopped | -0.5475 | 10.1175 | 128 | -0.05 | 0.9569 |
| Week 2 | Group*SampleName | Vegetable | Asparagus puree | 3.3414 | 10.1175 | 128 | 0.33 | 0.7417 |
| Week 2 | Group*SampleName | Vegetable | Beef puree | -15.1586 | 10.1175 | 128 | -1.5 | 0.1365 |
| Week 2 | Group*SampleName | Vegetable | Black bean puree | -3.8253 | 10.1175 | 128 | -0.38 | 0.706 |
| Week 2 | Group*SampleName | Vegetable | Broccoli chopped | 12.3969 | 10.1175 | 128 | 1.23 | 0.2227 |
| Week 2 | Group*SampleName | Vegetable | Broccoli puree | 18.3969 | 10.1175 | 128 | 1.82 | 0.0714 |
| Week 2 | Group*SampleName | Vegetable | Chicken puree | -14.992 | 10.1175 | 128 | -1.48 | 0.1409 |
| Week 2 | Group*SampleName | Vegetable | Kale chopped | -11.2142 | 10.1175 | 128 | -1.11 | 0.2698 |
| Week 2 | Group*SampleName | Vegetable | Kale puree | -6.492 | 10.1175 | 128 | -0.64 | 0.5222 |
| Week 2 | Group*SampleName | Vegetable | Oat puree | 13.1747 | 10.1175 | 128 | 1.3 | 0.1952 |
| Week 2 | Group*SampleName | Vegetable | Spinach chopped | 7.0636 | 10.1175 | 128 | 0.7 | 0.4864 |
| Week 2 | Group*SampleName | Vegetable | Spinach puree | -6.3808 | 10.1175 | 128 | -0.63 | 0.5294 |
| Week 2 | Group*SampleName | Control | Asparagus chopped | 13.1661 | 10.3796 | 145 | 1.27 | 0.2067 |
| Week 2 | Group*SampleName | Control | Asparagus puree | 11.0411 | 10.3796 | 145 | 1.06 | 0.2892 |
| Week 2 | Group*SampleName | Control | Beef puree | -0.08388 | 10.3796 | 145 | -0.01 | 0.9936 |
| Week 2 | Group*SampleName | Control | Black bean puree | 8.8536 | 10.3796 | 145 | 0.85 | 0.3951 |
| Week 2 | Group*SampleName | Control | Broccoli chopped | 19.0411 | 10.3796 | 145 | 1.83 | 0.0686 |
| Week 2 | Group*SampleName | Control | Broccoli puree | 11.8536 | 10.3796 | 145 | 1.14 | 0.2553 |
| Week 2 | Group*SampleName | Control | Chicken puree | -1.0214 | 10.3796 | 145 | -0.1 | 0.9217 |
| Week 2 | Group*SampleName | Control | Kale chopped | -23.3964 | 10.3796 | 145 | -2.25 | 0.0257 |
| Week 2 | Group*SampleName | Control | Kale puree | -24.7089 | 10.3796 | 145 | -2.38 | 0.0186 |
| Week 2 | Group*SampleName | Control | Oat puree | -8.3339 | 10.3796 | 145 | -0.8 | 0.4233 |
| Week 2 | Group*SampleName | Control | Spinach chopped | -5.0214 | 10.3796 | 145 | -0.48 | 0.6293 |
| Week 2 | Group*SampleName | Control | Spinach puree | -15.0214 | 10.3796 | 145 | -1.45 | 0.15 |

### Differences between Least Squared Means

P-values (Probt) in this table indicate whether the estimate of the difference between the two means is significantly different from 0.

| **GameVisit** | **Effect** | **Group** | **SampleName** | **_Group** | **_SampleName** | **Estimate** | **StdErr** | **DF** | **tValue** | **Probt** |
| --- | --- | --- | --- | --- | --- | --- | --- | --- | --- | --- |
| Baseline | Group | Vegetable |  | Control |  | -7.0973 | 8.2025 | 31 | -0.87 | 0.3935 |
| Baseline | SampleName |  | Asparagus chopped |  | Asparagus puree | 3.0139 | 8.531 | 352 | 0.35 | 0.7241 |
| Baseline | SampleName |  | Asparagus chopped |  | Beef puree | 22.0868 | 8.531 | 352 | 2.59 | 0.01 |
| Baseline | SampleName |  | Asparagus chopped |  | Black bean puree | 12.2257 | 8.531 | 352 | 1.43 | 0.1527 |
| Baseline | SampleName |  | Asparagus chopped |  | Broccoli chopped | -7.8611 | 8.531 | 352 | -0.92 | 0.3574 |
| Baseline | SampleName |  | Asparagus chopped |  | Broccoli puree | -6.8646 | 8.531 | 352 | -0.8 | 0.4216 |
| Baseline | SampleName |  | Asparagus chopped |  | Chicken puree | 23.7917 | 8.531 | 352 | 2.79 | 0.0056 |
| Baseline | SampleName |  | Asparagus chopped |  | Kale chopped | 29.4861 | 8.531 | 352 | 3.46 | 0.0006 |
| Baseline | SampleName |  | Asparagus chopped |  | Kale puree | 18.3229 | 8.531 | 352 | 2.15 | 0.0324 |
| Baseline | SampleName |  | Asparagus chopped |  | Oat puree | 18.9896 | 8.531 | 352 | 2.23 | 0.0267 |
| Baseline | SampleName |  | Asparagus chopped |  | Spinach chopped | 12.875 | 8.531 | 352 | 1.51 | 0.1321 |
| Baseline | SampleName |  | Asparagus chopped |  | Spinach puree | 14.1562 | 8.531 | 352 | 1.66 | 0.0979 |
| Baseline | SampleName |  | Asparagus puree |  | Beef puree | 19.0729 | 8.531 | 352 | 2.24 | 0.026 |
| Baseline | SampleName |  | Asparagus puree |  | Black bean puree | 9.2118 | 8.531 | 352 | 1.08 | 0.281 |
| Baseline | SampleName |  | Asparagus puree |  | Broccoli chopped | -10.875 | 8.531 | 352 | -1.27 | 0.2032 |
| Baseline | SampleName |  | Asparagus puree |  | Broccoli puree | -9.8785 | 8.531 | 352 | -1.16 | 0.2477 |
| Baseline | SampleName |  | Asparagus puree |  | Chicken puree | 20.7778 | 8.531 | 352 | 2.44 | 0.0154 |
| Baseline | SampleName |  | Asparagus puree |  | Kale chopped | 26.4722 | 8.531 | 352 | 3.1 | 0.0021 |
| Baseline | SampleName |  | Asparagus puree |  | Kale puree | 15.309 | 8.531 | 352 | 1.79 | 0.0736 |
| Baseline | SampleName |  | Asparagus puree |  | Oat puree | 15.9757 | 8.531 | 352 | 1.87 | 0.0619 |
| Baseline | SampleName |  | Asparagus puree |  | Spinach chopped | 9.8611 | 8.531 | 352 | 1.16 | 0.2485 |
| Baseline | SampleName |  | Asparagus puree |  | Spinach puree | 11.1424 | 8.531 | 352 | 1.31 | 0.1924 |
| Baseline | SampleName |  | Beef puree |  | Black bean puree | -9.8611 | 8.531 | 352 | -1.16 | 0.2485 |
| Baseline | SampleName |  | Beef puree |  | Broccoli chopped | -29.9479 | 8.531 | 352 | -3.51 | 0.0005 |
| Baseline | SampleName |  | Beef puree |  | Broccoli puree | -28.9514 | 8.531 | 352 | -3.39 | 0.0008 |
| Baseline | SampleName |  | Beef puree |  | Chicken puree | 1.7049 | 8.531 | 352 | 0.2 | 0.8417 |
| Baseline | SampleName |  | Beef puree |  | Kale chopped | 7.3993 | 8.531 | 352 | 0.87 | 0.3863 |
| Baseline | SampleName |  | Beef puree |  | Kale puree | -3.7639 | 8.531 | 352 | -0.44 | 0.6593 |
| Baseline | SampleName |  | Beef puree |  | Oat puree | -3.0972 | 8.531 | 352 | -0.36 | 0.7168 |
| Baseline | SampleName |  | Beef puree |  | Spinach chopped | -9.2118 | 8.531 | 352 | -1.08 | 0.281 |
| Baseline | SampleName |  | Beef puree |  | Spinach puree | -7.9306 | 8.531 | 352 | -0.93 | 0.3532 |
| Baseline | SampleName |  | Black bean puree |  | Broccoli chopped | -20.0868 | 8.531 | 352 | -2.35 | 0.0191 |
| Baseline | SampleName |  | Black bean puree |  | Broccoli puree | -19.0903 | 8.531 | 352 | -2.24 | 0.0259 |
| Baseline | SampleName |  | Black bean puree |  | Chicken puree | 11.566 | 8.531 | 352 | 1.36 | 0.176 |
| Baseline | SampleName |  | Black bean puree |  | Kale chopped | 17.2604 | 8.531 | 352 | 2.02 | 0.0438 |
| Baseline | SampleName |  | Black bean puree |  | Kale puree | 6.0972 | 8.531 | 352 | 0.71 | 0.4753 |
| Baseline | SampleName |  | Black bean puree |  | Oat puree | 6.7639 | 8.531 | 352 | 0.79 | 0.4284 |
| Baseline | SampleName |  | Black bean puree |  | Spinach chopped | 0.6493 | 8.531 | 352 | 0.08 | 0.9394 |
| Baseline | SampleName |  | Black bean puree |  | Spinach puree | 1.9306 | 8.531 | 352 | 0.23 | 0.8211 |
| Baseline | SampleName |  | Broccoli chopped |  | Broccoli puree | 0.9965 | 8.531 | 352 | 0.12 | 0.9071 |
| Baseline | SampleName |  | Broccoli chopped |  | Chicken puree | 31.6528 | 8.531 | 352 | 3.71 | 0.0002 |
| Baseline | SampleName |  | Broccoli chopped |  | Kale chopped | 37.3472 | 8.531 | 352 | 4.38 | <.0001 |
| Baseline | SampleName |  | Broccoli chopped |  | Kale puree | 26.184 | 8.531 | 352 | 3.07 | 0.0023 |
| Baseline | SampleName |  | Broccoli chopped |  | Oat puree | 26.8507 | 8.531 | 352 | 3.15 | 0.0018 |
| Baseline | SampleName |  | Broccoli chopped |  | Spinach chopped | 20.7361 | 8.531 | 352 | 2.43 | 0.0156 |
| Baseline | SampleName |  | Broccoli chopped |  | Spinach puree | 22.0174 | 8.531 | 352 | 2.58 | 0.0103 |
| Baseline | SampleName |  | Broccoli puree |  | Chicken puree | 30.6563 | 8.531 | 352 | 3.59 | 0.0004 |
| Baseline | SampleName |  | Broccoli puree |  | Kale chopped | 36.3507 | 8.531 | 352 | 4.26 | <.0001 |
| Baseline | SampleName |  | Broccoli puree |  | Kale puree | 25.1875 | 8.531 | 352 | 2.95 | 0.0034 |
| Baseline | SampleName |  | Broccoli puree |  | Oat puree | 25.8542 | 8.531 | 352 | 3.03 | 0.0026 |
| Baseline | SampleName |  | Broccoli puree |  | Spinach chopped | 19.7396 | 8.531 | 352 | 2.31 | 0.0213 |
| Baseline | SampleName |  | Broccoli puree |  | Spinach puree | 21.0208 | 8.531 | 352 | 2.46 | 0.0142 |
| Baseline | SampleName |  | Chicken puree |  | Kale chopped | 5.6944 | 8.531 | 352 | 0.67 | 0.5049 |
| Baseline | SampleName |  | Chicken puree |  | Kale puree | -5.4688 | 8.531 | 352 | -0.64 | 0.5219 |
| Baseline | SampleName |  | Chicken puree |  | Oat puree | -4.8021 | 8.531 | 352 | -0.56 | 0.5739 |
| Baseline | SampleName |  | Chicken puree |  | Spinach chopped | -10.9167 | 8.531 | 352 | -1.28 | 0.2015 |
| Baseline | SampleName |  | Chicken puree |  | Spinach puree | -9.6354 | 8.531 | 352 | -1.13 | 0.2595 |
| Baseline | SampleName |  | Kale chopped |  | Kale puree | -11.1632 | 8.531 | 352 | -1.31 | 0.1915 |
| Baseline | SampleName |  | Kale chopped |  | Oat puree | -10.4965 | 8.531 | 352 | -1.23 | 0.2194 |
| Baseline | SampleName |  | Kale chopped |  | Spinach chopped | -16.6111 | 8.531 | 352 | -1.95 | 0.0523 |
| Baseline | SampleName |  | Kale chopped |  | Spinach puree | -15.3299 | 8.531 | 352 | -1.8 | 0.0732 |
| Baseline | SampleName |  | Kale puree |  | Oat puree | 0.6667 | 8.531 | 352 | 0.08 | 0.9378 |
| Baseline | SampleName |  | Kale puree |  | Spinach chopped | -5.4479 | 8.531 | 352 | -0.64 | 0.5235 |
| Baseline | SampleName |  | Kale puree |  | Spinach puree | -4.1667 | 8.531 | 352 | -0.49 | 0.6256 |
| Baseline | SampleName |  | Oat puree |  | Spinach chopped | -6.1146 | 8.531 | 352 | -0.72 | 0.474 |
| Baseline | SampleName |  | Oat puree |  | Spinach puree | -4.8333 | 8.531 | 352 | -0.57 | 0.5714 |
| Baseline | SampleName |  | Spinach chopped |  | Spinach puree | 1.2812 | 8.531 | 352 | 0.15 | 0.8807 |
| Baseline | Group*SampleName | Vegetable | Asparagus chopped | Vegetable | Asparagus puree | 0.2778 | 11.7045 | 352 | 0.02 | 0.9811 |
| Baseline | Group*SampleName | Vegetable | Asparagus chopped | Vegetable | Beef puree | 19.1111 | 11.7045 | 352 | 1.63 | 0.1034 |
| Baseline | Group*SampleName | Vegetable | Asparagus chopped | Vegetable | Black bean puree | 6.8889 | 11.7045 | 352 | 0.59 | 0.5565 |
| Baseline | Group*SampleName | Vegetable | Asparagus chopped | Vegetable | Broccoli chopped | -17.7222 | 11.7045 | 352 | -1.51 | 0.1309 |
| Baseline | Group*SampleName | Vegetable | Asparagus chopped | Vegetable | Broccoli puree | -21.6667 | 11.7045 | 352 | -1.85 | 0.065 |
| Baseline | Group*SampleName | Vegetable | Asparagus chopped | Vegetable | Chicken puree | 22.3333 | 11.7045 | 352 | 1.91 | 0.0572 |
| Baseline | Group*SampleName | Vegetable | Asparagus chopped | Vegetable | Kale chopped | 27.2222 | 11.7045 | 352 | 2.33 | 0.0206 |
| Baseline | Group*SampleName | Vegetable | Asparagus chopped | Vegetable | Kale puree | 13.3333 | 11.7045 | 352 | 1.14 | 0.2554 |
| Baseline | Group*SampleName | Vegetable | Asparagus chopped | Vegetable | Oat puree | 0.1667 | 11.7045 | 352 | 0.01 | 0.9886 |
| Baseline | Group*SampleName | Vegetable | Asparagus chopped | Vegetable | Spinach chopped | 1.5 | 11.7045 | 352 | 0.13 | 0.8981 |
| Baseline | Group*SampleName | Vegetable | Asparagus chopped | Vegetable | Spinach puree | 3.5 | 11.7045 | 352 | 0.3 | 0.7651 |
| Baseline | Group*SampleName | Vegetable | Asparagus chopped | Control | Asparagus chopped | -21.3103 | 14.1671 | 205 | -1.5 | 0.1341 |
| Baseline | Group*SampleName | Vegetable | Asparagus puree | Vegetable | Beef puree | 18.8333 | 11.7045 | 352 | 1.61 | 0.1085 |
| Baseline | Group*SampleName | Vegetable | Asparagus puree | Vegetable | Black bean puree | 6.6111 | 11.7045 | 352 | 0.56 | 0.5725 |
| Baseline | Group*SampleName | Vegetable | Asparagus puree | Vegetable | Broccoli chopped | -18 | 11.7045 | 352 | -1.54 | 0.125 |
| Baseline | Group*SampleName | Vegetable | Asparagus puree | Vegetable | Broccoli puree | -21.9444 | 11.7045 | 352 | -1.87 | 0.0616 |
| Baseline | Group*SampleName | Vegetable | Asparagus puree | Vegetable | Chicken puree | 22.0556 | 11.7045 | 352 | 1.88 | 0.0603 |
| Baseline | Group*SampleName | Vegetable | Asparagus puree | Vegetable | Kale chopped | 26.9444 | 11.7045 | 352 | 2.3 | 0.0219 |
| Baseline | Group*SampleName | Vegetable | Asparagus puree | Vegetable | Kale puree | 13.0556 | 11.7045 | 352 | 1.12 | 0.2654 |
| Baseline | Group*SampleName | Vegetable | Asparagus puree | Vegetable | Oat puree | -0.1111 | 11.7045 | 352 | -0.01 | 0.9924 |
| Baseline | Group*SampleName | Vegetable | Asparagus puree | Vegetable | Spinach chopped | 1.2222 | 11.7045 | 352 | 0.1 | 0.9169 |
| Baseline | Group*SampleName | Vegetable | Asparagus puree | Vegetable | Spinach puree | 3.2222 | 11.7045 | 352 | 0.28 | 0.7832 |
| Baseline | Group*SampleName | Vegetable | Asparagus puree | Control | Asparagus puree | -15.8381 | 14.1671 | 205 | -1.12 | 0.2649 |
| Baseline | Group*SampleName | Vegetable | Beef puree | Vegetable | Black bean puree | -12.2222 | 11.7045 | 352 | -1.04 | 0.2971 |
| Baseline | Group*SampleName | Vegetable | Beef puree | Vegetable | Broccoli chopped | -36.8333 | 11.7045 | 352 | -3.15 | 0.0018 |
| Baseline | Group*SampleName | Vegetable | Beef puree | Vegetable | Broccoli puree | -40.7778 | 11.7045 | 352 | -3.48 | 0.0006 |
| Baseline | Group*SampleName | Vegetable | Beef puree | Vegetable | Chicken puree | 3.2222 | 11.7045 | 352 | 0.28 | 0.7832 |
| Baseline | Group*SampleName | Vegetable | Beef puree | Vegetable | Kale chopped | 8.1111 | 11.7045 | 352 | 0.69 | 0.4888 |
| Baseline | Group*SampleName | Vegetable | Beef puree | Vegetable | Kale puree | -5.7778 | 11.7045 | 352 | -0.49 | 0.6219 |
| Baseline | Group*SampleName | Vegetable | Beef puree | Vegetable | Oat puree | -18.9444 | 11.7045 | 352 | -1.62 | 0.1064 |
| Baseline | Group*SampleName | Vegetable | Beef puree | Vegetable | Spinach chopped | -17.6111 | 11.7045 | 352 | -1.5 | 0.1333 |
| Baseline | Group*SampleName | Vegetable | Beef puree | Vegetable | Spinach puree | -15.6111 | 11.7045 | 352 | -1.33 | 0.1831 |
| Baseline | Group*SampleName | Vegetable | Beef puree | Control | Beef puree | -15.3589 | 14.1671 | 205 | -1.08 | 0.2796 |
| Baseline | Group*SampleName | Vegetable | Black bean puree | Vegetable | Broccoli chopped | -24.6111 | 11.7045 | 352 | -2.1 | 0.0362 |
| Baseline | Group*SampleName | Vegetable | Black bean puree | Vegetable | Broccoli puree | -28.5556 | 11.7045 | 352 | -2.44 | 0.0152 |
| Baseline | Group*SampleName | Vegetable | Black bean puree | Vegetable | Chicken puree | 15.4444 | 11.7045 | 352 | 1.32 | 0.1878 |
| Baseline | Group*SampleName | Vegetable | Black bean puree | Vegetable | Kale chopped | 20.3333 | 11.7045 | 352 | 1.74 | 0.0832 |
| Baseline | Group*SampleName | Vegetable | Black bean puree | Vegetable | Kale puree | 6.4444 | 11.7045 | 352 | 0.55 | 0.5823 |
| Baseline | Group*SampleName | Vegetable | Black bean puree | Vegetable | Oat puree | -6.7222 | 11.7045 | 352 | -0.57 | 0.5661 |
| Baseline | Group*SampleName | Vegetable | Black bean puree | Vegetable | Spinach chopped | -5.3889 | 11.7045 | 352 | -0.46 | 0.6455 |
| Baseline | Group*SampleName | Vegetable | Black bean puree | Vegetable | Spinach puree | -3.3889 | 11.7045 | 352 | -0.29 | 0.7723 |
| Baseline | Group*SampleName | Vegetable | Black bean puree | Control | Black bean puree | -10.6367 | 14.1671 | 205 | -0.75 | 0.4536 |
| Baseline | Group*SampleName | Vegetable | Broccoli chopped | Vegetable | Broccoli puree | -3.9444 | 11.7045 | 352 | -0.34 | 0.7363 |
| Baseline | Group*SampleName | Vegetable | Broccoli chopped | Vegetable | Chicken puree | 40.0556 | 11.7045 | 352 | 3.42 | 0.0007 |
| Baseline | Group*SampleName | Vegetable | Broccoli chopped | Vegetable | Kale chopped | 44.9444 | 11.7045 | 352 | 3.84 | 0.0001 |
| Baseline | Group*SampleName | Vegetable | Broccoli chopped | Vegetable | Kale puree | 31.0556 | 11.7045 | 352 | 2.65 | 0.0083 |
| Baseline | Group*SampleName | Vegetable | Broccoli chopped | Vegetable | Oat puree | 17.8889 | 11.7045 | 352 | 1.53 | 0.1273 |
| Baseline | Group*SampleName | Vegetable | Broccoli chopped | Vegetable | Spinach chopped | 19.2222 | 11.7045 | 352 | 1.64 | 0.1014 |
| Baseline | Group*SampleName | Vegetable | Broccoli chopped | Vegetable | Spinach puree | 21.2222 | 11.7045 | 352 | 1.81 | 0.0707 |
| Baseline | Group*SampleName | Vegetable | Broccoli chopped | Control | Broccoli chopped | -1.5881 | 14.1671 | 205 | -0.11 | 0.9109 |
| Baseline | Group*SampleName | Vegetable | Broccoli puree | Vegetable | Chicken puree | 44 | 11.7045 | 352 | 3.76 | 0.0002 |
| Baseline | Group*SampleName | Vegetable | Broccoli puree | Vegetable | Kale chopped | 48.8889 | 11.7045 | 352 | 4.18 | <.0001 |
| Baseline | Group*SampleName | Vegetable | Broccoli puree | Vegetable | Kale puree | 35 | 11.7045 | 352 | 2.99 | 0.003 |
| Baseline | Group*SampleName | Vegetable | Broccoli puree | Vegetable | Oat puree | 21.8333 | 11.7045 | 352 | 1.87 | 0.063 |
| Baseline | Group*SampleName | Vegetable | Broccoli puree | Vegetable | Spinach chopped | 23.1667 | 11.7045 | 352 | 1.98 | 0.0486 |
| Baseline | Group*SampleName | Vegetable | Broccoli puree | Vegetable | Spinach puree | 25.1667 | 11.7045 | 352 | 2.15 | 0.0322 |
| Baseline | Group*SampleName | Vegetable | Broccoli puree | Control | Broccoli puree | 8.2939 | 14.1671 | 205 | 0.59 | 0.5589 |
| Baseline | Group*SampleName | Vegetable | Chicken puree | Vegetable | Kale chopped | 4.8889 | 11.7045 | 352 | 0.42 | 0.6764 |
| Baseline | Group*SampleName | Vegetable | Chicken puree | Vegetable | Kale puree | -9 | 11.7045 | 352 | -0.77 | 0.4424 |
| Baseline | Group*SampleName | Vegetable | Chicken puree | Vegetable | Oat puree | -22.1667 | 11.7045 | 352 | -1.89 | 0.0591 |
| Baseline | Group*SampleName | Vegetable | Chicken puree | Vegetable | Spinach chopped | -20.8333 | 11.7045 | 352 | -1.78 | 0.0759 |
| Baseline | Group*SampleName | Vegetable | Chicken puree | Vegetable | Spinach puree | -18.8333 | 11.7045 | 352 | -1.61 | 0.1085 |
| Baseline | Group*SampleName | Vegetable | Chicken puree | Control | Chicken puree | -18.3936 | 14.1671 | 205 | -1.3 | 0.1956 |
| Baseline | Group*SampleName | Vegetable | Kale chopped | Vegetable | Kale puree | -13.8889 | 11.7045 | 352 | -1.19 | 0.2362 |
| Baseline | Group*SampleName | Vegetable | Kale chopped | Vegetable | Oat puree | -27.0556 | 11.7045 | 352 | -2.31 | 0.0214 |
| Baseline | Group*SampleName | Vegetable | Kale chopped | Vegetable | Spinach chopped | -25.7222 | 11.7045 | 352 | -2.2 | 0.0286 |
| Baseline | Group*SampleName | Vegetable | Kale chopped | Vegetable | Spinach puree | -23.7222 | 11.7045 | 352 | -2.03 | 0.0434 |
| Baseline | Group*SampleName | Vegetable | Kale chopped | Control | Kale chopped | -16.7825 | 14.1671 | 205 | -1.18 | 0.2375 |
| Baseline | Group*SampleName | Vegetable | Kale puree | Vegetable | Oat puree | -13.1667 | 11.7045 | 352 | -1.12 | 0.2614 |
| Baseline | Group*SampleName | Vegetable | Kale puree | Vegetable | Spinach chopped | -11.8333 | 11.7045 | 352 | -1.01 | 0.3127 |
| Baseline | Group*SampleName | Vegetable | Kale puree | Vegetable | Spinach puree | -9.8333 | 11.7045 | 352 | -0.84 | 0.4014 |
| Baseline | Group*SampleName | Vegetable | Kale puree | Control | Kale puree | -11.3311 | 14.1671 | 205 | -0.8 | 0.4247 |
| Baseline | Group*SampleName | Vegetable | Oat puree | Vegetable | Spinach chopped | 1.3333 | 11.7045 | 352 | 0.11 | 0.9094 |
| Baseline | Group*SampleName | Vegetable | Oat puree | Vegetable | Spinach puree | 3.3333 | 11.7045 | 352 | 0.28 | 0.776 |
| Baseline | Group*SampleName | Vegetable | Oat puree | Control | Oat puree | 16.3355 | 14.1671 | 205 | 1.15 | 0.2502 |
| Baseline | Group*SampleName | Vegetable | Spinach chopped | Vegetable | Spinach puree | 2 | 11.7045 | 352 | 0.17 | 0.8644 |
| Baseline | Group*SampleName | Vegetable | Spinach chopped | Control | Spinach chopped | 1.4397 | 14.1671 | 205 | 0.1 | 0.9192 |
| Baseline | Group*SampleName | Vegetable | Spinach puree | Control | Spinach puree | 0.002202 | 14.1671 | 205 | 0 | 0.9999 |
| Baseline | Group*SampleName | Control | Asparagus chopped | Control | Asparagus puree | 5.75 | 12.4145 | 352 | 0.46 | 0.6435 |
| Baseline | Group*SampleName | Control | Asparagus chopped | Control | Beef puree | 25.0625 | 12.4145 | 352 | 2.02 | 0.0443 |
| Baseline | Group*SampleName | Control | Asparagus chopped | Control | Black bean puree | 17.5625 | 12.4145 | 352 | 1.41 | 0.158 |
| Baseline | Group*SampleName | Control | Asparagus chopped | Control | Broccoli chopped | 2 | 12.4145 | 352 | 0.16 | 0.8721 |
| Baseline | Group*SampleName | Control | Asparagus chopped | Control | Broccoli puree | 7.9375 | 12.4145 | 352 | 0.64 | 0.523 |
| Baseline | Group*SampleName | Control | Asparagus chopped | Control | Chicken puree | 25.25 | 12.4145 | 352 | 2.03 | 0.0427 |
| Baseline | Group*SampleName | Control | Asparagus chopped | Control | Kale chopped | 31.75 | 12.4145 | 352 | 2.56 | 0.011 |
| Baseline | Group*SampleName | Control | Asparagus chopped | Control | Kale puree | 23.3125 | 12.4145 | 352 | 1.88 | 0.0612 |
| Baseline | Group*SampleName | Control | Asparagus chopped | Control | Oat puree | 37.8125 | 12.4145 | 352 | 3.05 | 0.0025 |
| Baseline | Group*SampleName | Control | Asparagus chopped | Control | Spinach chopped | 24.25 | 12.4145 | 352 | 1.95 | 0.0516 |
| Baseline | Group*SampleName | Control | Asparagus chopped | Control | Spinach puree | 24.8125 | 12.4145 | 352 | 2 | 0.0464 |
| Baseline | Group*SampleName | Control | Asparagus puree | Control | Beef puree | 19.3125 | 12.4145 | 352 | 1.56 | 0.1207 |
| Baseline | Group*SampleName | Control | Asparagus puree | Control | Black bean puree | 11.8125 | 12.4145 | 352 | 0.95 | 0.342 |
| Baseline | Group*SampleName | Control | Asparagus puree | Control | Broccoli chopped | -3.75 | 12.4145 | 352 | -0.3 | 0.7628 |
| Baseline | Group*SampleName | Control | Asparagus puree | Control | Broccoli puree | 2.1875 | 12.4145 | 352 | 0.18 | 0.8602 |
| Baseline | Group*SampleName | Control | Asparagus puree | Control | Chicken puree | 19.5 | 12.4145 | 352 | 1.57 | 0.1171 |
| Baseline | Group*SampleName | Control | Asparagus puree | Control | Kale chopped | 26 | 12.4145 | 352 | 2.09 | 0.0369 |
| Baseline | Group*SampleName | Control | Asparagus puree | Control | Kale puree | 17.5625 | 12.4145 | 352 | 1.41 | 0.158 |
| Baseline | Group*SampleName | Control | Asparagus puree | Control | Oat puree | 32.0625 | 12.4145 | 352 | 2.58 | 0.0102 |
| Baseline | Group*SampleName | Control | Asparagus puree | Control | Spinach chopped | 18.5 | 12.4145 | 352 | 1.49 | 0.1371 |
| Baseline | Group*SampleName | Control | Asparagus puree | Control | Spinach puree | 19.0625 | 12.4145 | 352 | 1.54 | 0.1256 |
| Baseline | Group*SampleName | Control | Beef puree | Control | Black bean puree | -7.5 | 12.4145 | 352 | -0.6 | 0.5461 |
| Baseline | Group*SampleName | Control | Beef puree | Control | Broccoli chopped | -23.0625 | 12.4145 | 352 | -1.86 | 0.064 |
| Baseline | Group*SampleName | Control | Beef puree | Control | Broccoli puree | -17.125 | 12.4145 | 352 | -1.38 | 0.1686 |
| Baseline | Group*SampleName | Control | Beef puree | Control | Chicken puree | 0.1875 | 12.4145 | 352 | 0.02 | 0.988 |
| Baseline | Group*SampleName | Control | Beef puree | Control | Kale chopped | 6.6875 | 12.4145 | 352 | 0.54 | 0.5904 |
| Baseline | Group*SampleName | Control | Beef puree | Control | Kale puree | -1.75 | 12.4145 | 352 | -0.14 | 0.888 |
| Baseline | Group*SampleName | Control | Beef puree | Control | Oat puree | 12.75 | 12.4145 | 352 | 1.03 | 0.3051 |
| Baseline | Group*SampleName | Control | Beef puree | Control | Spinach chopped | -0.8125 | 12.4145 | 352 | -0.07 | 0.9479 |
| Baseline | Group*SampleName | Control | Beef puree | Control | Spinach puree | -0.25 | 12.4145 | 352 | -0.02 | 0.9839 |
| Baseline | Group*SampleName | Control | Black bean puree | Control | Broccoli chopped | -15.5625 | 12.4145 | 352 | -1.25 | 0.2108 |
| Baseline | Group*SampleName | Control | Black bean puree | Control | Broccoli puree | -9.625 | 12.4145 | 352 | -0.78 | 0.4387 |
| Baseline | Group*SampleName | Control | Black bean puree | Control | Chicken puree | 7.6875 | 12.4145 | 352 | 0.62 | 0.5362 |
| Baseline | Group*SampleName | Control | Black bean puree | Control | Kale chopped | 14.1875 | 12.4145 | 352 | 1.14 | 0.2539 |
| Baseline | Group*SampleName | Control | Black bean puree | Control | Kale puree | 5.75 | 12.4145 | 352 | 0.46 | 0.6435 |
| Baseline | Group*SampleName | Control | Black bean puree | Control | Oat puree | 20.25 | 12.4145 | 352 | 1.63 | 0.1038 |
| Baseline | Group*SampleName | Control | Black bean puree | Control | Spinach chopped | 6.6875 | 12.4145 | 352 | 0.54 | 0.5904 |
| Baseline | Group*SampleName | Control | Black bean puree | Control | Spinach puree | 7.25 | 12.4145 | 352 | 0.58 | 0.5596 |
| Baseline | Group*SampleName | Control | Broccoli chopped | Control | Broccoli puree | 5.9375 | 12.4145 | 352 | 0.48 | 0.6328 |
| Baseline | Group*SampleName | Control | Broccoli chopped | Control | Chicken puree | 23.25 | 12.4145 | 352 | 1.87 | 0.0619 |
| Baseline | Group*SampleName | Control | Broccoli chopped | Control | Kale chopped | 29.75 | 12.4145 | 352 | 2.4 | 0.0171 |
| Baseline | Group*SampleName | Control | Broccoli chopped | Control | Kale puree | 21.3125 | 12.4145 | 352 | 1.72 | 0.0869 |
| Baseline | Group*SampleName | Control | Broccoli chopped | Control | Oat puree | 35.8125 | 12.4145 | 352 | 2.88 | 0.0042 |
| Baseline | Group*SampleName | Control | Broccoli chopped | Control | Spinach chopped | 22.25 | 12.4145 | 352 | 1.79 | 0.0739 |
| Baseline | Group*SampleName | Control | Broccoli chopped | Control | Spinach puree | 22.8125 | 12.4145 | 352 | 1.84 | 0.067 |
| Baseline | Group*SampleName | Control | Broccoli puree | Control | Chicken puree | 17.3125 | 12.4145 | 352 | 1.39 | 0.164 |
| Baseline | Group*SampleName | Control | Broccoli puree | Control | Kale chopped | 23.8125 | 12.4145 | 352 | 1.92 | 0.0559 |
| Baseline | Group*SampleName | Control | Broccoli puree | Control | Kale puree | 15.375 | 12.4145 | 352 | 1.24 | 0.2164 |
| Baseline | Group*SampleName | Control | Broccoli puree | Control | Oat puree | 29.875 | 12.4145 | 352 | 2.41 | 0.0166 |
| Baseline | Group*SampleName | Control | Broccoli puree | Control | Spinach chopped | 16.3125 | 12.4145 | 352 | 1.31 | 0.1897 |
| Baseline | Group*SampleName | Control | Broccoli puree | Control | Spinach puree | 16.875 | 12.4145 | 352 | 1.36 | 0.1749 |
| Baseline | Group*SampleName | Control | Chicken puree | Control | Kale chopped | 6.5 | 12.4145 | 352 | 0.52 | 0.6009 |
| Baseline | Group*SampleName | Control | Chicken puree | Control | Kale puree | -1.9375 | 12.4145 | 352 | -0.16 | 0.8761 |
| Baseline | Group*SampleName | Control | Chicken puree | Control | Oat puree | 12.5625 | 12.4145 | 352 | 1.01 | 0.3123 |
| Baseline | Group*SampleName | Control | Chicken puree | Control | Spinach chopped | -1 | 12.4145 | 352 | -0.08 | 0.9358 |
| Baseline | Group*SampleName | Control | Chicken puree | Control | Spinach puree | -0.4375 | 12.4145 | 352 | -0.04 | 0.9719 |
| Baseline | Group*SampleName | Control | Kale chopped | Control | Kale puree | -8.4375 | 12.4145 | 352 | -0.68 | 0.4972 |
| Baseline | Group*SampleName | Control | Kale chopped | Control | Oat puree | 6.0625 | 12.4145 | 352 | 0.49 | 0.6256 |
| Baseline | Group*SampleName | Control | Kale chopped | Control | Spinach chopped | -7.5 | 12.4145 | 352 | -0.6 | 0.5461 |
| Baseline | Group*SampleName | Control | Kale chopped | Control | Spinach puree | -6.9375 | 12.4145 | 352 | -0.56 | 0.5766 |
| Baseline | Group*SampleName | Control | Kale puree | Control | Oat puree | 14.5 | 12.4145 | 352 | 1.17 | 0.2436 |
| Baseline | Group*SampleName | Control | Kale puree | Control | Spinach chopped | 0.9375 | 12.4145 | 352 | 0.08 | 0.9398 |
| Baseline | Group*SampleName | Control | Kale puree | Control | Spinach puree | 1.5 | 12.4145 | 352 | 0.12 | 0.9039 |
| Baseline | Group*SampleName | Control | Oat puree | Control | Spinach chopped | -13.5625 | 12.4145 | 352 | -1.09 | 0.2754 |
| Baseline | Group*SampleName | Control | Oat puree | Control | Spinach puree | -13 | 12.4145 | 352 | -1.05 | 0.2957 |
| Baseline | Group*SampleName | Control | Spinach chopped | Control | Spinach puree | 0.5625 | 12.4145 | 352 | 0.05 | 0.9639 |
| Week 1 | Group | Vegetable |  | Control |  | -4.1751 | 7.8725 | 31 | -0.53 | 0.5997 |
| Week 1 | SampleName |  | Asparagus chopped |  | Asparagus puree | 4.1771 | 8.3312 | 352 | 0.5 | 0.6164 |
| Week 1 | SampleName |  | Asparagus chopped |  | Beef puree | 27.8542 | 8.3312 | 352 | 3.34 | 0.0009 |
| Week 1 | SampleName |  | Asparagus chopped |  | Black bean puree | 9.5347 | 8.3312 | 352 | 1.14 | 0.2532 |
| Week 1 | SampleName |  | Asparagus chopped |  | Broccoli chopped | -6.9306 | 8.3312 | 352 | -0.83 | 0.406 |
| Week 1 | SampleName |  | Asparagus chopped |  | Broccoli puree | 2.9236 | 8.3312 | 352 | 0.35 | 0.7259 |
| Week 1 | SampleName |  | Asparagus chopped |  | Chicken puree | 20.8229 | 8.3312 | 352 | 2.5 | 0.0129 |
| Week 1 | SampleName |  | Asparagus chopped |  | Kale chopped | 29.0486 | 8.3312 | 352 | 3.49 | 0.0006 |
| Week 1 | SampleName |  | Asparagus chopped |  | Kale puree | 29.0868 | 8.3312 | 352 | 3.49 | 0.0005 |
| Week 1 | SampleName |  | Asparagus chopped |  | Oat puree | 19.5938 | 8.3312 | 352 | 2.35 | 0.0192 |
| Week 1 | SampleName |  | Asparagus chopped |  | Spinach chopped | 19.0903 | 8.3312 | 352 | 2.29 | 0.0225 |
| Week 1 | SampleName |  | Asparagus chopped |  | Spinach puree | 23.125 | 8.3312 | 352 | 2.78 | 0.0058 |
| Week 1 | SampleName |  | Asparagus puree |  | Beef puree | 23.6771 | 8.3312 | 352 | 2.84 | 0.0047 |
| Week 1 | SampleName |  | Asparagus puree |  | Black bean puree | 5.3576 | 8.3312 | 352 | 0.64 | 0.5206 |
| Week 1 | SampleName |  | Asparagus puree |  | Broccoli chopped | -11.1076 | 8.3312 | 352 | -1.33 | 0.1833 |
| Week 1 | SampleName |  | Asparagus puree |  | Broccoli puree | -1.2535 | 8.3312 | 352 | -0.15 | 0.8805 |
| Week 1 | SampleName |  | Asparagus puree |  | Chicken puree | 16.6458 | 8.3312 | 352 | 2 | 0.0465 |
| Week 1 | SampleName |  | Asparagus puree |  | Kale chopped | 24.8715 | 8.3312 | 352 | 2.99 | 0.003 |
| Week 1 | SampleName |  | Asparagus puree |  | Kale puree | 24.9097 | 8.3312 | 352 | 2.99 | 0.003 |
| Week 1 | SampleName |  | Asparagus puree |  | Oat puree | 15.4167 | 8.3312 | 352 | 1.85 | 0.0651 |
| Week 1 | SampleName |  | Asparagus puree |  | Spinach chopped | 14.9132 | 8.3312 | 352 | 1.79 | 0.0743 |
| Week 1 | SampleName |  | Asparagus puree |  | Spinach puree | 18.9479 | 8.3312 | 352 | 2.27 | 0.0235 |
| Week 1 | SampleName |  | Beef puree |  | Black bean puree | -18.3194 | 8.3312 | 352 | -2.2 | 0.0285 |
| Week 1 | SampleName |  | Beef puree |  | Broccoli chopped | -34.7847 | 8.3312 | 352 | -4.18 | <.0001 |
| Week 1 | SampleName |  | Beef puree |  | Broccoli puree | -24.9306 | 8.3312 | 352 | -2.99 | 0.003 |
| Week 1 | SampleName |  | Beef puree |  | Chicken puree | -7.0312 | 8.3312 | 352 | -0.84 | 0.3993 |
| Week 1 | SampleName |  | Beef puree |  | Kale chopped | 1.1944 | 8.3312 | 352 | 0.14 | 0.8861 |
| Week 1 | SampleName |  | Beef puree |  | Kale puree | 1.2326 | 8.3312 | 352 | 0.15 | 0.8825 |
| Week 1 | SampleName |  | Beef puree |  | Oat puree | -8.2604 | 8.3312 | 352 | -0.99 | 0.3221 |
| Week 1 | SampleName |  | Beef puree |  | Spinach chopped | -8.7639 | 8.3312 | 352 | -1.05 | 0.2935 |
| Week 1 | SampleName |  | Beef puree |  | Spinach puree | -4.7292 | 8.3312 | 352 | -0.57 | 0.5706 |
| Week 1 | SampleName |  | Black bean puree |  | Broccoli chopped | -16.4653 | 8.3312 | 352 | -1.98 | 0.0489 |
| Week 1 | SampleName |  | Black bean puree |  | Broccoli puree | -6.6111 | 8.3312 | 352 | -0.79 | 0.428 |
| Week 1 | SampleName |  | Black bean puree |  | Chicken puree | 11.2882 | 8.3312 | 352 | 1.35 | 0.1763 |
| Week 1 | SampleName |  | Black bean puree |  | Kale chopped | 19.5139 | 8.3312 | 352 | 2.34 | 0.0197 |
| Week 1 | SampleName |  | Black bean puree |  | Kale puree | 19.5521 | 8.3312 | 352 | 2.35 | 0.0195 |
| Week 1 | SampleName |  | Black bean puree |  | Oat puree | 10.059 | 8.3312 | 352 | 1.21 | 0.2281 |
| Week 1 | SampleName |  | Black bean puree |  | Spinach chopped | 9.5556 | 8.3312 | 352 | 1.15 | 0.2522 |
| Week 1 | SampleName |  | Black bean puree |  | Spinach puree | 13.5903 | 8.3312 | 352 | 1.63 | 0.1037 |
| Week 1 | SampleName |  | Broccoli chopped |  | Broccoli puree | 9.8542 | 8.3312 | 352 | 1.18 | 0.2377 |
| Week 1 | SampleName |  | Broccoli chopped |  | Chicken puree | 27.7535 | 8.3312 | 352 | 3.33 | 0.001 |
| Week 1 | SampleName |  | Broccoli chopped |  | Kale chopped | 35.9792 | 8.3312 | 352 | 4.32 | <.0001 |
| Week 1 | SampleName |  | Broccoli chopped |  | Kale puree | 36.0174 | 8.3312 | 352 | 4.32 | <.0001 |
| Week 1 | SampleName |  | Broccoli chopped |  | Oat puree | 26.5243 | 8.3312 | 352 | 3.18 | 0.0016 |
| Week 1 | SampleName |  | Broccoli chopped |  | Spinach chopped | 26.0208 | 8.3312 | 352 | 3.12 | 0.0019 |
| Week 1 | SampleName |  | Broccoli chopped |  | Spinach puree | 30.0556 | 8.3312 | 352 | 3.61 | 0.0004 |
| Week 1 | SampleName |  | Broccoli puree |  | Chicken puree | 17.8993 | 8.3312 | 352 | 2.15 | 0.0324 |
| Week 1 | SampleName |  | Broccoli puree |  | Kale chopped | 26.125 | 8.3312 | 352 | 3.14 | 0.0019 |
| Week 1 | SampleName |  | Broccoli puree |  | Kale puree | 26.1632 | 8.3312 | 352 | 3.14 | 0.0018 |
| Week 1 | SampleName |  | Broccoli puree |  | Oat puree | 16.6701 | 8.3312 | 352 | 2 | 0.0462 |
| Week 1 | SampleName |  | Broccoli puree |  | Spinach chopped | 16.1667 | 8.3312 | 352 | 1.94 | 0.0531 |
| Week 1 | SampleName |  | Broccoli puree |  | Spinach puree | 20.2014 | 8.3312 | 352 | 2.42 | 0.0158 |
| Week 1 | SampleName |  | Chicken puree |  | Kale chopped | 8.2257 | 8.3312 | 352 | 0.99 | 0.3242 |
| Week 1 | SampleName |  | Chicken puree |  | Kale puree | 8.2639 | 8.3312 | 352 | 0.99 | 0.3219 |
| Week 1 | SampleName |  | Chicken puree |  | Oat puree | -1.2292 | 8.3312 | 352 | -0.15 | 0.8828 |
| Week 1 | SampleName |  | Chicken puree |  | Spinach chopped | -1.7326 | 8.3312 | 352 | -0.21 | 0.8354 |
| Week 1 | SampleName |  | Chicken puree |  | Spinach puree | 2.3021 | 8.3312 | 352 | 0.28 | 0.7825 |
| Week 1 | SampleName |  | Kale chopped |  | Kale puree | 0.03819 | 8.3312 | 352 | 0 | 0.9963 |
| Week 1 | SampleName |  | Kale chopped |  | Oat puree | -9.4549 | 8.3312 | 352 | -1.13 | 0.2572 |
| Week 1 | SampleName |  | Kale chopped |  | Spinach chopped | -9.9583 | 8.3312 | 352 | -1.2 | 0.2328 |
| Week 1 | SampleName |  | Kale chopped |  | Spinach puree | -5.9236 | 8.3312 | 352 | -0.71 | 0.4775 |
| Week 1 | SampleName |  | Kale puree |  | Oat puree | -9.4931 | 8.3312 | 352 | -1.14 | 0.2553 |
| Week 1 | SampleName |  | Kale puree |  | Spinach chopped | -9.9965 | 8.3312 | 352 | -1.2 | 0.231 |
| Week 1 | SampleName |  | Kale puree |  | Spinach puree | -5.9618 | 8.3312 | 352 | -0.72 | 0.4747 |
| Week 1 | SampleName |  | Oat puree |  | Spinach chopped | -0.5035 | 8.3312 | 352 | -0.06 | 0.9518 |
| Week 1 | SampleName |  | Oat puree |  | Spinach puree | 3.5312 | 8.3312 | 352 | 0.42 | 0.6719 |
| Week 1 | SampleName |  | Spinach chopped |  | Spinach puree | 4.0347 | 8.3312 | 352 | 0.48 | 0.6285 |
| Week 1 | Group*SampleName | Vegetable | Asparagus chopped | Vegetable | Asparagus puree | 13.1667 | 11.4303 | 352 | 1.15 | 0.2501 |
| Week 1 | Group*SampleName | Vegetable | Asparagus chopped | Vegetable | Beef puree | 38.8333 | 11.4303 | 352 | 3.4 | 0.0008 |
| Week 1 | Group*SampleName | Vegetable | Asparagus chopped | Vegetable | Black bean puree | 17.9444 | 11.4303 | 352 | 1.57 | 0.1173 |
| Week 1 | Group*SampleName | Vegetable | Asparagus chopped | Vegetable | Broccoli chopped | -2.1111 | 11.4303 | 352 | -0.18 | 0.8536 |
| Week 1 | Group*SampleName | Vegetable | Asparagus chopped | Vegetable | Broccoli puree | -2.7778 | 11.4303 | 352 | -0.24 | 0.8081 |
| Week 1 | Group*SampleName | Vegetable | Asparagus chopped | Vegetable | Chicken puree | 28.3333 | 11.4303 | 352 | 2.48 | 0.0137 |
| Week 1 | Group*SampleName | Vegetable | Asparagus chopped | Vegetable | Kale chopped | 24.7222 | 11.4303 | 352 | 2.16 | 0.0312 |
| Week 1 | Group*SampleName | Vegetable | Asparagus chopped | Vegetable | Kale puree | 25.6111 | 11.4303 | 352 | 2.24 | 0.0257 |
| Week 1 | Group*SampleName | Vegetable | Asparagus chopped | Vegetable | Oat puree | 13.5 | 11.4303 | 352 | 1.18 | 0.2384 |
| Week 1 | Group*SampleName | Vegetable | Asparagus chopped | Vegetable | Spinach chopped | 17.0556 | 11.4303 | 352 | 1.49 | 0.1366 |
| Week 1 | Group*SampleName | Vegetable | Asparagus chopped | Vegetable | Spinach puree | 23 | 11.4303 | 352 | 2.01 | 0.045 |
| Week 1 | Group*SampleName | Vegetable | Asparagus chopped | Control | Asparagus chopped | -1.0165 | 13.7559 | 211 | -0.07 | 0.9412 |
| Week 1 | Group*SampleName | Vegetable | Asparagus puree | Vegetable | Beef puree | 25.6667 | 11.4303 | 352 | 2.25 | 0.0254 |
| Week 1 | Group*SampleName | Vegetable | Asparagus puree | Vegetable | Black bean puree | 4.7778 | 11.4303 | 352 | 0.42 | 0.6762 |
| Week 1 | Group*SampleName | Vegetable | Asparagus puree | Vegetable | Broccoli chopped | -15.2778 | 11.4303 | 352 | -1.34 | 0.1822 |
| Week 1 | Group*SampleName | Vegetable | Asparagus puree | Vegetable | Broccoli puree | -15.9444 | 11.4303 | 352 | -1.39 | 0.1639 |
| Week 1 | Group*SampleName | Vegetable | Asparagus puree | Vegetable | Chicken puree | 15.1667 | 11.4303 | 352 | 1.33 | 0.1854 |
| Week 1 | Group*SampleName | Vegetable | Asparagus puree | Vegetable | Kale chopped | 11.5556 | 11.4303 | 352 | 1.01 | 0.3127 |
| Week 1 | Group*SampleName | Vegetable | Asparagus puree | Vegetable | Kale puree | 12.4444 | 11.4303 | 352 | 1.09 | 0.277 |
| Week 1 | Group*SampleName | Vegetable | Asparagus puree | Vegetable | Oat puree | 0.3333 | 11.4303 | 352 | 0.03 | 0.9768 |
| Week 1 | Group*SampleName | Vegetable | Asparagus puree | Vegetable | Spinach chopped | 3.8889 | 11.4303 | 352 | 0.34 | 0.7339 |
| Week 1 | Group*SampleName | Vegetable | Asparagus puree | Vegetable | Spinach puree | 9.8333 | 11.4303 | 352 | 0.86 | 0.3902 |
| Week 1 | Group*SampleName | Vegetable | Asparagus puree | Control | Asparagus puree | -18.9957 | 13.7559 | 211 | -1.38 | 0.1688 |
| Week 1 | Group*SampleName | Vegetable | Beef puree | Vegetable | Black bean puree | -20.8889 | 11.4303 | 352 | -1.83 | 0.0685 |
| Week 1 | Group*SampleName | Vegetable | Beef puree | Vegetable | Broccoli chopped | -40.9444 | 11.4303 | 352 | -3.58 | 0.0004 |
| Week 1 | Group*SampleName | Vegetable | Beef puree | Vegetable | Broccoli puree | -41.6111 | 11.4303 | 352 | -3.64 | 0.0003 |
| Week 1 | Group*SampleName | Vegetable | Beef puree | Vegetable | Chicken puree | -10.5 | 11.4303 | 352 | -0.92 | 0.3589 |
| Week 1 | Group*SampleName | Vegetable | Beef puree | Vegetable | Kale chopped | -14.1111 | 11.4303 | 352 | -1.23 | 0.2178 |
| Week 1 | Group*SampleName | Vegetable | Beef puree | Vegetable | Kale puree | -13.2222 | 11.4303 | 352 | -1.16 | 0.2481 |
| Week 1 | Group*SampleName | Vegetable | Beef puree | Vegetable | Oat puree | -25.3333 | 11.4303 | 352 | -2.22 | 0.0273 |
| Week 1 | Group*SampleName | Vegetable | Beef puree | Vegetable | Spinach chopped | -21.7778 | 11.4303 | 352 | -1.91 | 0.0576 |
| Week 1 | Group*SampleName | Vegetable | Beef puree | Vegetable | Spinach puree | -15.8333 | 11.4303 | 352 | -1.39 | 0.1669 |
| Week 1 | Group*SampleName | Vegetable | Beef puree | Control | Beef puree | -22.9749 | 13.7559 | 211 | -1.67 | 0.0964 |
| Week 1 | Group*SampleName | Vegetable | Black bean puree | Vegetable | Broccoli chopped | -20.0556 | 11.4303 | 352 | -1.75 | 0.0802 |
| Week 1 | Group*SampleName | Vegetable | Black bean puree | Vegetable | Broccoli puree | -20.7222 | 11.4303 | 352 | -1.81 | 0.0707 |
| Week 1 | Group*SampleName | Vegetable | Black bean puree | Vegetable | Chicken puree | 10.3889 | 11.4303 | 352 | 0.91 | 0.364 |
| Week 1 | Group*SampleName | Vegetable | Black bean puree | Vegetable | Kale chopped | 6.7778 | 11.4303 | 352 | 0.59 | 0.5536 |
| Week 1 | Group*SampleName | Vegetable | Black bean puree | Vegetable | Kale puree | 7.6667 | 11.4303 | 352 | 0.67 | 0.5028 |
| Week 1 | Group*SampleName | Vegetable | Black bean puree | Vegetable | Oat puree | -4.4444 | 11.4303 | 352 | -0.39 | 0.6976 |
| Week 1 | Group*SampleName | Vegetable | Black bean puree | Vegetable | Spinach chopped | -0.8889 | 11.4303 | 352 | -0.08 | 0.9381 |
| Week 1 | Group*SampleName | Vegetable | Black bean puree | Vegetable | Spinach puree | 5.0556 | 11.4303 | 352 | 0.44 | 0.6585 |
| Week 1 | Group*SampleName | Vegetable | Black bean puree | Control | Black bean puree | -17.836 | 13.7559 | 211 | -1.3 | 0.1962 |
| Week 1 | Group*SampleName | Vegetable | Broccoli chopped | Vegetable | Broccoli puree | -0.6667 | 11.4303 | 352 | -0.06 | 0.9535 |
| Week 1 | Group*SampleName | Vegetable | Broccoli chopped | Vegetable | Chicken puree | 30.4444 | 11.4303 | 352 | 2.66 | 0.0081 |
| Week 1 | Group*SampleName | Vegetable | Broccoli chopped | Vegetable | Kale chopped | 26.8333 | 11.4303 | 352 | 2.35 | 0.0194 |
| Week 1 | Group*SampleName | Vegetable | Broccoli chopped | Vegetable | Kale puree | 27.7222 | 11.4303 | 352 | 2.43 | 0.0158 |
| Week 1 | Group*SampleName | Vegetable | Broccoli chopped | Vegetable | Oat puree | 15.6111 | 11.4303 | 352 | 1.37 | 0.1729 |
| Week 1 | Group*SampleName | Vegetable | Broccoli chopped | Vegetable | Spinach chopped | 19.1667 | 11.4303 | 352 | 1.68 | 0.0945 |
| Week 1 | Group*SampleName | Vegetable | Broccoli chopped | Vegetable | Spinach puree | 25.1111 | 11.4303 | 352 | 2.2 | 0.0287 |
| Week 1 | Group*SampleName | Vegetable | Broccoli chopped | Control | Broccoli chopped | -10.6554 | 13.7559 | 211 | -0.77 | 0.4394 |
| Week 1 | Group*SampleName | Vegetable | Broccoli puree | Vegetable | Chicken puree | 31.1111 | 11.4303 | 352 | 2.72 | 0.0068 |
| Week 1 | Group*SampleName | Vegetable | Broccoli puree | Vegetable | Kale chopped | 27.5 | 11.4303 | 352 | 2.41 | 0.0166 |
| Week 1 | Group*SampleName | Vegetable | Broccoli puree | Vegetable | Kale puree | 28.3889 | 11.4303 | 352 | 2.48 | 0.0135 |
| Week 1 | Group*SampleName | Vegetable | Broccoli puree | Vegetable | Oat puree | 16.2778 | 11.4303 | 352 | 1.42 | 0.1553 |
| Week 1 | Group*SampleName | Vegetable | Broccoli puree | Vegetable | Spinach chopped | 19.8333 | 11.4303 | 352 | 1.74 | 0.0836 |
| Week 1 | Group*SampleName | Vegetable | Broccoli puree | Vegetable | Spinach puree | 25.7778 | 11.4303 | 352 | 2.26 | 0.0247 |
| Week 1 | Group*SampleName | Vegetable | Broccoli puree | Control | Broccoli puree | 10.3862 | 13.7559 | 211 | 0.76 | 0.4511 |
| Week 1 | Group*SampleName | Vegetable | Chicken puree | Vegetable | Kale chopped | -3.6111 | 11.4303 | 352 | -0.32 | 0.7522 |
| Week 1 | Group*SampleName | Vegetable | Chicken puree | Vegetable | Kale puree | -2.7222 | 11.4303 | 352 | -0.24 | 0.8119 |
| Week 1 | Group*SampleName | Vegetable | Chicken puree | Vegetable | Oat puree | -14.8333 | 11.4303 | 352 | -1.3 | 0.1952 |
| Week 1 | Group*SampleName | Vegetable | Chicken puree | Vegetable | Spinach chopped | -11.2778 | 11.4303 | 352 | -0.99 | 0.3245 |
| Week 1 | Group*SampleName | Vegetable | Chicken puree | Vegetable | Spinach puree | -5.3333 | 11.4303 | 352 | -0.47 | 0.6411 |
| Week 1 | Group*SampleName | Vegetable | Chicken puree | Control | Chicken puree | -16.0374 | 13.7559 | 211 | -1.17 | 0.245 |
| Week 1 | Group*SampleName | Vegetable | Kale chopped | Vegetable | Kale puree | 0.8889 | 11.4303 | 352 | 0.08 | 0.9381 |
| Week 1 | Group*SampleName | Vegetable | Kale chopped | Vegetable | Oat puree | -11.2222 | 11.4303 | 352 | -0.98 | 0.3269 |
| Week 1 | Group*SampleName | Vegetable | Kale chopped | Vegetable | Spinach chopped | -7.6667 | 11.4303 | 352 | -0.67 | 0.5028 |
| Week 1 | Group*SampleName | Vegetable | Kale chopped | Vegetable | Spinach puree | -1.7222 | 11.4303 | 352 | -0.15 | 0.8803 |
| Week 1 | Group*SampleName | Vegetable | Kale chopped | Control | Kale chopped | 7.6362 | 13.7559 | 211 | 0.56 | 0.5794 |
| Week 1 | Group*SampleName | Vegetable | Kale puree | Vegetable | Oat puree | -12.1111 | 11.4303 | 352 | -1.06 | 0.2901 |
| Week 1 | Group*SampleName | Vegetable | Kale puree | Vegetable | Spinach chopped | -8.5556 | 11.4303 | 352 | -0.75 | 0.4547 |
| Week 1 | Group*SampleName | Vegetable | Kale puree | Vegetable | Spinach puree | -2.6111 | 11.4303 | 352 | -0.23 | 0.8194 |
| Week 1 | Group*SampleName | Vegetable | Kale puree | Control | Kale puree | 5.9349 | 13.7559 | 211 | 0.43 | 0.6666 |
| Week 1 | Group*SampleName | Vegetable | Oat puree | Vegetable | Spinach chopped | 3.5556 | 11.4303 | 352 | 0.31 | 0.7559 |
| Week 1 | Group*SampleName | Vegetable | Oat puree | Vegetable | Spinach puree | 9.5 | 11.4303 | 352 | 0.83 | 0.4065 |
| Week 1 | Group*SampleName | Vegetable | Oat puree | Control | Oat puree | 11.171 | 13.7559 | 211 | 0.81 | 0.4177 |
| Week 1 | Group*SampleName | Vegetable | Spinach chopped | Vegetable | Spinach puree | 5.9444 | 11.4303 | 352 | 0.52 | 0.6033 |
| Week 1 | Group*SampleName | Vegetable | Spinach chopped | Control | Spinach chopped | 3.0529 | 13.7559 | 211 | 0.22 | 0.8246 |
| Week 1 | Group*SampleName | Vegetable | Spinach puree | Control | Spinach puree | -0.7665 | 13.7559 | 211 | -0.06 | 0.9556 |
| Week 1 | Group*SampleName | Control | Asparagus chopped | Control | Asparagus puree | -4.8125 | 12.1236 | 352 | -0.4 | 0.6916 |
| Week 1 | Group*SampleName | Control | Asparagus chopped | Control | Beef puree | 16.875 | 12.1236 | 352 | 1.39 | 0.1648 |
| Week 1 | Group*SampleName | Control | Asparagus chopped | Control | Black bean puree | 1.125 | 12.1236 | 352 | 0.09 | 0.9261 |
| Week 1 | Group*SampleName | Control | Asparagus chopped | Control | Broccoli chopped | -11.75 | 12.1236 | 352 | -0.97 | 0.3331 |
| Week 1 | Group*SampleName | Control | Asparagus chopped | Control | Broccoli puree | 8.625 | 12.1236 | 352 | 0.71 | 0.4773 |
| Week 1 | Group*SampleName | Control | Asparagus chopped | Control | Chicken puree | 13.3125 | 12.1236 | 352 | 1.1 | 0.2729 |
| Week 1 | Group*SampleName | Control | Asparagus chopped | Control | Kale chopped | 33.375 | 12.1236 | 352 | 2.75 | 0.0062 |
| Week 1 | Group*SampleName | Control | Asparagus chopped | Control | Kale puree | 32.5625 | 12.1236 | 352 | 2.69 | 0.0076 |
| Week 1 | Group*SampleName | Control | Asparagus chopped | Control | Oat puree | 25.6875 | 12.1236 | 352 | 2.12 | 0.0348 |
| Week 1 | Group*SampleName | Control | Asparagus chopped | Control | Spinach chopped | 21.125 | 12.1236 | 352 | 1.74 | 0.0823 |
| Week 1 | Group*SampleName | Control | Asparagus chopped | Control | Spinach puree | 23.25 | 12.1236 | 352 | 1.92 | 0.056 |
| Week 1 | Group*SampleName | Control | Asparagus puree | Control | Beef puree | 21.6875 | 12.1236 | 352 | 1.79 | 0.0745 |
| Week 1 | Group*SampleName | Control | Asparagus puree | Control | Black bean puree | 5.9375 | 12.1236 | 352 | 0.49 | 0.6246 |
| Week 1 | Group*SampleName | Control | Asparagus puree | Control | Broccoli chopped | -6.9375 | 12.1236 | 352 | -0.57 | 0.5675 |
| Week 1 | Group*SampleName | Control | Asparagus puree | Control | Broccoli puree | 13.4375 | 12.1236 | 352 | 1.11 | 0.2685 |
| Week 1 | Group*SampleName | Control | Asparagus puree | Control | Chicken puree | 18.125 | 12.1236 | 352 | 1.5 | 0.1358 |
| Week 1 | Group*SampleName | Control | Asparagus puree | Control | Kale chopped | 38.1875 | 12.1236 | 352 | 3.15 | 0.0018 |
| Week 1 | Group*SampleName | Control | Asparagus puree | Control | Kale puree | 37.375 | 12.1236 | 352 | 3.08 | 0.0022 |
| Week 1 | Group*SampleName | Control | Asparagus puree | Control | Oat puree | 30.5 | 12.1236 | 352 | 2.52 | 0.0123 |
| Week 1 | Group*SampleName | Control | Asparagus puree | Control | Spinach chopped | 25.9375 | 12.1236 | 352 | 2.14 | 0.0331 |
| Week 1 | Group*SampleName | Control | Asparagus puree | Control | Spinach puree | 28.0625 | 12.1236 | 352 | 2.31 | 0.0212 |
| Week 1 | Group*SampleName | Control | Beef puree | Control | Black bean puree | -15.75 | 12.1236 | 352 | -1.3 | 0.1948 |
| Week 1 | Group*SampleName | Control | Beef puree | Control | Broccoli chopped | -28.625 | 12.1236 | 352 | -2.36 | 0.0188 |
| Week 1 | Group*SampleName | Control | Beef puree | Control | Broccoli puree | -8.25 | 12.1236 | 352 | -0.68 | 0.4966 |
| Week 1 | Group*SampleName | Control | Beef puree | Control | Chicken puree | -3.5625 | 12.1236 | 352 | -0.29 | 0.769 |
| Week 1 | Group*SampleName | Control | Beef puree | Control | Kale chopped | 16.5 | 12.1236 | 352 | 1.36 | 0.1744 |
| Week 1 | Group*SampleName | Control | Beef puree | Control | Kale puree | 15.6875 | 12.1236 | 352 | 1.29 | 0.1965 |
| Week 1 | Group*SampleName | Control | Beef puree | Control | Oat puree | 8.8125 | 12.1236 | 352 | 0.73 | 0.4678 |
| Week 1 | Group*SampleName | Control | Beef puree | Control | Spinach chopped | 4.25 | 12.1236 | 352 | 0.35 | 0.7261 |
| Week 1 | Group*SampleName | Control | Beef puree | Control | Spinach puree | 6.375 | 12.1236 | 352 | 0.53 | 0.5993 |
| Week 1 | Group*SampleName | Control | Black bean puree | Control | Broccoli chopped | -12.875 | 12.1236 | 352 | -1.06 | 0.289 |
| Week 1 | Group*SampleName | Control | Black bean puree | Control | Broccoli puree | 7.5 | 12.1236 | 352 | 0.62 | 0.5366 |
| Week 1 | Group*SampleName | Control | Black bean puree | Control | Chicken puree | 12.1875 | 12.1236 | 352 | 1.01 | 0.3155 |
| Week 1 | Group*SampleName | Control | Black bean puree | Control | Kale chopped | 32.25 | 12.1236 | 352 | 2.66 | 0.0082 |
| Week 1 | Group*SampleName | Control | Black bean puree | Control | Kale puree | 31.4375 | 12.1236 | 352 | 2.59 | 0.0099 |
| Week 1 | Group*SampleName | Control | Black bean puree | Control | Oat puree | 24.5625 | 12.1236 | 352 | 2.03 | 0.0435 |
| Week 1 | Group*SampleName | Control | Black bean puree | Control | Spinach chopped | 20 | 12.1236 | 352 | 1.65 | 0.0999 |
| Week 1 | Group*SampleName | Control | Black bean puree | Control | Spinach puree | 22.125 | 12.1236 | 352 | 1.82 | 0.0689 |
| Week 1 | Group*SampleName | Control | Broccoli chopped | Control | Broccoli puree | 20.375 | 12.1236 | 352 | 1.68 | 0.0937 |
| Week 1 | Group*SampleName | Control | Broccoli chopped | Control | Chicken puree | 25.0625 | 12.1236 | 352 | 2.07 | 0.0394 |
| Week 1 | Group*SampleName | Control | Broccoli chopped | Control | Kale chopped | 45.125 | 12.1236 | 352 | 3.72 | 0.0002 |
| Week 1 | Group*SampleName | Control | Broccoli chopped | Control | Kale puree | 44.3125 | 12.1236 | 352 | 3.66 | 0.0003 |
| Week 1 | Group*SampleName | Control | Broccoli chopped | Control | Oat puree | 37.4375 | 12.1236 | 352 | 3.09 | 0.0022 |
| Week 1 | Group*SampleName | Control | Broccoli chopped | Control | Spinach chopped | 32.875 | 12.1236 | 352 | 2.71 | 0.007 |
| Week 1 | Group*SampleName | Control | Broccoli chopped | Control | Spinach puree | 35 | 12.1236 | 352 | 2.89 | 0.0041 |
| Week 1 | Group*SampleName | Control | Broccoli puree | Control | Chicken puree | 4.6875 | 12.1236 | 352 | 0.39 | 0.6993 |
| Week 1 | Group*SampleName | Control | Broccoli puree | Control | Kale chopped | 24.75 | 12.1236 | 352 | 2.04 | 0.0419 |
| Week 1 | Group*SampleName | Control | Broccoli puree | Control | Kale puree | 23.9375 | 12.1236 | 352 | 1.97 | 0.0491 |
| Week 1 | Group*SampleName | Control | Broccoli puree | Control | Oat puree | 17.0625 | 12.1236 | 352 | 1.41 | 0.1602 |
| Week 1 | Group*SampleName | Control | Broccoli puree | Control | Spinach chopped | 12.5 | 12.1236 | 352 | 1.03 | 0.3032 |
| Week 1 | Group*SampleName | Control | Broccoli puree | Control | Spinach puree | 14.625 | 12.1236 | 352 | 1.21 | 0.2285 |
| Week 1 | Group*SampleName | Control | Chicken puree | Control | Kale chopped | 20.0625 | 12.1236 | 352 | 1.65 | 0.0989 |
| Week 1 | Group*SampleName | Control | Chicken puree | Control | Kale puree | 19.25 | 12.1236 | 352 | 1.59 | 0.1132 |
| Week 1 | Group*SampleName | Control | Chicken puree | Control | Oat puree | 12.375 | 12.1236 | 352 | 1.02 | 0.3081 |
| Week 1 | Group*SampleName | Control | Chicken puree | Control | Spinach chopped | 7.8125 | 12.1236 | 352 | 0.64 | 0.5197 |
| Week 1 | Group*SampleName | Control | Chicken puree | Control | Spinach puree | 9.9375 | 12.1236 | 352 | 0.82 | 0.413 |
| Week 1 | Group*SampleName | Control | Kale chopped | Control | Kale puree | -0.8125 | 12.1236 | 352 | -0.07 | 0.9466 |
| Week 1 | Group*SampleName | Control | Kale chopped | Control | Oat puree | -7.6875 | 12.1236 | 352 | -0.63 | 0.5264 |
| Week 1 | Group*SampleName | Control | Kale chopped | Control | Spinach chopped | -12.25 | 12.1236 | 352 | -1.01 | 0.313 |
| Week 1 | Group*SampleName | Control | Kale chopped | Control | Spinach puree | -10.125 | 12.1236 | 352 | -0.84 | 0.4042 |
| Week 1 | Group*SampleName | Control | Kale puree | Control | Oat puree | -6.875 | 12.1236 | 352 | -0.57 | 0.571 |
| Week 1 | Group*SampleName | Control | Kale puree | Control | Spinach chopped | -11.4375 | 12.1236 | 352 | -0.94 | 0.3461 |
| Week 1 | Group*SampleName | Control | Kale puree | Control | Spinach puree | -9.3125 | 12.1236 | 352 | -0.77 | 0.4429 |
| Week 1 | Group*SampleName | Control | Oat puree | Control | Spinach chopped | -4.5625 | 12.1236 | 352 | -0.38 | 0.7069 |
| Week 1 | Group*SampleName | Control | Oat puree | Control | Spinach puree | -2.4375 | 12.1236 | 352 | -0.2 | 0.8408 |
| Week 1 | Group*SampleName | Control | Spinach chopped | Control | Spinach puree | 2.125 | 12.1236 | 352 | 0.18 | 0.861 |
| Week 2 | Group | Vegetable |  | Control |  | 0.7829 | 9.3226 | 31 | 0.08 | 0.9336 |
| Week 2 | SampleName |  | Asparagus chopped |  | Asparagus puree | -0.8819 | 7.9526 | 352 | -0.11 | 0.9118 |
| Week 2 | SampleName |  | Asparagus chopped |  | Beef puree | 13.9306 | 7.9526 | 352 | 1.75 | 0.0807 |
| Week 2 | SampleName |  | Asparagus chopped |  | Black bean puree | 3.7951 | 7.9526 | 352 | 0.48 | 0.6335 |
| Week 2 | SampleName |  | Asparagus chopped |  | Broccoli chopped | -9.4097 | 7.9526 | 352 | -1.18 | 0.2375 |
| Week 2 | SampleName |  | Asparagus chopped |  | Broccoli puree | -8.816 | 7.9526 | 352 | -1.11 | 0.2684 |
| Week 2 | SampleName |  | Asparagus chopped |  | Chicken puree | 14.316 | 7.9526 | 352 | 1.8 | 0.0727 |
| Week 2 | SampleName |  | Asparagus chopped |  | Kale chopped | 23.6146 | 7.9526 | 352 | 2.97 | 0.0032 |
| Week 2 | SampleName |  | Asparagus chopped |  | Kale puree | 21.9097 | 7.9526 | 352 | 2.76 | 0.0062 |
| Week 2 | SampleName |  | Asparagus chopped |  | Oat puree | 3.8889 | 7.9526 | 352 | 0.49 | 0.6251 |
| Week 2 | SampleName |  | Asparagus chopped |  | Spinach chopped | 5.2882 | 7.9526 | 352 | 0.66 | 0.5065 |
| Week 2 | SampleName |  | Asparagus chopped |  | Spinach puree | 17.0104 | 7.9526 | 352 | 2.14 | 0.0331 |
| Week 2 | SampleName |  | Asparagus puree |  | Beef puree | 14.8125 | 7.9526 | 352 | 1.86 | 0.0634 |
| Week 2 | SampleName |  | Asparagus puree |  | Black bean puree | 4.6771 | 7.9526 | 352 | 0.59 | 0.5568 |
| Week 2 | SampleName |  | Asparagus puree |  | Broccoli chopped | -8.5278 | 7.9526 | 352 | -1.07 | 0.2843 |
| Week 2 | SampleName |  | Asparagus puree |  | Broccoli puree | -7.934 | 7.9526 | 352 | -1 | 0.3191 |
| Week 2 | SampleName |  | Asparagus puree |  | Chicken puree | 15.1979 | 7.9526 | 352 | 1.91 | 0.0568 |
| Week 2 | SampleName |  | Asparagus puree |  | Kale chopped | 24.4965 | 7.9526 | 352 | 3.08 | 0.0022 |
| Week 2 | SampleName |  | Asparagus puree |  | Kale puree | 22.7917 | 7.9526 | 352 | 2.87 | 0.0044 |
| Week 2 | SampleName |  | Asparagus puree |  | Oat puree | 4.7708 | 7.9526 | 352 | 0.6 | 0.549 |
| Week 2 | SampleName |  | Asparagus puree |  | Spinach chopped | 6.1701 | 7.9526 | 352 | 0.78 | 0.4383 |
| Week 2 | SampleName |  | Asparagus puree |  | Spinach puree | 17.8924 | 7.9526 | 352 | 2.25 | 0.0251 |
| Week 2 | SampleName |  | Beef puree |  | Black bean puree | -10.1354 | 7.9526 | 352 | -1.27 | 0.2033 |
| Week 2 | SampleName |  | Beef puree |  | Broccoli chopped | -23.3403 | 7.9526 | 352 | -2.93 | 0.0036 |
| Week 2 | SampleName |  | Beef puree |  | Broccoli puree | -22.7465 | 7.9526 | 352 | -2.86 | 0.0045 |
| Week 2 | SampleName |  | Beef puree |  | Chicken puree | 0.3854 | 7.9526 | 352 | 0.05 | 0.9614 |
| Week 2 | SampleName |  | Beef puree |  | Kale chopped | 9.684 | 7.9526 | 352 | 1.22 | 0.2241 |
| Week 2 | SampleName |  | Beef puree |  | Kale puree | 7.9792 | 7.9526 | 352 | 1 | 0.3164 |
| Week 2 | SampleName |  | Beef puree |  | Oat puree | -10.0417 | 7.9526 | 352 | -1.26 | 0.2075 |
| Week 2 | SampleName |  | Beef puree |  | Spinach chopped | -8.6424 | 7.9526 | 352 | -1.09 | 0.2779 |
| Week 2 | SampleName |  | Beef puree |  | Spinach puree | 3.0799 | 7.9526 | 352 | 0.39 | 0.6988 |
| Week 2 | SampleName |  | Black bean puree |  | Broccoli chopped | -13.2049 | 7.9526 | 352 | -1.66 | 0.0977 |
| Week 2 | SampleName |  | Black bean puree |  | Broccoli puree | -12.6111 | 7.9526 | 352 | -1.59 | 0.1137 |
| Week 2 | SampleName |  | Black bean puree |  | Chicken puree | 10.5208 | 7.9526 | 352 | 1.32 | 0.1867 |
| Week 2 | SampleName |  | Black bean puree |  | Kale chopped | 19.8194 | 7.9526 | 352 | 2.49 | 0.0132 |
| Week 2 | SampleName |  | Black bean puree |  | Kale puree | 18.1146 | 7.9526 | 352 | 2.28 | 0.0233 |
| Week 2 | SampleName |  | Black bean puree |  | Oat puree | 0.09375 | 7.9526 | 352 | 0.01 | 0.9906 |
| Week 2 | SampleName |  | Black bean puree |  | Spinach chopped | 1.4931 | 7.9526 | 352 | 0.19 | 0.8512 |
| Week 2 | SampleName |  | Black bean puree |  | Spinach puree | 13.2153 | 7.9526 | 352 | 1.66 | 0.0975 |
| Week 2 | SampleName |  | Broccoli chopped |  | Broccoli puree | 0.5937 | 7.9526 | 352 | 0.07 | 0.9405 |
| Week 2 | SampleName |  | Broccoli chopped |  | Chicken puree | 23.7257 | 7.9526 | 352 | 2.98 | 0.003 |
| Week 2 | SampleName |  | Broccoli chopped |  | Kale chopped | 33.0243 | 7.9526 | 352 | 4.15 | <.0001 |
| Week 2 | SampleName |  | Broccoli chopped |  | Kale puree | 31.3194 | 7.9526 | 352 | 3.94 | <.0001 |
| Week 2 | SampleName |  | Broccoli chopped |  | Oat puree | 13.2986 | 7.9526 | 352 | 1.67 | 0.0954 |
| Week 2 | SampleName |  | Broccoli chopped |  | Spinach chopped | 14.6979 | 7.9526 | 352 | 1.85 | 0.0654 |
| Week 2 | SampleName |  | Broccoli chopped |  | Spinach puree | 26.4201 | 7.9526 | 352 | 3.32 | 0.001 |
| Week 2 | SampleName |  | Broccoli puree |  | Chicken puree | 23.1319 | 7.9526 | 352 | 2.91 | 0.0039 |
| Week 2 | SampleName |  | Broccoli puree |  | Kale chopped | 32.4306 | 7.9526 | 352 | 4.08 | <.0001 |
| Week 2 | SampleName |  | Broccoli puree |  | Kale puree | 30.7257 | 7.9526 | 352 | 3.86 | 0.0001 |
| Week 2 | SampleName |  | Broccoli puree |  | Oat puree | 12.7049 | 7.9526 | 352 | 1.6 | 0.111 |
| Week 2 | SampleName |  | Broccoli puree |  | Spinach chopped | 14.1042 | 7.9526 | 352 | 1.77 | 0.077 |
| Week 2 | SampleName |  | Broccoli puree |  | Spinach puree | 25.8264 | 7.9526 | 352 | 3.25 | 0.0013 |
| Week 2 | SampleName |  | Chicken puree |  | Kale chopped | 9.2986 | 7.9526 | 352 | 1.17 | 0.2431 |
| Week 2 | SampleName |  | Chicken puree |  | Kale puree | 7.5938 | 7.9526 | 352 | 0.95 | 0.3403 |
| Week 2 | SampleName |  | Chicken puree |  | Oat puree | -10.4271 | 7.9526 | 352 | -1.31 | 0.1907 |
| Week 2 | SampleName |  | Chicken puree |  | Spinach chopped | -9.0278 | 7.9526 | 352 | -1.14 | 0.2571 |
| Week 2 | SampleName |  | Chicken puree |  | Spinach puree | 2.6944 | 7.9526 | 352 | 0.34 | 0.735 |
| Week 2 | SampleName |  | Kale chopped |  | Kale puree | -1.7049 | 7.9526 | 352 | -0.21 | 0.8304 |
| Week 2 | SampleName |  | Kale chopped |  | Oat puree | -19.7257 | 7.9526 | 352 | -2.48 | 0.0136 |
| Week 2 | SampleName |  | Kale chopped |  | Spinach chopped | -18.3264 | 7.9526 | 352 | -2.3 | 0.0218 |
| Week 2 | SampleName |  | Kale chopped |  | Spinach puree | -6.6042 | 7.9526 | 352 | -0.83 | 0.4069 |
| Week 2 | SampleName |  | Kale puree |  | Oat puree | -18.0208 | 7.9526 | 352 | -2.27 | 0.0241 |
| Week 2 | SampleName |  | Kale puree |  | Spinach chopped | -16.6215 | 7.9526 | 352 | -2.09 | 0.0373 |
| Week 2 | SampleName |  | Kale puree |  | Spinach puree | -4.8993 | 7.9526 | 352 | -0.62 | 0.5382 |
| Week 2 | SampleName |  | Oat puree |  | Spinach chopped | 1.3993 | 7.9526 | 352 | 0.18 | 0.8604 |
| Week 2 | SampleName |  | Oat puree |  | Spinach puree | 13.1215 | 7.9526 | 352 | 1.65 | 0.0998 |
| Week 2 | SampleName |  | Spinach chopped |  | Spinach puree | 11.7222 | 7.9526 | 352 | 1.47 | 0.1414 |
| Week 2 | Group*SampleName | Vegetable | Asparagus chopped | Vegetable | Asparagus puree | -3.8889 | 10.9108 | 352 | -0.36 | 0.7217 |
| Week 2 | Group*SampleName | Vegetable | Asparagus chopped | Vegetable | Beef puree | 14.6111 | 10.9108 | 352 | 1.34 | 0.1814 |
| Week 2 | Group*SampleName | Vegetable | Asparagus chopped | Vegetable | Black bean puree | 3.2778 | 10.9108 | 352 | 0.3 | 0.764 |
| Week 2 | Group*SampleName | Vegetable | Asparagus chopped | Vegetable | Broccoli chopped | -12.9444 | 10.9108 | 352 | -1.19 | 0.2363 |
| Week 2 | Group*SampleName | Vegetable | Asparagus chopped | Vegetable | Broccoli puree | -18.9444 | 10.9108 | 352 | -1.74 | 0.0834 |
| Week 2 | Group*SampleName | Vegetable | Asparagus chopped | Vegetable | Chicken puree | 14.4444 | 10.9108 | 352 | 1.32 | 0.1864 |
| Week 2 | Group*SampleName | Vegetable | Asparagus chopped | Vegetable | Kale chopped | 10.6667 | 10.9108 | 352 | 0.98 | 0.3289 |
| Week 2 | Group*SampleName | Vegetable | Asparagus chopped | Vegetable | Kale puree | 5.9444 | 10.9108 | 352 | 0.54 | 0.5862 |
| Week 2 | Group*SampleName | Vegetable | Asparagus chopped | Vegetable | Oat puree | -13.7222 | 10.9108 | 352 | -1.26 | 0.2093 |
| Week 2 | Group*SampleName | Vegetable | Asparagus chopped | Vegetable | Spinach chopped | -7.6111 | 10.9108 | 352 | -0.7 | 0.4859 |
| Week 2 | Group*SampleName | Vegetable | Asparagus chopped | Vegetable | Spinach puree | 5.8333 | 10.9108 | 352 | 0.53 | 0.5932 |
| Week 2 | Group*SampleName | Vegetable | Asparagus chopped | Control | Asparagus chopped | -13.7136 | 14.2428 | 146 | -0.96 | 0.3372 |
| Week 2 | Group*SampleName | Vegetable | Asparagus puree | Vegetable | Beef puree | 18.5 | 10.9108 | 352 | 1.7 | 0.0909 |
| Week 2 | Group*SampleName | Vegetable | Asparagus puree | Vegetable | Black bean puree | 7.1667 | 10.9108 | 352 | 0.66 | 0.5117 |
| Week 2 | Group*SampleName | Vegetable | Asparagus puree | Vegetable | Broccoli chopped | -9.0556 | 10.9108 | 352 | -0.83 | 0.4071 |
| Week 2 | Group*SampleName | Vegetable | Asparagus puree | Vegetable | Broccoli puree | -15.0556 | 10.9108 | 352 | -1.38 | 0.1685 |
| Week 2 | Group*SampleName | Vegetable | Asparagus puree | Vegetable | Chicken puree | 18.3333 | 10.9108 | 352 | 1.68 | 0.0938 |
| Week 2 | Group*SampleName | Vegetable | Asparagus puree | Vegetable | Kale chopped | 14.5556 | 10.9108 | 352 | 1.33 | 0.1831 |
| Week 2 | Group*SampleName | Vegetable | Asparagus puree | Vegetable | Kale puree | 9.8333 | 10.9108 | 352 | 0.9 | 0.3681 |
| Week 2 | Group*SampleName | Vegetable | Asparagus puree | Vegetable | Oat puree | -9.8333 | 10.9108 | 352 | -0.9 | 0.3681 |
| Week 2 | Group*SampleName | Vegetable | Asparagus puree | Vegetable | Spinach chopped | -3.7222 | 10.9108 | 352 | -0.34 | 0.7332 |
| Week 2 | Group*SampleName | Vegetable | Asparagus puree | Vegetable | Spinach puree | 9.7222 | 10.9108 | 352 | 0.89 | 0.3735 |
| Week 2 | Group*SampleName | Vegetable | Asparagus puree | Control | Asparagus puree | -7.6997 | 14.2428 | 146 | -0.54 | 0.5896 |
| Week 2 | Group*SampleName | Vegetable | Beef puree | Vegetable | Black bean puree | -11.3333 | 10.9108 | 352 | -1.04 | 0.2996 |
| Week 2 | Group*SampleName | Vegetable | Beef puree | Vegetable | Broccoli chopped | -27.5556 | 10.9108 | 352 | -2.53 | 0.012 |
| Week 2 | Group*SampleName | Vegetable | Beef puree | Vegetable | Broccoli puree | -33.5556 | 10.9108 | 352 | -3.08 | 0.0023 |
| Week 2 | Group*SampleName | Vegetable | Beef puree | Vegetable | Chicken puree | -0.1667 | 10.9108 | 352 | -0.02 | 0.9878 |
| Week 2 | Group*SampleName | Vegetable | Beef puree | Vegetable | Kale chopped | -3.9444 | 10.9108 | 352 | -0.36 | 0.7179 |
| Week 2 | Group*SampleName | Vegetable | Beef puree | Vegetable | Kale puree | -8.6667 | 10.9108 | 352 | -0.79 | 0.4275 |
| Week 2 | Group*SampleName | Vegetable | Beef puree | Vegetable | Oat puree | -28.3333 | 10.9108 | 352 | -2.6 | 0.0098 |
| Week 2 | Group*SampleName | Vegetable | Beef puree | Vegetable | Spinach chopped | -22.2222 | 10.9108 | 352 | -2.04 | 0.0424 |
| Week 2 | Group*SampleName | Vegetable | Beef puree | Vegetable | Spinach puree | -8.7778 | 10.9108 | 352 | -0.8 | 0.4217 |
| Week 2 | Group*SampleName | Vegetable | Beef puree | Control | Beef puree | -15.0747 | 14.2428 | 146 | -1.06 | 0.2916 |
| Week 2 | Group*SampleName | Vegetable | Black bean puree | Vegetable | Broccoli chopped | -16.2222 | 10.9108 | 352 | -1.49 | 0.138 |
| Week 2 | Group*SampleName | Vegetable | Black bean puree | Vegetable | Broccoli puree | -22.2222 | 10.9108 | 352 | -2.04 | 0.0424 |
| Week 2 | Group*SampleName | Vegetable | Black bean puree | Vegetable | Chicken puree | 11.1667 | 10.9108 | 352 | 1.02 | 0.3068 |
| Week 2 | Group*SampleName | Vegetable | Black bean puree | Vegetable | Kale chopped | 7.3889 | 10.9108 | 352 | 0.68 | 0.4987 |
| Week 2 | Group*SampleName | Vegetable | Black bean puree | Vegetable | Kale puree | 2.6667 | 10.9108 | 352 | 0.24 | 0.8071 |
| Week 2 | Group*SampleName | Vegetable | Black bean puree | Vegetable | Oat puree | -17 | 10.9108 | 352 | -1.56 | 0.1201 |
| Week 2 | Group*SampleName | Vegetable | Black bean puree | Vegetable | Spinach chopped | -10.8889 | 10.9108 | 352 | -1 | 0.319 |
| Week 2 | Group*SampleName | Vegetable | Black bean puree | Vegetable | Spinach puree | 2.5556 | 10.9108 | 352 | 0.23 | 0.8149 |
| Week 2 | Group*SampleName | Vegetable | Black bean puree | Control | Black bean puree | -12.6789 | 14.2428 | 146 | -0.89 | 0.3748 |
| Week 2 | Group*SampleName | Vegetable | Broccoli chopped | Vegetable | Broccoli puree | -6 | 10.9108 | 352 | -0.55 | 0.5827 |
| Week 2 | Group*SampleName | Vegetable | Broccoli chopped | Vegetable | Chicken puree | 27.3889 | 10.9108 | 352 | 2.51 | 0.0125 |
| Week 2 | Group*SampleName | Vegetable | Broccoli chopped | Vegetable | Kale chopped | 23.6111 | 10.9108 | 352 | 2.16 | 0.0311 |
| Week 2 | Group*SampleName | Vegetable | Broccoli chopped | Vegetable | Kale puree | 18.8889 | 10.9108 | 352 | 1.73 | 0.0843 |
| Week 2 | Group*SampleName | Vegetable | Broccoli chopped | Vegetable | Oat puree | -0.7778 | 10.9108 | 352 | -0.07 | 0.9432 |
| Week 2 | Group*SampleName | Vegetable | Broccoli chopped | Vegetable | Spinach chopped | 5.3333 | 10.9108 | 352 | 0.49 | 0.6253 |
| Week 2 | Group*SampleName | Vegetable | Broccoli chopped | Vegetable | Spinach puree | 18.7778 | 10.9108 | 352 | 1.72 | 0.0861 |
| Week 2 | Group*SampleName | Vegetable | Broccoli chopped | Control | Broccoli chopped | -6.6442 | 14.2428 | 146 | -0.47 | 0.6416 |
| Week 2 | Group*SampleName | Vegetable | Broccoli puree | Vegetable | Chicken puree | 33.3889 | 10.9108 | 352 | 3.06 | 0.0024 |
| Week 2 | Group*SampleName | Vegetable | Broccoli puree | Vegetable | Kale chopped | 29.6111 | 10.9108 | 352 | 2.71 | 0.007 |
| Week 2 | Group*SampleName | Vegetable | Broccoli puree | Vegetable | Kale puree | 24.8889 | 10.9108 | 352 | 2.28 | 0.0231 |
| Week 2 | Group*SampleName | Vegetable | Broccoli puree | Vegetable | Oat puree | 5.2222 | 10.9108 | 352 | 0.48 | 0.6325 |
| Week 2 | Group*SampleName | Vegetable | Broccoli puree | Vegetable | Spinach chopped | 11.3333 | 10.9108 | 352 | 1.04 | 0.2996 |
| Week 2 | Group*SampleName | Vegetable | Broccoli puree | Vegetable | Spinach puree | 24.7778 | 10.9108 | 352 | 2.27 | 0.0238 |
| Week 2 | Group*SampleName | Vegetable | Broccoli puree | Control | Broccoli puree | 6.5433 | 14.2428 | 146 | 0.46 | 0.6466 |
| Week 2 | Group*SampleName | Vegetable | Chicken puree | Vegetable | Kale chopped | -3.7778 | 10.9108 | 352 | -0.35 | 0.7294 |
| Week 2 | Group*SampleName | Vegetable | Chicken puree | Vegetable | Kale puree | -8.5 | 10.9108 | 352 | -0.78 | 0.4365 |
| Week 2 | Group*SampleName | Vegetable | Chicken puree | Vegetable | Oat puree | -28.1667 | 10.9108 | 352 | -2.58 | 0.0102 |
| Week 2 | Group*SampleName | Vegetable | Chicken puree | Vegetable | Spinach chopped | -22.0556 | 10.9108 | 352 | -2.02 | 0.044 |
| Week 2 | Group*SampleName | Vegetable | Chicken puree | Vegetable | Spinach puree | -8.6111 | 10.9108 | 352 | -0.79 | 0.4305 |
| Week 2 | Group*SampleName | Vegetable | Chicken puree | Control | Chicken puree | -13.9706 | 14.2428 | 146 | -0.98 | 0.3283 |
| Week 2 | Group*SampleName | Vegetable | Kale chopped | Vegetable | Kale puree | -4.7222 | 10.9108 | 352 | -0.43 | 0.6654 |
| Week 2 | Group*SampleName | Vegetable | Kale chopped | Vegetable | Oat puree | -24.3889 | 10.9108 | 352 | -2.24 | 0.026 |
| Week 2 | Group*SampleName | Vegetable | Kale chopped | Vegetable | Spinach chopped | -18.2778 | 10.9108 | 352 | -1.68 | 0.0948 |
| Week 2 | Group*SampleName | Vegetable | Kale chopped | Vegetable | Spinach puree | -4.8333 | 10.9108 | 352 | -0.44 | 0.658 |
| Week 2 | Group*SampleName | Vegetable | Kale chopped | Control | Kale chopped | 12.1822 | 14.2428 | 146 | 0.86 | 0.3938 |
| Week 2 | Group*SampleName | Vegetable | Kale puree | Vegetable | Oat puree | -19.6667 | 10.9108 | 352 | -1.8 | 0.0723 |
| Week 2 | Group*SampleName | Vegetable | Kale puree | Vegetable | Spinach chopped | -13.5556 | 10.9108 | 352 | -1.24 | 0.2149 |
| Week 2 | Group*SampleName | Vegetable | Kale puree | Vegetable | Spinach puree | -0.1111 | 10.9108 | 352 | -0.01 | 0.9919 |
| Week 2 | Group*SampleName | Vegetable | Kale puree | Control | Kale puree | 18.2169 | 14.2428 | 146 | 1.28 | 0.2029 |
| Week 2 | Group*SampleName | Vegetable | Oat puree | Vegetable | Spinach chopped | 6.1111 | 10.9108 | 352 | 0.56 | 0.5758 |
| Week 2 | Group*SampleName | Vegetable | Oat puree | Vegetable | Spinach puree | 19.5556 | 10.9108 | 352 | 1.79 | 0.0739 |
| Week 2 | Group*SampleName | Vegetable | Oat puree | Control | Oat puree | 21.5086 | 14.2428 | 146 | 1.51 | 0.1332 |
| Week 2 | Group*SampleName | Vegetable | Spinach chopped | Vegetable | Spinach puree | 13.4444 | 10.9108 | 352 | 1.23 | 0.2187 |
| Week 2 | Group*SampleName | Vegetable | Spinach chopped | Control | Spinach chopped | 12.085 | 14.2428 | 146 | 0.85 | 0.3976 |
| Week 2 | Group*SampleName | Vegetable | Spinach puree | Control | Spinach puree | 8.6405 | 14.2428 | 146 | 0.61 | 0.545 |
| Week 2 | Group*SampleName | Control | Asparagus chopped | Control | Asparagus puree | 2.125 | 11.5727 | 352 | 0.18 | 0.8544 |
| Week 2 | Group*SampleName | Control | Asparagus chopped | Control | Beef puree | 13.25 | 11.5727 | 352 | 1.14 | 0.253 |
| Week 2 | Group*SampleName | Control | Asparagus chopped | Control | Black bean puree | 4.3125 | 11.5727 | 352 | 0.37 | 0.7096 |
| Week 2 | Group*SampleName | Control | Asparagus chopped | Control | Broccoli chopped | -5.875 | 11.5727 | 352 | -0.51 | 0.612 |
| Week 2 | Group*SampleName | Control | Asparagus chopped | Control | Broccoli puree | 1.3125 | 11.5727 | 352 | 0.11 | 0.9098 |
| Week 2 | Group*SampleName | Control | Asparagus chopped | Control | Chicken puree | 14.1875 | 11.5727 | 352 | 1.23 | 0.221 |
| Week 2 | Group*SampleName | Control | Asparagus chopped | Control | Kale chopped | 36.5625 | 11.5727 | 352 | 3.16 | 0.0017 |
| Week 2 | Group*SampleName | Control | Asparagus chopped | Control | Kale puree | 37.875 | 11.5727 | 352 | 3.27 | 0.0012 |
| Week 2 | Group*SampleName | Control | Asparagus chopped | Control | Oat puree | 21.5 | 11.5727 | 352 | 1.86 | 0.064 |
| Week 2 | Group*SampleName | Control | Asparagus chopped | Control | Spinach chopped | 18.1875 | 11.5727 | 352 | 1.57 | 0.1169 |
| Week 2 | Group*SampleName | Control | Asparagus chopped | Control | Spinach puree | 28.1875 | 11.5727 | 352 | 2.44 | 0.0154 |
| Week 2 | Group*SampleName | Control | Asparagus puree | Control | Beef puree | 11.125 | 11.5727 | 352 | 0.96 | 0.3371 |
| Week 2 | Group*SampleName | Control | Asparagus puree | Control | Black bean puree | 2.1875 | 11.5727 | 352 | 0.19 | 0.8502 |
| Week 2 | Group*SampleName | Control | Asparagus puree | Control | Broccoli chopped | -8 | 11.5727 | 352 | -0.69 | 0.4898 |
| Week 2 | Group*SampleName | Control | Asparagus puree | Control | Broccoli puree | -0.8125 | 11.5727 | 352 | -0.07 | 0.9441 |
| Week 2 | Group*SampleName | Control | Asparagus puree | Control | Chicken puree | 12.0625 | 11.5727 | 352 | 1.04 | 0.298 |
| Week 2 | Group*SampleName | Control | Asparagus puree | Control | Kale chopped | 34.4375 | 11.5727 | 352 | 2.98 | 0.0031 |
| Week 2 | Group*SampleName | Control | Asparagus puree | Control | Kale puree | 35.75 | 11.5727 | 352 | 3.09 | 0.0022 |
| Week 2 | Group*SampleName | Control | Asparagus puree | Control | Oat puree | 19.375 | 11.5727 | 352 | 1.67 | 0.095 |
| Week 2 | Group*SampleName | Control | Asparagus puree | Control | Spinach chopped | 16.0625 | 11.5727 | 352 | 1.39 | 0.166 |
| Week 2 | Group*SampleName | Control | Asparagus puree | Control | Spinach puree | 26.0625 | 11.5727 | 352 | 2.25 | 0.0249 |
| Week 2 | Group*SampleName | Control | Beef puree | Control | Black bean puree | -8.9375 | 11.5727 | 352 | -0.77 | 0.4405 |
| Week 2 | Group*SampleName | Control | Beef puree | Control | Broccoli chopped | -19.125 | 11.5727 | 352 | -1.65 | 0.0993 |
| Week 2 | Group*SampleName | Control | Beef puree | Control | Broccoli puree | -11.9375 | 11.5727 | 352 | -1.03 | 0.303 |
| Week 2 | Group*SampleName | Control | Beef puree | Control | Chicken puree | 0.9375 | 11.5727 | 352 | 0.08 | 0.9355 |
| Week 2 | Group*SampleName | Control | Beef puree | Control | Kale chopped | 23.3125 | 11.5727 | 352 | 2.01 | 0.0447 |
| Week 2 | Group*SampleName | Control | Beef puree | Control | Kale puree | 24.625 | 11.5727 | 352 | 2.13 | 0.034 |
| Week 2 | Group*SampleName | Control | Beef puree | Control | Oat puree | 8.25 | 11.5727 | 352 | 0.71 | 0.4764 |
| Week 2 | Group*SampleName | Control | Beef puree | Control | Spinach chopped | 4.9375 | 11.5727 | 352 | 0.43 | 0.6699 |
| Week 2 | Group*SampleName | Control | Beef puree | Control | Spinach puree | 14.9375 | 11.5727 | 352 | 1.29 | 0.1976 |
| Week 2 | Group*SampleName | Control | Black bean puree | Control | Broccoli chopped | -10.1875 | 11.5727 | 352 | -0.88 | 0.3793 |
| Week 2 | Group*SampleName | Control | Black bean puree | Control | Broccoli puree | -3 | 11.5727 | 352 | -0.26 | 0.7956 |
| Week 2 | Group*SampleName | Control | Black bean puree | Control | Chicken puree | 9.875 | 11.5727 | 352 | 0.85 | 0.3941 |
| Week 2 | Group*SampleName | Control | Black bean puree | Control | Kale chopped | 32.25 | 11.5727 | 352 | 2.79 | 0.0056 |
| Week 2 | Group*SampleName | Control | Black bean puree | Control | Kale puree | 33.5625 | 11.5727 | 352 | 2.9 | 0.004 |
| Week 2 | Group*SampleName | Control | Black bean puree | Control | Oat puree | 17.1875 | 11.5727 | 352 | 1.49 | 0.1384 |
| Week 2 | Group*SampleName | Control | Black bean puree | Control | Spinach chopped | 13.875 | 11.5727 | 352 | 1.2 | 0.2314 |
| Week 2 | Group*SampleName | Control | Black bean puree | Control | Spinach puree | 23.875 | 11.5727 | 352 | 2.06 | 0.0398 |
| Week 2 | Group*SampleName | Control | Broccoli chopped | Control | Broccoli puree | 7.1875 | 11.5727 | 352 | 0.62 | 0.535 |
| Week 2 | Group*SampleName | Control | Broccoli chopped | Control | Chicken puree | 20.0625 | 11.5727 | 352 | 1.73 | 0.0839 |
| Week 2 | Group*SampleName | Control | Broccoli chopped | Control | Kale chopped | 42.4375 | 11.5727 | 352 | 3.67 | 0.0003 |
| Week 2 | Group*SampleName | Control | Broccoli chopped | Control | Kale puree | 43.75 | 11.5727 | 352 | 3.78 | 0.0002 |
| Week 2 | Group*SampleName | Control | Broccoli chopped | Control | Oat puree | 27.375 | 11.5727 | 352 | 2.37 | 0.0185 |
| Week 2 | Group*SampleName | Control | Broccoli chopped | Control | Spinach chopped | 24.0625 | 11.5727 | 352 | 2.08 | 0.0383 |
| Week 2 | Group*SampleName | Control | Broccoli chopped | Control | Spinach puree | 34.0625 | 11.5727 | 352 | 2.94 | 0.0035 |
| Week 2 | Group*SampleName | Control | Broccoli puree | Control | Chicken puree | 12.875 | 11.5727 | 352 | 1.11 | 0.2667 |
| Week 2 | Group*SampleName | Control | Broccoli puree | Control | Kale chopped | 35.25 | 11.5727 | 352 | 3.05 | 0.0025 |
| Week 2 | Group*SampleName | Control | Broccoli puree | Control | Kale puree | 36.5625 | 11.5727 | 352 | 3.16 | 0.0017 |
| Week 2 | Group*SampleName | Control | Broccoli puree | Control | Oat puree | 20.1875 | 11.5727 | 352 | 1.74 | 0.082 |
| Week 2 | Group*SampleName | Control | Broccoli puree | Control | Spinach chopped | 16.875 | 11.5727 | 352 | 1.46 | 0.1457 |
| Week 2 | Group*SampleName | Control | Broccoli puree | Control | Spinach puree | 26.875 | 11.5727 | 352 | 2.32 | 0.0208 |
| Week 2 | Group*SampleName | Control | Chicken puree | Control | Kale chopped | 22.375 | 11.5727 | 352 | 1.93 | 0.054 |
| Week 2 | Group*SampleName | Control | Chicken puree | Control | Kale puree | 23.6875 | 11.5727 | 352 | 2.05 | 0.0414 |
| Week 2 | Group*SampleName | Control | Chicken puree | Control | Oat puree | 7.3125 | 11.5727 | 352 | 0.63 | 0.5279 |
| Week 2 | Group*SampleName | Control | Chicken puree | Control | Spinach chopped | 4 | 11.5727 | 352 | 0.35 | 0.7298 |
| Week 2 | Group*SampleName | Control | Chicken puree | Control | Spinach puree | 14 | 11.5727 | 352 | 1.21 | 0.2272 |
| Week 2 | Group*SampleName | Control | Kale chopped | Control | Kale puree | 1.3125 | 11.5727 | 352 | 0.11 | 0.9098 |
| Week 2 | Group*SampleName | Control | Kale chopped | Control | Oat puree | -15.0625 | 11.5727 | 352 | -1.3 | 0.1939 |
| Week 2 | Group*SampleName | Control | Kale chopped | Control | Spinach chopped | -18.375 | 11.5727 | 352 | -1.59 | 0.1132 |
| Week 2 | Group*SampleName | Control | Kale chopped | Control | Spinach puree | -8.375 | 11.5727 | 352 | -0.72 | 0.4697 |
| Week 2 | Group*SampleName | Control | Kale puree | Control | Oat puree | -16.375 | 11.5727 | 352 | -1.41 | 0.158 |
| Week 2 | Group*SampleName | Control | Kale puree | Control | Spinach chopped | -19.6875 | 11.5727 | 352 | -1.7 | 0.0898 |
| Week 2 | Group*SampleName | Control | Kale puree | Control | Spinach puree | -9.6875 | 11.5727 | 352 | -0.84 | 0.4031 |
| Week 2 | Group*SampleName | Control | Oat puree | Control | Spinach chopped | -3.3125 | 11.5727 | 352 | -0.29 | 0.7749 |
| Week 2 | Group*SampleName | Control | Oat puree | Control | Spinach puree | 6.6875 | 11.5727 | 352 | 0.58 | 0.5637 |
| Week 2 | Group*SampleName | Control | Spinach chopped | Control | Spinach puree | 10 | 11.5727 | 352 | 0.86 | 0.3881 |

### Paired t-tests

Comparisons of sensory for Vegetables samples within group between Baseline and Week 1 or Week 2

| **Vegetable** | **Quality** | **Group** | **Baseline to Week 1, t-stat** | **Baseline to Week 1, p-value** | **Baseline to Week 2, t-stat** | **Baseline to Week 2, p-value** |
| --- | --- | --- | --- | --- | --- | --- |
| Broccoli puree | Liking | Vegetable | 0.709259 | 0.487778 | -0.042506 | 0.966590 |
| Broccoli puree | Liking | Control | 0.914019 | 0.375165 | -0.258954 | 0.799191 |
| Broccoli puree | Sweetness | Vegetable | 0.053747 | 0.957764 | 0.122818 | 0.903691 |
| Broccoli puree | Sweetness | Control | -0.083726 | 0.934382 | -0.863458 | 0.401482 |
| Broccoli puree | Bitterness | Vegetable | 0.598180 | 0.557606 | 0.176835 | 0.861728 |
| Broccoli puree | Bitterness | Control | 0.266078 | 0.793802 | 0.971099 | 0.346899 |
| Kale puree | Liking | Vegetable | -0.427440 | 0.674424 | -1.247173 | 0.229243 |
| Kale puree | Liking | Control | 2.502007 | 0.024409 | 2.533376 | 0.022941 |
| Kale puree | Sweetness | Vegetable | -0.615450 | 0.546409 | -0.624109 | 0.540842 |
| Kale puree | Sweetness | Control | 0.236011 | 0.816616 | 0.563876 | 0.581172 |
| Kale puree | Bitterness | Vegetable | 0.856699 | 0.403527 | -0.168914 | 0.867858 |
| Kale puree | Bitterness | Control | -0.902083 | 0.381270 | -0.991309 | 0.337257 |
| Asparagus puree | Liking | Vegetable | -0.246964 | 0.807894 | -0.824149 | 0.421268 |
| Asparagus puree | Liking | Control | -0.784556 | 0.444928 | 0.122420 | 0.904191 |
| Asparagus puree | Sweetness | Vegetable | 0.778377 | 0.447046 | -0.469399 | 0.644748 |
| Asparagus puree | Sweetness | Control | -1.493480 | 0.156047 | 1.123059 | 0.279067 |
| Asparagus puree | Bitterness | Vegetable | -0.357734 | 0.724945 | 0.100422 | 0.921184 |
| Asparagus puree | Bitterness | Control | -1.737864 | 0.102715 | -1.800410 | 0.091935 |
| Spinach puree | Liking | Vegetable | 0.483534 | 0.634886 | -0.108527 | 0.914849 |
| Spinach puree | Liking | Control | 0.706677 | 0.490600 | 1.826415 | 0.087756 |
| Spinach puree | Sweetness | Vegetable | -0.695022 | 0.496432 | -1.761561 | 0.096116 |
| Spinach puree | Sweetness | Control | 0.416828 | 0.682708 | 0.182271 | 0.857811 |
| Spinach puree | Bitterness | Vegetable | -0.103745 | 0.918586 | 0.127448 | 0.900081 |
| Spinach puree | Bitterness | Control | -2.427637 | 0.028254 | -0.249201 | 0.806585 |
| Chicken puree | Liking | Vegetable | -1.083689 | 0.293634 | -1.350282 | 0.194629 |
| Chicken puree | Liking | Control | -0.721164 | 0.481898 | -0.850928 | 0.408188 |
| Chicken puree | Sweetness | Vegetable | 0.605584 | 0.552791 | 0.353521 | 0.728044 |
| Chicken puree | Sweetness | Control | -1.964536 | 0.068272 | -1.413300 | 0.177983 |
| Chicken puree | Bitterness | Vegetable | -0.648057 | 0.525605 | -1.251253 | 0.227788 |
| Chicken puree | Bitterness | Control | -1.398084 | 0.182420 | 0.062844 | 0.950721 |
| Beef puree | Liking | Vegetable | 0.673667 | 0.509579 | -1.339864 | 0.197926 |
| Beef puree | Liking | Control | -0.450969 | 0.658463 | -0.883178 | 0.391075 |
| Beef puree | Sweetness | Vegetable | -0.533246 | 0.600766 | -1.627186 | 0.122089 |
| Beef puree | Sweetness | Control | -1.554057 | 0.141013 | -1.705742 | 0.108670 |
| Beef puree | Bitterness | Vegetable | -0.257590 | 0.799815 | -0.662886 | 0.516291 |
| Beef puree | Bitterness | Control | -1.424867 | 0.174670 | 1.315580 | 0.208069 |
| Black bean puree | Liking | Vegetable | -0.441392 | 0.664492 | -0.763309 | 0.455742 |
| Black bean puree | Liking | Control | -0.996570 | 0.334779 | -0.792487 | 0.440431 |
| Black bean puree | Sweetness | Vegetable | -0.447530 | 0.660143 | -0.536412 | 0.598624 |
| Black bean puree | Sweetness | Control | 0.738703 | 0.471489 | -1.364727 | 0.192462 |
| Black bean puree | Bitterness | Vegetable | -2.020564 | 0.059367 | 0.101339 | 0.920467 |
| Black bean puree | Bitterness | Control | -2.355979 | 0.032498 | -0.177795 | 0.861263 |
| Oat puree | Liking | Vegetable | -0.267862 | 0.792028 | -2.282962 | 0.035575 |
| Oat puree | Liking | Control | -0.742872 | 0.469034 | -1.032215 | 0.318328 |
| Oat puree | Sweetness | Vegetable | -0.246475 | 0.808266 | -1.188258 | 0.251068 |
| Oat puree | Sweetness | Control | 1.014385 | 0.326483 | 0.246532 | 0.808613 |
| Oat puree | Bitterness | Vegetable | 0.325485 | 0.748786 | 0.376198 | 0.711423 |
| Oat puree | Bitterness | Control | -1.012772 | 0.327228 | -0.966948 | 0.348903 |
| Broccoli chopped | Liking | Vegetable | 0.090779 | 0.928729 | 0.256737 | 0.800463 |
| Broccoli chopped | Liking | Control | -1.965819 | 0.068111 | -0.272357 | 0.789061 |
| Broccoli chopped | Sweetness | Vegetable | 0.400243 | 0.693962 | 1.147817 | 0.266941 |
| Broccoli chopped | Sweetness | Control | -0.521896 | 0.609364 | -0.557847 | 0.585179 |
| Broccoli chopped | Bitterness | Vegetable | -1.872189 | 0.078487 | -0.555650 | 0.585689 |
| Broccoli chopped | Bitterness | Control | -0.741145 | 0.470050 | 0.415700 | 0.683516 |
| Kale chopped | Liking | Vegetable | -2.494131 | 0.023225 | -2.182879 | 0.043359 |
| Kale chopped | Liking | Control | 1.176805 | 0.257611 | 1.181696 | 0.255722 |
| Kale chopped | Sweetness | Vegetable | -1.894928 | 0.075239 | -1.536591 | 0.142796 |
| Kale chopped | Sweetness | Control | 1.224352 | 0.239702 | 0.664615 | 0.516385 |
| Kale chopped | Bitterness | Vegetable | 0.154243 | 0.879235 | -0.054209 | 0.957400 |
| Kale chopped | Bitterness | Control | -1.168619 | 0.260795 | -0.906854 | 0.378822 |
| Asparagus chopped | Liking | Vegetable | -1.974615 | 0.064784 | -0.507170 | 0.618551 |
| Asparagus chopped | Liking | Control | 0.614759 | 0.547927 | 0.575050 | 0.573782 |
| Asparagus chopped | Sweetness | Vegetable | 0.888218 | 0.386819 | 0.507551 | 0.618290 |
| Asparagus chopped | Sweetness | Control | 0.186697 | 0.854400 | -0.238964 | 0.814368 |
| Asparagus chopped | Bitterness | Vegetable | -1.298460 | 0.211468 | 0.117122 | 0.908136 |
| Asparagus chopped | Bitterness | Control | -3.023929 | 0.008546 | -3.397736 | 0.003976 |
| Spinach chopped | Liking | Vegetable | 0.061563 | 0.951629 | -1.937821 | 0.069438 |
| Spinach chopped | Liking | Control | 0.463930 | 0.649359 | -0.266207 | 0.793704 |
| Spinach chopped | Sweetness | Vegetable | 0.707743 | 0.488695 | -1.981993 | 0.063885 |
| Spinach chopped | Sweetness | Control | 0.186555 | 0.854509 | -0.867870 | 0.399138 |
| Spinach chopped | Bitterness | Vegetable | -0.585497 | 0.565905 | 2.416622 | 0.027196 |
| Spinach chopped | Bitterness | Control | -1.848296 | 0.084371 | 1.778207 | 0.095641 |
